# Full-length 16S rRNA gene amplicon analysis of human gut microbiota using MinION™ nanopore sequencing confers species-level resolution

**DOI:** 10.1186/s12866-021-02094-5

**Published:** 2021-01-26

**Authors:** Yoshiyuki Matsuo, Shinnosuke Komiya, Yoshiaki Yasumizu, Yuki Yasuoka, Katsura Mizushima, Tomohisa Takagi, Kirill Kryukov, Aisaku Fukuda, Yoshiharu Morimoto, Yuji Naito, Hidetaka Okada, Hidemasa Bono, So Nakagawa, Kiichi Hirota

**Affiliations:** 1 Department of Human Stress Response Science, Institute of Biomedical Science, Kansai Medical University, 2-5-1 Shin-machi, Hirakata, Osaka, 573-1010 Japan; 2 HORAC Grand Front Osaka Clinic, Osaka, Japan; 3 Obstetrics and Gynecology, Kansai Medical University Graduate School of Medicine, Hirakata, Japan; 4 Department of Experimental Immunology, Immunology Frontier Research Center, Osaka University, Osaka, Japan; 5 Faculty of Medicine, Osaka University, Osaka, Japan; 6 Molecular Gastroenterology and Hepatology, Kyoto Prefectural University of Medicine, Kyoto, Japan; 7 Department of Molecular Life Science, Tokai University School of Medicine, Isehara, Japan; 8 Department of Genomics and Evolutionary Biology, National Institute of Genetics, Mishima, Japan; 9 IVF Osaka Clinic, Osaka, Japan; 10 Database Center for Life Science (DBCLS), Research Organization of Information and Systems, Mishima, Japan; 11 Program of Biomedical Science, Graduate School of Integrated Sciences for Life, Hiroshima University, Higashi-Hiroshima, Japan

**Keywords:** 16S rRNA, Gut microbiota, MinION™, Nanopore sequencing

## Abstract

**Background:**

Species-level genetic characterization of complex bacterial communities has important clinical applications in both diagnosis and treatment. Amplicon sequencing of the 16S ribosomal RNA (rRNA) gene has proven to be a powerful strategy for the taxonomic classification of bacteria. This study aims to improve the method for full-length 16S rRNA gene analysis using the nanopore long-read sequencer MinION™. We compared it to the conventional short-read sequencing method in both a mock bacterial community and human fecal samples.

**Results:**

We modified our existing protocol for full-length 16S rRNA gene amplicon sequencing by MinION™. A new strategy for library construction with an optimized primer set overcame PCR-associated bias and enabled taxonomic classification across a broad range of bacterial species. We compared the performance of full-length and short-read 16S rRNA gene amplicon sequencing for the characterization of human gut microbiota with a complex bacterial composition. The relative abundance of dominant bacterial genera was highly similar between full-length and short-read sequencing. At the species level, MinION™ long-read sequencing had better resolution for discriminating between members of particular taxa such as *Bifidobacterium*, allowing an accurate representation of the sample bacterial composition.

**Conclusions:**

Our present microbiome study, comparing the discriminatory power of full-length and short-read sequencing, clearly illustrated the analytical advantage of sequencing the full-length 16S rRNA gene.

**Supplementary Information:**

The online version contains supplementary material available at 10.1186/s12866-021-02094-5.

## Background

Recent advances in DNA sequencing technology have had a revolutionary impact on clinical microbiology [1]. Next-generation sequencing (NGS) technology enables parallel sequencing of DNA on a massive scale to generate vast quantities of accurate data. NGS platforms are now increasingly used in the field of clinical research [2]. Metagenomic sequencing offers numerous advantages over traditional culture-based techniques that have long been the standard test for detecting pathogenic bacteria. This method is particularly useful for characterizing uncultured bacteria and novel pathogens [3].

Among sequence-based bacterial analyses, amplicon sequencing of the 16S ribosomal RNA (rRNA) gene has proven to be a reliable and efficient option for taxonomic classification [4, 5]. The bacterial 16S rRNA gene contains nine variable regions (V1 to V9) that are separated by highly conserved sequences across different taxa. For bacterial identification, the 16S rRNA gene is first amplified by polymerase chain reaction (PCR) with primers annealing to conserved regions and then sequenced. The sequencing data are subjected to bioinformatic analysis in which the variable regions are used to discriminate between bacterial taxa [6].

Since the conventional parallel-type short-read sequencer cannot yield reads covering the full length of the 16S rRNA gene [7], several regions of it have been targeted for sequencing, which often causes ambiguity in taxonomic classification [8]. New sequencing platforms have overcome these technical restrictions, particularly those affecting read length. A prime example is the MinION™ sequencer from Oxford Nanopore Technologies, which is capable of producing long sequences with no theoretical read length limit [9–11]. MinION™ sequencing targets the entire 16S rRNA gene, allowing the identification of bacteria with more accuracy and sensitivity [12, 13]. Furthermore, MinION™ produces sequencing data in real time, which reduces turnaround time for data processing [14, 15]. Currently, the MinION™ sequencing technology has some drawbacks, such as a relatively lower throughput and higher error rates compared to other sequencing platforms [16]. Despite the lower per read accuracy, the nearly unrestricted read length provided by the MinION™ sequencer offers promise in sequence-based bacterial analyses.

Given these features of MinION™ sequencing, we had previously conducted full-length 16S rRNA gene amplicon sequencing analyses using the MinION™ platform coupled to a bioinformatics pipeline [17, 18] (Additional File 1), which allowed us to identify bacterial pathogens with a total analysis time of under two hours [19]. However, we also found that the approach of using the commercial 16S Barcoding Kit (SQK-RAB204) available from Oxford Nanopore Technologies has a limited ability to detect particular taxa such as *Bifidobacterium* [19]. This is probably due to sequence mismatches in the primer used for 16S rRNA gene amplification [20]. Deviations or aberrancies in the *Bifidobacterium* composition in the human gut have been reported in several diseases including obesity, allergy, and inflammatory disorders [21]. Based on their putative health-promoting effects, several strains of *Bifidobacterium* have been utilized as probiotics [22]. Within these contexts, the species-level characterization of *Bifidobacterium* diversity in human gut microbiota is potentially important in clinical practice.

Our 16S rRNA gene sequence analysis using MinION™ has been tested only with pre-characterized mock bacterial DNA and a limited number of pathogenic bacteria from a patient-derived sample [23]. Its applicability to highly complex bacterial communities has not yet been thoroughly investigated. Therefore, in this study we modified our existing protocol for 16S rRNA gene amplicon sequencing by MinION™ and applied it to human gut microbiota with a complex bacterial composition [24], including *Bifidobacterium*, to determine whether full-length 16S rRNA gene sequencing with MinION™ is an effective characterization tool.

## Results

### Classification of the mock bacterial community

**Fig. 1:**
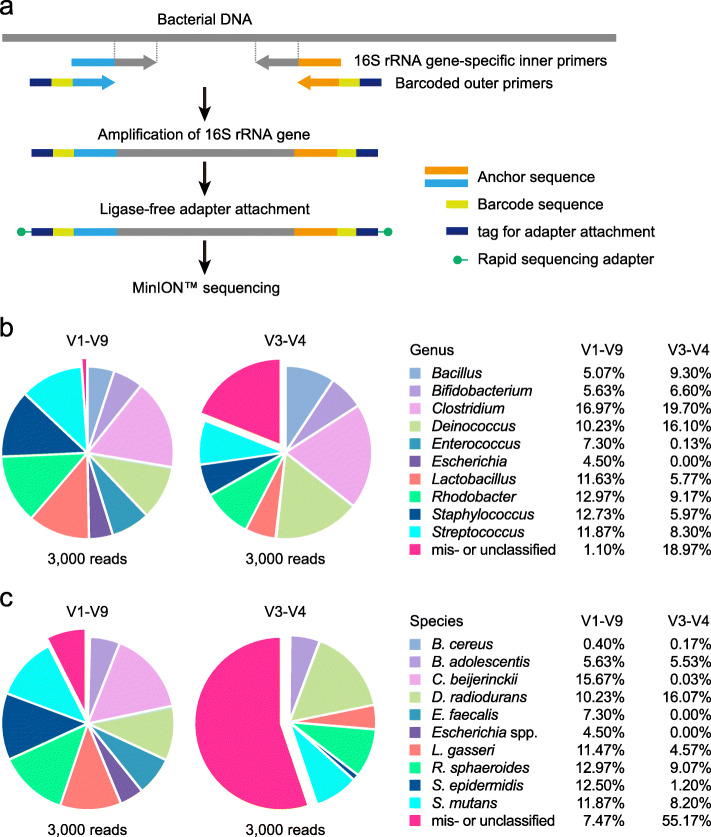
16S rRNA gene sequence analysis using the MinION™ nanopore sequencer. **a** Workflow of 16S rRNA gene amplicon sequencing on the MinION™ platform. Sequencing libraries are generated by the four-primer PCR-based strategy, enabling simplified post-PCR adapter attachment. At the initial stage of PCR, the 16S rRNA gene is amplified with the inner primer pairs. The resulting PCR products are targeted for amplification with the outer primers to introduce the barcode and tag sequences at both ends, to which adapter molecules can be attached in a single-step reaction. **b, c** Taxonomic assignments of a mock community analyzed by MinION™ sequencing. The V1-V9 or V3-V4 region of the 16S rRNA gene was amplified from a pre-characterized mock community sample comprising ten bacterial species and sequenced on the MinION™ platform. Three thousand reads were randomly selected from the processed data set and aligned directly to the reference genome database of 5850 representative bacterial species. The pie charts represent taxonomic profiles at the (b) genus and (c) species levels. Even with the full-length 16S rRNA gene analysis, species-level resolution is not possible for *Bacillus* and *Escherichia*. Slices corresponding to misclassified (assigned to bacteria not present in the mock community) or unclassified (not classified at the given level but placed in a higher taxonomic rank) reads are exploded. The relative abundance (%) of each taxon is shown

The 16S rRNA gene sequence of *Bifidobacterium* has three base mismatches with the 27F forward primer provided in the commercial sequencing kit (16S Barcoding Kit, SQK-RAB204, Oxford Nanopore Technologies; Additional File 2: Supplementary Fig. S1a), which biases amplification toward underrepresentation of *Bifidobacterium* species (Additional File 2: Supplementary Fig. S2, Additional File 3: Supplementary Table S1-S3). To overcome this drawback, we introduced three degenerate bases to the 16S rRNA gene-specific sequences of the primer (Additional File 2: Supplementary Fig. S1b). The competence of the modified primer set was then evaluated by 16S rRNA gene sequence analysis of a ten-species mock community. The V1-V9 region of the 16S rRNA gene was amplified by the four-primer PCR method with the rapid adapter attachment chemistry and sequenced (Fig. 1a). MinION™ sequencing generated 8651 pass reads (Table 1). Following adapter trimming and size selection, 6972 reads (80.6% of pass reads with an average read length of 1473 bp) were retained for bacterial identification. Randomly sampled 3000 reads were aligned against our in-house genome database GenomeSync [18]. Full-length 16S rRNA gene amplicon sequencing with the modified primer set led to the successful identification of all expected bacterial genera, including *Bifidobacterium* (Fig. 1b, Additional File 3: Supplementary Table S4). The dataset was also analyzed by using the different bioinformatics workflows, where the V1-V9 sequences were taxonomically assigned against either the NCBI (Additional File 4) or SILVA 16S rRNA database (Additional File 5). The three alignment tools produced almost similar taxonomic profiles for the V1-V9 MiniON™ reads at the family and genus levels (Additional File 2: Supplementary Fig. S3a, S3b). The majority of reads were correctly classified down to the species level, demonstrating the excellent discriminatory power of the full-length sequencing method for bacterial identification (Fig. 1c, Additional File 2: Supplementary Fig. S3c). *Bacillus* species was an exception in the analysis with both the GenomeSync and NCBI reference database (the SILVA database does not include species level information), and discrimination of *Bacillus cereus* from the closely related species such as *Bacillus anthracis* and *Bacillus thuringiensis* was not achieved (Additional File 4, Additional File 6). Likewise, *Escherichia coli* was not reliably distinguished from *Shigella* and other *Escherichia* species sharing the high 16S rRNA gene sequence similarity to each other [25, 26], and species-level resolution was not possible.

**Table 1:** MinION™ sequencing statistics for the mock community sample

Primers	Pass reads				Trimmed reads		Filtered reads		
	No. of reads	Min (bp)	Avg (bp)	Max (bp)	No. of reads	Avg (bp)	No. of reads	Avg (bp)	Q score
V1-V9	8651	237	1497	3292	8455	1367.1	6972 (80.6%)	1473	9.0
V3-V4	101,372	180	585.7	1977	99,937	451.8	96,189 (94.9%)	454.9	9.2

Min: minimum read length, Avg: average read length, Max: maximum read length, Q score: average Phred quality score. The percentage of reads retained after size filtering is shown in parentheses

We compared the resolution of full-length and short-read 16S rRNA gene amplicon sequencing for the taxonomic classification of bacteria. The V3-V4 region was amplified by four-primer PCR from the ten-species mock community DNA, and the samples were sequenced on MinION™. After removing the adapter/barcode sequences and filtering reads by length, 96,189 reads with an average length of 454.9 bp for downstream analysis were yielded (Table 1). In contrast to full-length sequencing with the highest resolution, a significant number of V3-V4 reads were misclassified or assigned to a higher taxonomic rank (Fig. 1b, c, Additional File 2: Supplementary Fig. S3). The three alignment tools worked with some differences in assigning the V3-V4 sequences. This was notable for alignments against the GenomeSync database, where most V3-V4 reads derived from *Enterococcus faecalis* and *Escherichia coli* were not correctly assigned to each taxon, as more than one species produced the same similarity score for the sequence read queries and the reads were ranked at the lowest common ancestor (Additional File 3: Supplementary Table S5, Additional File 7). We could not classify these bacteria even at the phylum level. The results suggest an analytical problem such as database errors, which may give rise to assigning a distantly related organism to the query sequences. The classifications were not affected by increasing the number of analyzed reads to 10,000 (Additional File 2: Supplementary Fig. S4, Additional File 3: Supplementary Table S6). These classification problems were solved, for the most part, by the V1-V9 long-read sequencing. Thus, regardless of program and database used, the full-length 16S rRNA gene sequencing appeared to give better resolution for bacterial identification.

**Fig. 2:**
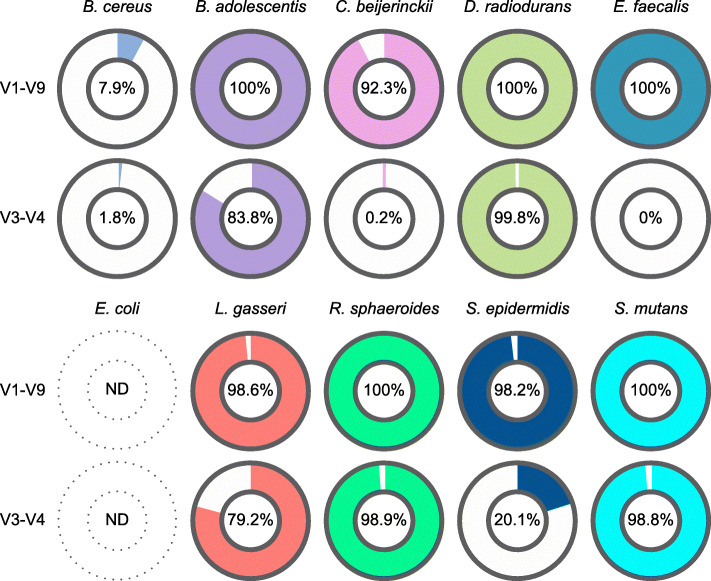
Accurate taxonomic assignment afforded by full-length MinION™ sequencing of the 16S rRNA gene. Classification accuracy compared between full-length (V1-V9) and partial (V3-V4) 16S rRNA gene sequencing data obtained from composition profiling of the ten-species mock community. The donut charts show the proportions of reads correctly assigned to the species constituting the mock community. The percentage of correctly classified reads is shown in the center hole. ND: not determined (species-level resolution is not possible for *Escherichia*)

For seven of the ten bacterial strains constituting the mock community, each subset of V1-V9 sequencing reads classified to the specific genus was assigned with a high degree of accuracy (> 90%) to the corresponding species against both the GenomeSync (Fig. 2) and NCBI 16S rRNA database (Additional File 2: Supplementary Fig. S5). V3-V4 short-read sequencing showed a discriminatory power comparable to that of V1-V9 full-length sequencing in the classification of *Deinococcus*, *Rhodobacter*, and *Streptococcus*. However, the V3-V4 region was not suitable for species-level identification of some genera such as *Clostridium* and *Staphylococcus*. These results suggest a lower resolution of the V3-V4 region for species-level classification, emphasizing the advantage of long-read sequencing for obtaining an accurate representation of the sample bacterial composition.

### Classification of human fecal bacteria

**Fig. 3:**
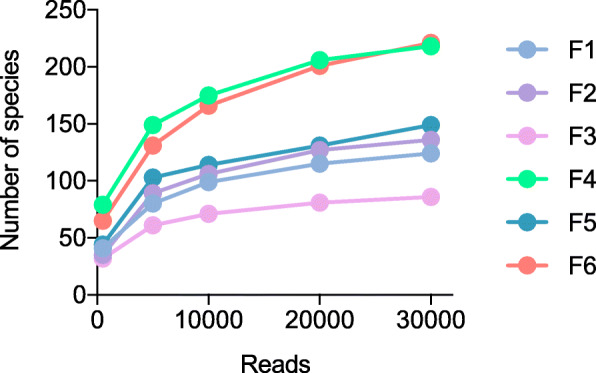
16S rRNA gene sequence analysis of human gut microbiota. Six human fecal samples (F1-F6) were subjected to full-length 16S rRNA gene amplicon sequencing via MinION™. Numbers of detected species are plotted against numbers of reads used for taxonomic classification

We assessed the performance of our full-length 16S rRNA gene amplicon sequencing approach in the context of a highly complex bacterial community. The V1-V9 region was amplified by four-primer PCR from six human fecal samples (F1-F6) and analyzed by MinION™ sequencing. (Table 2). The reads were mapped against the GenomeSync reference database for taxonomic assignment. In Fig. 3, the numbers of species detected are plotted against the numbers of reads analyzed. The curve started to plateau at around 20,000 reads. There was a highly significant correlation between the read numbers 20,000 and 30,000 (Pearson’s correlation coefficient *r* > 0.999, Additional File 8: Supplementary Table S7). Based on these observations, randomly sampled 20,000 reads were used in further analysis to determine the bacterial composition of the human gut.

**Table 2:** Statistics of MinION™ sequencing data for human fecal samples

Sample	Pass reads				Trimmed reads		Filtered reads		
	No. of reads	Min (bp)	Avg (bp)	Max (bp)	No. of reads	Avg (bp)	No. of reads	Avg (bp)	Q score
F1/N V1-V9	104,895	186	1521.1	4549	103,100	1386.4	89,752 (85.6%)	1463.7	9.4
F2/N V1-V9	84,065	169	1393.8	4253	82,458	1259.8	60,326 (71.8%)	1461.4	9.4
F3/N V1-V9	76,968	168	1474.3	4829	74,479	1343.3	60,713 (78.9%)	1465.5	9.4
F4/N V1-V9	114,060	168	1541.7	4836	111,436	1410.6	100,569 (88.2%)	1469.9	9.4
F5/N V1-V9	85,912	177	1536.0	4877	83,038	1405.4	74,168 (86.3%)	1474.2	9.4
F6/N V1-V9	108,938	213	1525.1	4866	106,857	1393.5	93,146 (85.5%)	1467.4	9.4
F1/N V3-V4	52,864	160	568.8	2759	52,283	435.2	48,494 (91.7%)	442.5	9.1
F2/N V3-V4	92,816	174	583.4	2886	91,989	442.8	89,016 (95.9%)	444.7	9.2
F3/N V3-V4	60,200	163	568.5	2062	59,435	434.6	55,706 (92.5%)	441.1	9.2
F4/N V3-V4	83,021	202	578.0	2050	81,734	446.1	77,995 (93.9%)	450.0	9.2
F5/N V3-V4	78,409	167	578.4	1796	76,135	447.8	72,526 (92.5%)	453.1	9.2
F6/N V3-V4	74,931	114	580.3	2246	73,946	446.1	71,330 (95.2%)	449.1	9.2

N: Oxford Nanopore MinION™, Min: minimum read length, Avg: average read length, Max: maximum read length, Q score: average Phred quality score. The percentage of reads retained after size filtering is shown in parentheses

For comparison, amplicon sequencing of the V3-V4 region was also conducted using the MinION™ (Table 2) and the Illumina MiSeq™ platform (Table 3). The processed reads from each data set were allocated to the reference bacterial genome using our bioinformatics pipeline to determine the bacterial compositions (Additional File 9 for V1-V9 MinION™ sequencing, Additional File 10 for V3-V4 MinION™ sequencing, and Additional File 11 for V3-V4 MiSeq™ sequencing). From MiSeq™ sequencing data, the bacterial composition was also analyzed by the operational taxonomic unit (OTU)-based approach using the QIIME 2 (ver. 2019.7) pipeline (Additional File 2: Supplementary Fig. S6, Additional File 12) [27, 28]. Although *Bacteroides* was underrepresented in the OTU-based analysis, the two analytical methods (our bioinformatics pipeline and OTU-based method) produced similar taxonomic profiles in the dominant phylotypes for the MiSeq™ data. This result confirmed the validity of our method for the taxonomic classification of the bacterial community.

**Table 3:** Statistics of MiSeq™ sequencing data for human fecal samples

Sample	Paired reads	Merged reads		Filtered reads		Q Score
	No. of reads	No. of reads	Avg (bp)	No. of reads	Avg (bp)	
F1/I V3-V4	66,242	63,821	449.3	63,778 (96.3%)	449.5	31.4
F2/I V3-V4	68,824	66,640	447.6	66,490 (96.6%)	448.3	32.3
F3/I V3-V4	132,057	128,095	446.9	127,999 (96.9%)	447.1	31.6
F4/I V3-V4	103,532	100,945	451.4	100,853 (97.4%)	451.7	32.8
F5/I V3-V4	72,136	70,521	451	70,459 (97.7%)	451.3	33.2
F6/I V3-V4	52,182	50,907	449	50,841 (97.4%)	449.5	33.2

I: Illumina MiSeq™, Avg: average read length, Q score: average Phred quality score. The percentage of reads retained after size filtering is shown in parentheses

**Fig. 4:**
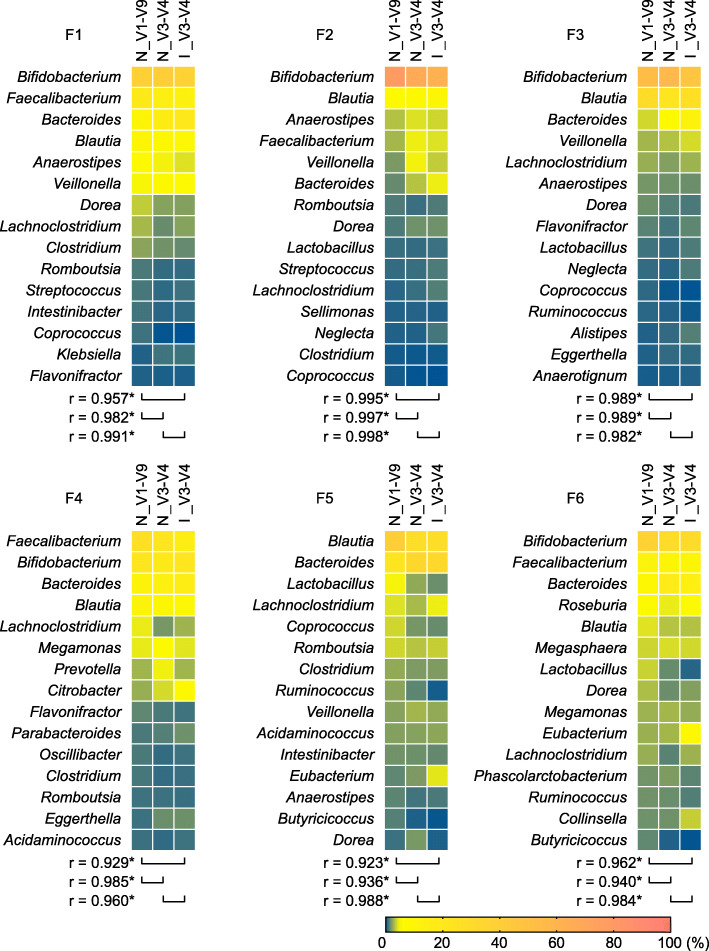
Comparison of taxonomic profiles of human gut microbiota between sequencing methodologies. Six fecal samples (F1-F6) were analyzed by sequencing the entire 16S rRNA gene using MinION™ (N_V1-V9). For comparison, the V3-V4 region was sequenced on MinION™ (N_V3-V4) or MiSeq™ platforms (I_V1-V9). Randomly sampled 20,000 reads from each data set were allocated to the reference genome database of 5850 representative bacterial species. A heat map shows the relative genus abundance (%) of classified reads. The 15 most abundant taxa are shown. The Pearson correlation coefficient (*r*) between sequencing methods was computed. Asterisks indicate significant correlations at *P* < 0.05

The three sequencing methods (V1-V9 MinION™ sequencing, V3-V4 MinION™ sequencing, and V3-V4 MiSeq™ sequencing) revealed similar profiles for the six fecal samples at the genus level (Fig. 4). Statistically significant similarities have been found in the relative genus abundances across these sequencing methods. Thus, at the genus level, V1-V9 full-length MinION™ sequencing exhibited a discriminatory power comparable to that of high-quality short-read sequencing with MiSeq™ technology.

### The species-level taxonomic resolution achieved by full-length sequencing of the 16S rRNA gene using MinION™

**Fig. 5:**
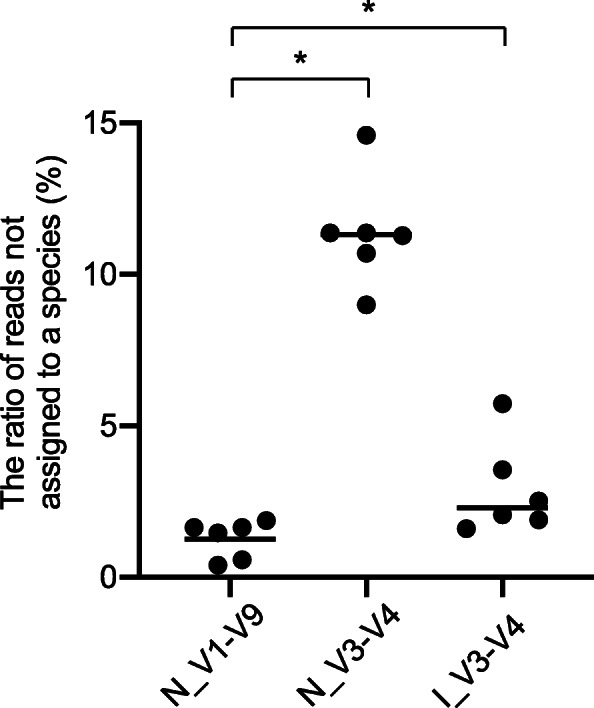
Comparison of taxonomic resolution. The percentages of ambiguous reads not assigned to the species level are plotted for six fecal samples analyzed by MinION™ (N_V1-V9 and N_V3-V4) or MiSeq™ (I_V3-V4). Horizontal bars represent mean values. * *P* < 0.05 (statistically significant)

While genus classification using long versus short reads was relatively comparable, we observed considerable differences across amplified regions in the species-level profiling of human gut microbiota. As shown in Fig. 5, the number of ambiguous reads that were not assigned to species but could be classified at a higher level was significantly greater in the V3-V4 data set in comparison than in the V1-V9 data set. The MinION™ V3-V4 data had the highest proportion of ambiguous reads. In comparison with the V3-V4 reads sequenced on the MiSeq™ platform (Table 3), the MinION™ V3-V4 reads had lower average quality scores (Table 2). The poorer read quality gave rise to assigning multiple species to a query sequence, leading to the increased number of reads not classified at the species level (Additional File 10).

**Fig. 6:**
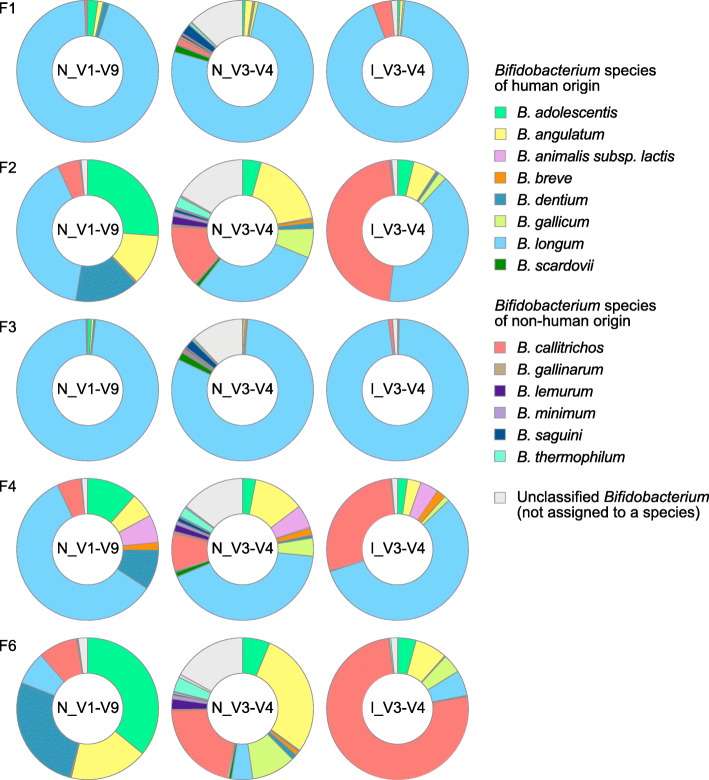
Species composition of *Bifidobacterium* in six fecal samples. MinION™ V1-V9 sequencing confers species-level resolution for bacterial composition profiling. Results obtained by the three sequencing methods are shown. The legends show the 14 most abundant *Bifidobacterium* species

When species compositions of the dominant taxa (*Bifidobacterium*, *Blautia*, and *Bacteroides*) were analyzed, the V1-V9 and V3-V4 sequencing produced comparable results for *Blautia* (Additional File 2: Supplementary Fig. S7, Additional File 8: Supplementary Table S8) and *Bacteroides* genus (Additional File 2: Supplementary Fig. S8, Additional File 8: Supplementary Table S9) in most of the fecal samples. For *Bifidobacterium*, there appeared to be considerable deviations in the relative species abundances depending on the sequencing method used (Fig. 6, Additional File 8: Supplementary Table S10). Notably, most of the *Bifidobacterium* reads generated by V1-V9 MinION™ sequencing were classified into the *Bifidobacterium* species that were isolated from human sources [21, 29]. A significant number of the V3-V4 reads, however, were assigned erroneously to *Bifidobacterium* species of non-human origin in the direct read mapping approach using the relatively shallow GenomeSync reference database (Additional File 2: Supplementary Fig. S9). Consistently, the OTU-based classification analysis for V3-V4 MiSeq™ data using the QIIME 2 pipeline also revealed a lower resolution of short-read sequencing for taxonomic separation of *Bifidobacterium* genus. Except for *Bifidobacterium longum*, *Bifidobacterium* species could not be reliably identified by the V3-V4 sequencing strategy and they were ranked at the genus level (Additional File 2: Supplementary Fig. S10). These results suggest that MinION™ long-read sequencing, which targets the full-length 16S rRNA gene, can provide better resolution for discriminating between members of particular taxa such as *Bifidobacterium*.

## Discussion

16S rRNA gene amplicon sequencing is a powerful strategy for taxonomic classification of bacteria and has been extensively employed for analyzing samples from environmental and clinical sources [5, 30, 31]. We assessed the performance of MinION™ sequencing by comparing the resolution of the V1-V9 and V3-V4 reads for the taxonomic classification of bacteria. Due to the error-prone nature of MinION™ sequencing, the existing OTU-based approach, requiring at least 97% sequence identity threshold, has been considered unsuitable for taxonomic classification of MinION™ reads [32, 33]. Instead, the reads were analyzed by the direct read mapping method that assigns sequences to taxonomic bins based on the similarity to a reference database [14, 15]. Long-read MinION™ sequencing with the optimized primer set successfully identified *Bifidobacterium* species leading to a better representation of the species composition of the mock community. For improving the classification results, the reads were filtered by length to eliminate those outside the expected size range. Typically, extremely short reads possess only one primer-binding site, suggesting that they are derived from incomplete sequencing. There also exist unexpectedly longer reads with a continuous sequence structure in which two 16S rRNA gene amplicons are linked end-to-end. Because these reads can potentially result in unclassified reads or misclassification, they were eliminated before alignment to the reference sequences of the bacterial genome.

We also modified library construction for MinION™ sequencing with a four-primer PCR strategy, which enabled ligase-free adapter attachment to occur in a single-step reaction. The four-primer PCR generates amplicons with particular chemical modifications at the 5′ ends to which adapter molecules can be attached non-enzymatically. Unlike the ligation-based approach, the PCR products amplified by the four-primer method are subjected directly to the adapter attachment reaction without repairing their 5′ ends, substantially reducing the time required for sample preparation. Furthermore, because the protocol is free of Nanopore’s transposase-based technology (e.g. Rapid Sequencing Kit, SQK-RAD004) that cleaves DNA molecules to produce chemically modified ends for library construction, the PCR products are kept intact, enabling sequencing of the entire amplified region. Thus, the four-primer PCR-based method allowed us to perform amplicon sequencing on the MinION™ platform with user-defined arbitrary primer pairs, taking advantage of the rapid adapter attachment chemistry. This method can be applied to a wide range of sequence-based analyses, including detection of functional genetic markers like antimicrobial resistance genes and identification of genetic variations in targeted loci [11, 34, 35].

Our present microbiome study, comparing the discriminatory power of the V1-V9 and V3-V4 reads sequenced on the MinION™ platform, clearly illustrated the advantage of sequencing the entire 16S rRNA gene. The full-length 16S rRNA gene sequencing provided better resolution than short-read sequencing for discriminating between members of certain bacterial taxa, including *Bifidobacterium*, *Clostridium*, *Enterococcus*, and *Staphylococcus*. Consistently, comprehensive microbiome studies using a sequencing data set consisting of different regions of the 16S rRNA gene have shown that the choice of the regions to be sequenced substantially affects the classification results, and some bacterial species are identified only by sequencing the entire 16S rRNA gene [6, 36, 37]. It is important to note, however, that even full-length 16S rRNA gene analysis fails to discriminate some closely related species such as members of *Bacillus cereus* group and *Escherichia*, indicating the limitations of the 16S rRNA gene amplicon sequencing alone in species allocation. Long read sequencing targeting other phylogenetic markers may be an alternative to 16S rRNA gene amplicon sequencing and provide better resolution for bacterial identification.

In the analysis of the human fecal samples, we used the taxonomic resolution of the V3-V4 region sequenced with MiSeq™, which generates highly accurate reads, as a benchmark for the taxonomic resolution of the full-length 16S rRNA gene sequenced with MinION™. The relative abundance of dominant bacterial taxa was highly similar at the genus level between full-length MinION™ and short-read MiSeq™ sequencing. Despite the lower read quality, the full-length sequencing by MinION™ enabled reliable identification of bacterial genera with an accuracy comparable to MiSeq™ technology. The MiSeq™ platform enables 16S rRNA gene sequencing on a massive scale with reduced cost (approximately 20 USD per sample). Considering a low equipment price (1000 USD) and an affordable per-run cost (approximately 50 USD per sample), the MinION™ sequencer could be a viable option for practical applications in clinical microbiology.

Our study has some limitations. The use of the GenomeSync reference database with a limited number of sequences could not allow a comprehensive survey of bacterial communities. In addition to the database size, the analysis methods may also influence the identification results. In our bioinformatics pipeline, a query sequence is directly mapped to the reference genome and assigned to a specific taxon according to the alignment score. When more than one taxon is identified, the read is assigned to the lowest common ancestor. It seemed that this approach worked well for the V1-V9 sequencing with better resolution, and the reads had a higher probability of being assigned down to the species level. However, as evident in the classification of *Enterococcus* and *Escherichia*, the V3-V4 sequence alignment against GenomeSync produced a higher proportion of ambiguous identification. In the case of bifidobacteria, non-human species were erroneously identified by the V3-V4 short-read sequencing in the analysis of human fecal samples. Such improbable errors were not observed in QIIME2 analysis, in which species-level calls were not made and the reads were ranked at the genus level as more specific classification was impossible. Due to the low discriminatory power of the V3-V4 region, the direct read mapping against a flawed or incomplete reference database may potentially give rise to assigning an unrelated organism marked as a top hit. Since the taxonomic assignment is a critical step for analyzing the bacterial diversity and community composition, the reference database quality and the alignment algorithm must be further evaluated for each sequencing data set.

## Conclusions

Our modified protocol for 16S rRNA gene amplicon sequencing overcame known limitations, such as the primer-associated bias toward the underrepresentation of *Bifidobacterium*, and enabled taxonomic classification across a broad range of bacterial species. Benchmarking with MiSeq™ sequencing technology demonstrated the analytical advantage of sequencing the full-length 16S rRNA gene with MinION™, which could counteract the lower sequence accuracy and provide better resolution. With the recent progress in nanopore sequencing chemistry and base-calling algorithms, sequencing accuracy is continuously improving [38, 39]. This will soon enable us to exploit the full potential of MinION™ long-read sequencing technology. High-quality long sequences will allow better discrimination between closely related species in sequence-based bacterial analyses.

## Methods

### Mock bacterial community DNA

A mixture of bacterial DNA (10 Strain Even Mix Genomic Material, MSA-1000) was obtained from the American Type Culture Collection (ATCC, Manassas, VA, USA), comprising genomic DNA prepared from the following ten bacterial strains: *Bacillus cereus* (ATCC 10987), *Bifidobacterium adolescentis* (ATCC 15703), *Clostridium beijerinckii* (ATCC 35702), *Deinococcus radiodurans* (ATCC BAA­816), *Enterococcus faecalis* (ATCC 47077), *Escherichia coli* (ATCC 700926), *Lactobacillus gasseri* (ATCC 33323), *Rhodobacter sphaeroides* (ATCC 17029), *Staphylococcus epidermidis* (ATCC 12228), and *Streptococcus mutans* (ATCC 700610).

### Fecal DNA

DNA was extracted from six human fecal samples using the NucleoSpin® Microbial DNA Kit (Macherey-Nagel, Düren, Germany), as described previously [40]. Briefly, human feces stored using the Feces Collection Kit (Techno Suruga Lab, Shizuoka, Japan) were subjected to mechanical disruption by bead-beating, and DNA was isolated using silica membrane spin columns. Extracted DNA was purified with the Agencourt AMPure® XP (Beckman Coulter, Brea, CA, USA).

### 16S rRNA gene sequencing on the MinION™ platform

Four-primer PCR with rapid adapter attachment chemistry generated 16S rRNA gene amplicons with modified 5′ ends for simplified post-PCR adapter attachment following the manufacturer’s instructions with slight modifications. For amplification of the V1-V9 region of the 16S rRNA gene, the following inner primers were used, with 16S rRNA gene-specific sequences underlined: forward primer (S-D-Bact-0008-c-S-20 [41]) with anchor sequence 5′-TTTCTGTTGGTGCTGATATTGCAGRGTTYGATYMTGGCTCAG-3′ and reverse primer (1492R) with anchor sequence 5′-ACTTGCCTGTCGCTCTATCTTCCGGYTACCTTGTTACGACTT-3′. For amplification of the V3-V4 region, the following inner primers were used, with 16S rRNA gene-specific sequences underlined: 341F with anchor sequence 5′-TTTCTGTTGGTGCTGATATTGCCCTACGGGNGGCWGCAG-3′ and 806R with anchor sequence 5′-ACTTGCCTGTCGCTCTATCTTCGGACTACHVGGGTWTCTAAT-3′. PCR amplification of 16S rRNA genes was conducted using the KAPA2G™ Robust HotStart ReadyMix PCR Kit (Kapa Biosystems, Wilmington, MA, USA) in a total volume of 25 μl containing inner primer pairs (50 nM each) and the barcoded outer primer mixture (3%) from the PCR Barcoding Kit (SQK-PBK004; Oxford Nanopore Technologies, Oxford, UK). Amplification was performed with the following PCR conditions: initial denaturation at 95 °C for 3 min, 5 cycles of 95 °C for 15 s, 55 °C for 15 s, and 72 °C for 30 s, 30 cycles of 95 °C for 15 s, 62 °C for 15 s, and 72 °C for 30 s, followed by a final extension at 72 °C for 1 min. Amplified DNA was purified using AMPure® XP (Beckman Coulter) and quantified by a NanoDrop® 1000 (Thermo Fischer Scientific, Waltham, MA, USA). A total of 100 ng of DNA was incubated with 1 μl of Rapid Adapter at room temperature for 5 min. The prepared DNA library (11 μl) was mixed with 34 μl of Sequencing Buffer, 25.5 μl of Loading Beads, and 4.5 μl of water, loaded onto the R9.4 flow cell (FLO-MIN106; Oxford Nanopore Technologies), and sequenced on the MinION™ Mk1B. MINKNOW software ver. 1.11.5 (Oxford Nanopore Technologies) was used for data acquisition.

### 16S rRNA gene sequencing on the MiSeq™ platform

Sequencing libraries were constructed as described previously [40]. Briefly, the V3-V4 regions of the 16S rRNA gene were amplified using a 16S (V3–V4) Metagenomic Library Construction Kit for NGS (Takara Bio Inc., Kusatsu, Japan). The following primers were used (16S rRNA gene-specific sequences are underlined): 341F with overhang adapter 5′-TCGTCGGCAGCGTCAGATGTGTATAAGAGACAGCCTACGGGNGGCWGCAG-3′ and 806R with overhang adapter 5′-GTCTCGTGGGCTCGGAGATGTGTATAAGAGACAGGGACTACHVGGGTWTCTAAT-3′. The second PCR was performed using the Nextera® XT Index Kit (Illumina, San Diego, CA, USA) for sample multiplexing with index adapters. The libraries were sequenced on the MiSeq™ platform using the MiSeq™ Reagent Kit v3 (2 × 250 bp; Illumina).

### Bioinformatics analysis

Albacore software ver. 2.3.4 (Oxford Nanopore Technologies) was used for basecalling the MinION™ sequencing data (FAST5 files) to generate pass reads (FASTQ format) with a mean quality score > 7. The adapter and barcode sequences were trimmed using the EPI2ME Fastq Barcoding workflow ver. 3.10.4 (Oxford Nanopore Technologies). The reads were filtered by size using SeqKit software ver. 0.10.0 [42], retaining 1300–1950 bp sequences for the V1-V9 region and 350–600 bp sequences for the V3-V4 region, based on the size distribution of 16S rRNA gene sequences in the SILVA database ver. 132 [43, 44]. The average Phred quality score was assessed using NanoPlot ver. 1.27.0 [45]. The processed reads from each set were analyzed using our bioinformatics pipeline [17], as described previously [14, 15]. Briefly, FASTQ files were converted to FASTA files. Simple repetitive sequences were masked using the TANTAN program ver. 18 with default parameters [46]. To remove reads derived from human DNA, we searched each read against the human genome (GRCh38) using minimap2 ver. 2.14 with a map-ont-option [47]. Then, unmatched reads were regarded as reads derived from bacteria. For each read, a minimap2 search with 5850 representative bacterial genome sequences stored in the GenomeSync database (Additional File 1) [18] was performed. For each read, the species showing the highest minimap2 score were assigned to the query sequence. When more than one species showed the same similarity score, the reads were classified at any higher taxonomic rank covering all the identified species. Taxa were determined based on the NCBI taxonomy database [48]. Low-abundance taxa with less than 0.01% of total reads were discarded from the analysis.

### Statistical analyses

Differences between groups were evaluated by one-way analysis of variance (ANOVA) followed by Dunnett’s test for multiple comparisons. The Pearson correlation coefficient was computed to compare the bacterial compositions analyzed by different sequencing methods. Statistical significance was defined by a *P*-value < 0.05. Statistical analyses were performed with Prism8 (GraphPad Software, Inc. La Jolla, CA, USA).

## Supplementary Information

**Additional file 1.** Representative bacterial genomes stored in the GenomeSync database.

**Additional file 2 Fig. S1.** Sequence heterogeneities of the 27F primer-annealing site in 16S rRNA genes. **Fig. S2.** Evaluation of 16S rRNA PCR primers for identification of bacterial species. **Fig. S3.** Classification results of a mock community analyzed by the different bioinformatics workflows. **Fig. S4.** Effect of read number on taxonomic classification. **Fig. S5.** Species composition of a mock community analyzed by FASTQ 16S workflow. **Fig. S6.** Rarefaction curves of observed OTUs in V3-V4 16S rRNA gene amplicon sequencing of human fecal samples using the MiSeq™ platform. **Fig. S7.** Species composition of *Blautia* in human fecal samples. **Fig. S8.** Species composition of *Bacteroides* in human fecal samples. **Fig. S9.** Deviations in the relative abundances of *Bifidobacterium* species in human fecal samples. **Fig. S10.** Comparison of species composition of fecal *Bifidobacterium* between classification methods.

**Additional file 3 Tables S1-S6.** Taxonomic assignment of the mock community analyzed by MinION™ sequencing.

**Additional file 4.** Mock community data analysis using FASTQ 16S workflow and the NCBI bacterial 16S rRNA database.

**Additional file 5.** Mock community data analysis using Kraken 2 and the SILVA 16S rRNA reference database.

**Additional file 6.** Alignment search results for V1-V9 amplicon sequencing of the mock community.

**Additional file 7.** Alignment search results for V3-V4 amplicon sequencing of the mock community.

**Additional file 8 Table S7.** Correlations between numbers of reads and numbers of detected species in 16S rRNA gene sequencing of human fecal samples. **Table S8.** Comparison of species composition of fecal *Blautia* between sequencing methods. **Table S9.** Comparison of species composition of fecal *Bacteroides* between sequencing methods. **Table S10.** Comparison of species composition of fecal *Bifidobacterium* between sequencing methods.

**Additional file 9.** Taxonomic profile of human fecal samples from MinION™ sequencing (amplicons: V1-V9).

**Additional file 10.** Taxonomic profile of human fecal samples from MinION™ sequencing (amplicons: V3-V4).

**Additional file 11.** Taxonomic profile of human fecal samples from MiSeq™ sequencing (amplicons: V3-V4).

**Additional file 12.** Taxonomic profiles of human fecal samples from MiSeq™ sequencing (amplicons: V3-V4, taxonomic classification by OTU-based analysis using the QIIME 2 pipeline).

## Data Availability

The sequence datasets supporting the conclusions of this article are available in the DDBJ DRA database (https://www.ddbj.nig.ac.jp/dra/index-e.html) under accession numbers DRR225043 to DRR225065.

## Abbreviations

NGS: next-generation sequencing
OTU: operational taxonomic unit
PCR: polymerase chain reaction
rRNA: ribosomal RNA

## References

[1] Chiu CY, Miller SA (2019). Clinical metagenomics. Nat Rev Genet.

[2] Loman NJ, Misra RV, Dallman TJ, Constantinidou C, Gharbia SE, Wain J (2012). Performance comparison of benchtop high-throughput sequencing platforms. Nat Biotechnol.

[3] Didelot X, Bowden R, Wilson DJ, Peto TEA, Crook DW (2012). Transforming clinical microbiology with bacterial genome sequencing. Nat Rev Genet..

[4] Clarridge JE, 3rd. Impact of 16S rRNA gene sequence analysis for identification of bacteria on clinical microbiology and infectious diseases. Clin Microbiol Rev. 2004;17(4):840–62, table of contents. 10.1128/CMR.17.4.840-862.2004 PMC523561 15489351

[5] Langille MG, Zaneveld J, Caporaso JG, McDonald D, Knights D, Reyes JA (2013). Predictive functional profiling of microbial communities using 16S rRNA marker gene sequences. Nat Biotechnol.

[6] Johnson JS, Spakowicz DJ, Hong BY, Petersen LM, Demkowicz P, Chen L (2019). Evaluation of 16S rRNA gene sequencing for species and strain-level microbiome analysis. Nat Commun.

[7] Ravi RK, Walton K, Khosroheidari M (1706). MiSeq: a next generation sequencing platform for genomic analysis. Methods Mol Biol.

[8] Kuczynski J, Lauber CL, Walters WA, Parfrey LW, Clemente JC, Gevers D (2011). Experimental and analytical tools for studying the human microbiome. Nat Rev Genet..

[9] Leggett RM, Clark MD (2017). A world of opportunities with nanopore sequencing. J Exp Bot.

[10] Quick J, Ashton P, Calus S, Chatt C, Gossain S, Hawker J (2015). Rapid draft sequencing and real-time nanopore sequencing in a hospital outbreak of salmonella. Genome Biol.

[11] Leggett RM, Alcon-Giner C, Heavens D, Caim S, Brook TC, Kujawska M (2020). Rapid MinION profiling of preterm microbiota and antimicrobial-resistant pathogens. Nat Microbiol.

[12] Benitez-Paez A, Sanz Y (2017). Multi-locus and long amplicon sequencing approach to study microbial diversity at species level using the MinION portable nanopore sequencer. Gigascience..

[13] Shin H, Lee E, Shin J, Ko SR, Oh HS, Ahn CY (2018). Elucidation of the bacterial communities associated with the harmful microalgae Alexandrium tamarense and Cochlodinium polykrikoides using nanopore sequencing. Sci Rep.

[14] Mitsuhashi S, Kryukov K, Nakagawa S, Takeuchi JS, Shiraishi Y, Asano K (2017). A portable system for rapid bacterial composition analysis using a nanopore-based sequencer and laptop computer. Sci Rep.

[15] Nakagawa S, Inoue S, Kryukov K, Yamagishi J, Ohno A, Hayashida K (2019). Rapid sequencing-based diagnosis of infectious bacterial species from meningitis patients in Zambia. Clin Transl Immunology.

[16] Kono N, Arakawa K (2019). Nanopore sequencing: review of potential applications in functional genomics. Develop Growth Differ.

[17] Genome Search Toolkit. http://kirill-kryukov.com/study/tools/gstk/

[18] GenomeSync. http://genomesync.org

[19] Kai S, Matsuo Y, Nakagawa S, Kryukov K, Matsukawa S, Tanaka H (2019). Rapid bacterial identification by direct PCR amplification of 16S rRNA genes using the MinION nanopore sequencer. FEBS Open Bio.

[20] Kim SW, Suda W, Kim S, Oshima K, Fukuda S, Ohno H (2013). Robustness of gut microbiota of healthy adults in response to probiotic intervention revealed by high-throughput pyrosequencing. DNA Res.

[21] Arboleya S, Watkins C, Stanton C, Ross RP (2016). Gut Bifidobacteria populations in human health and aging. Front Microbiol.

[22] Backhed F, Ley RE, Sonnenburg JL, Peterson DA, Gordon JI (2005). Host-bacterial mutualism in the human intestine. Science..

[23] Tanaka H, Matsuo Y, Nakagawa S, Nishi K, Okamoto A, Kai S (2019). Real-time diagnostic analysis of MinION-based metagenomic sequencing in clinical microbiology evaluation: a case report. JA Clin Rep.

[24] Arumugam M, Raes J, Pelletier E, Le Paslier D, Yamada T, Mende DR (2011). Enterotypes of the human gut microbiome. Nature..

[25] Devanga Ragupathi NK, Muthuirulandi Sethuvel DP, Inbanathan FY, Veeraraghavan B (2018). Accurate differentiation of Escherichia coli and Shigella serogroups: challenges and strategies. New Microbes New Infect.

[26] Lukjancenko O, Wassenaar TM, Ussery DW (2010). Comparison of 61 sequenced Escherichia coli genomes. Microb Ecol.

[27] Caporaso JG, Kuczynski J, Stombaugh J, Bittinger K, Bushman FD, Costello EK (2010). QIIME allows analysis of high-throughput community sequencing data. Nat Methods.

[28] Bolyen E, Rideout JR, Dillon MR, Bokulich NA, Abnet CC, Al-Ghalith GA (2019). Reproducible, interactive, scalable and extensible microbiome data science using QIIME 2. Nat Biotechnol.

[29] Milani C, Lugli GA, Duranti S, Turroni F, Bottacini F, Mangifesta M (2014). Genomic encyclopedia of type strains of the genus Bifidobacterium. Appl Environ Microbiol.

[30] Human Microbiome Project C (2012). A framework for human microbiome research. Nature..

[31] Srinivasan R, Karaoz U, Volegova M, MacKichan J, Kato-Maeda M, Miller S (2015). Use of 16S rRNA gene for identification of a broad range of clinically relevant bacterial pathogens. PLoS One.

[32] Santos A, van Aerle R, Barrientos L, Martinez-Urtaza J (2020). Computational methods for 16S metabarcoding studies using Nanopore sequencing data. Comput Struct Biotechnol J.

[33] Ma X, Stachler E, Bibby K. Evaluation of Oxford Nanopore MinION™ Sequencing for 16S rRNA Microbiome Characterization. bioRxiv. 2017 10.1101/099960

[34] Quick J, Grubaugh ND, Pullan ST, Claro IM, Smith AD, Gangavarapu K (2017). Multiplex PCR method for MinION and Illumina sequencing of Zika and other virus genomes directly from clinical samples. Nat Protoc.

[35] Cornelis S, Gansemans Y, Deleye L, Deforce D, Van Nieuwerburgh F (2017). Forensic SNP genotyping using Nanopore MinION sequencing. Sci Rep.

[36] Bukin YS, Galachyants YP, Morozov IV, Bukin SV, Zakharenko AS, Zemskaya TI (2019). The effect of 16S rRNA region choice on bacterial community metabarcoding results. Sci Data.

[37] Shin J, Lee S, Go MJ, Lee SY, Kim SC, Lee CH (2016). Analysis of the mouse gut microbiome using full-length 16S rRNA amplicon sequencing. Sci Rep.

[38] Magi A, Semeraro R, Mingrino A, Giusti B, D'Aurizio R (2018). Nanopore sequencing data analysis: state of the art, applications and challenges. Brief Bioinform.

[39] Rang FJ, Kloosterman WP, de Ridder J (2018). From squiggle to basepair: computational approaches for improving nanopore sequencing read accuracy. Genome Biol.

[40] Takagi T, Naito Y, Inoue R, Kashiwagi S, Uchiyama K, Mizushima K (2019). Differences in gut microbiota associated with age, sex, and stool consistency in healthy Japanese subjects. J Gastroenterol.

[41] Klindworth A, Pruesse E, Schweer T, Peplies J, Quast C, Horn M (2013). Evaluation of general 16S ribosomal RNA gene PCR primers for classical and next-generation sequencing-based diversity studies. Nucleic Acids Res.

[42] Shen W, Le S, Li Y, Hu F (2016). SeqKit: a cross-platform and ultrafast toolkit for FASTA/Q file manipulation. PLoS One.

[43] Quast C, Pruesse E, Yilmaz P, Gerken J, Schweer T, Yarza P (2013). The SILVA ribosomal RNA gene database project: improved data processing and web-based tools. Nucleic Acids Res.

[44] Silva reference files. https://mothur.org/wiki/silva_reference_files/

[45] De Coster W, D'Hert S, Schultz DT, Cruts M, Van Broeckhoven C (2018). NanoPack: visualizing and processing long-read sequencing data. Bioinformatics..

[46] Frith MC (2011). A new repeat-masking method enables specific detection of homologous sequences. Nucleic Acids Res.

[47] Li H (2018). Minimap2: pairwise alignment for nucleotide sequences. Bioinformatics..

[48] Federhen S (2012). The NCBI taxonomy database. Nucleic Acids Res.

