# Supplementary material for: Full-length 16S rRNA gene amplicon analysis of human gut microbiota using MinION™ nanopore sequencing confers species-level resolution
Source: BMC Microbiol. 2021 Jan 26;21:35. doi: 10.1186/s12866-021-02094-5 (PMC7836573; doi:10.1186/s12866-021-02094-5)
Supplement: Supplementary file 2 — Additional file 2 Fig. S1. Sequence heterogeneities of the 27F primer-annealing site in 16S rRNA genes. Fig. S2. Evaluation of 16S rRNA PCR primers for identification of bacterial species. Fig. S3. Classification results of a mock community analyzed by the different bioinformatics workflows. Fig. S4. Effect of read number on taxonomic classification. Fig. S5. Species composition of a mock community analyzed by FASTQ 16S workflow. Fig. S6. Rarefaction curves of observed OTUs in V3-V4 16S rRNA gene amplicon sequencing of human fecal samples using the MiSeq™ platform. Fig. S7. Species composition of Blautia in human fecal samples. Fig. S8. Species composition of Bacteroides in human fecal samples. Fig. S9. Deviations in the relative abundances of Bifidobacterium species in human fecal samples. Fig. S10. Comparison of species composition of fecal Bifidobacterium between classification methods. [file 12866_2021_2094_MOESM2_ESM.pdf]

a

Species	Sequence
Bacillus cereus	AGAGTTTGATCCTGGCTCAG
Bifidobacterium adolescentis	AGGGTTCGATTCTGGCTCAG
Clostridium beijerinckii	AGAGTTTGATCCTGGCTCAG
Deinococcus radiodurans	AGAGTTTGATCCTGGCTCAG
Enterococcus faecalis	AGAGTTTGATCCTGGCTCAG
Escherichia coli	AGAGTTTGATCATGGCTCAG
Lactobacillus gasseri	AGAGTTTGATCCTGGCTCAG
Rhodobacter sphaeroides	AGAGTTTGATCCTGGCTCAG
Staphylococcus epidermidis	AGAGTTTGATCCTGGCTCAG
Streptococcus mutans	AGAGTTTGATCCTGGCTCAG

b

Forward primer	Sequence
Original 27F (ONT, SQK-RAB204)	AGAGTTTGATCMTGGCTCAG
This study (S-D-Bact-0008-c-S-20)	AGRGTTYGATYMTGGCTCAG
27F-I	AGAGTTTGATCATGGCTCAG
27F-II	AGGGTTCGATTCTGGCTCAG
27F-III	AGAGTTTGATCCTGGCTCAG

Supplementary Fig. S1 Sequence heterogeneities of the 27F primer-annealing site in 16S rRNA genes. **a** Multiple sequence alignment for the ten bacterial species constituting the mock community. Variable nucleotides are shaded. Three mismatched bases in *Bifidobacterium* are shown in red. **b** 16S rRNA gene-specific sequences of the original 27F (Oxford Nanopore Technologies, ONT) and the primers used in this study. 27F-**I**, 27F-**II**, and 27F-**III** correspond to the sequences for *Escherichia coli*, *Bifidobacterium adolescentis*, and the other eight species, respectively.

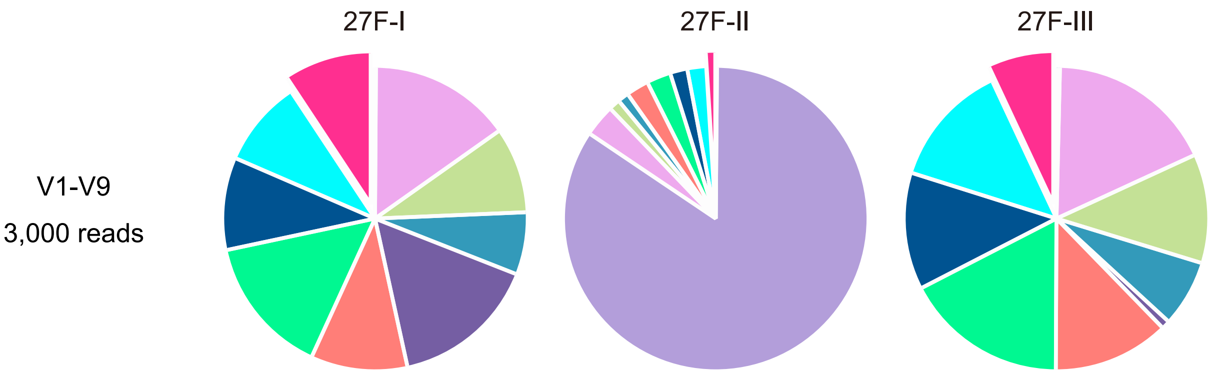

Species	27F-I	27F-II	27F-III
Bacillus cereus	0.10%	0.13%	0.37%
Bifidobacterium adolescentis	0.00%	84.30%	0.00%
Clostridium beijerinckii	15.10%	3.40%	17.77%
Deinococcus radiodurans	9.13%	1.20%	11.60%
Enterococcus faecalis	6.63%	1.17%	7.10%
Escherichia spp.	15.60%	0.00%	0.90%
Lactobacillus gasseri	10.27%	2.40%	12.33%
Rhodobacter sphaeroides	14.83%	2.57%	17.37%
Staphylococcus epidermidis	9.83%	1.83%	12.43%
Streptococcus mutans	9.20%	2.00%	13.17%
mis- or unclassified	9.30%	1.00%	6.97%

Supplementary Fig. S2 Evaluation of 16S rRNA PCR primers for identification of bacterial species. The V1-V9 region of the 16S rRNA gene was amplified from the ten-species mock community sample using the indicated 27F variant and 1492R primer and sequenced on MinION™. The pie charts represent taxonomic profiles at the species level, and the relative abundances (%) of each taxon are shown.

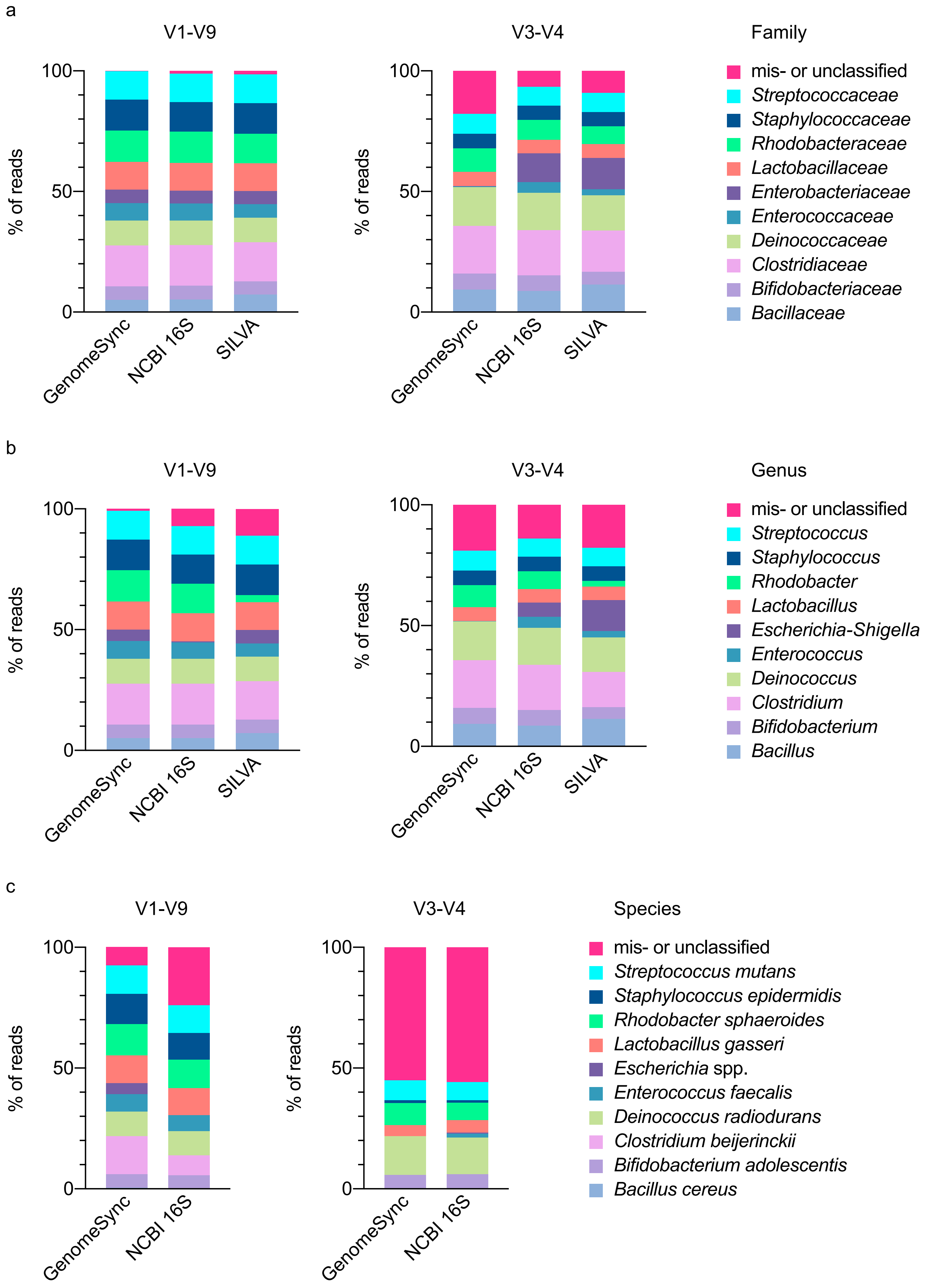

Supplementary Fig. S3 Classification results of a mock community analyzed by the different bioinformatics workflows. The V1-V9 or V3-V4 MinION™ sequencing reads from a 10-species mock community sample were taxonomically assigned against the GenomeSync (showing the same results as in Fig. 1b and 1c), NCBI, and SILVA reference databases. Three thousand sequences were used and the relative abundances (%) of each taxon at the (a) family, (b) genus, and (c) species levels are shown (the SILVA database does not include species level information). For the genera *Escherichia* and *Shigella*, the SILVA database uses the combined genus name *Escherichia-Shigella*, which has been applied to all analyses.

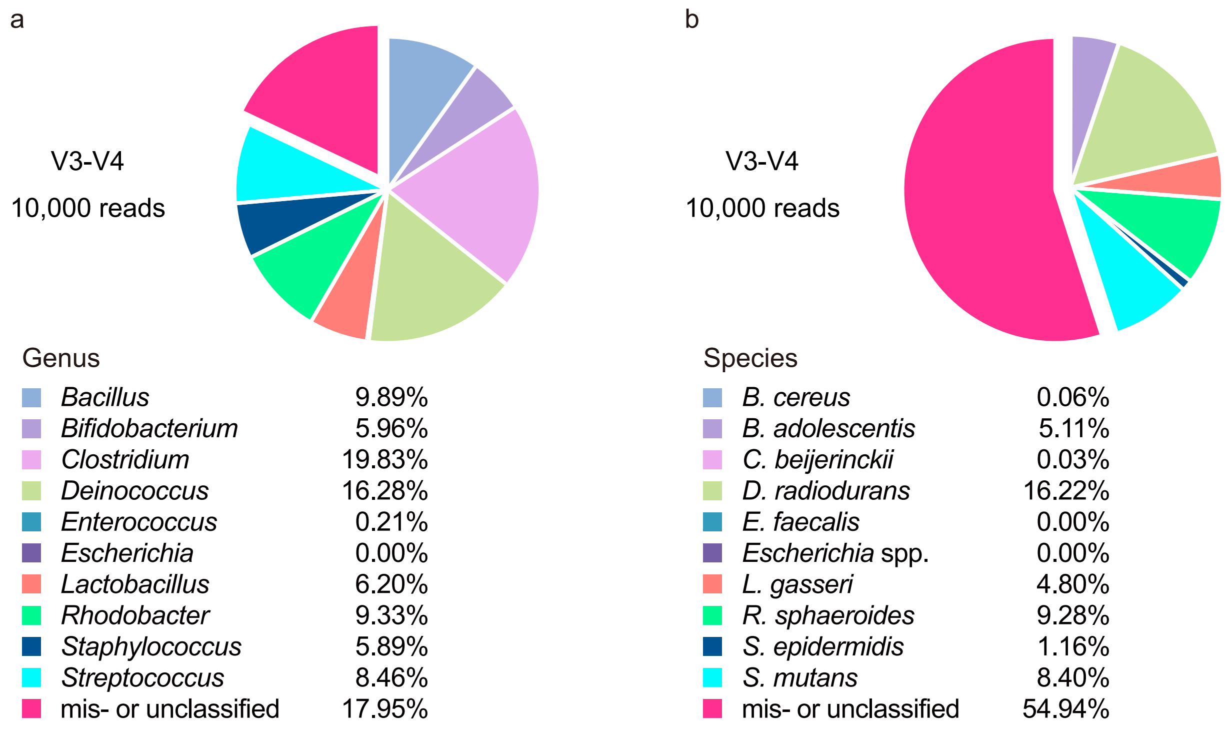

Supplementary Fig. S4 Effect of read number on taxonomic classification.

a, b The V3-V4 region of the 16S rRNA gene was amplified from a 10-species mock community sample and sequenced on MinION™ as in Fig. 1. Ten thousand reads were used for taxonomic profiling. The pie charts represent taxonomic profiles at the (a) genus and (b) species level. The relative abundances (%) of each taxon are shown.

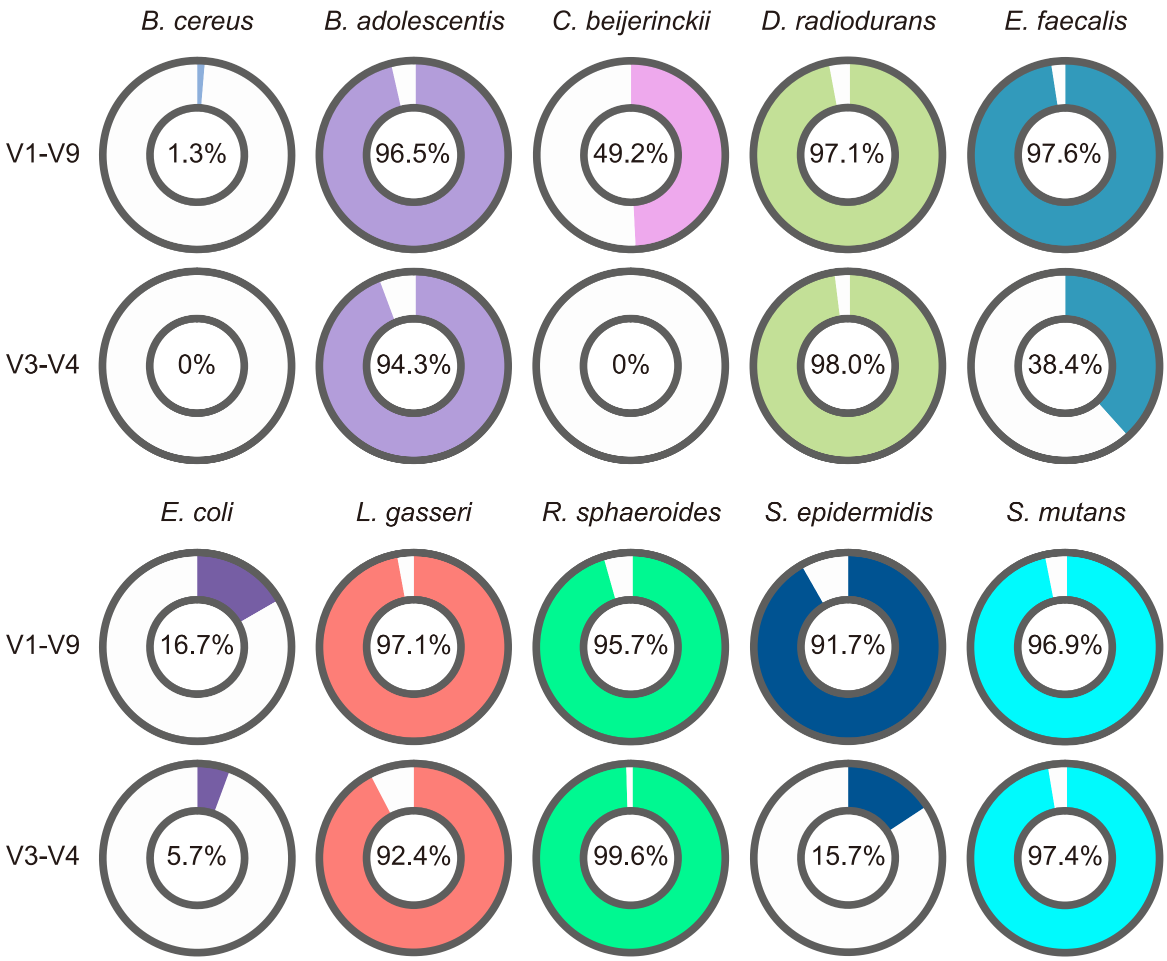

Supplementary Fig. S5 Species composition of a mock community analyzed by FASTQ 16S workflow. The V1-V9 or V3-V4 MinION™ sequencing reads from a 10-species mock community sample were aligned against the NCBI bacterial 16S rRNA gene database using EPI2ME FASTQ 16S workflow. The donut charts show the proportions of reads correctly assigned to the species constituting the mock community.

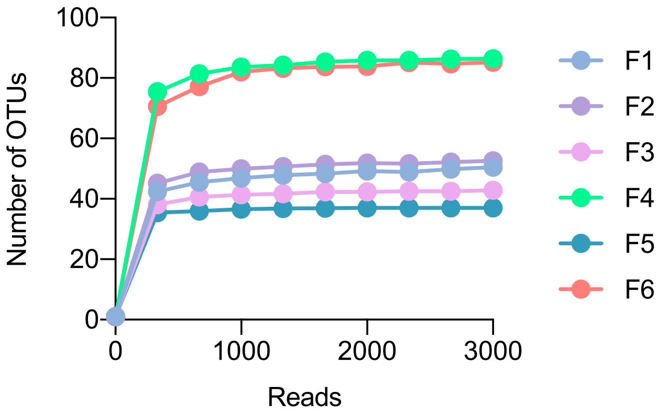

Supplementary Fig. S6 Rarefaction curves of observed OTUs in V3-V4 16S rRNA gene amplicon sequencing of human fecal samples using the MiSeq™ platform.

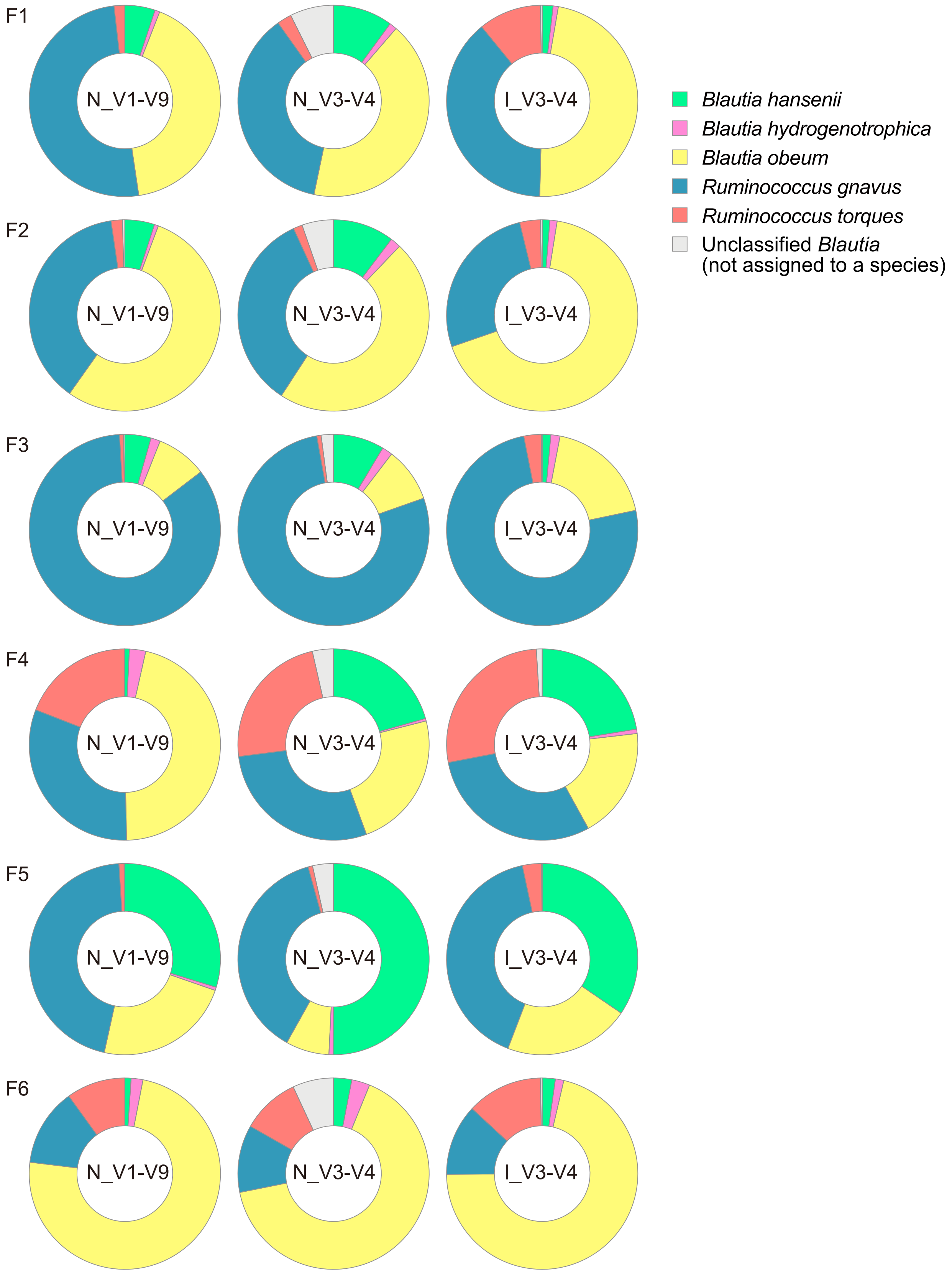

Supplementary Fig. S7 Species composition of *Blautia* in human fecal samples. Results of the 16S rRNA gene analysis obtained by the three sequencing methods are shown. N_V1-V9: sequencing of the V1-V9 region using Oxford Nanopore MinION™. N_V3-V4: sequencing of the V3-V4 region using Oxford Nanopore MinION™. I_V3-V4: sequencing of the V3-V4 region using Illumina MiSeq™.

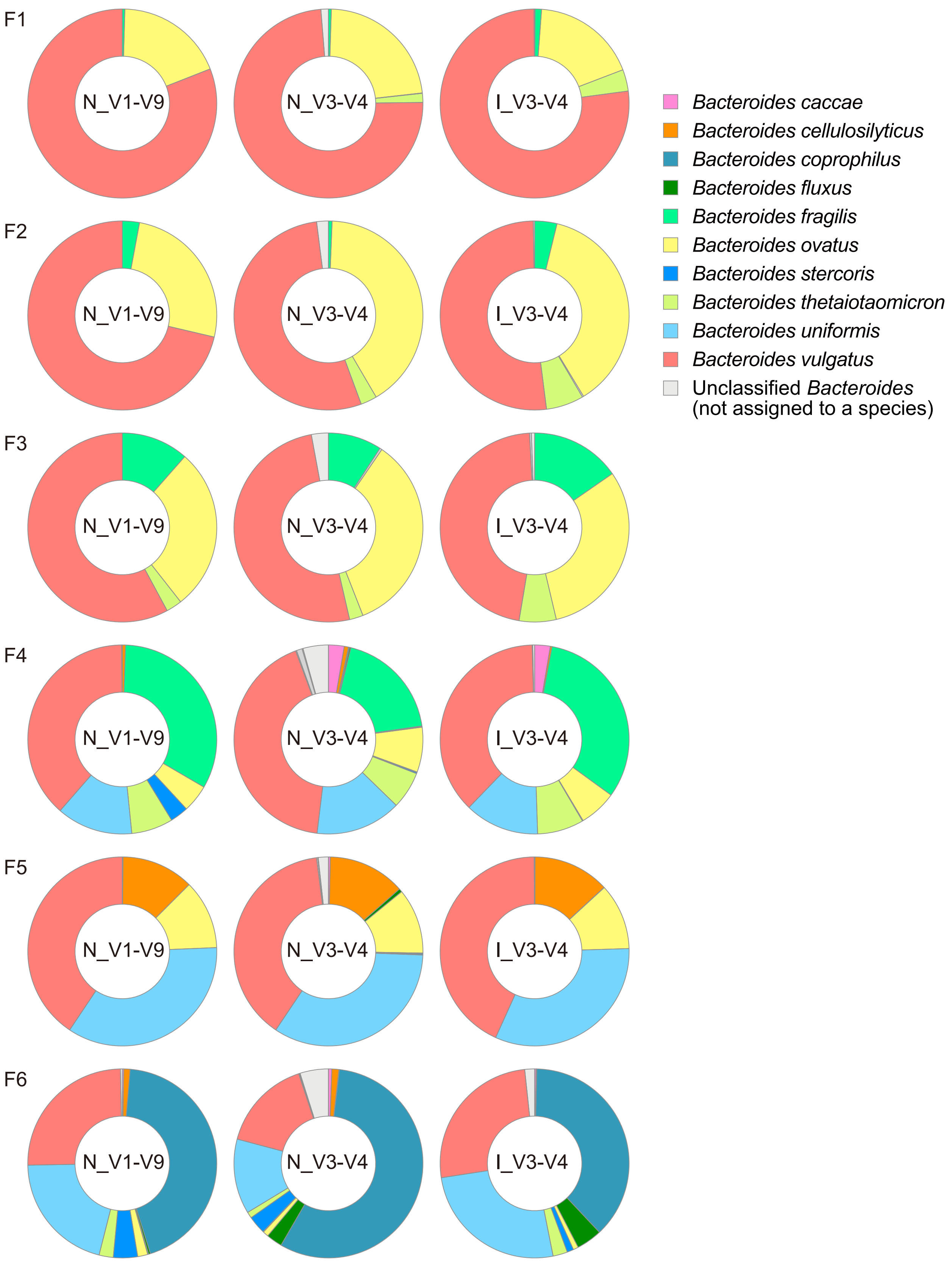

Supplementary Fig. S8 Species composition of *Bacteroides* in human fecal samples. Results of the 16S rRNA gene analysis obtained by the three sequencing methods are shown. N_V1-V9: sequencing of the V1-V9 region using Oxford Nanopore MinION™. N_V3-V4: sequencing of the V3-V4 region using Oxford Nanopore MinION™. I_V3-V4: sequencing of the V3-V4 region using Illumina MiSeq™. The legends show the ten most abundant species.

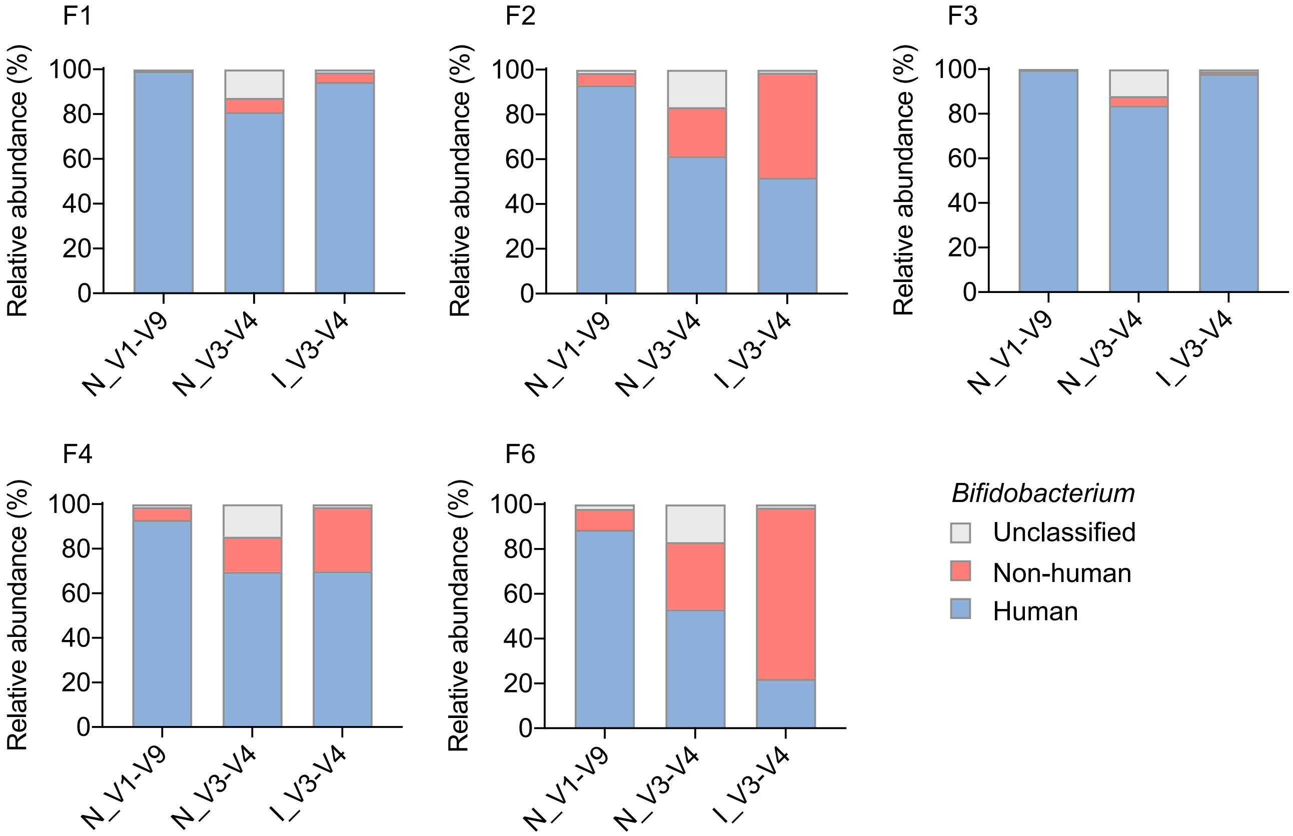

Supplementary Fig. S9 Deviations in the relative abundances of *Bifidobacterium* species in the 16S rRNA gene analysis of human fecal samples. The proportions (%) of *Bifidobacterium* species originating from human or non-human hosts are shown. N_V1-V9: sequencing of the V1-V9 region using Oxford Nanopore MinION™. N_V3-V4: sequencing of the V3-V4 region using Oxford Nanopore MinION™. I_V3-V4: sequencing of the V3-V4 region using Illumina MiSeq™.

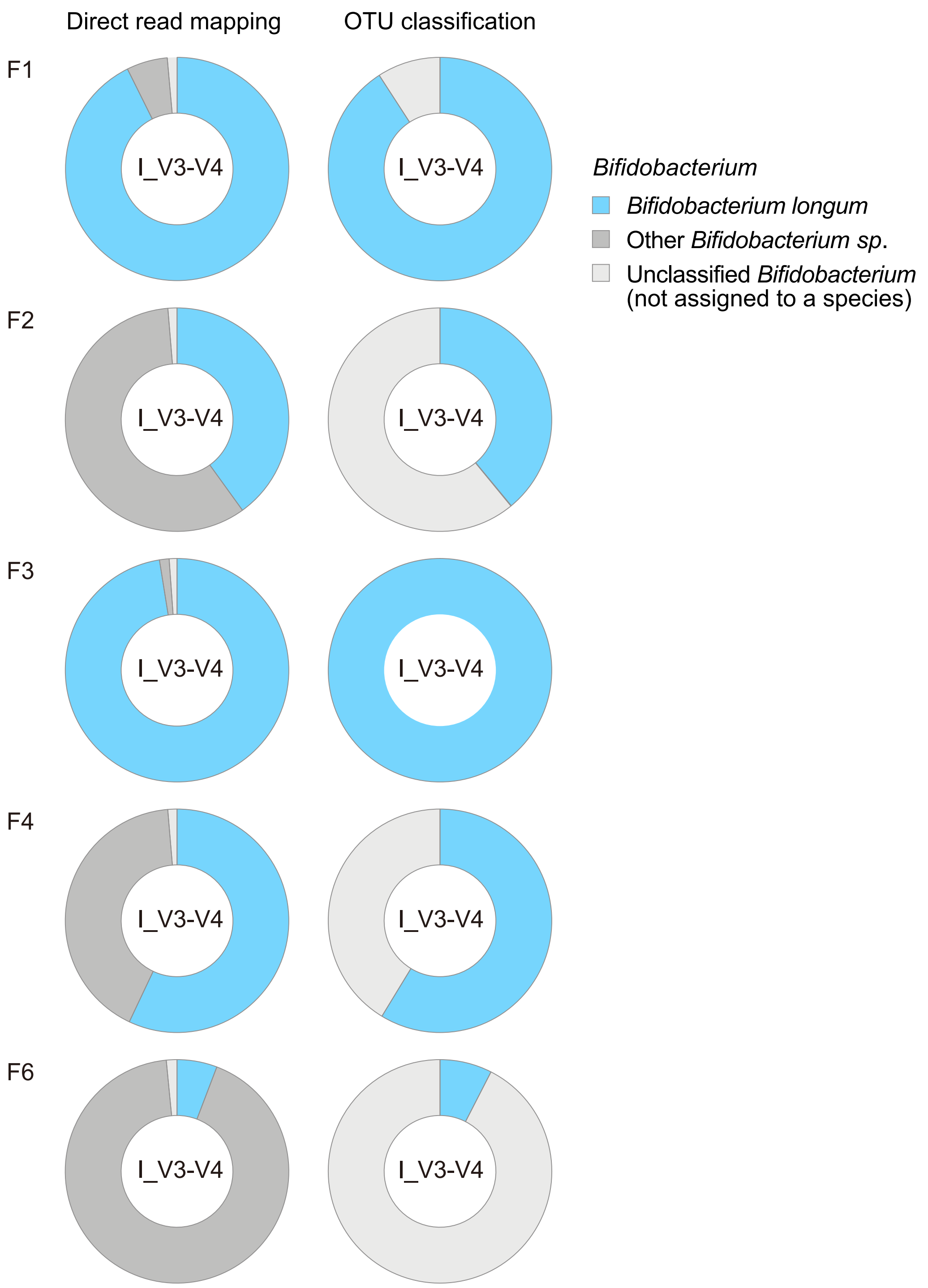

Supplementary Fig. S10 Comparison of species composition of fecal *Bifidobacterium* between classification methods. MiSeq™ V3-V4 reads (I_V3-V4) from the human fecal samples (F1-F6) were either mapped directly to the reference bacterial genome (Direct read mapping) or clustered into OTUs followed by taxonomic annotation using the QIIME 2 pipeline (OTU classification). Taxonomic profiles for reads assigned to *Bifidobacterium* are shown. The OTU-based method identified *Bifidobacterium longum,* and other reads were not assigned to species level, categorized as "Unclassified *Bifidobacterium*" in the charts (right). For clarity, reads assigned to *Bifidobacterium* species other than *Bifidobacterium longum* in our direct mapping approach were shown as "Other *Bifidobacterium*" (left).
