# Supplementary material for: Full-length 16S rRNA gene amplicon analysis of human gut microbiota using MinION™ nanopore sequencing confers species-level resolution
Source: BMC Microbiol. 2021 Jan 26;21:35. doi: 10.1186/s12866-021-02094-5 (PMC7836573; doi:10.1186/s12866-021-02094-5)
Supplement: Supplementary file 6 — Additional file 6. Alignment search results for V1-V9 amplicon sequencing of the mock community. [file 12866_2021_2094_MOESM6_ESM.pdf]

Alignment search results for V1–V9 amplicon sequencing of the mock community.
The top three hits for 3,000 query sequences are shown. The values represent the bit score of alignment.

1
735 Escherichia coli str. K-12 substr. MG1655 GCA_000005845.2
733 Escherichia coli O104_3AH4 str. 2011C-3493 GCA_000299455.1
733 Shigella flexneri 2a str. 301 GCA_000006925.2
720 Escherichia coli O157_3AH7 str. Sakai GCA_000008865.1
2
399 Deinococcus radiodurans R1 GCA_000008565.1
240 Deinococcus hopiensis KR-140 GCA_900176165.1
239 Deinococcus deserti VCD115 GCA_000020685.1
3
823 Clostridium beijerinckii GCA_000833105.2
786 Clostridium saccharoperbutylacetonicum N1-4_28HMT_29 GCA_000340885.1
757 Clostridium puniceum GCA_002006345.1
4
716 Clostridium beijerinckii GCA_000833105.2
656 Clostridium saccharobutylicum DSM 13864 GCA_000473995.1
653 Clostridium saccharoperbutylacetonicum N1-4_28HMT_29 GCA_000340885.1
5
474 Staphylococcus epidermidis ATCC 12228 GCA_000007645.1
448 Staphylococcus capitis subsp. capitis GCA_001028645.1
386 Megasphaera cerevisiae DSM 20462 GCA_001045675.1
386 Staphylococcus warneri SG1 GCA_000332735.1
6
544 Staphylococcus epidermidis ATCC 12228 GCA_000007645.1
513 Staphylococcus capitis subsp. capitis GCA_001028645.1
448 Staphylococcus hominis subsp. hominis C80 GCA_000183685.1
7
773 Clostridium beijerinckii GCA_000833105.2
762 Clostridium saccharoperbutylacetonicum N1-4_28HMT_29 GCA_000340885.1
721 Clostridium puniceum GCA_002006345.1
8
680 Lactobacillus gasseri ATCC 33323 = JCM 1131 GCA_000014425.1
609 Lactobacillus hominis DSM 23910 = CRBIP 24.179 GCA_000296835.1
446 Lactobacillus iners DSM 13335 GCA_000160875.1
9
765 Lactobacillus gasseri ATCC 33323 = JCM 1131 GCA_000014425.1
701 Lactobacillus hominis DSM 23910 = CRBIP 24.179 GCA_000296835.1
499 Lactobacillus psittaci DSM 15354 GCA_000425905.1
a
675 Clostridium beijerinckii GCA_000833105.2
635 Clostridium saccharoperbutylacetonicum N1-4_28HMT_29 GCA_000340885.1
594 Clostridium puniceum GCA_002006345.1
b
462 Clostridium beijerinckii GCA_000833105.2
440 Clostridium saccharobutylicum DSM 13864 GCA_000473995.1
438 Clostridium butyricum GCA_001456065.2
c
676 Lactobacillus gasseri ATCC 33323 = JCM 1131 GCA_000014425.1
645 Lactobacillus hominis DSM 23910 = CRBIP 24.179 GCA_000296835.1
480 Lactobacillus iners DSM 13335 GCA_000160875.1
d
753 Clostridium beijerinckii GCA_000833105.2
724 Clostridium saccharoperbutylacetonicum N1-4_28HMT_29 GCA_000340885.1
697 Clostridium puniceum GCA_002006345.1
e
742 Streptococcus mutans UA159 GCA_000007465.2
546 Streptococcus ratti FA-1 = DSM 20564 GCA_000286075.1
490 Streptococcus halotolerans GCA_001598035.1
f
384 Enterococcus faecalis V583 GCA_000007785.1
384 Streptomyces cinnamoneus GCA_001885705.1
351 Enterococcus faecium DO GCA_000174395.2
338 Enterococcus casseliflavus EC20 GCA_000157355.2
338 Enterococcus dispar ATCC 51266 GCA_000406945.1
338 Enterococcus hirae ATCC 9790 GCA_000271405.2
338 Enterococcus massiliensis GCA_001050095.1
338 Enterococcus mundtii QU 25 GCA_000504125.1
338 Enterococcus saccharolyticus subsp. saccharolyticus ATCC 43076 GCA_000407285.1
g
824 Deinococcus radiodurans R1 GCA_000008565.1
529 Deinococcus deserti VCD115 GCA_000020685.1
486 Deinococcus hopiensis KR-140 GCA_900176165.1

h
787 Staphylococcus epidermidis ATCC 12228 GCA_000007645.1
776 Staphylococcus capitis subsp. capitis GCA_001028645.1
704 Staphylococcus hominis subsp. hominis C80 GCA_000183685.1
i
668 Staphylococcus epidermidis ATCC 12228 GCA_000007645.1
627 Staphylococcus capitis subsp. capitis GCA_001028645.1
573 Staphylococcus hominis subsp. hominis C80 GCA_000183685.1
j
736 Enterococcus faecalis V583 GCA_000007785.1
689 Streptomyces cinnamoneus GCA_001885705.1
564 Enterococcus rivorum GCA_001742285.1
k
672 Rhodobacter sphaeroides 2.4.1 GCA_000012905.2
624 Rhodobacter sphaeroides ATCC 17025 GCA_000016405.1
520 Gemmobacter aquatilis GCA_900110025.1
l
753 Rhodobacter sphaeroides 2.4.1 GCA_000012905.2
688 Rhodobacter sphaeroides ATCC 17025 GCA_000016405.1
561 Gemmobacter aquatilis GCA_900110025.1
m
717 Bacillus anthracis str. Ames GCA_000007845.1
717 Bacillus anthracis str. Sterne GCA_000008165.1
717 Bacillus cereus ATCC 14579 GCA_000007825.1
717 Bacillus thuringiensis YBT-1518 GCA_000497525.2
717 _5BBacillus thuringiensis_5D serovar konkukian str. 97-27 GCA_000008505.1
704 Bacillus pseudomycoides DSM 12442 GCA_000161455.1
697 Bacillus mycoides GCA_000832605.1
n
536 Lactobacillus gasseri ATCC 33323 = JCM 1131 GCA_000014425.1
522 Lactobacillus hominis DSM 23910 = CRBIP 24.179 GCA_000296835.1
382 Lactobacillus iners DSM 13335 GCA_000160875.1
o
522 Escherichia coli str. K-12 substr. MG1655 GCA_000005845.2
513 Escherichia coli O104_3AH4 str. 2011C-3493 GCA_000299455.1
513 Shigella flexneri 2a str. 301 GCA_000006925.2
506 Escherichia coli O157_3AH7 str. Sakai GCA_000008865.1
p
833 Clostridium beijerinckii GCA_000833105.2
774 Clostridium saccharoperbutylacetonicum N1-4_28HMT_29 GCA_000340885.1
770 Clostridium puniceum GCA_002006345.1
q
514 Staphylococcus epidermidis ATCC 12228 GCA_000007645.1
505 Staphylococcus capitis subsp. capitis GCA_001028645.1
479 Staphylococcus condimenti GCA_001618885.1
479 Staphylococcus simulans GCA_001559115.1
r
510 Escherichia coli O157_3AH7 str. Sakai GCA_000008865.1
510 Escherichia coli str. K-12 substr. MG1655 GCA_000005845.2
501 Escherichia coli IAI39 GCA_000026345.1
495 Escherichia coli UMN026 GCA_000026325.2
s
476 Staphylococcus epidermidis ATCC 12228 GCA_000007645.1
440 Staphylococcus capitis subsp. capitis GCA_001028645.1
383 Staphylococcus lugdunensis HKU09-01 GCA_000025085.1
t
640 Staphylococcus epidermidis ATCC 12228 GCA_000007645.1
618 Staphylococcus capitis subsp. capitis GCA_001028645.1
582 Staphylococcus aureus subsp. aureus NCTC 8325 GCA_000013425.1
u
593 Rhodobacter sphaeroides 2.4.1 GCA_000012905.2
524 Rhodobacter sphaeroides ATCC 17025 GCA_000016405.1
443 Gemmobacter aquatilis GCA_900110025.1
v
788 Deinococcus radiodurans R1 GCA_000008565.1
518 Deinococcus deserti VCD115 GCA_000020685.1
489 Deinococcus gobiensis I-0 GCA_000252445.1
w
1035 Enterococcus faecalis V583 GCA_000007785.1
989 Streptomyces cinnamoneus GCA_001885705.1
929 Enterococcus rivorum GCA_001742285.1
x
519 Enterococcus faecalis V583 GCA_000007785.1
473 Enterococcus rivorum GCA_001742285.1
472 Streptomyces cinnamoneus GCA_001885705.1
y

518 Staphylococcus epidermidis ATCC 12228 GCA_000007645.1
478 Staphylococcus capitis subsp. capitis GCA_001028645.1
450 Staphylococcus warneri SG1 GCA_000332735.1
z
492 Escherichia coli str. K-12 substr. MG1655 GCA_000005845.2
482 Shigella flexneri 2a str. 301 GCA_000006925.2
478 Escherichia coli UMN026 GCA_000026325.2
A
876 Staphylococcus epidermidis ATCC 12228 GCA_000007645.1
868 Staphylococcus capitis subsp. capitis GCA_001028645.1
800 Staphylococcus haemolyticus JCSC1435 GCA_000009865.1
800 Staphylococcus hominis subsp. hominis C80 GCA_000183685.1
B
639 Escherichia coli O157_3AH7 str. Sakai GCA_000008865.1
635 Escherichia coli UMN026 GCA_000026325.2
635 Escherichia coli str. K-12 substr. MG1655 GCA_000005845.2
631 Escherichia coli IAI39 GCA_000026345.1
631 Escherichia coli O83_3AH1 str. NRG 857C GCA_000183345.1
631 Shigella flexneri 2a str. 301 GCA_000006925.2
C
660 Deinococcus radiodurans R1 GCA_000008565.1
452 Deinococcus gobiensis I-0 GCA_000252445.1
434 Deinococcus deserti VCD115 GCA_000020685.1
D
571 Escherichia coli str. K-12 substr. MG1655 GCA_000005845.2
558 Escherichia coli O157_3AH7 str. Sakai GCA_000008865.1
558 Escherichia coli O83_3AH1 str. NRG 857C GCA_000183345.1
558 Escherichia coli UMN026 GCA_000026325.2
558 Shigella flexneri 2a str. 301 GCA_000006925.2
554 Escherichia coli O104_3AH4 str. 2011C-3493 GCA_000299455.1
E
709 Rhodobacter sphaeroides 2.4.1 GCA_000012905.2
615 Rhodobacter sphaeroides ATCC 17025 GCA_000016405.1
544 Gemmobacter aquatilis GCA_900110025.1
F
943 Streptococcus mutans UA159 GCA_000007465.2
662 Streptococcus ratti FA-1 = DSM 20564 GCA_000286075.1
592 Streptococcus macacae NCTC 11558 GCA_000187995.3
G
549 Clostridium saccharoperbutylacetonicum N1-4_28HMT_29 GCA_000340885.1
543 Clostridium beijerinckii GCA_000833105.2
513 Clostridium puniceum GCA_002006345.1
H
806 Streptococcus mutans UA159 GCA_000007465.2
581 Streptococcus ratti FA-1 = DSM 20564 GCA_000286075.1
560 Streptococcus gallolyticus subsp. gallolyticus DSM 16831 GCA_002000985.1
I
711 Enterococcus faecalis V583 GCA_000007785.1
665 Streptomyces cinnamoneus GCA_001885705.1
621 Enterococcus rivorum GCA_001742285.1
J
796 Clostridium beijerinckii GCA_000833105.2
776 Clostridium saccharoperbutylacetonicum N1-4_28HMT_29 GCA_000340885.1
762 Clostridium saccharobutylicum DSM 13864 GCA_000473995.1
K
818 Streptococcus mutans UA159 GCA_000007465.2
653 Streptococcus ratti FA-1 = DSM 20564 GCA_000286075.1
640 Streptococcus merionis DSM 19192 GCA_000380085.1
L
586 Clostridium beijerinckii GCA_000833105.2
563 Clostridium saccharoperbutylacetonicum N1-4_28HMT_29 GCA_000340885.1
538 Clostridium puniceum GCA_002006345.1
M
707 Deinococcus radiodurans R1 GCA_000008565.1
470 Deinococcus deserti VCD115 GCA_000020685.1
426 Deinococcus soli Cha et al. 2016 GCA_001007995.1
N
738 Bifidobacterium adolescentis ATCC 15703 GCA_000010425.1
697 Bifidobacterium dentium JCM 1195 = DSM 20436 GCA_001042595.1
660 Bifidobacterium angulatum DSM 20098 = JCM 7096 GCA_001025155.1
O
462 Staphylococcus epidermidis ATCC 12228 GCA_000007645.1
456 Staphylococcus lugdunensis HKU09-01 GCA_000025085.1
449 Staphylococcus lutrae GCA_002101335.1
P
830 Enterococcus faecalis V583 GCA_000007785.1

783 Streptomyces cinnamoneus GCA_001885705.1
778 Enterococcus haemoperoxidus ATCC BAA-382 GCA_000407165.1
Q
742 Clostridium beijerinckii GCA_000833105.2
696 Clostridium saccharoperbutylacetonicum N1-4_28HMT_29 GCA_000340885.1
679 Clostridium puniceum GCA_002006345.1
R
789 Clostridium beijerinckii GCA_000833105.2
788 Clostridium saccharoperbutylacetonicum N1-4_28HMT_29 GCA_000340885.1
772 Clostridium puniceum GCA_002006345.1
S
619 Clostridium beijerinckii GCA_000833105.2
593 Clostridium saccharoperbutylacetonicum N1-4_28HMT_29 GCA_000340885.1
592 Clostridium puniceum GCA_002006345.1
T
533 Escherichia coli O157_3AH7 str. Sakai GCA_000008865.1
519 Escherichia coli str. K-12 substr. MG1655 GCA_000005845.2
519 Shigella flexneri 2a str. 301 GCA_000006925.2
518 Escherichia coli UMN026 GCA_000026325.2
U
738 Clostridium beijerinckii GCA_000833105.2
705 Clostridium puniceum GCA_002006345.1
705 Clostridium saccharoperbutylacetonicum N1-4_28HMT_29 GCA_000340885.1
693 Clostridium saccharobutylicum DSM 13864 GCA_000473995.1
V
606 Bifidobacterium adolescentis ATCC 15703 GCA_000010425.1
520 Bifidobacterium dentium JCM 1195 = DSM 20436 GCA_001042595.1
476 Bifidobacterium angulatum DSM 20098 = JCM 7096 GCA_001025155.1
W
685 Deinococcus radiodurans R1 GCA_000008565.1
472 Deinococcus deserti VCD115 GCA_000020685.1
430 Deinococcus puniceus GCA_001644565.1
X
901 Lactobacillus gasseri ATCC 33323 = JCM 1131 GCA_000014425.1
834 Lactobacillus hominis DSM 23910 = CRBIP 24.179 GCA_000296835.1
592 Lactobacillus iners DSM 13335 GCA_000160875.1
Y
658 Bifidobacterium adolescentis ATCC 15703 GCA_000010425.1
501 Bifidobacterium angulatum DSM 20098 = JCM 7096 GCA_001025155.1
476 Bifidobacterium dentium JCM 1195 = DSM 20436 GCA_001042595.1
Z
819 Lactobacillus gasseri ATCC 33323 = JCM 1131 GCA_000014425.1
764 Lactobacillus hominis DSM 23910 = CRBIP 24.179 GCA_000296835.1
576 Lactobacillus iners DSM 13335 GCA_000160875.1
10
803 Lactobacillus gasseri ATCC 33323 = JCM 1131 GCA_000014425.1
709 Lactobacillus hominis DSM 23910 = CRBIP 24.179 GCA_000296835.1
594 Lactobacillus iners DSM 13335 GCA_000160875.1
11
523 Deinococcus radiodurans R1 GCA_000008565.1
375 Deinococcus deserti VCD115 GCA_000020685.1
370 Deinococcus hopiensis KR-140 GCA_900176165.1
12
652 Deinococcus radiodurans R1 GCA_000008565.1
384 Deinococcus deserti VCD115 GCA_000020685.1
340 Deinococcus proteolyticus MRP GCA_000190555.1
13
558 Staphylococcus epidermidis ATCC 12228 GCA_000007645.1
527 Staphylococcus haemolyticus JCSC1435 GCA_000009865.1
518 Staphylococcus capitis subsp. capitis GCA_001028645.1
14
880 Clostridium beijerinckii GCA_000833105.2
822 Clostridium saccharoperbutylacetonicum N1-4_28HMT_29 GCA_000340885.1
797 Clostridium butyricum GCA_001456065.2
15
786 Clostridium beijerinckii GCA_000833105.2
753 Clostridium saccharoperbutylacetonicum N1-4_28HMT_29 GCA_000340885.1
730 Clostridium puniceum GCA_002006345.1
16
704 Escherichia coli O157_3AH7 str. Sakai GCA_000008865.1
704 Escherichia coli str. K-12 substr. MG1655 GCA_000005845.2
680 Shigella flexneri 2a str. 301 GCA_000006925.2
674 Escherichia coli UMN026 GCA_000026325.2
17
499 Staphylococcus epidermidis ATCC 12228 GCA_000007645.1
481 Staphylococcus capitis subsp. capitis GCA_001028645.1

422 Staphylococcus warneri SG1 GCA_000332735.1
18
516 Clostridium beijerinckii GCA_000833105.2
516 Clostridium saccharobutylicum DSM 13864 GCA_000473995.1
501 Clostridium saccharoperbutylacetonicum N1-4_28HMT_29 GCA_000340885.1
482 Clostridium puniceum GCA_002006345.1
19
636 Rhodobacter sphaeroides 2.4.1 GCA_000012905.2
563 Rhodobacter sphaeroides ATCC 17025 GCA_000016405.1
488 Pseudorhodobacter ferrugineus DSM 5888 GCA_000420745.1
1a
712 Bifidobacterium adolescentis ATCC 15703 GCA_000010425.1
550 Bifidobacterium callitrichos DSM 23973 GCA_000741175.1
539 Bifidobacterium angulatum DSM 20098 = JCM 7096 GCA_001025155.1
1b
781 Clostridium beijerinckii GCA_000833105.2
777 Clostridium puniceum GCA_002006345.1
777 Clostridium saccharoperbutylacetonicum N1-4_28HMT_29 GCA_000340885.1
747 Clostridium saccharobutylicum DSM 13864 GCA_000473995.1
1c
586 Clostridium beijerinckii GCA_000833105.2
547 Clostridium saccharoperbutylacetonicum N1-4_28HMT_29 GCA_000340885.1
540 Clostridium saccharobutylicum DSM 13864 GCA_000473995.1
1d
525 Streptococcus mutans UA159 GCA_000007465.2
357 Streptococcus ratti FA-1 = DSM 20564 GCA_000286075.1
353 Streptococcus sobrinus DSM 20742 = ATCC 33478 GCA_000686605.1
1e
811 Clostridium beijerinckii GCA_000833105.2
778 Clostridium saccharoperbutylacetonicum N1-4_28HMT_29 GCA_000340885.1
738 Clostridium saccharobutylicum DSM 13864 GCA_000473995.1
1f
470 Clostridium beijerinckii GCA_000833105.2
441 Clostridium saccharoperbutylacetonicum N1-4_28HMT_29 GCA_000340885.1
421 Clostridium saccharobutylicum DSM 13864 GCA_000473995.1
1g
963 Staphylococcus epidermidis ATCC 12228 GCA_000007645.1
916 Staphylococcus capitis subsp. capitis GCA_001028645.1
853 Staphylococcus warneri SG1 GCA_000332735.1
1h
785 Rhodobacter sphaeroides 2.4.1 GCA_000012905.2
753 Rhodobacter sphaeroides ATCC 17025 GCA_000016405.1
675 Gemmobacter aquatilis GCA_900110025.1
1i
688 Lactobacillus gasseri ATCC 33323 = JCM 1131 GCA_000014425.1
629 Lactobacillus hominis DSM 23910 = CRBIP 24.179 GCA_000296835.1
459 Lactobacillus psittaci DSM 15354 GCA_000425905.1
1j
742 Staphylococcus epidermidis ATCC 12228 GCA_000007645.1
732 Staphylococcus capitis subsp. capitis GCA_001028645.1
679 Megasphaera cerevisiae DSM 20462 GCA_001045675.1
679 Staphylococcus warneri SG1 GCA_000332735.1
1k
608 Bacillus anthracis str. Ames GCA_000007845.1
608 Bacillus anthracis str. Sterne GCA_000008165.1
608 _5BBacillus thuringiensis_5D serovar konkukian str. 97-27 GCA_000008505.1
607 Bacillus cereus ATCC 14579 GCA_000007825.1
602 Bacillus thuringiensis YBT-1518 GCA_000497525.2
1l
621 Clostridium beijerinckii GCA_000833105.2
577 Clostridium saccharoperbutylacetonicum N1-4_28HMT_29 GCA_000340885.1
550 Clostridium puniceum GCA_002006345.1
550 Clostridium saccharobutylicum DSM 13864 GCA_000473995.1
1m
705 Deinococcus radiodurans R1 GCA_000008565.1
399 Deinococcus deserti VCD115 GCA_000020685.1
376 Deinococcus gobiensis I-0 GCA_000252445.1
1n
526 Lactobacillus gasseri ATCC 33323 = JCM 1131 GCA_000014425.1
461 Lactobacillus hominis DSM 23910 = CRBIP 24.179 GCA_000296835.1
399 Lactobacillus iners DSM 13335 GCA_000160875.1
1o
863 Deinococcus radiodurans R1 GCA_000008565.1
561 Deinococcus deserti VCD115 GCA_000020685.1
550 Deinococcus hopiensis KR-140 GCA_900176165.1
1p

696 Staphylococcus epidermidis ATCC 12228 GCA_000007645.1
692 Staphylococcus capitis subsp. capitis GCA_001028645.1
628 Staphylococcus warneri SG1 GCA_000332735.1
1q
473 Lactobacillus gasseri ATCC 33323 = JCM 1131 GCA_000014425.1
420 Lactobacillus hominis DSM 23910 = CRBIP 24.179 GCA_000296835.1
332 Lactobacillus iners DSM 13335 GCA_000160875.1
1r
776 Deinococcus radiodurans R1 GCA_000008565.1
461 Deinococcus marmoris DSM 12784 GCA_000701405.1
457 Deinococcus puniceus GCA_001644565.1
1s
806 Rhodobacter sphaeroides 2.4.1 GCA_000012905.2
768 Rhodobacter sphaeroides ATCC 17025 GCA_000016405.1
608 Gemmobacter aquatilis GCA_900110025.1
1t
778 Staphylococcus epidermidis ATCC 12228 GCA_000007645.1
728 Staphylococcus capitis subsp. capitis GCA_001028645.1
670 Staphylococcus aureus subsp. aureus NCTC 8325 GCA_000013425.1
1u
620 Enterococcus faecalis V583 GCA_000007785.1
606 Enterococcus massiliensis GCA_001050095.1
596 Enterococcus mundtii QU 25 GCA_000504125.1
1v
708 Staphylococcus epidermidis ATCC 12228 GCA_000007645.1
677 Staphylococcus capitis subsp. capitis GCA_001028645.1
645 Staphylococcus lugdunensis HKU09-01 GCA_000025085.1
1w
768 Deinococcus radiodurans R1 GCA_000008565.1
539 Deinococcus marmoris DSM 12784 GCA_000701405.1
539 Deinococcus swuensis GCA_000800395.1
536 Deinococcus deserti VCD115 GCA_000020685.1
1x
742 Rhodobacter sphaeroides 2.4.1 GCA_000012905.2
691 Rhodobacter sphaeroides ATCC 17025 GCA_000016405.1
544 Gemmobacter aquatilis GCA_900110025.1
1y
520 Clostridium beijerinckii GCA_000833105.2
493 Clostridium saccharobutylicum DSM 13864 GCA_000473995.1
493 Clostridium saccharoperbutylacetonicum N1-4_28HMT_29 GCA_000340885.1
487 Clostridium chromiireducens GCA_002029255.1
1z
900 Streptococcus mutans UA159 GCA_000007465.2
679 Streptococcus ratti FA-1 = DSM 20564 GCA_000286075.1
645 Streptococcus gordonii str. Challis substr. CH1 GCA_000017005.1
1A
769 Clostridium beijerinckii GCA_000833105.2
735 Clostridium saccharoperbutylacetonicum N1-4_28HMT_29 GCA_000340885.1
688 Clostridium puniceum GCA_002006345.1
1B
695 Rhodobacter sphaeroides 2.4.1 GCA_000012905.2
596 Rhodobacter sphaeroides ATCC 17025 GCA_000016405.1
555 Gemmobacter aquatilis GCA_900110025.1
1C
526 Rhodobacter sphaeroides 2.4.1 GCA_000012905.2
455 Rhodobacter sphaeroides ATCC 17025 GCA_000016405.1
333 Pseudorhodobacter psychrotolerans GCA_001294535.1
1D
625 Lactobacillus gasseri ATCC 33323 = JCM 1131 GCA_000014425.1
575 Lactobacillus hominis DSM 23910 = CRBIP 24.179 GCA_000296835.1
475 Lactobacillus iners DSM 13335 GCA_000160875.1
1E
495 Lactobacillus gasseri ATCC 33323 = JCM 1131 GCA_000014425.1
457 Lactobacillus hominis DSM 23910 = CRBIP 24.179 GCA_000296835.1
337 Lactobacillus iners DSM 13335 GCA_000160875.1
1F
784 Escherichia coli str. K-12 substr. MG1655 GCA_000005845.2
761 Escherichia coli O104_3AH4 str. 2011C-3493 GCA_000299455.1
752 Shigella flexneri 2a str. 301 GCA_000006925.2
1G
542 Clostridium saccharoperbutylacetonicum N1-4_28HMT_29 GCA_000340885.1
540 Clostridium beijerinckii GCA_000833105.2
513 Clostridium butyricum GCA_001456065.2
1H
648 Rhodobacter sphaeroides 2.4.1 GCA_000012905.2
608 Rhodobacter sphaeroides ATCC 17025 GCA_000016405.1

502 Gemmobacter aquatilis GCA_900110025.1
1I
745 Clostridium beijerinckii GCA_000833105.2
692 Clostridium saccharobutylicum DSM 13864 GCA_000473995.1
692 Clostridium saccharoperbutylacetonicum N1-4_28HMT_29 GCA_000340885.1
677 Clostridium puniceum GCA_002006345.1
1J
824 Lactobacillus gasseri ATCC 33323 = JCM 1131 GCA_000014425.1
771 Lactobacillus hominis DSM 23910 = CRBIP 24.179 GCA_000296835.1
517 Lactobacillus iners DSM 13335 GCA_000160875.1
1K
818 Staphylococcus epidermidis ATCC 12228 GCA_000007645.1
773 Staphylococcus capitis subsp. capitis GCA_001028645.1
702 Staphylococcus warneri SG1 GCA_000332735.1
1L
777 Staphylococcus epidermidis ATCC 12228 GCA_000007645.1
739 Staphylococcus capitis subsp. capitis GCA_001028645.1
661 Staphylococcus haemolyticus JCSC1435 GCA_000009865.1
661 Staphylococcus hominis subsp. hominis C80 GCA_000183685.1
1M
380 Lactobacillus gasseri ATCC 33323 = JCM 1131 GCA_000014425.1
380 Lactobacillus hominis DSM 23910 = CRBIP 24.179 GCA_000296835.1
358 Lactobacillus kimchicus JCM 15530 GCA_001433995.1
356 Enterococcus faecalis V583 GCA_000007785.1
1N
414 Deinococcus radiodurans R1 GCA_000008565.1
254 Deinococcus deserti VCD115 GCA_000020685.1
244 Deinococcus hopiensis KR-140 GCA_900176165.1
1O
592 Bifidobacterium adolescentis ATCC 15703 GCA_000010425.1
457 Bifidobacterium dentium JCM 1195 = DSM 20436 GCA_001042595.1
450 Bifidobacterium angulatum DSM 20098 = JCM 7096 GCA_001025155.1
450 Bifidobacterium callitrichos DSM 23973 GCA_000741175.1
1P
645 Enterococcus faecalis V583 GCA_000007785.1
598 Streptomyces cinnamoneus GCA_001885705.1
577 Enterococcus rivorum GCA_001742285.1
1Q
439 Enterococcus faecalis V583 GCA_000007785.1
399 Enterococcus haemoperoxidus ATCC BAA-382 GCA_000407165.1
393 Enterococcus rivorum GCA_001742285.1
1R
490 Clostridium beijerinckii GCA_000833105.2
487 Clostridium saccharoperbutylacetonicum N1-4_28HMT_29 GCA_000340885.1
482 Clostridium saccharobutylicum DSM 13864 GCA_000473995.1
1S
780 Escherichia coli O104_3AH4 str. 2011C-3493 GCA_000299455.1
780 Escherichia coli str. K-12 substr. MG1655 GCA_000005845.2
773 Shigella flexneri 2a str. 301 GCA_000006925.2
758 Escherichia coli O157_3AH7 str. Sakai GCA_000008865.1
758 Escherichia coli UMN026 GCA_000026325.2
1T
417 Clostridium beijerinckii GCA_000833105.2
379 Clostridium saccharoperbutylacetonicum N1-4_28HMT_29 GCA_000340885.1
369 Clostridium saccharobutylicum DSM 13864 GCA_000473995.1
1U
780 Streptococcus mutans UA159 GCA_000007465.2
586 Streptococcus ratti FA-1 = DSM 20564 GCA_000286075.1
537 Streptococcus gordonii str. Challis substr. CH1 GCA_000017005.1
1V
720 Staphylococcus epidermidis ATCC 12228 GCA_000007645.1
659 Staphylococcus capitis subsp. capitis GCA_001028645.1
635 Staphylococcus aureus subsp. aureus NCTC 8325 GCA_000013425.1
1W
485 Bifidobacterium adolescentis ATCC 15703 GCA_000010425.1
451 Bifidobacterium dentium JCM 1195 = DSM 20436 GCA_001042595.1
440 Bifidobacterium angulatum DSM 20098 = JCM 7096 GCA_001025155.1
1X
723 Enterococcus faecalis V583 GCA_000007785.1
676 Streptomyces cinnamoneus GCA_001885705.1
629 Enterococcus saccharolyticus subsp. saccharolyticus ATCC 43076 GCA_000407285.1
1Y
793 Staphylococcus epidermidis ATCC 12228 GCA_000007645.1
755 Staphylococcus capitis subsp. capitis GCA_001028645.1
708 Staphylococcus haemolyticus JCSC1435 GCA_000009865.1
1Z

736 Staphylococcus epidermidis ATCC 12228 GCA_000007645.1
673 Staphylococcus capitis subsp. capitis GCA_001028645.1
637 Staphylococcus hominis subsp. hominis C80 GCA_000183685.1
20
634 Deinococcus radiodurans R1 GCA_000008565.1
393 Deinococcus deserti VCD115 GCA_000020685.1
341 Deinococcus hopiensis KR-140 GCA_900176165.1
21
445 Lactobacillus gasseri ATCC 33323 = JCM 1131 GCA_000014425.1
432 Lactobacillus hominis DSM 23910 = CRBIP 24.179 GCA_000296835.1
334 Lactobacillus hamsteri DSM 5661 = JCM 6256 GCA_000615445.1
22
767 Clostridium beijerinckii GCA_000833105.2
743 Clostridium saccharoperbutylacetonicum N1-4_28HMT_29 GCA_000340885.1
706 Clostridium saccharobutylicum DSM 13864 GCA_000473995.1
23
778 Bacillus anthracis str. Ames GCA_000007845.1
778 Bacillus anthracis str. Sterne GCA_000008165.1
778 _5BBacillus thuringiensis_5D serovar konkukian str. 97-27 GCA_000008505.1
763 Bacillus cereus ATCC 14579 GCA_000007825.1
763 Bacillus pseudomycoides DSM 12442 GCA_000161455.1
748 Bacillus thuringiensis YBT-1518 GCA_000497525.2
24
616 Staphylococcus epidermidis ATCC 12228 GCA_000007645.1
605 Staphylococcus capitis subsp. capitis GCA_001028645.1
551 Staphylococcus warneri SG1 GCA_000332735.1
25
710 Staphylococcus epidermidis ATCC 12228 GCA_000007645.1
660 Staphylococcus capitis subsp. capitis GCA_001028645.1
605 Staphylococcus haemolyticus JCSC1435 GCA_000009865.1
26
502 Clostridium beijerinckii GCA_000833105.2
457 Clostridium saccharoperbutylacetonicum N1-4_28HMT_29 GCA_000340885.1
435 Clostridium butyricum GCA_001456065.2
27
996 Streptococcus mutans UA159 GCA_000007465.2
768 Streptococcus ratti FA-1 = DSM 20564 GCA_000286075.1
732 Streptococcus gordonii str. Challis substr. CH1 GCA_000017005.1
28
586 Clostridium beijerinckii GCA_000833105.2
548 Clostridium saccharoperbutylacetonicum N1-4_28HMT_29 GCA_000340885.1
529 Clostridium butyricum GCA_001456065.2
29
865 Lactobacillus gasseri ATCC 33323 = JCM 1131 GCA_000014425.1
794 Lactobacillus hominis DSM 23910 = CRBIP 24.179 GCA_000296835.1
633 Lactobacillus iners DSM 13335 GCA_000160875.1
2a
623 Deinococcus radiodurans R1 GCA_000008565.1
402 Deinococcus gobiensis I-0 GCA_000252445.1
367 Deinococcus pimensis DSM 21231 GCA_000519345.1
2b
676 Staphylococcus epidermidis ATCC 12228 GCA_000007645.1
659 Staphylococcus capitis subsp. capitis GCA_001028645.1
629 Staphylococcus warneri SG1 GCA_000332735.1
2c
777 Clostridium beijerinckii GCA_000833105.2
769 Clostridium saccharoperbutylacetonicum N1-4_28HMT_29 GCA_000340885.1
748 Clostridium saccharobutylicum DSM 13864 GCA_000473995.1
2d
856 Staphylococcus epidermidis ATCC 12228 GCA_000007645.1
829 Staphylococcus capitis subsp. capitis GCA_001028645.1
761 Staphylococcus warneri SG1 GCA_000332735.1
2e
890 Streptococcus mutans UA159 GCA_000007465.2
683 Streptococcus ratti FA-1 = DSM 20564 GCA_000286075.1
642 Streptococcus gordonii str. Challis substr. CH1 GCA_000017005.1
2f
812 Lactobacillus gasseri ATCC 33323 = JCM 1131 GCA_000014425.1
757 Lactobacillus hominis DSM 23910 = CRBIP 24.179 GCA_000296835.1
548 Lactobacillus psittaci DSM 15354 GCA_000425905.1
2g
421 Enterococcus faecalis V583 GCA_000007785.1
374 Streptomyces cinnamoneus GCA_001885705.1
338 Enterococcus faecium DO GCA_000174395.2
2h
766 Rhodobacter sphaeroides 2.4.1 GCA_000012905.2

712 Rhodobacter sphaeroides ATCC 17025 GCA_000016405.1
556 Defluviimonas alba GCA_001620265.1
556 Pseudorhodobacter ferrugineus DSM 5888 GCA_000420745.1
2i
745 Lactobacillus gasseri ATCC 33323 = JCM 1131 GCA_000014425.1
701 Lactobacillus hominis DSM 23910 = CRBIP 24.179 GCA_000296835.1
457 Lactobacillus iners DSM 13335 GCA_000160875.1
2j
588 Escherichia coli str. K-12 substr. MG1655 GCA_000005845.2
563 Escherichia coli O104_3AH4 str. 2011C-3493 GCA_000299455.1
563 Escherichia coli O157_3AH7 str. Sakai GCA_000008865.1
563 Shigella flexneri 2a str. 301 GCA_000006925.2
558 Escherichia coli UMN026 GCA_000026325.2
2k
857 Streptococcus mutans UA159 GCA_000007465.2
630 Streptococcus ratti FA-1 = DSM 20564 GCA_000286075.1
591 Streptococcus ferus DSM 20646 GCA_000372425.1
2l
780 Staphylococcus epidermidis ATCC 12228 GCA_000007645.1
757 Staphylococcus capitis subsp. capitis GCA_001028645.1
703 Staphylococcus warneri SG1 GCA_000332735.1
2m
701 Lactobacillus gasseri ATCC 33323 = JCM 1131 GCA_000014425.1
686 Lactobacillus hominis DSM 23910 = CRBIP 24.179 GCA_000296835.1
550 Lactobacillus iners DSM 13335 GCA_000160875.1
2n
713 Rhodobacter sphaeroides 2.4.1 GCA_000012905.2
611 Rhodobacter sphaeroides ATCC 17025 GCA_000016405.1
472 Gemmobacter aquatilis GCA_900110025.1
2o
702 Escherichia coli str. K-12 substr. MG1655 GCA_000005845.2
688 Escherichia coli O157_3AH7 str. Sakai GCA_000008865.1
688 Escherichia coli UMN026 GCA_000026325.2
684 Escherichia coli IAI39 GCA_000026345.1
2p
712 Enterococcus faecalis V583 GCA_000007785.1
665 Streptomyces cinnamoneus GCA_001885705.1
599 Enterococcus rivorum GCA_001742285.1
2q
541 Staphylococcus epidermidis ATCC 12228 GCA_000007645.1
514 Staphylococcus capitis subsp. capitis GCA_001028645.1
514 Staphylococcus haemolyticus JCSC1435 GCA_000009865.1
499 Staphylococcus aureus subsp. aureus NCTC 8325 GCA_000013425.1
499 Staphylococcus hominis subsp. hominis C80 GCA_000183685.1
499 Staphylococcus lugdunensis HKU09-01 GCA_000025085.1
2r
826 Clostridium beijerinckii GCA_000833105.2
767 Clostridium saccharoperbutylacetonicum N1-4_28HMT_29 GCA_000340885.1
755 Clostridium butyricum GCA_001456065.2
2s
845 Clostridium beijerinckii GCA_000833105.2
803 Clostridium saccharoperbutylacetonicum N1-4_28HMT_29 GCA_000340885.1
775 Clostridium butyricum GCA_001456065.2
775 Clostridium saccharobutylicum DSM 13864 GCA_000473995.1
2t
921 Staphylococcus epidermidis ATCC 12228 GCA_000007645.1
865 Staphylococcus capitis subsp. capitis GCA_001028645.1
780 Staphylococcus aureus subsp. aureus NCTC 8325 GCA_000013425.1
2u
916 Lactobacillus gasseri ATCC 33323 = JCM 1131 GCA_000014425.1
813 Lactobacillus hominis DSM 23910 = CRBIP 24.179 GCA_000296835.1
593 Lactobacillus iners DSM 13335 GCA_000160875.1
2v
589 Lactobacillus gasseri ATCC 33323 = JCM 1131 GCA_000014425.1
555 Lactobacillus hominis DSM 23910 = CRBIP 24.179 GCA_000296835.1
457 Lactobacillus iners DSM 13335 GCA_000160875.1
2w
408 Clostridium beijerinckii GCA_000833105.2
388 Clostridium saccharobutylicum DSM 13864 GCA_000473995.1
375 Clostridium saccharoperbutylacetonicum N1-4_28HMT_29 GCA_000340885.1
2x
584 Rhodobacter sphaeroides 2.4.1 GCA_000012905.2
549 Rhodobacter sphaeroides ATCC 17025 GCA_000016405.1
503 Gemmobacter aquatilis GCA_900110025.1
2y
700 Deinococcus radiodurans R1 GCA_000008565.1

478 Deinococcus gobiensis I-0 GCA_000252445.1
463 Deinococcus deserti VCD115 GCA_000020685.1
2z
903 Clostridium beijerinckii GCA_000833105.2
895 Clostridium saccharoperbutylacetonicum N1-4_28HMT_29 GCA_000340885.1
888 Clostridium saccharobutylicum DSM 13864 GCA_000473995.1
2A
578 Rhodobacter sphaeroides 2.4.1 GCA_000012905.2
501 Rhodobacter sphaeroides ATCC 17025 GCA_000016405.1
426 Gemmobacter aquatilis GCA_900110025.1
426 Rhodovulum sulfidophilum DSM 1374 GCA_001633165.1
2B
516 Rhodobacter sphaeroides 2.4.1 GCA_000012905.2
425 Rhodobacter sphaeroides ATCC 17025 GCA_000016405.1
350 Pseudorhodobacter psychrotolerans GCA_001294535.1
2C
755 Clostridium beijerinckii GCA_000833105.2
736 Clostridium saccharoperbutylacetonicum N1-4_28HMT_29 GCA_000340885.1
712 Clostridium puniceum GCA_002006345.1
2D
442 Rhodobacter sphaeroides 2.4.1 GCA_000012905.2
389 Rhodobacter sphaeroides ATCC 17025 GCA_000016405.1
292 Gemmobacter aquatilis GCA_900110025.1
2E
859 Lactobacillus gasseri ATCC 33323 = JCM 1131 GCA_000014425.1
801 Lactobacillus hominis DSM 23910 = CRBIP 24.179 GCA_000296835.1
617 Lactobacillus iners DSM 13335 GCA_000160875.1
2F
581 Enterococcus faecalis V583 GCA_000007785.1
552 Enterococcus canis NBRC 100695 GCA_001544375.1
547 Enterococcus phoeniculicola ATCC BAA-412 GCA_000407505.1
2G
718 Lactobacillus gasseri ATCC 33323 = JCM 1131 GCA_000014425.1
639 Lactobacillus hominis DSM 23910 = CRBIP 24.179 GCA_000296835.1
419 Lactobacillus iners DSM 13335 GCA_000160875.1
2H
740 Bifidobacterium adolescentis ATCC 15703 GCA_000010425.1
623 Bifidobacterium dentium JCM 1195 = DSM 20436 GCA_001042595.1
594 Bifidobacterium angulatum DSM 20098 = JCM 7096 GCA_001025155.1
2I
544 Lactobacillus gasseri ATCC 33323 = JCM 1131 GCA_000014425.1
465 Lactobacillus hominis DSM 23910 = CRBIP 24.179 GCA_000296835.1
348 Lactobacillus iners DSM 13335 GCA_000160875.1
2J
325 Staphylococcus epidermidis ATCC 12228 GCA_000007645.1
305 Staphylococcus capitis subsp. capitis GCA_001028645.1
274 Staphylococcus lugdunensis HKU09-01 GCA_000025085.1
2K
788 Bifidobacterium adolescentis ATCC 15703 GCA_000010425.1
642 Bifidobacterium dentium JCM 1195 = DSM 20436 GCA_001042595.1
601 Bifidobacterium angulatum DSM 20098 = JCM 7096 GCA_001025155.1
2L
787 Staphylococcus epidermidis ATCC 12228 GCA_000007645.1
726 Staphylococcus capitis subsp. capitis GCA_001028645.1
684 Staphylococcus aureus subsp. aureus NCTC 8325 GCA_000013425.1
2M
604 Bacillus thuringiensis YBT-1518 GCA_000497525.2
588 Bacillus anthracis str. Ames GCA_000007845.1
588 Bacillus anthracis str. Sterne GCA_000008165.1
588 Bacillus cereus ATCC 14579 GCA_000007825.1
588 _5BBacillus thuringiensis_5D serovar konkukian str. 97-27 GCA_000008505.1
565 Bacillus pseudomycoides DSM 12442 GCA_000161455.1
2N
896 Enterococcus faecalis V583 GCA_000007785.1
849 Streptomyces cinnamoneus GCA_001885705.1
783 Enterococcus rivorum GCA_001742285.1
2O
680 Lactobacillus gasseri ATCC 33323 = JCM 1131 GCA_000014425.1
600 Lactobacillus hominis DSM 23910 = CRBIP 24.179 GCA_000296835.1
407 Lactobacillus jensenii GCA_001936235.1
407 Lactobacillus psittaci DSM 15354 GCA_000425905.1
2P
923 Lactobacillus gasseri ATCC 33323 = JCM 1131 GCA_000014425.1
860 Lactobacillus hominis DSM 23910 = CRBIP 24.179 GCA_000296835.1
627 Lactobacillus iners DSM 13335 GCA_000160875.1
2Q

706 Lactobacillus gasseri ATCC 33323 = JCM 1131 GCA_000014425.1
660 Lactobacillus hominis DSM 23910 = CRBIP 24.179 GCA_000296835.1
449 Lactobacillus iners DSM 13335 GCA_000160875.1
2R
591 Clostridium beijerinckii GCA_000833105.2
578 Clostridium saccharobutylicum DSM 13864 GCA_000473995.1
574 Clostridium puniceum GCA_002006345.1
2S
776 Enterococcus faecalis V583 GCA_000007785.1
729 Streptomyces cinnamoneus GCA_001885705.1
653 Enterococcus rivorum GCA_001742285.1
2T
609 Clostridium beijerinckii GCA_000833105.2
587 Clostridium saccharoperbutylacetonicum N1-4_28HMT_29 GCA_000340885.1
547 Clostridium puniceum GCA_002006345.1
2U
858 Enterococcus faecalis V583 GCA_000007785.1
814 Streptomyces cinnamoneus GCA_001885705.1
773 Enterococcus rivorum GCA_001742285.1
2V
1085 Streptococcus mutans UA159 GCA_000007465.2
807 Streptococcus ratti FA-1 = DSM 20564 GCA_000286075.1
757 Streptococcus gordonii str. Challis substr. CH1 GCA_000017005.1
2W
816 Clostridium beijerinckii GCA_000833105.2
771 Clostridium saccharoperbutylacetonicum N1-4_28HMT_29 GCA_000340885.1
758 Clostridium puniceum GCA_002006345.1
2X
464 Bifidobacterium adolescentis ATCC 15703 GCA_000010425.1
385 Bifidobacterium dentium JCM 1195 = DSM 20436 GCA_001042595.1
370 Bifidobacterium angulatum DSM 20098 = JCM 7096 GCA_001025155.1
2Y
376 Lactobacillus gasseri ATCC 33323 = JCM 1131 GCA_000014425.1
332 Lactobacillus hominis DSM 23910 = CRBIP 24.179 GCA_000296835.1
253 Lactobacillus iners DSM 13335 GCA_000160875.1
2Z
705 Clostridium beijerinckii GCA_000833105.2
676 Clostridium saccharoperbutylacetonicum N1-4_28HMT_29 GCA_000340885.1
648 Clostridium saccharobutylicum DSM 13864 GCA_000473995.1
30
792 Enterococcus faecalis V583 GCA_000007785.1
748 Streptomyces cinnamoneus GCA_001885705.1
710 Enterococcus rivorum GCA_001742285.1
31
839 Enterococcus faecalis V583 GCA_000007785.1
792 Streptomyces cinnamoneus GCA_001885705.1
712 Enterococcus rivorum GCA_001742285.1
32
841 Enterococcus faecalis V583 GCA_000007785.1
794 Streptomyces cinnamoneus GCA_001885705.1
735 Enterococcus haemoperoxidus ATCC BAA-382 GCA_000407165.1
33
595 Streptococcus mutans UA159 GCA_000007465.2
441 Streptococcus ratti FA-1 = DSM 20564 GCA_000286075.1
419 Streptococcus equinus GCA_000964315.1
419 Streptococcus gallolyticus subsp. gallolyticus DSM 16831 GCA_002000985.1
34
830 Lactobacillus gasseri ATCC 33323 = JCM 1131 GCA_000014425.1
759 Lactobacillus hominis DSM 23910 = CRBIP 24.179 GCA_000296835.1
525 Lactobacillus iners DSM 13335 GCA_000160875.1
35
754 Staphylococcus epidermidis ATCC 12228 GCA_000007645.1
702 Staphylococcus capitis subsp. capitis GCA_001028645.1
633 Staphylococcus aureus subsp. aureus NCTC 8325 GCA_000013425.1
36
777 Streptococcus mutans UA159 GCA_000007465.2
581 Streptococcus ratti FA-1 = DSM 20564 GCA_000286075.1
532 Streptococcus gordonii str. Challis substr. CH1 GCA_000017005.1
37
737 Deinococcus radiodurans R1 GCA_000008565.1
423 Deinococcus deserti VCD115 GCA_000020685.1
419 Deinococcus puniceus GCA_001644565.1
38
753 Streptococcus mutans UA159 GCA_000007465.2
501 Streptococcus ratti FA-1 = DSM 20564 GCA_000286075.1
457 Streptococcus gordonii str. Challis substr. CH1 GCA_000017005.1

39
684 Clostridium beijerinckii GCA_000833105.2
635 Clostridium saccharoperbutylacetonicum N1-4_28HMT_29 GCA_000340885.1
628 Clostridium puniceum GCA_002006345.1
3a
527 Clostridium beijerinckii GCA_000833105.2
482 Clostridium puniceum GCA_002006345.1
482 Clostridium saccharoperbutylacetonicum N1-4_28HMT_29 GCA_000340885.1
463 Clostridium saccharobutylicum DSM 13864 GCA_000473995.1
3b
648 Streptococcus mutans UA159 GCA_000007465.2
446 Streptococcus ratti FA-1 = DSM 20564 GCA_000286075.1
433 Streptococcus ferus DSM 20646 GCA_000372425.1
3c
366 Staphylococcus epidermidis ATCC 12228 GCA_000007645.1
358 Staphylococcus haemolyticus JCSC1435 GCA_000009865.1
343 Staphylococcus hominis subsp. hominis C80 GCA_000183685.1
343 Staphylococcus lugdunensis HKU09-01 GCA_000025085.1
3d
711 Bifidobacterium adolescentis ATCC 15703 GCA_000010425.1
561 Bifidobacterium dentium JCM 1195 = DSM 20436 GCA_001042595.1
542 Bifidobacterium angulatum DSM 20098 = JCM 7096 GCA_001025155.1
3e
722 Deinococcus radiodurans R1 GCA_000008565.1
444 Deinococcus deserti VCD115 GCA_000020685.1
403 Deinococcus gobiensis I-0 GCA_000252445.1
3f
570 Clostridium beijerinckii GCA_000833105.2
570 Clostridium saccharoperbutylacetonicum N1-4_28HMT_29 GCA_000340885.1
549 Clostridium puniceum GCA_002006345.1
527 Clostridium butyricum GCA_001456065.2
3g
643 Deinococcus radiodurans R1 GCA_000008565.1
468 Deinococcus gobiensis I-0 GCA_000252445.1
433 Deinococcus soli Cha et al. 2016 GCA_001007995.1
3h
617 Clostridium beijerinckii GCA_000833105.2
595 Clostridium saccharoperbutylacetonicum N1-4_28HMT_29 GCA_000340885.1
572 Clostridium saccharobutylicum DSM 13864 GCA_000473995.1
3i
782 Enterococcus faecalis V583 GCA_000007785.1
735 Streptomyces cinnamoneus GCA_001885705.1
693 Enterococcus rivorum GCA_001742285.1
3j
716 Clostridium beijerinckii GCA_000833105.2
691 Clostridium saccharoperbutylacetonicum N1-4_28HMT_29 GCA_000340885.1
672 Clostridium saccharobutylicum DSM 13864 GCA_000473995.1
3k
711 Bifidobacterium adolescentis ATCC 15703 GCA_000010425.1
615 Bifidobacterium dentium JCM 1195 = DSM 20436 GCA_001042595.1
565 Bifidobacterium angulatum DSM 20098 = JCM 7096 GCA_001025155.1
3l
893 Lactobacillus gasseri ATCC 33323 = JCM 1131 GCA_000014425.1
826 Lactobacillus hominis DSM 23910 = CRBIP 24.179 GCA_000296835.1
653 Lactobacillus iners DSM 13335 GCA_000160875.1
3m
729 Streptococcus mutans UA159 GCA_000007465.2
503 Streptococcus ratti FA-1 = DSM 20564 GCA_000286075.1
498 Streptococcus macacae NCTC 11558 GCA_000187995.3
3n
732 Escherichia coli O104_3AH4 str. 2011C-3493 GCA_000299455.1
732 Escherichia coli str. K-12 substr. MG1655 GCA_000005845.2
724 Escherichia coli IAI39 GCA_000026345.1
717 Escherichia coli O157_3AH7 str. Sakai GCA_000008865.1
717 Shigella flexneri 2a str. 301 GCA_000006925.2
3o
462 Streptococcus mutans UA159 GCA_000007465.2
360 Streptococcus ferus DSM 20646 GCA_000372425.1
355 Streptococcus ratti FA-1 = DSM 20564 GCA_000286075.1
3p
815 Enterococcus faecalis V583 GCA_000007785.1
768 Streptomyces cinnamoneus GCA_001885705.1
700 Enterococcus asini ATCC 700915 GCA_000407365.1
3q
551 Streptococcus mutans UA159 GCA_000007465.2
418 Streptococcus ratti FA-1 = DSM 20564 GCA_000286075.1

361 Streptococcus equinus GCA_000964315.1
3r
724 Streptococcus mutans UA159 GCA_000007465.2
528 Streptococcus gordonii str. Challis substr. CH1 GCA_000017005.1
508 Streptococcus ratti FA-1 = DSM 20564 GCA_000286075.1
3s
644 Lactobacillus gasseri ATCC 33323 = JCM 1131 GCA_000014425.1
582 Lactobacillus hominis DSM 23910 = CRBIP 24.179 GCA_000296835.1
447 Lactobacillus iners DSM 13335 GCA_000160875.1
3t
837 Deinococcus radiodurans R1 GCA_000008565.1
533 Deinococcus deserti VCD115 GCA_000020685.1
509 Deinococcus gobiensis I-0 GCA_000252445.1
3u
804 Staphylococcus epidermidis ATCC 12228 GCA_000007645.1
789 Staphylococcus capitis subsp. capitis GCA_001028645.1
703 Staphylococcus aureus subsp. aureus NCTC 8325 GCA_000013425.1
3v
670 Escherichia coli str. K-12 substr. MG1655 GCA_000005845.2
659 Escherichia coli O104_3AH4 str. 2011C-3493 GCA_000299455.1
644 Shigella flexneri 2a str. 301 GCA_000006925.2
3w
795 Bifidobacterium adolescentis ATCC 15703 GCA_000010425.1
699 Bifidobacterium angulatum DSM 20098 = JCM 7096 GCA_001025155.1
668 Bifidobacterium longum NCC2705 GCA_000007525.1
3x
708 Bifidobacterium adolescentis ATCC 15703 GCA_000010425.1
633 Bifidobacterium dentium JCM 1195 = DSM 20436 GCA_001042595.1
631 Bifidobacterium angulatum DSM 20098 = JCM 7096 GCA_001025155.1
3y
416 Clostridium beijerinckii GCA_000833105.2
398 Clostridium saccharoperbutylacetonicum N1-4_28HMT_29 GCA_000340885.1
374 Clostridium butyricum GCA_001456065.2
3z
752 Clostridium beijerinckii GCA_000833105.2
714 Clostridium saccharoperbutylacetonicum N1-4_28HMT_29 GCA_000340885.1
696 Clostridium puniceum GCA_002006345.1
3A
800 Bacillus thuringiensis YBT-1518 GCA_000497525.2
789 Bacillus anthracis str. Ames GCA_000007845.1
789 Bacillus anthracis str. Sterne GCA_000008165.1
789 Bacillus cereus ATCC 14579 GCA_000007825.1
789 _5BBacillus thuringiensis_5D serovar konkukian str. 97-27 GCA_000008505.1
784 Bacillus pseudomycoides DSM 12442 GCA_000161455.1
3B
605 Deinococcus radiodurans R1 GCA_000008565.1
396 Deinococcus deserti VCD115 GCA_000020685.1
355 Deinococcus proteolyticus MRP GCA_000190555.1
3C
657 Rhodobacter sphaeroides 2.4.1 GCA_000012905.2
622 Rhodobacter sphaeroides ATCC 17025 GCA_000016405.1
551 Pseudorhodobacter psychrotolerans GCA_001294535.1
3D
720 Staphylococcus epidermidis ATCC 12228 GCA_000007645.1
633 Staphylococcus capitis subsp. capitis GCA_001028645.1
578 Staphylococcus hominis subsp. hominis C80 GCA_000183685.1
3E
738 Staphylococcus epidermidis ATCC 12228 GCA_000007645.1
715 Staphylococcus capitis subsp. capitis GCA_001028645.1
629 Staphylococcus lugdunensis HKU09-01 GCA_000025085.1
3F
793 Deinococcus radiodurans R1 GCA_000008565.1
494 Deinococcus deserti VCD115 GCA_000020685.1
449 Deinococcus gobiensis I-0 GCA_000252445.1
3G
662 Clostridium beijerinckii GCA_000833105.2
611 Clostridium puniceum GCA_002006345.1
611 Clostridium saccharobutylicum DSM 13864 GCA_000473995.1
611 Clostridium saccharoperbutylacetonicum N1-4_28HMT_29 GCA_000340885.1
582 Clostridium butyricum GCA_001456065.2
3H
669 Enterococcus faecalis V583 GCA_000007785.1
622 Streptomyces cinnamoneus GCA_001885705.1
600 Enterococcus faecium DO GCA_000174395.2
3I
775 Deinococcus radiodurans R1 GCA_000008565.1

468 Deinococcus soli Cha et al. 2016 GCA_001007995.1
436 Deinococcus deserti VCD115 GCA_000020685.1
3J
586 Streptococcus mutans UA159 GCA_000007465.2
440 Streptococcus ratti FA-1 = DSM 20564 GCA_000286075.1
424 Streptococcus halotolerans GCA_001598035.1
3K
761 Staphylococcus epidermidis ATCC 12228 GCA_000007645.1
747 Staphylococcus capitis subsp. capitis GCA_001028645.1
672 Staphylococcus warneri SG1 GCA_000332735.1
3L
857 Staphylococcus epidermidis ATCC 12228 GCA_000007645.1
808 Staphylococcus capitis subsp. capitis GCA_001028645.1
782 Megasphaera cerevisiae DSM 20462 GCA_001045675.1
782 Staphylococcus warneri SG1 GCA_000332735.1
3M
548 Streptococcus mutans UA159 GCA_000007465.2
347 Streptococcus ratti FA-1 = DSM 20564 GCA_000286075.1
330 Streptococcus macacae NCTC 11558 GCA_000187995.3
3N
503 Escherichia coli IAI39 GCA_000026345.1
503 Escherichia coli O157_3AH7 str. Sakai GCA_000008865.1
503 Escherichia coli str. K-12 substr. MG1655 GCA_000005845.2
495 Escherichia coli UMN026 GCA_000026325.2
477 Escherichia coli O83_3AH1 str. NRG 857C GCA_000183345.1
3O
438 Clostridium beijerinckii GCA_000833105.2
406 Clostridium saccharoperbutylacetonicum N1-4_28HMT_29 GCA_000340885.1
385 Clostridium saccharobutylicum DSM 13864 GCA_000473995.1
3P
654 Staphylococcus epidermidis ATCC 12228 GCA_000007645.1
605 Staphylococcus capitis subsp. capitis GCA_001028645.1
552 Megasphaera cerevisiae DSM 20462 GCA_001045675.1
552 Staphylococcus warneri SG1 GCA_000332735.1
3Q
642 Bacillus anthracis str. Ames GCA_000007845.1
642 Bacillus anthracis str. Sterne GCA_000008165.1
642 Bacillus cereus ATCC 14579 GCA_000007825.1
642 _5BBacillus thuringiensis_5D serovar konkukian str. 97-27 GCA_000008505.1
621 Bacillus thuringiensis YBT-1518 GCA_000497525.2
620 Bacillus mycoides GCA_000832605.1
3R
784 Streptococcus mutans UA159 GCA_000007465.2
575 Streptococcus ratti FA-1 = DSM 20564 GCA_000286075.1
531 Streptococcus gordonii str. Challis substr. CH1 GCA_000017005.1
3S
784 Deinococcus radiodurans R1 GCA_000008565.1
531 Deinococcus deserti VCD115 GCA_000020685.1
485 Deinococcus gobiensis I-0 GCA_000252445.1
3T
752 Lactobacillus gasseri ATCC 33323 = JCM 1131 GCA_000014425.1
730 Lactobacillus hominis DSM 23910 = CRBIP 24.179 GCA_000296835.1
553 Lactobacillus iners DSM 13335 GCA_000160875.1
3U
454 Clostridium saccharoperbutylacetonicum N1-4_28HMT_29 GCA_000340885.1
453 Clostridium beijerinckii GCA_000833105.2
451 Clostridium saccharobutylicum DSM 13864 GCA_000473995.1
3V
750 Staphylococcus epidermidis ATCC 12228 GCA_000007645.1
733 Staphylococcus capitis subsp. capitis GCA_001028645.1
673 Staphylococcus warneri SG1 GCA_000332735.1
3W
598 Clostridium beijerinckii GCA_000833105.2
588 Clostridium saccharoperbutylacetonicum N1-4_28HMT_29 GCA_000340885.1
559 Clostridium puniceum GCA_002006345.1
3X
723 Enterococcus faecalis V583 GCA_000007785.1
677 Streptomyces cinnamoneus GCA_001885705.1
622 Enterococcus haemoperoxidus ATCC BAA-382 GCA_000407165.1
3Y
613 Clostridium beijerinckii GCA_000833105.2
576 Clostridium puniceum GCA_002006345.1
576 Clostridium saccharoperbutylacetonicum N1-4_28HMT_29 GCA_000340885.1
561 Clostridium butyricum GCA_001456065.2
3Z
768 Deinococcus radiodurans R1 GCA_000008565.1

507 Deinococcus deserti VCD115 GCA_000020685.1
456 Deinococcus murrayi DSM 11303 GCA_000482805.1
40
683 Staphylococcus epidermidis ATCC 12228 GCA_000007645.1
626 Staphylococcus capitis subsp. capitis GCA_001028645.1
518 Staphylococcus aureus subsp. aureus NCTC 8325 GCA_000013425.1
41
468 Rhodobacter sphaeroides 2.4.1 GCA_000012905.2
403 Rhodobacter sphaeroides ATCC 17025 GCA_000016405.1
318 Defluviimonas indica GCA_900106675.1
42
738 Escherichia coli O157_3AH7 str. Sakai GCA_000008865.1
738 Escherichia coli str. K-12 substr. MG1655 GCA_000005845.2
719 Escherichia coli IAI39 GCA_000026345.1
709 Escherichia coli UMN026 GCA_000026325.2
43
475 Escherichia coli O157_3AH7 str. Sakai GCA_000008865.1
475 Escherichia coli str. K-12 substr. MG1655 GCA_000005845.2
471 Escherichia coli IAI39 GCA_000026345.1
470 Escherichia coli UMN026 GCA_000026325.2
44
690 Enterococcus faecalis V583 GCA_000007785.1
643 Streptomyces cinnamoneus GCA_001885705.1
620 Enterococcus rivorum GCA_001742285.1
45
851 Bacillus cereus ATCC 14579 GCA_000007825.1
851 Bacillus thuringiensis YBT-1518 GCA_000497525.2
849 Bacillus anthracis str. Ames GCA_000007845.1
849 Bacillus anthracis str. Sterne GCA_000008165.1
849 _5BBacillus thuringiensis_5D serovar konkukian str. 97-27 GCA_000008505.1
834 Bacillus pseudomycoides DSM 12442 GCA_000161455.1
46
349 Clostridium saccharoperbutylacetonicum N1-4_28HMT_29 GCA_000340885.1
346 Clostridium beijerinckii GCA_000833105.2
344 Clostridium butyricum GCA_001456065.2
344 Clostridium saccharobutylicum DSM 13864 GCA_000473995.1
47
794 Streptococcus mutans UA159 GCA_000007465.2
550 Streptococcus ratti FA-1 = DSM 20564 GCA_000286075.1
510 Streptococcus gordonii str. Challis substr. CH1 GCA_000017005.1
48
728 Clostridium beijerinckii GCA_000833105.2
700 Clostridium saccharoperbutylacetonicum N1-4_28HMT_29 GCA_000340885.1
681 Clostridium puniceum GCA_002006345.1
49
494 Rhodobacter sphaeroides 2.4.1 GCA_000012905.2
389 Gemmobacter aquatilis GCA_900110025.1
383 Rhodobacter sphaeroides ATCC 17025 GCA_000016405.1
4a
432 Clostridium beijerinckii GCA_000833105.2
415 Clostridium saccharoperbutylacetonicum N1-4_28HMT_29 GCA_000340885.1
393 Clostridium butyricum GCA_001456065.2
4b
774 Streptococcus mutans UA159 GCA_000007465.2
599 Streptococcus ratti FA-1 = DSM 20564 GCA_000286075.1
537 Streptococcus ferus DSM 20646 GCA_000372425.1
4c
746 Streptococcus mutans UA159 GCA_000007465.2
488 Streptococcus gordonii str. Challis substr. CH1 GCA_000017005.1
486 Streptococcus ratti FA-1 = DSM 20564 GCA_000286075.1
4d
1002 Streptococcus mutans UA159 GCA_000007465.2
762 Streptococcus ratti FA-1 = DSM 20564 GCA_000286075.1
666 Streptococcus gordonii str. Challis substr. CH1 GCA_000017005.1
4e
780 Lactobacillus gasseri ATCC 33323 = JCM 1131 GCA_000014425.1
719 Lactobacillus hominis DSM 23910 = CRBIP 24.179 GCA_000296835.1
554 Lactobacillus iners DSM 13335 GCA_000160875.1
4f
708 Staphylococcus epidermidis ATCC 12228 GCA_000007645.1
661 Staphylococcus capitis subsp. capitis GCA_001028645.1
655 Staphylococcus haemolyticus JCSC1435 GCA_000009865.1
4g
702 Rhodobacter sphaeroides 2.4.1 GCA_000012905.2
621 Rhodobacter sphaeroides ATCC 17025 GCA_000016405.1
500 Defluviimonas alba GCA_001620265.1

4h
774 Enterococcus faecalis V583 GCA_000007785.1
731 Enterococcus canis NBRC 100695 GCA_001544375.1
727 Streptomyces cinnamoneus GCA_001885705.1
4i
926 Clostridium beijerinckii GCA_000833105.2
898 Clostridium saccharoperbutylacetonicum N1-4_28HMT_29 GCA_000340885.1
876 Clostridium puniceum GCA_002006345.1
4j
929 Staphylococcus epidermidis ATCC 12228 GCA_000007645.1
877 Staphylococcus capitis subsp. capitis GCA_001028645.1
836 Staphylococcus haemolyticus JCSC1435 GCA_000009865.1
4k
434 Escherichia coli O104_3AH4 str. 2011C-3493 GCA_000299455.1
434 Escherichia coli O157_3AH7 str. Sakai GCA_000008865.1
434 Escherichia coli O83_3AH1 str. NRG 857C GCA_000183345.1
434 Escherichia coli UMN026 GCA_000026325.2
434 Escherichia coli str. K-12 substr. MG1655 GCA_000005845.2
434 Shigella flexneri 2a str. 301 GCA_000006925.2
409 Escherichia coli IAI39 GCA_000026345.1
406 Salmonella enterica subsp. enterica serovar Typhimurium str. LT2 GCA_000006945.2
4l
598 Staphylococcus epidermidis ATCC 12228 GCA_000007645.1
559 Staphylococcus capitis subsp. capitis GCA_001028645.1
527 Staphylococcus hominis subsp. hominis C80 GCA_000183685.1
4m
591 Staphylococcus epidermidis ATCC 12228 GCA_000007645.1
515 Staphylococcus capitis subsp. capitis GCA_001028645.1
513 Staphylococcus haemolyticus JCSC1435 GCA_000009865.1
513 Staphylococcus hominis subsp. hominis C80 GCA_000183685.1
4n
613 Lactobacillus gasseri ATCC 33323 = JCM 1131 GCA_000014425.1
576 Lactobacillus hominis DSM 23910 = CRBIP 24.179 GCA_000296835.1
475 Lactobacillus iners DSM 13335 GCA_000160875.1
4o
681 Streptococcus mutans UA159 GCA_000007465.2
501 Streptococcus ratti FA-1 = DSM 20564 GCA_000286075.1
469 Streptococcus macacae NCTC 11558 GCA_000187995.3
4p
488 Bacillus thuringiensis YBT-1518 GCA_000497525.2
480 Bacillus anthracis str. Ames GCA_000007845.1
480 Bacillus anthracis str. Sterne GCA_000008165.1
480 Bacillus pseudomycoides DSM 12442 GCA_000161455.1
480 _5BBacillus thuringiensis_5D serovar konkukian str. 97-27 GCA_000008505.1
479 Bacillus cereus ATCC 14579 GCA_000007825.1
4q
599 Streptococcus mutans UA159 GCA_000007465.2
464 Streptococcus ratti FA-1 = DSM 20564 GCA_000286075.1
432 Streptococcus gordonii str. Challis substr. CH1 GCA_000017005.1
4r
644 Bifidobacterium adolescentis ATCC 15703 GCA_000010425.1
552 Bifidobacterium dentium JCM 1195 = DSM 20436 GCA_001042595.1
529 Bifidobacterium angulatum DSM 20098 = JCM 7096 GCA_001025155.1
4s
717 Bacillus anthracis str. Ames GCA_000007845.1
717 Bacillus anthracis str. Sterne GCA_000008165.1
717 Bacillus cereus ATCC 14579 GCA_000007825.1
717 _5BBacillus thuringiensis_5D serovar konkukian str. 97-27 GCA_000008505.1
712 Bacillus thuringiensis YBT-1518 GCA_000497525.2
701 Bacillus pseudomycoides DSM 12442 GCA_000161455.1
4t
807 Clostridium beijerinckii GCA_000833105.2
755 Clostridium saccharobutylicum DSM 13864 GCA_000473995.1
747 Clostridium puniceum GCA_002006345.1
4u
750 Escherichia coli str. K-12 substr. MG1655 GCA_000005845.2
739 Escherichia coli O104_3AH4 str. 2011C-3493 GCA_000299455.1
739 Escherichia coli O157_3AH7 str. Sakai GCA_000008865.1
739 Shigella flexneri 2a str. 301 GCA_000006925.2
735 Escherichia coli IAI39 GCA_000026345.1
735 Shigella dysenteriae Sd197 GCA_000012005.1
4v
483 Streptococcus mutans UA159 GCA_000007465.2
389 Lactobacillus gasseri ATCC 33323 = JCM 1131 GCA_000014425.1
374 Lactobacillus hominis DSM 23910 = CRBIP 24.179 GCA_000296835.1
4w

765 Deinococcus radiodurans R1 GCA_000008565.1
452 Deinococcus deserti VCD115 GCA_000020685.1
413 Deinococcus soli Cha et al. 2016 GCA_001007995.1
4x
799 Clostridium beijerinckii GCA_000833105.2
775 Clostridium saccharoperbutylacetonicum N1-4_28HMT_29 GCA_000340885.1
743 Clostridium puniceum GCA_002006345.1
4y
509 Streptococcus mutans UA159 GCA_000007465.2
415 Streptococcus equinus GCA_000964315.1
402 Streptococcus anginosus C238 GCA_000463505.1
4z
758 Deinococcus radiodurans R1 GCA_000008565.1
481 Deinococcus deserti VCD115 GCA_000020685.1
477 Deinococcus gobiensis I-0 GCA_000252445.1
4A
899 Staphylococcus epidermidis ATCC 12228 GCA_000007645.1
858 Staphylococcus capitis subsp. capitis GCA_001028645.1
788 Staphylococcus aureus subsp. aureus NCTC 8325 GCA_000013425.1
4B
733 Staphylococcus epidermidis ATCC 12228 GCA_000007645.1
688 Staphylococcus capitis subsp. capitis GCA_001028645.1
654 Staphylococcus aureus subsp. aureus NCTC 8325 GCA_000013425.1
4C
737 Escherichia coli O157_3AH7 str. Sakai GCA_000008865.1
737 Escherichia coli str. K-12 substr. MG1655 GCA_000005845.2
731 Escherichia coli O83_3AH1 str. NRG 857C GCA_000183345.1
723 Shigella flexneri 2a str. 301 GCA_000006925.2
4D
825 Rhodobacter sphaeroides 2.4.1 GCA_000012905.2
780 Rhodobacter sphaeroides ATCC 17025 GCA_000016405.1
594 Gemmobacter aquatilis GCA_900110025.1
594 Pseudorhodobacter ferrugineus DSM 5888 GCA_000420745.1
4E
677 Deinococcus radiodurans R1 GCA_000008565.1
419 Deinococcus deserti VCD115 GCA_000020685.1
410 Deinococcus puniceus GCA_001644565.1
4F
801 Streptococcus mutans UA159 GCA_000007465.2
606 Streptococcus ratti FA-1 = DSM 20564 GCA_000286075.1
589 Streptococcus equinus GCA_000964315.1
4G
652 Staphylococcus epidermidis ATCC 12228 GCA_000007645.1
623 Staphylococcus capitis subsp. capitis GCA_001028645.1
593 Staphylococcus aureus subsp. aureus NCTC 8325 GCA_000013425.1
4H
686 Bifidobacterium adolescentis ATCC 15703 GCA_000010425.1
558 Bifidobacterium dentium JCM 1195 = DSM 20436 GCA_001042595.1
541 Bifidobacterium angulatum DSM 20098 = JCM 7096 GCA_001025155.1
4I
614 Staphylococcus epidermidis ATCC 12228 GCA_000007645.1
556 Staphylococcus capitis subsp. capitis GCA_001028645.1
526 Staphylococcus warneri SG1 GCA_000332735.1
4J
600 Rhodobacter sphaeroides 2.4.1 GCA_000012905.2
503 Rhodobacter sphaeroides ATCC 17025 GCA_000016405.1
459 Gemmobacter megaterium GCA_900156815.1
4K
535 Lactobacillus gasseri ATCC 33323 = JCM 1131 GCA_000014425.1
528 Clostridium beijerinckii GCA_000833105.2
515 Clostridium saccharoperbutylacetonicum N1-4_28HMT_29 GCA_000340885.1
4L
658 Streptococcus mutans UA159 GCA_000007465.2
505 Streptococcus ratti FA-1 = DSM 20564 GCA_000286075.1
470 Streptococcus gordonii str. Challis substr. CH1 GCA_000017005.1
4M
454 Bifidobacterium adolescentis ATCC 15703 GCA_000010425.1
395 Bifidobacterium dentium JCM 1195 = DSM 20436 GCA_001042595.1
356 Bifidobacterium stellenboschense GCA_000741785.1
4N
662 Clostridium beijerinckii GCA_000833105.2
618 Clostridium saccharoperbutylacetonicum N1-4_28HMT_29 GCA_000340885.1
590 Clostridium puniceum GCA_002006345.1
590 Clostridium saccharobutylicum DSM 13864 GCA_000473995.1
4O
703 Staphylococcus epidermidis ATCC 12228 GCA_000007645.1

698 Staphylococcus capitis subsp. capitis GCA_001028645.1
659 Staphylococcus aureus subsp. aureus NCTC 8325 GCA_000013425.1
4P
578 Rhodobacter sphaeroides 2.4.1 GCA_000012905.2
519 Rhodobacter sphaeroides ATCC 17025 GCA_000016405.1
432 Gemmobacter aquatilis GCA_900110025.1
432 Gemmobacter megaterium GCA_900156815.1
4Q
835 Bacillus anthracis str. Ames GCA_000007845.1
835 Bacillus anthracis str. Sterne GCA_000008165.1
835 _5BBacillus thuringiensis_5D serovar konkukian str. 97-27 GCA_000008505.1
824 Bacillus pseudomycoides DSM 12442 GCA_000161455.1
820 Bacillus cereus ATCC 14579 GCA_000007825.1
4R
594 Bacillus anthracis str. Ames GCA_000007845.1
594 Bacillus anthracis str. Sterne GCA_000008165.1
594 _5BBacillus thuringiensis_5D serovar konkukian str. 97-27 GCA_000008505.1
586 Bacillus cereus ATCC 14579 GCA_000007825.1
576 Bacillus pseudomycoides DSM 12442 GCA_000161455.1
4S
627 Lactobacillus gasseri ATCC 33323 = JCM 1131 GCA_000014425.1
561 Lactobacillus hominis DSM 23910 = CRBIP 24.179 GCA_000296835.1
475 Lactobacillus crispatus ST1 GCA_000091765.1
4T
453 Lactobacillus gasseri ATCC 33323 = JCM 1131 GCA_000014425.1
426 Lactobacillus hominis DSM 23910 = CRBIP 24.179 GCA_000296835.1
325 Lactobacillus iners DSM 13335 GCA_000160875.1
4U
647 Lactobacillus gasseri ATCC 33323 = JCM 1131 GCA_000014425.1
561 Lactobacillus hominis DSM 23910 = CRBIP 24.179 GCA_000296835.1
438 Lactobacillus iners DSM 13335 GCA_000160875.1
4V
353 Rhodobacter sphaeroides 2.4.1 GCA_000012905.2
316 Rhodobacter sphaeroides ATCC 17025 GCA_000016405.1
281 Rhodobacter vinaykumarii GCA_900156695.1
4W
720 Rhodobacter sphaeroides 2.4.1 GCA_000012905.2
608 Rhodobacter sphaeroides ATCC 17025 GCA_000016405.1
511 Gemmobacter megaterium GCA_900156815.1
4X
805 Clostridium beijerinckii GCA_000833105.2
794 Clostridium saccharoperbutylacetonicum N1-4_28HMT_29 GCA_000340885.1
751 Clostridium puniceum GCA_002006345.1
4Y
768 Streptococcus mutans UA159 GCA_000007465.2
579 Streptococcus ratti FA-1 = DSM 20564 GCA_000286075.1
565 Streptococcus gordonii str. Challis substr. CH1 GCA_000017005.1
4Z
767 Staphylococcus epidermidis ATCC 12228 GCA_000007645.1
754 Staphylococcus capitis subsp. capitis GCA_001028645.1
693 Staphylococcus aureus subsp. aureus NCTC 8325 GCA_000013425.1
50
870 Enterococcus faecalis V583 GCA_000007785.1
823 Streptomyces cinnamoneus GCA_001885705.1
783 Enterococcus rivorum GCA_001742285.1
51
818 Bifidobacterium adolescentis ATCC 15703 GCA_000010425.1
750 Bifidobacterium dentium JCM 1195 = DSM 20436 GCA_001042595.1
710 Bifidobacterium longum NCC2705 GCA_000007525.1
52
834 Lactobacillus gasseri ATCC 33323 = JCM 1131 GCA_000014425.1
771 Lactobacillus hominis DSM 23910 = CRBIP 24.179 GCA_000296835.1
586 Lactobacillus iners DSM 13335 GCA_000160875.1
53
884 Staphylococcus epidermidis ATCC 12228 GCA_000007645.1
840 Staphylococcus capitis subsp. capitis GCA_001028645.1
812 Staphylococcus aureus subsp. aureus NCTC 8325 GCA_000013425.1
54
700 Bifidobacterium adolescentis ATCC 15703 GCA_000010425.1
564 Bifidobacterium dentium JCM 1195 = DSM 20436 GCA_001042595.1
547 Bifidobacterium callitrichos DSM 23973 GCA_000741175.1
55
655 Streptococcus mutans UA159 GCA_000007465.2
432 Streptococcus gordonii str. Challis substr. CH1 GCA_000017005.1
415 Streptococcus ratti FA-1 = DSM 20564 GCA_000286075.1
56

721 Deinococcus radiodurans R1 GCA_000008565.1
403 Deinococcus deserti VCD115 GCA_000020685.1
394 Deinococcus hopiensis KR-140 GCA_900176165.1
57
772 Deinococcus radiodurans R1 GCA_000008565.1
441 Deinococcus deserti VCD115 GCA_000020685.1
441 Deinococcus gobiensis I-0 GCA_000252445.1
434 Deinococcus soli Cha et al. 2016 GCA_001007995.1
58
640 Deinococcus radiodurans R1 GCA_000008565.1
398 Deinococcus deserti VCD115 GCA_000020685.1
390 Deinococcus soli Cha et al. 2016 GCA_001007995.1
59
905 Deinococcus radiodurans R1 GCA_000008565.1
586 Deinococcus deserti VCD115 GCA_000020685.1
560 Deinococcus murrayi DSM 11303 GCA_000482805.1
5a
603 Rhodobacter sphaeroides 2.4.1 GCA_000012905.2
547 Rhodobacter sphaeroides ATCC 17025 GCA_000016405.1
446 Defluviimonas alba GCA_001620265.1
5b
435 Streptococcus mutans UA159 GCA_000007465.2
302 Streptococcus ratti FA-1 = DSM 20564 GCA_000286075.1
285 Streptococcus gordonii str. Challis substr. CH1 GCA_000017005.1
5c
606 Deinococcus radiodurans R1 GCA_000008565.1
403 Deinococcus deserti VCD115 GCA_000020685.1
391 Deinococcus soli Cha et al. 2016 GCA_001007995.1
5d
764 Lactobacillus gasseri ATCC 33323 = JCM 1131 GCA_000014425.1
679 Lactobacillus hominis DSM 23910 = CRBIP 24.179 GCA_000296835.1
523 Lactobacillus hamsteri DSM 5661 = JCM 6256 GCA_000615445.1
5e
462 Escherichia coli O157_3AH7 str. Sakai GCA_000008865.1
462 Escherichia coli str. K-12 substr. MG1655 GCA_000005845.2
462 Shigella flexneri 2a str. 301 GCA_000006925.2
461 Escherichia coli O104_3AH4 str. 2011C-3493 GCA_000299455.1
454 Escherichia coli IAI39 GCA_000026345.1
454 Escherichia coli UMN026 GCA_000026325.2
5f
626 Bifidobacterium adolescentis ATCC 15703 GCA_000010425.1
507 Bifidobacterium angulatum DSM 20098 = JCM 7096 GCA_001025155.1
485 Bifidobacterium callitrichos DSM 23973 GCA_000741175.1
5g
832 Deinococcus radiodurans R1 GCA_000008565.1
517 Deinococcus gobiensis I-0 GCA_000252445.1
497 Deinococcus deserti VCD115 GCA_000020685.1
5h
1023 Streptococcus mutans UA159 GCA_000007465.2
770 Streptococcus ratti FA-1 = DSM 20564 GCA_000286075.1
702 Streptococcus sobrinus DSM 20742 = ATCC 33478 GCA_000686605.1
5i
741 Clostridium beijerinckii GCA_000833105.2
711 Clostridium puniceum GCA_002006345.1
697 Clostridium saccharoperbutylacetonicum N1-4_28HMT_29 GCA_000340885.1
5j
413 Bifidobacterium adolescentis ATCC 15703 GCA_000010425.1
363 Bifidobacterium angulatum DSM 20098 = JCM 7096 GCA_001025155.1
346 Bifidobacterium breve DSM 20213 = JCM 1192 GCA_001025175.1
5k
645 Staphylococcus epidermidis ATCC 12228 GCA_000007645.1
619 Staphylococcus capitis subsp. capitis GCA_001028645.1
534 Staphylococcus haemolyticus JCSC1435 GCA_000009865.1
5l
783 Staphylococcus epidermidis ATCC 12228 GCA_000007645.1
752 Staphylococcus capitis subsp. capitis GCA_001028645.1
671 Staphylococcus aureus subsp. aureus NCTC 8325 GCA_000013425.1
671 Staphylococcus warneri SG1 GCA_000332735.1
5m
566 Enterococcus faecalis V583 GCA_000007785.1
539 Enterococcus rivorum GCA_001742285.1
523 Streptomyces cinnamoneus GCA_001885705.1
5n
740 Clostridium beijerinckii GCA_000833105.2
712 Clostridium saccharoperbutylacetonicum N1-4_28HMT_29 GCA_000340885.1
666 Clostridium saccharobutylicum DSM 13864 GCA_000473995.1

5o
737 Clostridium beijerinckii GCA_000833105.2
686 Clostridium saccharoperbutylacetonicum N1-4_28HMT_29 GCA_000340885.1
666 Clostridium puniceum GCA_002006345.1
5p
923 Lactobacillus gasseri ATCC 33323 = JCM 1131 GCA_000014425.1
852 Lactobacillus hominis DSM 23910 = CRBIP 24.179 GCA_000296835.1
656 Lactobacillus iners DSM 13335 GCA_000160875.1
5q
733 Staphylococcus epidermidis ATCC 12228 GCA_000007645.1
669 Staphylococcus capitis subsp. capitis GCA_001028645.1
626 Staphylococcus warneri SG1 GCA_000332735.1
5r
532 Rhodobacter sphaeroides 2.4.1 GCA_000012905.2
511 Rhodobacter sphaeroides ATCC 17025 GCA_000016405.1
416 Defluviimonas alba GCA_001620265.1
416 Thioclava indica GCA_000714545.1
5s
813 Streptococcus mutans UA159 GCA_000007465.2
613 Streptococcus ratti FA-1 = DSM 20564 GCA_000286075.1
580 Streptococcus merionis DSM 19192 GCA_000380085.1
5t
709 Clostridium beijerinckii GCA_000833105.2
660 Clostridium saccharoperbutylacetonicum N1-4_28HMT_29 GCA_000340885.1
643 Clostridium saccharobutylicum DSM 13864 GCA_000473995.1
5u
818 Streptococcus mutans UA159 GCA_000007465.2
621 Streptococcus ratti FA-1 = DSM 20564 GCA_000286075.1
589 Streptococcus gordonii str. Challis substr. CH1 GCA_000017005.1
5v
681 Deinococcus radiodurans R1 GCA_000008565.1
421 Deinococcus deserti VCD115 GCA_000020685.1
417 Deinococcus puniceus GCA_001644565.1
5w
578 Lactobacillus gasseri ATCC 33323 = JCM 1131 GCA_000014425.1
530 Lactobacillus hominis DSM 23910 = CRBIP 24.179 GCA_000296835.1
415 Lactobacillus jensenii GCA_001936235.1
415 Lactobacillus psittaci DSM 15354 GCA_000425905.1
5x
511 Staphylococcus epidermidis ATCC 12228 GCA_000007645.1
505 Streptomyces cinnamoneus GCA_001885705.1
480 Staphylococcus capitis subsp. capitis GCA_001028645.1
5y
746 Streptococcus mutans UA159 GCA_000007465.2
524 Streptococcus ferus DSM 20646 GCA_000372425.1
513 Streptococcus gordonii str. Challis substr. CH1 GCA_000017005.1
5z
664 Escherichia coli str. K-12 substr. MG1655 GCA_000005845.2
658 Escherichia coli IAI39 GCA_000026345.1
658 Escherichia coli O157_3AH7 str. Sakai GCA_000008865.1
658 Escherichia coli O83_3AH1 str. NRG 857C GCA_000183345.1
658 Escherichia coli UMN026 GCA_000026325.2
658 Shigella flexneri 2a str. 301 GCA_000006925.2
643 Shigella dysenteriae Sd197 GCA_000012005.1
5A
879 Bacillus anthracis str. Ames GCA_000007845.1
879 Bacillus anthracis str. Sterne GCA_000008165.1
879 _5BBacillus thuringiensis_5D serovar konkukian str. 97-27 GCA_000008505.1
853 Bacillus cereus ATCC 14579 GCA_000007825.1
842 Bacillus pseudomycoides DSM 12442 GCA_000161455.1
5B
787 Lactobacillus gasseri ATCC 33323 = JCM 1131 GCA_000014425.1
736 Lactobacillus hominis DSM 23910 = CRBIP 24.179 GCA_000296835.1
578 Lactobacillus iners DSM 13335 GCA_000160875.1
5C
1005 _5BBacillus thuringiensis_5D serovar konkukian str. 97-27 GCA_000008505.1
995 Bacillus anthracis str. Ames GCA_000007845.1
995 Bacillus anthracis str. Sterne GCA_000008165.1
983 Bacillus cereus ATCC 14579 GCA_000007825.1
5D
735 Lactobacillus gasseri ATCC 33323 = JCM 1131 GCA_000014425.1
702 Lactobacillus hominis DSM 23910 = CRBIP 24.179 GCA_000296835.1
512 Lactobacillus iners DSM 13335 GCA_000160875.1
5E
426 Streptococcus mutans UA159 GCA_000007465.2
320 Streptococcus ratti FA-1 = DSM 20564 GCA_000286075.1

309 Streptococcus sobrinus DSM 20742 = ATCC 33478 GCA_000686605.1
5F
771 Enterococcus faecalis V583 GCA_000007785.1
724 Streptomyces cinnamoneus GCA_001885705.1
661 Enterococcus faecium DO GCA_000174395.2
5G
694 Clostridium beijerinckii GCA_000833105.2
680 Clostridium saccharoperbutylacetonicum N1-4_28HMT_29 GCA_000340885.1
657 Clostridium puniceum GCA_002006345.1
5H
664 Rhodobacter sphaeroides 2.4.1 GCA_000012905.2
618 Rhodobacter sphaeroides ATCC 17025 GCA_000016405.1
469 Pseudorhodobacter ferrugineus DSM 5888 GCA_000420745.1
5I
599 Rhodobacter sphaeroides 2.4.1 GCA_000012905.2
516 Rhodobacter sphaeroides ATCC 17025 GCA_000016405.1
475 Gemmobacter aquatilis GCA_900110025.1
5J
537 Deinococcus radiodurans R1 GCA_000008565.1
384 Deinococcus deserti VCD115 GCA_000020685.1
372 Deinococcus soli Cha et al. 2016 GCA_001007995.1
5K
681 Clostridium beijerinckii GCA_000833105.2
652 Clostridium saccharoperbutylacetonicum N1-4_28HMT_29 GCA_000340885.1
620 Clostridium puniceum GCA_002006345.1
5L
903 Clostridium beijerinckii GCA_000833105.2
873 Clostridium saccharoperbutylacetonicum N1-4_28HMT_29 GCA_000340885.1
838 Clostridium puniceum GCA_002006345.1
5M
763 Bifidobacterium adolescentis ATCC 15703 GCA_000010425.1
634 Bifidobacterium callitrichos DSM 23973 GCA_000741175.1
618 Bifidobacterium asteroides PRL2011 GCA_000304215.1
5N
624 Bifidobacterium adolescentis ATCC 15703 GCA_000010425.1
537 Bifidobacterium dentium JCM 1195 = DSM 20436 GCA_001042595.1
519 Bifidobacterium longum NCC2705 GCA_000007525.1
5O
840 Streptococcus mutans UA159 GCA_000007465.2
697 Streptococcus ratti FA-1 = DSM 20564 GCA_000286075.1
651 Streptococcus sobrinus DSM 20742 = ATCC 33478 GCA_000686605.1
5P
473 Rhodobacter sphaeroides 2.4.1 GCA_000012905.2
420 Rhodobacter sphaeroides ATCC 17025 GCA_000016405.1
379 Gemmobacter aquatilis GCA_900110025.1
5Q
845 Rhodobacter sphaeroides 2.4.1 GCA_000012905.2
764 Rhodobacter sphaeroides ATCC 17025 GCA_000016405.1
635 Gemmobacter aquatilis GCA_900110025.1
5R
587 Enterococcus faecalis V583 GCA_000007785.1
546 Streptomyces cinnamoneus GCA_001885705.1
521 Enterococcus saccharolyticus subsp. saccharolyticus ATCC 43076 GCA_000407285.1
5S
853 Streptococcus mutans UA159 GCA_000007465.2
631 Streptococcus ratti FA-1 = DSM 20564 GCA_000286075.1
600 Streptococcus gordonii str. Challis substr. CH1 GCA_000017005.1
5T
601 Deinococcus radiodurans R1 GCA_000008565.1
431 Deinococcus deserti VCD115 GCA_000020685.1
404 Deinococcus gobiensis I-0 GCA_000252445.1
5U
659 Lactobacillus gasseri ATCC 33323 = JCM 1131 GCA_000014425.1
622 Lactobacillus hominis DSM 23910 = CRBIP 24.179 GCA_000296835.1
474 Lactobacillus psittaci DSM 15354 GCA_000425905.1
5V
806 Bacillus anthracis str. Ames GCA_000007845.1
806 Bacillus anthracis str. Sterne GCA_000008165.1
806 _5BBacillus thuringiensis_5D serovar konkukian str. 97-27 GCA_000008505.1
793 Bacillus cereus ATCC 14579 GCA_000007825.1
793 Bacillus mycoides GCA_000832605.1
781 Bacillus thuringiensis YBT-1518 GCA_000497525.2
5W
746 Deinococcus radiodurans R1 GCA_000008565.1
448 Deinococcus deserti VCD115 GCA_000020685.1
445 Deinococcus hopiensis KR-140 GCA_900176165.1

5X
784 Rhodobacter sphaeroides 2.4.1 GCA_000012905.2
721 Rhodobacter sphaeroides ATCC 17025 GCA_000016405.1
586 Gemmobacter megaterium GCA_900156815.1
5Y
468 Deinococcus radiodurans R1 GCA_000008565.1
348 Deinococcus deserti VCD115 GCA_000020685.1
315 Deinococcus soli Cha et al. 2016 GCA_001007995.1
5Z
772 Lactobacillus gasseri ATCC 33323 = JCM 1131 GCA_000014425.1
732 Lactobacillus hominis DSM 23910 = CRBIP 24.179 GCA_000296835.1
558 Lactobacillus iners DSM 13335 GCA_000160875.1
60
458 Rhodobacter sphaeroides 2.4.1 GCA_000012905.2
398 Rhodobacter sphaeroides ATCC 17025 GCA_000016405.1
338 Defluviimonas alba GCA_001620265.1
61
630 Rhodobacter sphaeroides 2.4.1 GCA_000012905.2
592 Rhodobacter sphaeroides ATCC 17025 GCA_000016405.1
463 Pseudorhodobacter psychrotolerans GCA_001294535.1
62
579 Deinococcus radiodurans R1 GCA_000008565.1
419 Deinococcus deserti VCD115 GCA_000020685.1
407 Deinococcus hopiensis KR-140 GCA_900176165.1
407 Deinococcus marmoris DSM 12784 GCA_000701405.1
63
798 Lactobacillus gasseri ATCC 33323 = JCM 1131 GCA_000014425.1
718 Lactobacillus hominis DSM 23910 = CRBIP 24.179 GCA_000296835.1
530 Lactobacillus iners DSM 13335 GCA_000160875.1
64
396 Deinococcus radiodurans R1 GCA_000008565.1
228 Deinococcus deserti VCD115 GCA_000020685.1
195 Deinococcus marmoris DSM 12784 GCA_000701405.1
65
741 Escherichia coli IAI39 GCA_000026345.1
741 Escherichia coli O157_3AH7 str. Sakai GCA_000008865.1
741 Escherichia coli str. K-12 substr. MG1655 GCA_000005845.2
740 Escherichia coli UMN026 GCA_000026325.2
729 Escherichia coli O83_3AH1 str. NRG 857C GCA_000183345.1
66
721 Deinococcus radiodurans R1 GCA_000008565.1
459 Deinococcus gobiensis I-0 GCA_000252445.1
448 Deinococcus hopiensis KR-140 GCA_900176165.1
67
739 Staphylococcus epidermidis ATCC 12228 GCA_000007645.1
693 Staphylococcus capitis subsp. capitis GCA_001028645.1
628 Staphylococcus haemolyticus JCSC1435 GCA_000009865.1
68
875 Staphylococcus epidermidis ATCC 12228 GCA_000007645.1
828 Staphylococcus capitis subsp. capitis GCA_001028645.1
771 Staphylococcus warneri SG1 GCA_000332735.1
69
730 Deinococcus radiodurans R1 GCA_000008565.1
449 Deinococcus deserti VCD115 GCA_000020685.1
422 Deinococcus gobiensis I-0 GCA_000252445.1
422 Deinococcus soli Cha et al. 2016 GCA_001007995.1
6a
878 Staphylococcus epidermidis ATCC 12228 GCA_000007645.1
805 Staphylococcus capitis subsp. capitis GCA_001028645.1
762 Staphylococcus aureus subsp. aureus NCTC 8325 GCA_000013425.1
6b
679 Bifidobacterium adolescentis ATCC 15703 GCA_000010425.1
567 Bifidobacterium dentium JCM 1195 = DSM 20436 GCA_001042595.1
542 Bifidobacterium angulatum DSM 20098 = JCM 7096 GCA_001025155.1
6c
692 Rhodobacter sphaeroides 2.4.1 GCA_000012905.2
596 Rhodobacter sphaeroides ATCC 17025 GCA_000016405.1
523 Gemmobacter aquatilis GCA_900110025.1
6d
624 Lactobacillus gasseri ATCC 33323 = JCM 1131 GCA_000014425.1
567 Lactobacillus hominis DSM 23910 = CRBIP 24.179 GCA_000296835.1
434 Lactobacillus iners DSM 13335 GCA_000160875.1
6e
537 Rhodobacter sphaeroides 2.4.1 GCA_000012905.2
494 Rhodobacter sphaeroides ATCC 17025 GCA_000016405.1
454 Defluviimonas alba GCA_001620265.1

6f
862 Bifidobacterium adolescentis ATCC 15703 GCA_000010425.1
747 Bifidobacterium dentium JCM 1195 = DSM 20436 GCA_001042595.1
713 Bifidobacterium stellenboschense GCA_000741785.1
6g
586 Bifidobacterium adolescentis ATCC 15703 GCA_000010425.1
508 Bifidobacterium dentium JCM 1195 = DSM 20436 GCA_001042595.1
495 Bifidobacterium angulatum DSM 20098 = JCM 7096 GCA_001025155.1
6h
775 Deinococcus radiodurans R1 GCA_000008565.1
475 Deinococcus deserti VCD115 GCA_000020685.1
423 Deinococcus soli Cha et al. 2016 GCA_001007995.1
6i
801 Streptococcus mutans UA159 GCA_000007465.2
547 Streptococcus ratti FA-1 = DSM 20564 GCA_000286075.1
487 Streptococcus criceti HS-6 GCA_000187975.3
6j
526 Enterococcus faecalis V583 GCA_000007785.1
479 Streptomyces cinnamoneus GCA_001885705.1
472 Enterococcus canis NBRC 100695 GCA_001544375.1
6k
558 Streptococcus mutans UA159 GCA_000007465.2
370 Streptococcus ratti FA-1 = DSM 20564 GCA_000286075.1
348 Streptococcus macacae NCTC 11558 GCA_000187995.3
6l
453 Clostridium beijerinckii GCA_000833105.2
440 Clostridium saccharoperbutylacetonicum N1-4_28HMT_29 GCA_000340885.1
439 Clostridium chromiireducens GCA_002029255.1
6m
755 Rhodobacter sphaeroides 2.4.1 GCA_000012905.2
674 Rhodobacter sphaeroides ATCC 17025 GCA_000016405.1
573 Gemmobacter megaterium GCA_900156815.1
6n
386 Rhodobacter sphaeroides 2.4.1 GCA_000012905.2
321 Rhodobacter sphaeroides ATCC 17025 GCA_000016405.1
239 Rhodobacter capsulatus SB 1003 GCA_000021865.1
6o
646 Staphylococcus epidermidis ATCC 12228 GCA_000007645.1
609 Staphylococcus capitis subsp. capitis GCA_001028645.1
578 Staphylococcus aureus subsp. aureus NCTC 8325 GCA_000013425.1
6p
555 Lactobacillus gasseri ATCC 33323 = JCM 1131 GCA_000014425.1
555 Lactobacillus hominis DSM 23910 = CRBIP 24.179 GCA_000296835.1
436 Lactobacillus iners DSM 13335 GCA_000160875.1
383 Lactobacillus hamsteri DSM 5661 = JCM 6256 GCA_000615445.1
6q
506 Enterococcus faecalis V583 GCA_000007785.1
462 Streptomyces cinnamoneus GCA_001885705.1
404 Enterococcus asini ATCC 700915 GCA_000407365.1
6r
633 Clostridium beijerinckii GCA_000833105.2
604 Clostridium saccharobutylicum DSM 13864 GCA_000473995.1
601 Clostridium saccharoperbutylacetonicum N1-4_28HMT_29 GCA_000340885.1
6s
916 Clostridium beijerinckii GCA_000833105.2
913 Clostridium saccharoperbutylacetonicum N1-4_28HMT_29 GCA_000340885.1
880 Clostridium puniceum GCA_002006345.1
880 Clostridium saccharobutylicum DSM 13864 GCA_000473995.1
6t
326 Staphylococcus epidermidis ATCC 12228 GCA_000007645.1
311 Staphylococcus capitis subsp. capitis GCA_001028645.1
279 Staphylococcus haemolyticus JCSC1435 GCA_000009865.1
279 Staphylococcus lugdunensis HKU09-01 GCA_000025085.1
6u
673 Clostridium beijerinckii GCA_000833105.2
655 Clostridium saccharoperbutylacetonicum N1-4_28HMT_29 GCA_000340885.1
613 Clostridium saccharobutylicum DSM 13864 GCA_000473995.1
6v
523 Clostridium beijerinckii GCA_000833105.2
476 Clostridium saccharoperbutylacetonicum N1-4_28HMT_29 GCA_000340885.1
467 Clostridium puniceum GCA_002006345.1
6w
620 Bifidobacterium adolescentis ATCC 15703 GCA_000010425.1
515 Bifidobacterium angulatum DSM 20098 = JCM 7096 GCA_001025155.1
489 Bifidobacterium dentium JCM 1195 = DSM 20436 GCA_001042595.1
6x

730 Enterococcus faecalis V583 GCA_000007785.1
683 Streptomyces cinnamoneus GCA_001885705.1
639 Enterococcus rivorum GCA_001742285.1
6y
505 Rhodobacter sphaeroides 2.4.1 GCA_000012905.2
421 Rhodobacter sphaeroides ATCC 17025 GCA_000016405.1
418 Gemmobacter aquatilis GCA_900110025.1
6z
776 Staphylococcus epidermidis ATCC 12228 GCA_000007645.1
766 Staphylococcus capitis subsp. capitis GCA_001028645.1
708 Staphylococcus aureus subsp. aureus NCTC 8325 GCA_000013425.1
6A
883 Staphylococcus epidermidis ATCC 12228 GCA_000007645.1
827 Staphylococcus capitis subsp. capitis GCA_001028645.1
781 Staphylococcus haemolyticus JCSC1435 GCA_000009865.1
6B
959 Enterococcus faecalis V583 GCA_000007785.1
912 Streptomyces cinnamoneus GCA_001885705.1
884 Enterococcus rivorum GCA_001742285.1
6C
834 Clostridium beijerinckii GCA_000833105.2
825 Clostridium saccharoperbutylacetonicum N1-4_28HMT_29 GCA_000340885.1
793 Clostridium puniceum GCA_002006345.1
6D
559 Enterococcus faecalis V583 GCA_000007785.1
512 Streptomyces cinnamoneus GCA_001885705.1
431 Enterococcus rivorum GCA_001742285.1
6E
608 Staphylococcus epidermidis ATCC 12228 GCA_000007645.1
573 Staphylococcus capitis subsp. capitis GCA_001028645.1
540 Staphylococcus haemolyticus JCSC1435 GCA_000009865.1
6F
864 Escherichia coli O104_3AH4 str. 2011C-3493 GCA_000299455.1
864 Escherichia coli str. K-12 substr. MG1655 GCA_000005845.2
849 Shigella flexneri 2a str. 301 GCA_000006925.2
810 Escherichia coli O157_3AH7 str. Sakai GCA_000008865.1
6G
881 Bacillus anthracis str. Ames GCA_000007845.1
881 Bacillus anthracis str. Sterne GCA_000008165.1
881 Bacillus pseudomycoides DSM 12442 GCA_000161455.1
881 _5BBacillus thuringiensis_5D serovar konkukian str. 97-27 GCA_000008505.1
873 Bacillus cereus ATCC 14579 GCA_000007825.1
854 Bacillus thuringiensis YBT-1518 GCA_000497525.2
6H
701 Rhodobacter sphaeroides 2.4.1 GCA_000012905.2
607 Rhodobacter sphaeroides ATCC 17025 GCA_000016405.1
459 Gemmobacter aquatilis GCA_900110025.1
6I
802 Clostridium beijerinckii GCA_000833105.2
779 Clostridium saccharoperbutylacetonicum N1-4_28HMT_29 GCA_000340885.1
758 Clostridium puniceum GCA_002006345.1
6J
602 Clostridium beijerinckii GCA_000833105.2
602 Clostridium saccharoperbutylacetonicum N1-4_28HMT_29 GCA_000340885.1
569 Clostridium puniceum GCA_002006345.1
562 Clostridium butyricum GCA_001456065.2
6K
828 Bifidobacterium adolescentis ATCC 15703 GCA_000010425.1
719 Bifidobacterium dentium JCM 1195 = DSM 20436 GCA_001042595.1
625 Bifidobacterium asteroides PRL2011 GCA_000304215.1
6L
761 Streptococcus mutans UA159 GCA_000007465.2
544 Streptococcus gordonii str. Challis substr. CH1 GCA_000017005.1
536 Streptococcus ratti FA-1 = DSM 20564 GCA_000286075.1
6M
879 Enterococcus faecalis V583 GCA_000007785.1
832 Streptomyces cinnamoneus GCA_001885705.1
742 Enterococcus asini ATCC 700915 GCA_000407365.1
6N
747 Lactobacillus gasseri ATCC 33323 = JCM 1131 GCA_000014425.1
694 Lactobacillus hominis DSM 23910 = CRBIP 24.179 GCA_000296835.1
505 Lactobacillus jensenii GCA_001936235.1
505 Lactobacillus psittaci DSM 15354 GCA_000425905.1
6O
714 Clostridium beijerinckii GCA_000833105.2
690 Clostridium saccharobutylicum DSM 13864 GCA_000473995.1

680 Clostridium saccharoperbutylacetonicum N1-4_28HMT_29 GCA_000340885.1
6P
956 Lactobacillus gasseri ATCC 33323 = JCM 1131 GCA_000014425.1
878 Lactobacillus hominis DSM 23910 = CRBIP 24.179 GCA_000296835.1
642 Lactobacillus iners DSM 13335 GCA_000160875.1
6Q
684 Rhodobacter sphaeroides 2.4.1 GCA_000012905.2
640 Rhodobacter sphaeroides ATCC 17025 GCA_000016405.1
597 Gemmobacter aquatilis GCA_900110025.1
6R
599 Lactobacillus gasseri ATCC 33323 = JCM 1131 GCA_000014425.1
556 Lactobacillus hominis DSM 23910 = CRBIP 24.179 GCA_000296835.1
409 Lactobacillus iners DSM 13335 GCA_000160875.1
6S
523 Clostridium beijerinckii GCA_000833105.2
508 Clostridium puniceum GCA_002006345.1
508 Clostridium saccharobutylicum DSM 13864 GCA_000473995.1
508 Clostridium saccharoperbutylacetonicum N1-4_28HMT_29 GCA_000340885.1
484 Clostridium butyricum GCA_001456065.2
6T
849 Rhodobacter sphaeroides 2.4.1 GCA_000012905.2
723 Rhodobacter sphaeroides ATCC 17025 GCA_000016405.1
631 Defluviimonas alba GCA_001620265.1
6U
817 Escherichia coli str. K-12 substr. MG1655 GCA_000005845.2
797 Shigella flexneri 2a str. 301 GCA_000006925.2
790 Escherichia coli UMN026 GCA_000026325.2
6V
808 Bacillus anthracis str. Ames GCA_000007845.1
808 Bacillus anthracis str. Sterne GCA_000008165.1
808 _5BBacillus thuringiensis_5D serovar konkukian str. 97-27 GCA_000008505.1
796 Bacillus cereus ATCC 14579 GCA_000007825.1
783 Bacillus pseudomycoides DSM 12442 GCA_000161455.1
6W
450 Bifidobacterium adolescentis ATCC 15703 GCA_000010425.1
351 Bifidobacterium callitrichos DSM 23973 GCA_000741175.1
346 Bifidobacterium longum NCC2705 GCA_000007525.1
6X
682 Streptococcus mutans UA159 GCA_000007465.2
525 Streptococcus ratti FA-1 = DSM 20564 GCA_000286075.1
497 Streptococcus halotolerans GCA_001598035.1
6Y
568 Enterococcus faecalis V583 GCA_000007785.1
525 Streptomyces cinnamoneus GCA_001885705.1
451 Enterococcus massiliensis GCA_001050095.1
6Z
642 Streptococcus mutans UA159 GCA_000007465.2
456 Streptococcus ratti FA-1 = DSM 20564 GCA_000286075.1
442 Streptococcus gordonii str. Challis substr. CH1 GCA_000017005.1
70
602 Shigella flexneri 2a str. 301 GCA_000006925.2
599 Escherichia coli O104_3AH4 str. 2011C-3493 GCA_000299455.1
599 Escherichia coli O157_3AH7 str. Sakai GCA_000008865.1
599 Escherichia coli str. K-12 substr. MG1655 GCA_000005845.2
590 Escherichia coli O83_3AH1 str. NRG 857C GCA_000183345.1
590 Escherichia coli UMN026 GCA_000026325.2
71
752 Enterococcus faecalis V583 GCA_000007785.1
732 Enterococcus faecium DO GCA_000174395.2
709 Enterococcus canis NBRC 100695 GCA_001544375.1
72
681 Lactobacillus gasseri ATCC 33323 = JCM 1131 GCA_000014425.1
616 Lactobacillus hominis DSM 23910 = CRBIP 24.179 GCA_000296835.1
486 Lactobacillus iners DSM 13335 GCA_000160875.1
73
688 Enterococcus faecalis V583 GCA_000007785.1
647 Streptomyces cinnamoneus GCA_001885705.1
618 Enterococcus faecium DO GCA_000174395.2
74
380 Bifidobacterium adolescentis ATCC 15703 GCA_000010425.1
330 Bifidobacterium dentium JCM 1195 = DSM 20436 GCA_001042595.1
304 Bifidobacterium tsurumiense GCA_000741765.1
75
636 Streptococcus mutans UA159 GCA_000007465.2
469 Streptococcus equinus GCA_000964315.1
467 Streptococcus ratti FA-1 = DSM 20564 GCA_000286075.1

76
673 Staphylococcus epidermidis ATCC 12228 GCA_000007645.1
662 Staphylococcus warneri SG1 GCA_000332735.1
660 Staphylococcus capitis subsp. capitis GCA_001028645.1
77
878 Enterococcus faecalis V583 GCA_000007785.1
832 Streptomyces cinnamoneus GCA_001885705.1
770 Enterococcus canis NBRC 100695 GCA_001544375.1
78
746 Streptococcus mutans UA159 GCA_000007465.2
560 Streptococcus ratti FA-1 = DSM 20564 GCA_000286075.1
501 Streptococcus gordonii str. Challis substr. CH1 GCA_000017005.1
79
816 Clostridium beijerinckii GCA_000833105.2
760 Clostridium saccharoperbutylacetonicum N1-4_28HMT_29 GCA_000340885.1
739 Clostridium butyricum GCA_001456065.2
7a
603 Rhodobacter sphaeroides 2.4.1 GCA_000012905.2
566 Rhodobacter sphaeroides ATCC 17025 GCA_000016405.1
474 Gemmobacter aquatilis GCA_900110025.1
7b
783 Staphylococcus epidermidis ATCC 12228 GCA_000007645.1
748 Staphylococcus haemolyticus JCSC1435 GCA_000009865.1
731 Staphylococcus capitis subsp. capitis GCA_001028645.1
7c
689 Deinococcus radiodurans R1 GCA_000008565.1
383 Deinococcus deserti VCD115 GCA_000020685.1
362 Deinococcus gobiensis I-0 GCA_000252445.1
7d
577 Streptococcus mutans UA159 GCA_000007465.2
450 Streptococcus ratti FA-1 = DSM 20564 GCA_000286075.1
411 Streptococcus ferus DSM 20646 GCA_000372425.1
7e
759 Staphylococcus epidermidis ATCC 12228 GCA_000007645.1
728 Staphylococcus capitis subsp. capitis GCA_001028645.1
667 Staphylococcus warneri SG1 GCA_000332735.1
7f
731 Lactobacillus gasseri ATCC 33323 = JCM 1131 GCA_000014425.1
652 Lactobacillus hominis DSM 23910 = CRBIP 24.179 GCA_000296835.1
500 Lactobacillus jensenii GCA_001936235.1
7g
675 Deinococcus radiodurans R1 GCA_000008565.1
458 Deinococcus gobiensis I-0 GCA_000252445.1
420 Deinococcus deserti VCD115 GCA_000020685.1
7h
716 Deinococcus radiodurans R1 GCA_000008565.1
491 Deinococcus deserti VCD115 GCA_000020685.1
475 Deinococcus hopiensis KR-140 GCA_900176165.1
7i
422 Rhodobacter sphaeroides 2.4.1 GCA_000012905.2
392 Rhodobacter sphaeroides ATCC 17025 GCA_000016405.1
287 Pseudorhodobacter wandonensis GCA_001202035.1
7j
805 Lactobacillus gasseri ATCC 33323 = JCM 1131 GCA_000014425.1
743 Lactobacillus hominis DSM 23910 = CRBIP 24.179 GCA_000296835.1
543 Lactobacillus iners DSM 13335 GCA_000160875.1
7k
739 Bacillus cereus ATCC 14579 GCA_000007825.1
724 Bacillus anthracis str. Ames GCA_000007845.1
724 Bacillus anthracis str. Sterne GCA_000008165.1
724 _5BBacillus thuringiensis_5D serovar konkukian str. 97-27 GCA_000008505.1
719 Bacillus thuringiensis YBT-1518 GCA_000497525.2
7l
618 Enterococcus faecalis V583 GCA_000007785.1
586 Streptomyces cinnamoneus GCA_001885705.1
518 Enterococcus rivorum GCA_001742285.1
7m
763 Rhodobacter sphaeroides 2.4.1 GCA_000012905.2
681 Rhodobacter sphaeroides ATCC 17025 GCA_000016405.1
552 Pseudorhodobacter psychrotolerans GCA_001294535.1
7n
783 Rhodobacter sphaeroides 2.4.1 GCA_000012905.2
733 Rhodobacter sphaeroides ATCC 17025 GCA_000016405.1
588 Pseudorhodobacter ferrugineus DSM 5888 GCA_000420745.1
7o
541 Enterococcus faecalis V583 GCA_000007785.1

494 Streptomyces cinnamoneus GCA_001885705.1
454 Enterococcus canis NBRC 100695 GCA_001544375.1
7p
460 Streptococcus mutans UA159 GCA_000007465.2
340 Streptococcus equinus GCA_000964315.1
338 Streptococcus sobrinus DSM 20742 = ATCC 33478 GCA_000686605.1
7q
843 Staphylococcus epidermidis ATCC 12228 GCA_000007645.1
789 Staphylococcus capitis subsp. capitis GCA_001028645.1
708 Staphylococcus hominis subsp. hominis C80 GCA_000183685.1
7r
939 Lactobacillus gasseri ATCC 33323 = JCM 1131 GCA_000014425.1
823 Lactobacillus hominis DSM 23910 = CRBIP 24.179 GCA_000296835.1
615 Lactobacillus iners DSM 13335 GCA_000160875.1
7s
657 Escherichia coli str. K-12 substr. MG1655 GCA_000005845.2
654 Escherichia coli O157_3AH7 str. Sakai GCA_000008865.1
654 Shigella flexneri 2a str. 301 GCA_000006925.2
646 Escherichia coli UMN026 GCA_000026325.2
7t
878 Staphylococcus epidermidis ATCC 12228 GCA_000007645.1
835 Staphylococcus capitis subsp. capitis GCA_001028645.1
743 Staphylococcus hominis subsp. hominis C80 GCA_000183685.1
743 Staphylococcus warneri SG1 GCA_000332735.1
7u
798 Clostridium beijerinckii GCA_000833105.2
770 Clostridium saccharoperbutylacetonicum N1-4_28HMT_29 GCA_000340885.1
749 Clostridium puniceum GCA_002006345.1
7v
641 Lactobacillus gasseri ATCC 33323 = JCM 1131 GCA_000014425.1
612 Lactobacillus hominis DSM 23910 = CRBIP 24.179 GCA_000296835.1
454 Lactobacillus psittaci DSM 15354 GCA_000425905.1
7w
837 Bacillus anthracis str. Ames GCA_000007845.1
837 Bacillus anthracis str. Sterne GCA_000008165.1
837 _5BBacillus thuringiensis_5D serovar konkukian str. 97-27 GCA_000008505.1
822 Bacillus cereus ATCC 14579 GCA_000007825.1
802 Bacillus thuringiensis YBT-1518 GCA_000497525.2
7x
638 Deinococcus radiodurans R1 GCA_000008565.1
430 Deinococcus deserti VCD115 GCA_000020685.1
430 Deinococcus hopiensis KR-140 GCA_900176165.1
402 Deinococcus murrayi DSM 11303 GCA_000482805.1
7y
651 Deinococcus radiodurans R1 GCA_000008565.1
406 Deinococcus deserti VCD115 GCA_000020685.1
390 Deinococcus puniceus GCA_001644565.1
7z
857 Lactobacillus gasseri ATCC 33323 = JCM 1131 GCA_000014425.1
763 Lactobacillus hominis DSM 23910 = CRBIP 24.179 GCA_000296835.1
593 Lactobacillus iners DSM 13335 GCA_000160875.1
7A
519 Escherichia coli O104_3AH4 str. 2011C-3493 GCA_000299455.1
514 Escherichia coli str. K-12 substr. MG1655 GCA_000005845.2
514 Shigella flexneri 2a str. 301 GCA_000006925.2
488 Escherichia coli O157_3AH7 str. Sakai GCA_000008865.1
7B
668 Rhodobacter sphaeroides 2.4.1 GCA_000012905.2
637 Rhodobacter sphaeroides ATCC 17025 GCA_000016405.1
562 Pseudorhodobacter psychrotolerans GCA_001294535.1
7C
609 Staphylococcus epidermidis ATCC 12228 GCA_000007645.1
565 Staphylococcus capitis subsp. capitis GCA_001028645.1
513 Staphylococcus warneri SG1 GCA_000332735.1
7D
844 Deinococcus radiodurans R1 GCA_000008565.1
591 Deinococcus deserti VCD115 GCA_000020685.1
560 Deinococcus proteolyticus MRP GCA_000190555.1
7E
836 Staphylococcus epidermidis ATCC 12228 GCA_000007645.1
791 Staphylococcus capitis subsp. capitis GCA_001028645.1
736 Megasphaera cerevisiae DSM 20462 GCA_001045675.1
736 Staphylococcus warneri SG1 GCA_000332735.1
7F
539 Bifidobacterium adolescentis ATCC 15703 GCA_000010425.1
432 Bifidobacterium dentium JCM 1195 = DSM 20436 GCA_001042595.1

360 Bifidobacterium asteroides PRL2011 GCA_000304215.1
7G
713 Escherichia coli str. K-12 substr. MG1655 GCA_000005845.2
704 Escherichia coli O104_3AH4 str. 2011C-3493 GCA_000299455.1
688 Shigella flexneri 2a str. 301 GCA_000006925.2
7H
707 Deinococcus radiodurans R1 GCA_000008565.1
456 Deinococcus deserti VCD115 GCA_000020685.1
440 Deinococcus marmoris DSM 12784 GCA_000701405.1
7I
678 Clostridium beijerinckii GCA_000833105.2
642 Clostridium saccharoperbutylacetonicum N1-4_28HMT_29 GCA_000340885.1
616 Clostridium saccharobutylicum DSM 13864 GCA_000473995.1
7J
480 Bifidobacterium adolescentis ATCC 15703 GCA_000010425.1
403 Bifidobacterium dentium JCM 1195 = DSM 20436 GCA_001042595.1
388 Bifidobacterium breve DSM 20213 = JCM 1192 GCA_001025175.1
7K
721 Streptococcus mutans UA159 GCA_000007465.2
490 Streptococcus ratti FA-1 = DSM 20564 GCA_000286075.1
481 Streptococcus criceti HS-6 GCA_000187975.3
7L
657 Lactobacillus gasseri ATCC 33323 = JCM 1131 GCA_000014425.1
609 Lactobacillus hominis DSM 23910 = CRBIP 24.179 GCA_000296835.1
494 Lactobacillus psittaci DSM 15354 GCA_000425905.1
7M
685 Staphylococcus epidermidis ATCC 12228 GCA_000007645.1
673 Staphylococcus capitis subsp. capitis GCA_001028645.1
602 Staphylococcus warneri SG1 GCA_000332735.1
7N
618 Escherichia coli IAI39 GCA_000026345.1
618 Escherichia coli O157_3AH7 str. Sakai GCA_000008865.1
618 Escherichia coli str. K-12 substr. MG1655 GCA_000005845.2
609 Escherichia coli UMN026 GCA_000026325.2
606 Escherichia coli O83_3AH1 str. NRG 857C GCA_000183345.1
7O
738 Staphylococcus epidermidis ATCC 12228 GCA_000007645.1
707 Staphylococcus capitis subsp. capitis GCA_001028645.1
664 Staphylococcus hominis subsp. hominis C80 GCA_000183685.1
7P
876 Streptococcus mutans UA159 GCA_000007465.2
612 Streptococcus gordonii str. Challis substr. CH1 GCA_000017005.1
606 Streptococcus ratti FA-1 = DSM 20564 GCA_000286075.1
7Q
344 Rhodobacter sphaeroides 2.4.1 GCA_000012905.2
292 Rhodobacter sphaeroides ATCC 17025 GCA_000016405.1
272 Defluviimonas alba GCA_001620265.1
7R
483 Staphylococcus epidermidis ATCC 12228 GCA_000007645.1
440 Staphylococcus capitis subsp. capitis GCA_001028645.1
401 Megasphaera cerevisiae DSM 20462 GCA_001045675.1
401 Staphylococcus warneri SG1 GCA_000332735.1
7S
705 Deinococcus radiodurans R1 GCA_000008565.1
395 Deinococcus deserti VCD115 GCA_000020685.1
368 Deinococcus proteolyticus MRP GCA_000190555.1
7T
629 Bacillus thuringiensis YBT-1518 GCA_000497525.2
628 Bacillus anthracis str. Ames GCA_000007845.1
628 Bacillus anthracis str. Sterne GCA_000008165.1
628 Bacillus cereus ATCC 14579 GCA_000007825.1
628 Bacillus pseudomycoides DSM 12442 GCA_000161455.1
628 _5BBacillus thuringiensis_5D serovar konkukian str. 97-27 GCA_000008505.1
618 Bacillus mycoides GCA_000832605.1
7U
824 Staphylococcus epidermidis ATCC 12228 GCA_000007645.1
798 Staphylococcus capitis subsp. capitis GCA_001028645.1
724 Staphylococcus hominis subsp. hominis C80 GCA_000183685.1
7V
756 Staphylococcus epidermidis ATCC 12228 GCA_000007645.1
729 Staphylococcus capitis subsp. capitis GCA_001028645.1
696 Staphylococcus aureus subsp. aureus NCTC 8325 GCA_000013425.1
7W
581 Bifidobacterium adolescentis ATCC 15703 GCA_000010425.1
454 Bifidobacterium dentium JCM 1195 = DSM 20436 GCA_001042595.1
453 Bifidobacterium longum NCC2705 GCA_000007525.1

7X
593 Lactobacillus gasseri ATCC 33323 = JCM 1131 GCA_000014425.1
523 Lactobacillus hominis DSM 23910 = CRBIP 24.179 GCA_000296835.1
379 Lactobacillus jensenii GCA_001936235.1
379 Lactobacillus psittaci DSM 15354 GCA_000425905.1
7Y
782 Escherichia coli IAI39 GCA_000026345.1
782 Escherichia coli O157_3AH7 str. Sakai GCA_000008865.1
782 Escherichia coli str. K-12 substr. MG1655 GCA_000005845.2
771 Escherichia coli O83_3AH1 str. NRG 857C GCA_000183345.1
759 Shigella flexneri 2a str. 301 GCA_000006925.2
7Z
902 Streptococcus mutans UA159 GCA_000007465.2
657 Streptococcus ratti FA-1 = DSM 20564 GCA_000286075.1
649 Streptococcus gordonii str. Challis substr. CH1 GCA_000017005.1
80
777 Clostridium beijerinckii GCA_000833105.2
752 Clostridium saccharoperbutylacetonicum N1-4_28HMT_29 GCA_000340885.1
721 Clostridium saccharobutylicum DSM 13864 GCA_000473995.1
81
501 Staphylococcus epidermidis ATCC 12228 GCA_000007645.1
451 Staphylococcus capitis subsp. capitis GCA_001028645.1
440 Staphylococcus aureus subsp. aureus NCTC 8325 GCA_000013425.1
82
949 Rhodobacter sphaeroides 2.4.1 GCA_000012905.2
866 Rhodobacter sphaeroides ATCC 17025 GCA_000016405.1
711 Pseudorhodobacter psychrotolerans GCA_001294535.1
83
811 Deinococcus radiodurans R1 GCA_000008565.1
494 Deinococcus deserti VCD115 GCA_000020685.1
492 Deinococcus gobiensis I-0 GCA_000252445.1
84
524 Clostridium beijerinckii GCA_000833105.2
495 Clostridium saccharobutylicum DSM 13864 GCA_000473995.1
485 Clostridium puniceum GCA_002006345.1
485 Clostridium saccharoperbutylacetonicum N1-4_28HMT_29 GCA_000340885.1
85
660 Bifidobacterium adolescentis ATCC 15703 GCA_000010425.1
522 Bifidobacterium dentium JCM 1195 = DSM 20436 GCA_001042595.1
503 Bifidobacterium angulatum DSM 20098 = JCM 7096 GCA_001025155.1
86
736 Rhodobacter sphaeroides 2.4.1 GCA_000012905.2
662 Rhodobacter sphaeroides ATCC 17025 GCA_000016405.1
552 Gemmobacter aquatilis GCA_900110025.1
87
775 Deinococcus radiodurans R1 GCA_000008565.1
461 Deinococcus proteolyticus MRP GCA_000190555.1
460 Deinococcus deserti VCD115 GCA_000020685.1
88
803 Streptococcus mutans UA159 GCA_000007465.2
565 Streptococcus ratti FA-1 = DSM 20564 GCA_000286075.1
561 Streptococcus gordonii str. Challis substr. CH1 GCA_000017005.1
89
635 Deinococcus radiodurans R1 GCA_000008565.1
448 Deinococcus deserti VCD115 GCA_000020685.1
408 Deinococcus gobiensis I-0 GCA_000252445.1
8a
419 Bifidobacterium adolescentis ATCC 15703 GCA_000010425.1
348 Bifidobacterium angulatum DSM 20098 = JCM 7096 GCA_001025155.1
326 Bifidobacterium dentium JCM 1195 = DSM 20436 GCA_001042595.1
8b
448 Clostridium beijerinckii GCA_000833105.2
427 Clostridium saccharoperbutylacetonicum N1-4_28HMT_29 GCA_000340885.1
415 Clostridium saccharobutylicum DSM 13864 GCA_000473995.1
8c
696 Clostridium beijerinckii GCA_000833105.2
680 Clostridium saccharoperbutylacetonicum N1-4_28HMT_29 GCA_000340885.1
651 Clostridium saccharobutylicum DSM 13864 GCA_000473995.1
8d
690 Rhodobacter sphaeroides 2.4.1 GCA_000012905.2
589 Rhodobacter sphaeroides ATCC 17025 GCA_000016405.1
482 Gemmobacter aquatilis GCA_900110025.1
8e
525 Enterococcus faecalis V583 GCA_000007785.1
492 Enterococcus thailandicus GCA_001652875.1
491 Enterococcus canis NBRC 100695 GCA_001544375.1

8f
591 Streptococcus mutans UA159 GCA_000007465.2
417 Streptococcus ratti FA-1 = DSM 20564 GCA_000286075.1
398 Streptococcus sobrinus DSM 20742 = ATCC 33478 GCA_000686605.1
8g
439 Enterococcus faecalis V583 GCA_000007785.1
395 Streptomyces cinnamoneus GCA_001885705.1
390 Enterococcus faecium DO GCA_000174395.2
8h
773 Lactobacillus gasseri ATCC 33323 = JCM 1131 GCA_000014425.1
692 Lactobacillus hominis DSM 23910 = CRBIP 24.179 GCA_000296835.1
577 Lactobacillus iners DSM 13335 GCA_000160875.1
8i
697 Deinococcus radiodurans R1 GCA_000008565.1
425 Deinococcus deserti VCD115 GCA_000020685.1
406 Deinococcus gobiensis I-0 GCA_000252445.1
406 Deinococcus murrayi DSM 11303 GCA_000482805.1
8j
332 Rhodobacter sphaeroides 2.4.1 GCA_000012905.2
253 Rhodobacter sphaeroides ATCC 17025 GCA_000016405.1
250 Pseudorhodobacter psychrotolerans GCA_001294535.1
8k
607 Staphylococcus epidermidis ATCC 12228 GCA_000007645.1
521 Staphylococcus capitis subsp. capitis GCA_001028645.1
509 Staphylococcus haemolyticus JCSC1435 GCA_000009865.1
509 Staphylococcus hominis subsp. hominis C80 GCA_000183685.1
8l
611 Deinococcus radiodurans R1 GCA_000008565.1
379 Deinococcus deserti VCD115 GCA_000020685.1
365 Deinococcus hopiensis KR-140 GCA_900176165.1
8m
769 Escherichia coli O104_3AH4 str. 2011C-3493 GCA_000299455.1
768 Escherichia coli str. K-12 substr. MG1655 GCA_000005845.2
768 Shigella flexneri 2a str. 301 GCA_000006925.2
765 Escherichia coli O157_3AH7 str. Sakai GCA_000008865.1
8n
466 Clostridium saccharobutylicum DSM 13864 GCA_000473995.1
462 Clostridium beijerinckii GCA_000833105.2
451 Clostridium puniceum GCA_002006345.1
451 Clostridium saccharoperbutylacetonicum N1-4_28HMT_29 GCA_000340885.1
8o
1002 Streptococcus mutans UA159 GCA_000007465.2
777 Streptococcus ratti FA-1 = DSM 20564 GCA_000286075.1
756 Streptococcus gordonii str. Challis substr. CH1 GCA_000017005.1
8p
770 Lactobacillus gasseri ATCC 33323 = JCM 1131 GCA_000014425.1
713 Lactobacillus hominis DSM 23910 = CRBIP 24.179 GCA_000296835.1
543 Lactobacillus iners DSM 13335 GCA_000160875.1
8q
864 Enterococcus faecalis V583 GCA_000007785.1
817 Streptomyces cinnamoneus GCA_001885705.1
744 Enterococcus rivorum GCA_001742285.1
8r
615 Enterococcus faecalis V583 GCA_000007785.1
568 Streptomyces cinnamoneus GCA_001885705.1
509 Enterococcus rivorum GCA_001742285.1
8s
531 Lactobacillus gasseri ATCC 33323 = JCM 1131 GCA_000014425.1
476 Lactobacillus hominis DSM 23910 = CRBIP 24.179 GCA_000296835.1
374 Lactobacillus amylolyticus GCA_002075105.1
374 Lactobacillus hamsteri DSM 5661 = JCM 6256 GCA_000615445.1
8t
466 Bacillus anthracis str. Ames GCA_000007845.1
466 Bacillus anthracis str. Sterne GCA_000008165.1
466 Bacillus cereus ATCC 14579 GCA_000007825.1
466 Bacillus mycoides GCA_000832605.1
466 Bacillus pseudomycoides DSM 12442 GCA_000161455.1
466 _5BBacillus thuringiensis_5D serovar konkukian str. 97-27 GCA_000008505.1
451 Bacillus thuringiensis YBT-1518 GCA_000497525.2
391 Bacillus cytotoxicus NVH 391-98 GCA_000017425.1
8u
818 Bacillus anthracis str. Ames GCA_000007845.1
818 Bacillus anthracis str. Sterne GCA_000008165.1
818 Bacillus cereus ATCC 14579 GCA_000007825.1
818 Bacillus thuringiensis YBT-1518 GCA_000497525.2
818 _5BBacillus thuringiensis_5D serovar konkukian str. 97-27 GCA_000008505.1

803 Bacillus pseudomycoides DSM 12442 GCA_000161455.1
778 Bacillus mycoides GCA_000832605.1
8v
350 Enterococcus faecalis V583 GCA_000007785.1
312 Enterococcus rivorum GCA_001742285.1
303 Streptomyces cinnamoneus GCA_001885705.1
8w
848 Bacillus cereus ATCC 14579 GCA_000007825.1
846 Bacillus anthracis str. Ames GCA_000007845.1
846 Bacillus anthracis str. Sterne GCA_000008165.1
846 _5BBacillus thuringiensis_5D serovar konkukian str. 97-27 GCA_000008505.1
811 Bacillus mycoides GCA_000832605.1
811 Bacillus thuringiensis YBT-1518 GCA_000497525.2
8x
648 Bifidobacterium adolescentis ATCC 15703 GCA_000010425.1
574 Bifidobacterium angulatum DSM 20098 = JCM 7096 GCA_001025155.1
543 Bifidobacterium dentium JCM 1195 = DSM 20436 GCA_001042595.1
8y
620 Clostridium beijerinckii GCA_000833105.2
577 Clostridium puniceum GCA_002006345.1
577 Clostridium saccharoperbutylacetonicum N1-4_28HMT_29 GCA_000340885.1
565 Clostridium butyricum GCA_001456065.2
8z
819 Rhodobacter sphaeroides 2.4.1 GCA_000012905.2
731 Rhodobacter sphaeroides ATCC 17025 GCA_000016405.1
607 Pseudorhodobacter psychrotolerans GCA_001294535.1
8A
749 Deinococcus radiodurans R1 GCA_000008565.1
466 Deinococcus deserti VCD115 GCA_000020685.1
444 Deinococcus soli Cha et al. 2016 GCA_001007995.1
8B
731 Clostridium beijerinckii GCA_000833105.2
690 Clostridium puniceum GCA_002006345.1
690 Clostridium saccharoperbutylacetonicum N1-4_28HMT_29 GCA_000340885.1
682 Clostridium saccharobutylicum DSM 13864 GCA_000473995.1
8C
861 Deinococcus radiodurans R1 GCA_000008565.1
592 Deinococcus gobiensis I-0 GCA_000252445.1
560 Deinococcus deserti VCD115 GCA_000020685.1
8D
737 Clostridium beijerinckii GCA_000833105.2
676 Clostridium saccharoperbutylacetonicum N1-4_28HMT_29 GCA_000340885.1
661 Clostridium puniceum GCA_002006345.1
8E
928 Enterococcus faecalis V583 GCA_000007785.1
881 Streptomyces cinnamoneus GCA_001885705.1
795 Enterococcus rivorum GCA_001742285.1
8F
714 Lactobacillus gasseri ATCC 33323 = JCM 1131 GCA_000014425.1
662 Lactobacillus hominis DSM 23910 = CRBIP 24.179 GCA_000296835.1
464 Lactobacillus jensenii GCA_001936235.1
464 Lactobacillus psittaci DSM 15354 GCA_000425905.1
8G
887 Staphylococcus epidermidis ATCC 12228 GCA_000007645.1
823 Staphylococcus capitis subsp. capitis GCA_001028645.1
720 Staphylococcus warneri SG1 GCA_000332735.1
8H
510 Bifidobacterium adolescentis ATCC 15703 GCA_000010425.1
405 Bifidobacterium dentium JCM 1195 = DSM 20436 GCA_001042595.1
403 Bifidobacterium longum NCC2705 GCA_000007525.1
8I
526 Rhodobacter sphaeroides 2.4.1 GCA_000012905.2
439 Rhodobacter sphaeroides ATCC 17025 GCA_000016405.1
372 Gemmobacter megaterium GCA_900156815.1
8J
683 Clostridium beijerinckii GCA_000833105.2
655 Clostridium saccharoperbutylacetonicum N1-4_28HMT_29 GCA_000340885.1
642 Clostridium saccharobutylicum DSM 13864 GCA_000473995.1
8K
579 Clostridium beijerinckii GCA_000833105.2
529 Clostridium saccharobutylicum DSM 13864 GCA_000473995.1
524 Clostridium butyricum GCA_001456065.2
524 Clostridium saccharoperbutylacetonicum N1-4_28HMT_29 GCA_000340885.1
8L
715 Staphylococcus epidermidis ATCC 12228 GCA_000007645.1
678 Staphylococcus capitis subsp. capitis GCA_001028645.1

583 Staphylococcus haemolyticus JCSC1435 GCA_000009865.1
8M
737 Streptococcus mutans UA159 GCA_000007465.2
553 Streptococcus ratti FA-1 = DSM 20564 GCA_000286075.1
486 Streptococcus halotolerans GCA_001598035.1
8N
433 Staphylococcus epidermidis ATCC 12228 GCA_000007645.1
415 Staphylococcus capitis subsp. capitis GCA_001028645.1
372 Staphylococcus hominis subsp. hominis C80 GCA_000183685.1
8O
568 Enterococcus faecalis V583 GCA_000007785.1
524 Streptomyces cinnamoneus GCA_001885705.1
480 Enterococcus rivorum GCA_001742285.1
8P
724 Clostridium beijerinckii GCA_000833105.2
680 Clostridium saccharoperbutylacetonicum N1-4_28HMT_29 GCA_000340885.1
674 Clostridium butyricum GCA_001456065.2
8Q
810 Rhodobacter sphaeroides 2.4.1 GCA_000012905.2
726 Rhodobacter sphaeroides ATCC 17025 GCA_000016405.1
600 Gemmobacter aquatilis GCA_900110025.1
8R
681 Rhodobacter sphaeroides 2.4.1 GCA_000012905.2
595 Rhodobacter sphaeroides ATCC 17025 GCA_000016405.1
512 Gemmobacter aquatilis GCA_900110025.1
8S
797 Streptococcus mutans UA159 GCA_000007465.2
593 Streptococcus ratti FA-1 = DSM 20564 GCA_000286075.1
542 Streptococcus gordonii str. Challis substr. CH1 GCA_000017005.1
8T
670 Lactobacillus gasseri ATCC 33323 = JCM 1131 GCA_000014425.1
599 Lactobacillus hominis DSM 23910 = CRBIP 24.179 GCA_000296835.1
473 Lactobacillus hamsteri DSM 5661 = JCM 6256 GCA_000615445.1
8U
724 Deinococcus radiodurans R1 GCA_000008565.1
373 Deinococcus deserti VCD115 GCA_000020685.1
373 Deinococcus proteolyticus MRP GCA_000190555.1
361 Deinococcus murrayi DSM 11303 GCA_000482805.1
8V
683 Rhodobacter sphaeroides 2.4.1 GCA_000012905.2
645 Rhodobacter sphaeroides ATCC 17025 GCA_000016405.1
527 Defluviimonas alba GCA_001620265.1
527 Pseudorhodobacter psychrotolerans GCA_001294535.1
8W
886 Rhodobacter sphaeroides 2.4.1 GCA_000012905.2
846 Rhodobacter sphaeroides ATCC 17025 GCA_000016405.1
688 Gemmobacter aquatilis GCA_900110025.1
8X
731 Lactobacillus gasseri ATCC 33323 = JCM 1131 GCA_000014425.1
676 Lactobacillus hominis DSM 23910 = CRBIP 24.179 GCA_000296835.1
496 Lactobacillus iners DSM 13335 GCA_000160875.1
8Y
789 Deinococcus radiodurans R1 GCA_000008565.1
474 Deinococcus deserti VCD115 GCA_000020685.1
425 Deinococcus gobiensis I-0 GCA_000252445.1
8Z
654 Rhodobacter sphaeroides 2.4.1 GCA_000012905.2
574 Rhodobacter sphaeroides ATCC 17025 GCA_000016405.1
503 Gemmobacter megaterium GCA_900156815.1
90
375 Lactobacillus gasseri ATCC 33323 = JCM 1131 GCA_000014425.1
375 Lactobacillus hominis DSM 23910 = CRBIP 24.179 GCA_000296835.1
313 Lactobacillus jensenii GCA_001936235.1
313 Lactobacillus psittaci DSM 15354 GCA_000425905.1
296 Lactobacillus mellis GCA_000967245.1
91
391 Deinococcus radiodurans R1 GCA_000008565.1
222 Deinococcus gobiensis I-0 GCA_000252445.1
202 Deinococcus maricopensis DSM 21211 GCA_000186385.1
92
750 Streptococcus mutans UA159 GCA_000007465.2
478 Streptococcus gordonii str. Challis substr. CH1 GCA_000017005.1
478 Streptococcus ratti FA-1 = DSM 20564 GCA_000286075.1
467 Streptococcus gallolyticus subsp. gallolyticus DSM 16831 GCA_002000985.1
93
682 Streptococcus mutans UA159 GCA_000007465.2

494 Streptococcus ratti FA-1 = DSM 20564 GCA_000286075.1
473 Streptococcus gordonii str. Challis substr. CH1 GCA_000017005.1
94
777 Bifidobacterium adolescentis ATCC 15703 GCA_000010425.1
683 Bifidobacterium dentium JCM 1195 = DSM 20436 GCA_001042595.1
641 Bifidobacterium thermophilum GCA_000741495.1
95
642 Staphylococcus epidermidis ATCC 12228 GCA_000007645.1
582 Staphylococcus capitis subsp. capitis GCA_001028645.1
553 Staphylococcus hominis subsp. hominis C80 GCA_000183685.1
96
350 Deinococcus radiodurans R1 GCA_000008565.1
155 Deinococcus deserti VCD115 GCA_000020685.1
155 Deinococcus proteolyticus MRP GCA_000190555.1
141 Deinococcus frigens DSM 12807 GCA_000701425.1
141 Deinococcus puniceus GCA_001644565.1
97
477 Escherichia coli O104_3AH4 str. 2011C-3493 GCA_000299455.1
477 Escherichia coli str. K-12 substr. MG1655 GCA_000005845.2
471 Escherichia coli IAI39 GCA_000026345.1
471 Shigella flexneri 2a str. 301 GCA_000006925.2
464 Escherichia coli O157_3AH7 str. Sakai GCA_000008865.1
464 Shigella dysenteriae Sd197 GCA_000012005.1
98
419 Clostridium beijerinckii GCA_000833105.2
390 Clostridium saccharoperbutylacetonicum N1-4_28HMT_29 GCA_000340885.1
384 Clostridium puniceum GCA_002006345.1
99
886 Clostridium beijerinckii GCA_000833105.2
847 Clostridium saccharoperbutylacetonicum N1-4_28HMT_29 GCA_000340885.1
788 Clostridium saccharobutylicum DSM 13864 GCA_000473995.1
9a
641 Enterococcus faecalis V583 GCA_000007785.1
594 Streptomyces cinnamoneus GCA_001885705.1
552 Enterococcus asini ATCC 700915 GCA_000407365.1
9b
628 Enterococcus faecalis V583 GCA_000007785.1
581 Streptomyces cinnamoneus GCA_001885705.1
555 Enterococcus asini ATCC 700915 GCA_000407365.1
9c
639 Streptococcus mutans UA159 GCA_000007465.2
462 Streptococcus ratti FA-1 = DSM 20564 GCA_000286075.1
441 Streptococcus halotolerans GCA_001598035.1
9d
720 Staphylococcus epidermidis ATCC 12228 GCA_000007645.1
686 Staphylococcus capitis subsp. capitis GCA_001028645.1
640 Staphylococcus haemolyticus JCSC1435 GCA_000009865.1
9e
829 Rhodobacter sphaeroides 2.4.1 GCA_000012905.2
695 Rhodobacter sphaeroides ATCC 17025 GCA_000016405.1
562 Defluviimonas alba GCA_001620265.1
9f
645 Lactobacillus gasseri ATCC 33323 = JCM 1131 GCA_000014425.1
601 Lactobacillus hominis DSM 23910 = CRBIP 24.179 GCA_000296835.1
449 Lactobacillus iners DSM 13335 GCA_000160875.1
9g
674 Enterococcus faecalis V583 GCA_000007785.1
627 Streptomyces cinnamoneus GCA_001885705.1
576 Enterococcus faecium DO GCA_000174395.2
9h
726 Streptococcus mutans UA159 GCA_000007465.2
523 Streptococcus ratti FA-1 = DSM 20564 GCA_000286075.1
494 Streptococcus salivarius GCA_000785515.1
9i
893 Lactobacillus gasseri ATCC 33323 = JCM 1131 GCA_000014425.1
781 Lactobacillus hominis DSM 23910 = CRBIP 24.179 GCA_000296835.1
613 Lactobacillus psittaci DSM 15354 GCA_000425905.1
9j
799 Clostridium beijerinckii GCA_000833105.2
778 Clostridium saccharoperbutylacetonicum N1-4_28HMT_29 GCA_000340885.1
738 Clostridium puniceum GCA_002006345.1
9k
710 Bacillus anthracis str. Ames GCA_000007845.1
710 Bacillus anthracis str. Sterne GCA_000008165.1
710 _5BBacillus thuringiensis_5D serovar konkukian str. 97-27 GCA_000008505.1
697 Bacillus cereus ATCC 14579 GCA_000007825.1

689 Bacillus thuringiensis YBT-1518 GCA_000497525.2
9l
790 Staphylococcus epidermidis ATCC 12228 GCA_000007645.1
763 Staphylococcus capitis subsp. capitis GCA_001028645.1
678 Staphylococcus hominis subsp. hominis C80 GCA_000183685.1
9m
752 Rhodobacter sphaeroides 2.4.1 GCA_000012905.2
681 Rhodobacter sphaeroides ATCC 17025 GCA_000016405.1
523 Pseudorhodobacter psychrotolerans GCA_001294535.1
9n
708 Rhodobacter sphaeroides 2.4.1 GCA_000012905.2
631 Rhodobacter sphaeroides ATCC 17025 GCA_000016405.1
461 Gemmobacter aquatilis GCA_900110025.1
9o
666 Staphylococcus epidermidis ATCC 12228 GCA_000007645.1
626 Staphylococcus capitis subsp. capitis GCA_001028645.1
590 Staphylococcus haemolyticus JCSC1435 GCA_000009865.1
590 Staphylococcus warneri SG1 GCA_000332735.1
9p
754 Clostridium beijerinckii GCA_000833105.2
745 Clostridium saccharoperbutylacetonicum N1-4_28HMT_29 GCA_000340885.1
728 Clostridium saccharobutylicum DSM 13864 GCA_000473995.1
9q
475 Clostridium beijerinckii GCA_000833105.2
468 Clostridium puniceum GCA_002006345.1
468 Clostridium saccharoperbutylacetonicum N1-4_28HMT_29 GCA_000340885.1
453 Clostridium chromiireducens GCA_002029255.1
9r
736 Lactobacillus gasseri ATCC 33323 = JCM 1131 GCA_000014425.1
640 Lactobacillus hominis DSM 23910 = CRBIP 24.179 GCA_000296835.1
485 Lactobacillus iners DSM 13335 GCA_000160875.1
9s
274 Staphylococcus epidermidis ATCC 12228 GCA_000007645.1
237 Staphylococcus capitis subsp. capitis GCA_001028645.1
236 Staphylococcus haemolyticus JCSC1435 GCA_000009865.1
9t
960 Bacillus anthracis str. Ames GCA_000007845.1
960 Bacillus anthracis str. Sterne GCA_000008165.1
952 Bacillus cereus ATCC 14579 GCA_000007825.1
952 _5BBacillus thuringiensis_5D serovar konkukian str. 97-27 GCA_000008505.1
937 Bacillus thuringiensis YBT-1518 GCA_000497525.2
9u
834 Escherichia coli str. K-12 substr. MG1655 GCA_000005845.2
808 Escherichia coli UMN026 GCA_000026325.2
805 Shigella flexneri 2a str. 301 GCA_000006925.2
9v
884 Streptococcus mutans UA159 GCA_000007465.2
652 Streptococcus ratti FA-1 = DSM 20564 GCA_000286075.1
606 Streptococcus ferus DSM 20646 GCA_000372425.1
9w
559 Deinococcus radiodurans R1 GCA_000008565.1
313 Deinococcus soli Cha et al. 2016 GCA_001007995.1
306 Deinococcus deserti VCD115 GCA_000020685.1
9x
638 Enterococcus faecalis V583 GCA_000007785.1
591 Streptomyces cinnamoneus GCA_001885705.1
573 Enterococcus rivorum GCA_001742285.1
9y
585 Rhodobacter sphaeroides 2.4.1 GCA_000012905.2
522 Rhodobacter sphaeroides ATCC 17025 GCA_000016405.1
428 Defluviimonas alba GCA_001620265.1
9z
896 Streptococcus mutans UA159 GCA_000007465.2
641 Streptococcus gordonii str. Challis substr. CH1 GCA_000017005.1
636 Streptococcus ratti FA-1 = DSM 20564 GCA_000286075.1
9A
448 Lactobacillus gasseri ATCC 33323 = JCM 1131 GCA_000014425.1
443 Lactobacillus hominis DSM 23910 = CRBIP 24.179 GCA_000296835.1
323 Lactobacillus kefiranofaciens ZW3 GCA_000214785.1
9B
809 Enterococcus faecalis V583 GCA_000007785.1
769 Streptomyces cinnamoneus GCA_001885705.1
687 Enterococcus rivorum GCA_001742285.1
9C
455 Enterococcus faecalis V583 GCA_000007785.1
425 Enterococcus dispar ATCC 51266 GCA_000406945.1

424 Enterococcus phoeniculicola ATCC BAA-412 GCA_000407505.1
9D
568 Staphylococcus epidermidis ATCC 12228 GCA_000007645.1
545 Staphylococcus capitis subsp. capitis GCA_001028645.1
474 Staphylococcus aureus subsp. aureus NCTC 8325 GCA_000013425.1
9E
396 Lactobacillus gasseri ATCC 33323 = JCM 1131 GCA_000014425.1
360 Lactobacillus hominis DSM 23910 = CRBIP 24.179 GCA_000296835.1
310 Lactobacillus iners DSM 13335 GCA_000160875.1
9F
935 Escherichia coli O157_3AH7 str. Sakai GCA_000008865.1
935 Escherichia coli UMN026 GCA_000026325.2
935 Escherichia coli str. K-12 substr. MG1655 GCA_000005845.2
924 Escherichia coli O83_3AH1 str. NRG 857C GCA_000183345.1
921 Shigella flexneri 2a str. 301 GCA_000006925.2
9G
661 Streptococcus mutans UA159 GCA_000007465.2
458 Streptococcus agalactiae 2603V_2FR GCA_000007265.1
455 Streptococcus gordonii str. Challis substr. CH1 GCA_000017005.1
9H
414 Deinococcus radiodurans R1 GCA_000008565.1
177 Deinococcus gobiensis I-0 GCA_000252445.1
167 Deinococcus deserti VCD115 GCA_000020685.1
9I
913 Lactobacillus gasseri ATCC 33323 = JCM 1131 GCA_000014425.1
848 Lactobacillus hominis DSM 23910 = CRBIP 24.179 GCA_000296835.1
589 Lactobacillus psittaci DSM 15354 GCA_000425905.1
9J
800 Staphylococcus epidermidis ATCC 12228 GCA_000007645.1
781 Staphylococcus capitis subsp. capitis GCA_001028645.1
738 Staphylococcus aureus subsp. aureus NCTC 8325 GCA_000013425.1
9K
540 Bacillus pseudomycoides DSM 12442 GCA_000161455.1
525 Bacillus anthracis str. Ames GCA_000007845.1
525 Bacillus anthracis str. Sterne GCA_000008165.1
525 _5BBacillus thuringiensis_5D serovar konkukian str. 97-27 GCA_000008505.1
500 Bacillus cereus ATCC 14579 GCA_000007825.1
9L
823 Lactobacillus gasseri ATCC 33323 = JCM 1131 GCA_000014425.1
764 Lactobacillus hominis DSM 23910 = CRBIP 24.179 GCA_000296835.1
596 Lactobacillus iners DSM 13335 GCA_000160875.1
9M
775 Clostridium beijerinckii GCA_000833105.2
710 Clostridium saccharoperbutylacetonicum N1-4_28HMT_29 GCA_000340885.1
709 Clostridium saccharobutylicum DSM 13864 GCA_000473995.1
9N
687 Enterococcus faecalis V583 GCA_000007785.1
640 Streptomyces cinnamoneus GCA_001885705.1
555 Enterococcus massiliensis GCA_001050095.1
9O
731 Staphylococcus epidermidis ATCC 12228 GCA_000007645.1
713 Staphylococcus capitis subsp. capitis GCA_001028645.1
643 Staphylococcus haemolyticus JCSC1435 GCA_000009865.1
9P
580 Lactobacillus gasseri ATCC 33323 = JCM 1131 GCA_000014425.1
557 Lactobacillus hominis DSM 23910 = CRBIP 24.179 GCA_000296835.1
441 Lactobacillus iners DSM 13335 GCA_000160875.1
9Q
744 Rhodobacter sphaeroides 2.4.1 GCA_000012905.2
677 Rhodobacter sphaeroides ATCC 17025 GCA_000016405.1
545 Pseudorhodobacter psychrotolerans GCA_001294535.1
9R
541 Bacillus anthracis str. Ames GCA_000007845.1
541 Bacillus anthracis str. Sterne GCA_000008165.1
541 _5BBacillus thuringiensis_5D serovar konkukian str. 97-27 GCA_000008505.1
525 Bacillus cereus ATCC 14579 GCA_000007825.1
525 Bacillus thuringiensis YBT-1518 GCA_000497525.2
516 Bacillus pseudomycoides DSM 12442 GCA_000161455.1
9S
793 Clostridium beijerinckii GCA_000833105.2
748 Clostridium puniceum GCA_002006345.1
748 Clostridium saccharoperbutylacetonicum N1-4_28HMT_29 GCA_000340885.1
719 Clostridium saccharobutylicum DSM 13864 GCA_000473995.1
9T
985 Deinococcus radiodurans R1 GCA_000008565.1
622 Deinococcus deserti VCD115 GCA_000020685.1

581 Deinococcus gobiensis I-0 GCA_000252445.1
9U
582 Deinococcus radiodurans R1 GCA_000008565.1
372 Deinococcus gobiensis I-0 GCA_000252445.1
363 Deinococcus marmoris DSM 12784 GCA_000701405.1
363 Deinococcus swuensis GCA_000800395.1
9V
675 Streptococcus mutans UA159 GCA_000007465.2
478 Streptococcus gordonii str. Challis substr. CH1 GCA_000017005.1
471 Streptococcus ratti FA-1 = DSM 20564 GCA_000286075.1
9W
498 Clostridium beijerinckii GCA_000833105.2
496 Clostridium saccharobutylicum DSM 13864 GCA_000473995.1
485 Clostridium saccharoperbutylacetonicum N1-4_28HMT_29 GCA_000340885.1
9X
869 Streptococcus mutans UA159 GCA_000007465.2
658 Streptococcus ratti FA-1 = DSM 20564 GCA_000286075.1
619 Streptococcus gordonii str. Challis substr. CH1 GCA_000017005.1
9Y
330 Bifidobacterium adolescentis ATCC 15703 GCA_000010425.1
287 Bifidobacterium breve DSM 20213 = JCM 1192 GCA_001025175.1
286 Bifidobacterium lemurum GCA_001895165.1
286 Bifidobacterium mongoliense DSM 21395 GCA_000741285.1
9Z
598 Rhodobacter sphaeroides 2.4.1 GCA_000012905.2
551 Rhodobacter sphaeroides ATCC 17025 GCA_000016405.1
439 Pseudorhodobacter psychrotolerans GCA_001294535.1
a0
822 Lactobacillus gasseri ATCC 33323 = JCM 1131 GCA_000014425.1
783 Lactobacillus hominis DSM 23910 = CRBIP 24.179 GCA_000296835.1
567 Lactobacillus hamsteri DSM 5661 = JCM 6256 GCA_000615445.1
567 Lactobacillus psittaci DSM 15354 GCA_000425905.1
a1
833 Enterococcus faecalis V583 GCA_000007785.1
786 Streptomyces cinnamoneus GCA_001885705.1
748 Enterococcus rivorum GCA_001742285.1
a2
767 Deinococcus radiodurans R1 GCA_000008565.1
495 Deinococcus deserti VCD115 GCA_000020685.1
466 Deinococcus hopiensis KR-140 GCA_900176165.1
a3
768 Lactobacillus gasseri ATCC 33323 = JCM 1131 GCA_000014425.1
709 Lactobacillus hominis DSM 23910 = CRBIP 24.179 GCA_000296835.1
533 Lactobacillus iners DSM 13335 GCA_000160875.1
a4
553 Bifidobacterium adolescentis ATCC 15703 GCA_000010425.1
454 Bifidobacterium dentium JCM 1195 = DSM 20436 GCA_001042595.1
432 Bifidobacterium callitrichos DSM 23973 GCA_000741175.1
a5
569 Rhodobacter sphaeroides 2.4.1 GCA_000012905.2
539 Rhodobacter sphaeroides ATCC 17025 GCA_000016405.1
460 Pseudorhodobacter psychrotolerans GCA_001294535.1
a6
623 Rhodobacter sphaeroides 2.4.1 GCA_000012905.2
561 Rhodobacter sphaeroides ATCC 17025 GCA_000016405.1
460 Defluviimonas alba GCA_001620265.1
a7
520 Clostridium beijerinckii GCA_000833105.2
507 Clostridium saccharoperbutylacetonicum N1-4_28HMT_29 GCA_000340885.1
500 Clostridium saccharobutylicum DSM 13864 GCA_000473995.1
a8
754 Clostridium beijerinckii GCA_000833105.2
753 Clostridium saccharoperbutylacetonicum N1-4_28HMT_29 GCA_000340885.1
719 Clostridium saccharobutylicum DSM 13864 GCA_000473995.1
a9
734 Rhodobacter sphaeroides 2.4.1 GCA_000012905.2
688 Rhodobacter sphaeroides ATCC 17025 GCA_000016405.1
598 Gemmobacter aquatilis GCA_900110025.1
aa
388 Rhodobacter sphaeroides 2.4.1 GCA_000012905.2
358 Rhodobacter sphaeroides ATCC 17025 GCA_000016405.1
320 Gemmobacter aquatilis GCA_900110025.1
ab
393 Rhodobacter sphaeroides 2.4.1 GCA_000012905.2
320 Rhodobacter sphaeroides ATCC 17025 GCA_000016405.1
269 Gemmobacter megaterium GCA_900156815.1

269 Pseudorhodobacter psychrotolerans GCA_001294535.1
ac
726 Rhodobacter sphaeroides 2.4.1 GCA_000012905.2
679 Rhodobacter sphaeroides ATCC 17025 GCA_000016405.1
591 Defluviimonas alba GCA_001620265.1
ad
528 Bacillus anthracis str. Ames GCA_000007845.1
528 Bacillus anthracis str. Sterne GCA_000008165.1
528 Bacillus cereus ATCC 14579 GCA_000007825.1
528 Bacillus pseudomycoides DSM 12442 GCA_000161455.1
528 Bacillus thuringiensis YBT-1518 GCA_000497525.2
528 _5BBacillus thuringiensis_5D serovar konkukian str. 97-27 GCA_000008505.1
521 Bacillus mycoides GCA_000832605.1
472 Bacillus cytotoxicus NVH 391-98 GCA_000017425.1
ae
649 Enterococcus faecalis V583 GCA_000007785.1
602 Streptomyces cinnamoneus GCA_001885705.1
560 Enterococcus rivorum GCA_001742285.1
af
795 Deinococcus radiodurans R1 GCA_000008565.1
522 Deinococcus deserti VCD115 GCA_000020685.1
454 Deinococcus hopiensis KR-140 GCA_900176165.1
ag
574 Bacillus thuringiensis YBT-1518 GCA_000497525.2
564 Bacillus anthracis str. Ames GCA_000007845.1
564 Bacillus anthracis str. Sterne GCA_000008165.1
564 Bacillus cereus ATCC 14579 GCA_000007825.1
564 _5BBacillus thuringiensis_5D serovar konkukian str. 97-27 GCA_000008505.1
549 Bacillus mycoides GCA_000832605.1
ah
802 Rhodobacter sphaeroides 2.4.1 GCA_000012905.2
745 Rhodobacter sphaeroides ATCC 17025 GCA_000016405.1
613 Pseudorhodobacter psychrotolerans GCA_001294535.1
ai
851 Streptococcus mutans UA159 GCA_000007465.2
603 Streptococcus ratti FA-1 = DSM 20564 GCA_000286075.1
564 Streptococcus halotolerans GCA_001598035.1
aj
832 Streptococcus mutans UA159 GCA_000007465.2
592 Streptococcus ratti FA-1 = DSM 20564 GCA_000286075.1
537 Streptococcus ferus DSM 20646 GCA_000372425.1
ak
660 Streptococcus mutans UA159 GCA_000007465.2
466 Streptococcus ratti FA-1 = DSM 20564 GCA_000286075.1
451 Streptococcus iniae GCA_000831485.1
al
750 Streptococcus mutans UA159 GCA_000007465.2
545 Streptococcus ratti FA-1 = DSM 20564 GCA_000286075.1
494 Streptococcus sobrinus DSM 20742 = ATCC 33478 GCA_000686605.1
am
863 Lactobacillus gasseri ATCC 33323 = JCM 1131 GCA_000014425.1
802 Lactobacillus hominis DSM 23910 = CRBIP 24.179 GCA_000296835.1
562 Lactobacillus iners DSM 13335 GCA_000160875.1
an
569 Escherichia coli str. K-12 substr. MG1655 GCA_000005845.2
557 Escherichia coli O104_3AH4 str. 2011C-3493 GCA_000299455.1
543 Shigella flexneri 2a str. 301 GCA_000006925.2
ao
645 Rhodobacter sphaeroides 2.4.1 GCA_000012905.2
552 Rhodobacter sphaeroides ATCC 17025 GCA_000016405.1
475 Gemmobacter megaterium GCA_900156815.1
ap
842 Staphylococcus epidermidis ATCC 12228 GCA_000007645.1
765 Megasphaera cerevisiae DSM 20462 GCA_001045675.1
765 Staphylococcus warneri SG1 GCA_000332735.1
754 Staphylococcus capitis subsp. capitis GCA_001028645.1
aq
742 Staphylococcus epidermidis ATCC 12228 GCA_000007645.1
700 Staphylococcus capitis subsp. capitis GCA_001028645.1
631 Megasphaera cerevisiae DSM 20462 GCA_001045675.1
631 Staphylococcus warneri SG1 GCA_000332735.1
ar
527 Clostridium saccharoperbutylacetonicum N1-4_28HMT_29 GCA_000340885.1
512 Clostridium puniceum GCA_002006345.1
509 Clostridium beijerinckii GCA_000833105.2
as

633 Clostridium beijerinckii GCA_000833105.2
616 Clostridium saccharoperbutylacetonicum N1-4_28HMT_29 GCA_000340885.1
607 Clostridium saccharobutylicum DSM 13864 GCA_000473995.1
at
215 Lactobacillus gasseri ATCC 33323 = JCM 1131 GCA_000014425.1
203 Lactobacillus hominis DSM 23910 = CRBIP 24.179 GCA_000296835.1
108 Lactobacillus delbrueckii subsp. bulgaricus ATCC 11842 = JCM 1002 GCA_000056065.1
au
826 Deinococcus radiodurans R1 GCA_000008565.1
497 Deinococcus gobiensis I-0 GCA_000252445.1
485 Deinococcus deserti VCD115 GCA_000020685.1
av
641 Enterococcus faecalis V583 GCA_000007785.1
594 Streptomyces cinnamoneus GCA_001885705.1
559 Enterococcus rivorum GCA_001742285.1
aw
570 Streptococcus mutans UA159 GCA_000007465.2
376 Streptococcus gordonii str. Challis substr. CH1 GCA_000017005.1
373 Streptococcus equinus GCA_000964315.1
373 Streptococcus gallolyticus subsp. gallolyticus DSM 16831 GCA_002000985.1
ax
755 Lactobacillus gasseri ATCC 33323 = JCM 1131 GCA_000014425.1
687 Lactobacillus hominis DSM 23910 = CRBIP 24.179 GCA_000296835.1
442 Lactobacillus iners DSM 13335 GCA_000160875.1
ay
796 Staphylococcus epidermidis ATCC 12228 GCA_000007645.1
781 Staphylococcus capitis subsp. capitis GCA_001028645.1
742 Staphylococcus hominis subsp. hominis C80 GCA_000183685.1
az
401 Staphylococcus epidermidis ATCC 12228 GCA_000007645.1
363 Staphylococcus haemolyticus JCSC1435 GCA_000009865.1
347 Staphylococcus hominis subsp. hominis C80 GCA_000183685.1
aA
534 Clostridium beijerinckii GCA_000833105.2
497 Clostridium saccharoperbutylacetonicum N1-4_28HMT_29 GCA_000340885.1
474 Clostridium saccharobutylicum DSM 13864 GCA_000473995.1
aB
453 Streptococcus mutans UA159 GCA_000007465.2
333 Streptococcus ratti FA-1 = DSM 20564 GCA_000286075.1
319 Streptococcus sobrinus DSM 20742 = ATCC 33478 GCA_000686605.1
aC
655 Clostridium beijerinckii GCA_000833105.2
601 Clostridium saccharoperbutylacetonicum N1-4_28HMT_29 GCA_000340885.1
567 Clostridium puniceum GCA_002006345.1
aD
552 Clostridium beijerinckii GCA_000833105.2
536 Clostridium saccharoperbutylacetonicum N1-4_28HMT_29 GCA_000340885.1
529 Clostridium saccharobutylicum DSM 13864 GCA_000473995.1
aE
730 Bifidobacterium adolescentis ATCC 15703 GCA_000010425.1
637 Bifidobacterium dentium JCM 1195 = DSM 20436 GCA_001042595.1
596 Bifidobacterium angulatum DSM 20098 = JCM 7096 GCA_001025155.1
aF
548 Clostridium puniceum GCA_002006345.1
534 Clostridium beijerinckii GCA_000833105.2
533 Clostridium saccharoperbutylacetonicum N1-4_28HMT_29 GCA_000340885.1
aG
843 Deinococcus radiodurans R1 GCA_000008565.1
519 Deinococcus deserti VCD115 GCA_000020685.1
505 Deinococcus hopiensis KR-140 GCA_900176165.1
aH
510 Streptococcus mutans UA159 GCA_000007465.2
375 Streptococcus ratti FA-1 = DSM 20564 GCA_000286075.1
365 Streptococcus macacae NCTC 11558 GCA_000187995.3
aI
712 Bifidobacterium adolescentis ATCC 15703 GCA_000010425.1
612 Bifidobacterium dentium JCM 1195 = DSM 20436 GCA_001042595.1
553 Bifidobacterium angulatum DSM 20098 = JCM 7096 GCA_001025155.1
aJ
628 Clostridium beijerinckii GCA_000833105.2
606 Clostridium saccharoperbutylacetonicum N1-4_28HMT_29 GCA_000340885.1
597 Clostridium saccharobutylicum DSM 13864 GCA_000473995.1
aK
658 Staphylococcus epidermidis ATCC 12228 GCA_000007645.1
602 Staphylococcus capitis subsp. capitis GCA_001028645.1
601 Staphylococcus simulans GCA_001559115.1

aL
746 Streptococcus mutans UA159 GCA_000007465.2
572 Streptococcus ratti FA-1 = DSM 20564 GCA_000286075.1
506 Streptococcus ferus DSM 20646 GCA_000372425.1
aM
608 Rhodobacter sphaeroides 2.4.1 GCA_000012905.2
553 Rhodobacter sphaeroides ATCC 17025 GCA_000016405.1
460 Gemmobacter megaterium GCA_900156815.1
aN
612 Rhodobacter sphaeroides 2.4.1 GCA_000012905.2
582 Rhodobacter sphaeroides ATCC 17025 GCA_000016405.1
507 Pseudorhodobacter psychrotolerans GCA_001294535.1
aO
707 Staphylococcus epidermidis ATCC 12228 GCA_000007645.1
684 Staphylococcus capitis subsp. capitis GCA_001028645.1
659 Staphylococcus warneri SG1 GCA_000332735.1
aP
362 Streptococcus mutans UA159 GCA_000007465.2
266 Streptococcus equinus GCA_000964315.1
262 Streptococcus gallolyticus subsp. gallolyticus DSM 16831 GCA_002000985.1
262 Streptococcus gordonii str. Challis substr. CH1 GCA_000017005.1
aQ
900 Clostridium beijerinckii GCA_000833105.2
868 Clostridium saccharoperbutylacetonicum N1-4_28HMT_29 GCA_000340885.1
838 Clostridium puniceum GCA_002006345.1
aR
741 Enterococcus faecalis V583 GCA_000007785.1
694 Streptomyces cinnamoneus GCA_001885705.1
621 Enterococcus massiliensis GCA_001050095.1
aS
736 Bacillus anthracis str. Ames GCA_000007845.1
736 Bacillus anthracis str. Sterne GCA_000008165.1
736 _5BBacillus thuringiensis_5D serovar konkukian str. 97-27 GCA_000008505.1
721 Bacillus cereus ATCC 14579 GCA_000007825.1
711 Bacillus thuringiensis YBT-1518 GCA_000497525.2
aT
863 Lactobacillus gasseri ATCC 33323 = JCM 1131 GCA_000014425.1
820 Lactobacillus hominis DSM 23910 = CRBIP 24.179 GCA_000296835.1
656 Lactobacillus iners DSM 13335 GCA_000160875.1
aU
745 Rhodobacter sphaeroides 2.4.1 GCA_000012905.2
666 Rhodobacter sphaeroides ATCC 17025 GCA_000016405.1
543 Gemmobacter aquatilis GCA_900110025.1
aV
692 Streptococcus mutans UA159 GCA_000007465.2
476 Streptococcus ratti FA-1 = DSM 20564 GCA_000286075.1
468 Streptococcus equinus GCA_000964315.1
aW
791 Clostridium beijerinckii GCA_000833105.2
715 Clostridium saccharoperbutylacetonicum N1-4_28HMT_29 GCA_000340885.1
681 Clostridium saccharobutylicum DSM 13864 GCA_000473995.1
aX
425 Staphylococcus epidermidis ATCC 12228 GCA_000007645.1
392 Staphylococcus capitis subsp. capitis GCA_001028645.1
388 Staphylococcus aureus subsp. aureus NCTC 8325 GCA_000013425.1
388 Staphylococcus haemolyticus JCSC1435 GCA_000009865.1
aY
813 Bacillus cereus ATCC 14579 GCA_000007825.1
813 _5BBacillus thuringiensis_5D serovar konkukian str. 97-27 GCA_000008505.1
812 Bacillus anthracis str. Ames GCA_000007845.1
812 Bacillus anthracis str. Sterne GCA_000008165.1
807 Bacillus thuringiensis YBT-1518 GCA_000497525.2
aZ
649 Clostridium beijerinckii GCA_000833105.2
617 Clostridium saccharoperbutylacetonicum N1-4_28HMT_29 GCA_000340885.1
612 Clostridium puniceum GCA_002006345.1
b0
642 Deinococcus radiodurans R1 GCA_000008565.1
375 Deinococcus gobiensis I-0 GCA_000252445.1
332 Deinococcus deserti VCD115 GCA_000020685.1
332 Deinococcus hopiensis KR-140 GCA_900176165.1
b1
543 Deinococcus radiodurans R1 GCA_000008565.1
299 Deinococcus deserti VCD115 GCA_000020685.1
282 Deinococcus gobiensis I-0 GCA_000252445.1
b2

615 Staphylococcus epidermidis ATCC 12228 GCA_000007645.1
559 Staphylococcus capitis subsp. capitis GCA_001028645.1
513 Staphylococcus aureus subsp. aureus NCTC 8325 GCA_000013425.1
b3
687 Clostridium beijerinckii GCA_000833105.2
641 Clostridium saccharoperbutylacetonicum N1-4_28HMT_29 GCA_000340885.1
605 Clostridium puniceum GCA_002006345.1
b4
920 Staphylococcus epidermidis ATCC 12228 GCA_000007645.1
891 Staphylococcus capitis subsp. capitis GCA_001028645.1
813 Staphylococcus aureus subsp. aureus NCTC 8325 GCA_000013425.1
b5
848 Streptococcus mutans UA159 GCA_000007465.2
640 Streptococcus ratti FA-1 = DSM 20564 GCA_000286075.1
619 Streptococcus gordonii str. Challis substr. CH1 GCA_000017005.1
b6
787 Staphylococcus epidermidis ATCC 12228 GCA_000007645.1
743 Staphylococcus capitis subsp. capitis GCA_001028645.1
714 Staphylococcus aureus subsp. aureus NCTC 8325 GCA_000013425.1
b7
705 Staphylococcus epidermidis ATCC 12228 GCA_000007645.1
678 Staphylococcus capitis subsp. capitis GCA_001028645.1
642 Staphylococcus warneri SG1 GCA_000332735.1
b8
607 Staphylococcus epidermidis ATCC 12228 GCA_000007645.1
549 Staphylococcus capitis subsp. capitis GCA_001028645.1
520 Staphylococcus warneri SG1 GCA_000332735.1
b9
750 Streptococcus mutans UA159 GCA_000007465.2
543 Streptococcus ratti FA-1 = DSM 20564 GCA_000286075.1
531 Streptococcus equinus GCA_000964315.1
ba
786 Bifidobacterium adolescentis ATCC 15703 GCA_000010425.1
624 Bifidobacterium dentium JCM 1195 = DSM 20436 GCA_001042595.1
609 Bifidobacterium breve DSM 20213 = JCM 1192 GCA_001025175.1
bb
656 Streptococcus mutans UA159 GCA_000007465.2
531 Streptococcus ratti FA-1 = DSM 20564 GCA_000286075.1
499 Streptococcus ferus DSM 20646 GCA_000372425.1
bc
526 Bifidobacterium adolescentis ATCC 15703 GCA_000010425.1
456 Bifidobacterium dentium JCM 1195 = DSM 20436 GCA_001042595.1
434 Bifidobacterium thermophilum GCA_000741495.1
bd
463 Streptococcus mutans UA159 GCA_000007465.2
352 Streptococcus ratti FA-1 = DSM 20564 GCA_000286075.1
345 Streptococcus equinus GCA_000964315.1
345 Streptococcus gallolyticus subsp. gallolyticus DSM 16831 GCA_002000985.1
be
533 Escherichia coli O157_3AH7 str. Sakai GCA_000008865.1
533 Escherichia coli str. K-12 substr. MG1655 GCA_000005845.2
524 Escherichia coli UMN026 GCA_000026325.2
508 Escherichia coli O104_3AH4 str. 2011C-3493 GCA_000299455.1
508 Escherichia coli O83_3AH1 str. NRG 857C GCA_000183345.1
508 Shigella flexneri 2a str. 301 GCA_000006925.2
bf
687 Lactobacillus gasseri ATCC 33323 = JCM 1131 GCA_000014425.1
616 Lactobacillus hominis DSM 23910 = CRBIP 24.179 GCA_000296835.1
450 Lactobacillus hamsteri DSM 5661 = JCM 6256 GCA_000615445.1
bg
743 Clostridium beijerinckii GCA_000833105.2
708 Clostridium saccharoperbutylacetonicum N1-4_28HMT_29 GCA_000340885.1
657 Clostridium puniceum GCA_002006345.1
bh
683 Streptococcus mutans UA159 GCA_000007465.2
471 Streptococcus ratti FA-1 = DSM 20564 GCA_000286075.1
443 Streptococcus ferus DSM 20646 GCA_000372425.1
bi
765 Staphylococcus epidermidis ATCC 12228 GCA_000007645.1
749 Staphylococcus capitis subsp. capitis GCA_001028645.1
660 Staphylococcus aureus subsp. aureus NCTC 8325 GCA_000013425.1
bj
716 Streptococcus mutans UA159 GCA_000007465.2
573 Streptococcus ratti FA-1 = DSM 20564 GCA_000286075.1
501 Streptococcus merionis DSM 19192 GCA_000380085.1
bk

539 Clostridium beijerinckii GCA_000833105.2
518 Clostridium saccharoperbutylacetonicum N1-4_28HMT_29 GCA_000340885.1
482 Clostridium saccharobutylicum DSM 13864 GCA_000473995.1
bl
831 Lactobacillus gasseri ATCC 33323 = JCM 1131 GCA_000014425.1
743 Lactobacillus hominis DSM 23910 = CRBIP 24.179 GCA_000296835.1
563 Lactobacillus iners DSM 13335 GCA_000160875.1
bm
658 Deinococcus radiodurans R1 GCA_000008565.1
413 Deinococcus gobiensis I-0 GCA_000252445.1
373 Deinococcus deserti VCD115 GCA_000020685.1
bn
662 Lactobacillus gasseri ATCC 33323 = JCM 1131 GCA_000014425.1
597 Lactobacillus hominis DSM 23910 = CRBIP 24.179 GCA_000296835.1
452 Lactobacillus iners DSM 13335 GCA_000160875.1
bo
622 Streptococcus mutans UA159 GCA_000007465.2
506 Streptococcus ratti FA-1 = DSM 20564 GCA_000286075.1
485 Streptococcus ovis DSM 16829 GCA_000380125.1
485 Streptococcus pyogenes M1 GAS GCA_000006785.2
485 Streptococcus uberis 0140J GCA_000009545.1
bp
237 Rhodobacter sphaeroides 2.4.1 GCA_000012905.2
184 Rhodobacter sphaeroides ATCC 17025 GCA_000016405.1
172 Rhodobacter vinaykumarii GCA_900156695.1
bq
536 Lactobacillus gasseri ATCC 33323 = JCM 1131 GCA_000014425.1
491 Lactobacillus hominis DSM 23910 = CRBIP 24.179 GCA_000296835.1
408 Lactobacillus iners DSM 13335 GCA_000160875.1
br
459 Staphylococcus epidermidis ATCC 12228 GCA_000007645.1
453 Staphylococcus capitis subsp. capitis GCA_001028645.1
398 Staphylococcus warneri SG1 GCA_000332735.1
bs
728 Escherichia coli str. K-12 substr. MG1655 GCA_000005845.2
706 Shigella flexneri 2a str. 301 GCA_000006925.2
704 Escherichia coli O104_3AH4 str. 2011C-3493 GCA_000299455.1
bt
748 Lactobacillus gasseri ATCC 33323 = JCM 1131 GCA_000014425.1
698 Lactobacillus hominis DSM 23910 = CRBIP 24.179 GCA_000296835.1
532 Lactobacillus iners DSM 13335 GCA_000160875.1
bu
684 Clostridium beijerinckii GCA_000833105.2
652 Clostridium saccharoperbutylacetonicum N1-4_28HMT_29 GCA_000340885.1
645 Clostridium saccharobutylicum DSM 13864 GCA_000473995.1
bv
878 Staphylococcus epidermidis ATCC 12228 GCA_000007645.1
816 Staphylococcus capitis subsp. capitis GCA_001028645.1
765 Staphylococcus hominis subsp. hominis C80 GCA_000183685.1
bw
529 Lactobacillus gasseri ATCC 33323 = JCM 1131 GCA_000014425.1
498 Lactobacillus hominis DSM 23910 = CRBIP 24.179 GCA_000296835.1
405 Lactobacillus iners DSM 13335 GCA_000160875.1
bx
606 Clostridium beijerinckii GCA_000833105.2
580 Clostridium saccharoperbutylacetonicum N1-4_28HMT_29 GCA_000340885.1
558 Clostridium saccharobutylicum DSM 13864 GCA_000473995.1
by
796 Clostridium beijerinckii GCA_000833105.2
768 Clostridium saccharoperbutylacetonicum N1-4_28HMT_29 GCA_000340885.1
755 Clostridium saccharobutylicum DSM 13864 GCA_000473995.1
bz
470 Streptococcus mutans UA159 GCA_000007465.2
279 Streptococcus gordonii str. Challis substr. CH1 GCA_000017005.1
274 Streptococcus anginosus C238 GCA_000463505.1
274 Streptococcus ratti FA-1 = DSM 20564 GCA_000286075.1
bA
593 Lactobacillus gasseri ATCC 33323 = JCM 1131 GCA_000014425.1
541 Lactobacillus hominis DSM 23910 = CRBIP 24.179 GCA_000296835.1
434 Lactobacillus iners DSM 13335 GCA_000160875.1
bB
752 Clostridium beijerinckii GCA_000833105.2
711 Clostridium saccharobutylicum DSM 13864 GCA_000473995.1
710 Clostridium saccharoperbutylacetonicum N1-4_28HMT_29 GCA_000340885.1
bC
772 Staphylococcus epidermidis ATCC 12228 GCA_000007645.1

747 Staphylococcus capitis subsp. capitis GCA_001028645.1
652 Staphylococcus aureus subsp. aureus NCTC 8325 GCA_000013425.1
bD
607 Deinococcus radiodurans R1 GCA_000008565.1
396 Deinococcus deserti VCD115 GCA_000020685.1
372 Deinococcus gobiensis I-0 GCA_000252445.1
bE
683 Deinococcus radiodurans R1 GCA_000008565.1
442 Deinococcus gobiensis I-0 GCA_000252445.1
426 Deinococcus deserti VCD115 GCA_000020685.1
bF
721 Streptococcus mutans UA159 GCA_000007465.2
575 Streptococcus ratti FA-1 = DSM 20564 GCA_000286075.1
567 Streptococcus gordonii str. Challis substr. CH1 GCA_000017005.1
bG
510 Clostridium beijerinckii GCA_000833105.2
510 Clostridium saccharoperbutylacetonicum N1-4_28HMT_29 GCA_000340885.1
495 Clostridium puniceum GCA_002006345.1
483 Clostridium taeniosporum GCA_001735765.1
bH
789 Clostridium beijerinckii GCA_000833105.2
789 Clostridium saccharoperbutylacetonicum N1-4_28HMT_29 GCA_000340885.1
773 Clostridium puniceum GCA_002006345.1
748 Clostridium butyricum GCA_001456065.2
bI
741 Clostridium beijerinckii GCA_000833105.2
692 Clostridium saccharoperbutylacetonicum N1-4_28HMT_29 GCA_000340885.1
672 Clostridium saccharobutylicum DSM 13864 GCA_000473995.1
bJ
538 Bifidobacterium adolescentis ATCC 15703 GCA_000010425.1
443 Bifidobacterium angulatum DSM 20098 = JCM 7096 GCA_001025155.1
406 Bifidobacterium gallicum DSM 20093 = LMG 11596 GCA_000741205.1
bK
616 Deinococcus radiodurans R1 GCA_000008565.1
367 Deinococcus deserti VCD115 GCA_000020685.1
366 Deinococcus gobiensis I-0 GCA_000252445.1
bL
683 Escherichia coli O157_3AH7 str. Sakai GCA_000008865.1
683 Escherichia coli str. K-12 substr. MG1655 GCA_000005845.2
678 Escherichia coli UMN026 GCA_000026325.2
650 Escherichia coli IAI39 GCA_000026345.1
650 Shigella flexneri 2a str. 301 GCA_000006925.2
bM
833 Streptococcus mutans UA159 GCA_000007465.2
608 Streptococcus ratti FA-1 = DSM 20564 GCA_000286075.1
568 Streptococcus criceti HS-6 GCA_000187975.3
bN
675 Lactobacillus gasseri ATCC 33323 = JCM 1131 GCA_000014425.1
648 Lactobacillus hominis DSM 23910 = CRBIP 24.179 GCA_000296835.1
467 Lactobacillus iners DSM 13335 GCA_000160875.1
bO
784 Rhodobacter sphaeroides 2.4.1 GCA_000012905.2
706 Rhodobacter sphaeroides ATCC 17025 GCA_000016405.1
579 Gemmobacter aquatilis GCA_900110025.1
bP
550 Bacillus anthracis str. Ames GCA_000007845.1
550 Bacillus anthracis str. Sterne GCA_000008165.1
550 _5BBacillus thuringiensis_5D serovar konkukian str. 97-27 GCA_000008505.1
533 Bacillus cereus ATCC 14579 GCA_000007825.1
533 Bacillus thuringiensis YBT-1518 GCA_000497525.2
508 Bacillus mycoides GCA_000832605.1
bQ
765 Clostridium beijerinckii GCA_000833105.2
698 Clostridium saccharoperbutylacetonicum N1-4_28HMT_29 GCA_000340885.1
693 Clostridium saccharobutylicum DSM 13864 GCA_000473995.1
bR
640 Clostridium beijerinckii GCA_000833105.2
635 Clostridium saccharoperbutylacetonicum N1-4_28HMT_29 GCA_000340885.1
630 Clostridium puniceum GCA_002006345.1
bS
797 Deinococcus radiodurans R1 GCA_000008565.1
530 Deinococcus gobiensis I-0 GCA_000252445.1
517 Deinococcus deserti VCD115 GCA_000020685.1
bT
550 Deinococcus radiodurans R1 GCA_000008565.1
351 Deinococcus deserti VCD115 GCA_000020685.1

351 Deinococcus hopiensis KR-140 GCA_900176165.1
323 Deinococcus puniceus GCA_001644565.1
bU
681 Rhodobacter sphaeroides 2.4.1 GCA_000012905.2
609 Rhodobacter sphaeroides ATCC 17025 GCA_000016405.1
496 Gemmobacter aquatilis GCA_900110025.1
bV
893 Clostridium beijerinckii GCA_000833105.2
865 Clostridium saccharoperbutylacetonicum N1-4_28HMT_29 GCA_000340885.1
817 Clostridium saccharobutylicum DSM 13864 GCA_000473995.1
bW
658 Deinococcus radiodurans R1 GCA_000008565.1
449 Deinococcus deserti VCD115 GCA_000020685.1
422 Deinococcus soli Cha et al. 2016 GCA_001007995.1
bX
677 Lactobacillus gasseri ATCC 33323 = JCM 1131 GCA_000014425.1
622 Lactobacillus hominis DSM 23910 = CRBIP 24.179 GCA_000296835.1
497 Lactobacillus iners DSM 13335 GCA_000160875.1
bY
624 Streptococcus mutans UA159 GCA_000007465.2
474 Streptococcus ratti FA-1 = DSM 20564 GCA_000286075.1
445 Streptococcus macacae NCTC 11558 GCA_000187995.3
bZ
392 Rhodobacter sphaeroides 2.4.1 GCA_000012905.2
352 Rhodobacter sphaeroides ATCC 17025 GCA_000016405.1
276 Defluviimonas alba GCA_001620265.1
c0
851 Streptococcus mutans UA159 GCA_000007465.2
636 Streptococcus ratti FA-1 = DSM 20564 GCA_000286075.1
617 Streptococcus ferus DSM 20646 GCA_000372425.1
c1
668 Deinococcus radiodurans R1 GCA_000008565.1
433 Deinococcus deserti VCD115 GCA_000020685.1
427 Deinococcus soli Cha et al. 2016 GCA_001007995.1
c2
714 Enterococcus faecalis V583 GCA_000007785.1
674 Streptomyces cinnamoneus GCA_001885705.1
632 Enterococcus rivorum GCA_001742285.1
c3
616 Bifidobacterium adolescentis ATCC 15703 GCA_000010425.1
489 Bifidobacterium angulatum DSM 20098 = JCM 7096 GCA_001025155.1
488 Bifidobacterium thermophilum GCA_000741495.1
c4
421 Rhodobacter sphaeroides 2.4.1 GCA_000012905.2
348 Rhodobacter sphaeroides ATCC 17025 GCA_000016405.1
319 Gemmobacter nectariphilus DSM 15620 GCA_000429765.1
319 Rhodobacter vinaykumarii GCA_900156695.1
c5
667 Staphylococcus epidermidis ATCC 12228 GCA_000007645.1
636 Staphylococcus capitis subsp. capitis GCA_001028645.1
566 Staphylococcus hominis subsp. hominis C80 GCA_000183685.1
c6
625 Bifidobacterium adolescentis ATCC 15703 GCA_000010425.1
540 Bifidobacterium dentium JCM 1195 = DSM 20436 GCA_001042595.1
483 Bifidobacterium stellenboschense GCA_000741785.1
c7
460 Streptococcus mutans UA159 GCA_000007465.2
345 Streptococcus ratti FA-1 = DSM 20564 GCA_000286075.1
338 Streptococcus gordonii str. Challis substr. CH1 GCA_000017005.1
c8
737 Staphylococcus epidermidis ATCC 12228 GCA_000007645.1
705 Staphylococcus capitis subsp. capitis GCA_001028645.1
656 Staphylococcus warneri SG1 GCA_000332735.1
c9
644 Streptococcus mutans UA159 GCA_000007465.2
431 Streptococcus ratti FA-1 = DSM 20564 GCA_000286075.1
377 Streptococcus halotolerans GCA_001598035.1
ca
399 Staphylococcus epidermidis ATCC 12228 GCA_000007645.1
374 Staphylococcus capitis subsp. capitis GCA_001028645.1
342 Staphylococcus lugdunensis HKU09-01 GCA_000025085.1
cb
738 Enterococcus faecalis V583 GCA_000007785.1
691 Streptomyces cinnamoneus GCA_001885705.1
618 Enterococcus canis NBRC 100695 GCA_001544375.1
cc

755 Rhodobacter sphaeroides 2.4.1 GCA_000012905.2
709 Rhodobacter sphaeroides ATCC 17025 GCA_000016405.1
609 Defluviimonas alba GCA_001620265.1
cd
583 Deinococcus radiodurans R1 GCA_000008565.1
277 Deinococcus deserti VCD115 GCA_000020685.1
266 Deinococcus marmoris DSM 12784 GCA_000701405.1
266 Deinococcus proteolyticus MRP GCA_000190555.1
ce
618 Clostridium beijerinckii GCA_000833105.2
589 Clostridium saccharoperbutylacetonicum N1-4_28HMT_29 GCA_000340885.1
579 Clostridium puniceum GCA_002006345.1
cf
306 Streptococcus mutans UA159 GCA_000007465.2
256 Streptococcus ratti FA-1 = DSM 20564 GCA_000286075.1
245 Streptococcus sanguinis SK36 GCA_000014205.1
cg
479 Rhodobacter sphaeroides 2.4.1 GCA_000012905.2
426 Rhodobacter sphaeroides ATCC 17025 GCA_000016405.1
339 Gemmobacter aquatilis GCA_900110025.1
ch
602 Rhodobacter sphaeroides 2.4.1 GCA_000012905.2
573 Rhodobacter sphaeroides ATCC 17025 GCA_000016405.1
459 Defluviimonas indica GCA_900106675.1
ci
406 Clostridium saccharoperbutylacetonicum N1-4_28HMT_29 GCA_000340885.1
403 Clostridium beijerinckii GCA_000833105.2
376 Clostridium puniceum GCA_002006345.1
376 Clostridium saccharobutylicum DSM 13864 GCA_000473995.1
cj
715 Clostridium beijerinckii GCA_000833105.2
673 Clostridium saccharoperbutylacetonicum N1-4_28HMT_29 GCA_000340885.1
668 Clostridium butyricum GCA_001456065.2
ck
780 Streptococcus mutans UA159 GCA_000007465.2
560 Streptococcus ratti FA-1 = DSM 20564 GCA_000286075.1
533 Streptococcus equinus GCA_000964315.1
cl
742 Clostridium beijerinckii GCA_000833105.2
705 Clostridium saccharoperbutylacetonicum N1-4_28HMT_29 GCA_000340885.1
669 Clostridium puniceum GCA_002006345.1
cm
920 Clostridium beijerinckii GCA_000833105.2
889 Clostridium saccharoperbutylacetonicum N1-4_28HMT_29 GCA_000340885.1
872 Clostridium saccharobutylicum DSM 13864 GCA_000473995.1
cn
701 Rhodobacter sphaeroides 2.4.1 GCA_000012905.2
627 Rhodobacter sphaeroides ATCC 17025 GCA_000016405.1
587 Gemmobacter aquatilis GCA_900110025.1
co
742 Bacillus anthracis str. Ames GCA_000007845.1
742 Bacillus anthracis str. Sterne GCA_000008165.1
742 _5BBacillus thuringiensis_5D serovar konkukian str. 97-27 GCA_000008505.1
740 Bacillus pseudomycoides DSM 12442 GCA_000161455.1
726 Bacillus cereus ATCC 14579 GCA_000007825.1
cp
700 Lactobacillus gasseri ATCC 33323 = JCM 1131 GCA_000014425.1
653 Lactobacillus hominis DSM 23910 = CRBIP 24.179 GCA_000296835.1
495 Lactobacillus jensenii GCA_001936235.1
cq
701 Lactobacillus gasseri ATCC 33323 = JCM 1131 GCA_000014425.1
629 Lactobacillus hominis DSM 23910 = CRBIP 24.179 GCA_000296835.1
473 Lactobacillus kalixensis DSM 16043 GCA_001434335.1
cr
523 Bacillus anthracis str. Ames GCA_000007845.1
523 Bacillus anthracis str. Sterne GCA_000008165.1
523 _5BBacillus thuringiensis_5D serovar konkukian str. 97-27 GCA_000008505.1
514 Bacillus cereus ATCC 14579 GCA_000007825.1
499 Bacillus thuringiensis YBT-1518 GCA_000497525.2
cs
701 Lactobacillus gasseri ATCC 33323 = JCM 1131 GCA_000014425.1
655 Lactobacillus hominis DSM 23910 = CRBIP 24.179 GCA_000296835.1
471 Lactobacillus iners DSM 13335 GCA_000160875.1
ct
683 Streptococcus mutans UA159 GCA_000007465.2
454 Streptococcus ratti FA-1 = DSM 20564 GCA_000286075.1

429 Streptococcus equinus GCA_000964315.1
cu
487 Rhodobacter sphaeroides 2.4.1 GCA_000012905.2
450 Rhodobacter sphaeroides ATCC 17025 GCA_000016405.1
379 Gemmobacter aquatilis GCA_900110025.1
cv
674 Rhodobacter sphaeroides 2.4.1 GCA_000012905.2
592 Rhodobacter sphaeroides ATCC 17025 GCA_000016405.1
560 Gemmobacter aquatilis GCA_900110025.1
cw
683 Staphylococcus epidermidis ATCC 12228 GCA_000007645.1
623 Staphylococcus capitis subsp. capitis GCA_001028645.1
542 Staphylococcus aureus subsp. aureus NCTC 8325 GCA_000013425.1
cx
677 Staphylococcus epidermidis ATCC 12228 GCA_000007645.1
653 Staphylococcus capitis subsp. capitis GCA_001028645.1
634 Staphylococcus haemolyticus JCSC1435 GCA_000009865.1
cy
785 Clostridium saccharoperbutylacetonicum N1-4_28HMT_29 GCA_000340885.1
784 Clostridium beijerinckii GCA_000833105.2
760 Clostridium saccharobutylicum DSM 13864 GCA_000473995.1
cz
574 Lactobacillus gasseri ATCC 33323 = JCM 1131 GCA_000014425.1
548 Lactobacillus hominis DSM 23910 = CRBIP 24.179 GCA_000296835.1
457 Lactobacillus iners DSM 13335 GCA_000160875.1
cA
697 Lactobacillus gasseri ATCC 33323 = JCM 1131 GCA_000014425.1
664 Lactobacillus hominis DSM 23910 = CRBIP 24.179 GCA_000296835.1
532 Lactobacillus iners DSM 13335 GCA_000160875.1
cB
770 Enterococcus faecalis V583 GCA_000007785.1
724 Streptomyces cinnamoneus GCA_001885705.1
672 Enterococcus rivorum GCA_001742285.1
cC
752 Streptococcus mutans UA159 GCA_000007465.2
587 Streptococcus ratti FA-1 = DSM 20564 GCA_000286075.1
510 Streptococcus equinus GCA_000964315.1
cD
598 Clostridium beijerinckii GCA_000833105.2
583 Clostridium saccharoperbutylacetonicum N1-4_28HMT_29 GCA_000340885.1
570 Clostridium puniceum GCA_002006345.1
cE
506 Escherichia coli O157_3AH7 str. Sakai GCA_000008865.1
506 Escherichia coli UMN026 GCA_000026325.2
506 Escherichia coli str. K-12 substr. MG1655 GCA_000005845.2
495 Escherichia coli O104_3AH4 str. 2011C-3493 GCA_000299455.1
495 Escherichia coli O83_3AH1 str. NRG 857C GCA_000183345.1
495 Shigella flexneri 2a str. 301 GCA_000006925.2
475 Escherichia coli IAI39 GCA_000026345.1
cF
716 Bacillus cereus ATCC 14579 GCA_000007825.1
702 Bacillus anthracis str. Ames GCA_000007845.1
702 Bacillus anthracis str. Sterne GCA_000008165.1
702 _5BBacillus thuringiensis_5D serovar konkukian str. 97-27 GCA_000008505.1
700 Bacillus thuringiensis YBT-1518 GCA_000497525.2
cG
650 Bifidobacterium adolescentis ATCC 15703 GCA_000010425.1
493 Bifidobacterium dentium JCM 1195 = DSM 20436 GCA_001042595.1
464 Bifidobacterium angulatum DSM 20098 = JCM 7096 GCA_001025155.1
cH
768 Clostridium beijerinckii GCA_000833105.2
755 Clostridium saccharoperbutylacetonicum N1-4_28HMT_29 GCA_000340885.1
727 Clostridium saccharobutylicum DSM 13864 GCA_000473995.1
cI
646 Streptococcus mutans UA159 GCA_000007465.2
506 Streptococcus ratti FA-1 = DSM 20564 GCA_000286075.1
471 Streptococcus equinus GCA_000964315.1
cJ
453 Staphylococcus epidermidis ATCC 12228 GCA_000007645.1
433 Staphylococcus capitis subsp. capitis GCA_001028645.1
420 Staphylococcus warneri SG1 GCA_000332735.1
cK
602 Enterococcus faecalis V583 GCA_000007785.1
555 Streptomyces cinnamoneus GCA_001885705.1
524 Enterococcus sulfureus ATCC 49903 GCA_000407605.1
cL

675 Rhodobacter sphaeroides 2.4.1 GCA_000012905.2
624 Rhodobacter sphaeroides ATCC 17025 GCA_000016405.1
490 Pseudorhodobacter wandonensis GCA_001202035.1
cM
701 Staphylococcus epidermidis ATCC 12228 GCA_000007645.1
657 Staphylococcus capitis subsp. capitis GCA_001028645.1
639 Staphylococcus lugdunensis HKU09-01 GCA_000025085.1
cN
762 Deinococcus radiodurans R1 GCA_000008565.1
474 Deinococcus gobiensis I-0 GCA_000252445.1
465 Deinococcus deserti VCD115 GCA_000020685.1
cO
658 Enterococcus faecalis V583 GCA_000007785.1
611 Streptomyces cinnamoneus GCA_001885705.1
579 Enterococcus hirae ATCC 9790 GCA_000271405.2
cP
726 Deinococcus radiodurans R1 GCA_000008565.1
475 Deinococcus deserti VCD115 GCA_000020685.1
442 Deinococcus gobiensis I-0 GCA_000252445.1
442 Deinococcus soli Cha et al. 2016 GCA_001007995.1
cQ
617 Clostridium saccharoperbutylacetonicum N1-4_28HMT_29 GCA_000340885.1
601 Clostridium beijerinckii GCA_000833105.2
586 Clostridium saccharobutylicum DSM 13864 GCA_000473995.1
cR
612 Rhodobacter sphaeroides 2.4.1 GCA_000012905.2
581 Rhodobacter sphaeroides ATCC 17025 GCA_000016405.1
473 Pseudorhodobacter wandonensis GCA_001202035.1
cS
569 Bifidobacterium adolescentis ATCC 15703 GCA_000010425.1
432 Bifidobacterium dentium JCM 1195 = DSM 20436 GCA_001042595.1
391 Bifidobacterium angulatum DSM 20098 = JCM 7096 GCA_001025155.1
cT
803 Clostridium beijerinckii GCA_000833105.2
787 Clostridium puniceum GCA_002006345.1
786 Clostridium saccharoperbutylacetonicum N1-4_28HMT_29 GCA_000340885.1
cU
621 Lactobacillus gasseri ATCC 33323 = JCM 1131 GCA_000014425.1
586 Lactobacillus hominis DSM 23910 = CRBIP 24.179 GCA_000296835.1
455 Lactobacillus iners DSM 13335 GCA_000160875.1
cV
359 Rhodobacter sphaeroides 2.4.1 GCA_000012905.2
324 Rhodobacter sphaeroides ATCC 17025 GCA_000016405.1
274 Pseudorhodobacter wandonensis GCA_001202035.1
cW
356 Bifidobacterium adolescentis ATCC 15703 GCA_000010425.1
301 Bifidobacterium dentium JCM 1195 = DSM 20436 GCA_001042595.1
289 Bifidobacterium callitrichos DSM 23973 GCA_000741175.1
cX
848 Lactobacillus gasseri ATCC 33323 = JCM 1131 GCA_000014425.1
777 Lactobacillus hominis DSM 23910 = CRBIP 24.179 GCA_000296835.1
610 Lactobacillus iners DSM 13335 GCA_000160875.1
cY
513 Rhodobacter sphaeroides 2.4.1 GCA_000012905.2
487 Rhodobacter sphaeroides ATCC 17025 GCA_000016405.1
361 Pseudorhodobacter wandonensis GCA_001202035.1
cZ
700 Deinococcus radiodurans R1 GCA_000008565.1
393 Deinococcus deserti VCD115 GCA_000020685.1
386 Deinococcus hopiensis KR-140 GCA_900176165.1
d0
752 Bacillus anthracis str. Ames GCA_000007845.1
752 Bacillus anthracis str. Sterne GCA_000008165.1
752 _5BBacillus thuringiensis_5D serovar konkukian str. 97-27 GCA_000008505.1
735 Bacillus pseudomycoides DSM 12442 GCA_000161455.1
733 Bacillus cereus ATCC 14579 GCA_000007825.1
d1
695 Rhodobacter sphaeroides 2.4.1 GCA_000012905.2
633 Rhodobacter sphaeroides ATCC 17025 GCA_000016405.1
539 Gemmobacter aquatilis GCA_900110025.1
d2
601 Staphylococcus epidermidis ATCC 12228 GCA_000007645.1
567 Staphylococcus capitis subsp. capitis GCA_001028645.1
536 Staphylococcus haemolyticus JCSC1435 GCA_000009865.1
d3
608 Streptococcus mutans UA159 GCA_000007465.2

431 Streptococcus ratti FA-1 = DSM 20564 GCA_000286075.1
387 Streptococcus ovis DSM 16829 GCA_000380125.1
d4
795 Staphylococcus epidermidis ATCC 12228 GCA_000007645.1
737 Staphylococcus capitis subsp. capitis GCA_001028645.1
710 Staphylococcus hominis subsp. hominis C80 GCA_000183685.1
d5
720 Clostridium beijerinckii GCA_000833105.2
691 Clostridium saccharoperbutylacetonicum N1-4_28HMT_29 GCA_000340885.1
676 Clostridium puniceum GCA_002006345.1
d6
788 Bacillus anthracis str. Ames GCA_000007845.1
788 Bacillus anthracis str. Sterne GCA_000008165.1
788 _5BBacillus thuringiensis_5D serovar konkukian str. 97-27 GCA_000008505.1
768 Bacillus cereus ATCC 14579 GCA_000007825.1
762 Bacillus thuringiensis YBT-1518 GCA_000497525.2
d7
756 Clostridium beijerinckii GCA_000833105.2
732 Clostridium saccharoperbutylacetonicum N1-4_28HMT_29 GCA_000340885.1
727 Clostridium saccharobutylicum DSM 13864 GCA_000473995.1
d8
694 Bifidobacterium adolescentis ATCC 15703 GCA_000010425.1
561 Bifidobacterium dentium JCM 1195 = DSM 20436 GCA_001042595.1
543 Bifidobacterium angulatum DSM 20098 = JCM 7096 GCA_001025155.1
d9
652 Enterococcus faecalis V583 GCA_000007785.1
605 Streptomyces cinnamoneus GCA_001885705.1
586 Enterococcus faecium DO GCA_000174395.2
da
651 Clostridium beijerinckii GCA_000833105.2
629 Clostridium saccharoperbutylacetonicum N1-4_28HMT_29 GCA_000340885.1
621 Clostridium saccharobutylicum DSM 13864 GCA_000473995.1
db
591 Rhodobacter sphaeroides 2.4.1 GCA_000012905.2
513 Rhodobacter sphaeroides ATCC 17025 GCA_000016405.1
375 Defluviimonas alba GCA_001620265.1
dc
667 Rhodobacter sphaeroides 2.4.1 GCA_000012905.2
575 Rhodobacter sphaeroides ATCC 17025 GCA_000016405.1
487 Defluviimonas alba GCA_001620265.1
dd
746 Rhodobacter sphaeroides 2.4.1 GCA_000012905.2
666 Rhodobacter sphaeroides ATCC 17025 GCA_000016405.1
591 Gemmobacter aquatilis GCA_900110025.1
de
624 Deinococcus radiodurans R1 GCA_000008565.1
290 Deinococcus deserti VCD115 GCA_000020685.1
289 Deinococcus soli Cha et al. 2016 GCA_001007995.1
df
941 Lactobacillus gasseri ATCC 33323 = JCM 1131 GCA_000014425.1
837 Lactobacillus hominis DSM 23910 = CRBIP 24.179 GCA_000296835.1
647 Lactobacillus iners DSM 13335 GCA_000160875.1
dg
935 Bacillus anthracis str. Ames GCA_000007845.1
935 Bacillus anthracis str. Sterne GCA_000008165.1
935 Bacillus cereus ATCC 14579 GCA_000007825.1
935 _5BBacillus thuringiensis_5D serovar konkukian str. 97-27 GCA_000008505.1
904 Bacillus mycoides GCA_000832605.1
904 Bacillus thuringiensis YBT-1518 GCA_000497525.2
887 Bacillus pseudomycoides DSM 12442 GCA_000161455.1
dh
603 Enterococcus faecalis V583 GCA_000007785.1
556 Streptomyces cinnamoneus GCA_001885705.1
547 Enterococcus asini ATCC 700915 GCA_000407365.1
di
411 Deinococcus radiodurans R1 GCA_000008565.1
273 Deinococcus gobiensis I-0 GCA_000252445.1
266 Deinococcus frigens DSM 12807 GCA_000701425.1
dj
407 Clostridium beijerinckii GCA_000833105.2
389 Clostridium puniceum GCA_002006345.1
367 Clostridium saccharoperbutylacetonicum N1-4_28HMT_29 GCA_000340885.1
dk
697 Deinococcus radiodurans R1 GCA_000008565.1
486 Deinococcus deserti VCD115 GCA_000020685.1
466 Deinococcus soli Cha et al. 2016 GCA_001007995.1

dl
823 Staphylococcus epidermidis ATCC 12228 GCA_000007645.1
776 Staphylococcus capitis subsp. capitis GCA_001028645.1
722 Staphylococcus warneri SG1 GCA_000332735.1
dm
621 Bacillus anthracis str. Ames GCA_000007845.1
621 Bacillus anthracis str. Sterne GCA_000008165.1
620 _5BBacillus thuringiensis_5D serovar konkukian str. 97-27 GCA_000008505.1
601 Bacillus cereus ATCC 14579 GCA_000007825.1
dn
249 Bifidobacterium adolescentis ATCC 15703 GCA_000010425.1
201 Bifidobacterium dentium JCM 1195 = DSM 20436 GCA_001042595.1
188 Bifidobacterium callitrichos DSM 23973 GCA_000741175.1
do
312 Clostridium beijerinckii GCA_000833105.2
311 Clostridium saccharoperbutylacetonicum N1-4_28HMT_29 GCA_000340885.1
305 Clostridium puniceum GCA_002006345.1
dp
445 Bifidobacterium adolescentis ATCC 15703 GCA_000010425.1
355 Bifidobacterium dentium JCM 1195 = DSM 20436 GCA_001042595.1
314 Bifidobacterium reuteri DSM 23975 GCA_000741695.1
dq
600 Clostridium saccharoperbutylacetonicum N1-4_28HMT_29 GCA_000340885.1
591 Clostridium beijerinckii GCA_000833105.2
579 Clostridium puniceum GCA_002006345.1
dr
673 Rhodobacter sphaeroides 2.4.1 GCA_000012905.2
614 Rhodobacter sphaeroides ATCC 17025 GCA_000016405.1
518 Gemmobacter megaterium GCA_900156815.1
ds
633 Rhodobacter sphaeroides 2.4.1 GCA_000012905.2
579 Rhodobacter sphaeroides ATCC 17025 GCA_000016405.1
473 Thioclava atlantica GCA_000737065.1
dt
647 Bifidobacterium adolescentis ATCC 15703 GCA_000010425.1
522 Bifidobacterium angulatum DSM 20098 = JCM 7096 GCA_001025155.1
521 Bifidobacterium dentium JCM 1195 = DSM 20436 GCA_001042595.1
du
632 Streptococcus mutans UA159 GCA_000007465.2
465 Streptococcus macacae NCTC 11558 GCA_000187995.3
460 Streptococcus ratti FA-1 = DSM 20564 GCA_000286075.1
dv
589 Staphylococcus capitis subsp. capitis GCA_001028645.1
587 Staphylococcus epidermidis ATCC 12228 GCA_000007645.1
579 Megasphaera cerevisiae DSM 20462 GCA_001045675.1
579 Staphylococcus warneri SG1 GCA_000332735.1
dw
471 Escherichia coli O157_3AH7 str. Sakai GCA_000008865.1
471 Escherichia coli str. K-12 substr. MG1655 GCA_000005845.2
471 Shigella flexneri 2a str. 301 GCA_000006925.2
469 Escherichia coli O83_3AH1 str. NRG 857C GCA_000183345.1
469 Escherichia coli UMN026 GCA_000026325.2
456 Shigella dysenteriae Sd197 GCA_000012005.1
dx
675 Escherichia coli IAI39 GCA_000026345.1
675 Escherichia coli O157_3AH7 str. Sakai GCA_000008865.1
675 Escherichia coli UMN026 GCA_000026325.2
675 Escherichia coli str. K-12 substr. MG1655 GCA_000005845.2
670 Escherichia coli O83_3AH1 str. NRG 857C GCA_000183345.1
662 Shigella flexneri 2a str. 301 GCA_000006925.2
dy
640 Staphylococcus epidermidis ATCC 12228 GCA_000007645.1
586 Staphylococcus capitis subsp. capitis GCA_001028645.1
546 Staphylococcus haemolyticus JCSC1435 GCA_000009865.1
dz
878 Rhodobacter sphaeroides 2.4.1 GCA_000012905.2
761 Rhodobacter sphaeroides ATCC 17025 GCA_000016405.1
625 Gemmobacter aquatilis GCA_900110025.1
dA
672 Lactobacillus gasseri ATCC 33323 = JCM 1131 GCA_000014425.1
582 Lactobacillus hominis DSM 23910 = CRBIP 24.179 GCA_000296835.1
420 Lactobacillus hamsteri DSM 5661 = JCM 6256 GCA_000615445.1
dB
648 Bifidobacterium adolescentis ATCC 15703 GCA_000010425.1
550 Bifidobacterium dentium JCM 1195 = DSM 20436 GCA_001042595.1
513 Bifidobacterium angulatum DSM 20098 = JCM 7096 GCA_001025155.1

dC
660 Clostridium beijerinckii GCA_000833105.2
634 Clostridium saccharoperbutylacetonicum N1-4_28HMT_29 GCA_000340885.1
600 Clostridium butyricum GCA_001456065.2
dD
817 Bifidobacterium adolescentis ATCC 15703 GCA_000010425.1
686 Bifidobacterium dentium JCM 1195 = DSM 20436 GCA_001042595.1
646 Bifidobacterium stellenboschense GCA_000741785.1
dE
433 Deinococcus radiodurans R1 GCA_000008565.1
228 Deinococcus gobiensis I-0 GCA_000252445.1
218 Deinococcus murrayi DSM 11303 GCA_000482805.1
dF
709 Clostridium beijerinckii GCA_000833105.2
636 Clostridium puniceum GCA_002006345.1
635 Clostridium saccharoperbutylacetonicum N1-4_28HMT_29 GCA_000340885.1
dG
844 Staphylococcus epidermidis ATCC 12228 GCA_000007645.1
790 Staphylococcus capitis subsp. capitis GCA_001028645.1
743 Staphylococcus aureus subsp. aureus NCTC 8325 GCA_000013425.1
dH
799 Streptococcus mutans UA159 GCA_000007465.2
605 Streptococcus ratti FA-1 = DSM 20564 GCA_000286075.1
568 Streptococcus gordonii str. Challis substr. CH1 GCA_000017005.1
dI
729 Clostridium beijerinckii GCA_000833105.2
682 Clostridium saccharoperbutylacetonicum N1-4_28HMT_29 GCA_000340885.1
671 Clostridium saccharobutylicum DSM 13864 GCA_000473995.1
dJ
610 Clostridium beijerinckii GCA_000833105.2
573 Clostridium saccharoperbutylacetonicum N1-4_28HMT_29 GCA_000340885.1
571 Clostridium saccharobutylicum DSM 13864 GCA_000473995.1
dK
801 Rhodobacter sphaeroides 2.4.1 GCA_000012905.2
730 Rhodobacter sphaeroides ATCC 17025 GCA_000016405.1
664 Pseudorhodobacter psychrotolerans GCA_001294535.1
dL
809 Lactobacillus gasseri ATCC 33323 = JCM 1131 GCA_000014425.1
713 Lactobacillus hominis DSM 23910 = CRBIP 24.179 GCA_000296835.1
566 Lactobacillus iners DSM 13335 GCA_000160875.1
dM
503 Clostridium beijerinckii GCA_000833105.2
501 Clostridium puniceum GCA_002006345.1
501 Clostridium saccharoperbutylacetonicum N1-4_28HMT_29 GCA_000340885.1
486 Clostridium saccharobutylicum DSM 13864 GCA_000473995.1
dN
776 Clostridium beijerinckii GCA_000833105.2
734 Clostridium saccharobutylicum DSM 13864 GCA_000473995.1
733 Clostridium saccharoperbutylacetonicum N1-4_28HMT_29 GCA_000340885.1
dO
768 Deinococcus radiodurans R1 GCA_000008565.1
431 Deinococcus deserti VCD115 GCA_000020685.1
427 Deinococcus gobiensis I-0 GCA_000252445.1
dP
674 Bacillus anthracis str. Ames GCA_000007845.1
674 Bacillus anthracis str. Sterne GCA_000008165.1
674 _5BBacillus thuringiensis_5D serovar konkukian str. 97-27 GCA_000008505.1
665 Bacillus cereus ATCC 14579 GCA_000007825.1
661 Bacillus pseudomycoides DSM 12442 GCA_000161455.1
dQ
942 Enterococcus faecalis V583 GCA_000007785.1
895 Streptomyces cinnamoneus GCA_001885705.1
808 Enterococcus rivorum GCA_001742285.1
dR
858 Clostridium beijerinckii GCA_000833105.2
824 Clostridium saccharoperbutylacetonicum N1-4_28HMT_29 GCA_000340885.1
762 Clostridium butyricum GCA_001456065.2
762 Clostridium puniceum GCA_002006345.1
dS
756 Bacillus cereus ATCC 14579 GCA_000007825.1
754 Bacillus anthracis str. Ames GCA_000007845.1
754 Bacillus anthracis str. Sterne GCA_000008165.1
754 _5BBacillus thuringiensis_5D serovar konkukian str. 97-27 GCA_000008505.1
746 Bacillus mycoides GCA_000832605.1
dT
807 Enterococcus faecalis V583 GCA_000007785.1

764 Streptomyces cinnamoneus GCA_001885705.1
705 Enterococcus faecium DO GCA_000174395.2
dU
421 Bifidobacterium adolescentis ATCC 15703 GCA_000010425.1
295 Bifidobacterium dentium JCM 1195 = DSM 20436 GCA_001042595.1
275 Bifidobacterium asteroides PRL2011 GCA_000304215.1
dV
698 Clostridium beijerinckii GCA_000833105.2
664 Clostridium saccharobutylicum DSM 13864 GCA_000473995.1
657 Clostridium saccharoperbutylacetonicum N1-4_28HMT_29 GCA_000340885.1
dW
602 Clostridium beijerinckii GCA_000833105.2
587 Clostridium saccharobutylicum DSM 13864 GCA_000473995.1
587 Clostridium saccharoperbutylacetonicum N1-4_28HMT_29 GCA_000340885.1
555 Clostridium chromiireducens GCA_002029255.1
dX
577 Lactobacillus gasseri ATCC 33323 = JCM 1131 GCA_000014425.1
540 Lactobacillus hominis DSM 23910 = CRBIP 24.179 GCA_000296835.1
420 Lactobacillus iners DSM 13335 GCA_000160875.1
dY
705 Clostridium beijerinckii GCA_000833105.2
682 Clostridium saccharoperbutylacetonicum N1-4_28HMT_29 GCA_000340885.1
652 Clostridium puniceum GCA_002006345.1
dZ
368 Staphylococcus epidermidis ATCC 12228 GCA_000007645.1
353 Staphylococcus condimenti GCA_001618885.1
340 Staphylococcus lugdunensis HKU09-01 GCA_000025085.1
e0
724 Clostridium beijerinckii GCA_000833105.2
707 Clostridium saccharoperbutylacetonicum N1-4_28HMT_29 GCA_000340885.1
661 Clostridium butyricum GCA_001456065.2
e1
752 Enterococcus faecalis V583 GCA_000007785.1
706 Streptomyces cinnamoneus GCA_001885705.1
642 Enterococcus faecium DO GCA_000174395.2
e2
550 Bifidobacterium adolescentis ATCC 15703 GCA_000010425.1
475 Bifidobacterium dentium JCM 1195 = DSM 20436 GCA_001042595.1
448 Bifidobacterium bifidum PRL2010 GCA_000165905.1
e3
713 Enterococcus faecalis V583 GCA_000007785.1
667 Streptomyces cinnamoneus GCA_001885705.1
617 Enterococcus asini ATCC 700915 GCA_000407365.1
e4
685 Clostridium beijerinckii GCA_000833105.2
645 Clostridium saccharoperbutylacetonicum N1-4_28HMT_29 GCA_000340885.1
640 Clostridium saccharobutylicum DSM 13864 GCA_000473995.1
e5
546 Streptococcus mutans UA159 GCA_000007465.2
347 Streptococcus equinus GCA_000964315.1
347 Streptococcus gordonii str. Challis substr. CH1 GCA_000017005.1
346 Streptococcus gallolyticus subsp. gallolyticus DSM 16831 GCA_002000985.1
e6
739 Staphylococcus epidermidis ATCC 12228 GCA_000007645.1
709 Staphylococcus capitis subsp. capitis GCA_001028645.1
678 Staphylococcus hominis subsp. hominis C80 GCA_000183685.1
e7
570 Enterococcus faecalis V583 GCA_000007785.1
524 Enterococcus thailandicus GCA_001652875.1
523 Enterococcus faecium DO GCA_000174395.2
523 Streptomyces cinnamoneus GCA_001885705.1
e8
230 Streptococcus mutans UA159 GCA_000007465.2
176 Streptococcus ferus DSM 20646 GCA_000372425.1
172 Streptococcus urinalis 2285-97 GCA_000188055.3
e9
876 Staphylococcus epidermidis ATCC 12228 GCA_000007645.1
828 Staphylococcus capitis subsp. capitis GCA_001028645.1
806 Staphylococcus aureus subsp. aureus NCTC 8325 GCA_000013425.1
ea
669 Deinococcus radiodurans R1 GCA_000008565.1
387 Deinococcus deserti VCD115 GCA_000020685.1
369 Deinococcus gobiensis I-0 GCA_000252445.1
eb
702 Enterococcus faecalis V583 GCA_000007785.1
656 Streptomyces cinnamoneus GCA_001885705.1

612 Enterococcus faecium DO GCA_000174395.2
ec
617 Clostridium beijerinckii GCA_000833105.2
609 Clostridium saccharoperbutylacetonicum N1-4_28HMT_29 GCA_000340885.1
600 Clostridium saccharobutylicum DSM 13864 GCA_000473995.1
ed
763 Clostridium beijerinckii GCA_000833105.2
738 Clostridium saccharoperbutylacetonicum N1-4_28HMT_29 GCA_000340885.1
696 Clostridium saccharobutylicum DSM 13864 GCA_000473995.1
ee
942 Lactobacillus gasseri ATCC 33323 = JCM 1131 GCA_000014425.1
852 Lactobacillus hominis DSM 23910 = CRBIP 24.179 GCA_000296835.1
639 Lactobacillus hamsteri DSM 5661 = JCM 6256 GCA_000615445.1
639 Lactobacillus iners DSM 13335 GCA_000160875.1
ef
706 Escherichia coli O157_3AH7 str. Sakai GCA_000008865.1
706 Escherichia coli UMN026 GCA_000026325.2
706 Escherichia coli str. K-12 substr. MG1655 GCA_000005845.2
702 Escherichia coli IAI39 GCA_000026345.1
702 Shigella flexneri 2a str. 301 GCA_000006925.2
701 Escherichia coli O83_3AH1 str. NRG 857C GCA_000183345.1
eg
402 Rhodobacter sphaeroides 2.4.1 GCA_000012905.2
342 Rhodobacter sphaeroides ATCC 17025 GCA_000016405.1
295 Defluviimonas alba GCA_001620265.1
eh
682 Deinococcus radiodurans R1 GCA_000008565.1
386 Deinococcus deserti VCD115 GCA_000020685.1
356 Deinococcus marmoris DSM 12784 GCA_000701405.1
ei
683 Streptococcus mutans UA159 GCA_000007465.2
479 Streptococcus ratti FA-1 = DSM 20564 GCA_000286075.1
449 Streptococcus sobrinus DSM 20742 = ATCC 33478 GCA_000686605.1
ej
643 Clostridium beijerinckii GCA_000833105.2
618 Clostridium saccharoperbutylacetonicum N1-4_28HMT_29 GCA_000340885.1
605 Clostridium saccharobutylicum DSM 13864 GCA_000473995.1
ek
420 Streptococcus mutans UA159 GCA_000007465.2
291 Streptococcus gordonii str. Challis substr. CH1 GCA_000017005.1
283 Streptococcus ratti FA-1 = DSM 20564 GCA_000286075.1
el
797 Staphylococcus epidermidis ATCC 12228 GCA_000007645.1
734 Staphylococcus capitis subsp. capitis GCA_001028645.1
711 Staphylococcus aureus subsp. aureus NCTC 8325 GCA_000013425.1
em
673 Clostridium beijerinckii GCA_000833105.2
643 Clostridium saccharoperbutylacetonicum N1-4_28HMT_29 GCA_000340885.1
639 Clostridium puniceum GCA_002006345.1
en
586 Clostridium beijerinckii GCA_000833105.2
551 Clostridium puniceum GCA_002006345.1
551 Clostridium saccharoperbutylacetonicum N1-4_28HMT_29 GCA_000340885.1
529 Clostridium saccharobutylicum DSM 13864 GCA_000473995.1
eo
907 Enterococcus faecalis V583 GCA_000007785.1
860 Streptomyces cinnamoneus GCA_001885705.1
836 Enterococcus rivorum GCA_001742285.1
ep
689 Staphylococcus epidermidis ATCC 12228 GCA_000007645.1
666 Staphylococcus capitis subsp. capitis GCA_001028645.1
629 Staphylococcus warneri SG1 GCA_000332735.1
eq
695 Staphylococcus epidermidis ATCC 12228 GCA_000007645.1
665 Staphylococcus capitis subsp. capitis GCA_001028645.1
603 Staphylococcus lugdunensis HKU09-01 GCA_000025085.1
er
608 Staphylococcus capitis subsp. capitis GCA_001028645.1
608 Staphylococcus epidermidis ATCC 12228 GCA_000007645.1
548 Staphylococcus haemolyticus JCSC1435 GCA_000009865.1
545 Staphylococcus aureus subsp. aureus NCTC 8325 GCA_000013425.1
es
668 Clostridium beijerinckii GCA_000833105.2
658 Clostridium saccharoperbutylacetonicum N1-4_28HMT_29 GCA_000340885.1
635 Clostridium puniceum GCA_002006345.1
et

704 Deinococcus radiodurans R1 GCA_000008565.1
444 Deinococcus deserti VCD115 GCA_000020685.1
404 Deinococcus hopiensis KR-140 GCA_900176165.1
eu
759 Lactobacillus gasseri ATCC 33323 = JCM 1131 GCA_000014425.1
662 Lactobacillus hominis DSM 23910 = CRBIP 24.179 GCA_000296835.1
478 Lactobacillus iners DSM 13335 GCA_000160875.1
ev
656 Lactobacillus gasseri ATCC 33323 = JCM 1131 GCA_000014425.1
580 Lactobacillus hominis DSM 23910 = CRBIP 24.179 GCA_000296835.1
454 Lactobacillus hamsteri DSM 5661 = JCM 6256 GCA_000615445.1
ew
294 Escherichia coli O104_3AH4 str. 2011C-3493 GCA_000299455.1
294 Escherichia coli str. K-12 substr. MG1655 GCA_000005845.2
294 Shigella flexneri 2a str. 301 GCA_000006925.2
269 Escherichia coli O157_3AH7 str. Sakai GCA_000008865.1
269 Escherichia coli O83_3AH1 str. NRG 857C GCA_000183345.1
269 Escherichia coli UMN026 GCA_000026325.2
233 Pluralibacter gergoviae GCA_000757785.1
ex
890 Staphylococcus epidermidis ATCC 12228 GCA_000007645.1
872 Staphylococcus capitis subsp. capitis GCA_001028645.1
829 Staphylococcus haemolyticus JCSC1435 GCA_000009865.1
ey
660 Deinococcus radiodurans R1 GCA_000008565.1
420 Deinococcus deserti VCD115 GCA_000020685.1
382 Deinococcus soli Cha et al. 2016 GCA_001007995.1
ez
567 Rhodobacter sphaeroides 2.4.1 GCA_000012905.2
535 Rhodobacter sphaeroides ATCC 17025 GCA_000016405.1
441 Defluviimonas alba GCA_001620265.1
eA
813 Rhodobacter sphaeroides 2.4.1 GCA_000012905.2
683 Rhodobacter sphaeroides ATCC 17025 GCA_000016405.1
548 Gemmobacter aquatilis GCA_900110025.1
eB
736 Lactobacillus gasseri ATCC 33323 = JCM 1131 GCA_000014425.1
652 Lactobacillus hominis DSM 23910 = CRBIP 24.179 GCA_000296835.1
462 Lactobacillus iners DSM 13335 GCA_000160875.1
eC
637 Bacillus anthracis str. Ames GCA_000007845.1
637 Bacillus anthracis str. Sterne GCA_000008165.1
637 Bacillus cereus ATCC 14579 GCA_000007825.1
637 _5BBacillus thuringiensis_5D serovar konkukian str. 97-27 GCA_000008505.1
622 Bacillus thuringiensis YBT-1518 GCA_000497525.2
605 Bacillus pseudomycoides DSM 12442 GCA_000161455.1
eD
785 Escherichia coli IAI39 GCA_000026345.1
785 Escherichia coli O83_3AH1 str. NRG 857C GCA_000183345.1
770 Escherichia coli O157_3AH7 str. Sakai GCA_000008865.1
770 Escherichia coli UMN026 GCA_000026325.2
770 Escherichia coli str. K-12 substr. MG1655 GCA_000005845.2
754 Shigella dysenteriae Sd197 GCA_000012005.1
eE
587 Rhodobacter sphaeroides 2.4.1 GCA_000012905.2
564 Rhodobacter sphaeroides ATCC 17025 GCA_000016405.1
485 Gemmobacter megaterium GCA_900156815.1
eF
419 Clostridium beijerinckii GCA_000833105.2
398 Clostridium saccharobutylicum DSM 13864 GCA_000473995.1
385 Clostridium saccharoperbutylacetonicum N1-4_28HMT_29 GCA_000340885.1
eG
734 Deinococcus radiodurans R1 GCA_000008565.1
424 Deinococcus deserti VCD115 GCA_000020685.1
420 Deinococcus gobiensis I-0 GCA_000252445.1
eH
703 Clostridium beijerinckii GCA_000833105.2
676 Clostridium saccharoperbutylacetonicum N1-4_28HMT_29 GCA_000340885.1
654 Clostridium saccharobutylicum DSM 13864 GCA_000473995.1
eI
540 Rhodobacter sphaeroides 2.4.1 GCA_000012905.2
459 Rhodobacter sphaeroides ATCC 17025 GCA_000016405.1
393 Defluviimonas alba GCA_001620265.1
eJ
474 Lactobacillus gasseri ATCC 33323 = JCM 1131 GCA_000014425.1
463 Lactobacillus hominis DSM 23910 = CRBIP 24.179 GCA_000296835.1

384 Lactobacillus iners DSM 13335 GCA_000160875.1
eK
480 Deinococcus radiodurans R1 GCA_000008565.1
322 Deinococcus proteolyticus MRP GCA_000190555.1
311 Deinococcus soli Cha et al. 2016 GCA_001007995.1
eL
485 Bifidobacterium adolescentis ATCC 15703 GCA_000010425.1
381 Bifidobacterium angulatum DSM 20098 = JCM 7096 GCA_001025155.1
378 Bifidobacterium dentium JCM 1195 = DSM 20436 GCA_001042595.1
eM
821 Clostridium beijerinckii GCA_000833105.2
790 Clostridium saccharoperbutylacetonicum N1-4_28HMT_29 GCA_000340885.1
753 Clostridium puniceum GCA_002006345.1
eN
899 Staphylococcus epidermidis ATCC 12228 GCA_000007645.1
844 Staphylococcus capitis subsp. capitis GCA_001028645.1
797 Megasphaera cerevisiae DSM 20462 GCA_001045675.1
797 Staphylococcus warneri SG1 GCA_000332735.1
eO
698 Clostridium beijerinckii GCA_000833105.2
655 Clostridium saccharoperbutylacetonicum N1-4_28HMT_29 GCA_000340885.1
644 Clostridium puniceum GCA_002006345.1
eP
703 Clostridium beijerinckii GCA_000833105.2
678 Clostridium saccharoperbutylacetonicum N1-4_28HMT_29 GCA_000340885.1
677 Clostridium saccharobutylicum DSM 13864 GCA_000473995.1
eQ
517 Rhodobacter sphaeroides 2.4.1 GCA_000012905.2
490 Rhodobacter sphaeroides ATCC 17025 GCA_000016405.1
441 Pseudorhodobacter ferrugineus DSM 5888 GCA_000420745.1
eR
879 Lactobacillus gasseri ATCC 33323 = JCM 1131 GCA_000014425.1
808 Lactobacillus hominis DSM 23910 = CRBIP 24.179 GCA_000296835.1
605 Lactobacillus hamsteri DSM 5661 = JCM 6256 GCA_000615445.1
eS
763 Lactobacillus gasseri ATCC 33323 = JCM 1131 GCA_000014425.1
696 Lactobacillus hominis DSM 23910 = CRBIP 24.179 GCA_000296835.1
508 Lactobacillus iners DSM 13335 GCA_000160875.1
eT
466 Streptococcus mutans UA159 GCA_000007465.2
353 Streptococcus sobrinus DSM 20742 = ATCC 33478 GCA_000686605.1
340 Streptococcus ratti FA-1 = DSM 20564 GCA_000286075.1
eU
613 Lactobacillus gasseri ATCC 33323 = JCM 1131 GCA_000014425.1
528 Lactobacillus hominis DSM 23910 = CRBIP 24.179 GCA_000296835.1
400 Lactobacillus hamsteri DSM 5661 = JCM 6256 GCA_000615445.1
eV
554 Bifidobacterium adolescentis ATCC 15703 GCA_000010425.1
455 Bifidobacterium dentium JCM 1195 = DSM 20436 GCA_001042595.1
435 Bifidobacterium angulatum DSM 20098 = JCM 7096 GCA_001025155.1
eW
541 Bifidobacterium adolescentis ATCC 15703 GCA_000010425.1
387 Bifidobacterium angulatum DSM 20098 = JCM 7096 GCA_001025155.1
385 Bifidobacterium callitrichos DSM 23973 GCA_000741175.1
eX
617 Streptococcus mutans UA159 GCA_000007465.2
413 Streptococcus ratti FA-1 = DSM 20564 GCA_000286075.1
408 Streptococcus ferus DSM 20646 GCA_000372425.1
eY
643 Streptococcus mutans UA159 GCA_000007465.2
547 Streptococcus ratti FA-1 = DSM 20564 GCA_000286075.1
499 Streptococcus gallolyticus subsp. gallolyticus DSM 16831 GCA_002000985.1
eZ
596 Lactobacillus gasseri ATCC 33323 = JCM 1131 GCA_000014425.1
523 Lactobacillus hominis DSM 23910 = CRBIP 24.179 GCA_000296835.1
408 Lactobacillus iners DSM 13335 GCA_000160875.1
f0
833 Lactobacillus gasseri ATCC 33323 = JCM 1131 GCA_000014425.1
765 Lactobacillus hominis DSM 23910 = CRBIP 24.179 GCA_000296835.1
558 Lactobacillus iners DSM 13335 GCA_000160875.1
558 Lactobacillus psittaci DSM 15354 GCA_000425905.1
f1
849 Staphylococcus epidermidis ATCC 12228 GCA_000007645.1
818 Staphylococcus capitis subsp. capitis GCA_001028645.1
795 Staphylococcus lugdunensis HKU09-01 GCA_000025085.1
f2

786 Enterococcus faecalis V583 GCA_000007785.1
739 Streptomyces cinnamoneus GCA_001885705.1
682 Enterococcus rivorum GCA_001742285.1
f3
760 Clostridium beijerinckii GCA_000833105.2
708 Clostridium saccharoperbutylacetonicum N1-4_28HMT_29 GCA_000340885.1
671 Clostridium puniceum GCA_002006345.1
f4
610 Escherichia coli str. K-12 substr. MG1655 GCA_000005845.2
599 Shigella flexneri 2a str. 301 GCA_000006925.2
590 Escherichia coli IAI39 GCA_000026345.1
f5
439 Clostridium beijerinckii GCA_000833105.2
375 Clostridium puniceum GCA_002006345.1
375 Clostridium saccharoperbutylacetonicum N1-4_28HMT_29 GCA_000340885.1
364 Clostridium saccharobutylicum DSM 13864 GCA_000473995.1
f6
657 Escherichia coli O157_3AH7 str. Sakai GCA_000008865.1
657 Escherichia coli str. K-12 substr. MG1655 GCA_000005845.2
656 Escherichia coli UMN026 GCA_000026325.2
642 Shigella flexneri 2a str. 301 GCA_000006925.2
f7
665 Clostridium beijerinckii GCA_000833105.2
640 Clostridium saccharoperbutylacetonicum N1-4_28HMT_29 GCA_000340885.1
636 Clostridium chromiireducens GCA_002029255.1
f8
936 Streptococcus mutans UA159 GCA_000007465.2
673 Streptococcus gordonii str. Challis substr. CH1 GCA_000017005.1
655 Streptococcus ratti FA-1 = DSM 20564 GCA_000286075.1
f9
823 Escherichia coli str. K-12 substr. MG1655 GCA_000005845.2
802 Escherichia coli O104_3AH4 str. 2011C-3493 GCA_000299455.1
791 Shigella flexneri 2a str. 301 GCA_000006925.2
fa
518 Deinococcus radiodurans R1 GCA_000008565.1
329 Deinococcus deserti VCD115 GCA_000020685.1
325 Deinococcus hopiensis KR-140 GCA_900176165.1
fb
804 Clostridium beijerinckii GCA_000833105.2
772 Clostridium saccharoperbutylacetonicum N1-4_28HMT_29 GCA_000340885.1
714 Clostridium puniceum GCA_002006345.1
fc
705 Bacillus anthracis str. Ames GCA_000007845.1
705 Bacillus anthracis str. Sterne GCA_000008165.1
705 _5BBacillus thuringiensis_5D serovar konkukian str. 97-27 GCA_000008505.1
694 Bacillus pseudomycoides DSM 12442 GCA_000161455.1
693 Bacillus cereus ATCC 14579 GCA_000007825.1
fd
614 Lactobacillus gasseri ATCC 33323 = JCM 1131 GCA_000014425.1
571 Lactobacillus hominis DSM 23910 = CRBIP 24.179 GCA_000296835.1
452 Lactobacillus iners DSM 13335 GCA_000160875.1
fe
469 Bacillus mycoides GCA_000832605.1
465 Bacillus cereus ATCC 14579 GCA_000007825.1
465 Bacillus thuringiensis YBT-1518 GCA_000497525.2
463 Bacillus anthracis str. Ames GCA_000007845.1
463 Bacillus anthracis str. Sterne GCA_000008165.1
463 _5BBacillus thuringiensis_5D serovar konkukian str. 97-27 GCA_000008505.1
ff
739 Bacillus anthracis str. Ames GCA_000007845.1
739 Bacillus anthracis str. Sterne GCA_000008165.1
739 Bacillus cereus ATCC 14579 GCA_000007825.1
739 Bacillus thuringiensis YBT-1518 GCA_000497525.2
739 _5BBacillus thuringiensis_5D serovar konkukian str. 97-27 GCA_000008505.1
716 Bacillus mycoides GCA_000832605.1
715 Bacillus pseudomycoides DSM 12442 GCA_000161455.1
fg
739 Streptococcus mutans UA159 GCA_000007465.2
528 Streptococcus ratti FA-1 = DSM 20564 GCA_000286075.1
479 Streptococcus gordonii str. Challis substr. CH1 GCA_000017005.1
fh
680 Deinococcus radiodurans R1 GCA_000008565.1
403 Deinococcus deserti VCD115 GCA_000020685.1
393 Deinococcus hopiensis KR-140 GCA_900176165.1
fi
689 Lactobacillus gasseri ATCC 33323 = JCM 1131 GCA_000014425.1

603 Lactobacillus hominis DSM 23910 = CRBIP 24.179 GCA_000296835.1
426 Lactobacillus iners DSM 13335 GCA_000160875.1
fj
563 Rhodobacter sphaeroides 2.4.1 GCA_000012905.2
511 Rhodobacter sphaeroides ATCC 17025 GCA_000016405.1
394 Defluviimonas alba GCA_001620265.1
394 Pseudorhodobacter wandonensis GCA_001202035.1
fk
693 Rhodobacter sphaeroides 2.4.1 GCA_000012905.2
620 Rhodobacter sphaeroides ATCC 17025 GCA_000016405.1
448 Defluviimonas alba GCA_001620265.1
fl
841 Enterococcus faecalis V583 GCA_000007785.1
794 Streptomyces cinnamoneus GCA_001885705.1
725 Enterococcus asini ATCC 700915 GCA_000407365.1
fm
540 Deinococcus radiodurans R1 GCA_000008565.1
348 Deinococcus deserti VCD115 GCA_000020685.1
317 Deinococcus soli Cha et al. 2016 GCA_001007995.1
fn
758 Staphylococcus epidermidis ATCC 12228 GCA_000007645.1
724 Staphylococcus capitis subsp. capitis GCA_001028645.1
677 Staphylococcus haemolyticus JCSC1435 GCA_000009865.1
fo
815 Staphylococcus epidermidis ATCC 12228 GCA_000007645.1
765 Staphylococcus capitis subsp. capitis GCA_001028645.1
702 Staphylococcus haemolyticus JCSC1435 GCA_000009865.1
fp
625 Streptococcus mutans UA159 GCA_000007465.2
469 Streptococcus pseudoporcinus LQ 940-04 GCA_000188035.3
467 Streptococcus iniae GCA_000831485.1
fq
657 Rhodobacter sphaeroides 2.4.1 GCA_000012905.2
588 Rhodobacter sphaeroides ATCC 17025 GCA_000016405.1
548 Gemmobacter aquatilis GCA_900110025.1
fr
528 Clostridium beijerinckii GCA_000833105.2
511 Clostridium saccharoperbutylacetonicum N1-4_28HMT_29 GCA_000340885.1
475 Clostridium saccharobutylicum DSM 13864 GCA_000473995.1
fs
825 Deinococcus radiodurans R1 GCA_000008565.1
535 Deinococcus deserti VCD115 GCA_000020685.1
505 Deinococcus gobiensis I-0 GCA_000252445.1
ft
691 Streptococcus mutans UA159 GCA_000007465.2
541 Streptococcus gordonii str. Challis substr. CH1 GCA_000017005.1
525 Streptococcus ratti FA-1 = DSM 20564 GCA_000286075.1
fu
527 Clostridium saccharoperbutylacetonicum N1-4_28HMT_29 GCA_000340885.1
523 Clostridium beijerinckii GCA_000833105.2
502 Clostridium puniceum GCA_002006345.1
fv
576 Staphylococcus epidermidis ATCC 12228 GCA_000007645.1
561 Staphylococcus capitis subsp. capitis GCA_001028645.1
546 Staphylococcus condimenti GCA_001618885.1
fw
962 Enterococcus faecalis V583 GCA_000007785.1
915 Streptomyces cinnamoneus GCA_001885705.1
853 Enterococcus massiliensis GCA_001050095.1
fx
736 Lactobacillus gasseri ATCC 33323 = JCM 1131 GCA_000014425.1
672 Lactobacillus hominis DSM 23910 = CRBIP 24.179 GCA_000296835.1
462 Lactobacillus iners DSM 13335 GCA_000160875.1
fy
862 Streptococcus mutans UA159 GCA_000007465.2
582 Streptococcus ratti FA-1 = DSM 20564 GCA_000286075.1
566 Streptococcus halotolerans GCA_001598035.1
fz
925 Staphylococcus epidermidis ATCC 12228 GCA_000007645.1
842 Staphylococcus capitis subsp. capitis GCA_001028645.1
771 Staphylococcus lugdunensis HKU09-01 GCA_000025085.1
fA
443 Bifidobacterium adolescentis ATCC 15703 GCA_000010425.1
385 Bifidobacterium dentium JCM 1195 = DSM 20436 GCA_001042595.1
355 Bifidobacterium angulatum DSM 20098 = JCM 7096 GCA_001025155.1
fB

795 Lactobacillus gasseri ATCC 33323 = JCM 1131 GCA_000014425.1
712 Lactobacillus hominis DSM 23910 = CRBIP 24.179 GCA_000296835.1
522 Lactobacillus iners DSM 13335 GCA_000160875.1
fC
443 Staphylococcus epidermidis ATCC 12228 GCA_000007645.1
409 Staphylococcus capitis subsp. capitis GCA_001028645.1
355 Staphylococcus warneri SG1 GCA_000332735.1
fD
715 Bacillus anthracis str. Ames GCA_000007845.1
715 Bacillus anthracis str. Sterne GCA_000008165.1
715 Bacillus cereus ATCC 14579 GCA_000007825.1
715 Bacillus thuringiensis YBT-1518 GCA_000497525.2
715 _5BBacillus thuringiensis_5D serovar konkukian str. 97-27 GCA_000008505.1
699 Bacillus pseudomycoides DSM 12442 GCA_000161455.1
697 Bacillus mycoides GCA_000832605.1
fE
635 Streptococcus mutans UA159 GCA_000007465.2
444 Streptococcus ratti FA-1 = DSM 20564 GCA_000286075.1
422 Streptococcus ferus DSM 20646 GCA_000372425.1
422 Streptococcus gordonii str. Challis substr. CH1 GCA_000017005.1
fF
620 Rhodobacter sphaeroides 2.4.1 GCA_000012905.2
566 Rhodobacter sphaeroides ATCC 17025 GCA_000016405.1
527 Gemmobacter megaterium GCA_900156815.1
fG
858 Shigella flexneri 2a str. 301 GCA_000006925.2
856 Escherichia coli O83_3AH1 str. NRG 857C GCA_000183345.1
847 Escherichia coli O157_3AH7 str. Sakai GCA_000008865.1
847 Escherichia coli UMN026 GCA_000026325.2
847 Escherichia coli str. K-12 substr. MG1655 GCA_000005845.2
fH
583 Streptococcus mutans UA159 GCA_000007465.2
405 Streptococcus ratti FA-1 = DSM 20564 GCA_000286075.1
356 Streptococcus ferus DSM 20646 GCA_000372425.1
fI
747 Streptococcus mutans UA159 GCA_000007465.2
586 Streptococcus ferus DSM 20646 GCA_000372425.1
583 Streptococcus ratti FA-1 = DSM 20564 GCA_000286075.1
fJ
676 Enterococcus faecalis V583 GCA_000007785.1
629 Streptomyces cinnamoneus GCA_001885705.1
556 Enterococcus faecium DO GCA_000174395.2
556 Enterococcus rivorum GCA_001742285.1
fK
673 Deinococcus radiodurans R1 GCA_000008565.1
390 Deinococcus gobiensis I-0 GCA_000252445.1
377 Deinococcus deserti VCD115 GCA_000020685.1
fL
766 Bifidobacterium adolescentis ATCC 15703 GCA_000010425.1
658 Bifidobacterium dentium JCM 1195 = DSM 20436 GCA_001042595.1
645 Bifidobacterium angulatum DSM 20098 = JCM 7096 GCA_001025155.1
fM
581 Staphylococcus epidermidis ATCC 12228 GCA_000007645.1
559 Staphylococcus capitis subsp. capitis GCA_001028645.1
542 Staphylococcus haemolyticus JCSC1435 GCA_000009865.1
fN
499 Clostridium beijerinckii GCA_000833105.2
498 Clostridium saccharobutylicum DSM 13864 GCA_000473995.1
489 Clostridium puniceum GCA_002006345.1
489 Clostridium saccharoperbutylacetonicum N1-4_28HMT_29 GCA_000340885.1
fO
562 Clostridium beijerinckii GCA_000833105.2
544 Clostridium saccharoperbutylacetonicum N1-4_28HMT_29 GCA_000340885.1
534 Clostridium puniceum GCA_002006345.1
fP
901 Deinococcus radiodurans R1 GCA_000008565.1
555 Deinococcus deserti VCD115 GCA_000020685.1
513 Deinococcus murrayi DSM 11303 GCA_000482805.1
fQ
652 Enterococcus faecalis V583 GCA_000007785.1
620 Streptomyces cinnamoneus GCA_001885705.1
577 Enterococcus rivorum GCA_001742285.1
fR
771 Staphylococcus epidermidis ATCC 12228 GCA_000007645.1
748 Staphylococcus capitis subsp. capitis GCA_001028645.1
703 Staphylococcus aureus subsp. aureus NCTC 8325 GCA_000013425.1

fS
614 Enterococcus faecalis V583 GCA_000007785.1
567 Streptomyces cinnamoneus GCA_001885705.1
509 Enterococcus rivorum GCA_001742285.1
fT
712 Enterococcus faecalis V583 GCA_000007785.1
666 Streptomyces cinnamoneus GCA_001885705.1
641 Enterococcus dispar ATCC 51266 GCA_000406945.1
fU
473 Bifidobacterium adolescentis ATCC 15703 GCA_000010425.1
390 Bifidobacterium callitrichos DSM 23973 GCA_000741175.1
388 Bifidobacterium angulatum DSM 20098 = JCM 7096 GCA_001025155.1
fV
693 Rhodobacter sphaeroides 2.4.1 GCA_000012905.2
599 Rhodobacter sphaeroides ATCC 17025 GCA_000016405.1
507 Pseudorhodobacter psychrotolerans GCA_001294535.1
fW
846 Rhodobacter sphaeroides 2.4.1 GCA_000012905.2
757 Rhodobacter sphaeroides ATCC 17025 GCA_000016405.1
662 Defluviimonas alba GCA_001620265.1
fX
810 Streptococcus mutans UA159 GCA_000007465.2
625 Streptococcus ratti FA-1 = DSM 20564 GCA_000286075.1
621 Streptococcus ferus DSM 20646 GCA_000372425.1
fY
647 Enterococcus faecalis V583 GCA_000007785.1
600 Streptomyces cinnamoneus GCA_001885705.1
545 Enterococcus rivorum GCA_001742285.1
fZ
615 Rhodobacter sphaeroides 2.4.1 GCA_000012905.2
513 Rhodobacter sphaeroides ATCC 17025 GCA_000016405.1
428 Gemmobacter megaterium GCA_900156815.1
g0
723 Streptococcus mutans UA159 GCA_000007465.2
576 Streptococcus gordonii str. Challis substr. CH1 GCA_000017005.1
549 Streptococcus salivarius GCA_000785515.1
549 Streptococcus thermophilus JIM 8232 GCA_000253395.1
g1
668 Lactobacillus gasseri ATCC 33323 = JCM 1131 GCA_000014425.1
635 Lactobacillus hominis DSM 23910 = CRBIP 24.179 GCA_000296835.1
470 Lactobacillus iners DSM 13335 GCA_000160875.1
g2
494 Lactobacillus gasseri ATCC 33323 = JCM 1131 GCA_000014425.1
446 Lactobacillus hominis DSM 23910 = CRBIP 24.179 GCA_000296835.1
355 Staphylococcus epidermidis ATCC 12228 GCA_000007645.1
g3
513 Bifidobacterium adolescentis ATCC 15703 GCA_000010425.1
393 Bifidobacterium dentium JCM 1195 = DSM 20436 GCA_001042595.1
342 Bifidobacterium animalis subsp. lactis DSM 10140 GCA_000022965.1
342 Bifidobacterium thermophilum RBL67 GCA_000347695.1
g4
720 Rhodobacter sphaeroides 2.4.1 GCA_000012905.2
622 Rhodobacter sphaeroides ATCC 17025 GCA_000016405.1
494 Gemmobacter aquatilis GCA_900110025.1
g5
666 Clostridium beijerinckii GCA_000833105.2
646 Clostridium saccharoperbutylacetonicum N1-4_28HMT_29 GCA_000340885.1
640 Clostridium puniceum GCA_002006345.1
g6
566 Rhodobacter sphaeroides 2.4.1 GCA_000012905.2
545 Rhodobacter sphaeroides ATCC 17025 GCA_000016405.1
466 Pseudorhodobacter psychrotolerans GCA_001294535.1
g7
449 Staphylococcus epidermidis ATCC 12228 GCA_000007645.1
400 Staphylococcus capitis subsp. capitis GCA_001028645.1
358 Staphylococcus lutrae GCA_002101335.1
g8
796 Deinococcus radiodurans R1 GCA_000008565.1
439 Deinococcus gobiensis I-0 GCA_000252445.1
429 Deinococcus deserti VCD115 GCA_000020685.1
g9
528 Clostridium beijerinckii GCA_000833105.2
495 Clostridium saccharoperbutylacetonicum N1-4_28HMT_29 GCA_000340885.1
481 Clostridium saccharobutylicum DSM 13864 GCA_000473995.1
ga
493 Rhodobacter sphaeroides 2.4.1 GCA_000012905.2

462 Rhodobacter sphaeroides ATCC 17025 GCA_000016405.1
393 Dinoroseobacter shibae DFL 12 = DSM 16493 GCA_000018145.1
gb
689 Lactobacillus gasseri ATCC 33323 = JCM 1131 GCA_000014425.1
659 Lactobacillus hominis DSM 23910 = CRBIP 24.179 GCA_000296835.1
513 Lactobacillus iners DSM 13335 GCA_000160875.1
gc
731 Staphylococcus epidermidis ATCC 12228 GCA_000007645.1
701 Staphylococcus capitis subsp. capitis GCA_001028645.1
651 Staphylococcus lugdunensis HKU09-01 GCA_000025085.1
gd
694 Streptococcus mutans UA159 GCA_000007465.2
522 Streptococcus ratti FA-1 = DSM 20564 GCA_000286075.1
510 Streptococcus gordonii str. Challis substr. CH1 GCA_000017005.1
ge
609 Deinococcus radiodurans R1 GCA_000008565.1
405 Deinococcus deserti VCD115 GCA_000020685.1
375 Deinococcus frigens DSM 12807 GCA_000701425.1
gf
663 Clostridium beijerinckii GCA_000833105.2
644 Clostridium saccharoperbutylacetonicum N1-4_28HMT_29 GCA_000340885.1
622 Clostridium puniceum GCA_002006345.1
gg
499 Lactobacillus gasseri ATCC 33323 = JCM 1131 GCA_000014425.1
471 Lactobacillus hominis DSM 23910 = CRBIP 24.179 GCA_000296835.1
416 Lactobacillus iners DSM 13335 GCA_000160875.1
gh
326 Bifidobacterium adolescentis ATCC 15703 GCA_000010425.1
294 Bifidobacterium longum NCC2705 GCA_000007525.1
275 Bifidobacterium subtile GCA_000741775.1
gi
963 Bacillus cereus ATCC 14579 GCA_000007825.1
961 Bacillus anthracis str. Ames GCA_000007845.1
961 Bacillus anthracis str. Sterne GCA_000008165.1
961 _5BBacillus thuringiensis_5D serovar konkukian str. 97-27 GCA_000008505.1
935 Bacillus thuringiensis YBT-1518 GCA_000497525.2
gj
916 Lactobacillus gasseri ATCC 33323 = JCM 1131 GCA_000014425.1
906 Lactobacillus hominis DSM 23910 = CRBIP 24.179 GCA_000296835.1
681 Lactobacillus jensenii GCA_001936235.1
gk
736 Enterococcus faecalis V583 GCA_000007785.1
689 Streptomyces cinnamoneus GCA_001885705.1
649 Enterococcus rivorum GCA_001742285.1
gl
520 Deinococcus radiodurans R1 GCA_000008565.1
274 Deinococcus deserti VCD115 GCA_000020685.1
256 Deinococcus gobiensis I-0 GCA_000252445.1
gm
545 Escherichia coli str. K-12 substr. MG1655 GCA_000005845.2
531 Escherichia coli O83_3AH1 str. NRG 857C GCA_000183345.1
527 Escherichia coli O157_3AH7 str. Sakai GCA_000008865.1
527 Escherichia coli UMN026 GCA_000026325.2
527 Shigella flexneri 2a str. 301 GCA_000006925.2
gn
639 Lactobacillus gasseri ATCC 33323 = JCM 1131 GCA_000014425.1
609 Lactobacillus hominis DSM 23910 = CRBIP 24.179 GCA_000296835.1
429 Lactobacillus iners DSM 13335 GCA_000160875.1
go
736 Staphylococcus epidermidis ATCC 12228 GCA_000007645.1
686 Staphylococcus capitis subsp. capitis GCA_001028645.1
644 Staphylococcus hominis subsp. hominis C80 GCA_000183685.1
gp
744 Bacillus anthracis str. Ames GCA_000007845.1
744 Bacillus anthracis str. Sterne GCA_000008165.1
744 _5BBacillus thuringiensis_5D serovar konkukian str. 97-27 GCA_000008505.1
728 Bacillus pseudomycoides DSM 12442 GCA_000161455.1
724 Bacillus cereus ATCC 14579 GCA_000007825.1
gq
720 Bacillus anthracis str. Ames GCA_000007845.1
720 Bacillus anthracis str. Sterne GCA_000008165.1
720 Bacillus cereus ATCC 14579 GCA_000007825.1
720 _5BBacillus thuringiensis_5D serovar konkukian str. 97-27 GCA_000008505.1
702 Bacillus thuringiensis YBT-1518 GCA_000497525.2
699 Bacillus mycoides GCA_000832605.1
gr

546 Rhodobacter sphaeroides 2.4.1 GCA_000012905.2
504 Rhodobacter sphaeroides ATCC 17025 GCA_000016405.1
396 Gemmobacter megaterium GCA_900156815.1
gs
683 Bifidobacterium adolescentis ATCC 15703 GCA_000010425.1
556 Bifidobacterium dentium JCM 1195 = DSM 20436 GCA_001042595.1
527 Bifidobacterium angulatum DSM 20098 = JCM 7096 GCA_001025155.1
527 Bifidobacterium stellenboschense GCA_000741785.1
gt
680 Rhodobacter sphaeroides 2.4.1 GCA_000012905.2
612 Rhodobacter sphaeroides ATCC 17025 GCA_000016405.1
532 Defluviimonas alba GCA_001620265.1
gu
504 Escherichia coli O157_3AH7 str. Sakai GCA_000008865.1
504 Escherichia coli str. K-12 substr. MG1655 GCA_000005845.2
496 Escherichia coli O104_3AH4 str. 2011C-3493 GCA_000299455.1
496 Shigella flexneri 2a str. 301 GCA_000006925.2
491 Escherichia coli UMN026 GCA_000026325.2
gv
952 Bacillus anthracis str. Ames GCA_000007845.1
952 Bacillus anthracis str. Sterne GCA_000008165.1
952 _5BBacillus thuringiensis_5D serovar konkukian str. 97-27 GCA_000008505.1
935 Bacillus cereus ATCC 14579 GCA_000007825.1
935 Bacillus pseudomycoides DSM 12442 GCA_000161455.1
909 Bacillus thuringiensis YBT-1518 GCA_000497525.2
gw
608 Streptococcus mutans UA159 GCA_000007465.2
455 Streptococcus ratti FA-1 = DSM 20564 GCA_000286075.1
445 Streptococcus ferus DSM 20646 GCA_000372425.1
gx
839 Deinococcus radiodurans R1 GCA_000008565.1
443 Deinococcus deserti VCD115 GCA_000020685.1
439 Deinococcus soli Cha et al. 2016 GCA_001007995.1
gy
492 Clostridium beijerinckii GCA_000833105.2
465 Clostridium saccharoperbutylacetonicum N1-4_28HMT_29 GCA_000340885.1
435 Clostridium butyricum GCA_001456065.2
gz
540 Clostridium beijerinckii GCA_000833105.2
522 Clostridium saccharobutylicum DSM 13864 GCA_000473995.1
504 Clostridium saccharoperbutylacetonicum N1-4_28HMT_29 GCA_000340885.1
gA
852 Bifidobacterium adolescentis ATCC 15703 GCA_000010425.1
715 Bifidobacterium dentium JCM 1195 = DSM 20436 GCA_001042595.1
695 Bifidobacterium angulatum DSM 20098 = JCM 7096 GCA_001025155.1
gB
604 Lactobacillus gasseri ATCC 33323 = JCM 1131 GCA_000014425.1
565 Lactobacillus hominis DSM 23910 = CRBIP 24.179 GCA_000296835.1
380 Lactobacillus iners DSM 13335 GCA_000160875.1
gC
690 Lactobacillus gasseri ATCC 33323 = JCM 1131 GCA_000014425.1
646 Lactobacillus hominis DSM 23910 = CRBIP 24.179 GCA_000296835.1
424 Lactobacillus iners DSM 13335 GCA_000160875.1
gD
478 Staphylococcus epidermidis ATCC 12228 GCA_000007645.1
458 Megasphaera cerevisiae DSM 20462 GCA_001045675.1
458 Staphylococcus warneri SG1 GCA_000332735.1
453 Staphylococcus capitis subsp. capitis GCA_001028645.1
gE
737 Lactobacillus gasseri ATCC 33323 = JCM 1131 GCA_000014425.1
651 Lactobacillus hominis DSM 23910 = CRBIP 24.179 GCA_000296835.1
510 Lactobacillus psittaci DSM 15354 GCA_000425905.1
gF
752 Deinococcus radiodurans R1 GCA_000008565.1
449 Deinococcus deserti VCD115 GCA_000020685.1
441 Deinococcus proteolyticus MRP GCA_000190555.1
gG
834 Escherichia coli str. K-12 substr. MG1655 GCA_000005845.2
833 Escherichia coli IAI39 GCA_000026345.1
833 Escherichia coli O157_3AH7 str. Sakai GCA_000008865.1
817 Escherichia coli UMN026 GCA_000026325.2
gH
836 Lactobacillus gasseri ATCC 33323 = JCM 1131 GCA_000014425.1
749 Lactobacillus hominis DSM 23910 = CRBIP 24.179 GCA_000296835.1
543 Lactobacillus iners DSM 13335 GCA_000160875.1
gI

934 Staphylococcus epidermidis ATCC 12228 GCA_000007645.1
852 Staphylococcus capitis subsp. capitis GCA_001028645.1
802 Staphylococcus hominis subsp. hominis C80 GCA_000183685.1
gJ
845 Bacillus anthracis str. Ames GCA_000007845.1
845 Bacillus anthracis str. Sterne GCA_000008165.1
845 _5BBacillus thuringiensis_5D serovar konkukian str. 97-27 GCA_000008505.1
830 Bacillus cereus ATCC 14579 GCA_000007825.1
822 Bacillus pseudomycoides DSM 12442 GCA_000161455.1
gK
578 Clostridium beijerinckii GCA_000833105.2
546 Clostridium saccharoperbutylacetonicum N1-4_28HMT_29 GCA_000340885.1
527 Clostridium butyricum GCA_001456065.2
527 Clostridium neonatale GCA_001458595.1
gL
821 Streptococcus mutans UA159 GCA_000007465.2
558 Streptococcus ratti FA-1 = DSM 20564 GCA_000286075.1
530 Streptococcus macacae NCTC 11558 GCA_000187995.3
gM
737 Enterococcus faecalis V583 GCA_000007785.1
690 Streptomyces cinnamoneus GCA_001885705.1
652 Enterococcus massiliensis GCA_001050095.1
gN
554 Staphylococcus epidermidis ATCC 12228 GCA_000007645.1
504 Staphylococcus capitis subsp. capitis GCA_001028645.1
488 Staphylococcus aureus subsp. aureus NCTC 8325 GCA_000013425.1
gO
690 Clostridium beijerinckii GCA_000833105.2
669 Clostridium saccharoperbutylacetonicum N1-4_28HMT_29 GCA_000340885.1
662 Clostridium saccharobutylicum DSM 13864 GCA_000473995.1
gP
809 Bacillus thuringiensis YBT-1518 GCA_000497525.2
800 Bacillus anthracis str. Ames GCA_000007845.1
800 Bacillus anthracis str. Sterne GCA_000008165.1
800 Bacillus cereus ATCC 14579 GCA_000007825.1
800 _5BBacillus thuringiensis_5D serovar konkukian str. 97-27 GCA_000008505.1
785 Bacillus pseudomycoides DSM 12442 GCA_000161455.1
gQ
802 Bifidobacterium adolescentis ATCC 15703 GCA_000010425.1
657 Bifidobacterium dentium JCM 1195 = DSM 20436 GCA_001042595.1
612 Bifidobacterium angulatum DSM 20098 = JCM 7096 GCA_001025155.1
gR
562 Lactobacillus gasseri ATCC 33323 = JCM 1131 GCA_000014425.1
478 Lactobacillus hominis DSM 23910 = CRBIP 24.179 GCA_000296835.1
301 Lactobacillus psittaci DSM 15354 GCA_000425905.1
gS
660 Rhodobacter sphaeroides 2.4.1 GCA_000012905.2
616 Rhodobacter sphaeroides ATCC 17025 GCA_000016405.1
495 Pseudorhodobacter psychrotolerans GCA_001294535.1
gT
756 Enterococcus faecalis V583 GCA_000007785.1
716 Streptomyces cinnamoneus GCA_001885705.1
668 Enterococcus canis NBRC 100695 GCA_001544375.1
gU
871 Clostridium beijerinckii GCA_000833105.2
848 Clostridium saccharoperbutylacetonicum N1-4_28HMT_29 GCA_000340885.1
844 Clostridium puniceum GCA_002006345.1
gV
639 Clostridium beijerinckii GCA_000833105.2
597 Clostridium saccharoperbutylacetonicum N1-4_28HMT_29 GCA_000340885.1
590 Clostridium puniceum GCA_002006345.1
gW
452 Clostridium beijerinckii GCA_000833105.2
428 Clostridium saccharoperbutylacetonicum N1-4_28HMT_29 GCA_000340885.1
423 Clostridium chromiireducens GCA_002029255.1
gX
872 Bacillus anthracis str. Ames GCA_000007845.1
872 Bacillus anthracis str. Sterne GCA_000008165.1
872 _5BBacillus thuringiensis_5D serovar konkukian str. 97-27 GCA_000008505.1
871 Bacillus cereus ATCC 14579 GCA_000007825.1
871 Bacillus thuringiensis YBT-1518 GCA_000497525.2
843 Bacillus pseudomycoides DSM 12442 GCA_000161455.1
gY
721 Streptococcus mutans UA159 GCA_000007465.2
545 Streptococcus ratti FA-1 = DSM 20564 GCA_000286075.1
489 Streptococcus gordonii str. Challis substr. CH1 GCA_000017005.1

gZ
602 Deinococcus radiodurans R1 GCA_000008565.1
394 Deinococcus deserti VCD115 GCA_000020685.1
367 Deinococcus hopiensis KR-140 GCA_900176165.1
h0
475 Streptococcus mutans UA159 GCA_000007465.2
355 Streptococcus ratti FA-1 = DSM 20564 GCA_000286075.1
340 Streptococcus macacae NCTC 11558 GCA_000187995.3
h1
542 Streptococcus mutans UA159 GCA_000007465.2
401 Streptococcus gordonii str. Challis substr. CH1 GCA_000017005.1
388 Streptococcus salivarius GCA_000785515.1
h2
502 Deinococcus radiodurans R1 GCA_000008565.1
341 Deinococcus deserti VCD115 GCA_000020685.1
341 Deinococcus puniceus GCA_001644565.1
339 Deinococcus murrayi DSM 11303 GCA_000482805.1
339 Deinococcus phoenicis GCA_000599865.1
h3
845 Bacillus anthracis str. Ames GCA_000007845.1
845 Bacillus anthracis str. Sterne GCA_000008165.1
845 _5BBacillus thuringiensis_5D serovar konkukian str. 97-27 GCA_000008505.1
835 Bacillus cereus ATCC 14579 GCA_000007825.1
810 Bacillus thuringiensis YBT-1518 GCA_000497525.2
h4
739 Deinococcus radiodurans R1 GCA_000008565.1
535 Deinococcus puniceus GCA_001644565.1
528 Deinococcus gobiensis I-0 GCA_000252445.1
h5
540 Streptococcus mutans UA159 GCA_000007465.2
418 Streptococcus ratti FA-1 = DSM 20564 GCA_000286075.1
409 Streptococcus macacae NCTC 11558 GCA_000187995.3
h6
979 _5BBacillus thuringiensis_5D serovar konkukian str. 97-27 GCA_000008505.1
963 Bacillus anthracis str. Ames GCA_000007845.1
963 Bacillus anthracis str. Sterne GCA_000008165.1
943 Bacillus cereus ATCC 14579 GCA_000007825.1
943 Bacillus thuringiensis YBT-1518 GCA_000497525.2
h7
797 Lactobacillus gasseri ATCC 33323 = JCM 1131 GCA_000014425.1
761 Lactobacillus hominis DSM 23910 = CRBIP 24.179 GCA_000296835.1
579 Lactobacillus psittaci DSM 15354 GCA_000425905.1
h8
573 Rhodobacter sphaeroides 2.4.1 GCA_000012905.2
558 Rhodobacter sphaeroides ATCC 17025 GCA_000016405.1
513 Gemmobacter aquatilis GCA_900110025.1
h9
562 Bacillus thuringiensis YBT-1518 GCA_000497525.2
542 Bacillus anthracis str. Ames GCA_000007845.1
542 Bacillus anthracis str. Sterne GCA_000008165.1
542 _5BBacillus thuringiensis_5D serovar konkukian str. 97-27 GCA_000008505.1
541 Bacillus cereus ATCC 14579 GCA_000007825.1
ha
401 Bifidobacterium adolescentis ATCC 15703 GCA_000010425.1
308 Bifidobacterium dentium JCM 1195 = DSM 20436 GCA_001042595.1
293 Bifidobacterium stellenboschense GCA_000741785.1
hb
973 Enterococcus faecalis V583 GCA_000007785.1
926 Streptomyces cinnamoneus GCA_001885705.1
842 Enterococcus saccharolyticus subsp. saccharolyticus ATCC 43076 GCA_000407285.1
hc
714 Escherichia coli O104_3AH4 str. 2011C-3493 GCA_000299455.1
714 Escherichia coli str. K-12 substr. MG1655 GCA_000005845.2
707 Shigella flexneri 2a str. 301 GCA_000006925.2
685 Escherichia coli O157_3AH7 str. Sakai GCA_000008865.1
hd
561 Rhodobacter sphaeroides 2.4.1 GCA_000012905.2
506 Rhodobacter sphaeroides ATCC 17025 GCA_000016405.1
480 Gemmobacter aquatilis GCA_900110025.1
he
572 Deinococcus radiodurans R1 GCA_000008565.1
353 Deinococcus gobiensis I-0 GCA_000252445.1
329 Deinococcus deserti VCD115 GCA_000020685.1
hf
695 Enterococcus faecalis V583 GCA_000007785.1
648 Streptomyces cinnamoneus GCA_001885705.1

634 Enterococcus rivorum GCA_001742285.1
hg
615 Clostridium beijerinckii GCA_000833105.2
578 Clostridium saccharoperbutylacetonicum N1-4_28HMT_29 GCA_000340885.1
564 Clostridium puniceum GCA_002006345.1
hh
631 Lactobacillus gasseri ATCC 33323 = JCM 1131 GCA_000014425.1
611 Lactobacillus hominis DSM 23910 = CRBIP 24.179 GCA_000296835.1
432 Lactobacillus iners DSM 13335 GCA_000160875.1
hi
828 Staphylococcus epidermidis ATCC 12228 GCA_000007645.1
753 Staphylococcus capitis subsp. capitis GCA_001028645.1
713 Staphylococcus warneri SG1 GCA_000332735.1
hj
684 Staphylococcus epidermidis ATCC 12228 GCA_000007645.1
643 Staphylococcus capitis subsp. capitis GCA_001028645.1
623 Staphylococcus aureus subsp. aureus NCTC 8325 GCA_000013425.1
hk
915 Staphylococcus epidermidis ATCC 12228 GCA_000007645.1
852 Staphylococcus capitis subsp. capitis GCA_001028645.1
798 Staphylococcus aureus subsp. aureus NCTC 8325 GCA_000013425.1
hl
698 Clostridium beijerinckii GCA_000833105.2
685 Clostridium saccharoperbutylacetonicum N1-4_28HMT_29 GCA_000340885.1
675 Clostridium saccharobutylicum DSM 13864 GCA_000473995.1
hm
678 Rhodobacter sphaeroides 2.4.1 GCA_000012905.2
586 Rhodobacter sphaeroides ATCC 17025 GCA_000016405.1
509 Jannaschia donghaensis GCA_001403795.1
hn
656 Rhodobacter sphaeroides 2.4.1 GCA_000012905.2
562 Rhodobacter sphaeroides ATCC 17025 GCA_000016405.1
500 Pseudorhodobacter psychrotolerans GCA_001294535.1
ho
773 Clostridium beijerinckii GCA_000833105.2
751 Clostridium saccharoperbutylacetonicum N1-4_28HMT_29 GCA_000340885.1
722 Clostridium saccharobutylicum DSM 13864 GCA_000473995.1
hp
631 Staphylococcus epidermidis ATCC 12228 GCA_000007645.1
606 Staphylococcus capitis subsp. capitis GCA_001028645.1
543 Staphylococcus lugdunensis HKU09-01 GCA_000025085.1
hq
709 Bifidobacterium adolescentis ATCC 15703 GCA_000010425.1
569 Bifidobacterium dentium JCM 1195 = DSM 20436 GCA_001042595.1
543 Bifidobacterium angulatum DSM 20098 = JCM 7096 GCA_001025155.1
hr
769 Deinococcus radiodurans R1 GCA_000008565.1
490 Deinococcus deserti VCD115 GCA_000020685.1
465 Deinococcus gobiensis I-0 GCA_000252445.1
hs
624 Lactobacillus gasseri ATCC 33323 = JCM 1131 GCA_000014425.1
596 Lactobacillus hominis DSM 23910 = CRBIP 24.179 GCA_000296835.1
447 Lactobacillus hamsteri DSM 5661 = JCM 6256 GCA_000615445.1
ht
743 Clostridium beijerinckii GCA_000833105.2
718 Clostridium saccharoperbutylacetonicum N1-4_28HMT_29 GCA_000340885.1
692 Clostridium saccharobutylicum DSM 13864 GCA_000473995.1
hu
543 Escherichia coli str. K-12 substr. MG1655 GCA_000005845.2
525 Escherichia coli O157_3AH7 str. Sakai GCA_000008865.1
525 Shigella flexneri 2a str. 301 GCA_000006925.2
521 Escherichia coli O104_3AH4 str. 2011C-3493 GCA_000299455.1
hv
572 Deinococcus radiodurans R1 GCA_000008565.1
374 Deinococcus deserti VCD115 GCA_000020685.1
374 Deinococcus hopiensis KR-140 GCA_900176165.1
364 Deinococcus puniceus GCA_001644565.1
hw
892 Streptococcus mutans UA159 GCA_000007465.2
660 Streptococcus ratti FA-1 = DSM 20564 GCA_000286075.1
592 Streptococcus sobrinus DSM 20742 = ATCC 33478 GCA_000686605.1
hx
659 Staphylococcus epidermidis ATCC 12228 GCA_000007645.1
609 Staphylococcus capitis subsp. capitis GCA_001028645.1
590 Staphylococcus aureus subsp. aureus NCTC 8325 GCA_000013425.1
hy

907 Deinococcus radiodurans R1 GCA_000008565.1
541 Deinococcus deserti VCD115 GCA_000020685.1
517 Deinococcus gobiensis I-0 GCA_000252445.1
hz
697 Lactobacillus gasseri ATCC 33323 = JCM 1131 GCA_000014425.1
651 Lactobacillus hominis DSM 23910 = CRBIP 24.179 GCA_000296835.1
461 Lactobacillus iners DSM 13335 GCA_000160875.1
hA
396 Rhodobacter sphaeroides 2.4.1 GCA_000012905.2
355 Rhodobacter sphaeroides ATCC 17025 GCA_000016405.1
316 Defluviimonas alba GCA_001620265.1
hB
449 Escherichia coli str. K-12 substr. MG1655 GCA_000005845.2
433 Escherichia coli O104_3AH4 str. 2011C-3493 GCA_000299455.1
433 Escherichia coli O157_3AH7 str. Sakai GCA_000008865.1
433 Shigella flexneri 2a str. 301 GCA_000006925.2
432 Escherichia coli O83_3AH1 str. NRG 857C GCA_000183345.1
432 Escherichia coli UMN026 GCA_000026325.2
hC
508 Rhodobacter sphaeroides 2.4.1 GCA_000012905.2
442 Rhodobacter sphaeroides ATCC 17025 GCA_000016405.1
428 Gemmobacter aquatilis GCA_900110025.1
hD
659 Enterococcus faecalis V583 GCA_000007785.1
632 Enterococcus rivorum GCA_001742285.1
620 Enterococcus massiliensis GCA_001050095.1
hE
691 Clostridium beijerinckii GCA_000833105.2
681 Clostridium saccharoperbutylacetonicum N1-4_28HMT_29 GCA_000340885.1
664 Clostridium saccharobutylicum DSM 13864 GCA_000473995.1
hF
644 Staphylococcus epidermidis ATCC 12228 GCA_000007645.1
621 Megasphaera cerevisiae DSM 20462 GCA_001045675.1
621 Staphylococcus warneri SG1 GCA_000332735.1
604 Staphylococcus capitis subsp. capitis GCA_001028645.1
hG
895 Deinococcus radiodurans R1 GCA_000008565.1
605 Deinococcus deserti VCD115 GCA_000020685.1
563 Deinococcus murrayi DSM 11303 GCA_000482805.1
hH
713 Staphylococcus epidermidis ATCC 12228 GCA_000007645.1
696 Staphylococcus capitis subsp. capitis GCA_001028645.1
651 Staphylococcus warneri SG1 GCA_000332735.1
hI
747 Staphylococcus epidermidis ATCC 12228 GCA_000007645.1
712 Staphylococcus capitis subsp. capitis GCA_001028645.1
654 Staphylococcus warneri SG1 GCA_000332735.1
hJ
642 Clostridium beijerinckii GCA_000833105.2
605 Clostridium saccharoperbutylacetonicum N1-4_28HMT_29 GCA_000340885.1
592 Clostridium puniceum GCA_002006345.1
hK
633 Deinococcus radiodurans R1 GCA_000008565.1
413 Deinococcus deserti VCD115 GCA_000020685.1
409 Deinococcus hopiensis KR-140 GCA_900176165.1
hL
728 Lactobacillus gasseri ATCC 33323 = JCM 1131 GCA_000014425.1
676 Lactobacillus hominis DSM 23910 = CRBIP 24.179 GCA_000296835.1
462 Lactobacillus acetotolerans GCA_001042405.1
462 Lactobacillus iners DSM 13335 GCA_000160875.1
hM
297 Rhodobacter sphaeroides 2.4.1 GCA_000012905.2
286 Rhodobacter sphaeroides ATCC 17025 GCA_000016405.1
210 Gemmobacter aquatilis GCA_900110025.1
hN
508 Escherichia coli IAI39 GCA_000026345.1
508 Escherichia coli O104_3AH4 str. 2011C-3493 GCA_000299455.1
508 Escherichia coli UMN026 GCA_000026325.2
508 Escherichia coli str. K-12 substr. MG1655 GCA_000005845.2
508 Shigella flexneri 2a str. 301 GCA_000006925.2
503 Escherichia coli O157_3AH7 str. Sakai GCA_000008865.1
494 Escherichia coli O83_3AH1 str. NRG 857C GCA_000183345.1
hO
658 Bacillus anthracis str. Ames GCA_000007845.1
658 Bacillus anthracis str. Sterne GCA_000008165.1
658 _5BBacillus thuringiensis_5D serovar konkukian str. 97-27 GCA_000008505.1

643 Bacillus cereus ATCC 14579 GCA_000007825.1
633 Bacillus thuringiensis YBT-1518 GCA_000497525.2
hP
871 Rhodobacter sphaeroides 2.4.1 GCA_000012905.2
836 Rhodobacter sphaeroides ATCC 17025 GCA_000016405.1
723 Gemmobacter aquatilis GCA_900110025.1
hQ
706 Streptococcus mutans UA159 GCA_000007465.2
517 Streptococcus ratti FA-1 = DSM 20564 GCA_000286075.1
487 Streptococcus gordonii str. Challis substr. CH1 GCA_000017005.1
hR
754 Rhodobacter sphaeroides 2.4.1 GCA_000012905.2
710 Rhodobacter sphaeroides ATCC 17025 GCA_000016405.1
617 Pseudorhodobacter psychrotolerans GCA_001294535.1
hS
838 Escherichia coli str. K-12 substr. MG1655 GCA_000005845.2
828 Escherichia coli O104_3AH4 str. 2011C-3493 GCA_000299455.1
812 Shigella flexneri 2a str. 301 GCA_000006925.2
hT
852 Deinococcus radiodurans R1 GCA_000008565.1
531 Deinococcus deserti VCD115 GCA_000020685.1
522 Deinococcus gobiensis I-0 GCA_000252445.1
hU
656 Lactobacillus gasseri ATCC 33323 = JCM 1131 GCA_000014425.1
636 Lactobacillus hominis DSM 23910 = CRBIP 24.179 GCA_000296835.1
482 Lactobacillus hamsteri DSM 5661 = JCM 6256 GCA_000615445.1
hV
870 Bifidobacterium adolescentis ATCC 15703 GCA_000010425.1
729 Bifidobacterium dentium JCM 1195 = DSM 20436 GCA_001042595.1
708 Bifidobacterium angulatum DSM 20098 = JCM 7096 GCA_001025155.1
hW
759 Enterococcus faecalis V583 GCA_000007785.1
716 Streptomyces cinnamoneus GCA_001885705.1
648 Enterococcus asini ATCC 700915 GCA_000407365.1
hX
758 Bifidobacterium adolescentis ATCC 15703 GCA_000010425.1
612 Bifidobacterium dentium JCM 1195 = DSM 20436 GCA_001042595.1
574 Bifidobacterium angulatum DSM 20098 = JCM 7096 GCA_001025155.1
hY
687 Streptococcus mutans UA159 GCA_000007465.2
494 Streptococcus ratti FA-1 = DSM 20564 GCA_000286075.1
485 Streptococcus macacae NCTC 11558 GCA_000187995.3
hZ
664 Deinococcus radiodurans R1 GCA_000008565.1
367 Deinococcus deserti VCD115 GCA_000020685.1
310 Deinococcus soli Cha et al. 2016 GCA_001007995.1
i0
513 Rhodobacter sphaeroides 2.4.1 GCA_000012905.2
462 Rhodobacter sphaeroides ATCC 17025 GCA_000016405.1
369 Defluviimonas alba GCA_001620265.1
i1
598 Rhodobacter sphaeroides 2.4.1 GCA_000012905.2
493 Rhodobacter sphaeroides ATCC 17025 GCA_000016405.1
438 Gemmobacter megaterium GCA_900156815.1
i2
881 Deinococcus radiodurans R1 GCA_000008565.1
542 Deinococcus deserti VCD115 GCA_000020685.1
537 Deinococcus gobiensis I-0 GCA_000252445.1
i3
750 Clostridium beijerinckii GCA_000833105.2
685 Clostridium saccharoperbutylacetonicum N1-4_28HMT_29 GCA_000340885.1
664 Clostridium puniceum GCA_002006345.1
i4
619 Rhodobacter sphaeroides 2.4.1 GCA_000012905.2
543 Rhodobacter sphaeroides ATCC 17025 GCA_000016405.1
447 Pseudorhodobacter wandonensis GCA_001202035.1
i5
466 Streptococcus mutans UA159 GCA_000007465.2
324 Streptococcus gordonii str. Challis substr. CH1 GCA_000017005.1
313 Streptococcus equinus GCA_000964315.1
i6
656 Clostridium beijerinckii GCA_000833105.2
655 Clostridium saccharoperbutylacetonicum N1-4_28HMT_29 GCA_000340885.1
634 Clostridium saccharobutylicum DSM 13864 GCA_000473995.1
i7
902 Streptococcus mutans UA159 GCA_000007465.2

647 Streptococcus ratti FA-1 = DSM 20564 GCA_000286075.1
616 Streptococcus gordonii str. Challis substr. CH1 GCA_000017005.1
i8
595 Bifidobacterium adolescentis ATCC 15703 GCA_000010425.1
540 Bifidobacterium dentium JCM 1195 = DSM 20436 GCA_001042595.1
511 Bifidobacterium angulatum DSM 20098 = JCM 7096 GCA_001025155.1
i9
653 Bifidobacterium adolescentis ATCC 15703 GCA_000010425.1
556 Bifidobacterium angulatum DSM 20098 = JCM 7096 GCA_001025155.1
543 Bifidobacterium dentium JCM 1195 = DSM 20436 GCA_001042595.1
ia
466 Rhodobacter sphaeroides 2.4.1 GCA_000012905.2
442 Rhodobacter sphaeroides ATCC 17025 GCA_000016405.1
384 Pseudorhodobacter psychrotolerans GCA_001294535.1
ib
439 Rhodobacter sphaeroides 2.4.1 GCA_000012905.2
392 Rhodobacter sphaeroides ATCC 17025 GCA_000016405.1
346 Thioclava indica GCA_000714545.1
ic
194 Deinococcus radiodurans R1 GCA_000008565.1
97 Deinococcus gobiensis I-0 GCA_000252445.1
85 Deinococcus deserti VCD115 GCA_000020685.1
85 Deinococcus hopiensis KR-140 GCA_900176165.1
85 Deinococcus indicus GCA_002198095.1
85 Deinococcus soli Cha et al. 2016 GCA_001007995.1
id
562 Clostridium beijerinckii GCA_000833105.2
556 Clostridium saccharoperbutylacetonicum N1-4_28HMT_29 GCA_000340885.1
534 Bacillus anthracis str. Ames GCA_000007845.1
534 Bacillus anthracis str. Sterne GCA_000008165.1
534 Clostridium saccharobutylicum DSM 13864 GCA_000473995.1
534 _5BBacillus thuringiensis_5D serovar konkukian str. 97-27 GCA_000008505.1
ie
422 Bifidobacterium adolescentis ATCC 15703 GCA_000010425.1
360 Bifidobacterium angulatum DSM 20098 = JCM 7096 GCA_001025155.1
333 Bifidobacterium dentium JCM 1195 = DSM 20436 GCA_001042595.1
if
607 Escherichia coli O104_3AH4 str. 2011C-3493 GCA_000299455.1
607 Escherichia coli str. K-12 substr. MG1655 GCA_000005845.2
607 Shigella flexneri 2a str. 301 GCA_000006925.2
606 Escherichia coli UMN026 GCA_000026325.2
599 Escherichia coli O83_3AH1 str. NRG 857C GCA_000183345.1
ig
644 Staphylococcus epidermidis ATCC 12228 GCA_000007645.1
580 Megasphaera cerevisiae DSM 20462 GCA_001045675.1
580 Staphylococcus capitis subsp. capitis GCA_001028645.1
580 Staphylococcus warneri SG1 GCA_000332735.1
553 Staphylococcus aureus subsp. aureus NCTC 8325 GCA_000013425.1
ih
846 Staphylococcus epidermidis ATCC 12228 GCA_000007645.1
794 Staphylococcus capitis subsp. capitis GCA_001028645.1
737 Staphylococcus hominis subsp. hominis C80 GCA_000183685.1
737 Staphylococcus lugdunensis HKU09-01 GCA_000025085.1
ii
620 Streptococcus mutans UA159 GCA_000007465.2
471 Streptococcus ratti FA-1 = DSM 20564 GCA_000286075.1
432 Streptococcus criceti HS-6 GCA_000187975.3
ij
842 Streptococcus mutans UA159 GCA_000007465.2
653 Streptococcus ratti FA-1 = DSM 20564 GCA_000286075.1
609 Streptococcus gordonii str. Challis substr. CH1 GCA_000017005.1
ik
775 Clostridium beijerinckii GCA_000833105.2
725 Clostridium saccharoperbutylacetonicum N1-4_28HMT_29 GCA_000340885.1
718 Clostridium puniceum GCA_002006345.1
il
805 Clostridium beijerinckii GCA_000833105.2
775 Clostridium saccharoperbutylacetonicum N1-4_28HMT_29 GCA_000340885.1
749 Clostridium saccharobutylicum DSM 13864 GCA_000473995.1
im
613 Lactobacillus gasseri ATCC 33323 = JCM 1131 GCA_000014425.1
597 Lactobacillus hominis DSM 23910 = CRBIP 24.179 GCA_000296835.1
549 Staphylococcus epidermidis ATCC 12228 GCA_000007645.1
in
739 Rhodobacter sphaeroides 2.4.1 GCA_000012905.2
665 Rhodobacter sphaeroides ATCC 17025 GCA_000016405.1

525 Rhodobacter capsulatus SB 1003 GCA_000021865.1
io
759 Streptococcus mutans UA159 GCA_000007465.2
560 Streptococcus ratti FA-1 = DSM 20564 GCA_000286075.1
491 Streptococcus ferus DSM 20646 GCA_000372425.1
ip
570 Lactobacillus gasseri ATCC 33323 = JCM 1131 GCA_000014425.1
513 Lactobacillus hominis DSM 23910 = CRBIP 24.179 GCA_000296835.1
379 Lactobacillus iners DSM 13335 GCA_000160875.1
iq
747 Rhodobacter sphaeroides 2.4.1 GCA_000012905.2
689 Rhodobacter sphaeroides ATCC 17025 GCA_000016405.1
534 Pseudorhodobacter psychrotolerans GCA_001294535.1
ir
604 Staphylococcus epidermidis ATCC 12228 GCA_000007645.1
549 Staphylococcus capitis subsp. capitis GCA_001028645.1
514 Staphylococcus hominis subsp. hominis C80 GCA_000183685.1
is
680 Shigella flexneri 2a str. 301 GCA_000006925.2
678 Escherichia coli O104_3AH4 str. 2011C-3493 GCA_000299455.1
677 Escherichia coli str. K-12 substr. MG1655 GCA_000005845.2
it
764 Lactobacillus gasseri ATCC 33323 = JCM 1131 GCA_000014425.1
717 Lactobacillus hominis DSM 23910 = CRBIP 24.179 GCA_000296835.1
584 Lactobacillus psittaci DSM 15354 GCA_000425905.1
iu
596 Streptococcus mutans UA159 GCA_000007465.2
482 Streptococcus sobrinus DSM 20742 = ATCC 33478 GCA_000686605.1
465 Streptococcus ratti FA-1 = DSM 20564 GCA_000286075.1
iv
607 Streptococcus mutans UA159 GCA_000007465.2
462 Streptococcus gordonii str. Challis substr. CH1 GCA_000017005.1
425 Streptococcus ratti FA-1 = DSM 20564 GCA_000286075.1
iw
787 Escherichia coli O83_3AH1 str. NRG 857C GCA_000183345.1
779 Escherichia coli str. K-12 substr. MG1655 GCA_000005845.2
773 Escherichia coli O104_3AH4 str. 2011C-3493 GCA_000299455.1
773 Escherichia coli O157_3AH7 str. Sakai GCA_000008865.1
773 Shigella flexneri 2a str. 301 GCA_000006925.2
ix
595 Clostridium beijerinckii GCA_000833105.2
564 Clostridium butyricum GCA_001456065.2
553 Clostridium puniceum GCA_002006345.1
553 Clostridium saccharoperbutylacetonicum N1-4_28HMT_29 GCA_000340885.1
iy
754 Enterococcus faecalis V583 GCA_000007785.1
713 Streptomyces cinnamoneus GCA_001885705.1
650 Enterococcus canis NBRC 100695 GCA_001544375.1
650 Enterococcus rivorum GCA_001742285.1
iz
560 Clostridium beijerinckii GCA_000833105.2
549 Clostridium saccharoperbutylacetonicum N1-4_28HMT_29 GCA_000340885.1
534 Clostridium puniceum GCA_002006345.1
534 Clostridium saccharobutylicum DSM 13864 GCA_000473995.1
iA
886 Enterococcus faecalis V583 GCA_000007785.1
839 Streptomyces cinnamoneus GCA_001885705.1
756 Enterococcus haemoperoxidus ATCC BAA-382 GCA_000407165.1
iB
847 Clostridium beijerinckii GCA_000833105.2
792 Clostridium saccharoperbutylacetonicum N1-4_28HMT_29 GCA_000340885.1
784 Clostridium saccharobutylicum DSM 13864 GCA_000473995.1
iC
694 Lactobacillus gasseri ATCC 33323 = JCM 1131 GCA_000014425.1
661 Lactobacillus hominis DSM 23910 = CRBIP 24.179 GCA_000296835.1
465 Lactobacillus iners DSM 13335 GCA_000160875.1
iD
782 Deinococcus radiodurans R1 GCA_000008565.1
552 Deinococcus deserti VCD115 GCA_000020685.1
489 Deinococcus hopiensis KR-140 GCA_900176165.1
iE
891 Streptococcus mutans UA159 GCA_000007465.2
667 Streptococcus ratti FA-1 = DSM 20564 GCA_000286075.1
641 Streptococcus anginosus C238 GCA_000463505.1
iF
800 Rhodobacter sphaeroides 2.4.1 GCA_000012905.2

734 Rhodobacter sphaeroides ATCC 17025 GCA_000016405.1
622 Defluviimonas alba GCA_001620265.1
iG
416 Bifidobacterium adolescentis ATCC 15703 GCA_000010425.1
321 Bifidobacterium dentium JCM 1195 = DSM 20436 GCA_001042595.1
304 Bifidobacterium breve DSM 20213 = JCM 1192 GCA_001025175.1
iH
506 Lactobacillus gasseri ATCC 33323 = JCM 1131 GCA_000014425.1
402 Lactobacillus hominis DSM 23910 = CRBIP 24.179 GCA_000296835.1
315 Lactobacillus acetotolerans GCA_001042405.1
iI
612 Clostridium beijerinckii GCA_000833105.2
595 Clostridium saccharoperbutylacetonicum N1-4_28HMT_29 GCA_000340885.1
581 Clostridium puniceum GCA_002006345.1
iJ
516 Clostridium beijerinckii GCA_000833105.2
495 Clostridium saccharoperbutylacetonicum N1-4_28HMT_29 GCA_000340885.1
479 Clostridium saccharobutylicum DSM 13864 GCA_000473995.1
iK
558 Deinococcus radiodurans R1 GCA_000008565.1
270 Deinococcus deserti VCD115 GCA_000020685.1
263 Deinococcus puniceus GCA_001644565.1
iL
464 Clostridium beijerinckii GCA_000833105.2
449 Clostridium saccharoperbutylacetonicum N1-4_28HMT_29 GCA_000340885.1
433 Clostridium puniceum GCA_002006345.1
iM
674 Clostridium beijerinckii GCA_000833105.2
634 Clostridium saccharoperbutylacetonicum N1-4_28HMT_29 GCA_000340885.1
619 Clostridium puniceum GCA_002006345.1
iN
619 Staphylococcus epidermidis ATCC 12228 GCA_000007645.1
594 Staphylococcus capitis subsp. capitis GCA_001028645.1
565 Staphylococcus haemolyticus JCSC1435 GCA_000009865.1
iO
749 Deinococcus radiodurans R1 GCA_000008565.1
555 Deinococcus gobiensis I-0 GCA_000252445.1
529 Deinococcus deserti VCD115 GCA_000020685.1
iP
708 _5BBacillus thuringiensis_5D serovar konkukian str. 97-27 GCA_000008505.1
707 Bacillus anthracis str. Ames GCA_000007845.1
707 Bacillus anthracis str. Sterne GCA_000008165.1
700 Bacillus cereus ATCC 14579 GCA_000007825.1
iQ
510 Deinococcus radiodurans R1 GCA_000008565.1
289 Deinococcus soli Cha et al. 2016 GCA_001007995.1
272 Deinococcus deserti VCD115 GCA_000020685.1
272 Deinococcus gobiensis I-0 GCA_000252445.1
iR
609 Bacillus anthracis str. Ames GCA_000007845.1
609 Bacillus anthracis str. Sterne GCA_000008165.1
608 Bacillus cereus ATCC 14579 GCA_000007825.1
608 _5BBacillus thuringiensis_5D serovar konkukian str. 97-27 GCA_000008505.1
593 Bacillus pseudomycoides DSM 12442 GCA_000161455.1
593 Bacillus thuringiensis YBT-1518 GCA_000497525.2
iS
689 Enterococcus faecalis V583 GCA_000007785.1
651 Enterococcus rivorum GCA_001742285.1
643 Streptomyces cinnamoneus GCA_001885705.1
iT
651 Deinococcus radiodurans R1 GCA_000008565.1
399 Deinococcus deserti VCD115 GCA_000020685.1
374 Deinococcus hopiensis KR-140 GCA_900176165.1
iU
755 Rhodobacter sphaeroides 2.4.1 GCA_000012905.2
698 Rhodobacter sphaeroides ATCC 17025 GCA_000016405.1
526 Gemmobacter aquatilis GCA_900110025.1
iV
552 Staphylococcus epidermidis ATCC 12228 GCA_000007645.1
510 Staphylococcus capitis subsp. capitis GCA_001028645.1
489 Staphylococcus aureus subsp. aureus NCTC 8325 GCA_000013425.1
iW
623 Rhodobacter sphaeroides 2.4.1 GCA_000012905.2
544 Rhodobacter sphaeroides ATCC 17025 GCA_000016405.1
489 Pseudorhodobacter ferrugineus DSM 5888 GCA_000420745.1
iX

896 Streptococcus mutans UA159 GCA_000007465.2
663 Streptococcus equinus GCA_000964315.1
663 Streptococcus ratti FA-1 = DSM 20564 GCA_000286075.1
646 Streptococcus pseudoporcinus LQ 940-04 GCA_000188035.3
iY
622 Lactobacillus gasseri ATCC 33323 = JCM 1131 GCA_000014425.1
561 Lactobacillus hominis DSM 23910 = CRBIP 24.179 GCA_000296835.1
382 Lactobacillus iners DSM 13335 GCA_000160875.1
iZ
821 Streptococcus mutans UA159 GCA_000007465.2
647 Streptococcus ratti FA-1 = DSM 20564 GCA_000286075.1
573 Streptococcus gordonii str. Challis substr. CH1 GCA_000017005.1
j0
795 Deinococcus radiodurans R1 GCA_000008565.1
517 Deinococcus deserti VCD115 GCA_000020685.1
488 Deinococcus hopiensis KR-140 GCA_900176165.1
j1
634 Clostridium beijerinckii GCA_000833105.2
577 Clostridium saccharoperbutylacetonicum N1-4_28HMT_29 GCA_000340885.1
573 Clostridium puniceum GCA_002006345.1
j2
734 Rhodobacter sphaeroides 2.4.1 GCA_000012905.2
694 Rhodobacter sphaeroides ATCC 17025 GCA_000016405.1
587 Pseudorhodobacter ferrugineus DSM 5888 GCA_000420745.1
j3
651 Staphylococcus epidermidis ATCC 12228 GCA_000007645.1
631 Staphylococcus capitis subsp. capitis GCA_001028645.1
576 Staphylococcus haemolyticus JCSC1435 GCA_000009865.1
576 Staphylococcus hominis subsp. hominis C80 GCA_000183685.1
j4
549 Bacillus anthracis str. Ames GCA_000007845.1
549 Bacillus anthracis str. Sterne GCA_000008165.1
549 Bacillus cereus ATCC 14579 GCA_000007825.1
549 Bacillus thuringiensis YBT-1518 GCA_000497525.2
549 _5BBacillus thuringiensis_5D serovar konkukian str. 97-27 GCA_000008505.1
534 Bacillus pseudomycoides DSM 12442 GCA_000161455.1
517 Bacillus cytotoxicus NVH 391-98 GCA_000017425.1
j5
512 Escherichia coli O83_3AH1 str. NRG 857C GCA_000183345.1
508 Escherichia coli O157_3AH7 str. Sakai GCA_000008865.1
508 Escherichia coli UMN026 GCA_000026325.2
508 Escherichia coli str. K-12 substr. MG1655 GCA_000005845.2
508 Shigella flexneri 2a str. 301 GCA_000006925.2
493 Escherichia coli IAI39 GCA_000026345.1
j6
796 Staphylococcus epidermidis ATCC 12228 GCA_000007645.1
765 Staphylococcus capitis subsp. capitis GCA_001028645.1
686 Staphylococcus warneri SG1 GCA_000332735.1
j7
770 Rhodobacter sphaeroides 2.4.1 GCA_000012905.2
707 Rhodobacter sphaeroides ATCC 17025 GCA_000016405.1
577 Pseudorhodobacter ferrugineus DSM 5888 GCA_000420745.1
j8
671 Staphylococcus epidermidis ATCC 12228 GCA_000007645.1
619 Staphylococcus capitis subsp. capitis GCA_001028645.1
568 Staphylococcus aureus subsp. aureus NCTC 8325 GCA_000013425.1
j9
528 Bifidobacterium adolescentis ATCC 15703 GCA_000010425.1
470 Bifidobacterium dentium JCM 1195 = DSM 20436 GCA_001042595.1
446 Bifidobacterium angulatum DSM 20098 = JCM 7096 GCA_001025155.1
ja
607 Rhodobacter sphaeroides 2.4.1 GCA_000012905.2
568 Rhodobacter sphaeroides ATCC 17025 GCA_000016405.1
500 Gemmobacter aquatilis GCA_900110025.1
jb
868 Staphylococcus epidermidis ATCC 12228 GCA_000007645.1
808 Staphylococcus capitis subsp. capitis GCA_001028645.1
795 Staphylococcus lugdunensis HKU09-01 GCA_000025085.1
jc
667 Deinococcus radiodurans R1 GCA_000008565.1
499 Deinococcus deserti VCD115 GCA_000020685.1
440 Deinococcus gobiensis I-0 GCA_000252445.1
jd
727 Clostridium beijerinckii GCA_000833105.2
695 Clostridium saccharoperbutylacetonicum N1-4_28HMT_29 GCA_000340885.1
688 Clostridium puniceum GCA_002006345.1

je
771 Bifidobacterium adolescentis ATCC 15703 GCA_000010425.1
618 Bifidobacterium angulatum DSM 20098 = JCM 7096 GCA_001025155.1
608 Bifidobacterium dentium JCM 1195 = DSM 20436 GCA_001042595.1
jf
834 Streptococcus mutans UA159 GCA_000007465.2
644 Streptococcus ratti FA-1 = DSM 20564 GCA_000286075.1
563 Streptococcus equinus GCA_000964315.1
jg
484 Deinococcus radiodurans R1 GCA_000008565.1
294 Deinococcus soli Cha et al. 2016 GCA_001007995.1
292 Deinococcus deserti VCD115 GCA_000020685.1
jh
558 Rhodobacter sphaeroides 2.4.1 GCA_000012905.2
486 Rhodobacter sphaeroides ATCC 17025 GCA_000016405.1
397 Gemmobacter aquatilis GCA_900110025.1
ji
494 Rhodobacter sphaeroides 2.4.1 GCA_000012905.2
442 Rhodobacter sphaeroides ATCC 17025 GCA_000016405.1
403 Gemmobacter nectariphilus DSM 15620 GCA_000429765.1
jj
627 Clostridium beijerinckii GCA_000833105.2
555 Clostridium saccharobutylicum DSM 13864 GCA_000473995.1
551 Clostridium saccharoperbutylacetonicum N1-4_28HMT_29 GCA_000340885.1
jk
964 Deinococcus radiodurans R1 GCA_000008565.1
619 Deinococcus deserti VCD115 GCA_000020685.1
577 Deinococcus marmoris DSM 12784 GCA_000701405.1
jl
863 Enterococcus faecalis V583 GCA_000007785.1
816 Streptomyces cinnamoneus GCA_001885705.1
727 Enterococcus rivorum GCA_001742285.1
jm
678 Lactobacillus gasseri ATCC 33323 = JCM 1131 GCA_000014425.1
642 Lactobacillus hominis DSM 23910 = CRBIP 24.179 GCA_000296835.1
425 Lactobacillus iners DSM 13335 GCA_000160875.1
jn
626 Clostridium beijerinckii GCA_000833105.2
607 Clostridium saccharoperbutylacetonicum N1-4_28HMT_29 GCA_000340885.1
598 Clostridium saccharobutylicum DSM 13864 GCA_000473995.1
jo
744 Enterococcus faecalis V583 GCA_000007785.1
703 Streptomyces cinnamoneus GCA_001885705.1
648 Enterococcus rivorum GCA_001742285.1
jp
728 Rhodobacter sphaeroides 2.4.1 GCA_000012905.2
636 Rhodobacter sphaeroides ATCC 17025 GCA_000016405.1
541 Pseudorhodobacter ferrugineus DSM 5888 GCA_000420745.1
jq
555 Clostridium saccharoperbutylacetonicum N1-4_28HMT_29 GCA_000340885.1
545 Clostridium beijerinckii GCA_000833105.2
538 Clostridium saccharobutylicum DSM 13864 GCA_000473995.1
jr
723 Clostridium beijerinckii GCA_000833105.2
696 Clostridium saccharoperbutylacetonicum N1-4_28HMT_29 GCA_000340885.1
672 Clostridium puniceum GCA_002006345.1
js
546 Clostridium saccharoperbutylacetonicum N1-4_28HMT_29 GCA_000340885.1
541 Clostridium beijerinckii GCA_000833105.2
516 Clostridium puniceum GCA_002006345.1
jt
824 Clostridium beijerinckii GCA_000833105.2
767 Clostridium saccharoperbutylacetonicum N1-4_28HMT_29 GCA_000340885.1
757 Clostridium saccharobutylicum DSM 13864 GCA_000473995.1
ju
714 Lactobacillus gasseri ATCC 33323 = JCM 1131 GCA_000014425.1
631 Lactobacillus hominis DSM 23910 = CRBIP 24.179 GCA_000296835.1
479 Lactobacillus iners DSM 13335 GCA_000160875.1
jv
470 Clostridium beijerinckii GCA_000833105.2
465 Clostridium saccharoperbutylacetonicum N1-4_28HMT_29 GCA_000340885.1
460 Staphylococcus epidermidis ATCC 12228 GCA_000007645.1
jw
850 Lactobacillus gasseri ATCC 33323 = JCM 1131 GCA_000014425.1
766 Lactobacillus hominis DSM 23910 = CRBIP 24.179 GCA_000296835.1
551 Lactobacillus iners DSM 13335 GCA_000160875.1

jx
672 Enterococcus faecalis V583 GCA_000007785.1
625 Streptomyces cinnamoneus GCA_001885705.1
595 Enterococcus phoeniculicola ATCC BAA-412 GCA_000407505.1
jy
565 Escherichia coli O104_3AH4 str. 2011C-3493 GCA_000299455.1
565 Escherichia coli O157_3AH7 str. Sakai GCA_000008865.1
565 Escherichia coli str. K-12 substr. MG1655 GCA_000005845.2
565 Shigella flexneri 2a str. 301 GCA_000006925.2
557 Escherichia coli O83_3AH1 str. NRG 857C GCA_000183345.1
557 Escherichia coli UMN026 GCA_000026325.2
520 Escherichia coli IAI39 GCA_000026345.1
jz
584 Staphylococcus epidermidis ATCC 12228 GCA_000007645.1
569 Staphylococcus capitis subsp. capitis GCA_001028645.1
514 Staphylococcus warneri SG1 GCA_000332735.1
jA
584 Enterococcus faecalis V583 GCA_000007785.1
538 Enterococcus rivorum GCA_001742285.1
538 Streptomyces cinnamoneus GCA_001885705.1
535 Enterococcus asini ATCC 700915 GCA_000407365.1
jB
729 Lactobacillus gasseri ATCC 33323 = JCM 1131 GCA_000014425.1
675 Lactobacillus hominis DSM 23910 = CRBIP 24.179 GCA_000296835.1
486 Lactobacillus hamsteri DSM 5661 = JCM 6256 GCA_000615445.1
jC
459 Escherichia coli str. K-12 substr. MG1655 GCA_000005845.2
417 Escherichia coli O104_3AH4 str. 2011C-3493 GCA_000299455.1
410 Escherichia coli UMN026 GCA_000026325.2
jD
675 Clostridium beijerinckii GCA_000833105.2
639 Clostridium saccharobutylicum DSM 13864 GCA_000473995.1
632 Clostridium saccharoperbutylacetonicum N1-4_28HMT_29 GCA_000340885.1
jE
752 Rhodobacter sphaeroides 2.4.1 GCA_000012905.2
700 Rhodobacter sphaeroides ATCC 17025 GCA_000016405.1
630 Gemmobacter aquatilis GCA_900110025.1
jF
347 Bacillus cereus ATCC 14579 GCA_000007825.1
333 Bacillus anthracis str. Ames GCA_000007845.1
333 Bacillus anthracis str. Sterne GCA_000008165.1
333 Bacillus thuringiensis YBT-1518 GCA_000497525.2
333 _5BBacillus thuringiensis_5D serovar konkukian str. 97-27 GCA_000008505.1
320 Bacillus mycoides GCA_000832605.1
320 Bacillus pseudomycoides DSM 12442 GCA_000161455.1
jG
768 Clostridium beijerinckii GCA_000833105.2
753 Clostridium saccharoperbutylacetonicum N1-4_28HMT_29 GCA_000340885.1
708 Clostridium puniceum GCA_002006345.1
jH
678 Clostridium beijerinckii GCA_000833105.2
643 Clostridium saccharoperbutylacetonicum N1-4_28HMT_29 GCA_000340885.1
600 Clostridium puniceum GCA_002006345.1
jI
676 Rhodobacter sphaeroides 2.4.1 GCA_000012905.2
613 Rhodobacter sphaeroides ATCC 17025 GCA_000016405.1
499 Pseudorhodobacter psychrotolerans GCA_001294535.1
jJ
697 Enterococcus faecalis V583 GCA_000007785.1
650 Streptomyces cinnamoneus GCA_001885705.1
642 Enterococcus rivorum GCA_001742285.1
jK
822 Enterococcus faecalis V583 GCA_000007785.1
775 Streptomyces cinnamoneus GCA_001885705.1
669 Enterococcus rivorum GCA_001742285.1
jL
763 Clostridium beijerinckii GCA_000833105.2
729 Clostridium saccharoperbutylacetonicum N1-4_28HMT_29 GCA_000340885.1
692 Clostridium saccharobutylicum DSM 13864 GCA_000473995.1
jM
904 Staphylococcus epidermidis ATCC 12228 GCA_000007645.1
837 Staphylococcus capitis subsp. capitis GCA_001028645.1
788 Staphylococcus aureus subsp. aureus NCTC 8325 GCA_000013425.1
jN
665 Bifidobacterium adolescentis ATCC 15703 GCA_000010425.1
487 Bifidobacterium angulatum DSM 20098 = JCM 7096 GCA_001025155.1

478 Bifidobacterium dentium JCM 1195 = DSM 20436 GCA_001042595.1
jO
720 Clostridium beijerinckii GCA_000833105.2
715 Clostridium saccharoperbutylacetonicum N1-4_28HMT_29 GCA_000340885.1
686 Clostridium saccharobutylicum DSM 13864 GCA_000473995.1
jP
815 Rhodobacter sphaeroides 2.4.1 GCA_000012905.2
737 Rhodobacter sphaeroides ATCC 17025 GCA_000016405.1
593 Gemmobacter aquatilis GCA_900110025.1
jQ
553 Staphylococcus epidermidis ATCC 12228 GCA_000007645.1
508 Staphylococcus capitis subsp. capitis GCA_001028645.1
498 Staphylococcus hominis subsp. hominis C80 GCA_000183685.1
jR
679 Clostridium beijerinckii GCA_000833105.2
654 Clostridium puniceum GCA_002006345.1
652 Clostridium saccharoperbutylacetonicum N1-4_28HMT_29 GCA_000340885.1
jS
715 Bacillus anthracis str. Ames GCA_000007845.1
715 Bacillus anthracis str. Sterne GCA_000008165.1
715 Bacillus cereus ATCC 14579 GCA_000007825.1
715 _5BBacillus thuringiensis_5D serovar konkukian str. 97-27 GCA_000008505.1
700 Bacillus thuringiensis YBT-1518 GCA_000497525.2
676 Bacillus mycoides GCA_000832605.1
jT
735 Escherichia coli O157_3AH7 str. Sakai GCA_000008865.1
735 Escherichia coli str. K-12 substr. MG1655 GCA_000005845.2
722 Escherichia coli IAI39 GCA_000026345.1
718 Escherichia coli UMN026 GCA_000026325.2
jU
935 Bacillus anthracis str. Ames GCA_000007845.1
935 Bacillus anthracis str. Sterne GCA_000008165.1
935 Bacillus cereus ATCC 14579 GCA_000007825.1
935 _5BBacillus thuringiensis_5D serovar konkukian str. 97-27 GCA_000008505.1
909 Bacillus thuringiensis YBT-1518 GCA_000497525.2
904 Bacillus pseudomycoides DSM 12442 GCA_000161455.1
jV
931 Rhodobacter sphaeroides 2.4.1 GCA_000012905.2
794 Rhodobacter sphaeroides ATCC 17025 GCA_000016405.1
668 Gemmobacter aquatilis GCA_900110025.1
668 Rhodobacter capsulatus SB 1003 GCA_000021865.1
jW
622 Streptococcus mutans UA159 GCA_000007465.2
455 Streptococcus gordonii str. Challis substr. CH1 GCA_000017005.1
436 Streptococcus orisratti DSM 15617 GCA_000380105.1
jX
828 Staphylococcus epidermidis ATCC 12228 GCA_000007645.1
813 Staphylococcus capitis subsp. capitis GCA_001028645.1
765 Staphylococcus aureus subsp. aureus NCTC 8325 GCA_000013425.1
jY
576 Bacillus anthracis str. Ames GCA_000007845.1
576 Bacillus anthracis str. Sterne GCA_000008165.1
576 Bacillus pseudomycoides DSM 12442 GCA_000161455.1
576 _5BBacillus thuringiensis_5D serovar konkukian str. 97-27 GCA_000008505.1
561 Bacillus cereus ATCC 14579 GCA_000007825.1
551 Bacillus thuringiensis YBT-1518 GCA_000497525.2
jZ
721 Rhodobacter sphaeroides 2.4.1 GCA_000012905.2
629 Rhodobacter sphaeroides ATCC 17025 GCA_000016405.1
551 Pseudorhodobacter ferrugineus DSM 5888 GCA_000420745.1
k0
795 Escherichia coli str. K-12 substr. MG1655 GCA_000005845.2
753 Escherichia coli O104_3AH4 str. 2011C-3493 GCA_000299455.1
739 Shigella flexneri 2a str. 301 GCA_000006925.2
k1
615 Escherichia coli O157_3AH7 str. Sakai GCA_000008865.1
615 Escherichia coli UMN026 GCA_000026325.2
615 Escherichia coli str. K-12 substr. MG1655 GCA_000005845.2
603 Escherichia coli O83_3AH1 str. NRG 857C GCA_000183345.1
599 Shigella flexneri 2a str. 301 GCA_000006925.2
k2
817 Enterococcus faecalis V583 GCA_000007785.1
770 Streptomyces cinnamoneus GCA_001885705.1
740 Enterococcus asini ATCC 700915 GCA_000407365.1
740 Enterococcus rivorum GCA_001742285.1
k3

709 Streptococcus mutans UA159 GCA_000007465.2
504 Streptococcus gordonii str. Challis substr. CH1 GCA_000017005.1
485 Streptococcus ratti FA-1 = DSM 20564 GCA_000286075.1
k4
719 Deinococcus radiodurans R1 GCA_000008565.1
499 Deinococcus gobiensis I-0 GCA_000252445.1
494 Deinococcus soli Cha et al. 2016 GCA_001007995.1
k5
595 Deinococcus radiodurans R1 GCA_000008565.1
361 Deinococcus gobiensis I-0 GCA_000252445.1
359 Deinococcus deserti VCD115 GCA_000020685.1
k6
695 Clostridium beijerinckii GCA_000833105.2
648 Clostridium butyricum GCA_001456065.2
645 Clostridium saccharobutylicum DSM 13864 GCA_000473995.1
k7
782 Bacillus cereus ATCC 14579 GCA_000007825.1
775 Bacillus anthracis str. Ames GCA_000007845.1
775 Bacillus anthracis str. Sterne GCA_000008165.1
775 Bacillus thuringiensis YBT-1518 GCA_000497525.2
775 _5BBacillus thuringiensis_5D serovar konkukian str. 97-27 GCA_000008505.1
766 Bacillus pseudomycoides DSM 12442 GCA_000161455.1
k8
636 Escherichia coli O157_3AH7 str. Sakai GCA_000008865.1
636 Escherichia coli str. K-12 substr. MG1655 GCA_000005845.2
628 Escherichia coli UMN026 GCA_000026325.2
614 Escherichia coli IAI39 GCA_000026345.1
k9
681 Streptococcus mutans UA159 GCA_000007465.2
547 Streptococcus ratti FA-1 = DSM 20564 GCA_000286075.1
506 Streptococcus macacae NCTC 11558 GCA_000187995.3
ka
793 Lactobacillus gasseri ATCC 33323 = JCM 1131 GCA_000014425.1
744 Lactobacillus hominis DSM 23910 = CRBIP 24.179 GCA_000296835.1
560 Lactobacillus iners DSM 13335 GCA_000160875.1
kb
595 Escherichia coli O104_3AH4 str. 2011C-3493 GCA_000299455.1
595 Escherichia coli O157_3AH7 str. Sakai GCA_000008865.1
595 Escherichia coli str. K-12 substr. MG1655 GCA_000005845.2
595 Shigella flexneri 2a str. 301 GCA_000006925.2
590 Escherichia coli IAI39 GCA_000026345.1
587 Escherichia coli O83_3AH1 str. NRG 857C GCA_000183345.1
587 Escherichia coli UMN026 GCA_000026325.2
kc
655 Escherichia coli O157_3AH7 str. Sakai GCA_000008865.1
655 Escherichia coli str. K-12 substr. MG1655 GCA_000005845.2
647 Escherichia coli UMN026 GCA_000026325.2
646 Shigella flexneri 2a str. 301 GCA_000006925.2
kd
533 Shigella flexneri 2a str. 301 GCA_000006925.2
528 Escherichia coli O104_3AH4 str. 2011C-3493 GCA_000299455.1
528 Escherichia coli O157_3AH7 str. Sakai GCA_000008865.1
528 Escherichia coli str. K-12 substr. MG1655 GCA_000005845.2
524 Escherichia coli IAI39 GCA_000026345.1
ke
615 Rhodobacter sphaeroides 2.4.1 GCA_000012905.2
501 Rhodobacter sphaeroides ATCC 17025 GCA_000016405.1
471 Gemmobacter megaterium GCA_900156815.1
kf
515 Lactobacillus gasseri ATCC 33323 = JCM 1131 GCA_000014425.1
490 Lactobacillus hominis DSM 23910 = CRBIP 24.179 GCA_000296835.1
329 Lactobacillus kefiranofaciens ZW3 GCA_000214785.1
kg
837 Escherichia coli O157_3AH7 str. Sakai GCA_000008865.1
837 Escherichia coli str. K-12 substr. MG1655 GCA_000005845.2
836 Escherichia coli UMN026 GCA_000026325.2
828 Shigella flexneri 2a str. 301 GCA_000006925.2
kh
761 Clostridium beijerinckii GCA_000833105.2
752 Clostridium saccharoperbutylacetonicum N1-4_28HMT_29 GCA_000340885.1
724 Clostridium puniceum GCA_002006345.1
ki
757 Rhodobacter sphaeroides 2.4.1 GCA_000012905.2
685 Rhodobacter sphaeroides ATCC 17025 GCA_000016405.1
545 Defluviimonas alba GCA_001620265.1
kj

746 Deinococcus radiodurans R1 GCA_000008565.1
438 Deinococcus deserti VCD115 GCA_000020685.1
422 Deinococcus proteolyticus MRP GCA_000190555.1
kk
491 Streptococcus mutans UA159 GCA_000007465.2
337 Streptococcus macacae NCTC 11558 GCA_000187995.3
323 Streptococcus ratti FA-1 = DSM 20564 GCA_000286075.1
kl
437 Staphylococcus epidermidis ATCC 12228 GCA_000007645.1
425 Staphylococcus hominis subsp. hominis C80 GCA_000183685.1
421 Megasphaera cerevisiae DSM 20462 GCA_001045675.1
421 Staphylococcus warneri SG1 GCA_000332735.1
km
522 Streptococcus mutans UA159 GCA_000007465.2
410 Streptococcus ratti FA-1 = DSM 20564 GCA_000286075.1
390 Streptococcus macacae NCTC 11558 GCA_000187995.3
kn
609 Rhodobacter sphaeroides 2.4.1 GCA_000012905.2
525 Rhodobacter sphaeroides ATCC 17025 GCA_000016405.1
477 Pseudorhodobacter ferrugineus DSM 5888 GCA_000420745.1
ko
761 Streptococcus mutans UA159 GCA_000007465.2
615 Streptococcus gordonii str. Challis substr. CH1 GCA_000017005.1
614 Streptococcus equinus GCA_000964315.1
kp
527 Lactobacillus gasseri ATCC 33323 = JCM 1131 GCA_000014425.1
521 Lactobacillus hominis DSM 23910 = CRBIP 24.179 GCA_000296835.1
391 Deinococcus radiodurans R1 GCA_000008565.1
kq
758 Bacillus anthracis str. Ames GCA_000007845.1
758 Bacillus anthracis str. Sterne GCA_000008165.1
758 _5BBacillus thuringiensis_5D serovar konkukian str. 97-27 GCA_000008505.1
733 Bacillus cereus ATCC 14579 GCA_000007825.1
727 Bacillus pseudomycoides DSM 12442 GCA_000161455.1
kr
710 Clostridium beijerinckii GCA_000833105.2
692 Clostridium saccharoperbutylacetonicum N1-4_28HMT_29 GCA_000340885.1
686 Clostridium puniceum GCA_002006345.1
ks
755 Bacillus cereus ATCC 14579 GCA_000007825.1
754 Bacillus thuringiensis YBT-1518 GCA_000497525.2
753 Bacillus anthracis str. Ames GCA_000007845.1
753 Bacillus anthracis str. Sterne GCA_000008165.1
753 _5BBacillus thuringiensis_5D serovar konkukian str. 97-27 GCA_000008505.1
kt
641 Deinococcus radiodurans R1 GCA_000008565.1
432 Deinococcus soli Cha et al. 2016 GCA_001007995.1
428 Deinococcus deserti VCD115 GCA_000020685.1
ku
492 Clostridium beijerinckii GCA_000833105.2
477 Clostridium saccharoperbutylacetonicum N1-4_28HMT_29 GCA_000340885.1
466 Clostridium puniceum GCA_002006345.1
kv
576 Bacillus thuringiensis YBT-1518 GCA_000497525.2
561 Bacillus cereus ATCC 14579 GCA_000007825.1
558 Bacillus anthracis str. Ames GCA_000007845.1
558 Bacillus anthracis str. Sterne GCA_000008165.1
558 _5BBacillus thuringiensis_5D serovar konkukian str. 97-27 GCA_000008505.1
kw
802 Bifidobacterium adolescentis ATCC 15703 GCA_000010425.1
658 Bifidobacterium angulatum DSM 20098 = JCM 7096 GCA_001025155.1
625 Bifidobacterium dentium JCM 1195 = DSM 20436 GCA_001042595.1
kx
694 Rhodobacter sphaeroides 2.4.1 GCA_000012905.2
605 Rhodobacter sphaeroides ATCC 17025 GCA_000016405.1
511 Gemmobacter aquatilis GCA_900110025.1
ky
617 Staphylococcus epidermidis ATCC 12228 GCA_000007645.1
515 Staphylococcus capitis subsp. capitis GCA_001028645.1
494 Megasphaera cerevisiae DSM 20462 GCA_001045675.1
494 Staphylococcus warneri SG1 GCA_000332735.1
kz
730 Deinococcus radiodurans R1 GCA_000008565.1
498 Deinococcus deserti VCD115 GCA_000020685.1
484 Deinococcus soli Cha et al. 2016 GCA_001007995.1
kA

692 Rhodobacter sphaeroides 2.4.1 GCA_000012905.2
656 Rhodobacter sphaeroides ATCC 17025 GCA_000016405.1
576 Gemmobacter megaterium GCA_900156815.1
kB
620 Bifidobacterium adolescentis ATCC 15703 GCA_000010425.1
468 Bifidobacterium callitrichos DSM 23973 GCA_000741175.1
466 Bifidobacterium dentium JCM 1195 = DSM 20436 GCA_001042595.1
kC
766 Bifidobacterium adolescentis ATCC 15703 GCA_000010425.1
617 Bifidobacterium dentium JCM 1195 = DSM 20436 GCA_001042595.1
600 Bifidobacterium callitrichos DSM 23973 GCA_000741175.1
kD
689 Streptococcus mutans UA159 GCA_000007465.2
500 Streptococcus equinus GCA_000964315.1
496 Streptococcus gallolyticus subsp. gallolyticus DSM 16831 GCA_002000985.1
kE
516 Streptococcus mutans UA159 GCA_000007465.2
361 Streptococcus ratti FA-1 = DSM 20564 GCA_000286075.1
350 Streptococcus sobrinus DSM 20742 = ATCC 33478 GCA_000686605.1
kF
950 Streptococcus mutans UA159 GCA_000007465.2
708 Streptococcus ratti FA-1 = DSM 20564 GCA_000286075.1
667 Streptococcus macacae NCTC 11558 GCA_000187995.3
kG
738 Deinococcus radiodurans R1 GCA_000008565.1
493 Deinococcus deserti VCD115 GCA_000020685.1
479 Deinococcus gobiensis I-0 GCA_000252445.1
kH
679 Enterococcus faecalis V583 GCA_000007785.1
632 Streptomyces cinnamoneus GCA_001885705.1
523 Enterococcus faecium DO GCA_000174395.2
kI
768 Rhodobacter sphaeroides 2.4.1 GCA_000012905.2
721 Rhodobacter sphaeroides ATCC 17025 GCA_000016405.1
604 Pseudorhodobacter psychrotolerans GCA_001294535.1
kJ
496 Streptococcus mutans UA159 GCA_000007465.2
401 Streptococcus ratti FA-1 = DSM 20564 GCA_000286075.1
378 Streptococcus sobrinus DSM 20742 = ATCC 33478 GCA_000686605.1
kK
988 Lactobacillus gasseri ATCC 33323 = JCM 1131 GCA_000014425.1
913 Lactobacillus hominis DSM 23910 = CRBIP 24.179 GCA_000296835.1
721 Lactobacillus iners DSM 13335 GCA_000160875.1
kL
722 Staphylococcus epidermidis ATCC 12228 GCA_000007645.1
681 Staphylococcus capitis subsp. capitis GCA_001028645.1
622 Staphylococcus aureus subsp. aureus NCTC 8325 GCA_000013425.1
kM
740 Clostridium beijerinckii GCA_000833105.2
726 Clostridium saccharoperbutylacetonicum N1-4_28HMT_29 GCA_000340885.1
682 Clostridium puniceum GCA_002006345.1
kN
655 Deinococcus radiodurans R1 GCA_000008565.1
439 Deinococcus deserti VCD115 GCA_000020685.1
432 Deinococcus gobiensis I-0 GCA_000252445.1
kO
348 Bifidobacterium adolescentis ATCC 15703 GCA_000010425.1
283 Bifidobacterium dentium JCM 1195 = DSM 20436 GCA_001042595.1
281 Bifidobacterium lemurum GCA_001895165.1
kP
749 Staphylococcus epidermidis ATCC 12228 GCA_000007645.1
727 Staphylococcus capitis subsp. capitis GCA_001028645.1
664 Staphylococcus aureus subsp. aureus NCTC 8325 GCA_000013425.1
kQ
734 Enterococcus faecalis V583 GCA_000007785.1
687 Streptomyces cinnamoneus GCA_001885705.1
647 Enterococcus rivorum GCA_001742285.1
kR
657 Clostridium beijerinckii GCA_000833105.2
643 Clostridium puniceum GCA_002006345.1
632 Clostridium saccharoperbutylacetonicum N1-4_28HMT_29 GCA_000340885.1
kS
533 Staphylococcus epidermidis ATCC 12228 GCA_000007645.1
493 Megasphaera cerevisiae DSM 20462 GCA_001045675.1
493 Staphylococcus warneri SG1 GCA_000332735.1
480 Staphylococcus aureus subsp. aureus NCTC 8325 GCA_000013425.1

kT
780 Bacillus anthracis str. Ames GCA_000007845.1
780 Bacillus anthracis str. Sterne GCA_000008165.1
780 Bacillus cereus ATCC 14579 GCA_000007825.1
780 Bacillus thuringiensis YBT-1518 GCA_000497525.2
780 _5BBacillus thuringiensis_5D serovar konkukian str. 97-27 GCA_000008505.1
732 Bacillus mycoides GCA_000832605.1
726 Bacillus pseudomycoides DSM 12442 GCA_000161455.1
kU
577 Streptococcus mutans UA159 GCA_000007465.2
446 Streptococcus ratti FA-1 = DSM 20564 GCA_000286075.1
420 Streptococcus gordonii str. Challis substr. CH1 GCA_000017005.1
kV
737 Lactobacillus gasseri ATCC 33323 = JCM 1131 GCA_000014425.1
658 Lactobacillus hominis DSM 23910 = CRBIP 24.179 GCA_000296835.1
461 Lactobacillus iners DSM 13335 GCA_000160875.1
kW
518 Deinococcus radiodurans R1 GCA_000008565.1
310 Deinococcus deserti VCD115 GCA_000020685.1
290 Deinococcus maricopensis DSM 21211 GCA_000186385.1
kX
950 Enterococcus faecalis V583 GCA_000007785.1
903 Streptomyces cinnamoneus GCA_001885705.1
831 Enterococcus canis NBRC 100695 GCA_001544375.1
kY
492 Bifidobacterium adolescentis ATCC 15703 GCA_000010425.1
364 Bifidobacterium angulatum DSM 20098 = JCM 7096 GCA_001025155.1
358 Bifidobacterium breve DSM 20213 = JCM 1192 GCA_001025175.1
kZ
847 Staphylococcus epidermidis ATCC 12228 GCA_000007645.1
811 Staphylococcus capitis subsp. capitis GCA_001028645.1
739 Staphylococcus aureus subsp. aureus NCTC 8325 GCA_000013425.1
l0
636 Rhodobacter sphaeroides 2.4.1 GCA_000012905.2
597 Rhodobacter sphaeroides ATCC 17025 GCA_000016405.1
480 Gemmobacter aquatilis GCA_900110025.1
l1
644 Deinococcus radiodurans R1 GCA_000008565.1
436 Deinococcus deserti VCD115 GCA_000020685.1
404 Deinococcus gobiensis I-0 GCA_000252445.1
l2
628 Rhodobacter sphaeroides 2.4.1 GCA_000012905.2
564 Rhodobacter sphaeroides ATCC 17025 GCA_000016405.1
507 Gemmobacter megaterium GCA_900156815.1
l3
633 Bacillus anthracis str. Ames GCA_000007845.1
633 Bacillus anthracis str. Sterne GCA_000008165.1
633 Bacillus pseudomycoides DSM 12442 GCA_000161455.1
633 _5BBacillus thuringiensis_5D serovar konkukian str. 97-27 GCA_000008505.1
614 Bacillus cereus ATCC 14579 GCA_000007825.1
614 Bacillus thuringiensis YBT-1518 GCA_000497525.2
599 Bacillus mycoides GCA_000832605.1
l4
752 Streptococcus mutans UA159 GCA_000007465.2
584 Streptococcus ratti FA-1 = DSM 20564 GCA_000286075.1
545 Streptococcus ferus DSM 20646 GCA_000372425.1
l5
541 Deinococcus radiodurans R1 GCA_000008565.1
341 Deinococcus hopiensis KR-140 GCA_900176165.1
341 Deinococcus puniceus GCA_001644565.1
332 Deinococcus phoenicis GCA_000599865.1
l6
363 Clostridium beijerinckii GCA_000833105.2
362 Clostridium saccharoperbutylacetonicum N1-4_28HMT_29 GCA_000340885.1
358 Clostridium saccharobutylicum DSM 13864 GCA_000473995.1
l7
415 Rhodobacter sphaeroides 2.4.1 GCA_000012905.2
382 Rhodobacter sphaeroides ATCC 17025 GCA_000016405.1
277 Gemmobacter aquatilis GCA_900110025.1
l8
522 Clostridium beijerinckii GCA_000833105.2
486 Clostridium saccharoperbutylacetonicum N1-4_28HMT_29 GCA_000340885.1
442 Clostridium butyricum GCA_001456065.2
l9
745 Bifidobacterium adolescentis ATCC 15703 GCA_000010425.1
574 Bifidobacterium dentium JCM 1195 = DSM 20436 GCA_001042595.1

535 Bifidobacterium bifidum PRL2010 GCA_000165905.1
la
441 Deinococcus radiodurans R1 GCA_000008565.1
278 Deinococcus soli Cha et al. 2016 GCA_001007995.1
266 Deinococcus deserti VCD115 GCA_000020685.1
lb
814 Escherichia coli str. K-12 substr. MG1655 GCA_000005845.2
791 Escherichia coli IAI39 GCA_000026345.1
780 Escherichia coli O157_3AH7 str. Sakai GCA_000008865.1
780 Shigella flexneri 2a str. 301 GCA_000006925.2
lc
278 Bifidobacterium adolescentis ATCC 15703 GCA_000010425.1
254 Bifidobacterium dentium JCM 1195 = DSM 20436 GCA_001042595.1
247 Bifidobacterium gallicum DSM 20093 = LMG 11596 GCA_000741205.1
ld
679 Staphylococcus epidermidis ATCC 12228 GCA_000007645.1
632 Staphylococcus haemolyticus JCSC1435 GCA_000009865.1
631 Staphylococcus capitis subsp. capitis GCA_001028645.1
le
735 Enterococcus faecalis V583 GCA_000007785.1
688 Streptomyces cinnamoneus GCA_001885705.1
632 Enterococcus faecium DO GCA_000174395.2
lf
671 Lactobacillus gasseri ATCC 33323 = JCM 1131 GCA_000014425.1
613 Lactobacillus hominis DSM 23910 = CRBIP 24.179 GCA_000296835.1
538 Lactobacillus iners DSM 13335 GCA_000160875.1
lg
323 Clostridium beijerinckii GCA_000833105.2
299 Clostridium saccharoperbutylacetonicum N1-4_28HMT_29 GCA_000340885.1
283 Clostridium saccharobutylicum DSM 13864 GCA_000473995.1
lh
738 Clostridium beijerinckii GCA_000833105.2
717 Clostridium saccharoperbutylacetonicum N1-4_28HMT_29 GCA_000340885.1
703 Clostridium puniceum GCA_002006345.1
li
905 Staphylococcus epidermidis ATCC 12228 GCA_000007645.1
860 Staphylococcus capitis subsp. capitis GCA_001028645.1
811 Staphylococcus lugdunensis HKU09-01 GCA_000025085.1
lj
488 Deinococcus radiodurans R1 GCA_000008565.1
281 Deinococcus deserti VCD115 GCA_000020685.1
278 Deinococcus gobiensis I-0 GCA_000252445.1
lk
643 Escherichia coli str. K-12 substr. MG1655 GCA_000005845.2
633 Escherichia coli O157_3AH7 str. Sakai GCA_000008865.1
618 Escherichia coli UMN026 GCA_000026325.2
ll
361 Clostridium beijerinckii GCA_000833105.2
346 Clostridium saccharoperbutylacetonicum N1-4_28HMT_29 GCA_000340885.1
339 Clostridium puniceum GCA_002006345.1
339 Clostridium saccharobutylicum DSM 13864 GCA_000473995.1
lm
640 Streptococcus mutans UA159 GCA_000007465.2
476 Streptococcus ratti FA-1 = DSM 20564 GCA_000286075.1
443 Streptococcus sobrinus DSM 20742 = ATCC 33478 GCA_000686605.1
ln
761 Rhodobacter sphaeroides 2.4.1 GCA_000012905.2
639 Rhodobacter sphaeroides ATCC 17025 GCA_000016405.1
528 Pseudorhodobacter psychrotolerans GCA_001294535.1
lo
504 Clostridium beijerinckii GCA_000833105.2
504 Clostridium saccharoperbutylacetonicum N1-4_28HMT_29 GCA_000340885.1
479 Clostridium butyricum GCA_001456065.2
471 Clostridium saccharobutylicum DSM 13864 GCA_000473995.1
lp
901 Streptococcus mutans UA159 GCA_000007465.2
659 Streptococcus ratti FA-1 = DSM 20564 GCA_000286075.1
625 Streptococcus gallolyticus subsp. gallolyticus DSM 16831 GCA_002000985.1
lq
664 Lactobacillus gasseri ATCC 33323 = JCM 1131 GCA_000014425.1
638 Lactobacillus hominis DSM 23910 = CRBIP 24.179 GCA_000296835.1
444 Lactobacillus psittaci DSM 15354 GCA_000425905.1
lr
600 Staphylococcus epidermidis ATCC 12228 GCA_000007645.1
555 Staphylococcus capitis subsp. capitis GCA_001028645.1
541 Staphylococcus aureus subsp. aureus NCTC 8325 GCA_000013425.1

541 Staphylococcus vitulinus F1028 GCA_000286335.1
ls
677 Rhodobacter sphaeroides 2.4.1 GCA_000012905.2
648 Rhodobacter sphaeroides ATCC 17025 GCA_000016405.1
507 Pseudorhodobacter psychrotolerans GCA_001294535.1
lt
387 Staphylococcus epidermidis ATCC 12228 GCA_000007645.1
379 Staphylococcus capitis subsp. capitis GCA_001028645.1
295 Staphylococcus lugdunensis HKU09-01 GCA_000025085.1
lu
539 Bifidobacterium adolescentis ATCC 15703 GCA_000010425.1
419 Bifidobacterium dentium JCM 1195 = DSM 20436 GCA_001042595.1
415 Bifidobacterium angulatum DSM 20098 = JCM 7096 GCA_001025155.1
lv
924 Enterococcus faecalis V583 GCA_000007785.1
877 Streptomyces cinnamoneus GCA_001885705.1
815 Enterococcus rivorum GCA_001742285.1
lw
562 Lactobacillus gasseri ATCC 33323 = JCM 1131 GCA_000014425.1
505 Lactobacillus hominis DSM 23910 = CRBIP 24.179 GCA_000296835.1
376 Lactobacillus iners DSM 13335 GCA_000160875.1
lx
888 Deinococcus radiodurans R1 GCA_000008565.1
535 Deinococcus deserti VCD115 GCA_000020685.1
498 Deinococcus hopiensis KR-140 GCA_900176165.1
ly
828 Streptococcus mutans UA159 GCA_000007465.2
597 Streptococcus ratti FA-1 = DSM 20564 GCA_000286075.1
554 Streptococcus ferus DSM 20646 GCA_000372425.1
554 Streptococcus gordonii str. Challis substr. CH1 GCA_000017005.1
lz
515 Rhodobacter sphaeroides 2.4.1 GCA_000012905.2
439 Rhodobacter sphaeroides ATCC 17025 GCA_000016405.1
373 Gemmobacter aquatilis GCA_900110025.1
lA
832 Lactobacillus gasseri ATCC 33323 = JCM 1131 GCA_000014425.1
794 Lactobacillus hominis DSM 23910 = CRBIP 24.179 GCA_000296835.1
552 Lactobacillus jensenii GCA_001936235.1
lB
870 Staphylococcus epidermidis ATCC 12228 GCA_000007645.1
813 Staphylococcus capitis subsp. capitis GCA_001028645.1
782 Staphylococcus warneri SG1 GCA_000332735.1
lC
699 Streptococcus mutans UA159 GCA_000007465.2
533 Streptococcus ratti FA-1 = DSM 20564 GCA_000286075.1
494 Streptococcus ferus DSM 20646 GCA_000372425.1
lD
826 Rhodobacter sphaeroides 2.4.1 GCA_000012905.2
732 Rhodobacter sphaeroides ATCC 17025 GCA_000016405.1
650 Pseudorhodobacter ferrugineus DSM 5888 GCA_000420745.1
lE
657 Streptococcus mutans UA159 GCA_000007465.2
480 Streptococcus gordonii str. Challis substr. CH1 GCA_000017005.1
479 Streptococcus ratti FA-1 = DSM 20564 GCA_000286075.1
lF
742 Streptococcus mutans UA159 GCA_000007465.2
527 Streptococcus ratti FA-1 = DSM 20564 GCA_000286075.1
517 Streptococcus gordonii str. Challis substr. CH1 GCA_000017005.1
lG
661 Deinococcus radiodurans R1 GCA_000008565.1
394 Deinococcus deserti VCD115 GCA_000020685.1
381 Deinococcus marmoris DSM 12784 GCA_000701405.1
lH
661 Staphylococcus epidermidis ATCC 12228 GCA_000007645.1
650 Staphylococcus capitis subsp. capitis GCA_001028645.1
606 Staphylococcus hominis subsp. hominis C80 GCA_000183685.1
lI
728 Lactobacillus gasseri ATCC 33323 = JCM 1131 GCA_000014425.1
658 Lactobacillus hominis DSM 23910 = CRBIP 24.179 GCA_000296835.1
525 Lactobacillus iners DSM 13335 GCA_000160875.1
lJ
873 Bacillus anthracis str. Ames GCA_000007845.1
873 Bacillus anthracis str. Sterne GCA_000008165.1
873 _5BBacillus thuringiensis_5D serovar konkukian str. 97-27 GCA_000008505.1
865 Bacillus cereus ATCC 14579 GCA_000007825.1
850 Bacillus thuringiensis YBT-1518 GCA_000497525.2

lK
643 Clostridium beijerinckii GCA_000833105.2
617 Clostridium saccharoperbutylacetonicum N1-4_28HMT_29 GCA_000340885.1
602 Clostridium saccharobutylicum DSM 13864 GCA_000473995.1
lL
552 Clostridium beijerinckii GCA_000833105.2
534 Clostridium saccharoperbutylacetonicum N1-4_28HMT_29 GCA_000340885.1
514 Clostridium butyricum GCA_001456065.2
514 Clostridium saccharobutylicum DSM 13864 GCA_000473995.1
lM
343 Clostridium beijerinckii GCA_000833105.2
328 Clostridium saccharoperbutylacetonicum N1-4_28HMT_29 GCA_000340885.1
323 Clostridium saccharobutylicum DSM 13864 GCA_000473995.1
lN
714 Lactobacillus gasseri ATCC 33323 = JCM 1131 GCA_000014425.1
638 Lactobacillus hominis DSM 23910 = CRBIP 24.179 GCA_000296835.1
463 Lactobacillus iners DSM 13335 GCA_000160875.1
lO
391 Streptococcus mutans UA159 GCA_000007465.2
286 Streptococcus ratti FA-1 = DSM 20564 GCA_000286075.1
279 Streptococcus ferus DSM 20646 GCA_000372425.1
lP
710 Staphylococcus epidermidis ATCC 12228 GCA_000007645.1
676 Staphylococcus capitis subsp. capitis GCA_001028645.1
659 Staphylococcus haemolyticus JCSC1435 GCA_000009865.1
lQ
860 Staphylococcus epidermidis ATCC 12228 GCA_000007645.1
808 Staphylococcus capitis subsp. capitis GCA_001028645.1
800 Staphylococcus aureus subsp. aureus NCTC 8325 GCA_000013425.1
lR
619 Escherichia coli str. K-12 substr. MG1655 GCA_000005845.2
612 Escherichia coli O104_3AH4 str. 2011C-3493 GCA_000299455.1
599 Shigella flexneri 2a str. 301 GCA_000006925.2
lS
686 Escherichia coli str. K-12 substr. MG1655 GCA_000005845.2
650 Escherichia coli UMN026 GCA_000026325.2
638 Shigella flexneri 2a str. 301 GCA_000006925.2
lT
653 Deinococcus radiodurans R1 GCA_000008565.1
369 Deinococcus deserti VCD115 GCA_000020685.1
349 Deinococcus gobiensis I-0 GCA_000252445.1
lU
515 Staphylococcus epidermidis ATCC 12228 GCA_000007645.1
501 Staphylococcus capitis subsp. capitis GCA_001028645.1
470 Staphylococcus simulans GCA_001559115.1
lV
664 Streptococcus mutans UA159 GCA_000007465.2
496 Streptococcus ratti FA-1 = DSM 20564 GCA_000286075.1
478 Streptococcus gordonii str. Challis substr. CH1 GCA_000017005.1
lW
582 Lactobacillus gasseri ATCC 33323 = JCM 1131 GCA_000014425.1
546 Lactobacillus hominis DSM 23910 = CRBIP 24.179 GCA_000296835.1
455 Lactobacillus helveticus GCA_001308285.1
lX
787 Clostridium beijerinckii GCA_000833105.2
735 Clostridium saccharoperbutylacetonicum N1-4_28HMT_29 GCA_000340885.1
718 Clostridium puniceum GCA_002006345.1
lY
769 Streptococcus mutans UA159 GCA_000007465.2
523 Streptococcus gordonii str. Challis substr. CH1 GCA_000017005.1
520 Streptococcus ratti FA-1 = DSM 20564 GCA_000286075.1
lZ
536 Deinococcus radiodurans R1 GCA_000008565.1
348 Deinococcus gobiensis I-0 GCA_000252445.1
340 Deinococcus soli Cha et al. 2016 GCA_001007995.1
m0
651 Streptococcus mutans UA159 GCA_000007465.2
473 Streptococcus ratti FA-1 = DSM 20564 GCA_000286075.1
440 Streptococcus gordonii str. Challis substr. CH1 GCA_000017005.1
m1
786 Enterococcus faecalis V583 GCA_000007785.1
740 Streptomyces cinnamoneus GCA_001885705.1
675 Enterococcus canis NBRC 100695 GCA_001544375.1
m2
765 Lactobacillus gasseri ATCC 33323 = JCM 1131 GCA_000014425.1
684 Lactobacillus hominis DSM 23910 = CRBIP 24.179 GCA_000296835.1

593 Lactobacillus psittaci DSM 15354 GCA_000425905.1
m3
755 Rhodobacter sphaeroides 2.4.1 GCA_000012905.2
662 Rhodobacter sphaeroides ATCC 17025 GCA_000016405.1
552 Gemmobacter aquatilis GCA_900110025.1
m4
388 Deinococcus radiodurans R1 GCA_000008565.1
292 Deinococcus deserti VCD115 GCA_000020685.1
274 Deinococcus gobiensis I-0 GCA_000252445.1
m5
854 Escherichia coli str. K-12 substr. MG1655 GCA_000005845.2
819 Shigella flexneri 2a str. 301 GCA_000006925.2
813 Escherichia coli O104_3AH4 str. 2011C-3493 GCA_000299455.1
m6
525 Deinococcus radiodurans R1 GCA_000008565.1
351 Deinococcus deserti VCD115 GCA_000020685.1
299 Deinococcus hopiensis KR-140 GCA_900176165.1
m7
955 Staphylococcus epidermidis ATCC 12228 GCA_000007645.1
909 Staphylococcus capitis subsp. capitis GCA_001028645.1
832 Staphylococcus aureus subsp. aureus NCTC 8325 GCA_000013425.1
m8
651 Deinococcus radiodurans R1 GCA_000008565.1
462 Deinococcus deserti VCD115 GCA_000020685.1
434 Deinococcus gobiensis I-0 GCA_000252445.1
m9
769 Streptococcus mutans UA159 GCA_000007465.2
598 Streptococcus ratti FA-1 = DSM 20564 GCA_000286075.1
564 Streptococcus sanguinis SK36 GCA_000014205.1
ma
506 Deinococcus radiodurans R1 GCA_000008565.1
300 Deinococcus deserti VCD115 GCA_000020685.1
296 Deinococcus puniceus GCA_001644565.1
mb
901 Bacillus anthracis str. Ames GCA_000007845.1
901 Bacillus anthracis str. Sterne GCA_000008165.1
893 _5BBacillus thuringiensis_5D serovar konkukian str. 97-27 GCA_000008505.1
880 Bacillus cereus ATCC 14579 GCA_000007825.1
880 Bacillus pseudomycoides DSM 12442 GCA_000161455.1
mc
693 Streptococcus mutans UA159 GCA_000007465.2
476 Streptococcus ferus DSM 20646 GCA_000372425.1
476 Streptococcus ratti FA-1 = DSM 20564 GCA_000286075.1
468 Streptococcus gordonii str. Challis substr. CH1 GCA_000017005.1
md
627 Clostridium beijerinckii GCA_000833105.2
609 Clostridium saccharoperbutylacetonicum N1-4_28HMT_29 GCA_000340885.1
572 Clostridium puniceum GCA_002006345.1
me
840 Bifidobacterium adolescentis ATCC 15703 GCA_000010425.1
684 Bifidobacterium dentium JCM 1195 = DSM 20436 GCA_001042595.1
643 Bifidobacterium angulatum DSM 20098 = JCM 7096 GCA_001025155.1
mf
567 Clostridium beijerinckii GCA_000833105.2
542 Clostridium saccharoperbutylacetonicum N1-4_28HMT_29 GCA_000340885.1
529 Clostridium saccharobutylicum DSM 13864 GCA_000473995.1
mg
612 Streptococcus mutans UA159 GCA_000007465.2
457 Streptococcus gordonii str. Challis substr. CH1 GCA_000017005.1
420 Streptococcus iniae GCA_000831485.1
mh
864 Lactobacillus gasseri ATCC 33323 = JCM 1131 GCA_000014425.1
782 Lactobacillus hominis DSM 23910 = CRBIP 24.179 GCA_000296835.1
544 Lactobacillus iners DSM 13335 GCA_000160875.1
mi
699 Clostridium beijerinckii GCA_000833105.2
645 Clostridium saccharoperbutylacetonicum N1-4_28HMT_29 GCA_000340885.1
629 Clostridium puniceum GCA_002006345.1
mj
749 Lactobacillus gasseri ATCC 33323 = JCM 1131 GCA_000014425.1
711 Lactobacillus hominis DSM 23910 = CRBIP 24.179 GCA_000296835.1
508 Lactobacillus iners DSM 13335 GCA_000160875.1
mk
655 Escherichia coli str. K-12 substr. MG1655 GCA_000005845.2
633 Shigella flexneri 2a str. 301 GCA_000006925.2
631 Escherichia coli O104_3AH4 str. 2011C-3493 GCA_000299455.1

631 Escherichia coli O157_3AH7 str. Sakai GCA_000008865.1
ml
569 Clostridium beijerinckii GCA_000833105.2
549 Clostridium saccharoperbutylacetonicum N1-4_28HMT_29 GCA_000340885.1
541 Clostridium saccharobutylicum DSM 13864 GCA_000473995.1
mm
512 Bacillus thuringiensis YBT-1518 GCA_000497525.2
496 Bacillus anthracis str. Ames GCA_000007845.1
496 Bacillus anthracis str. Sterne GCA_000008165.1
496 _5BBacillus thuringiensis_5D serovar konkukian str. 97-27 GCA_000008505.1
493 Bacillus pseudomycoides DSM 12442 GCA_000161455.1
mn
862 Bacillus anthracis str. Ames GCA_000007845.1
862 Bacillus anthracis str. Sterne GCA_000008165.1
862 _5BBacillus thuringiensis_5D serovar konkukian str. 97-27 GCA_000008505.1
851 Bacillus pseudomycoides DSM 12442 GCA_000161455.1
836 Bacillus cereus ATCC 14579 GCA_000007825.1
836 Bacillus thuringiensis YBT-1518 GCA_000497525.2
mo
627 Enterococcus faecalis V583 GCA_000007785.1
587 Streptomyces cinnamoneus GCA_001885705.1
538 Enterococcus massiliensis GCA_001050095.1
mp
596 Deinococcus radiodurans R1 GCA_000008565.1
368 Deinococcus soli Cha et al. 2016 GCA_001007995.1
360 Deinococcus deserti VCD115 GCA_000020685.1
mq
305 Rhodobacter sphaeroides 2.4.1 GCA_000012905.2
276 Rhodobacter sphaeroides ATCC 17025 GCA_000016405.1
255 Rhodobacter vinaykumarii GCA_900156695.1
mr
963 Staphylococcus epidermidis ATCC 12228 GCA_000007645.1
927 Staphylococcus capitis subsp. capitis GCA_001028645.1
845 Megasphaera cerevisiae DSM 20462 GCA_001045675.1
845 Staphylococcus warneri SG1 GCA_000332735.1
ms
714 Rhodobacter sphaeroides 2.4.1 GCA_000012905.2
599 Rhodobacter sphaeroides ATCC 17025 GCA_000016405.1
483 Defluviimonas alba GCA_001620265.1
mt
543 Staphylococcus epidermidis ATCC 12228 GCA_000007645.1
495 Staphylococcus capitis subsp. capitis GCA_001028645.1
477 Megasphaera cerevisiae DSM 20462 GCA_001045675.1
477 Staphylococcus warneri SG1 GCA_000332735.1
mu
739 Bifidobacterium adolescentis ATCC 15703 GCA_000010425.1
557 Bifidobacterium dentium JCM 1195 = DSM 20436 GCA_001042595.1
529 Bifidobacterium longum NCC2705 GCA_000007525.1
mv
866 Streptococcus mutans UA159 GCA_000007465.2
636 Streptococcus ratti FA-1 = DSM 20564 GCA_000286075.1
585 Streptococcus sanguinis SK36 GCA_000014205.1
mw
870 Bifidobacterium adolescentis ATCC 15703 GCA_000010425.1
744 Bifidobacterium dentium JCM 1195 = DSM 20436 GCA_001042595.1
657 Bifidobacterium callitrichos DSM 23973 GCA_000741175.1
mx
598 Rhodobacter sphaeroides 2.4.1 GCA_000012905.2
531 Rhodobacter sphaeroides ATCC 17025 GCA_000016405.1
468 Pseudorhodobacter ferrugineus DSM 5888 GCA_000420745.1
my
815 Staphylococcus epidermidis ATCC 12228 GCA_000007645.1
762 Staphylococcus capitis subsp. capitis GCA_001028645.1
683 Staphylococcus haemolyticus JCSC1435 GCA_000009865.1
mz
582 Streptococcus mutans UA159 GCA_000007465.2
444 Streptococcus ratti FA-1 = DSM 20564 GCA_000286075.1
401 Streptococcus henryi DSM 19005 GCA_000376985.1
mA
530 Streptococcus mutans UA159 GCA_000007465.2
378 Streptococcus ratti FA-1 = DSM 20564 GCA_000286075.1
345 Streptococcus ferus DSM 20646 GCA_000372425.1
mB
768 Clostridium beijerinckii GCA_000833105.2
738 Clostridium saccharoperbutylacetonicum N1-4_28HMT_29 GCA_000340885.1
673 Clostridium puniceum GCA_002006345.1

mC
628 Lactobacillus gasseri ATCC 33323 = JCM 1131 GCA_000014425.1
543 Lactobacillus hominis DSM 23910 = CRBIP 24.179 GCA_000296835.1
389 Lactobacillus iners DSM 13335 GCA_000160875.1
mD
798 Bacillus anthracis str. Ames GCA_000007845.1
798 Bacillus anthracis str. Sterne GCA_000008165.1
798 _5BBacillus thuringiensis_5D serovar konkukian str. 97-27 GCA_000008505.1
778 Bacillus cereus ATCC 14579 GCA_000007825.1
762 Bacillus thuringiensis YBT-1518 GCA_000497525.2
mE
731 Rhodobacter sphaeroides 2.4.1 GCA_000012905.2
609 Rhodobacter sphaeroides ATCC 17025 GCA_000016405.1
537 Gemmobacter megaterium GCA_900156815.1
mF
725 Escherichia coli O157_3AH7 str. Sakai GCA_000008865.1
725 Escherichia coli UMN026 GCA_000026325.2
725 Escherichia coli str. K-12 substr. MG1655 GCA_000005845.2
710 Shigella flexneri 2a str. 301 GCA_000006925.2
705 Escherichia coli O83_3AH1 str. NRG 857C GCA_000183345.1
mG
292 Streptococcus mutans UA159 GCA_000007465.2
248 Streptococcus salivarius GCA_000785515.1
248 Streptococcus thermophilus JIM 8232 GCA_000253395.1
247 Streptococcus criceti HS-6 GCA_000187975.3
247 Streptococcus gordonii str. Challis substr. CH1 GCA_000017005.1
mH
785 Deinococcus radiodurans R1 GCA_000008565.1
532 Deinococcus hopiensis KR-140 GCA_900176165.1
527 Deinococcus soli Cha et al. 2016 GCA_001007995.1
mI
675 Clostridium beijerinckii GCA_000833105.2
629 Clostridium saccharobutylicum DSM 13864 GCA_000473995.1
620 Clostridium saccharoperbutylacetonicum N1-4_28HMT_29 GCA_000340885.1
mJ
705 Staphylococcus epidermidis ATCC 12228 GCA_000007645.1
666 Staphylococcus capitis subsp. capitis GCA_001028645.1
642 Staphylococcus aureus subsp. aureus NCTC 8325 GCA_000013425.1
mK
587 Lactobacillus gasseri ATCC 33323 = JCM 1131 GCA_000014425.1
572 Lactobacillus hominis DSM 23910 = CRBIP 24.179 GCA_000296835.1
414 Lactobacillus jensenii GCA_001936235.1
414 Lactobacillus psittaci DSM 15354 GCA_000425905.1
mL
743 Clostridium beijerinckii GCA_000833105.2
724 Clostridium saccharoperbutylacetonicum N1-4_28HMT_29 GCA_000340885.1
693 Clostridium saccharobutylicum DSM 13864 GCA_000473995.1
mM
576 Rhodobacter sphaeroides 2.4.1 GCA_000012905.2
526 Rhodobacter sphaeroides ATCC 17025 GCA_000016405.1
408 Gemmobacter aquatilis GCA_900110025.1
mN
896 Staphylococcus epidermidis ATCC 12228 GCA_000007645.1
823 Staphylococcus capitis subsp. capitis GCA_001028645.1
759 Staphylococcus aureus subsp. aureus NCTC 8325 GCA_000013425.1
mO
639 Streptococcus mutans UA159 GCA_000007465.2
486 Streptococcus ratti FA-1 = DSM 20564 GCA_000286075.1
461 Streptococcus macacae NCTC 11558 GCA_000187995.3
mP
484 Bifidobacterium adolescentis ATCC 15703 GCA_000010425.1
422 Bifidobacterium dentium JCM 1195 = DSM 20436 GCA_001042595.1
375 Bifidobacterium stellenboschense GCA_000741785.1
mQ
589 Streptococcus mutans UA159 GCA_000007465.2
425 Streptococcus ratti FA-1 = DSM 20564 GCA_000286075.1
403 Streptococcus gordonii str. Challis substr. CH1 GCA_000017005.1
mR
660 Lactobacillus gasseri ATCC 33323 = JCM 1131 GCA_000014425.1
653 Lactobacillus hominis DSM 23910 = CRBIP 24.179 GCA_000296835.1
444 Lactobacillus hamsteri DSM 5661 = JCM 6256 GCA_000615445.1
mS
642 Rhodobacter sphaeroides 2.4.1 GCA_000012905.2
587 Rhodobacter sphaeroides ATCC 17025 GCA_000016405.1
492 Gemmobacter aquatilis GCA_900110025.1
mT

595 Bacillus thuringiensis YBT-1518 GCA_000497525.2
574 Bacillus anthracis str. Ames GCA_000007845.1
574 Bacillus anthracis str. Sterne GCA_000008165.1
574 Bacillus pseudomycoides DSM 12442 GCA_000161455.1
574 _5BBacillus thuringiensis_5D serovar konkukian str. 97-27 GCA_000008505.1
558 Bacillus cereus ATCC 14579 GCA_000007825.1
mU
616 Clostridium beijerinckii GCA_000833105.2
562 Clostridium saccharoperbutylacetonicum N1-4_28HMT_29 GCA_000340885.1
540 Clostridium butyricum GCA_001456065.2
mV
613 Clostridium beijerinckii GCA_000833105.2
595 Clostridium saccharoperbutylacetonicum N1-4_28HMT_29 GCA_000340885.1
580 Clostridium puniceum GCA_002006345.1
mW
585 Lactobacillus gasseri ATCC 33323 = JCM 1131 GCA_000014425.1
558 Lactobacillus hominis DSM 23910 = CRBIP 24.179 GCA_000296835.1
394 Lactobacillus gallinarum GCA_001314245.2
mX
606 Deinococcus radiodurans R1 GCA_000008565.1
414 Deinococcus deserti VCD115 GCA_000020685.1
363 Deinococcus hopiensis KR-140 GCA_900176165.1
mY
755 Clostridium beijerinckii GCA_000833105.2
720 Clostridium saccharoperbutylacetonicum N1-4_28HMT_29 GCA_000340885.1
693 Clostridium saccharobutylicum DSM 13864 GCA_000473995.1
mZ
411 Deinococcus radiodurans R1 GCA_000008565.1
259 Deinococcus deserti VCD115 GCA_000020685.1
236 Deinococcus murrayi DSM 11303 GCA_000482805.1
n0
429 Rhodobacter sphaeroides 2.4.1 GCA_000012905.2
393 Rhodobacter sphaeroides ATCC 17025 GCA_000016405.1
273 Defluviimonas alba GCA_001620265.1
n1
767 Staphylococcus epidermidis ATCC 12228 GCA_000007645.1
712 Staphylococcus capitis subsp. capitis GCA_001028645.1
618 Staphylococcus haemolyticus JCSC1435 GCA_000009865.1
n2
644 Staphylococcus epidermidis ATCC 12228 GCA_000007645.1
613 Staphylococcus capitis subsp. capitis GCA_001028645.1
590 Staphylococcus haemolyticus JCSC1435 GCA_000009865.1
n3
750 Staphylococcus epidermidis ATCC 12228 GCA_000007645.1
710 Staphylococcus capitis subsp. capitis GCA_001028645.1
639 Staphylococcus warneri SG1 GCA_000332735.1
n4
908 Lactobacillus gasseri ATCC 33323 = JCM 1131 GCA_000014425.1
826 Lactobacillus hominis DSM 23910 = CRBIP 24.179 GCA_000296835.1
633 Lactobacillus iners DSM 13335 GCA_000160875.1
n5
487 Enterococcus faecalis V583 GCA_000007785.1
455 Streptomyces cinnamoneus GCA_001885705.1
404 Enterococcus faecium DO GCA_000174395.2
n6
670 Clostridium beijerinckii GCA_000833105.2
610 Clostridium saccharoperbutylacetonicum N1-4_28HMT_29 GCA_000340885.1
600 Clostridium butyricum GCA_001456065.2
n7
558 Staphylococcus epidermidis ATCC 12228 GCA_000007645.1
524 Staphylococcus capitis subsp. capitis GCA_001028645.1
504 Staphylococcus pettenkoferi GCA_002208805.1
n8
649 Clostridium beijerinckii GCA_000833105.2
617 Clostridium saccharoperbutylacetonicum N1-4_28HMT_29 GCA_000340885.1
592 Clostridium puniceum GCA_002006345.1
n9
949 Streptococcus mutans UA159 GCA_000007465.2
707 Streptococcus ratti FA-1 = DSM 20564 GCA_000286075.1
655 Streptococcus gordonii str. Challis substr. CH1 GCA_000017005.1
na
590 Clostridium beijerinckii GCA_000833105.2
584 Clostridium saccharoperbutylacetonicum N1-4_28HMT_29 GCA_000340885.1
581 Clostridium saccharobutylicum DSM 13864 GCA_000473995.1
nb
678 Deinococcus radiodurans R1 GCA_000008565.1

423 Deinococcus deserti VCD115 GCA_000020685.1
409 Deinococcus hopiensis KR-140 GCA_900176165.1
nc
357 Streptococcus mutans UA159 GCA_000007465.2
214 Streptococcus ferus DSM 20646 GCA_000372425.1
199 Streptococcus dysgalactiae subsp. equisimilis AC-2713 GCA_000317855.1
nd
820 Bacillus cereus ATCC 14579 GCA_000007825.1
805 Bacillus anthracis str. Ames GCA_000007845.1
805 Bacillus anthracis str. Sterne GCA_000008165.1
805 Bacillus pseudomycoides DSM 12442 GCA_000161455.1
805 _5BBacillus thuringiensis_5D serovar konkukian str. 97-27 GCA_000008505.1
800 Bacillus mycoides GCA_000832605.1
ne
781 Deinococcus radiodurans R1 GCA_000008565.1
455 Deinococcus deserti VCD115 GCA_000020685.1
449 Deinococcus hopiensis KR-140 GCA_900176165.1
nf
481 Deinococcus radiodurans R1 GCA_000008565.1
309 Deinococcus deserti VCD115 GCA_000020685.1
305 Deinococcus puniceus GCA_001644565.1
ng
498 Staphylococcus epidermidis ATCC 12228 GCA_000007645.1
478 Staphylococcus capitis subsp. capitis GCA_001028645.1
442 Staphylococcus haemolyticus JCSC1435 GCA_000009865.1
442 Staphylococcus hominis subsp. hominis C80 GCA_000183685.1
nh
651 Lactobacillus gasseri ATCC 33323 = JCM 1131 GCA_000014425.1
598 Lactobacillus hominis DSM 23910 = CRBIP 24.179 GCA_000296835.1
466 Lactobacillus iners DSM 13335 GCA_000160875.1
ni
730 Clostridium beijerinckii GCA_000833105.2
720 Clostridium saccharoperbutylacetonicum N1-4_28HMT_29 GCA_000340885.1
702 Clostridium saccharobutylicum DSM 13864 GCA_000473995.1
nj
810 Deinococcus radiodurans R1 GCA_000008565.1
508 Deinococcus deserti VCD115 GCA_000020685.1
504 Deinococcus puniceus GCA_001644565.1
nk
648 Lactobacillus gasseri ATCC 33323 = JCM 1131 GCA_000014425.1
610 Lactobacillus hominis DSM 23910 = CRBIP 24.179 GCA_000296835.1
430 Lactobacillus iners DSM 13335 GCA_000160875.1
nl
603 Lactobacillus gasseri ATCC 33323 = JCM 1131 GCA_000014425.1
555 Lactobacillus hominis DSM 23910 = CRBIP 24.179 GCA_000296835.1
416 Lactobacillus hamsteri DSM 5661 = JCM 6256 GCA_000615445.1
nm
919 Streptococcus mutans UA159 GCA_000007465.2
673 Streptococcus ratti FA-1 = DSM 20564 GCA_000286075.1
619 Streptococcus criceti HS-6 GCA_000187975.3
nn
658 Lactobacillus gasseri ATCC 33323 = JCM 1131 GCA_000014425.1
598 Lactobacillus hominis DSM 23910 = CRBIP 24.179 GCA_000296835.1
468 Lactobacillus iners DSM 13335 GCA_000160875.1
no
641 Rhodobacter sphaeroides 2.4.1 GCA_000012905.2
523 Rhodobacter sphaeroides ATCC 17025 GCA_000016405.1
450 Gemmobacter aquatilis GCA_900110025.1
450 Gemmobacter megaterium GCA_900156815.1
np
642 Lactobacillus gasseri ATCC 33323 = JCM 1131 GCA_000014425.1
601 Lactobacillus hominis DSM 23910 = CRBIP 24.179 GCA_000296835.1
463 Lactobacillus hamsteri DSM 5661 = JCM 6256 GCA_000615445.1
nq
782 Clostridium beijerinckii GCA_000833105.2
738 Clostridium saccharoperbutylacetonicum N1-4_28HMT_29 GCA_000340885.1
735 Clostridium saccharobutylicum DSM 13864 GCA_000473995.1
nr
821 Staphylococcus epidermidis ATCC 12228 GCA_000007645.1
788 Staphylococcus capitis subsp. capitis GCA_001028645.1
698 Staphylococcus lugdunensis HKU09-01 GCA_000025085.1
ns
850 Deinococcus radiodurans R1 GCA_000008565.1
507 Deinococcus gobiensis I-0 GCA_000252445.1
488 Deinococcus proteolyticus MRP GCA_000190555.1
nt

855 Enterococcus faecalis V583 GCA_000007785.1
798 Enterococcus rivorum GCA_001742285.1
793 Streptomyces cinnamoneus GCA_001885705.1
nu
701 Rhodobacter sphaeroides 2.4.1 GCA_000012905.2
597 Rhodobacter sphaeroides ATCC 17025 GCA_000016405.1
476 Pseudorhodobacter ferrugineus DSM 5888 GCA_000420745.1
nv
606 Staphylococcus epidermidis ATCC 12228 GCA_000007645.1
559 Staphylococcus capitis subsp. capitis GCA_001028645.1
533 Staphylococcus hominis subsp. hominis C80 GCA_000183685.1
nw
777 Streptococcus mutans UA159 GCA_000007465.2
511 Streptococcus ratti FA-1 = DSM 20564 GCA_000286075.1
480 Streptococcus gordonii str. Challis substr. CH1 GCA_000017005.1
nx
541 Bifidobacterium adolescentis ATCC 15703 GCA_000010425.1
435 Bifidobacterium dentium JCM 1195 = DSM 20436 GCA_001042595.1
397 Bifidobacterium callitrichos DSM 23973 GCA_000741175.1
ny
518 Enterococcus faecalis V583 GCA_000007785.1
472 Streptomyces cinnamoneus GCA_001885705.1
458 Enterococcus rivorum GCA_001742285.1
nz
442 Escherichia coli str. K-12 substr. MG1655 GCA_000005845.2
412 Escherichia coli O104_3AH4 str. 2011C-3493 GCA_000299455.1
411 Escherichia coli UMN026 GCA_000026325.2
nA
821 Lactobacillus gasseri ATCC 33323 = JCM 1131 GCA_000014425.1
755 Lactobacillus hominis DSM 23910 = CRBIP 24.179 GCA_000296835.1
574 Lactobacillus iners DSM 13335 GCA_000160875.1
nB
818 Clostridium beijerinckii GCA_000833105.2
757 Clostridium saccharoperbutylacetonicum N1-4_28HMT_29 GCA_000340885.1
748 Clostridium saccharobutylicum DSM 13864 GCA_000473995.1
nC
728 Deinococcus radiodurans R1 GCA_000008565.1
440 Deinococcus deserti VCD115 GCA_000020685.1
437 Deinococcus soli Cha et al. 2016 GCA_001007995.1
nD
775 Enterococcus faecalis V583 GCA_000007785.1
732 Enterococcus rivorum GCA_001742285.1
728 Streptomyces cinnamoneus GCA_001885705.1
nE
733 Streptococcus mutans UA159 GCA_000007465.2
556 Streptococcus gordonii str. Challis substr. CH1 GCA_000017005.1
555 Streptococcus pseudoporcinus LQ 940-04 GCA_000188035.3
555 Streptococcus ratti FA-1 = DSM 20564 GCA_000286075.1
nF
764 Rhodobacter sphaeroides 2.4.1 GCA_000012905.2
711 Rhodobacter sphaeroides ATCC 17025 GCA_000016405.1
530 Pseudorhodobacter psychrotolerans GCA_001294535.1
nG
945 Streptococcus mutans UA159 GCA_000007465.2
669 Streptococcus ratti FA-1 = DSM 20564 GCA_000286075.1
661 Streptococcus gordonii str. Challis substr. CH1 GCA_000017005.1
nH
776 Streptococcus mutans UA159 GCA_000007465.2
570 Streptococcus ratti FA-1 = DSM 20564 GCA_000286075.1
536 Streptococcus gallolyticus subsp. gallolyticus DSM 16831 GCA_002000985.1
nI
928 Lactobacillus gasseri ATCC 33323 = JCM 1131 GCA_000014425.1
809 Lactobacillus hominis DSM 23910 = CRBIP 24.179 GCA_000296835.1
626 Lactobacillus iners DSM 13335 GCA_000160875.1
nJ
596 Rhodobacter sphaeroides 2.4.1 GCA_000012905.2
542 Rhodobacter sphaeroides ATCC 17025 GCA_000016405.1
484 Gemmobacter aquatilis GCA_900110025.1
nK
649 Bacillus anthracis str. Ames GCA_000007845.1
649 Bacillus anthracis str. Sterne GCA_000008165.1
649 _5BBacillus thuringiensis_5D serovar konkukian str. 97-27 GCA_000008505.1
641 Bacillus pseudomycoides DSM 12442 GCA_000161455.1
639 Bacillus thuringiensis YBT-1518 GCA_000497525.2
nL
788 Lactobacillus gasseri ATCC 33323 = JCM 1131 GCA_000014425.1

757 Lactobacillus hominis DSM 23910 = CRBIP 24.179 GCA_000296835.1
582 Lactobacillus iners DSM 13335 GCA_000160875.1
nM
769 Escherichia coli str. K-12 substr. MG1655 GCA_000005845.2
754 Escherichia coli O157_3AH7 str. Sakai GCA_000008865.1
754 Shigella flexneri 2a str. 301 GCA_000006925.2
751 Escherichia coli UMN026 GCA_000026325.2
nN
619 Escherichia coli O157_3AH7 str. Sakai GCA_000008865.1
619 Escherichia coli str. K-12 substr. MG1655 GCA_000005845.2
604 Shigella flexneri 2a str. 301 GCA_000006925.2
602 Escherichia coli UMN026 GCA_000026325.2
nO
696 Deinococcus radiodurans R1 GCA_000008565.1
492 Deinococcus deserti VCD115 GCA_000020685.1
467 Deinococcus gobiensis I-0 GCA_000252445.1
nP
733 Staphylococcus epidermidis ATCC 12228 GCA_000007645.1
668 Staphylococcus capitis subsp. capitis GCA_001028645.1
626 Staphylococcus aureus subsp. aureus NCTC 8325 GCA_000013425.1
nQ
613 Lactobacillus gasseri ATCC 33323 = JCM 1131 GCA_000014425.1
590 Lactobacillus hominis DSM 23910 = CRBIP 24.179 GCA_000296835.1
402 Lactobacillus iners DSM 13335 GCA_000160875.1
nR
718 Lactobacillus gasseri ATCC 33323 = JCM 1131 GCA_000014425.1
670 Lactobacillus hominis DSM 23910 = CRBIP 24.179 GCA_000296835.1
479 Lactobacillus jensenii GCA_001936235.1
479 Lactobacillus psittaci DSM 15354 GCA_000425905.1
nS
481 Staphylococcus epidermidis ATCC 12228 GCA_000007645.1
449 Staphylococcus capitis subsp. capitis GCA_001028645.1
424 Staphylococcus aureus subsp. aureus NCTC 8325 GCA_000013425.1
nT
859 Clostridium beijerinckii GCA_000833105.2
848 Clostridium saccharoperbutylacetonicum N1-4_28HMT_29 GCA_000340885.1
817 Clostridium saccharobutylicum DSM 13864 GCA_000473995.1
nU
601 Clostridium beijerinckii GCA_000833105.2
550 Clostridium saccharoperbutylacetonicum N1-4_28HMT_29 GCA_000340885.1
528 Clostridium chromiireducens GCA_002029255.1
nV
582 Clostridium saccharoperbutylacetonicum N1-4_28HMT_29 GCA_000340885.1
571 Clostridium beijerinckii GCA_000833105.2
570 Clostridium saccharobutylicum DSM 13864 GCA_000473995.1
nW
641 Clostridium beijerinckii GCA_000833105.2
639 Clostridium saccharoperbutylacetonicum N1-4_28HMT_29 GCA_000340885.1
622 Clostridium puniceum GCA_002006345.1
622 Clostridium saccharobutylicum DSM 13864 GCA_000473995.1
nX
779 Deinococcus radiodurans R1 GCA_000008565.1
466 Deinococcus deserti VCD115 GCA_000020685.1
412 Deinococcus proteolyticus MRP GCA_000190555.1
nY
711 Deinococcus radiodurans R1 GCA_000008565.1
412 Deinococcus deserti VCD115 GCA_000020685.1
393 Deinococcus soli Cha et al. 2016 GCA_001007995.1
nZ
739 Escherichia coli str. K-12 substr. MG1655 GCA_000005845.2
733 Escherichia coli IAI39 GCA_000026345.1
733 Shigella flexneri 2a str. 301 GCA_000006925.2
717 Escherichia coli O104_3AH4 str. 2011C-3493 GCA_000299455.1
717 Escherichia coli O157_3AH7 str. Sakai GCA_000008865.1
o0
863 Clostridium beijerinckii GCA_000833105.2
859 Clostridium saccharoperbutylacetonicum N1-4_28HMT_29 GCA_000340885.1
846 Clostridium puniceum GCA_002006345.1
o1
516 Clostridium beijerinckii GCA_000833105.2
515 Clostridium saccharoperbutylacetonicum N1-4_28HMT_29 GCA_000340885.1
496 Clostridium butyricum GCA_001456065.2
o2
518 Staphylococcus epidermidis ATCC 12228 GCA_000007645.1
488 Staphylococcus capitis subsp. capitis GCA_001028645.1
488 Staphylococcus haemolyticus JCSC1435 GCA_000009865.1

487 Staphylococcus hominis subsp. hominis C80 GCA_000183685.1
o3
807 Enterococcus faecalis V583 GCA_000007785.1
760 Streptomyces cinnamoneus GCA_001885705.1
719 Enterococcus massiliensis GCA_001050095.1
o4
519 Rhodobacter sphaeroides 2.4.1 GCA_000012905.2
504 Rhodobacter sphaeroides ATCC 17025 GCA_000016405.1
441 Pseudorhodobacter psychrotolerans GCA_001294535.1
o5
585 Escherichia coli str. K-12 substr. MG1655 GCA_000005845.2
580 Escherichia coli O157_3AH7 str. Sakai GCA_000008865.1
580 Shigella flexneri 2a str. 301 GCA_000006925.2
579 Escherichia coli O83_3AH1 str. NRG 857C GCA_000183345.1
579 Escherichia coli UMN026 GCA_000026325.2
o6
742 Deinococcus radiodurans R1 GCA_000008565.1
485 Deinococcus murrayi DSM 11303 GCA_000482805.1
480 Deinococcus deserti VCD115 GCA_000020685.1
o7
615 Staphylococcus epidermidis ATCC 12228 GCA_000007645.1
564 Staphylococcus capitis subsp. capitis GCA_001028645.1
556 Staphylococcus sciuri GCA_002209165.1
556 Staphylococcus warneri SG1 GCA_000332735.1
o8
726 Lactobacillus gasseri ATCC 33323 = JCM 1131 GCA_000014425.1
652 Lactobacillus hominis DSM 23910 = CRBIP 24.179 GCA_000296835.1
464 Lactobacillus iners DSM 13335 GCA_000160875.1
o9
720 Streptococcus mutans UA159 GCA_000007465.2
564 Streptococcus ratti FA-1 = DSM 20564 GCA_000286075.1
517 Streptococcus equinus GCA_000964315.1
517 Streptococcus gallolyticus subsp. gallolyticus DSM 16831 GCA_002000985.1
oa
815 Lactobacillus gasseri ATCC 33323 = JCM 1131 GCA_000014425.1
773 Lactobacillus hominis DSM 23910 = CRBIP 24.179 GCA_000296835.1
592 Lactobacillus hamsteri DSM 5661 = JCM 6256 GCA_000615445.1
ob
671 Escherichia coli O157_3AH7 str. Sakai GCA_000008865.1
671 Escherichia coli str. K-12 substr. MG1655 GCA_000005845.2
660 Escherichia coli O104_3AH4 str. 2011C-3493 GCA_000299455.1
660 Shigella flexneri 2a str. 301 GCA_000006925.2
651 Escherichia coli O83_3AH1 str. NRG 857C GCA_000183345.1
oc
570 Clostridium beijerinckii GCA_000833105.2
555 Clostridium saccharoperbutylacetonicum N1-4_28HMT_29 GCA_000340885.1
533 Clostridium saccharobutylicum DSM 13864 GCA_000473995.1
od
725 Lactobacillus gasseri ATCC 33323 = JCM 1131 GCA_000014425.1
660 Lactobacillus hominis DSM 23910 = CRBIP 24.179 GCA_000296835.1
478 Lactobacillus acetotolerans GCA_001042405.1
oe
769 Enterococcus faecalis V583 GCA_000007785.1
722 Streptomyces cinnamoneus GCA_001885705.1
703 Enterococcus phoeniculicola ATCC BAA-412 GCA_000407505.1
of
657 Lactobacillus gasseri ATCC 33323 = JCM 1131 GCA_000014425.1
576 Lactobacillus hominis DSM 23910 = CRBIP 24.179 GCA_000296835.1
441 Lactobacillus iners DSM 13335 GCA_000160875.1
og
792 Lactobacillus gasseri ATCC 33323 = JCM 1131 GCA_000014425.1
698 Lactobacillus hominis DSM 23910 = CRBIP 24.179 GCA_000296835.1
538 Lactobacillus iners DSM 13335 GCA_000160875.1
oh
626 Rhodobacter sphaeroides 2.4.1 GCA_000012905.2
554 Rhodobacter sphaeroides ATCC 17025 GCA_000016405.1
460 Gemmobacter megaterium GCA_900156815.1
oi
417 Rhodobacter sphaeroides 2.4.1 GCA_000012905.2
390 Rhodobacter sphaeroides ATCC 17025 GCA_000016405.1
283 Pseudorhodobacter wandonensis GCA_001202035.1
oj
838 Lactobacillus gasseri ATCC 33323 = JCM 1131 GCA_000014425.1
763 Lactobacillus hominis DSM 23910 = CRBIP 24.179 GCA_000296835.1
574 Lactobacillus iners DSM 13335 GCA_000160875.1
ok

695 Clostridium beijerinckii GCA_000833105.2
664 Clostridium saccharoperbutylacetonicum N1-4_28HMT_29 GCA_000340885.1
621 Clostridium puniceum GCA_002006345.1
ol
541 Streptococcus mutans UA159 GCA_000007465.2
371 Streptococcus ratti FA-1 = DSM 20564 GCA_000286075.1
335 Streptococcus gallolyticus subsp. gallolyticus DSM 16831 GCA_002000985.1
om
706 Clostridium beijerinckii GCA_000833105.2
668 Clostridium saccharobutylicum DSM 13864 GCA_000473995.1
665 Clostridium saccharoperbutylacetonicum N1-4_28HMT_29 GCA_000340885.1
on
775 Lactobacillus gasseri ATCC 33323 = JCM 1131 GCA_000014425.1
710 Lactobacillus hominis DSM 23910 = CRBIP 24.179 GCA_000296835.1
542 Lactobacillus iners DSM 13335 GCA_000160875.1
oo
691 Deinococcus radiodurans R1 GCA_000008565.1
423 Deinococcus deserti VCD115 GCA_000020685.1
390 Deinococcus hopiensis KR-140 GCA_900176165.1
op
822 Clostridium saccharoperbutylacetonicum N1-4_28HMT_29 GCA_000340885.1
817 Clostridium beijerinckii GCA_000833105.2
789 Clostridium saccharobutylicum DSM 13864 GCA_000473995.1
oq
513 Staphylococcus capitis subsp. capitis GCA_001028645.1
513 Staphylococcus epidermidis ATCC 12228 GCA_000007645.1
485 Megasphaera cerevisiae DSM 20462 GCA_001045675.1
485 Staphylococcus warneri SG1 GCA_000332735.1
458 Staphylococcus saprophyticus subsp. saprophyticus ATCC 15305 GCA_000010125.1
458 Staphylococcus xylosus GCA_000706685.1
or
1047 Lactobacillus gasseri ATCC 33323 = JCM 1131 GCA_000014425.1
930 Lactobacillus hominis DSM 23910 = CRBIP 24.179 GCA_000296835.1
692 Lactobacillus iners DSM 13335 GCA_000160875.1
os
717 Streptococcus mutans UA159 GCA_000007465.2
488 Streptococcus gordonii str. Challis substr. CH1 GCA_000017005.1
468 Streptococcus ferus DSM 20646 GCA_000372425.1
ot
527 Streptococcus mutans UA159 GCA_000007465.2
374 Streptococcus ratti FA-1 = DSM 20564 GCA_000286075.1
360 Streptococcus ovis DSM 16829 GCA_000380125.1
ou
706 Rhodobacter sphaeroides 2.4.1 GCA_000012905.2
598 Rhodobacter sphaeroides ATCC 17025 GCA_000016405.1
482 Gemmobacter aquatilis GCA_900110025.1
ov
666 Escherichia coli O104_3AH4 str. 2011C-3493 GCA_000299455.1
666 Escherichia coli str. K-12 substr. MG1655 GCA_000005845.2
659 Shigella flexneri 2a str. 301 GCA_000006925.2
646 Escherichia coli O157_3AH7 str. Sakai GCA_000008865.1
ow
621 Streptococcus mutans UA159 GCA_000007465.2
371 Streptococcus gordonii str. Challis substr. CH1 GCA_000017005.1
369 Streptococcus equinus GCA_000964315.1
ox
825 Staphylococcus epidermidis ATCC 12228 GCA_000007645.1
798 Staphylococcus capitis subsp. capitis GCA_001028645.1
704 Staphylococcus haemolyticus JCSC1435 GCA_000009865.1
704 Staphylococcus hominis subsp. hominis C80 GCA_000183685.1
oy
609 Staphylococcus epidermidis ATCC 12228 GCA_000007645.1
568 Staphylococcus capitis subsp. capitis GCA_001028645.1
524 Staphylococcus lutrae GCA_002101335.1
oz
705 Enterococcus faecalis V583 GCA_000007785.1
660 Streptomyces cinnamoneus GCA_001885705.1
623 Enterococcus rivorum GCA_001742285.1
oA
606 Bifidobacterium adolescentis ATCC 15703 GCA_000010425.1
576 Bifidobacterium dentium JCM 1195 = DSM 20436 GCA_001042595.1
539 Bifidobacterium angulatum DSM 20098 = JCM 7096 GCA_001025155.1
539 Bifidobacterium longum NCC2705 GCA_000007525.1
oB
644 Streptococcus mutans UA159 GCA_000007465.2
427 Streptococcus ratti FA-1 = DSM 20564 GCA_000286075.1

390 Streptococcus equinus GCA_000964315.1
oC
604 Streptococcus mutans UA159 GCA_000007465.2
500 Streptococcus ratti FA-1 = DSM 20564 GCA_000286075.1
481 Streptococcus macacae NCTC 11558 GCA_000187995.3
oD
687 Clostridium beijerinckii GCA_000833105.2
630 Clostridium saccharoperbutylacetonicum N1-4_28HMT_29 GCA_000340885.1
622 Clostridium puniceum GCA_002006345.1
oE
566 Streptococcus mutans UA159 GCA_000007465.2
386 Streptococcus gordonii str. Challis substr. CH1 GCA_000017005.1
371 Streptococcus ratti FA-1 = DSM 20564 GCA_000286075.1
oF
732 Lactobacillus gasseri ATCC 33323 = JCM 1131 GCA_000014425.1
666 Lactobacillus hominis DSM 23910 = CRBIP 24.179 GCA_000296835.1
486 Lactobacillus iners DSM 13335 GCA_000160875.1
oG
682 Deinococcus radiodurans R1 GCA_000008565.1
441 Deinococcus gobiensis I-0 GCA_000252445.1
440 Deinococcus deserti VCD115 GCA_000020685.1
oH
700 Lactobacillus gasseri ATCC 33323 = JCM 1131 GCA_000014425.1
632 Lactobacillus hominis DSM 23910 = CRBIP 24.179 GCA_000296835.1
471 Lactobacillus iners DSM 13335 GCA_000160875.1
oI
508 Staphylococcus epidermidis ATCC 12228 GCA_000007645.1
490 Staphylococcus capitis subsp. capitis GCA_001028645.1
435 Megasphaera cerevisiae DSM 20462 GCA_001045675.1
435 Staphylococcus warneri SG1 GCA_000332735.1
oJ
889 Deinococcus radiodurans R1 GCA_000008565.1
524 Deinococcus deserti VCD115 GCA_000020685.1
513 Deinococcus gobiensis I-0 GCA_000252445.1
oK
777 Clostridium beijerinckii GCA_000833105.2
768 Clostridium saccharoperbutylacetonicum N1-4_28HMT_29 GCA_000340885.1
744 Clostridium saccharobutylicum DSM 13864 GCA_000473995.1
oL
730 Enterococcus faecalis V583 GCA_000007785.1
683 Streptomyces cinnamoneus GCA_001885705.1
645 Enterococcus massiliensis GCA_001050095.1
oM
805 Streptococcus mutans UA159 GCA_000007465.2
575 Streptococcus ratti FA-1 = DSM 20564 GCA_000286075.1
527 Streptococcus halotolerans GCA_001598035.1
oN
724 Streptococcus mutans UA159 GCA_000007465.2
554 Streptococcus ratti FA-1 = DSM 20564 GCA_000286075.1
507 Streptococcus equinus GCA_000964315.1
oO
609 Streptococcus mutans UA159 GCA_000007465.2
464 Streptococcus ratti FA-1 = DSM 20564 GCA_000286075.1
422 Streptococcus macacae NCTC 11558 GCA_000187995.3
oP
798 Escherichia coli str. K-12 substr. MG1655 GCA_000005845.2
766 Escherichia coli O104_3AH4 str. 2011C-3493 GCA_000299455.1
766 Shigella flexneri 2a str. 301 GCA_000006925.2
740 Escherichia coli O157_3AH7 str. Sakai GCA_000008865.1
740 Escherichia coli O83_3AH1 str. NRG 857C GCA_000183345.1
oQ
456 Streptococcus mutans UA159 GCA_000007465.2
269 Streptococcus gordonii str. Challis substr. CH1 GCA_000017005.1
237 Streptococcus ratti FA-1 = DSM 20564 GCA_000286075.1
oR
416 Bifidobacterium adolescentis ATCC 15703 GCA_000010425.1
377 Bifidobacterium callitrichos DSM 23973 GCA_000741175.1
372 Bifidobacterium gallicum DSM 20093 = LMG 11596 GCA_000741205.1
oS
570 Enterococcus faecalis V583 GCA_000007785.1
527 Enterococcus haemoperoxidus ATCC BAA-382 GCA_000407165.1
527 Enterococcus rivorum GCA_001742285.1
524 Streptomyces cinnamoneus GCA_001885705.1
oT
777 Clostridium beijerinckii GCA_000833105.2
758 Clostridium saccharoperbutylacetonicum N1-4_28HMT_29 GCA_000340885.1

734 Clostridium saccharobutylicum DSM 13864 GCA_000473995.1
oU
660 Enterococcus faecalis V583 GCA_000007785.1
599 Streptomyces cinnamoneus GCA_001885705.1
537 Enterococcus haemoperoxidus ATCC BAA-382 GCA_000407165.1
oV
434 Escherichia coli O104_3AH4 str. 2011C-3493 GCA_000299455.1
434 Escherichia coli O157_3AH7 str. Sakai GCA_000008865.1
434 Escherichia coli str. K-12 substr. MG1655 GCA_000005845.2
434 Shigella flexneri 2a str. 301 GCA_000006925.2
433 Escherichia coli O83_3AH1 str. NRG 857C GCA_000183345.1
433 Escherichia coli UMN026 GCA_000026325.2
411 Escherichia coli IAI39 GCA_000026345.1
411 Shigella dysenteriae Sd197 GCA_000012005.1
oW
548 Clostridium saccharobutylicum DSM 13864 GCA_000473995.1
548 Clostridium saccharoperbutylacetonicum N1-4_28HMT_29 GCA_000340885.1
543 Clostridium beijerinckii GCA_000833105.2
531 Clostridium puniceum GCA_002006345.1
oX
746 Clostridium beijerinckii GCA_000833105.2
727 Clostridium saccharoperbutylacetonicum N1-4_28HMT_29 GCA_000340885.1
710 Clostridium saccharobutylicum DSM 13864 GCA_000473995.1
oY
517 Deinococcus radiodurans R1 GCA_000008565.1
332 Deinococcus gobiensis I-0 GCA_000252445.1
323 Deinococcus deserti VCD115 GCA_000020685.1
oZ
589 Bifidobacterium adolescentis ATCC 15703 GCA_000010425.1
523 Bifidobacterium dentium JCM 1195 = DSM 20436 GCA_001042595.1
450 Bifidobacterium tsurumiense GCA_000741765.1
p0
674 Staphylococcus epidermidis ATCC 12228 GCA_000007645.1
654 Staphylococcus capitis subsp. capitis GCA_001028645.1
619 Megasphaera cerevisiae DSM 20462 GCA_001045675.1
619 Staphylococcus warneri SG1 GCA_000332735.1
p1
383 Rhodobacter sphaeroides 2.4.1 GCA_000012905.2
359 Rhodobacter sphaeroides ATCC 17025 GCA_000016405.1
287 Gemmobacter aquatilis GCA_900110025.1
p2
650 Staphylococcus epidermidis ATCC 12228 GCA_000007645.1
624 Staphylococcus capitis subsp. capitis GCA_001028645.1
606 Staphylococcus hominis subsp. hominis C80 GCA_000183685.1
p3
653 Enterococcus faecalis V583 GCA_000007785.1
648 Streptomyces cinnamoneus GCA_001885705.1
596 Enterococcus saccharolyticus subsp. saccharolyticus ATCC 43076 GCA_000407285.1
p4
637 Enterococcus faecalis V583 GCA_000007785.1
590 Streptomyces cinnamoneus GCA_001885705.1
507 Enterococcus rivorum GCA_001742285.1
p5
725 Lactobacillus gasseri ATCC 33323 = JCM 1131 GCA_000014425.1
689 Lactobacillus hominis DSM 23910 = CRBIP 24.179 GCA_000296835.1
554 Lactobacillus iners DSM 13335 GCA_000160875.1
p6
601 Clostridium beijerinckii GCA_000833105.2
555 Clostridium saccharoperbutylacetonicum N1-4_28HMT_29 GCA_000340885.1
541 Clostridium chromiireducens GCA_002029255.1
p7
417 Enterococcus faecalis V583 GCA_000007785.1
387 Streptomyces cinnamoneus GCA_001885705.1
386 Enterococcus asini ATCC 700915 GCA_000407365.1
p8
493 Streptococcus mutans UA159 GCA_000007465.2
352 Streptococcus gallolyticus subsp. gallolyticus DSM 16831 GCA_002000985.1
350 Streptococcus equinus GCA_000964315.1
p9
619 Streptococcus mutans UA159 GCA_000007465.2
418 Streptococcus ratti FA-1 = DSM 20564 GCA_000286075.1
384 Streptococcus gordonii str. Challis substr. CH1 GCA_000017005.1
pa
815 Escherichia coli str. K-12 substr. MG1655 GCA_000005845.2
814 Escherichia coli O157_3AH7 str. Sakai GCA_000008865.1
806 Escherichia coli UMN026 GCA_000026325.2

pb
442 Deinococcus radiodurans R1 GCA_000008565.1
296 Deinococcus deserti VCD115 GCA_000020685.1
260 Deinococcus hopiensis KR-140 GCA_900176165.1
pc
884 Staphylococcus epidermidis ATCC 12228 GCA_000007645.1
825 Staphylococcus capitis subsp. capitis GCA_001028645.1
749 Megasphaera cerevisiae DSM 20462 GCA_001045675.1
749 Staphylococcus warneri SG1 GCA_000332735.1
pd
753 Deinococcus radiodurans R1 GCA_000008565.1
438 Deinococcus deserti VCD115 GCA_000020685.1
425 Deinococcus soli Cha et al. 2016 GCA_001007995.1
pe
761 Clostridium beijerinckii GCA_000833105.2
749 Clostridium saccharoperbutylacetonicum N1-4_28HMT_29 GCA_000340885.1
725 Clostridium puniceum GCA_002006345.1
pf
603 Streptococcus mutans UA159 GCA_000007465.2
398 Streptococcus gordonii str. Challis substr. CH1 GCA_000017005.1
395 Streptococcus ratti FA-1 = DSM 20564 GCA_000286075.1
pg
500 Streptococcus mutans UA159 GCA_000007465.2
353 Streptococcus ratti FA-1 = DSM 20564 GCA_000286075.1
334 Streptococcus macacae NCTC 11558 GCA_000187995.3
ph
795 Escherichia coli O104_3AH4 str. 2011C-3493 GCA_000299455.1
795 Escherichia coli str. K-12 substr. MG1655 GCA_000005845.2
771 Shigella flexneri 2a str. 301 GCA_000006925.2
746 Escherichia coli UMN026 GCA_000026325.2
pi
748 Rhodobacter sphaeroides 2.4.1 GCA_000012905.2
647 Rhodobacter sphaeroides ATCC 17025 GCA_000016405.1
529 Gemmobacter aquatilis GCA_900110025.1
pj
480 Rhodobacter sphaeroides 2.4.1 GCA_000012905.2
449 Rhodobacter sphaeroides ATCC 17025 GCA_000016405.1
358 Pseudorhodobacter ferrugineus DSM 5888 GCA_000420745.1
pk
789 Streptococcus mutans UA159 GCA_000007465.2
585 Streptococcus ratti FA-1 = DSM 20564 GCA_000286075.1
544 Streptococcus ferus DSM 20646 GCA_000372425.1
pl
731 Streptococcus mutans UA159 GCA_000007465.2
556 Streptococcus ratti FA-1 = DSM 20564 GCA_000286075.1
484 Streptococcus ferus DSM 20646 GCA_000372425.1
pm
893 Lactobacillus gasseri ATCC 33323 = JCM 1131 GCA_000014425.1
827 Lactobacillus hominis DSM 23910 = CRBIP 24.179 GCA_000296835.1
615 Lactobacillus psittaci DSM 15354 GCA_000425905.1
pn
568 Deinococcus radiodurans R1 GCA_000008565.1
426 Deinococcus gobiensis I-0 GCA_000252445.1
416 Deinococcus deserti VCD115 GCA_000020685.1
po
448 Streptococcus mutans UA159 GCA_000007465.2
406 Streptomyces cinnamoneus GCA_001885705.1
388 Enterococcus faecalis V583 GCA_000007785.1
pp
688 Staphylococcus epidermidis ATCC 12228 GCA_000007645.1
652 Staphylococcus capitis subsp. capitis GCA_001028645.1
592 Staphylococcus lugdunensis HKU09-01 GCA_000025085.1
pq
720 Lactobacillus gasseri ATCC 33323 = JCM 1131 GCA_000014425.1
633 Lactobacillus hominis DSM 23910 = CRBIP 24.179 GCA_000296835.1
457 Lactobacillus iners DSM 13335 GCA_000160875.1
pr
664 Deinococcus radiodurans R1 GCA_000008565.1
438 Deinococcus deserti VCD115 GCA_000020685.1
399 Deinococcus hopiensis KR-140 GCA_900176165.1
ps
619 Clostridium beijerinckii GCA_000833105.2
603 Clostridium saccharoperbutylacetonicum N1-4_28HMT_29 GCA_000340885.1
594 Clostridium saccharobutylicum DSM 13864 GCA_000473995.1
pt
465 Enterococcus faecalis V583 GCA_000007785.1

419 Enterococcus thailandicus GCA_001652875.1
419 Streptomyces cinnamoneus GCA_001885705.1
412 Enterococcus rivorum GCA_001742285.1
pu
887 Staphylococcus epidermidis ATCC 12228 GCA_000007645.1
859 Staphylococcus capitis subsp. capitis GCA_001028645.1
788 Staphylococcus hominis subsp. hominis C80 GCA_000183685.1
pv
430 Streptococcus mutans UA159 GCA_000007465.2
270 Streptococcus sobrinus DSM 20742 = ATCC 33478 GCA_000686605.1
259 Streptococcus equinus GCA_000964315.1
259 Streptococcus ratti FA-1 = DSM 20564 GCA_000286075.1
pw
404 Streptococcus mutans UA159 GCA_000007465.2
254 Streptococcus gordonii str. Challis substr. CH1 GCA_000017005.1
236 Streptococcus orisratti DSM 15617 GCA_000380105.1
px
363 Enterococcus faecalis V583 GCA_000007785.1
328 Enterococcus rivorum GCA_001742285.1
320 Streptomyces cinnamoneus GCA_001885705.1
py
724 Clostridium beijerinckii GCA_000833105.2
707 Clostridium saccharoperbutylacetonicum N1-4_28HMT_29 GCA_000340885.1
696 Clostridium saccharobutylicum DSM 13864 GCA_000473995.1
pz
735 Staphylococcus epidermidis ATCC 12228 GCA_000007645.1
681 Staphylococcus haemolyticus JCSC1435 GCA_000009865.1
673 Staphylococcus capitis subsp. capitis GCA_001028645.1
pA
720 Clostridium beijerinckii GCA_000833105.2
684 Clostridium saccharoperbutylacetonicum N1-4_28HMT_29 GCA_000340885.1
661 Clostridium butyricum GCA_001456065.2
pB
751 Staphylococcus epidermidis ATCC 12228 GCA_000007645.1
733 Staphylococcus capitis subsp. capitis GCA_001028645.1
688 Staphylococcus hominis subsp. hominis C80 GCA_000183685.1
pC
495 Rhodobacter sphaeroides 2.4.1 GCA_000012905.2
457 Rhodobacter sphaeroides ATCC 17025 GCA_000016405.1
350 Gemmobacter aquatilis GCA_900110025.1
pD
784 Lactobacillus gasseri ATCC 33323 = JCM 1131 GCA_000014425.1
738 Lactobacillus hominis DSM 23910 = CRBIP 24.179 GCA_000296835.1
556 Lactobacillus iners DSM 13335 GCA_000160875.1
pE
506 Deinococcus radiodurans R1 GCA_000008565.1
280 Deinococcus deserti VCD115 GCA_000020685.1
261 Deinococcus gobiensis I-0 GCA_000252445.1
pF
625 Clostridium beijerinckii GCA_000833105.2
600 Clostridium saccharoperbutylacetonicum N1-4_28HMT_29 GCA_000340885.1
559 Clostridium puniceum GCA_002006345.1
pG
496 Clostridium beijerinckii GCA_000833105.2
481 Clostridium puniceum GCA_002006345.1
481 Clostridium saccharoperbutylacetonicum N1-4_28HMT_29 GCA_000340885.1
468 Clostridium saccharobutylicum DSM 13864 GCA_000473995.1
pH
647 Rhodobacter sphaeroides 2.4.1 GCA_000012905.2
564 Rhodobacter sphaeroides ATCC 17025 GCA_000016405.1
468 Defluviimonas alba GCA_001620265.1
pI
801 Bacillus anthracis str. Ames GCA_000007845.1
801 Bacillus anthracis str. Sterne GCA_000008165.1
801 Bacillus cereus ATCC 14579 GCA_000007825.1
801 _5BBacillus thuringiensis_5D serovar konkukian str. 97-27 GCA_000008505.1
787 Bacillus pseudomycoides DSM 12442 GCA_000161455.1
783 Bacillus thuringiensis YBT-1518 GCA_000497525.2
pJ
495 Escherichia coli str. K-12 substr. MG1655 GCA_000005845.2
484 Escherichia coli O104_3AH4 str. 2011C-3493 GCA_000299455.1
469 Escherichia coli O83_3AH1 str. NRG 857C GCA_000183345.1
pK
740 Enterococcus faecalis V583 GCA_000007785.1
693 Streptomyces cinnamoneus GCA_001885705.1
616 Enterococcus rivorum GCA_001742285.1

pL
660 Enterococcus faecalis V583 GCA_000007785.1
613 Streptomyces cinnamoneus GCA_001885705.1
538 Enterococcus rivorum GCA_001742285.1
pM
694 Clostridium beijerinckii GCA_000833105.2
628 Clostridium saccharoperbutylacetonicum N1-4_28HMT_29 GCA_000340885.1
618 Clostridium puniceum GCA_002006345.1
pN
849 Rhodobacter sphaeroides 2.4.1 GCA_000012905.2
772 Rhodobacter sphaeroides ATCC 17025 GCA_000016405.1
640 Defluviimonas alba GCA_001620265.1
pO
748 Bacillus anthracis str. Ames GCA_000007845.1
748 Bacillus anthracis str. Sterne GCA_000008165.1
740 Bacillus cereus ATCC 14579 GCA_000007825.1
740 _5BBacillus thuringiensis_5D serovar konkukian str. 97-27 GCA_000008505.1
722 Bacillus pseudomycoides DSM 12442 GCA_000161455.1
pP
705 Shigella flexneri 2a str. 301 GCA_000006925.2
698 Escherichia coli O104_3AH4 str. 2011C-3493 GCA_000299455.1
698 Escherichia coli str. K-12 substr. MG1655 GCA_000005845.2
658 Escherichia coli O83_3AH1 str. NRG 857C GCA_000183345.1
pQ
712 Deinococcus radiodurans R1 GCA_000008565.1
497 Deinococcus gobiensis I-0 GCA_000252445.1
491 Deinococcus puniceus GCA_001644565.1
pR
827 Streptococcus mutans UA159 GCA_000007465.2
652 Streptococcus gordonii str. Challis substr. CH1 GCA_000017005.1
625 Streptococcus cristatus AS 1.3089 GCA_000385925.1
pS
703 Clostridium saccharoperbutylacetonicum N1-4_28HMT_29 GCA_000340885.1
699 Clostridium beijerinckii GCA_000833105.2
675 Clostridium saccharobutylicum DSM 13864 GCA_000473995.1
pT
631 Deinococcus radiodurans R1 GCA_000008565.1
445 Deinococcus gobiensis I-0 GCA_000252445.1
435 Deinococcus deserti VCD115 GCA_000020685.1
pU
591 Clostridium beijerinckii GCA_000833105.2
563 Clostridium puniceum GCA_002006345.1
563 Clostridium saccharoperbutylacetonicum N1-4_28HMT_29 GCA_000340885.1
541 Clostridium saccharobutylicum DSM 13864 GCA_000473995.1
pV
674 Bifidobacterium adolescentis ATCC 15703 GCA_000010425.1
570 Bifidobacterium dentium JCM 1195 = DSM 20436 GCA_001042595.1
533 Bifidobacterium angulatum DSM 20098 = JCM 7096 GCA_001025155.1
pW
760 Bacillus anthracis str. Ames GCA_000007845.1
760 Bacillus anthracis str. Sterne GCA_000008165.1
759 _5BBacillus thuringiensis_5D serovar konkukian str. 97-27 GCA_000008505.1
746 Bacillus cereus ATCC 14579 GCA_000007825.1
746 Bacillus thuringiensis YBT-1518 GCA_000497525.2
pX
640 Clostridium beijerinckii GCA_000833105.2
608 Clostridium saccharoperbutylacetonicum N1-4_28HMT_29 GCA_000340885.1
584 Clostridium saccharobutylicum DSM 13864 GCA_000473995.1
pY
768 Rhodobacter sphaeroides 2.4.1 GCA_000012905.2
698 Rhodobacter sphaeroides ATCC 17025 GCA_000016405.1
546 Gemmobacter aquatilis GCA_900110025.1
pZ
382 Clostridium beijerinckii GCA_000833105.2
349 Clostridium puniceum GCA_002006345.1
349 Clostridium saccharoperbutylacetonicum N1-4_28HMT_29 GCA_000340885.1
334 Clostridium saccharobutylicum DSM 13864 GCA_000473995.1
q0
883 Streptococcus mutans UA159 GCA_000007465.2
660 Streptococcus ratti FA-1 = DSM 20564 GCA_000286075.1
650 Streptococcus gordonii str. Challis substr. CH1 GCA_000017005.1
q1
486 Clostridium beijerinckii GCA_000833105.2
484 Clostridium puniceum GCA_002006345.1
470 Clostridium saccharoperbutylacetonicum N1-4_28HMT_29 GCA_000340885.1
q2

610 Staphylococcus epidermidis ATCC 12228 GCA_000007645.1
560 Staphylococcus capitis subsp. capitis GCA_001028645.1
548 Staphylococcus condimenti GCA_001618885.1
q3
537 Staphylococcus epidermidis ATCC 12228 GCA_000007645.1
507 Staphylococcus capitis subsp. capitis GCA_001028645.1
466 Megasphaera cerevisiae DSM 20462 GCA_001045675.1
466 Staphylococcus pettenkoferi GCA_002208805.1
466 Staphylococcus warneri SG1 GCA_000332735.1
q4
785 Clostridium beijerinckii GCA_000833105.2
747 Clostridium saccharoperbutylacetonicum N1-4_28HMT_29 GCA_000340885.1
739 Clostridium saccharobutylicum DSM 13864 GCA_000473995.1
q5
519 Clostridium beijerinckii GCA_000833105.2
470 Clostridium saccharoperbutylacetonicum N1-4_28HMT_29 GCA_000340885.1
464 Clostridium puniceum GCA_002006345.1
q6
723 Staphylococcus epidermidis ATCC 12228 GCA_000007645.1
696 Staphylococcus capitis subsp. capitis GCA_001028645.1
675 Staphylococcus aureus subsp. aureus NCTC 8325 GCA_000013425.1
q7
614 Streptococcus mutans UA159 GCA_000007465.2
400 Streptococcus macacae NCTC 11558 GCA_000187995.3
384 Streptococcus ratti FA-1 = DSM 20564 GCA_000286075.1
q8
754 Rhodobacter sphaeroides 2.4.1 GCA_000012905.2
729 Rhodobacter sphaeroides ATCC 17025 GCA_000016405.1
621 Pseudorhodobacter ferrugineus DSM 5888 GCA_000420745.1
q9
678 Enterococcus faecalis V583 GCA_000007785.1
631 Streptomyces cinnamoneus GCA_001885705.1
606 Enterococcus rivorum GCA_001742285.1
qa
565 Lactobacillus gasseri ATCC 33323 = JCM 1131 GCA_000014425.1
486 Lactobacillus hominis DSM 23910 = CRBIP 24.179 GCA_000296835.1
292 Lactobacillus acidophilus NCFM GCA_000011985.1
292 Lactobacillus gallinarum GCA_001314245.2
qb
636 Rhodobacter sphaeroides 2.4.1 GCA_000012905.2
573 Rhodobacter sphaeroides ATCC 17025 GCA_000016405.1
491 Pseudorhodobacter ferrugineus DSM 5888 GCA_000420745.1
qc
587 Staphylococcus epidermidis ATCC 12228 GCA_000007645.1
572 Staphylococcus capitis subsp. capitis GCA_001028645.1
564 Megasphaera cerevisiae DSM 20462 GCA_001045675.1
564 Staphylococcus aureus subsp. aureus NCTC 8325 GCA_000013425.1
564 Staphylococcus warneri SG1 GCA_000332735.1
qd
660 Lactobacillus gasseri ATCC 33323 = JCM 1131 GCA_000014425.1
601 Lactobacillus hominis DSM 23910 = CRBIP 24.179 GCA_000296835.1
479 Lactobacillus iners DSM 13335 GCA_000160875.1
qe
774 Staphylococcus epidermidis ATCC 12228 GCA_000007645.1
708 Staphylococcus capitis subsp. capitis GCA_001028645.1
673 Staphylococcus warneri SG1 GCA_000332735.1
qf
785 Streptococcus mutans UA159 GCA_000007465.2
579 Streptococcus ratti FA-1 = DSM 20564 GCA_000286075.1
535 Streptococcus criceti HS-6 GCA_000187975.3
qg
803 Bacillus anthracis str. Ames GCA_000007845.1
803 Bacillus anthracis str. Sterne GCA_000008165.1
803 Bacillus cereus ATCC 14579 GCA_000007825.1
803 Bacillus thuringiensis YBT-1518 GCA_000497525.2
803 _5BBacillus thuringiensis_5D serovar konkukian str. 97-27 GCA_000008505.1
776 Bacillus mycoides GCA_000832605.1
771 Bacillus pseudomycoides DSM 12442 GCA_000161455.1
qh
756 Rhodobacter sphaeroides 2.4.1 GCA_000012905.2
620 Rhodobacter sphaeroides ATCC 17025 GCA_000016405.1
508 Defluviimonas alba GCA_001620265.1
qi
558 Staphylococcus epidermidis ATCC 12228 GCA_000007645.1
531 Staphylococcus capitis subsp. capitis GCA_001028645.1
505 Staphylococcus haemolyticus JCSC1435 GCA_000009865.1

qj
521 Bifidobacterium adolescentis ATCC 15703 GCA_000010425.1
473 Bifidobacterium dentium JCM 1195 = DSM 20436 GCA_001042595.1
435 Bifidobacterium stellenboschense GCA_000741785.1
qk
705 Bifidobacterium adolescentis ATCC 15703 GCA_000010425.1
594 Bifidobacterium dentium JCM 1195 = DSM 20436 GCA_001042595.1
525 Bifidobacterium bifidum PRL2010 GCA_000165905.1
ql
576 Rhodobacter sphaeroides 2.4.1 GCA_000012905.2
543 Rhodobacter sphaeroides ATCC 17025 GCA_000016405.1
420 Gemmobacter aquatilis GCA_900110025.1
qm
532 Streptococcus mutans UA159 GCA_000007465.2
350 Streptococcus halotolerans GCA_001598035.1
337 Streptococcus ratti FA-1 = DSM 20564 GCA_000286075.1
qn
485 Clostridium beijerinckii GCA_000833105.2
470 Clostridium saccharoperbutylacetonicum N1-4_28HMT_29 GCA_000340885.1
465 Clostridium saccharobutylicum DSM 13864 GCA_000473995.1
qo
521 Escherichia coli O104_3AH4 str. 2011C-3493 GCA_000299455.1
520 Escherichia coli O157_3AH7 str. Sakai GCA_000008865.1
520 Escherichia coli str. K-12 substr. MG1655 GCA_000005845.2
520 Shigella flexneri 2a str. 301 GCA_000006925.2
503 Escherichia coli O83_3AH1 str. NRG 857C GCA_000183345.1
503 Escherichia coli UMN026 GCA_000026325.2
qp
531 Lactobacillus gasseri ATCC 33323 = JCM 1131 GCA_000014425.1
497 Lactobacillus hominis DSM 23910 = CRBIP 24.179 GCA_000296835.1
372 Lactobacillus psittaci DSM 15354 GCA_000425905.1
qq
610 Rhodobacter sphaeroides 2.4.1 GCA_000012905.2
516 Rhodobacter sphaeroides ATCC 17025 GCA_000016405.1
462 Defluviimonas alba GCA_001620265.1
qr
627 Streptococcus mutans UA159 GCA_000007465.2
454 Streptococcus gordonii str. Challis substr. CH1 GCA_000017005.1
429 Streptococcus macacae NCTC 11558 GCA_000187995.3
qs
698 Enterococcus faecalis V583 GCA_000007785.1
658 Streptomyces cinnamoneus GCA_001885705.1
593 Enterococcus asini ATCC 700915 GCA_000407365.1
qt
736 Rhodobacter sphaeroides 2.4.1 GCA_000012905.2
671 Rhodobacter sphaeroides ATCC 17025 GCA_000016405.1
563 Gemmobacter megaterium GCA_900156815.1
qu
852 Enterococcus faecalis V583 GCA_000007785.1
805 Streptomyces cinnamoneus GCA_001885705.1
729 Enterococcus rivorum GCA_001742285.1
qv
657 Deinococcus radiodurans R1 GCA_000008565.1
406 Deinococcus deserti VCD115 GCA_000020685.1
378 Deinococcus proteolyticus MRP GCA_000190555.1
qw
722 Rhodobacter sphaeroides 2.4.1 GCA_000012905.2
695 Rhodobacter sphaeroides ATCC 17025 GCA_000016405.1
564 Defluviimonas alba GCA_001620265.1
qx
793 Staphylococcus epidermidis ATCC 12228 GCA_000007645.1
783 Staphylococcus capitis subsp. capitis GCA_001028645.1
743 Staphylococcus warneri SG1 GCA_000332735.1
qy
721 Streptococcus mutans UA159 GCA_000007465.2
544 Streptococcus ratti FA-1 = DSM 20564 GCA_000286075.1
486 Streptococcus sobrinus DSM 20742 = ATCC 33478 GCA_000686605.1
qz
978 Streptococcus mutans UA159 GCA_000007465.2
734 Streptococcus ratti FA-1 = DSM 20564 GCA_000286075.1
720 Streptococcus gordonii str. Challis substr. CH1 GCA_000017005.1
qA
443 Escherichia coli O157_3AH7 str. Sakai GCA_000008865.1
443 Escherichia coli str. K-12 substr. MG1655 GCA_000005845.2
441 Escherichia coli UMN026 GCA_000026325.2
428 Escherichia coli O104_3AH4 str. 2011C-3493 GCA_000299455.1

428 Shigella flexneri 2a str. 301 GCA_000006925.2
qB
675 Lactobacillus gasseri ATCC 33323 = JCM 1131 GCA_000014425.1
642 Lactobacillus hominis DSM 23910 = CRBIP 24.179 GCA_000296835.1
474 Lactobacillus iners DSM 13335 GCA_000160875.1
qC
736 Clostridium beijerinckii GCA_000833105.2
710 Clostridium saccharoperbutylacetonicum N1-4_28HMT_29 GCA_000340885.1
708 Clostridium saccharobutylicum DSM 13864 GCA_000473995.1
qD
461 Staphylococcus epidermidis ATCC 12228 GCA_000007645.1
445 Staphylococcus capitis subsp. capitis GCA_001028645.1
407 Megasphaera cerevisiae DSM 20462 GCA_001045675.1
407 Staphylococcus warneri SG1 GCA_000332735.1
qE
627 Escherichia coli O157_3AH7 str. Sakai GCA_000008865.1
627 Escherichia coli UMN026 GCA_000026325.2
627 Escherichia coli str. K-12 substr. MG1655 GCA_000005845.2
627 Shigella flexneri 2a str. 301 GCA_000006925.2
621 Escherichia coli O104_3AH4 str. 2011C-3493 GCA_000299455.1
621 Escherichia coli O83_3AH1 str. NRG 857C GCA_000183345.1
582 Klebsiella oxytoca GCA_001022195.1
qF
587 Rhodobacter sphaeroides 2.4.1 GCA_000012905.2
580 Rhodobacter sphaeroides ATCC 17025 GCA_000016405.1
447 Defluviimonas alba GCA_001620265.1
qG
693 Deinococcus radiodurans R1 GCA_000008565.1
434 Deinococcus deserti VCD115 GCA_000020685.1
417 Deinococcus hopiensis KR-140 GCA_900176165.1
qH
649 Bacillus cereus ATCC 14579 GCA_000007825.1
643 Bacillus anthracis str. Ames GCA_000007845.1
643 Bacillus anthracis str. Sterne GCA_000008165.1
643 _5BBacillus thuringiensis_5D serovar konkukian str. 97-27 GCA_000008505.1
634 Bacillus pseudomycoides DSM 12442 GCA_000161455.1
qI
649 Staphylococcus epidermidis ATCC 12228 GCA_000007645.1
590 Staphylococcus capitis subsp. capitis GCA_001028645.1
545 Staphylococcus haemolyticus JCSC1435 GCA_000009865.1
545 Staphylococcus hominis subsp. hominis C80 GCA_000183685.1
qJ
590 Lactobacillus gasseri ATCC 33323 = JCM 1131 GCA_000014425.1
552 Lactobacillus hominis DSM 23910 = CRBIP 24.179 GCA_000296835.1
476 Streptococcus mutans UA159 GCA_000007465.2
qK
487 Enterococcus faecalis V583 GCA_000007785.1
441 Streptomyces cinnamoneus GCA_001885705.1
414 Enterococcus rivorum GCA_001742285.1
qL
697 Rhodobacter sphaeroides 2.4.1 GCA_000012905.2
644 Rhodobacter sphaeroides ATCC 17025 GCA_000016405.1
569 Gemmobacter aquatilis GCA_900110025.1
qM
438 Lactobacillus gasseri ATCC 33323 = JCM 1131 GCA_000014425.1
415 Lactobacillus hominis DSM 23910 = CRBIP 24.179 GCA_000296835.1
287 Lactobacillus iners DSM 13335 GCA_000160875.1
qN
471 Enterococcus faecalis V583 GCA_000007785.1
425 Streptomyces cinnamoneus GCA_001885705.1
401 Enterococcus asini ATCC 700915 GCA_000407365.1
401 Enterococcus massiliensis GCA_001050095.1
401 Enterococcus saccharolyticus subsp. saccharolyticus ATCC 43076 GCA_000407285.1
qO
617 Escherichia coli IAI39 GCA_000026345.1
617 Escherichia coli O83_3AH1 str. NRG 857C GCA_000183345.1
608 Escherichia coli O157_3AH7 str. Sakai GCA_000008865.1
608 Escherichia coli str. K-12 substr. MG1655 GCA_000005845.2
608 Shigella flexneri 2a str. 301 GCA_000006925.2
605 Escherichia coli O104_3AH4 str. 2011C-3493 GCA_000299455.1
qP
651 Staphylococcus epidermidis ATCC 12228 GCA_000007645.1
588 Staphylococcus capitis subsp. capitis GCA_001028645.1
571 Staphylococcus haemolyticus JCSC1435 GCA_000009865.1
qQ
693 Enterococcus faecalis V583 GCA_000007785.1

647 Streptomyces cinnamoneus GCA_001885705.1
589 Vagococcus lutrae LBD1 GCA_000498295.1
qR
794 Rhodobacter sphaeroides 2.4.1 GCA_000012905.2
694 Rhodobacter sphaeroides ATCC 17025 GCA_000016405.1
571 Gemmobacter aquatilis GCA_900110025.1
qS
791 Staphylococcus epidermidis ATCC 12228 GCA_000007645.1
752 Staphylococcus capitis subsp. capitis GCA_001028645.1
748 Staphylococcus hominis subsp. hominis C80 GCA_000183685.1
qT
751 Rhodobacter sphaeroides 2.4.1 GCA_000012905.2
693 Rhodobacter sphaeroides ATCC 17025 GCA_000016405.1
547 Pseudorhodobacter psychrotolerans GCA_001294535.1
qU
737 Streptococcus mutans UA159 GCA_000007465.2
561 Streptococcus ratti FA-1 = DSM 20564 GCA_000286075.1
517 Streptococcus gordonii str. Challis substr. CH1 GCA_000017005.1
qV
573 Streptococcus mutans UA159 GCA_000007465.2
430 Streptococcus ratti FA-1 = DSM 20564 GCA_000286075.1
396 Streptococcus gordonii str. Challis substr. CH1 GCA_000017005.1
qW
560 Streptococcus mutans UA159 GCA_000007465.2
350 Streptococcus gordonii str. Challis substr. CH1 GCA_000017005.1
344 Streptococcus ratti FA-1 = DSM 20564 GCA_000286075.1
qX
820 Clostridium beijerinckii GCA_000833105.2
812 Clostridium saccharobutylicum DSM 13864 GCA_000473995.1
806 Clostridium saccharoperbutylacetonicum N1-4_28HMT_29 GCA_000340885.1
qY
797 Enterococcus faecalis V583 GCA_000007785.1
750 Streptomyces cinnamoneus GCA_001885705.1
729 Enterococcus rivorum GCA_001742285.1
qZ
690 Lactobacillus gasseri ATCC 33323 = JCM 1131 GCA_000014425.1
667 Lactobacillus hominis DSM 23910 = CRBIP 24.179 GCA_000296835.1
488 Lactobacillus jensenii GCA_001936235.1
488 Lactobacillus psittaci DSM 15354 GCA_000425905.1
r0
678 Enterococcus faecalis V583 GCA_000007785.1
631 Streptomyces cinnamoneus GCA_001885705.1
624 Enterococcus rivorum GCA_001742285.1
r1
630 Bacillus anthracis str. Ames GCA_000007845.1
630 Bacillus anthracis str. Sterne GCA_000008165.1
630 Bacillus cereus ATCC 14579 GCA_000007825.1
630 _5BBacillus thuringiensis_5D serovar konkukian str. 97-27 GCA_000008505.1
623 Bacillus mycoides GCA_000832605.1
618 Bacillus thuringiensis YBT-1518 GCA_000497525.2
r2
729 Streptococcus mutans UA159 GCA_000007465.2
573 Streptococcus ratti FA-1 = DSM 20564 GCA_000286075.1
528 Streptococcus gordonii str. Challis substr. CH1 GCA_000017005.1
r3
815 Rhodobacter sphaeroides 2.4.1 GCA_000012905.2
732 Rhodobacter sphaeroides ATCC 17025 GCA_000016405.1
617 Gemmobacter aquatilis GCA_900110025.1
r4
857 Clostridium beijerinckii GCA_000833105.2
842 Clostridium saccharoperbutylacetonicum N1-4_28HMT_29 GCA_000340885.1
832 Clostridium saccharobutylicum DSM 13864 GCA_000473995.1
r5
661 Bacillus anthracis str. Ames GCA_000007845.1
661 Bacillus anthracis str. Sterne GCA_000008165.1
661 _5BBacillus thuringiensis_5D serovar konkukian str. 97-27 GCA_000008505.1
657 Bacillus thuringiensis YBT-1518 GCA_000497525.2
644 Bacillus cereus ATCC 14579 GCA_000007825.1
r6
981 Lactobacillus gasseri ATCC 33323 = JCM 1131 GCA_000014425.1
899 Lactobacillus hominis DSM 23910 = CRBIP 24.179 GCA_000296835.1
671 Lactobacillus iners DSM 13335 GCA_000160875.1
r7
760 Deinococcus radiodurans R1 GCA_000008565.1
511 Deinococcus deserti VCD115 GCA_000020685.1
494 Deinococcus murrayi DSM 11303 GCA_000482805.1

r8
801 Clostridium beijerinckii GCA_000833105.2
759 Clostridium saccharoperbutylacetonicum N1-4_28HMT_29 GCA_000340885.1
730 Clostridium saccharobutylicum DSM 13864 GCA_000473995.1
r9
696 Deinococcus radiodurans R1 GCA_000008565.1
472 Deinococcus gobiensis I-0 GCA_000252445.1
457 Deinococcus deserti VCD115 GCA_000020685.1
ra
766 Lactobacillus gasseri ATCC 33323 = JCM 1131 GCA_000014425.1
704 Lactobacillus hominis DSM 23910 = CRBIP 24.179 GCA_000296835.1
590 Lactobacillus iners DSM 13335 GCA_000160875.1
rb
426 Clostridium beijerinckii GCA_000833105.2
396 Clostridium saccharoperbutylacetonicum N1-4_28HMT_29 GCA_000340885.1
374 Clostridium puniceum GCA_002006345.1
rc
759 Clostridium beijerinckii GCA_000833105.2
709 Clostridium saccharoperbutylacetonicum N1-4_28HMT_29 GCA_000340885.1
680 Clostridium puniceum GCA_002006345.1
rd
627 Deinococcus radiodurans R1 GCA_000008565.1
312 Deinococcus deserti VCD115 GCA_000020685.1
302 Deinococcus gobiensis I-0 GCA_000252445.1
re
670 Clostridium beijerinckii GCA_000833105.2
651 Clostridium saccharoperbutylacetonicum N1-4_28HMT_29 GCA_000340885.1
614 Clostridium saccharobutylicum DSM 13864 GCA_000473995.1
rf
641 Rhodobacter sphaeroides 2.4.1 GCA_000012905.2
585 Rhodobacter sphaeroides ATCC 17025 GCA_000016405.1
451 Rhodobacter aestuarii GCA_900156655.1
rg
779 Lactobacillus gasseri ATCC 33323 = JCM 1131 GCA_000014425.1
714 Lactobacillus hominis DSM 23910 = CRBIP 24.179 GCA_000296835.1
503 Lactobacillus iners DSM 13335 GCA_000160875.1
rh
641 Staphylococcus epidermidis ATCC 12228 GCA_000007645.1
600 Staphylococcus capitis subsp. capitis GCA_001028645.1
591 Megasphaera cerevisiae DSM 20462 GCA_001045675.1
591 Staphylococcus aureus subsp. aureus NCTC 8325 GCA_000013425.1
591 Staphylococcus haemolyticus JCSC1435 GCA_000009865.1
591 Staphylococcus hominis subsp. hominis C80 GCA_000183685.1
591 Staphylococcus warneri SG1 GCA_000332735.1
ri
719 Clostridium beijerinckii GCA_000833105.2
691 Clostridium saccharoperbutylacetonicum N1-4_28HMT_29 GCA_000340885.1
666 Clostridium puniceum GCA_002006345.1
rj
769 Bifidobacterium adolescentis ATCC 15703 GCA_000010425.1
630 Bifidobacterium dentium JCM 1195 = DSM 20436 GCA_001042595.1
576 Bifidobacterium callitrichos DSM 23973 GCA_000741175.1
rk
942 Bacillus anthracis str. Ames GCA_000007845.1
942 Bacillus anthracis str. Sterne GCA_000008165.1
935 _5BBacillus thuringiensis_5D serovar konkukian str. 97-27 GCA_000008505.1
930 Bacillus pseudomycoides DSM 12442 GCA_000161455.1
rl
675 Escherichia coli str. K-12 substr. MG1655 GCA_000005845.2
644 Escherichia coli O104_3AH4 str. 2011C-3493 GCA_000299455.1
644 Shigella flexneri 2a str. 301 GCA_000006925.2
616 Escherichia coli O157_3AH7 str. Sakai GCA_000008865.1
rm
711 Bifidobacterium adolescentis ATCC 15703 GCA_000010425.1
608 Bifidobacterium dentium JCM 1195 = DSM 20436 GCA_001042595.1
580 Bifidobacterium thermophilum GCA_000741495.1
580 Bifidobacterium thermophilum RBL67 GCA_000347695.1
rn
613 Clostridium beijerinckii GCA_000833105.2
552 Clostridium saccharoperbutylacetonicum N1-4_28HMT_29 GCA_000340885.1
536 Clostridium butyricum GCA_001456065.2
ro
850 Staphylococcus epidermidis ATCC 12228 GCA_000007645.1
803 Staphylococcus capitis subsp. capitis GCA_001028645.1
731 Staphylococcus haemolyticus JCSC1435 GCA_000009865.1
rp

751 Rhodobacter sphaeroides 2.4.1 GCA_000012905.2
642 Rhodobacter sphaeroides ATCC 17025 GCA_000016405.1
558 Gemmobacter aquatilis GCA_900110025.1
rq
979 Lactobacillus gasseri ATCC 33323 = JCM 1131 GCA_000014425.1
909 Lactobacillus hominis DSM 23910 = CRBIP 24.179 GCA_000296835.1
730 Lactobacillus iners DSM 13335 GCA_000160875.1
rr
778 Clostridium saccharoperbutylacetonicum N1-4_28HMT_29 GCA_000340885.1
767 Clostridium beijerinckii GCA_000833105.2
757 Clostridium saccharobutylicum DSM 13864 GCA_000473995.1
rs
775 Bifidobacterium adolescentis ATCC 15703 GCA_000010425.1
659 Bifidobacterium callitrichos DSM 23973 GCA_000741175.1
653 Bifidobacterium angulatum DSM 20098 = JCM 7096 GCA_001025155.1
rt
460 Clostridium beijerinckii GCA_000833105.2
456 Clostridium saccharoperbutylacetonicum N1-4_28HMT_29 GCA_000340885.1
440 Clostridium puniceum GCA_002006345.1
ru
663 Clostridium beijerinckii GCA_000833105.2
640 Clostridium saccharoperbutylacetonicum N1-4_28HMT_29 GCA_000340885.1
613 Clostridium puniceum GCA_002006345.1
rv
661 Staphylococcus epidermidis ATCC 12228 GCA_000007645.1
655 Staphylococcus capitis subsp. capitis GCA_001028645.1
614 Staphylococcus haemolyticus JCSC1435 GCA_000009865.1
614 Staphylococcus hominis subsp. hominis C80 GCA_000183685.1
rw
613 Rhodobacter sphaeroides 2.4.1 GCA_000012905.2
555 Rhodobacter sphaeroides ATCC 17025 GCA_000016405.1
486 Gemmobacter aquatilis GCA_900110025.1
rx
535 Staphylococcus epidermidis ATCC 12228 GCA_000007645.1
533 Staphylococcus capitis subsp. capitis GCA_001028645.1
481 Staphylococcus pettenkoferi GCA_002208805.1
ry
637 Clostridium beijerinckii GCA_000833105.2
630 Clostridium saccharoperbutylacetonicum N1-4_28HMT_29 GCA_000340885.1
623 Clostridium puniceum GCA_002006345.1
rz
401 Escherichia coli str. K-12 substr. MG1655 GCA_000005845.2
386 Escherichia coli O104_3AH4 str. 2011C-3493 GCA_000299455.1
386 Shigella flexneri 2a str. 301 GCA_000006925.2
378 Escherichia coli O157_3AH7 str. Sakai GCA_000008865.1
378 Escherichia coli UMN026 GCA_000026325.2
rA
818 Clostridium beijerinckii GCA_000833105.2
793 Clostridium saccharoperbutylacetonicum N1-4_28HMT_29 GCA_000340885.1
763 Clostridium puniceum GCA_002006345.1
rB
764 Deinococcus radiodurans R1 GCA_000008565.1
510 Deinococcus deserti VCD115 GCA_000020685.1
486 Deinococcus gobiensis I-0 GCA_000252445.1
rC
802 Escherichia coli O157_3AH7 str. Sakai GCA_000008865.1
802 Escherichia coli str. K-12 substr. MG1655 GCA_000005845.2
800 Shigella flexneri 2a str. 301 GCA_000006925.2
799 Escherichia coli UMN026 GCA_000026325.2
rD
638 Lactobacillus gasseri ATCC 33323 = JCM 1131 GCA_000014425.1
564 Lactobacillus hominis DSM 23910 = CRBIP 24.179 GCA_000296835.1
459 Lactobacillus iners DSM 13335 GCA_000160875.1
rE
840 Staphylococcus epidermidis ATCC 12228 GCA_000007645.1
799 Staphylococcus capitis subsp. capitis GCA_001028645.1
762 Staphylococcus aureus subsp. aureus NCTC 8325 GCA_000013425.1
rF
784 Lactobacillus gasseri ATCC 33323 = JCM 1131 GCA_000014425.1
728 Lactobacillus hominis DSM 23910 = CRBIP 24.179 GCA_000296835.1
538 Lactobacillus iners DSM 13335 GCA_000160875.1
rG
537 Clostridium beijerinckii GCA_000833105.2
510 Clostridium saccharoperbutylacetonicum N1-4_28HMT_29 GCA_000340885.1
495 Clostridium puniceum GCA_002006345.1
rH

664 Escherichia coli O104_3AH4 str. 2011C-3493 GCA_000299455.1
664 Escherichia coli O157_3AH7 str. Sakai GCA_000008865.1
664 Escherichia coli O83_3AH1 str. NRG 857C GCA_000183345.1
664 Escherichia coli UMN026 GCA_000026325.2
664 Escherichia coli str. K-12 substr. MG1655 GCA_000005845.2
664 Shigella flexneri 2a str. 301 GCA_000006925.2
649 Escherichia coli IAI39 GCA_000026345.1
634 Kosakonia sacchari SP1 GCA_000300455.4
634 Shigella dysenteriae Sd197 GCA_000012005.1
rI
618 Bacillus anthracis str. Ames GCA_000007845.1
618 Bacillus anthracis str. Sterne GCA_000008165.1
618 _5BBacillus thuringiensis_5D serovar konkukian str. 97-27 GCA_000008505.1
608 Bacillus cereus ATCC 14579 GCA_000007825.1
594 Bacillus pseudomycoides DSM 12442 GCA_000161455.1
rJ
617 Streptococcus mutans UA159 GCA_000007465.2
437 Streptococcus gordonii str. Challis substr. CH1 GCA_000017005.1
429 Streptococcus ratti FA-1 = DSM 20564 GCA_000286075.1
rK
576 Rhodobacter sphaeroides 2.4.1 GCA_000012905.2
549 Rhodobacter sphaeroides ATCC 17025 GCA_000016405.1
459 Defluviimonas alba GCA_001620265.1
rL
793 Escherichia coli str. K-12 substr. MG1655 GCA_000005845.2
793 Shigella flexneri 2a str. 301 GCA_000006925.2
792 Escherichia coli O83_3AH1 str. NRG 857C GCA_000183345.1
792 Escherichia coli UMN026 GCA_000026325.2
778 Escherichia coli O157_3AH7 str. Sakai GCA_000008865.1
rM
740 Staphylococcus epidermidis ATCC 12228 GCA_000007645.1
709 Staphylococcus hominis subsp. hominis C80 GCA_000183685.1
703 Staphylococcus haemolyticus JCSC1435 GCA_000009865.1
rN
627 Staphylococcus epidermidis ATCC 12228 GCA_000007645.1
593 Staphylococcus capitis subsp. capitis GCA_001028645.1
561 Staphylococcus aureus subsp. aureus NCTC 8325 GCA_000013425.1
rO
747 Enterococcus faecalis V583 GCA_000007785.1
704 Streptomyces cinnamoneus GCA_001885705.1
620 Enterococcus faecium DO GCA_000174395.2
rP
710 Lactobacillus gasseri ATCC 33323 = JCM 1131 GCA_000014425.1
687 Lactobacillus hominis DSM 23910 = CRBIP 24.179 GCA_000296835.1
501 Lactobacillus iners DSM 13335 GCA_000160875.1
rQ
613 Staphylococcus epidermidis ATCC 12228 GCA_000007645.1
551 Staphylococcus warneri SG1 GCA_000332735.1
537 Staphylococcus capitis subsp. capitis GCA_001028645.1
rR
518 Rhodobacter sphaeroides 2.4.1 GCA_000012905.2
464 Rhodobacter sphaeroides ATCC 17025 GCA_000016405.1
376 Gemmobacter megaterium GCA_900156815.1
rS
452 Deinococcus radiodurans R1 GCA_000008565.1
292 Deinococcus deserti VCD115 GCA_000020685.1
270 Deinococcus gobiensis I-0 GCA_000252445.1
rT
752 Lactobacillus gasseri ATCC 33323 = JCM 1131 GCA_000014425.1
695 Lactobacillus hominis DSM 23910 = CRBIP 24.179 GCA_000296835.1
540 Lactobacillus iners DSM 13335 GCA_000160875.1
rU
762 Rhodobacter sphaeroides 2.4.1 GCA_000012905.2
650 Rhodobacter sphaeroides ATCC 17025 GCA_000016405.1
584 Gemmobacter megaterium GCA_900156815.1
rV
760 Escherichia coli O104_3AH4 str. 2011C-3493 GCA_000299455.1
760 Escherichia coli O157_3AH7 str. Sakai GCA_000008865.1
760 Escherichia coli O83_3AH1 str. NRG 857C GCA_000183345.1
760 Escherichia coli UMN026 GCA_000026325.2
760 Escherichia coli str. K-12 substr. MG1655 GCA_000005845.2
760 Shigella flexneri 2a str. 301 GCA_000006925.2
719 Escherichia coli IAI39 GCA_000026345.1
707 Shigella dysenteriae Sd197 GCA_000012005.1
rW
790 Staphylococcus epidermidis ATCC 12228 GCA_000007645.1

728 Staphylococcus capitis subsp. capitis GCA_001028645.1
693 Staphylococcus warneri SG1 GCA_000332735.1
rX
717 Deinococcus radiodurans R1 GCA_000008565.1
436 Deinococcus deserti VCD115 GCA_000020685.1
410 Deinococcus soli Cha et al. 2016 GCA_001007995.1
rY
650 Streptococcus mutans UA159 GCA_000007465.2
437 Streptococcus ratti FA-1 = DSM 20564 GCA_000286075.1
384 Streptococcus salivarius GCA_000785515.1
384 Streptococcus thermophilus JIM 8232 GCA_000253395.1
rZ
678 Rhodobacter sphaeroides 2.4.1 GCA_000012905.2
643 Rhodobacter sphaeroides ATCC 17025 GCA_000016405.1
543 Pseudorhodobacter psychrotolerans GCA_001294535.1
s0
750 Streptococcus mutans UA159 GCA_000007465.2
579 Streptococcus ratti FA-1 = DSM 20564 GCA_000286075.1
534 Streptococcus gordonii str. Challis substr. CH1 GCA_000017005.1
s1
386 Enterococcus faecalis V583 GCA_000007785.1
354 Enterococcus haemoperoxidus ATCC BAA-382 GCA_000407165.1
354 Enterococcus massiliensis GCA_001050095.1
354 Enterococcus rivorum GCA_001742285.1
347 Enterococcus canis NBRC 100695 GCA_001544375.1
347 Enterococcus dispar ATCC 51266 GCA_000406945.1
s2
528 Rhodobacter sphaeroides 2.4.1 GCA_000012905.2
491 Rhodobacter sphaeroides ATCC 17025 GCA_000016405.1
442 Gemmobacter megaterium GCA_900156815.1
s3
805 Enterococcus faecalis V583 GCA_000007785.1
758 Streptomyces cinnamoneus GCA_001885705.1
712 Enterococcus rivorum GCA_001742285.1
s4
824 Lactobacillus gasseri ATCC 33323 = JCM 1131 GCA_000014425.1
735 Lactobacillus hominis DSM 23910 = CRBIP 24.179 GCA_000296835.1
567 Lactobacillus iners DSM 13335 GCA_000160875.1
s5
794 Streptococcus mutans UA159 GCA_000007465.2
578 Streptococcus ratti FA-1 = DSM 20564 GCA_000286075.1
507 Streptococcus sobrinus DSM 20742 = ATCC 33478 GCA_000686605.1
s6
720 Enterococcus faecalis V583 GCA_000007785.1
684 Enterococcus canis NBRC 100695 GCA_001544375.1
677 Enterococcus rivorum GCA_001742285.1
s7
414 Escherichia coli O157_3AH7 str. Sakai GCA_000008865.1
414 Escherichia coli str. K-12 substr. MG1655 GCA_000005845.2
414 Shigella flexneri 2a str. 301 GCA_000006925.2
410 Escherichia coli O104_3AH4 str. 2011C-3493 GCA_000299455.1
399 Escherichia coli O83_3AH1 str. NRG 857C GCA_000183345.1
399 Escherichia coli UMN026 GCA_000026325.2
s8
882 Lactobacillus gasseri ATCC 33323 = JCM 1131 GCA_000014425.1
817 Lactobacillus hominis DSM 23910 = CRBIP 24.179 GCA_000296835.1
602 Lactobacillus iners DSM 13335 GCA_000160875.1
s9
473 Staphylococcus epidermidis ATCC 12228 GCA_000007645.1
447 Staphylococcus capitis subsp. capitis GCA_001028645.1
428 Staphylococcus haemolyticus JCSC1435 GCA_000009865.1
sa
374 Staphylococcus epidermidis ATCC 12228 GCA_000007645.1
355 Staphylococcus capitis subsp. capitis GCA_001028645.1
330 Staphylococcus pseudintermedius HKU10-03 GCA_000185885.1
sb
822 Enterococcus faecalis V583 GCA_000007785.1
776 Streptomyces cinnamoneus GCA_001885705.1
707 Enterococcus rivorum GCA_001742285.1
sc
328 Rhodobacter sphaeroides 2.4.1 GCA_000012905.2
310 Rhodobacter sphaeroides ATCC 17025 GCA_000016405.1
276 Defluviimonas alba GCA_001620265.1
sd
429 Deinococcus radiodurans R1 GCA_000008565.1
289 Deinococcus deserti VCD115 GCA_000020685.1

282 Deinococcus soli Cha et al. 2016 GCA_001007995.1
se
439 Rhodobacter sphaeroides 2.4.1 GCA_000012905.2
403 Rhodobacter sphaeroides ATCC 17025 GCA_000016405.1
345 Gemmobacter aquatilis GCA_900110025.1
sf
580 Enterococcus faecalis V583 GCA_000007785.1
533 Streptomyces cinnamoneus GCA_001885705.1
459 Enterococcus hirae ATCC 9790 GCA_000271405.2
459 Enterococcus phoeniculicola ATCC BAA-412 GCA_000407505.1
sg
462 Streptomyces cinnamoneus GCA_001885705.1
460 Bacillus anthracis str. Ames GCA_000007845.1
460 Bacillus anthracis str. Sterne GCA_000008165.1
460 Bacillus cereus ATCC 14579 GCA_000007825.1
460 Bacillus thuringiensis YBT-1518 GCA_000497525.2
460 _5BBacillus thuringiensis_5D serovar konkukian str. 97-27 GCA_000008505.1
448 Enterococcus faecalis V583 GCA_000007785.1
sh
542 Rhodobacter sphaeroides 2.4.1 GCA_000012905.2
438 Rhodobacter sphaeroides ATCC 17025 GCA_000016405.1
348 Pseudorhodobacter wandonensis GCA_001202035.1
si
699 Staphylococcus epidermidis ATCC 12228 GCA_000007645.1
642 Staphylococcus capitis subsp. capitis GCA_001028645.1
628 Staphylococcus hominis subsp. hominis C80 GCA_000183685.1
sj
379 Clostridium beijerinckii GCA_000833105.2
354 Clostridium saccharoperbutylacetonicum N1-4_28HMT_29 GCA_000340885.1
353 Clostridium saccharobutylicum DSM 13864 GCA_000473995.1
sk
763 Lactobacillus gasseri ATCC 33323 = JCM 1131 GCA_000014425.1
744 Lactobacillus hominis DSM 23910 = CRBIP 24.179 GCA_000296835.1
531 Lactobacillus iners DSM 13335 GCA_000160875.1
sl
541 Bifidobacterium adolescentis ATCC 15703 GCA_000010425.1
446 Bifidobacterium dentium JCM 1195 = DSM 20436 GCA_001042595.1
432 Bifidobacterium longum NCC2705 GCA_000007525.1
sm
844 Deinococcus radiodurans R1 GCA_000008565.1
556 Deinococcus deserti VCD115 GCA_000020685.1
552 Deinococcus gobiensis I-0 GCA_000252445.1
sn
716 Lactobacillus gasseri ATCC 33323 = JCM 1131 GCA_000014425.1
660 Lactobacillus hominis DSM 23910 = CRBIP 24.179 GCA_000296835.1
456 Lactobacillus iners DSM 13335 GCA_000160875.1
so
678 Lactobacillus gasseri ATCC 33323 = JCM 1131 GCA_000014425.1
626 Lactobacillus hominis DSM 23910 = CRBIP 24.179 GCA_000296835.1
416 Lactobacillus psittaci DSM 15354 GCA_000425905.1
sp
788 Enterococcus faecalis V583 GCA_000007785.1
741 Streptomyces cinnamoneus GCA_001885705.1
706 Enterococcus rivorum GCA_001742285.1
sq
706 Lactobacillus gasseri ATCC 33323 = JCM 1131 GCA_000014425.1
691 Lactobacillus hominis DSM 23910 = CRBIP 24.179 GCA_000296835.1
547 Lactobacillus hamsteri DSM 5661 = JCM 6256 GCA_000615445.1
sr
742 Rhodobacter sphaeroides 2.4.1 GCA_000012905.2
650 Rhodobacter sphaeroides ATCC 17025 GCA_000016405.1
590 Pseudorhodobacter ferrugineus DSM 5888 GCA_000420745.1
ss
657 Enterococcus faecalis V583 GCA_000007785.1
610 Streptomyces cinnamoneus GCA_001885705.1
535 Enterococcus faecium DO GCA_000174395.2
st
796 Deinococcus radiodurans R1 GCA_000008565.1
568 Deinococcus deserti VCD115 GCA_000020685.1
541 Deinococcus murrayi DSM 11303 GCA_000482805.1
su
880 Bacillus anthracis str. Ames GCA_000007845.1
880 Bacillus anthracis str. Sterne GCA_000008165.1
880 _5BBacillus thuringiensis_5D serovar konkukian str. 97-27 GCA_000008505.1
864 Bacillus cereus ATCC 14579 GCA_000007825.1
834 Bacillus pseudomycoides DSM 12442 GCA_000161455.1

sv
429 Rhodobacter sphaeroides 2.4.1 GCA_000012905.2
423 Rhodobacter sphaeroides ATCC 17025 GCA_000016405.1
396 Pseudorhodobacter ferrugineus DSM 5888 GCA_000420745.1
sw
796 Lactobacillus gasseri ATCC 33323 = JCM 1131 GCA_000014425.1
770 Lactobacillus hominis DSM 23910 = CRBIP 24.179 GCA_000296835.1
560 Lactobacillus iners DSM 13335 GCA_000160875.1
sx
713 Streptococcus mutans UA159 GCA_000007465.2
496 Streptococcus gordonii str. Challis substr. CH1 GCA_000017005.1
471 Streptococcus ratti FA-1 = DSM 20564 GCA_000286075.1
sy
713 Staphylococcus epidermidis ATCC 12228 GCA_000007645.1
653 Staphylococcus capitis subsp. capitis GCA_001028645.1
610 Staphylococcus haemolyticus JCSC1435 GCA_000009865.1
sz
589 Staphylococcus epidermidis ATCC 12228 GCA_000007645.1
546 Staphylococcus lugdunensis HKU09-01 GCA_000025085.1
542 Staphylococcus capitis subsp. capitis GCA_001028645.1
sA
680 Clostridium beijerinckii GCA_000833105.2
644 Clostridium saccharoperbutylacetonicum N1-4_28HMT_29 GCA_000340885.1
621 Clostridium puniceum GCA_002006345.1
sB
600 Lactobacillus gasseri ATCC 33323 = JCM 1131 GCA_000014425.1
561 Lactobacillus hominis DSM 23910 = CRBIP 24.179 GCA_000296835.1
430 Lactobacillus iners DSM 13335 GCA_000160875.1
sC
425 Staphylococcus epidermidis ATCC 12228 GCA_000007645.1
390 Staphylococcus capitis subsp. capitis GCA_001028645.1
389 Staphylococcus haemolyticus JCSC1435 GCA_000009865.1
sD
412 Rhodobacter sphaeroides 2.4.1 GCA_000012905.2
359 Rhodobacter sphaeroides ATCC 17025 GCA_000016405.1
281 Gemmobacter aquatilis GCA_900110025.1
sE
762 Streptococcus mutans UA159 GCA_000007465.2
609 Streptococcus ratti FA-1 = DSM 20564 GCA_000286075.1
513 Streptococcus halotolerans GCA_001598035.1
sF
601 Staphylococcus epidermidis ATCC 12228 GCA_000007645.1
573 Staphylococcus capitis subsp. capitis GCA_001028645.1
502 Staphylococcus pettenkoferi GCA_002208805.1
sG
721 Staphylococcus epidermidis ATCC 12228 GCA_000007645.1
693 Staphylococcus capitis subsp. capitis GCA_001028645.1
629 Staphylococcus aureus subsp. aureus NCTC 8325 GCA_000013425.1
sH
683 Bacillus anthracis str. Ames GCA_000007845.1
683 Bacillus anthracis str. Sterne GCA_000008165.1
683 _5BBacillus thuringiensis_5D serovar konkukian str. 97-27 GCA_000008505.1
663 Bacillus cereus ATCC 14579 GCA_000007825.1
663 Bacillus thuringiensis YBT-1518 GCA_000497525.2
659 Bacillus pseudomycoides DSM 12442 GCA_000161455.1
sI
693 Deinococcus radiodurans R1 GCA_000008565.1
416 Deinococcus proteolyticus MRP GCA_000190555.1
402 Deinococcus gobiensis I-0 GCA_000252445.1
sJ
505 Escherichia coli O157_3AH7 str. Sakai GCA_000008865.1
505 Escherichia coli str. K-12 substr. MG1655 GCA_000005845.2
497 Escherichia coli UMN026 GCA_000026325.2
493 Escherichia coli IAI39 GCA_000026345.1
493 Escherichia coli O104_3AH4 str. 2011C-3493 GCA_000299455.1
493 Shigella flexneri 2a str. 301 GCA_000006925.2
sK
531 Clostridium beijerinckii GCA_000833105.2
493 Clostridium saccharoperbutylacetonicum N1-4_28HMT_29 GCA_000340885.1
483 Clostridium puniceum GCA_002006345.1
sL
851 Enterococcus faecalis V583 GCA_000007785.1
809 Streptomyces cinnamoneus GCA_001885705.1
749 Enterococcus asini ATCC 700915 GCA_000407365.1
sM
762 Clostridium beijerinckii GCA_000833105.2

733 Clostridium saccharoperbutylacetonicum N1-4_28HMT_29 GCA_000340885.1
706 Clostridium puniceum GCA_002006345.1
sN
644 Rhodobacter sphaeroides 2.4.1 GCA_000012905.2
618 Rhodobacter sphaeroides ATCC 17025 GCA_000016405.1
497 Defluviimonas alba GCA_001620265.1
sO
935 Lactobacillus gasseri ATCC 33323 = JCM 1131 GCA_000014425.1
825 Lactobacillus hominis DSM 23910 = CRBIP 24.179 GCA_000296835.1
596 Lactobacillus iners DSM 13335 GCA_000160875.1
sP
754 Bifidobacterium adolescentis ATCC 15703 GCA_000010425.1
680 Bifidobacterium dentium JCM 1195 = DSM 20436 GCA_001042595.1
597 Bifidobacterium breve DSM 20213 = JCM 1192 GCA_001025175.1
sQ
798 Clostridium beijerinckii GCA_000833105.2
725 Clostridium saccharoperbutylacetonicum N1-4_28HMT_29 GCA_000340885.1
695 Clostridium puniceum GCA_002006345.1
sR
658 Rhodobacter sphaeroides 2.4.1 GCA_000012905.2
600 Rhodobacter sphaeroides ATCC 17025 GCA_000016405.1
492 Gemmobacter aquatilis GCA_900110025.1
sS
542 Rhodobacter sphaeroides 2.4.1 GCA_000012905.2
515 Rhodobacter sphaeroides ATCC 17025 GCA_000016405.1
450 Gemmobacter aquatilis GCA_900110025.1
sT
415 Clostridium beijerinckii GCA_000833105.2
395 Clostridium saccharoperbutylacetonicum N1-4_28HMT_29 GCA_000340885.1
392 Clostridium saccharobutylicum DSM 13864 GCA_000473995.1
sU
662 Clostridium saccharoperbutylacetonicum N1-4_28HMT_29 GCA_000340885.1
658 Clostridium beijerinckii GCA_000833105.2
641 Clostridium puniceum GCA_002006345.1
sV
498 Lactobacillus gasseri ATCC 33323 = JCM 1131 GCA_000014425.1
424 Lactobacillus hominis DSM 23910 = CRBIP 24.179 GCA_000296835.1
318 Lactobacillus acetotolerans GCA_001042405.1
sW
510 Rhodobacter sphaeroides 2.4.1 GCA_000012905.2
456 Rhodobacter sphaeroides ATCC 17025 GCA_000016405.1
381 Gemmobacter aquatilis GCA_900110025.1
sX
697 Clostridium beijerinckii GCA_000833105.2
643 Clostridium saccharoperbutylacetonicum N1-4_28HMT_29 GCA_000340885.1
601 Clostridium saccharobutylicum DSM 13864 GCA_000473995.1
sY
699 Deinococcus radiodurans R1 GCA_000008565.1
448 Deinococcus deserti VCD115 GCA_000020685.1
419 Deinococcus proteolyticus MRP GCA_000190555.1
sZ
822 Streptococcus mutans UA159 GCA_000007465.2
601 Streptococcus ratti FA-1 = DSM 20564 GCA_000286075.1
599 Streptococcus ferus DSM 20646 GCA_000372425.1
t0
820 Clostridium beijerinckii GCA_000833105.2
797 Clostridium saccharoperbutylacetonicum N1-4_28HMT_29 GCA_000340885.1
793 Clostridium puniceum GCA_002006345.1
t1
615 Clostridium beijerinckii GCA_000833105.2
608 Clostridium saccharoperbutylacetonicum N1-4_28HMT_29 GCA_000340885.1
582 Clostridium saccharobutylicum DSM 13864 GCA_000473995.1
t2
526 Rhodobacter sphaeroides 2.4.1 GCA_000012905.2
505 Rhodobacter sphaeroides ATCC 17025 GCA_000016405.1
381 Pseudorhodobacter psychrotolerans GCA_001294535.1
t3
533 Lactobacillus gasseri ATCC 33323 = JCM 1131 GCA_000014425.1
460 Lactobacillus hominis DSM 23910 = CRBIP 24.179 GCA_000296835.1
423 Lactobacillus psittaci DSM 15354 GCA_000425905.1
t4
658 Lactobacillus gasseri ATCC 33323 = JCM 1131 GCA_000014425.1
561 Lactobacillus hominis DSM 23910 = CRBIP 24.179 GCA_000296835.1
434 Lactobacillus psittaci DSM 15354 GCA_000425905.1
t5
654 Clostridium beijerinckii GCA_000833105.2

637 Clostridium saccharoperbutylacetonicum N1-4_28HMT_29 GCA_000340885.1
600 Clostridium saccharobutylicum DSM 13864 GCA_000473995.1
t6
261 Rhodobacter sphaeroides 2.4.1 GCA_000012905.2
208 Rhodobacter sphaeroides ATCC 17025 GCA_000016405.1
200 Gemmobacter aquatilis GCA_900110025.1
t7
699 Rhodobacter sphaeroides 2.4.1 GCA_000012905.2
597 Rhodobacter sphaeroides ATCC 17025 GCA_000016405.1
522 Defluviimonas alba GCA_001620265.1
t8
735 Enterococcus faecalis V583 GCA_000007785.1
688 Streptomyces cinnamoneus GCA_001885705.1
603 Enterococcus rivorum GCA_001742285.1
t9
584 Streptococcus mutans UA159 GCA_000007465.2
390 Streptococcus ratti FA-1 = DSM 20564 GCA_000286075.1
348 Streptococcus ferus DSM 20646 GCA_000372425.1
348 Streptococcus henryi DSM 19005 GCA_000376985.1
ta
887 Bacillus anthracis str. Ames GCA_000007845.1
887 Bacillus anthracis str. Sterne GCA_000008165.1
887 _5BBacillus thuringiensis_5D serovar konkukian str. 97-27 GCA_000008505.1
871 Bacillus cereus ATCC 14579 GCA_000007825.1
867 Bacillus thuringiensis YBT-1518 GCA_000497525.2
tb
761 Lactobacillus gasseri ATCC 33323 = JCM 1131 GCA_000014425.1
695 Lactobacillus hominis DSM 23910 = CRBIP 24.179 GCA_000296835.1
487 Lactobacillus hamsteri DSM 5661 = JCM 6256 GCA_000615445.1
tc
733 Clostridium beijerinckii GCA_000833105.2
709 Clostridium saccharoperbutylacetonicum N1-4_28HMT_29 GCA_000340885.1
661 Clostridium saccharobutylicum DSM 13864 GCA_000473995.1
td
357 Rhodobacter sphaeroides 2.4.1 GCA_000012905.2
296 Rhodobacter sphaeroides ATCC 17025 GCA_000016405.1
217 Rhodobacter capsulatus SB 1003 GCA_000021865.1
te
757 Escherichia coli str. K-12 substr. MG1655 GCA_000005845.2
742 Escherichia coli O157_3AH7 str. Sakai GCA_000008865.1
737 Escherichia coli UMN026 GCA_000026325.2
tf
891 Rhodobacter sphaeroides 2.4.1 GCA_000012905.2
786 Rhodobacter sphaeroides ATCC 17025 GCA_000016405.1
625 Gemmobacter megaterium GCA_900156815.1
tg
672 Deinococcus radiodurans R1 GCA_000008565.1
349 Deinococcus deserti VCD115 GCA_000020685.1
333 Deinococcus gobiensis I-0 GCA_000252445.1
th
718 Clostridium beijerinckii GCA_000833105.2
689 Clostridium saccharoperbutylacetonicum N1-4_28HMT_29 GCA_000340885.1
659 Clostridium puniceum GCA_002006345.1
ti
700 Bifidobacterium adolescentis ATCC 15703 GCA_000010425.1
655 Bifidobacterium dentium JCM 1195 = DSM 20436 GCA_001042595.1
611 Bifidobacterium animalis subsp. lactis DSM 10140 GCA_000022965.1
tj
804 Clostridium beijerinckii GCA_000833105.2
770 Clostridium saccharoperbutylacetonicum N1-4_28HMT_29 GCA_000340885.1
729 Clostridium puniceum GCA_002006345.1
tk
579 Enterococcus faecalis V583 GCA_000007785.1
532 Streptomyces cinnamoneus GCA_001885705.1
506 Enterococcus faecium DO GCA_000174395.2
tl
426 Rhodobacter sphaeroides 2.4.1 GCA_000012905.2
354 Gemmobacter megaterium GCA_900156815.1
354 Rhodobacter sphaeroides ATCC 17025 GCA_000016405.1
351 Pseudorhodobacter psychrotolerans GCA_001294535.1
tm
589 Deinococcus radiodurans R1 GCA_000008565.1
380 Deinococcus deserti VCD115 GCA_000020685.1
372 Deinococcus marmoris DSM 12784 GCA_000701405.1
tn
658 Staphylococcus epidermidis ATCC 12228 GCA_000007645.1

637 Staphylococcus haemolyticus JCSC1435 GCA_000009865.1
636 Staphylococcus hominis subsp. hominis C80 GCA_000183685.1
 to
519 Clostridium beijerinckii GCA_000833105.2
493 Clostridium saccharoperbutylacetonicum N1-4_28HMT_29 GCA_000340885.1
454 Clostridium saccharobutylicum DSM 13864 GCA_000473995.1
 tp
558 Deinococcus radiodurans R1 GCA_000008565.1
323 Deinococcus deserti VCD115 GCA_000020685.1
313 Deinococcus gobiensis I-0 GCA_000252445.1
 tq
823 Rhodobacter sphaeroides 2.4.1 GCA_000012905.2
729 Rhodobacter sphaeroides ATCC 17025 GCA_000016405.1
583 Rhodobacter vinaykumarii GCA_900156695.1
 tr
537 Staphylococcus epidermidis ATCC 12228 GCA_000007645.1
506 Staphylococcus capitis subsp. capitis GCA_001028645.1
455 Megasphaera cerevisiae DSM 20462 GCA_001045675.1
455 Staphylococcus warneri SG1 GCA_000332735.1
 ts
752 Bacillus anthracis str. Ames GCA_000007845.1
752 Bacillus anthracis str. Sterne GCA_000008165.1
752 _5BBacillus thuringiensis_5D serovar konkukian str. 97-27 GCA_000008505.1
737 Bacillus cereus ATCC 14579 GCA_000007825.1
727 Bacillus pseudomycoides DSM 12442 GCA_000161455.1
 tt
617 Lactobacillus gasseri ATCC 33323 = JCM 1131 GCA_000014425.1
570 Lactobacillus hominis DSM 23910 = CRBIP 24.179 GCA_000296835.1
415 Lactobacillus iners DSM 13335 GCA_000160875.1
 tu
647 Clostridium beijerinckii GCA_000833105.2
616 Clostridium saccharoperbutylacetonicum N1-4_28HMT_29 GCA_000340885.1
590 Clostridium puniceum GCA_002006345.1
 tv
393 Enterococcus faecalis V583 GCA_000007785.1
370 Enterococcus canis NBRC 100695 GCA_001544375.1
355 Enterococcus thailandicus GCA_001652875.1
 tw
840 Lactobacillus gasseri ATCC 33323 = JCM 1131 GCA_000014425.1
790 Lactobacillus hominis DSM 23910 = CRBIP 24.179 GCA_000296835.1
620 Lactobacillus iners DSM 13335 GCA_000160875.1
 tx
613 Staphylococcus epidermidis ATCC 12228 GCA_000007645.1
589 Staphylococcus capitis subsp. capitis GCA_001028645.1
537 Staphylococcus hominis subsp. hominis C80 GCA_000183685.1
 ty
735 Staphylococcus epidermidis ATCC 12228 GCA_000007645.1
707 Staphylococcus capitis subsp. capitis GCA_001028645.1
666 Staphylococcus aureus subsp. aureus NCTC 8325 GCA_000013425.1
 tz
777 Staphylococcus epidermidis ATCC 12228 GCA_000007645.1
715 Staphylococcus capitis subsp. capitis GCA_001028645.1
679 Staphylococcus hominis subsp. hominis C80 GCA_000183685.1
 tA
563 Escherichia coli str. K-12 substr. MG1655 GCA_000005845.2
545 Escherichia coli O157_3AH7 str. Sakai GCA_000008865.1
545 Shigella flexneri 2a str. 301 GCA_000006925.2
544 Escherichia coli O83_3AH1 str. NRG 857C GCA_000183345.1
544 Escherichia coli UMN026 GCA_000026325.2
 tB
384 Escherichia coli O157_3AH7 str. Sakai GCA_000008865.1
384 Escherichia coli UMN026 GCA_000026325.2
384 Escherichia coli str. K-12 substr. MG1655 GCA_000005845.2
377 Escherichia coli IAI39 GCA_000026345.1
377 Escherichia coli O104_3AH4 str. 2011C-3493 GCA_000299455.1
377 Shigella flexneri 2a str. 301 GCA_000006925.2
376 Enterobacter hormaechei subsp. steigerwaltii GCA_001729725.1
 tC
566 Clostridium beijerinckii GCA_000833105.2
560 Clostridium saccharoperbutylacetonicum N1-4_28HMT_29 GCA_000340885.1
548 Clostridium neonatale GCA_001458595.1
 tD
775 Bacillus anthracis str. Ames GCA_000007845.1
775 Bacillus anthracis str. Sterne GCA_000008165.1
775 Bacillus cereus ATCC 14579 GCA_000007825.1
775 Bacillus thuringiensis YBT-1518 GCA_000497525.2

775 _5BBacillus thuringiensis_5D serovar konkukian str. 97-27 GCA_000008505.1
734 Bacillus pseudomycoides DSM 12442 GCA_000161455.1
731 Bacillus mycoides GCA_000832605.1
tE
694 Staphylococcus epidermidis ATCC 12228 GCA_000007645.1
681 Staphylococcus capitis subsp. capitis GCA_001028645.1
633 Megasphaera cerevisiae DSM 20462 GCA_001045675.1
633 Staphylococcus warneri SG1 GCA_000332735.1
tF
896 Staphylococcus epidermidis ATCC 12228 GCA_000007645.1
856 Staphylococcus capitis subsp. capitis GCA_001028645.1
799 Staphylococcus aureus subsp. aureus NCTC 8325 GCA_000013425.1
tG
798 Deinococcus radiodurans R1 GCA_000008565.1
493 Deinococcus deserti VCD115 GCA_000020685.1
480 Deinococcus gobiensis I-0 GCA_000252445.1
tH
793 Streptococcus mutans UA159 GCA_000007465.2
612 Streptococcus ratti FA-1 = DSM 20564 GCA_000286075.1
558 Streptococcus gordonii str. Challis substr. CH1 GCA_000017005.1
tI
700 Clostridium beijerinckii GCA_000833105.2
668 Clostridium saccharoperbutylacetonicum N1-4_28HMT_29 GCA_000340885.1
630 Clostridium puniceum GCA_002006345.1
tJ
934 Clostridium beijerinckii GCA_000833105.2
921 Clostridium saccharoperbutylacetonicum N1-4_28HMT_29 GCA_000340885.1
885 Clostridium saccharobutylicum DSM 13864 GCA_000473995.1
tK
806 Streptococcus mutans UA159 GCA_000007465.2
618 Streptococcus ratti FA-1 = DSM 20564 GCA_000286075.1
584 Streptococcus gordonii str. Challis substr. CH1 GCA_000017005.1
tL
694 Rhodobacter sphaeroides 2.4.1 GCA_000012905.2
624 Rhodobacter sphaeroides ATCC 17025 GCA_000016405.1
534 Gemmobacter aquatilis GCA_900110025.1
tM
704 Enterococcus faecalis V583 GCA_000007785.1
657 Streptomyces cinnamoneus GCA_001885705.1
607 Enterococcus dispar ATCC 51266 GCA_000406945.1
tN
616 Lactobacillus gasseri ATCC 33323 = JCM 1131 GCA_000014425.1
557 Lactobacillus hominis DSM 23910 = CRBIP 24.179 GCA_000296835.1
398 Lactobacillus acetotolerans GCA_001042405.1
398 Lactobacillus hamsteri DSM 5661 = JCM 6256 GCA_000615445.1
tO
593 Bacillus mycoides GCA_000832605.1
592 Bacillus thuringiensis YBT-1518 GCA_000497525.2
591 Bacillus anthracis str. Ames GCA_000007845.1
591 Bacillus anthracis str. Sterne GCA_000008165.1
591 _5BBacillus thuringiensis_5D serovar konkukian str. 97-27 GCA_000008505.1
tP
673 Streptococcus mutans UA159 GCA_000007465.2
497 Streptococcus ratti FA-1 = DSM 20564 GCA_000286075.1
439 Streptococcus halotolerans GCA_001598035.1
tQ
665 Streptococcus mutans UA159 GCA_000007465.2
468 Streptococcus ratti FA-1 = DSM 20564 GCA_000286075.1
422 Streptococcus sobrinus DSM 20742 = ATCC 33478 GCA_000686605.1
tR
454 Enterococcus faecalis V583 GCA_000007785.1
412 Enterococcus haemoperoxidus ATCC BAA-382 GCA_000407165.1
407 Enterococcus aquimarinus GCA_001885765.1
407 Enterococcus rivorum GCA_001742285.1
407 Streptomyces cinnamoneus GCA_001885705.1
tS
641 Clostridium beijerinckii GCA_000833105.2
593 Clostridium saccharoperbutylacetonicum N1-4_28HMT_29 GCA_000340885.1
572 Clostridium saccharobutylicum DSM 13864 GCA_000473995.1
tT
918 Escherichia coli str. K-12 substr. MG1655 GCA_000005845.2
917 Escherichia coli UMN026 GCA_000026325.2
910 Escherichia coli O157_3AH7 str. Sakai GCA_000008865.1
tU
277 Bifidobacterium adolescentis ATCC 15703 GCA_000010425.1
262 Bifidobacterium asteroides PRL2011 GCA_000304215.1

244 Bifidobacterium coryneforme GCA_000737865.1
tV
606 Staphylococcus epidermidis ATCC 12228 GCA_000007645.1
572 Staphylococcus haemolyticus JCSC1435 GCA_000009865.1
567 Staphylococcus hominis subsp. hominis C80 GCA_000183685.1
tW
707 Rhodobacter sphaeroides 2.4.1 GCA_000012905.2
657 Rhodobacter sphaeroides ATCC 17025 GCA_000016405.1
527 Gemmobacter megaterium GCA_900156815.1
tX
560 Escherichia coli O157_3AH7 str. Sakai GCA_000008865.1
560 Escherichia coli str. K-12 substr. MG1655 GCA_000005845.2
552 Escherichia coli UMN026 GCA_000026325.2
532 Escherichia coli O83_3AH1 str. NRG 857C GCA_000183345.1
tY
688 Staphylococcus epidermidis ATCC 12228 GCA_000007645.1
623 Staphylococcus capitis subsp. capitis GCA_001028645.1
590 Staphylococcus haemolyticus JCSC1435 GCA_000009865.1
590 Staphylococcus hominis subsp. hominis C80 GCA_000183685.1
tZ
821 Rhodobacter sphaeroides 2.4.1 GCA_000012905.2
718 Rhodobacter sphaeroides ATCC 17025 GCA_000016405.1
588 Defluviimonas alba GCA_001620265.1
u0
712 Clostridium beijerinckii GCA_000833105.2
695 Clostridium saccharoperbutylacetonicum N1-4_28HMT_29 GCA_000340885.1
674 Clostridium saccharobutylicum DSM 13864 GCA_000473995.1
u1
627 Rhodobacter sphaeroides 2.4.1 GCA_000012905.2
554 Rhodobacter sphaeroides ATCC 17025 GCA_000016405.1
439 Defluviimonas alba GCA_001620265.1
439 Pseudorhodobacter ferrugineus DSM 5888 GCA_000420745.1
u2
501 Bacillus cereus ATCC 14579 GCA_000007825.1
501 _5BBacillus thuringiensis_5D serovar konkukian str. 97-27 GCA_000008505.1
500 Bacillus anthracis str. Ames GCA_000007845.1
500 Bacillus anthracis str. Sterne GCA_000008165.1
500 Bacillus thuringiensis YBT-1518 GCA_000497525.2
479 Bacillus pseudomycoides DSM 12442 GCA_000161455.1
u3
827 Streptococcus mutans UA159 GCA_000007465.2
645 Streptococcus ratti FA-1 = DSM 20564 GCA_000286075.1
605 Streptococcus halotolerans GCA_001598035.1
u4
561 Deinococcus radiodurans R1 GCA_000008565.1
380 Deinococcus gobiensis I-0 GCA_000252445.1
368 Deinococcus soli Cha et al. 2016 GCA_001007995.1
u5
935 Streptococcus mutans UA159 GCA_000007465.2
670 Streptococcus ratti FA-1 = DSM 20564 GCA_000286075.1
648 Streptococcus gordonii str. Challis substr. CH1 GCA_000017005.1
u6
640 Enterococcus faecalis V583 GCA_000007785.1
595 Streptomyces cinnamoneus GCA_001885705.1
589 Enterococcus haemoperoxidus ATCC BAA-382 GCA_000407165.1
u7
672 Rhodobacter sphaeroides 2.4.1 GCA_000012905.2
604 Rhodobacter sphaeroides ATCC 17025 GCA_000016405.1
542 Pseudorhodobacter psychrotolerans GCA_001294535.1
u8
704 Bacillus anthracis str. Ames GCA_000007845.1
704 Bacillus anthracis str. Sterne GCA_000008165.1
690 _5BBacillus thuringiensis_5D serovar konkukian str. 97-27 GCA_000008505.1
686 Bacillus thuringiensis YBT-1518 GCA_000497525.2
u9
758 Deinococcus radiodurans R1 GCA_000008565.1
510 Deinococcus puniceus GCA_001644565.1
498 Deinococcus soli Cha et al. 2016 GCA_001007995.1
ua
908 Staphylococcus epidermidis ATCC 12228 GCA_000007645.1
846 Staphylococcus capitis subsp. capitis GCA_001028645.1
785 Staphylococcus aureus subsp. aureus NCTC 8325 GCA_000013425.1
ub
661 Rhodobacter sphaeroides 2.4.1 GCA_000012905.2
593 Rhodobacter sphaeroides ATCC 17025 GCA_000016405.1
526 Defluviimonas alba GCA_001620265.1

uc
823 Deinococcus radiodurans R1 GCA_000008565.1
567 Deinococcus deserti VCD115 GCA_000020685.1
537 Deinococcus murrayi DSM 11303 GCA_000482805.1
ud
809 Rhodobacter sphaeroides 2.4.1 GCA_000012905.2
720 Rhodobacter sphaeroides ATCC 17025 GCA_000016405.1
594 Gemmobacter aquatilis GCA_900110025.1
ue
568 Lactobacillus gasseri ATCC 33323 = JCM 1131 GCA_000014425.1
522 Lactobacillus hominis DSM 23910 = CRBIP 24.179 GCA_000296835.1
502 Lactobacillus iners DSM 13335 GCA_000160875.1
uf
616 Shigella flexneri 2a str. 301 GCA_000006925.2
615 Escherichia coli O104_3AH4 str. 2011C-3493 GCA_000299455.1
615 Escherichia coli O157_3AH7 str. Sakai GCA_000008865.1
615 Escherichia coli str. K-12 substr. MG1655 GCA_000005845.2
614 Escherichia coli O83_3AH1 str. NRG 857C GCA_000183345.1
614 Escherichia coli UMN026 GCA_000026325.2
ug
784 Bacillus cereus ATCC 14579 GCA_000007825.1
784 Bacillus thuringiensis YBT-1518 GCA_000497525.2
784 _5BBacillus thuringiensis_5D serovar konkukian str. 97-27 GCA_000008505.1
783 Bacillus anthracis str. Ames GCA_000007845.1
783 Bacillus anthracis str. Sterne GCA_000008165.1
739 Bacillus pseudomycoides DSM 12442 GCA_000161455.1
uh
901 Clostridium beijerinckii GCA_000833105.2
873 Clostridium saccharoperbutylacetonicum N1-4_28HMT_29 GCA_000340885.1
869 Clostridium puniceum GCA_002006345.1
ui
440 Staphylococcus epidermidis ATCC 12228 GCA_000007645.1
406 Staphylococcus capitis subsp. capitis GCA_001028645.1
390 Staphylococcus aureus subsp. aureus NCTC 8325 GCA_000013425.1
uj
774 Staphylococcus epidermidis ATCC 12228 GCA_000007645.1
709 Staphylococcus capitis subsp. capitis GCA_001028645.1
661 Staphylococcus lugdunensis HKU09-01 GCA_000025085.1
uk
674 Bifidobacterium adolescentis ATCC 15703 GCA_000010425.1
576 Bifidobacterium dentium JCM 1195 = DSM 20436 GCA_001042595.1
574 Bifidobacterium angulatum DSM 20098 = JCM 7096 GCA_001025155.1
ul
568 Streptococcus mutans UA159 GCA_000007465.2
449 Streptococcus ratti FA-1 = DSM 20564 GCA_000286075.1
429 Streptococcus ovis DSM 16829 GCA_000380125.1
um
639 Rhodobacter sphaeroides 2.4.1 GCA_000012905.2
525 Rhodobacter sphaeroides ATCC 17025 GCA_000016405.1
455 Gemmobacter aquatilis GCA_900110025.1
un
920 Lactobacillus gasseri ATCC 33323 = JCM 1131 GCA_000014425.1
831 Lactobacillus hominis DSM 23910 = CRBIP 24.179 GCA_000296835.1
617 Lactobacillus iners DSM 13335 GCA_000160875.1
uo
844 Clostridium beijerinckii GCA_000833105.2
802 Clostridium saccharoperbutylacetonicum N1-4_28HMT_29 GCA_000340885.1
776 Clostridium puniceum GCA_002006345.1
up
455 Streptococcus mutans UA159 GCA_000007465.2
313 Streptococcus gordonii str. Challis substr. CH1 GCA_000017005.1
303 Streptococcus ratti FA-1 = DSM 20564 GCA_000286075.1
uq
594 Deinococcus radiodurans R1 GCA_000008565.1
405 Deinococcus deserti VCD115 GCA_000020685.1
385 Deinococcus hopiensis KR-140 GCA_900176165.1
ur
904 Staphylococcus epidermidis ATCC 12228 GCA_000007645.1
861 Staphylococcus capitis subsp. capitis GCA_001028645.1
811 Staphylococcus haemolyticus JCSC1435 GCA_000009865.1
811 Staphylococcus hominis subsp. hominis C80 GCA_000183685.1
us
696 Rhodobacter sphaeroides 2.4.1 GCA_000012905.2
647 Rhodobacter sphaeroides ATCC 17025 GCA_000016405.1
487 Gemmobacter aquatilis GCA_900110025.1
ut

765 Deinococcus radiodurans R1 GCA_000008565.1
472 Deinococcus deserti VCD115 GCA_000020685.1
459 Deinococcus gobiensis I-0 GCA_000252445.1
uu
562 Streptococcus mutans UA159 GCA_000007465.2
414 Streptococcus ratti FA-1 = DSM 20564 GCA_000286075.1
392 Streptococcus sobrinus DSM 20742 = ATCC 33478 GCA_000686605.1
uv
436 Lactobacillus gasseri ATCC 33323 = JCM 1131 GCA_000014425.1
414 Lactobacillus hominis DSM 23910 = CRBIP 24.179 GCA_000296835.1
308 Lactobacillus iners DSM 13335 GCA_000160875.1
uw
645 Streptococcus mutans UA159 GCA_000007465.2
495 Streptococcus ratti FA-1 = DSM 20564 GCA_000286075.1
437 Streptococcus merionis DSM 19192 GCA_000380085.1
ux
725 Lactobacillus gasseri ATCC 33323 = JCM 1131 GCA_000014425.1
694 Lactobacillus hominis DSM 23910 = CRBIP 24.179 GCA_000296835.1
528 Lactobacillus iners DSM 13335 GCA_000160875.1
uy
503 Rhodobacter sphaeroides 2.4.1 GCA_000012905.2
433 Rhodobacter sphaeroides ATCC 17025 GCA_000016405.1
387 Gemmobacter megaterium GCA_900156815.1
uz
818 Clostridium beijerinckii GCA_000833105.2
778 Clostridium saccharoperbutylacetonicum N1-4_28HMT_29 GCA_000340885.1
763 Clostridium puniceum GCA_002006345.1
uA
639 Staphylococcus epidermidis ATCC 12228 GCA_000007645.1
598 Staphylococcus capitis subsp. capitis GCA_001028645.1
538 Staphylococcus aureus subsp. aureus NCTC 8325 GCA_000013425.1
538 Staphylococcus simulans GCA_001559115.1
uB
447 Bifidobacterium adolescentis ATCC 15703 GCA_000010425.1
381 Bifidobacterium longum NCC2705 GCA_000007525.1
375 Bifidobacterium subtile GCA_000741775.1
uC
628 Streptococcus mutans UA159 GCA_000007465.2
460 Streptococcus ratti FA-1 = DSM 20564 GCA_000286075.1
460 Streptococcus sanguinis SK36 GCA_000014205.1
456 Streptococcus ferus DSM 20646 GCA_000372425.1
uD
851 Clostridium beijerinckii GCA_000833105.2
821 Clostridium saccharoperbutylacetonicum N1-4_28HMT_29 GCA_000340885.1
810 Clostridium puniceum GCA_002006345.1
uE
764 Clostridium beijerinckii GCA_000833105.2
732 Clostridium saccharoperbutylacetonicum N1-4_28HMT_29 GCA_000340885.1
711 Clostridium saccharobutylicum DSM 13864 GCA_000473995.1
uF
701 Lactobacillus gasseri ATCC 33323 = JCM 1131 GCA_000014425.1
685 Lactobacillus hominis DSM 23910 = CRBIP 24.179 GCA_000296835.1
546 Lactobacillus jensenii GCA_001936235.1
uG
688 Clostridium beijerinckii GCA_000833105.2
656 Clostridium saccharobutylicum DSM 13864 GCA_000473995.1
654 Clostridium saccharoperbutylacetonicum N1-4_28HMT_29 GCA_000340885.1
uH
644 Lactobacillus gasseri ATCC 33323 = JCM 1131 GCA_000014425.1
617 Lactobacillus hominis DSM 23910 = CRBIP 24.179 GCA_000296835.1
460 Lactobacillus iners DSM 13335 GCA_000160875.1
uI
707 Clostridium beijerinckii GCA_000833105.2
666 Clostridium saccharoperbutylacetonicum N1-4_28HMT_29 GCA_000340885.1
624 Clostridium saccharobutylicum DSM 13864 GCA_000473995.1
uJ
513 Clostridium beijerinckii GCA_000833105.2
513 Clostridium saccharoperbutylacetonicum N1-4_28HMT_29 GCA_000340885.1
476 Clostridium puniceum GCA_002006345.1
454 Clostridium saccharobutylicum DSM 13864 GCA_000473995.1
uK
850 _5BBacillus thuringiensis_5D serovar konkukian str. 97-27 GCA_000008505.1
849 Bacillus anthracis str. Ames GCA_000007845.1
849 Bacillus anthracis str. Sterne GCA_000008165.1
834 Bacillus cereus ATCC 14579 GCA_000007825.1
uL

558 Staphylococcus epidermidis ATCC 12228 GCA_000007645.1
541 Staphylococcus capitis subsp. capitis GCA_001028645.1
537 Staphylococcus haemolyticus JCSC1435 GCA_000009865.1
537 Staphylococcus hominis subsp. hominis C80 GCA_000183685.1
537 Staphylococcus lugdunensis HKU09-01 GCA_000025085.1
uM
656 Enterococcus faecalis V583 GCA_000007785.1
609 Streptomyces cinnamoneus GCA_001885705.1
600 Enterococcus rivorum GCA_001742285.1
uN
458 Clostridium beijerinckii GCA_000833105.2
439 Clostridium saccharoperbutylacetonicum N1-4_28HMT_29 GCA_000340885.1
425 Clostridium puniceum GCA_002006345.1
uO
331 Escherichia coli O157_3AH7 str. Sakai GCA_000008865.1
331 Escherichia coli UMN026 GCA_000026325.2
331 Escherichia coli str. K-12 substr. MG1655 GCA_000005845.2
331 Shigella flexneri 2a str. 301 GCA_000006925.2
325 Escherichia coli O83_3AH1 str. NRG 857C GCA_000183345.1
315 Escherichia coli O104_3AH4 str. 2011C-3493 GCA_000299455.1
uP
701 Lactobacillus gasseri ATCC 33323 = JCM 1131 GCA_000014425.1
638 Lactobacillus hominis DSM 23910 = CRBIP 24.179 GCA_000296835.1
501 Lactobacillus psittaci DSM 15354 GCA_000425905.1
uQ
697 Staphylococcus epidermidis ATCC 12228 GCA_000007645.1
676 Staphylococcus capitis subsp. capitis GCA_001028645.1
648 Staphylococcus warneri SG1 GCA_000332735.1
uR
767 Deinococcus radiodurans R1 GCA_000008565.1
445 Deinococcus deserti VCD115 GCA_000020685.1
423 Deinococcus marmoris DSM 12784 GCA_000701405.1
uS
1022 Enterococcus faecalis V583 GCA_000007785.1
977 Streptomyces cinnamoneus GCA_001885705.1
896 Enterococcus rivorum GCA_001742285.1
uT
546 Rhodobacter sphaeroides 2.4.1 GCA_000012905.2
494 Rhodobacter sphaeroides ATCC 17025 GCA_000016405.1
434 Thioclava atlantica GCA_000737065.1
uU
508 Rhodobacter sphaeroides ATCC 17025 GCA_000016405.1
495 Rhodobacter sphaeroides 2.4.1 GCA_000012905.2
444 Pseudorhodobacter wandonensis GCA_001202035.1
uV
637 Staphylococcus epidermidis ATCC 12228 GCA_000007645.1
587 Staphylococcus capitis subsp. capitis GCA_001028645.1
558 Staphylococcus aureus subsp. aureus NCTC 8325 GCA_000013425.1
558 Staphylococcus warneri SG1 GCA_000332735.1
uW
913 Escherichia coli str. K-12 substr. MG1655 GCA_000005845.2
891 Escherichia coli O83_3AH1 str. NRG 857C GCA_000183345.1
888 Escherichia coli O157_3AH7 str. Sakai GCA_000008865.1
uX
774 Rhodobacter sphaeroides 2.4.1 GCA_000012905.2
682 Rhodobacter sphaeroides ATCC 17025 GCA_000016405.1
554 Pseudorhodobacter ferrugineus DSM 5888 GCA_000420745.1
uY
679 Rhodobacter sphaeroides 2.4.1 GCA_000012905.2
620 Rhodobacter sphaeroides ATCC 17025 GCA_000016405.1
553 Gemmobacter aquatilis GCA_900110025.1
uZ
562 Lactobacillus gasseri ATCC 33323 = JCM 1131 GCA_000014425.1
466 Lactobacillus hominis DSM 23910 = CRBIP 24.179 GCA_000296835.1
269 Lactobacillus hamsteri DSM 5661 = JCM 6256 GCA_000615445.1
v0
527 Rhodobacter sphaeroides 2.4.1 GCA_000012905.2
433 Rhodobacter sphaeroides ATCC 17025 GCA_000016405.1
412 Gemmobacter megaterium GCA_900156815.1
v1
990 Rhodobacter sphaeroides 2.4.1 GCA_000012905.2
912 Rhodobacter sphaeroides ATCC 17025 GCA_000016405.1
804 Gemmobacter aquatilis GCA_900110025.1
v2
536 Streptococcus mutans UA159 GCA_000007465.2
342 Streptococcus criceti HS-6 GCA_000187975.3

341 Streptococcus macacae NCTC 11558 GCA_000187995.3
v3
616 Clostridium beijerinckii GCA_000833105.2
584 Clostridium saccharobutylicum DSM 13864 GCA_000473995.1
584 Clostridium saccharoperbutylacetonicum N1-4_28HMT_29 GCA_000340885.1
569 Clostridium puniceum GCA_002006345.1
v4
595 Deinococcus radiodurans R1 GCA_000008565.1
393 Deinococcus deserti VCD115 GCA_000020685.1
382 Deinococcus gobiensis I-0 GCA_000252445.1
v5
763 Clostridium beijerinckii GCA_000833105.2
735 Clostridium saccharoperbutylacetonicum N1-4_28HMT_29 GCA_000340885.1
714 Clostridium saccharobutylicum DSM 13864 GCA_000473995.1
v6
670 Rhodobacter sphaeroides 2.4.1 GCA_000012905.2
622 Rhodobacter sphaeroides ATCC 17025 GCA_000016405.1
507 Gemmobacter aquatilis GCA_900110025.1
v7
566 Bacillus anthracis str. Ames GCA_000007845.1
566 Bacillus anthracis str. Sterne GCA_000008165.1
566 _5BBacillus thuringiensis_5D serovar konkukian str. 97-27 GCA_000008505.1
562 Bacillus pseudomycoides DSM 12442 GCA_000161455.1
547 Bacillus cereus ATCC 14579 GCA_000007825.1
v8
815 Staphylococcus epidermidis ATCC 12228 GCA_000007645.1
758 Staphylococcus capitis subsp. capitis GCA_001028645.1
745 Staphylococcus aureus subsp. aureus NCTC 8325 GCA_000013425.1
v9
790 Clostridium beijerinckii GCA_000833105.2
725 Clostridium saccharoperbutylacetonicum N1-4_28HMT_29 GCA_000340885.1
706 Clostridium saccharobutylicum DSM 13864 GCA_000473995.1
va
888 Staphylococcus epidermidis ATCC 12228 GCA_000007645.1
835 Staphylococcus capitis subsp. capitis GCA_001028645.1
759 Staphylococcus aureus subsp. aureus NCTC 8325 GCA_000013425.1
vb
699 Staphylococcus epidermidis ATCC 12228 GCA_000007645.1
648 Staphylococcus capitis subsp. capitis GCA_001028645.1
593 Staphylococcus haemolyticus JCSC1435 GCA_000009865.1
593 Staphylococcus hominis subsp. hominis C80 GCA_000183685.1
vc
623 Lactobacillus gasseri ATCC 33323 = JCM 1131 GCA_000014425.1
533 Lactobacillus hominis DSM 23910 = CRBIP 24.179 GCA_000296835.1
426 Lactobacillus apis GCA_000970735.1
vd
540 Enterococcus faecalis V583 GCA_000007785.1
499 Streptomyces cinnamoneus GCA_001885705.1
478 Enterococcus canis NBRC 100695 GCA_001544375.1
ve
437 Staphylococcus epidermidis ATCC 12228 GCA_000007645.1
416 Staphylococcus capitis subsp. capitis GCA_001028645.1
380 Staphylococcus haemolyticus JCSC1435 GCA_000009865.1
vf
558 Streptococcus mutans UA159 GCA_000007465.2
423 Streptococcus ratti FA-1 = DSM 20564 GCA_000286075.1
404 Streptococcus henryi DSM 19005 GCA_000376985.1
vg
626 Lactobacillus gasseri ATCC 33323 = JCM 1131 GCA_000014425.1
552 Lactobacillus hominis DSM 23910 = CRBIP 24.179 GCA_000296835.1
469 Lactobacillus iners DSM 13335 GCA_000160875.1
vh
750 Enterococcus faecalis V583 GCA_000007785.1
703 Streptomyces cinnamoneus GCA_001885705.1
636 Enterococcus rivorum GCA_001742285.1
vi
573 Deinococcus radiodurans R1 GCA_000008565.1
329 Deinococcus gobiensis I-0 GCA_000252445.1
290 Deinococcus deserti VCD115 GCA_000020685.1
vj
659 Enterococcus faecalis V583 GCA_000007785.1
627 Streptomyces cinnamoneus GCA_001885705.1
620 Enterococcus faecium DO GCA_000174395.2
vk
626 Streptococcus mutans UA159 GCA_000007465.2
442 Streptococcus gordonii str. Challis substr. CH1 GCA_000017005.1

393 Streptococcus iniae GCA_000831485.1
vl
516 Bacillus thuringiensis YBT-1518 GCA_000497525.2
501 Bacillus anthracis str. Ames GCA_000007845.1
501 Bacillus anthracis str. Sterne GCA_000008165.1
501 Bacillus cereus ATCC 14579 GCA_000007825.1
501 _5BBacillus thuringiensis_5D serovar konkukian str. 97-27 GCA_000008505.1
475 Bacillus pseudomycoides DSM 12442 GCA_000161455.1
vm
481 Streptococcus mutans UA159 GCA_000007465.2
408 Streptococcus ratti FA-1 = DSM 20564 GCA_000286075.1
372 Streptococcus henryi DSM 19005 GCA_000376985.1
vn
525 Bifidobacterium adolescentis ATCC 15703 GCA_000010425.1
406 Bifidobacterium dentium JCM 1195 = DSM 20436 GCA_001042595.1
391 Bifidobacterium angulatum DSM 20098 = JCM 7096 GCA_001025155.1
vo
679 Clostridium beijerinckii GCA_000833105.2
671 Clostridium saccharoperbutylacetonicum N1-4_28HMT_29 GCA_000340885.1
648 Clostridium saccharobutylicum DSM 13864 GCA_000473995.1
vp
729 Streptococcus mutans UA159 GCA_000007465.2
538 Streptococcus ratti FA-1 = DSM 20564 GCA_000286075.1
518 Streptococcus gordonii str. Challis substr. CH1 GCA_000017005.1
vq
678 Deinococcus radiodurans R1 GCA_000008565.1
469 Deinococcus gobiensis I-0 GCA_000252445.1
459 Deinococcus deserti VCD115 GCA_000020685.1
vr
297 Streptococcus mutans UA159 GCA_000007465.2
185 Streptococcus gordonii str. Challis substr. CH1 GCA_000017005.1
181 Streptococcus equinus GCA_000964315.1
181 Streptococcus gallolyticus subsp. gallolyticus DSM 16831 GCA_002000985.1
181 Streptococcus macacae NCTC 11558 GCA_000187995.3
181 Streptococcus salivarius GCA_000785515.1
181 Streptococcus sanguinis SK36 GCA_000014205.1
181 Streptococcus thermophilus JIM 8232 GCA_000253395.1
vs
641 Bifidobacterium adolescentis ATCC 15703 GCA_000010425.1
553 Bifidobacterium dentium JCM 1195 = DSM 20436 GCA_001042595.1
543 Bifidobacterium tsurumiense GCA_000741765.1
vt
879 Enterococcus faecalis V583 GCA_000007785.1
832 Streptomyces cinnamoneus GCA_001885705.1
747 Enterococcus canis NBRC 100695 GCA_001544375.1
vu
574 Rhodobacter sphaeroides 2.4.1 GCA_000012905.2
510 Rhodobacter sphaeroides ATCC 17025 GCA_000016405.1
464 Gemmobacter aquatilis GCA_900110025.1
vv
781 Lactobacillus gasseri ATCC 33323 = JCM 1131 GCA_000014425.1
737 Lactobacillus hominis DSM 23910 = CRBIP 24.179 GCA_000296835.1
609 Lactobacillus iners DSM 13335 GCA_000160875.1
vw
612 Rhodobacter sphaeroides 2.4.1 GCA_000012905.2
538 Rhodobacter sphaeroides ATCC 17025 GCA_000016405.1
450 Gemmobacter aquatilis GCA_900110025.1
vx
698 Escherichia coli str. K-12 substr. MG1655 GCA_000005845.2
685 Escherichia coli O157_3AH7 str. Sakai GCA_000008865.1
685 Escherichia coli O83_3AH1 str. NRG 857C GCA_000183345.1
685 Escherichia coli UMN026 GCA_000026325.2
685 Shigella flexneri 2a str. 301 GCA_000006925.2
670 Escherichia coli IAI39 GCA_000026345.1
vy
591 Staphylococcus epidermidis ATCC 12228 GCA_000007645.1
556 Staphylococcus capitis subsp. capitis GCA_001028645.1
505 Staphylococcus warneri SG1 GCA_000332735.1
vz
539 Staphylococcus epidermidis ATCC 12228 GCA_000007645.1
520 Staphylococcus capitis subsp. capitis GCA_001028645.1
514 Staphylococcus haemolyticus JCSC1435 GCA_000009865.1
vA
267 Rhodobacter sphaeroides ATCC 17025 GCA_000016405.1
266 Rhodobacter sphaeroides 2.4.1 GCA_000012905.2
218 Rhodobacter aestuarii GCA_900156655.1

vB
634 Escherichia coli UMN026 GCA_000026325.2
634 Escherichia coli str. K-12 substr. MG1655 GCA_000005845.2
621 Escherichia coli O157_3AH7 str. Sakai GCA_000008865.1
619 Shigella flexneri 2a str. 301 GCA_000006925.2
vC
943 Lactobacillus gasseri ATCC 33323 = JCM 1131 GCA_000014425.1
902 Lactobacillus hominis DSM 23910 = CRBIP 24.179 GCA_000296835.1
678 Lactobacillus iners DSM 13335 GCA_000160875.1
vD
614 Staphylococcus epidermidis ATCC 12228 GCA_000007645.1
609 Staphylococcus capitis subsp. capitis GCA_001028645.1
568 Staphylococcus hominis subsp. hominis C80 GCA_000183685.1
vE
418 Streptococcus mutans UA159 GCA_000007465.2
327 Streptococcus merionis DSM 19192 GCA_000380085.1
321 Streptococcus gordonii str. Challis substr. CH1 GCA_000017005.1
vF
763 Lactobacillus gasseri ATCC 33323 = JCM 1131 GCA_000014425.1
729 Lactobacillus hominis DSM 23910 = CRBIP 24.179 GCA_000296835.1
518 Lactobacillus iners DSM 13335 GCA_000160875.1
vG
628 Escherichia coli str. K-12 substr. MG1655 GCA_000005845.2
623 Escherichia coli IAI39 GCA_000026345.1
617 Escherichia coli UMN026 GCA_000026325.2
vH
747 Clostridium beijerinckii GCA_000833105.2
703 Clostridium saccharoperbutylacetonicum N1-4_28HMT_29 GCA_000340885.1
675 Clostridium saccharobutylicum DSM 13864 GCA_000473995.1
vI
580 Rhodobacter sphaeroides 2.4.1 GCA_000012905.2
520 Rhodobacter sphaeroides ATCC 17025 GCA_000016405.1
374 Pseudorhodobacter ferrugineus DSM 5888 GCA_000420745.1
vJ
837 Escherichia coli str. K-12 substr. MG1655 GCA_000005845.2
832 Escherichia coli UMN026 GCA_000026325.2
830 Escherichia coli O104_3AH4 str. 2011C-3493 GCA_000299455.1
830 Shigella flexneri 2a str. 301 GCA_000006925.2
vK
838 Streptococcus mutans UA159 GCA_000007465.2
600 Streptococcus ratti FA-1 = DSM 20564 GCA_000286075.1
559 Streptococcus gallolyticus subsp. gallolyticus DSM 16831 GCA_002000985.1
vL
762 Clostridium beijerinckii GCA_000833105.2
725 Clostridium saccharoperbutylacetonicum N1-4_28HMT_29 GCA_000340885.1
703 Clostridium puniceum GCA_002006345.1
vM
863 Bifidobacterium adolescentis ATCC 15703 GCA_000010425.1
743 Bifidobacterium dentium JCM 1195 = DSM 20436 GCA_001042595.1
668 Bifidobacterium angulatum DSM 20098 = JCM 7096 GCA_001025155.1
vN
694 Escherichia coli O104_3AH4 str. 2011C-3493 GCA_000299455.1
693 Escherichia coli str. K-12 substr. MG1655 GCA_000005845.2
671 Shigella flexneri 2a str. 301 GCA_000006925.2
vO
646 Staphylococcus epidermidis ATCC 12228 GCA_000007645.1
601 Staphylococcus capitis subsp. capitis GCA_001028645.1
595 Staphylococcus aureus subsp. aureus NCTC 8325 GCA_000013425.1
vP
886 Clostridium beijerinckii GCA_000833105.2
872 Clostridium saccharoperbutylacetonicum N1-4_28HMT_29 GCA_000340885.1
851 Clostridium saccharobutylicum DSM 13864 GCA_000473995.1
vQ
471 Streptococcus mutans UA159 GCA_000007465.2
375 Streptococcus ratti FA-1 = DSM 20564 GCA_000286075.1
358 Streptococcus macacae NCTC 11558 GCA_000187995.3
vR
420 Rhodobacter sphaeroides 2.4.1 GCA_000012905.2
382 Rhodobacter sphaeroides ATCC 17025 GCA_000016405.1
316 Defluviimonas alba GCA_001620265.1
vS
642 Deinococcus radiodurans R1 GCA_000008565.1
437 Deinococcus deserti VCD115 GCA_000020685.1
414 Deinococcus puniceus GCA_001644565.1
vT
845 Escherichia coli str. K-12 substr. MG1655 GCA_000005845.2

821 Escherichia coli O104_3AH4 str. 2011C-3493 GCA_000299455.1
821 Shigella flexneri 2a str. 301 GCA_000006925.2
796 Escherichia coli O157_3AH7 str. Sakai GCA_000008865.1
796 Escherichia coli UMN026 GCA_000026325.2
vU
638 Staphylococcus epidermidis ATCC 12228 GCA_000007645.1
581 Staphylococcus capitis subsp. capitis GCA_001028645.1
524 Staphylococcus hominis subsp. hominis C80 GCA_000183685.1
vV
487 Clostridium beijerinckii GCA_000833105.2
463 Clostridium puniceum GCA_002006345.1
463 Clostridium saccharoperbutylacetonicum N1-4_28HMT_29 GCA_000340885.1
445 Clostridium saccharobutylicum DSM 13864 GCA_000473995.1
vW
329 Rhodobacter sphaeroides 2.4.1 GCA_000012905.2
291 Rhodobacter sphaeroides ATCC 17025 GCA_000016405.1
274 Paracoccus solventivorans GCA_900142875.1
vX
662 Enterococcus faecalis V583 GCA_000007785.1
615 Streptomyces cinnamoneus GCA_001885705.1
610 Staphylococcus epidermidis ATCC 12228 GCA_000007645.1
vY
508 Streptococcus mutans UA159 GCA_000007465.2
362 Streptococcus ratti FA-1 = DSM 20564 GCA_000286075.1
352 Streptococcus sobrinus DSM 20742 = ATCC 33478 GCA_000686605.1
vZ
489 Clostridium beijerinckii GCA_000833105.2
474 Clostridium saccharoperbutylacetonicum N1-4_28HMT_29 GCA_000340885.1
453 Clostridium puniceum GCA_002006345.1
w0
656 Rhodobacter sphaeroides 2.4.1 GCA_000012905.2
595 Rhodobacter sphaeroides ATCC 17025 GCA_000016405.1
519 Gemmobacter aquatilis GCA_900110025.1
w1
642 Enterococcus faecalis V583 GCA_000007785.1
602 Enterococcus rivorum GCA_001742285.1
595 Streptomyces cinnamoneus GCA_001885705.1
w2
855 Deinococcus radiodurans R1 GCA_000008565.1
535 Deinococcus deserti VCD115 GCA_000020685.1
511 Deinococcus gobiensis I-0 GCA_000252445.1
w3
589 Deinococcus radiodurans R1 GCA_000008565.1
400 Deinococcus deserti VCD115 GCA_000020685.1
391 Deinococcus gobiensis I-0 GCA_000252445.1
w4
699 Lactobacillus gasseri ATCC 33323 = JCM 1131 GCA_000014425.1
679 Lactobacillus hominis DSM 23910 = CRBIP 24.179 GCA_000296835.1
492 Lactobacillus iners DSM 13335 GCA_000160875.1
w5
768 Enterococcus faecalis V583 GCA_000007785.1
722 Streptomyces cinnamoneus GCA_001885705.1
674 Enterococcus rivorum GCA_001742285.1
w6
859 Enterococcus faecalis V583 GCA_000007785.1
812 Streptomyces cinnamoneus GCA_001885705.1
736 Enterococcus canis NBRC 100695 GCA_001544375.1
w7
801 Rhodobacter sphaeroides 2.4.1 GCA_000012905.2
708 Rhodobacter sphaeroides ATCC 17025 GCA_000016405.1
591 Pseudorhodobacter ferrugineus DSM 5888 GCA_000420745.1
w8
710 Escherichia coli O157_3AH7 str. Sakai GCA_000008865.1
710 Escherichia coli UMN026 GCA_000026325.2
710 Escherichia coli str. K-12 substr. MG1655 GCA_000005845.2
706 Escherichia coli O104_3AH4 str. 2011C-3493 GCA_000299455.1
706 Escherichia coli O83_3AH1 str. NRG 857C GCA_000183345.1
706 Shigella flexneri 2a str. 301 GCA_000006925.2
676 Escherichia coli IAI39 GCA_000026345.1
676 Shigella dysenteriae Sd197 GCA_000012005.1
w9
839 Enterococcus faecalis V583 GCA_000007785.1
792 Streptomyces cinnamoneus GCA_001885705.1
672 Enterococcus rivorum GCA_001742285.1
wa
915 Clostridium beijerinckii GCA_000833105.2

842 Clostridium saccharobutylicum DSM 13864 GCA_000473995.1
841 Clostridium saccharoperbutylacetonicum N1-4_28HMT_29 GCA_000340885.1
wb
566 Escherichia coli O104_3AH4 str. 2011C-3493 GCA_000299455.1
566 Escherichia coli str. K-12 substr. MG1655 GCA_000005845.2
559 Shigella flexneri 2a str. 301 GCA_000006925.2
557 Escherichia coli UMN026 GCA_000026325.2
wc
420 Enterococcus faecalis V583 GCA_000007785.1
373 Streptomyces cinnamoneus GCA_001885705.1
356 Enterococcus haemoperoxidus ATCC BAA-382 GCA_000407165.1
wd
766 Bacillus anthracis str. Ames GCA_000007845.1
766 Bacillus anthracis str. Sterne GCA_000008165.1
766 _5BBacillus thuringiensis_5D serovar konkukian str. 97-27 GCA_000008505.1
740 Bacillus cereus ATCC 14579 GCA_000007825.1
734 Bacillus pseudomycoides DSM 12442 GCA_000161455.1
we
685 Deinococcus radiodurans R1 GCA_000008565.1
390 Deinococcus deserti VCD115 GCA_000020685.1
382 Deinococcus murrayi DSM 11303 GCA_000482805.1
wf
747 Deinococcus radiodurans R1 GCA_000008565.1
436 Deinococcus deserti VCD115 GCA_000020685.1
421 Deinococcus gobiensis I-0 GCA_000252445.1
wg
700 Streptococcus mutans UA159 GCA_000007465.2
526 Streptococcus ratti FA-1 = DSM 20564 GCA_000286075.1
518 Streptococcus gordonii str. Challis substr. CH1 GCA_000017005.1
wh
808 Bifidobacterium adolescentis ATCC 15703 GCA_000010425.1
720 Bifidobacterium dentium JCM 1195 = DSM 20436 GCA_001042595.1
673 Bifidobacterium angulatum DSM 20098 = JCM 7096 GCA_001025155.1
wi
698 Streptococcus mutans UA159 GCA_000007465.2
479 Streptococcus ratti FA-1 = DSM 20564 GCA_000286075.1
449 Streptococcus equinus GCA_000964315.1
wj
772 Escherichia coli O104_3AH4 str. 2011C-3493 GCA_000299455.1
772 Escherichia coli str. K-12 substr. MG1655 GCA_000005845.2
758 Shigella flexneri 2a str. 301 GCA_000006925.2
741 Escherichia coli O157_3AH7 str. Sakai GCA_000008865.1
wk
705 Lactobacillus gasseri ATCC 33323 = JCM 1131 GCA_000014425.1
645 Lactobacillus hominis DSM 23910 = CRBIP 24.179 GCA_000296835.1
472 Lactobacillus iners DSM 13335 GCA_000160875.1
wl
761 Deinococcus radiodurans R1 GCA_000008565.1
445 Deinococcus deserti VCD115 GCA_000020685.1
436 Deinococcus puniceus GCA_001644565.1
wm
767 Lactobacillus gasseri ATCC 33323 = JCM 1131 GCA_000014425.1
682 Lactobacillus hominis DSM 23910 = CRBIP 24.179 GCA_000296835.1
546 Lactobacillus iners DSM 13335 GCA_000160875.1
wn
577 Clostridium beijerinckii GCA_000833105.2
534 Clostridium saccharoperbutylacetonicum N1-4_28HMT_29 GCA_000340885.1
517 Clostridium puniceum GCA_002006345.1
wo
503 Lactobacillus gasseri ATCC 33323 = JCM 1131 GCA_000014425.1
455 Lactobacillus hominis DSM 23910 = CRBIP 24.179 GCA_000296835.1
309 Lactobacillus hamsteri DSM 5661 = JCM 6256 GCA_000615445.1
wp
850 Deinococcus radiodurans R1 GCA_000008565.1
562 Deinococcus deserti VCD115 GCA_000020685.1
542 Deinococcus puniceus GCA_001644565.1
wq
711 Bifidobacterium adolescentis ATCC 15703 GCA_000010425.1
638 Bifidobacterium dentium JCM 1195 = DSM 20436 GCA_001042595.1
585 Bifidobacterium angulatum DSM 20098 = JCM 7096 GCA_001025155.1
wr
842 Staphylococcus epidermidis ATCC 12228 GCA_000007645.1
797 Staphylococcus capitis subsp. capitis GCA_001028645.1
736 Staphylococcus hominis subsp. hominis C80 GCA_000183685.1
ws
565 Staphylococcus epidermidis ATCC 12228 GCA_000007645.1

536 Staphylococcus capitis subsp. capitis GCA_001028645.1
518 Staphylococcus warneri SG1 GCA_000332735.1
wt
667 Deinococcus radiodurans R1 GCA_000008565.1
442 Deinococcus deserti VCD115 GCA_000020685.1
396 Deinococcus soli Cha et al. 2016 GCA_001007995.1
wu
615 Bifidobacterium adolescentis ATCC 15703 GCA_000010425.1
504 Bifidobacterium dentium JCM 1195 = DSM 20436 GCA_001042595.1
451 Bifidobacterium angulatum DSM 20098 = JCM 7096 GCA_001025155.1
wv
806 Deinococcus radiodurans R1 GCA_000008565.1
584 Deinococcus deserti VCD115 GCA_000020685.1
550 Deinococcus gobiensis I-0 GCA_000252445.1
ww
580 Staphylococcus epidermidis ATCC 12228 GCA_000007645.1
534 Staphylococcus capitis subsp. capitis GCA_001028645.1
483 Staphylococcus warneri SG1 GCA_000332735.1
wx
770 Staphylococcus epidermidis ATCC 12228 GCA_000007645.1
724 Staphylococcus capitis subsp. capitis GCA_001028645.1
696 Staphylococcus haemolyticus JCSC1435 GCA_000009865.1
wy
548 Staphylococcus epidermidis ATCC 12228 GCA_000007645.1
487 Staphylococcus capitis subsp. capitis GCA_001028645.1
419 Staphylococcus hominis subsp. hominis C80 GCA_000183685.1
wz
474 Streptococcus mutans UA159 GCA_000007465.2
344 Streptococcus ratti FA-1 = DSM 20564 GCA_000286075.1
296 Streptococcus gordonii str. Challis substr. CH1 GCA_000017005.1
wA
970 Deinococcus radiodurans R1 GCA_000008565.1
612 Deinococcus deserti VCD115 GCA_000020685.1
582 Deinococcus gobiensis I-0 GCA_000252445.1
wB
765 Enterococcus faecalis V583 GCA_000007785.1
718 Streptomyces cinnamoneus GCA_001885705.1
652 Enterococcus thailandicus GCA_001652875.1
wC
473 Escherichia coli str. K-12 substr. MG1655 GCA_000005845.2
458 Escherichia coli O157_3AH7 str. Sakai GCA_000008865.1
458 Shigella flexneri 2a str. 301 GCA_000006925.2
455 Escherichia coli O83_3AH1 str. NRG 857C GCA_000183345.1
455 Escherichia coli UMN026 GCA_000026325.2
wD
381 Lactobacillus gasseri ATCC 33323 = JCM 1131 GCA_000014425.1
316 Lactobacillus hominis DSM 23910 = CRBIP 24.179 GCA_000296835.1
276 Lactobacillus iners DSM 13335 GCA_000160875.1
wE
525 Clostridium beijerinckii GCA_000833105.2
473 Clostridium saccharoperbutylacetonicum N1-4_28HMT_29 GCA_000340885.1
467 Clostridium puniceum GCA_002006345.1
wF
688 Clostridium beijerinckii GCA_000833105.2
648 Clostridium saccharoperbutylacetonicum N1-4_28HMT_29 GCA_000340885.1
624 Clostridium saccharobutylicum DSM 13864 GCA_000473995.1
wG
817 Staphylococcus epidermidis ATCC 12228 GCA_000007645.1
779 Staphylococcus capitis subsp. capitis GCA_001028645.1
746 Staphylococcus pettenkoferi GCA_002208805.1
wH
711 Deinococcus radiodurans R1 GCA_000008565.1
514 Deinococcus deserti VCD115 GCA_000020685.1
498 Deinococcus gobiensis I-0 GCA_000252445.1
wI
760 Lactobacillus gasseri ATCC 33323 = JCM 1131 GCA_000014425.1
693 Lactobacillus hominis DSM 23910 = CRBIP 24.179 GCA_000296835.1
552 Lactobacillus iners DSM 13335 GCA_000160875.1
wJ
850 Rhodobacter sphaeroides 2.4.1 GCA_000012905.2
766 Rhodobacter sphaeroides ATCC 17025 GCA_000016405.1
666 Defluviimonas alba GCA_001620265.1
wK
735 Staphylococcus epidermidis ATCC 12228 GCA_000007645.1
686 Staphylococcus capitis subsp. capitis GCA_001028645.1
656 Staphylococcus warneri SG1 GCA_000332735.1

wL
611 Clostridium beijerinckii GCA_000833105.2
584 Clostridium saccharoperbutylacetonicum N1-4_28HMT_29 GCA_000340885.1
554 Clostridium puniceum GCA_002006345.1
wM
826 _5BBacillus thuringiensis_5D serovar konkukian str. 97-27 GCA_000008505.1
825 Bacillus anthracis str. Ames GCA_000007845.1
825 Bacillus anthracis str. Sterne GCA_000008165.1
825 Bacillus cereus ATCC 14579 GCA_000007825.1
825 Bacillus thuringiensis YBT-1518 GCA_000497525.2
809 Bacillus mycoides GCA_000832605.1
809 Bacillus pseudomycoides DSM 12442 GCA_000161455.1
wN
638 Streptococcus mutans UA159 GCA_000007465.2
454 Streptococcus sobrinus DSM 20742 = ATCC 33478 GCA_000686605.1
421 Streptococcus dysgalactiae subsp. equisimilis AC-2713 GCA_000317855.1
wO
743 Rhodobacter sphaeroides 2.4.1 GCA_000012905.2
619 Rhodobacter sphaeroides ATCC 17025 GCA_000016405.1
534 Thioclava indica GCA_000714545.1
wP
791 Streptococcus mutans UA159 GCA_000007465.2
554 Streptococcus ratti FA-1 = DSM 20564 GCA_000286075.1
522 Streptococcus gordonii str. Challis substr. CH1 GCA_000017005.1
wQ
694 _5BBacillus thuringiensis_5D serovar konkukian str. 97-27 GCA_000008505.1
683 Bacillus anthracis str. Ames GCA_000007845.1
683 Bacillus anthracis str. Sterne GCA_000008165.1
653 Bacillus cereus ATCC 14579 GCA_000007825.1
653 Bacillus thuringiensis YBT-1518 GCA_000497525.2
wR
458 Lactobacillus gasseri ATCC 33323 = JCM 1131 GCA_000014425.1
403 Lactobacillus hominis DSM 23910 = CRBIP 24.179 GCA_000296835.1
300 Lactobacillus amylolyticus GCA_002075105.1
300 Lactobacillus hamsteri DSM 5661 = JCM 6256 GCA_000615445.1
wS
784 Enterococcus faecalis V583 GCA_000007785.1
737 Streptomyces cinnamoneus GCA_001885705.1
659 Enterococcus asini ATCC 700915 GCA_000407365.1
wT
473 Clostridium beijerinckii GCA_000833105.2
437 Clostridium saccharoperbutylacetonicum N1-4_28HMT_29 GCA_000340885.1
423 Clostridium saccharobutylicum DSM 13864 GCA_000473995.1
wU
652 Streptococcus mutans UA159 GCA_000007465.2
486 Streptococcus ratti FA-1 = DSM 20564 GCA_000286075.1
462 Streptococcus gordonii str. Challis substr. CH1 GCA_000017005.1
wV
729 Clostridium beijerinckii GCA_000833105.2
695 Clostridium saccharobutylicum DSM 13864 GCA_000473995.1
679 Clostridium saccharoperbutylacetonicum N1-4_28HMT_29 GCA_000340885.1
wW
916 Streptococcus mutans UA159 GCA_000007465.2
769 Streptococcus ratti FA-1 = DSM 20564 GCA_000286075.1
667 Streptococcus ferus DSM 20646 GCA_000372425.1
wX
823 Staphylococcus epidermidis ATCC 12228 GCA_000007645.1
772 Staphylococcus capitis subsp. capitis GCA_001028645.1
700 Staphylococcus lugdunensis HKU09-01 GCA_000025085.1
wY
805 Rhodobacter sphaeroides 2.4.1 GCA_000012905.2
698 Rhodobacter sphaeroides ATCC 17025 GCA_000016405.1
585 Gemmobacter megaterium GCA_900156815.1
wZ
562 Escherichia coli str. K-12 substr. MG1655 GCA_000005845.2
551 Escherichia coli O104_3AH4 str. 2011C-3493 GCA_000299455.1
536 Escherichia coli IAI39 GCA_000026345.1
536 Escherichia coli UMN026 GCA_000026325.2
x0
571 Rhodobacter sphaeroides 2.4.1 GCA_000012905.2
548 Rhodobacter sphaeroides ATCC 17025 GCA_000016405.1
399 Gemmobacter megaterium GCA_900156815.1
x1
705 Deinococcus radiodurans R1 GCA_000008565.1
511 Deinococcus deserti VCD115 GCA_000020685.1
493 Deinococcus gobiensis I-0 GCA_000252445.1

x2
623 Rhodobacter sphaeroides 2.4.1 GCA_000012905.2
561 Rhodobacter sphaeroides ATCC 17025 GCA_000016405.1
515 Gemmobacter aquatilis GCA_900110025.1
x3
486 Rhodobacter sphaeroides 2.4.1 GCA_000012905.2
411 Rhodobacter sphaeroides ATCC 17025 GCA_000016405.1
377 Pseudorhodobacter psychrotolerans GCA_001294535.1
x4
652 Rhodobacter sphaeroides 2.4.1 GCA_000012905.2
602 Rhodobacter sphaeroides ATCC 17025 GCA_000016405.1
497 Gemmobacter aquatilis GCA_900110025.1
x5
380 Rhodobacter sphaeroides 2.4.1 GCA_000012905.2
364 Rhodobacter sphaeroides ATCC 17025 GCA_000016405.1
331 Defluviimonas alba GCA_001620265.1
x6
461 Clostridium beijerinckii GCA_000833105.2
460 Clostridium saccharoperbutylacetonicum N1-4_28HMT_29 GCA_000340885.1
436 Clostridium puniceum GCA_002006345.1
x7
676 Bifidobacterium adolescentis ATCC 15703 GCA_000010425.1
565 Bifidobacterium dentium JCM 1195 = DSM 20436 GCA_001042595.1
529 Bifidobacterium callitrichos DSM 23973 GCA_000741175.1
x8
756 Clostridium beijerinckii GCA_000833105.2
738 Clostridium saccharoperbutylacetonicum N1-4_28HMT_29 GCA_000340885.1
722 Clostridium puniceum GCA_002006345.1
x9
768 Clostridium beijerinckii GCA_000833105.2
725 Clostridium saccharoperbutylacetonicum N1-4_28HMT_29 GCA_000340885.1
712 Clostridium saccharobutylicum DSM 13864 GCA_000473995.1
xa
743 Clostridium beijerinckii GCA_000833105.2
691 Clostridium saccharoperbutylacetonicum N1-4_28HMT_29 GCA_000340885.1
663 Clostridium puniceum GCA_002006345.1
xb
697 Staphylococcus epidermidis ATCC 12228 GCA_000007645.1
651 Staphylococcus capitis subsp. capitis GCA_001028645.1
605 Staphylococcus aureus subsp. aureus NCTC 8325 GCA_000013425.1
xc
554 Staphylococcus epidermidis ATCC 12228 GCA_000007645.1
540 Staphylococcus capitis subsp. capitis GCA_001028645.1
479 Staphylococcus warneri SG1 GCA_000332735.1
xd
798 Clostridium beijerinckii GCA_000833105.2
783 Clostridium saccharoperbutylacetonicum N1-4_28HMT_29 GCA_000340885.1
768 Clostridium saccharobutylicum DSM 13864 GCA_000473995.1
xe
696 Clostridium beijerinckii GCA_000833105.2
676 Clostridium saccharoperbutylacetonicum N1-4_28HMT_29 GCA_000340885.1
647 Clostridium saccharobutylicum DSM 13864 GCA_000473995.1
xf
475 Rhodobacter sphaeroides 2.4.1 GCA_000012905.2
415 Rhodobacter sphaeroides ATCC 17025 GCA_000016405.1
351 Gemmobacter aquatilis GCA_900110025.1
xg
660 Deinococcus radiodurans R1 GCA_000008565.1
463 Deinococcus deserti VCD115 GCA_000020685.1
427 Deinococcus soli Cha et al. 2016 GCA_001007995.1
xh
902 Lactobacillus gasseri ATCC 33323 = JCM 1131 GCA_000014425.1
815 Lactobacillus hominis DSM 23910 = CRBIP 24.179 GCA_000296835.1
536 Lactobacillus psittaci DSM 15354 GCA_000425905.1
xi
449 Rhodobacter sphaeroides 2.4.1 GCA_000012905.2
379 Rhodobacter sphaeroides ATCC 17025 GCA_000016405.1
281 Thioclava indica GCA_000714545.1
xj
913 Rhodobacter sphaeroides 2.4.1 GCA_000012905.2
860 Rhodobacter sphaeroides ATCC 17025 GCA_000016405.1
739 Gemmobacter aquatilis GCA_900110025.1
xk
836 Escherichia coli O157_3AH7 str. Sakai GCA_000008865.1
836 Escherichia coli str. K-12 substr. MG1655 GCA_000005845.2
830 Escherichia coli UMN026 GCA_000026325.2

821 Shigella flexneri 2a str. 301 GCA_000006925.2
xl
839 Streptococcus mutans UA159 GCA_000007465.2
655 Streptococcus ratti FA-1 = DSM 20564 GCA_000286075.1
629 Streptococcus gordonii str. Challis substr. CH1 GCA_000017005.1
xm
532 Lactobacillus gasseri ATCC 33323 = JCM 1131 GCA_000014425.1
492 Lactobacillus hominis DSM 23910 = CRBIP 24.179 GCA_000296835.1
380 Lactobacillus iners DSM 13335 GCA_000160875.1
xn
705 Bacillus anthracis str. Ames GCA_000007845.1
705 Bacillus anthracis str. Sterne GCA_000008165.1
705 Bacillus cereus ATCC 14579 GCA_000007825.1
705 _5BBacillus thuringiensis_5D serovar konkukian str. 97-27 GCA_000008505.1
695 Bacillus thuringiensis YBT-1518 GCA_000497525.2
671 Bacillus pseudomycoides DSM 12442 GCA_000161455.1
xo
854 Staphylococcus epidermidis ATCC 12228 GCA_000007645.1
831 Staphylococcus capitis subsp. capitis GCA_001028645.1
788 Staphylococcus aureus subsp. aureus NCTC 8325 GCA_000013425.1
xp
738 Deinococcus radiodurans R1 GCA_000008565.1
518 Deinococcus gobiensis I-0 GCA_000252445.1
475 Deinococcus deserti VCD115 GCA_000020685.1
xq
617 Deinococcus radiodurans R1 GCA_000008565.1
384 Deinococcus gobiensis I-0 GCA_000252445.1
372 Deinococcus hopiensis KR-140 GCA_900176165.1
xr
807 Clostridium beijerinckii GCA_000833105.2
743 Clostridium saccharoperbutylacetonicum N1-4_28HMT_29 GCA_000340885.1
738 Clostridium puniceum GCA_002006345.1
xs
683 Enterococcus faecalis V583 GCA_000007785.1
636 Streptomyces cinnamoneus GCA_001885705.1
565 Enterococcus faecium DO GCA_000174395.2
xt
698 Clostridium beijerinckii GCA_000833105.2
621 Clostridium saccharoperbutylacetonicum N1-4_28HMT_29 GCA_000340885.1
607 Clostridium saccharobutylicum DSM 13864 GCA_000473995.1
xu
538 Rhodobacter sphaeroides 2.4.1 GCA_000012905.2
478 Rhodobacter sphaeroides ATCC 17025 GCA_000016405.1
367 Pseudorhodobacter ferrugineus DSM 5888 GCA_000420745.1
xv
514 Staphylococcus epidermidis ATCC 12228 GCA_000007645.1
493 Staphylococcus capitis subsp. capitis GCA_001028645.1
481 Megasphaera cerevisiae DSM 20462 GCA_001045675.1
481 Staphylococcus warneri SG1 GCA_000332735.1
xw
661 Bacillus anthracis str. Ames GCA_000007845.1
661 Bacillus anthracis str. Sterne GCA_000008165.1
661 _5BBacillus thuringiensis_5D serovar konkukian str. 97-27 GCA_000008505.1
651 Bacillus cereus ATCC 14579 GCA_000007825.1
645 Bacillus thuringiensis YBT-1518 GCA_000497525.2
xx
414 Rhodobacter sphaeroides 2.4.1 GCA_000012905.2
327 Rhodobacter sphaeroides ATCC 17025 GCA_000016405.1
313 Defluviimonas alba GCA_001620265.1
xy
645 Lactobacillus gasseri ATCC 33323 = JCM 1131 GCA_000014425.1
623 Lactobacillus hominis DSM 23910 = CRBIP 24.179 GCA_000296835.1
483 Lactobacillus iners DSM 13335 GCA_000160875.1
xz
744 Streptococcus mutans UA159 GCA_000007465.2
582 Streptococcus ratti FA-1 = DSM 20564 GCA_000286075.1
526 Streptococcus gordonii str. Challis substr. CH1 GCA_000017005.1
xA
823 Streptococcus mutans UA159 GCA_000007465.2
647 Streptococcus ratti FA-1 = DSM 20564 GCA_000286075.1
619 Streptococcus gordonii str. Challis substr. CH1 GCA_000017005.1
xB
814 Enterococcus faecalis V583 GCA_000007785.1
767 Streptomyces cinnamoneus GCA_001885705.1
728 Enterococcus rivorum GCA_001742285.1
xC

506 Rhodobacter sphaeroides 2.4.1 GCA_000012905.2
490 Rhodobacter sphaeroides ATCC 17025 GCA_000016405.1
391 Gemmobacter megaterium GCA_900156815.1
xD
443 Deinococcus radiodurans R1 GCA_000008565.1
305 Deinococcus gobiensis I-0 GCA_000252445.1
287 Deinococcus proteolyticus MRP GCA_000190555.1
xE
505 _5BBacillus thuringiensis_5D serovar konkukian str. 97-27 GCA_000008505.1
491 Bacillus anthracis str. Ames GCA_000007845.1
491 Bacillus anthracis str. Sterne GCA_000008165.1
491 Bacillus cereus ATCC 14579 GCA_000007825.1
491 Bacillus thuringiensis YBT-1518 GCA_000497525.2
476 Bacillus mycoides GCA_000832605.1
xF
336 Deinococcus radiodurans R1 GCA_000008565.1
239 Deinococcus gobiensis I-0 GCA_000252445.1
215 Deinococcus soli Cha et al. 2016 GCA_001007995.1
xG
738 Streptococcus mutans UA159 GCA_000007465.2
576 Streptococcus gordonii str. Challis substr. CH1 GCA_000017005.1
557 Streptococcus ratti FA-1 = DSM 20564 GCA_000286075.1
xH
652 Rhodobacter sphaeroides 2.4.1 GCA_000012905.2
584 Rhodobacter sphaeroides ATCC 17025 GCA_000016405.1
465 Gemmobacter megaterium GCA_900156815.1
xI
274 Deinococcus radiodurans R1 GCA_000008565.1
145 Deinococcus deserti VCD115 GCA_000020685.1
138 Deinococcus hopiensis KR-140 GCA_900176165.1
xJ
711 Staphylococcus epidermidis ATCC 12228 GCA_000007645.1
680 Staphylococcus capitis subsp. capitis GCA_001028645.1
642 Staphylococcus warneri SG1 GCA_000332735.1
xK
516 Deinococcus radiodurans R1 GCA_000008565.1
326 Deinococcus deserti VCD115 GCA_000020685.1
301 Deinococcus soli Cha et al. 2016 GCA_001007995.1
xL
753 Deinococcus radiodurans R1 GCA_000008565.1
508 Deinococcus deserti VCD115 GCA_000020685.1
482 Deinococcus soli Cha et al. 2016 GCA_001007995.1
xM
613 Clostridium beijerinckii GCA_000833105.2
600 Clostridium saccharoperbutylacetonicum N1-4_28HMT_29 GCA_000340885.1
584 Clostridium saccharobutylicum DSM 13864 GCA_000473995.1
xN
773 Staphylococcus epidermidis ATCC 12228 GCA_000007645.1
753 Staphylococcus capitis subsp. capitis GCA_001028645.1
717 Staphylococcus aureus subsp. aureus NCTC 8325 GCA_000013425.1
xO
430 Clostridium beijerinckii GCA_000833105.2
391 Clostridium saccharoperbutylacetonicum N1-4_28HMT_29 GCA_000340885.1
386 Clostridium neonatale GCA_001458595.1
xP
695 Lactobacillus gasseri ATCC 33323 = JCM 1131 GCA_000014425.1
621 Lactobacillus hominis DSM 23910 = CRBIP 24.179 GCA_000296835.1
486 Lactobacillus jensenii GCA_001936235.1
486 Lactobacillus psittaci DSM 15354 GCA_000425905.1
xQ
627 Clostridium beijerinckii GCA_000833105.2
579 Clostridium puniceum GCA_002006345.1
579 Clostridium saccharoperbutylacetonicum N1-4_28HMT_29 GCA_000340885.1
576 Clostridium butyricum GCA_001456065.2
xR
678 Clostridium beijerinckii GCA_000833105.2
671 Clostridium saccharoperbutylacetonicum N1-4_28HMT_29 GCA_000340885.1
655 Clostridium saccharobutylicum DSM 13864 GCA_000473995.1
xS
938 Bifidobacterium adolescentis ATCC 15703 GCA_000010425.1
800 Bifidobacterium angulatum DSM 20098 = JCM 7096 GCA_001025155.1
782 Bifidobacterium dentium JCM 1195 = DSM 20436 GCA_001042595.1
xT
800 Staphylococcus epidermidis ATCC 12228 GCA_000007645.1
737 Staphylococcus capitis subsp. capitis GCA_001028645.1
723 Staphylococcus warneri SG1 GCA_000332735.1

xU
837 Bacillus anthracis str. Ames GCA_000007845.1
837 Bacillus anthracis str. Sterne GCA_000008165.1
837 _5BBacillus thuringiensis_5D serovar konkukian str. 97-27 GCA_000008505.1
812 Bacillus cereus ATCC 14579 GCA_000007825.1
797 Bacillus pseudomycoides DSM 12442 GCA_000161455.1
xV
730 Staphylococcus epidermidis ATCC 12228 GCA_000007645.1
682 Staphylococcus capitis subsp. capitis GCA_001028645.1
634 Staphylococcus hominis subsp. hominis C80 GCA_000183685.1
xW
533 Clostridium beijerinckii GCA_000833105.2
508 Clostridium puniceum GCA_002006345.1
508 Clostridium saccharoperbutylacetonicum N1-4_28HMT_29 GCA_000340885.1
495 Clostridium butyricum GCA_001456065.2
xX
690 Clostridium beijerinckii GCA_000833105.2
644 Clostridium saccharoperbutylacetonicum N1-4_28HMT_29 GCA_000340885.1
632 Clostridium butyricum GCA_001456065.2
xY
703 Rhodobacter sphaeroides 2.4.1 GCA_000012905.2
643 Rhodobacter sphaeroides ATCC 17025 GCA_000016405.1
571 Gemmobacter aquatilis GCA_900110025.1
xZ
737 Clostridium beijerinckii GCA_000833105.2
729 Clostridium saccharobutylicum DSM 13864 GCA_000473995.1
726 Clostridium saccharoperbutylacetonicum N1-4_28HMT_29 GCA_000340885.1
y0
400 Rhodobacter sphaeroides 2.4.1 GCA_000012905.2
346 Rhodobacter sphaeroides ATCC 17025 GCA_000016405.1
337 Defluviimonas alba GCA_001620265.1
y1
460 Enterococcus faecalis V583 GCA_000007785.1
413 Streptomyces cinnamoneus GCA_001885705.1
392 Enterococcus haemoperoxidus ATCC BAA-382 GCA_000407165.1
y2
805 Clostridium beijerinckii GCA_000833105.2
799 Clostridium saccharoperbutylacetonicum N1-4_28HMT_29 GCA_000340885.1
743 Clostridium puniceum GCA_002006345.1
y3
679 Staphylococcus epidermidis ATCC 12228 GCA_000007645.1
640 Staphylococcus capitis subsp. capitis GCA_001028645.1
627 Megasphaera cerevisiae DSM 20462 GCA_001045675.1
627 Staphylococcus warneri SG1 GCA_000332735.1
y4
895 Lactobacillus gasseri ATCC 33323 = JCM 1131 GCA_000014425.1
818 Lactobacillus hominis DSM 23910 = CRBIP 24.179 GCA_000296835.1
558 Lactobacillus iners DSM 13335 GCA_000160875.1
y5
234 Staphylococcus epidermidis ATCC 12228 GCA_000007645.1
226 Staphylococcus capitis subsp. capitis GCA_001028645.1
214 Megasphaera cerevisiae DSM 20462 GCA_001045675.1
214 Staphylococcus aureus subsp. aureus NCTC 8325 GCA_000013425.1
214 Staphylococcus warneri SG1 GCA_000332735.1
y6
574 Clostridium beijerinckii GCA_000833105.2
527 Clostridium puniceum GCA_002006345.1
525 Clostridium saccharoperbutylacetonicum N1-4_28HMT_29 GCA_000340885.1
y7
564 Rhodobacter sphaeroides 2.4.1 GCA_000012905.2
518 Rhodobacter sphaeroides ATCC 17025 GCA_000016405.1
445 Defluviimonas alba GCA_001620265.1
y8
427 Rhodobacter sphaeroides 2.4.1 GCA_000012905.2
389 Rhodobacter sphaeroides ATCC 17025 GCA_000016405.1
335 Gemmobacter aquatilis GCA_900110025.1
y9
672 Lactobacillus gasseri ATCC 33323 = JCM 1131 GCA_000014425.1
651 Lactobacillus hominis DSM 23910 = CRBIP 24.179 GCA_000296835.1
497 Lactobacillus iners DSM 13335 GCA_000160875.1
ya
747 Enterococcus faecalis V583 GCA_000007785.1
701 Streptomyces cinnamoneus GCA_001885705.1
627 Enterococcus hirae ATCC 9790 GCA_000271405.2
yb
670 Rhodobacter sphaeroides 2.4.1 GCA_000012905.2

609 Rhodobacter sphaeroides ATCC 17025 GCA_000016405.1
487 Pseudorhodobacter psychrotolerans GCA_001294535.1
yc
411 Clostridium beijerinckii GCA_000833105.2
402 Clostridium saccharoperbutylacetonicum N1-4_28HMT_29 GCA_000340885.1
388 Clostridium puniceum GCA_002006345.1
yd
642 Clostridium beijerinckii GCA_000833105.2
619 Clostridium saccharoperbutylacetonicum N1-4_28HMT_29 GCA_000340885.1
600 Clostridium puniceum GCA_002006345.1
ye
654 Lactobacillus gasseri ATCC 33323 = JCM 1131 GCA_000014425.1
601 Lactobacillus hominis DSM 23910 = CRBIP 24.179 GCA_000296835.1
411 Lactobacillus psittaci DSM 15354 GCA_000425905.1
yf
923 Staphylococcus epidermidis ATCC 12228 GCA_000007645.1
864 Staphylococcus capitis subsp. capitis GCA_001028645.1
821 Staphylococcus hominis subsp. hominis C80 GCA_000183685.1
yg
784 Enterococcus faecalis V583 GCA_000007785.1
737 Streptomyces cinnamoneus GCA_001885705.1
656 Enterococcus haemoperoxidus ATCC BAA-382 GCA_000407165.1
656 Enterococcus rivorum GCA_001742285.1
yh
755 Streptococcus mutans UA159 GCA_000007465.2
593 Streptococcus gordonii str. Challis substr. CH1 GCA_000017005.1
575 Streptococcus ratti FA-1 = DSM 20564 GCA_000286075.1
yi
752 Clostridium beijerinckii GCA_000833105.2
748 Clostridium saccharoperbutylacetonicum N1-4_28HMT_29 GCA_000340885.1
727 Clostridium puniceum GCA_002006345.1
727 Clostridium saccharobutylicum DSM 13864 GCA_000473995.1
yj
465 Deinococcus radiodurans R1 GCA_000008565.1
280 Deinococcus marmoris DSM 12784 GCA_000701405.1
271 Deinococcus deserti VCD115 GCA_000020685.1
271 Deinococcus hopiensis KR-140 GCA_900176165.1
yk
788 Lactobacillus gasseri ATCC 33323 = JCM 1131 GCA_000014425.1
721 Lactobacillus hominis DSM 23910 = CRBIP 24.179 GCA_000296835.1
569 Lactobacillus iners DSM 13335 GCA_000160875.1
yl
651 Lactobacillus gasseri ATCC 33323 = JCM 1131 GCA_000014425.1
562 Lactobacillus hominis DSM 23910 = CRBIP 24.179 GCA_000296835.1
437 Lactobacillus iners DSM 13335 GCA_000160875.1
ym
839 Streptococcus mutans UA159 GCA_000007465.2
557 Streptococcus halotolerans GCA_001598035.1
542 Streptococcus gordonii str. Challis substr. CH1 GCA_000017005.1
yn
466 Escherichia coli O157_3AH7 str. Sakai GCA_000008865.1
466 Escherichia coli UMN026 GCA_000026325.2
466 Escherichia coli str. K-12 substr. MG1655 GCA_000005845.2
442 Shigella flexneri 2a str. 301 GCA_000006925.2
439 Escherichia coli O83_3AH1 str. NRG 857C GCA_000183345.1
yo
512 Bifidobacterium adolescentis ATCC 15703 GCA_000010425.1
421 Bifidobacterium angulatum DSM 20098 = JCM 7096 GCA_001025155.1
411 Bifidobacterium dentium JCM 1195 = DSM 20436 GCA_001042595.1
yp
869 Clostridium beijerinckii GCA_000833105.2
817 Clostridium saccharoperbutylacetonicum N1-4_28HMT_29 GCA_000340885.1
780 Clostridium saccharobutylicum DSM 13864 GCA_000473995.1
yq
714 Staphylococcus epidermidis ATCC 12228 GCA_000007645.1
662 Staphylococcus capitis subsp. capitis GCA_001028645.1
630 Staphylococcus hominis subsp. hominis C80 GCA_000183685.1
yr
723 Clostridium beijerinckii GCA_000833105.2
698 Clostridium saccharoperbutylacetonicum N1-4_28HMT_29 GCA_000340885.1
692 Clostridium puniceum GCA_002006345.1
ys
730 Rhodobacter sphaeroides 2.4.1 GCA_000012905.2
685 Rhodobacter sphaeroides ATCC 17025 GCA_000016405.1
558 Pseudorhodobacter psychrotolerans GCA_001294535.1
yt

637 Staphylococcus epidermidis ATCC 12228 GCA_000007645.1
610 Staphylococcus capitis subsp. capitis GCA_001028645.1
587 Staphylococcus aureus subsp. aureus NCTC 8325 GCA_000013425.1
yu
640 Streptococcus mutans UA159 GCA_000007465.2
447 Streptococcus ratti FA-1 = DSM 20564 GCA_000286075.1
423 Streptococcus ovis DSM 16829 GCA_000380125.1
yv
542 Deinococcus radiodurans R1 GCA_000008565.1
308 Deinococcus deserti VCD115 GCA_000020685.1
303 Deinococcus soli Cha et al. 2016 GCA_001007995.1
yw
625 Clostridium beijerinckii GCA_000833105.2
578 Clostridium saccharoperbutylacetonicum N1-4_28HMT_29 GCA_000340885.1
571 Clostridium puniceum GCA_002006345.1
yx
574 Shigella flexneri 2a str. 301 GCA_000006925.2
573 Escherichia coli O104_3AH4 str. 2011C-3493 GCA_000299455.1
573 Escherichia coli str. K-12 substr. MG1655 GCA_000005845.2
548 Escherichia coli UMN026 GCA_000026325.2
yy
724 Bacillus anthracis str. Ames GCA_000007845.1
724 Bacillus anthracis str. Sterne GCA_000008165.1
724 _5BBacillus thuringiensis_5D serovar konkukian str. 97-27 GCA_000008505.1
715 Bacillus cereus ATCC 14579 GCA_000007825.1
703 Bacillus pseudomycoides DSM 12442 GCA_000161455.1
yz
742 Staphylococcus epidermidis ATCC 12228 GCA_000007645.1
689 Staphylococcus capitis subsp. capitis GCA_001028645.1
625 Staphylococcus hominis subsp. hominis C80 GCA_000183685.1
yA
533 Staphylococcus epidermidis ATCC 12228 GCA_000007645.1
516 Staphylococcus haemolyticus JCSC1435 GCA_000009865.1
508 Staphylococcus capitis subsp. capitis GCA_001028645.1
yB
495 Rhodobacter sphaeroides 2.4.1 GCA_000012905.2
431 Rhodobacter sphaeroides ATCC 17025 GCA_000016405.1
389 Gemmobacter megaterium GCA_900156815.1
yC
717 Clostridium beijerinckii GCA_000833105.2
701 Clostridium saccharoperbutylacetonicum N1-4_28HMT_29 GCA_000340885.1
692 Clostridium saccharobutylicum DSM 13864 GCA_000473995.1
yD
608 Streptococcus mutans UA159 GCA_000007465.2
428 Streptococcus ratti FA-1 = DSM 20564 GCA_000286075.1
399 Streptococcus equinus GCA_000964315.1
399 Streptococcus gallolyticus subsp. gallolyticus DSM 16831 GCA_002000985.1
yE
905 Staphylococcus epidermidis ATCC 12228 GCA_000007645.1
861 Staphylococcus capitis subsp. capitis GCA_001028645.1
778 Staphylococcus warneri SG1 GCA_000332735.1
yF
742 Deinococcus radiodurans R1 GCA_000008565.1
489 Deinococcus deserti VCD115 GCA_000020685.1
487 Deinococcus gobiensis I-0 GCA_000252445.1
yG
652 Streptococcus mutans UA159 GCA_000007465.2
492 Streptococcus halotolerans GCA_001598035.1
487 Streptococcus ratti FA-1 = DSM 20564 GCA_000286075.1
yH
592 Streptococcus mutans UA159 GCA_000007465.2
471 Streptococcus ratti FA-1 = DSM 20564 GCA_000286075.1
453 Streptococcus sobrinus DSM 20742 = ATCC 33478 GCA_000686605.1
yI
414 Escherichia coli str. K-12 substr. MG1655 GCA_000005845.2
399 Escherichia coli IAI39 GCA_000026345.1
391 Escherichia coli UMN026 GCA_000026325.2
yJ
671 Clostridium beijerinckii GCA_000833105.2
668 Clostridium saccharoperbutylacetonicum N1-4_28HMT_29 GCA_000340885.1
632 Clostridium puniceum GCA_002006345.1
yK
554 Clostridium beijerinckii GCA_000833105.2
497 Clostridium saccharoperbutylacetonicum N1-4_28HMT_29 GCA_000340885.1
470 Clostridium saccharobutylicum DSM 13864 GCA_000473995.1
yL

842 Clostridium beijerinckii GCA_000833105.2
801 Clostridium saccharoperbutylacetonicum N1-4_28HMT_29 GCA_000340885.1
760 Clostridium saccharobutylicum DSM 13864 GCA_000473995.1
yM
573 Clostridium beijerinckii GCA_000833105.2
559 Clostridium saccharoperbutylacetonicum N1-4_28HMT_29 GCA_000340885.1
555 Clostridium saccharobutylicum DSM 13864 GCA_000473995.1
yN
468 Clostridium beijerinckii GCA_000833105.2
444 Clostridium saccharoperbutylacetonicum N1-4_28HMT_29 GCA_000340885.1
435 Clostridium butyricum GCA_001456065.2
yO
550 Escherichia coli O104_3AH4 str. 2011C-3493 GCA_000299455.1
535 Escherichia coli str. K-12 substr. MG1655 GCA_000005845.2
535 Shigella flexneri 2a str. 301 GCA_000006925.2
520 Escherichia coli O157_3AH7 str. Sakai GCA_000008865.1
520 Escherichia coli O83_3AH1 str. NRG 857C GCA_000183345.1
520 Escherichia coli UMN026 GCA_000026325.2
yP
404 Deinococcus radiodurans R1 GCA_000008565.1
252 Deinococcus gobiensis I-0 GCA_000252445.1
251 Deinococcus proteolyticus MRP GCA_000190555.1
yQ
657 Streptococcus mutans UA159 GCA_000007465.2
454 Streptococcus gordonii str. Challis substr. CH1 GCA_000017005.1
450 Streptococcus ratti FA-1 = DSM 20564 GCA_000286075.1
yR
733 Deinococcus radiodurans R1 GCA_000008565.1
488 Deinococcus deserti VCD115 GCA_000020685.1
479 Deinococcus puniceus GCA_001644565.1
yS
819 Bacillus cereus ATCC 14579 GCA_000007825.1
819 _5BBacillus thuringiensis_5D serovar konkukian str. 97-27 GCA_000008505.1
818 Bacillus anthracis str. Ames GCA_000007845.1
818 Bacillus anthracis str. Sterne GCA_000008165.1
803 Bacillus thuringiensis YBT-1518 GCA_000497525.2
yT
667 Streptococcus mutans UA159 GCA_000007465.2
472 Streptococcus ratti FA-1 = DSM 20564 GCA_000286075.1
426 Streptococcus ferus DSM 20646 GCA_000372425.1
yU
447 Staphylococcus epidermidis ATCC 12228 GCA_000007645.1
420 Staphylococcus capitis subsp. capitis GCA_001028645.1
419 Staphylococcus haemolyticus JCSC1435 GCA_000009865.1
yV
594 Bifidobacterium adolescentis ATCC 15703 GCA_000010425.1
503 Bifidobacterium dentium JCM 1195 = DSM 20436 GCA_001042595.1
408 Bifidobacterium stellenboschense GCA_000741785.1
yW
765 Lactobacillus gasseri ATCC 33323 = JCM 1131 GCA_000014425.1
707 Lactobacillus hominis DSM 23910 = CRBIP 24.179 GCA_000296835.1
527 Lactobacillus iners DSM 13335 GCA_000160875.1
yX
697 Lactobacillus gasseri ATCC 33323 = JCM 1131 GCA_000014425.1
625 Lactobacillus hominis DSM 23910 = CRBIP 24.179 GCA_000296835.1
474 Lactobacillus iners DSM 13335 GCA_000160875.1
yY
562 Deinococcus radiodurans R1 GCA_000008565.1
342 Deinococcus deserti VCD115 GCA_000020685.1
335 Deinococcus soli Cha et al. 2016 GCA_001007995.1
yZ
795 Rhodobacter sphaeroides 2.4.1 GCA_000012905.2
739 Rhodobacter sphaeroides ATCC 17025 GCA_000016405.1
547 Gemmobacter aquatilis GCA_900110025.1
z0
584 Deinococcus radiodurans R1 GCA_000008565.1
395 Deinococcus proteolyticus MRP GCA_000190555.1
386 Deinococcus deserti VCD115 GCA_000020685.1
z1
556 Rhodobacter sphaeroides 2.4.1 GCA_000012905.2
490 Rhodobacter sphaeroides ATCC 17025 GCA_000016405.1
443 Rhodobacter vinaykumarii GCA_900156695.1
z2
607 Staphylococcus epidermidis ATCC 12228 GCA_000007645.1
601 Staphylococcus capitis subsp. capitis GCA_001028645.1
519 Staphylococcus warneri SG1 GCA_000332735.1

z3
730 Enterococcus faecalis V583 GCA_000007785.1
683 Streptomyces cinnamoneus GCA_001885705.1
632 Enterococcus massiliensis GCA_001050095.1
632 Enterococcus rivorum GCA_001742285.1
z4
750 Staphylococcus epidermidis ATCC 12228 GCA_000007645.1
724 Staphylococcus capitis subsp. capitis GCA_001028645.1
712 Staphylococcus haemolyticus JCSC1435 GCA_000009865.1
z5
808 Deinococcus radiodurans R1 GCA_000008565.1
478 Deinococcus deserti VCD115 GCA_000020685.1
465 Deinococcus gobiensis I-0 GCA_000252445.1
z6
866 Streptococcus mutans UA159 GCA_000007465.2
590 Streptococcus ratti FA-1 = DSM 20564 GCA_000286075.1
572 Streptococcus gordonii str. Challis substr. CH1 GCA_000017005.1
z7
553 Clostridium beijerinckii GCA_000833105.2
524 Clostridium saccharoperbutylacetonicum N1-4_28HMT_29 GCA_000340885.1
521 Clostridium saccharobutylicum DSM 13864 GCA_000473995.1
z8
915 Clostridium beijerinckii GCA_000833105.2
898 Clostridium saccharoperbutylacetonicum N1-4_28HMT_29 GCA_000340885.1
878 Clostridium puniceum GCA_002006345.1
z9
674 Staphylococcus epidermidis ATCC 12228 GCA_000007645.1
668 Staphylococcus capitis subsp. capitis GCA_001028645.1
612 Staphylococcus hominis subsp. hominis C80 GCA_000183685.1
za
635 Streptococcus mutans UA159 GCA_000007465.2
444 Streptococcus ratti FA-1 = DSM 20564 GCA_000286075.1
422 Streptococcus halotolerans GCA_001598035.1
zb
625 Rhodobacter sphaeroides 2.4.1 GCA_000012905.2
547 Rhodobacter sphaeroides ATCC 17025 GCA_000016405.1
490 Pseudorhodobacter psychrotolerans GCA_001294535.1
zc
545 Enterococcus faecalis V583 GCA_000007785.1
504 Streptomyces cinnamoneus GCA_001885705.1
478 Enterococcus rivorum GCA_001742285.1
zd
488 Lactobacillus gasseri ATCC 33323 = JCM 1131 GCA_000014425.1
477 Lactobacillus hominis DSM 23910 = CRBIP 24.179 GCA_000296835.1
317 Lactobacillus hamsteri DSM 5661 = JCM 6256 GCA_000615445.1
ze
596 Clostridium beijerinckii GCA_000833105.2
586 Clostridium saccharobutylicum DSM 13864 GCA_000473995.1
571 Clostridium saccharoperbutylacetonicum N1-4_28HMT_29 GCA_000340885.1
zf
515 Rhodobacter sphaeroides 2.4.1 GCA_000012905.2
465 Rhodobacter sphaeroides ATCC 17025 GCA_000016405.1
418 Gemmobacter aquatilis GCA_900110025.1
zg
570 Streptococcus mutans UA159 GCA_000007465.2
421 Streptococcus ratti FA-1 = DSM 20564 GCA_000286075.1
367 Streptococcus gallolyticus subsp. gallolyticus DSM 16831 GCA_002000985.1
367 Streptococcus henryi DSM 19005 GCA_000376985.1
zh
725 Rhodobacter sphaeroides 2.4.1 GCA_000012905.2
664 Rhodobacter sphaeroides ATCC 17025 GCA_000016405.1
593 Gemmobacter aquatilis GCA_900110025.1
zi
782 Clostridium beijerinckii GCA_000833105.2
772 Clostridium saccharoperbutylacetonicum N1-4_28HMT_29 GCA_000340885.1
760 Clostridium puniceum GCA_002006345.1
zj
527 Rhodobacter sphaeroides 2.4.1 GCA_000012905.2
451 Rhodobacter sphaeroides ATCC 17025 GCA_000016405.1
397 Rhodobacter capsulatus SB 1003 GCA_000021865.1
zk
815 Clostridium beijerinckii GCA_000833105.2
776 Clostridium saccharoperbutylacetonicum N1-4_28HMT_29 GCA_000340885.1
766 Clostridium puniceum GCA_002006345.1
zl
724 Deinococcus radiodurans R1 GCA_000008565.1

428 Deinococcus deserti VCD115 GCA_000020685.1
417 Deinococcus soli Cha et al. 2016 GCA_001007995.1
zm
791 Streptococcus mutans UA159 GCA_000007465.2
581 Streptococcus ratti FA-1 = DSM 20564 GCA_000286075.1
524 Streptococcus gordonii str. Challis substr. CH1 GCA_000017005.1
zn
776 Escherichia coli O157_3AH7 str. Sakai GCA_000008865.1
776 Escherichia coli UMN026 GCA_000026325.2
776 Escherichia coli str. K-12 substr. MG1655 GCA_000005845.2
748 Shigella flexneri 2a str. 301 GCA_000006925.2
743 Escherichia coli O83_3AH1 str. NRG 857C GCA_000183345.1
zo
586 Clostridium beijerinckii GCA_000833105.2
557 Clostridium saccharoperbutylacetonicum N1-4_28HMT_29 GCA_000340885.1
523 Clostridium puniceum GCA_002006345.1
zp
935 Rhodobacter sphaeroides 2.4.1 GCA_000012905.2
822 Rhodobacter sphaeroides ATCC 17025 GCA_000016405.1
717 Gemmobacter megaterium GCA_900156815.1
zq
748 Bifidobacterium adolescentis ATCC 15703 GCA_000010425.1
618 Bifidobacterium dentium JCM 1195 = DSM 20436 GCA_001042595.1
598 Bifidobacterium angulatum DSM 20098 = JCM 7096 GCA_001025155.1
zr
628 Enterococcus faecalis V583 GCA_000007785.1
581 Streptomyces cinnamoneus GCA_001885705.1
576 Enterococcus thailandicus GCA_001652875.1
zs
632 Rhodobacter sphaeroides 2.4.1 GCA_000012905.2
542 Rhodobacter sphaeroides ATCC 17025 GCA_000016405.1
400 Gemmobacter megaterium GCA_900156815.1
zt
825 Lactobacillus gasseri ATCC 33323 = JCM 1131 GCA_000014425.1
784 Lactobacillus hominis DSM 23910 = CRBIP 24.179 GCA_000296835.1
521 Lactobacillus helveticus GCA_001308285.1
zu
548 Staphylococcus epidermidis ATCC 12228 GCA_000007645.1
507 Staphylococcus capitis subsp. capitis GCA_001028645.1
498 Staphylococcus aureus subsp. aureus NCTC 8325 GCA_000013425.1
zv
849 Enterococcus faecalis V583 GCA_000007785.1
806 Streptomyces cinnamoneus GCA_001885705.1
718 Enterococcus rivorum GCA_001742285.1
zw
884 Clostridium beijerinckii GCA_000833105.2
836 Clostridium saccharoperbutylacetonicum N1-4_28HMT_29 GCA_000340885.1
822 Clostridium puniceum GCA_002006345.1
zx
647 Staphylococcus epidermidis ATCC 12228 GCA_000007645.1
591 Staphylococcus capitis subsp. capitis GCA_001028645.1
542 Staphylococcus lentus F1142 GCA_000286395.1
zy
717 Lactobacillus gasseri ATCC 33323 = JCM 1131 GCA_000014425.1
708 Lactobacillus hominis DSM 23910 = CRBIP 24.179 GCA_000296835.1
530 Lactobacillus psittaci DSM 15354 GCA_000425905.1
zz
632 Clostridium beijerinckii GCA_000833105.2
596 Clostridium saccharoperbutylacetonicum N1-4_28HMT_29 GCA_000340885.1
580 Clostridium saccharobutylicum DSM 13864 GCA_000473995.1
zA
544 Staphylococcus epidermidis ATCC 12228 GCA_000007645.1
529 Staphylococcus capitis subsp. capitis GCA_001028645.1
504 Staphylococcus pettenkoferi GCA_002208805.1
zB
977 Staphylococcus epidermidis ATCC 12228 GCA_000007645.1
936 Staphylococcus capitis subsp. capitis GCA_001028645.1
844 Staphylococcus warneri SG1 GCA_000332735.1
zC
777 Clostridium beijerinckii GCA_000833105.2
744 Clostridium saccharoperbutylacetonicum N1-4_28HMT_29 GCA_000340885.1
719 Clostridium puniceum GCA_002006345.1
zD
483 Rhodobacter sphaeroides 2.4.1 GCA_000012905.2
446 Rhodobacter sphaeroides ATCC 17025 GCA_000016405.1
335 Pseudorhodobacter ferrugineus DSM 5888 GCA_000420745.1

zE
534 Clostridium beijerinckii GCA_000833105.2
514 Clostridium saccharobutylicum DSM 13864 GCA_000473995.1
493 Clostridium saccharoperbutylacetonicum N1-4_28HMT_29 GCA_000340885.1
zF
928 Lactobacillus gasseri ATCC 33323 = JCM 1131 GCA_000014425.1
871 Lactobacillus hominis DSM 23910 = CRBIP 24.179 GCA_000296835.1
640 Lactobacillus iners DSM 13335 GCA_000160875.1
zG
709 Deinococcus radiodurans R1 GCA_000008565.1
483 Deinococcus deserti VCD115 GCA_000020685.1
436 Deinococcus gobiensis I-0 GCA_000252445.1
zH
357 Enterococcus faecalis V583 GCA_000007785.1
338 Streptomyces cinnamoneus GCA_001885705.1
335 Enterococcus rivorum GCA_001742285.1
zI
677 Rhodobacter sphaeroides 2.4.1 GCA_000012905.2
633 Rhodobacter sphaeroides ATCC 17025 GCA_000016405.1
513 Rhodobacter capsulatus SB 1003 GCA_000021865.1
zJ
711 Rhodobacter sphaeroides 2.4.1 GCA_000012905.2
630 Rhodobacter sphaeroides ATCC 17025 GCA_000016405.1
575 Gemmobacter aquatilis GCA_900110025.1
zK
828 Clostridium beijerinckii GCA_000833105.2
780 Clostridium saccharoperbutylacetonicum N1-4_28HMT_29 GCA_000340885.1
747 Clostridium saccharobutylicum DSM 13864 GCA_000473995.1
zL
737 Lactobacillus gasseri ATCC 33323 = JCM 1131 GCA_000014425.1
683 Lactobacillus hominis DSM 23910 = CRBIP 24.179 GCA_000296835.1
531 Lactobacillus iners DSM 13335 GCA_000160875.1
zM
884 Streptococcus mutans UA159 GCA_000007465.2
668 Streptococcus ratti FA-1 = DSM 20564 GCA_000286075.1
628 Streptococcus gordonii str. Challis substr. CH1 GCA_000017005.1
zN
579 Rhodobacter sphaeroides 2.4.1 GCA_000012905.2
517 Rhodobacter sphaeroides ATCC 17025 GCA_000016405.1
460 Gemmobacter aquatilis GCA_900110025.1
zO
826 Rhodobacter sphaeroides 2.4.1 GCA_000012905.2
720 Rhodobacter sphaeroides ATCC 17025 GCA_000016405.1
618 Gemmobacter aquatilis GCA_900110025.1
zP
642 Clostridium beijerinckii GCA_000833105.2
602 Clostridium saccharobutylicum DSM 13864 GCA_000473995.1
602 Clostridium saccharoperbutylacetonicum N1-4_28HMT_29 GCA_000340885.1
575 Clostridium butyricum GCA_001456065.2
zQ
847 Rhodobacter sphaeroides 2.4.1 GCA_000012905.2
754 Rhodobacter sphaeroides ATCC 17025 GCA_000016405.1
662 Gemmobacter aquatilis GCA_900110025.1
zR
336 Staphylococcus epidermidis ATCC 12228 GCA_000007645.1
313 Staphylococcus warneri SG1 GCA_000332735.1
303 Staphylococcus capitis subsp. capitis GCA_001028645.1
zS
726 Clostridium beijerinckii GCA_000833105.2
677 Clostridium saccharoperbutylacetonicum N1-4_28HMT_29 GCA_000340885.1
676 Clostridium saccharobutylicum DSM 13864 GCA_000473995.1
zT
637 Clostridium beijerinckii GCA_000833105.2
604 Clostridium saccharoperbutylacetonicum N1-4_28HMT_29 GCA_000340885.1
573 Clostridium puniceum GCA_002006345.1
zU
789 Clostridium beijerinckii GCA_000833105.2
752 Clostridium saccharoperbutylacetonicum N1-4_28HMT_29 GCA_000340885.1
733 Clostridium saccharobutylicum DSM 13864 GCA_000473995.1
zV
717 Staphylococcus epidermidis ATCC 12228 GCA_000007645.1
699 Staphylococcus capitis subsp. capitis GCA_001028645.1
622 Staphylococcus warneri SG1 GCA_000332735.1
zW
700 Streptococcus mutans UA159 GCA_000007465.2
516 Streptococcus ratti FA-1 = DSM 20564 GCA_000286075.1

487 Streptococcus iniae GCA_000831485.1
zX
887 Clostridium beijerinckii GCA_000833105.2
831 Clostridium saccharoperbutylacetonicum N1-4_28HMT_29 GCA_000340885.1
797 Clostridium puniceum GCA_002006345.1
zY
485 Streptococcus mutans UA159 GCA_000007465.2
409 Streptococcus ratti FA-1 = DSM 20564 GCA_000286075.1
377 Streptococcus halotolerans GCA_001598035.1
377 Streptococcus sobrinus DSM 20742 = ATCC 33478 GCA_000686605.1
zZ
588 Rhodobacter sphaeroides 2.4.1 GCA_000012905.2
554 Rhodobacter sphaeroides ATCC 17025 GCA_000016405.1
440 Gemmobacter aquatilis GCA_900110025.1
440 Pseudorhodobacter psychrotolerans GCA_001294535.1
A0
801 Deinococcus radiodurans R1 GCA_000008565.1
573 Deinococcus deserti VCD115 GCA_000020685.1
529 Deinococcus gobiensis I-0 GCA_000252445.1
A1
783 Lactobacillus gasseri ATCC 33323 = JCM 1131 GCA_000014425.1
719 Lactobacillus hominis DSM 23910 = CRBIP 24.179 GCA_000296835.1
609 Lactobacillus psittaci DSM 15354 GCA_000425905.1
A2
752 Streptococcus mutans UA159 GCA_000007465.2
522 Streptococcus gordonii str. Challis substr. CH1 GCA_000017005.1
518 Streptococcus sanguinis SK36 GCA_000014205.1
A3
803 Enterococcus faecalis V583 GCA_000007785.1
756 Streptomyces cinnamoneus GCA_001885705.1
719 Enterococcus rivorum GCA_001742285.1
A4
540 Rhodobacter sphaeroides 2.4.1 GCA_000012905.2
503 Rhodobacter sphaeroides ATCC 17025 GCA_000016405.1
445 Pseudorhodobacter wandonensis GCA_001202035.1
A5
842 Bacillus anthracis str. Ames GCA_000007845.1
842 Bacillus anthracis str. Sterne GCA_000008165.1
842 Bacillus cereus ATCC 14579 GCA_000007825.1
842 _5BBacillus thuringiensis_5D serovar konkukian str. 97-27 GCA_000008505.1
817 Bacillus thuringiensis YBT-1518 GCA_000497525.2
802 Bacillus pseudomycoides DSM 12442 GCA_000161455.1
A6
675 Rhodobacter sphaeroides 2.4.1 GCA_000012905.2
624 Rhodobacter sphaeroides ATCC 17025 GCA_000016405.1
513 Gemmobacter aquatilis GCA_900110025.1
A7
809 Enterococcus faecalis V583 GCA_000007785.1
762 Streptomyces cinnamoneus GCA_001885705.1
716 Enterococcus rivorum GCA_001742285.1
A8
754 Staphylococcus epidermidis ATCC 12228 GCA_000007645.1
681 Staphylococcus capitis subsp. capitis GCA_001028645.1
630 Staphylococcus lugdunensis HKU09-01 GCA_000025085.1
A9
774 Staphylococcus epidermidis ATCC 12228 GCA_000007645.1
715 Staphylococcus capitis subsp. capitis GCA_001028645.1
672 Staphylococcus hominis subsp. hominis C80 GCA_000183685.1
Aa
764 Streptococcus mutans UA159 GCA_000007465.2
584 Streptococcus ratti FA-1 = DSM 20564 GCA_000286075.1
567 Streptococcus gordonii str. Challis substr. CH1 GCA_000017005.1
Ab
910 Lactobacillus gasseri ATCC 33323 = JCM 1131 GCA_000014425.1
810 Lactobacillus hominis DSM 23910 = CRBIP 24.179 GCA_000296835.1
635 Lactobacillus iners DSM 13335 GCA_000160875.1
Ac
753 Lactobacillus gasseri ATCC 33323 = JCM 1131 GCA_000014425.1
687 Lactobacillus hominis DSM 23910 = CRBIP 24.179 GCA_000296835.1
585 Lactobacillus jensenii GCA_001936235.1
Ad
419 Staphylococcus epidermidis ATCC 12228 GCA_000007645.1
405 Staphylococcus capitis subsp. capitis GCA_001028645.1
401 Staphylococcus aureus subsp. aureus NCTC 8325 GCA_000013425.1
Ae
890 Bacillus anthracis str. Ames GCA_000007845.1

890 Bacillus anthracis str. Sterne GCA_000008165.1
890 _5BBacillus thuringiensis_5D serovar konkukian str. 97-27 GCA_000008505.1
879 Bacillus pseudomycoides DSM 12442 GCA_000161455.1
874 Bacillus cereus ATCC 14579 GCA_000007825.1
Af
740 Enterococcus faecalis V583 GCA_000007785.1
693 Streptomyces cinnamoneus GCA_001885705.1
643 Enterococcus rivorum GCA_001742285.1
Ag
683 Lactobacillus gasseri ATCC 33323 = JCM 1131 GCA_000014425.1
657 Lactobacillus hominis DSM 23910 = CRBIP 24.179 GCA_000296835.1
503 Lactobacillus iners DSM 13335 GCA_000160875.1
503 Lactobacillus jensenii GCA_001936235.1
503 Lactobacillus psittaci DSM 15354 GCA_000425905.1
Ah
619 Deinococcus radiodurans R1 GCA_000008565.1
388 Deinococcus deserti VCD115 GCA_000020685.1
350 Deinococcus hopiensis KR-140 GCA_900176165.1
Ai
792 Streptococcus mutans UA159 GCA_000007465.2
595 Streptococcus ratti FA-1 = DSM 20564 GCA_000286075.1
561 Streptococcus criceti HS-6 GCA_000187975.3
Aj
727 Deinococcus radiodurans R1 GCA_000008565.1
389 Deinococcus gobiensis I-0 GCA_000252445.1
359 Deinococcus proteolyticus MRP GCA_000190555.1
Ak
680 Streptococcus mutans UA159 GCA_000007465.2
474 Streptococcus ratti FA-1 = DSM 20564 GCA_000286075.1
451 Streptococcus pyogenes M1 GAS GCA_000006785.2
Al
738 Deinococcus radiodurans R1 GCA_000008565.1
468 Deinococcus deserti VCD115 GCA_000020685.1
429 Deinococcus soli Cha et al. 2016 GCA_001007995.1
Am
699 Streptococcus mutans UA159 GCA_000007465.2
464 Streptococcus ferus DSM 20646 GCA_000372425.1
456 Streptococcus ratti FA-1 = DSM 20564 GCA_000286075.1
An
623 Escherichia coli O157_3AH7 str. Sakai GCA_000008865.1
623 Escherichia coli str. K-12 substr. MG1655 GCA_000005845.2
623 Shigella flexneri 2a str. 301 GCA_000006925.2
615 Escherichia coli O83_3AH1 str. NRG 857C GCA_000183345.1
615 Escherichia coli UMN026 GCA_000026325.2
609 Escherichia coli O104_3AH4 str. 2011C-3493 GCA_000299455.1
Ao
651 Escherichia coli str. K-12 substr. MG1655 GCA_000005845.2
646 Escherichia coli O157_3AH7 str. Sakai GCA_000008865.1
646 Shigella flexneri 2a str. 301 GCA_000006925.2
643 Escherichia coli O83_3AH1 str. NRG 857C GCA_000183345.1
643 Escherichia coli UMN026 GCA_000026325.2
Ap
440 Enterococcus faecalis V583 GCA_000007785.1
407 Enterococcus hirae ATCC 9790 GCA_000271405.2
393 Enterococcus faecium DO GCA_000174395.2
393 Streptomyces cinnamoneus GCA_001885705.1
Aq
551 Rhodobacter sphaeroides 2.4.1 GCA_000012905.2
493 Rhodobacter sphaeroides ATCC 17025 GCA_000016405.1
458 Pseudorhodobacter ferrugineus DSM 5888 GCA_000420745.1
Ar
757 Staphylococcus epidermidis ATCC 12228 GCA_000007645.1
751 Staphylococcus capitis subsp. capitis GCA_001028645.1
719 Staphylococcus aureus subsp. aureus NCTC 8325 GCA_000013425.1
As
979 Staphylococcus epidermidis ATCC 12228 GCA_000007645.1
903 Staphylococcus capitis subsp. capitis GCA_001028645.1
830 Staphylococcus haemolyticus JCSC1435 GCA_000009865.1
At
692 Lactobacillus gasseri ATCC 33323 = JCM 1131 GCA_000014425.1
599 Lactobacillus hominis DSM 23910 = CRBIP 24.179 GCA_000296835.1
345 Lactobacillus amylovorus GCA_000191545.1
Au
699 Deinococcus radiodurans R1 GCA_000008565.1
381 Deinococcus deserti VCD115 GCA_000020685.1
377 Deinococcus soli Cha et al. 2016 GCA_001007995.1

Av
822 Lactobacillus gasseri ATCC 33323 = JCM 1131 GCA_000014425.1
733 Lactobacillus hominis DSM 23910 = CRBIP 24.179 GCA_000296835.1
579 Lactobacillus hamsteri DSM 5661 = JCM 6256 GCA_000615445.1
Aw
587 Streptococcus mutans UA159 GCA_000007465.2
439 Streptococcus ratti FA-1 = DSM 20564 GCA_000286075.1
395 Streptococcus sobrinus DSM 20742 = ATCC 33478 GCA_000686605.1
Ax
513 Staphylococcus epidermidis ATCC 12228 GCA_000007645.1
491 Staphylococcus capitis subsp. capitis GCA_001028645.1
453 Staphylococcus warneri SG1 GCA_000332735.1
Ay
695 Escherichia coli O104_3AH4 str. 2011C-3493 GCA_000299455.1
687 Escherichia coli str. K-12 substr. MG1655 GCA_000005845.2
686 Shigella flexneri 2a str. 301 GCA_000006925.2
Az
782 Escherichia coli O157_3AH7 str. Sakai GCA_000008865.1
782 Escherichia coli str. K-12 substr. MG1655 GCA_000005845.2
781 Escherichia coli UMN026 GCA_000026325.2
767 Escherichia coli O104_3AH4 str. 2011C-3493 GCA_000299455.1
767 Shigella flexneri 2a str. 301 GCA_000006925.2
AA
399 Lactobacillus gasseri ATCC 33323 = JCM 1131 GCA_000014425.1
387 Lactobacillus hominis DSM 23910 = CRBIP 24.179 GCA_000296835.1
305 Lactobacillus psittaci DSM 15354 GCA_000425905.1
AB
814 Staphylococcus epidermidis ATCC 12228 GCA_000007645.1
786 Staphylococcus capitis subsp. capitis GCA_001028645.1
709 Staphylococcus hominis subsp. hominis C80 GCA_000183685.1
AC
704 Deinococcus radiodurans R1 GCA_000008565.1
479 Deinococcus deserti VCD115 GCA_000020685.1
464 Deinococcus marmoris DSM 12784 GCA_000701405.1
AD
403 Deinococcus radiodurans R1 GCA_000008565.1
294 Deinococcus deserti VCD115 GCA_000020685.1
264 Deinococcus hopiensis KR-140 GCA_900176165.1
AE
688 Clostridium beijerinckii GCA_000833105.2
658 Clostridium saccharoperbutylacetonicum N1-4_28HMT_29 GCA_000340885.1
626 Clostridium puniceum GCA_002006345.1
AF
868 Clostridium beijerinckii GCA_000833105.2
824 Clostridium saccharoperbutylacetonicum N1-4_28HMT_29 GCA_000340885.1
818 Clostridium butyricum GCA_001456065.2
AG
948 Lactobacillus gasseri ATCC 33323 = JCM 1131 GCA_000014425.1
874 Lactobacillus hominis DSM 23910 = CRBIP 24.179 GCA_000296835.1
687 Lactobacillus iners DSM 13335 GCA_000160875.1
AH
570 Lactobacillus gasseri ATCC 33323 = JCM 1131 GCA_000014425.1
516 Lactobacillus hominis DSM 23910 = CRBIP 24.179 GCA_000296835.1
377 Lactobacillus iners DSM 13335 GCA_000160875.1
AI
733 Escherichia coli O104_3AH4 str. 2011C-3493 GCA_000299455.1
720 Escherichia coli str. K-12 substr. MG1655 GCA_000005845.2
715 Shigella flexneri 2a str. 301 GCA_000006925.2
AJ
782 Rhodobacter sphaeroides 2.4.1 GCA_000012905.2
713 Rhodobacter sphaeroides ATCC 17025 GCA_000016405.1
638 Gemmobacter aquatilis GCA_900110025.1
AK
424 Streptococcus mutans UA159 GCA_000007465.2
297 Streptococcus ratti FA-1 = DSM 20564 GCA_000286075.1
287 Streptococcus sobrinus DSM 20742 = ATCC 33478 GCA_000686605.1
AL
431 Enterococcus faecalis V583 GCA_000007785.1
384 Streptomyces cinnamoneus GCA_001885705.1
353 Enterococcus rivorum GCA_001742285.1
AM
514 Streptococcus mutans UA159 GCA_000007465.2
346 Streptococcus ratti FA-1 = DSM 20564 GCA_000286075.1
333 Streptococcus gordonii str. Challis substr. CH1 GCA_000017005.1
AN
636 Streptococcus mutans UA159 GCA_000007465.2

425 Streptococcus gordonii str. Challis substr. CH1 GCA_000017005.1
417 Streptococcus ratti FA-1 = DSM 20564 GCA_000286075.1
AO
903 Escherichia coli O104_3AH4 str. 2011C-3493 GCA_000299455.1
903 Escherichia coli str. K-12 substr. MG1655 GCA_000005845.2
895 Shigella flexneri 2a str. 301 GCA_000006925.2
857 Escherichia coli IAI39 GCA_000026345.1
AP
533 Lactobacillus gasseri ATCC 33323 = JCM 1131 GCA_000014425.1
496 Lactobacillus hominis DSM 23910 = CRBIP 24.179 GCA_000296835.1
355 Lactobacillus kalixensis DSM 16043 GCA_001434335.1
AQ
702 Rhodobacter sphaeroides 2.4.1 GCA_000012905.2
648 Rhodobacter sphaeroides ATCC 17025 GCA_000016405.1
541 Pseudorhodobacter ferrugineus DSM 5888 GCA_000420745.1
AR
782 Clostridium beijerinckii GCA_000833105.2
748 Clostridium saccharoperbutylacetonicum N1-4_28HMT_29 GCA_000340885.1
707 Clostridium puniceum GCA_002006345.1
AS
706 Staphylococcus epidermidis ATCC 12228 GCA_000007645.1
670 Staphylococcus capitis subsp. capitis GCA_001028645.1
660 Staphylococcus aureus subsp. aureus NCTC 8325 GCA_000013425.1
AT
751 Staphylococcus epidermidis ATCC 12228 GCA_000007645.1
714 Staphylococcus capitis subsp. capitis GCA_001028645.1
638 Staphylococcus hominis subsp. hominis C80 GCA_000183685.1
AU
795 Staphylococcus epidermidis ATCC 12228 GCA_000007645.1
756 Staphylococcus capitis subsp. capitis GCA_001028645.1
730 Staphylococcus hominis subsp. hominis C80 GCA_000183685.1
AV
570 Rhodobacter sphaeroides 2.4.1 GCA_000012905.2
528 Rhodobacter sphaeroides ATCC 17025 GCA_000016405.1
431 Pseudorhodobacter wandonensis GCA_001202035.1
AW
769 Deinococcus radiodurans R1 GCA_000008565.1
486 Deinococcus deserti VCD115 GCA_000020685.1
467 Deinococcus gobiensis I-0 GCA_000252445.1
AX
864 Rhodobacter sphaeroides 2.4.1 GCA_000012905.2
770 Rhodobacter sphaeroides ATCC 17025 GCA_000016405.1
685 Defluviimonas alba GCA_001620265.1
AY
743 Streptococcus mutans UA159 GCA_000007465.2
547 Streptococcus ratti FA-1 = DSM 20564 GCA_000286075.1
515 Streptococcus gordonii str. Challis substr. CH1 GCA_000017005.1
AZ
512 Clostridium beijerinckii GCA_000833105.2
508 Clostridium saccharobutylicum DSM 13864 GCA_000473995.1
494 Clostridium saccharoperbutylacetonicum N1-4_28HMT_29 GCA_000340885.1
B0
733 Streptococcus mutans UA159 GCA_000007465.2
528 Streptococcus gordonii str. Challis substr. CH1 GCA_000017005.1
528 Streptococcus ratti FA-1 = DSM 20564 GCA_000286075.1
509 Streptococcus henryi DSM 19005 GCA_000376985.1
B1
639 Staphylococcus epidermidis ATCC 12228 GCA_000007645.1
616 Staphylococcus capitis subsp. capitis GCA_001028645.1
578 Staphylococcus aureus subsp. aureus NCTC 8325 GCA_000013425.1
B2
680 Lactobacillus gasseri ATCC 33323 = JCM 1131 GCA_000014425.1
622 Lactobacillus hominis DSM 23910 = CRBIP 24.179 GCA_000296835.1
472 Lactobacillus iners DSM 13335 GCA_000160875.1
B3
712 Rhodobacter sphaeroides 2.4.1 GCA_000012905.2
616 Rhodobacter sphaeroides ATCC 17025 GCA_000016405.1
526 Defluviimonas alba GCA_001620265.1
B4
767 Lactobacillus gasseri ATCC 33323 = JCM 1131 GCA_000014425.1
710 Lactobacillus hominis DSM 23910 = CRBIP 24.179 GCA_000296835.1
574 Lactobacillus iners DSM 13335 GCA_000160875.1
B5
611 Deinococcus radiodurans R1 GCA_000008565.1
412 Deinococcus deserti VCD115 GCA_000020685.1
390 Deinococcus gobiensis I-0 GCA_000252445.1

B6
699 Streptococcus mutans UA159 GCA_000007465.2
556 Streptococcus ratti FA-1 = DSM 20564 GCA_000286075.1
529 Streptococcus sobrinus DSM 20742 = ATCC 33478 GCA_000686605.1
B7
721 Deinococcus radiodurans R1 GCA_000008565.1
481 Deinococcus murrayi DSM 11303 GCA_000482805.1
473 Deinococcus deserti VCD115 GCA_000020685.1
B8
752 Escherichia coli str. K-12 substr. MG1655 GCA_000005845.2
748 Escherichia coli IAI39 GCA_000026345.1
748 Escherichia coli O157_3AH7 str. Sakai GCA_000008865.1
727 Escherichia coli O83_3AH1 str. NRG 857C GCA_000183345.1
B9
590 Rhodobacter sphaeroides 2.4.1 GCA_000012905.2
517 Rhodobacter sphaeroides ATCC 17025 GCA_000016405.1
459 Paracoccus solventivorans GCA_900142875.1
Ba
580 Clostridium beijerinckii GCA_000833105.2
577 Clostridium saccharoperbutylacetonicum N1-4_28HMT_29 GCA_000340885.1
573 Clostridium puniceum GCA_002006345.1
Bb
710 Enterococcus faecalis V583 GCA_000007785.1
663 Streptomyces cinnamoneus GCA_001885705.1
619 Enterococcus faecium DO GCA_000174395.2
Bc
610 Escherichia coli IAI39 GCA_000026345.1
610 Escherichia coli O157_3AH7 str. Sakai GCA_000008865.1
610 Escherichia coli str. K-12 substr. MG1655 GCA_000005845.2
610 Shigella flexneri 2a str. 301 GCA_000006925.2
609 Escherichia coli O83_3AH1 str. NRG 857C GCA_000183345.1
609 Escherichia coli UMN026 GCA_000026325.2
585 Escherichia coli O104_3AH4 str. 2011C-3493 GCA_000299455.1
Bd
631 Clostridium beijerinckii GCA_000833105.2
604 Clostridium saccharoperbutylacetonicum N1-4_28HMT_29 GCA_000340885.1
563 Clostridium puniceum GCA_002006345.1
563 Clostridium saccharobutylicum DSM 13864 GCA_000473995.1
Be
571 Rhodobacter sphaeroides 2.4.1 GCA_000012905.2
517 Rhodobacter sphaeroides ATCC 17025 GCA_000016405.1
385 Pseudorhodobacter ferrugineus DSM 5888 GCA_000420745.1
Bf
803 Staphylococcus epidermidis ATCC 12228 GCA_000007645.1
774 Staphylococcus lugdunensis HKU09-01 GCA_000025085.1
766 Staphylococcus haemolyticus JCSC1435 GCA_000009865.1
Bg
575 Enterococcus faecalis V583 GCA_000007785.1
529 Streptomyces cinnamoneus GCA_001885705.1
515 Enterococcus asini ATCC 700915 GCA_000407365.1
Bh
749 Enterococcus faecalis V583 GCA_000007785.1
705 Streptomyces cinnamoneus GCA_001885705.1
689 Enterococcus rivorum GCA_001742285.1
Bi
833 Staphylococcus epidermidis ATCC 12228 GCA_000007645.1
799 Staphylococcus capitis subsp. capitis GCA_001028645.1
744 Staphylococcus hominis subsp. hominis C80 GCA_000183685.1
Bj
802 Deinococcus radiodurans R1 GCA_000008565.1
492 Deinococcus deserti VCD115 GCA_000020685.1
463 Deinococcus hopiensis KR-140 GCA_900176165.1
Bk
789 Deinococcus radiodurans R1 GCA_000008565.1
503 Deinococcus deserti VCD115 GCA_000020685.1
475 Deinococcus hopiensis KR-140 GCA_900176165.1
Bl
616 Rhodobacter sphaeroides 2.4.1 GCA_000012905.2
571 Rhodobacter sphaeroides ATCC 17025 GCA_000016405.1
496 Pseudorhodobacter psychrotolerans GCA_001294535.1
Bm
637 Staphylococcus epidermidis ATCC 12228 GCA_000007645.1
596 Staphylococcus capitis subsp. capitis GCA_001028645.1
554 Staphylococcus simulans GCA_001559115.1
Bn
550 Deinococcus radiodurans R1 GCA_000008565.1

303 Deinococcus deserti VCD115 GCA_000020685.1
297 Deinococcus gobiensis I-0 GCA_000252445.1
Bo
694 Bacillus anthracis str. Ames GCA_000007845.1
694 Bacillus anthracis str. Sterne GCA_000008165.1
694 Bacillus cereus ATCC 14579 GCA_000007825.1
694 _5BBacillus thuringiensis_5D serovar konkukian str. 97-27 GCA_000008505.1
673 Bacillus thuringiensis YBT-1518 GCA_000497525.2
667 Bacillus pseudomycoides DSM 12442 GCA_000161455.1
Bp
674 Lactobacillus gasseri ATCC 33323 = JCM 1131 GCA_000014425.1
626 Lactobacillus hominis DSM 23910 = CRBIP 24.179 GCA_000296835.1
515 Lactobacillus iners DSM 13335 GCA_000160875.1
Bq
605 Enterococcus faecalis V583 GCA_000007785.1
558 Streptomyces cinnamoneus GCA_001885705.1
500 Enterococcus faecium DO GCA_000174395.2
500 Enterococcus rivorum GCA_001742285.1
Br
436 Bacillus pseudomycoides DSM 12442 GCA_000161455.1
434 Bacillus anthracis str. Ames GCA_000007845.1
434 Bacillus anthracis str. Sterne GCA_000008165.1
434 _5BBacillus thuringiensis_5D serovar konkukian str. 97-27 GCA_000008505.1
425 Bacillus cereus ATCC 14579 GCA_000007825.1
425 Bacillus thuringiensis YBT-1518 GCA_000497525.2
Bs
808 Staphylococcus epidermidis ATCC 12228 GCA_000007645.1
764 Staphylococcus capitis subsp. capitis GCA_001028645.1
744 Staphylococcus haemolyticus JCSC1435 GCA_000009865.1
Bt
843 Clostridium beijerinckii GCA_000833105.2
837 Clostridium saccharoperbutylacetonicum N1-4_28HMT_29 GCA_000340885.1
836 Clostridium butyricum GCA_001456065.2
Bu
595 Deinococcus radiodurans R1 GCA_000008565.1
399 Deinococcus deserti VCD115 GCA_000020685.1
398 Deinococcus soli Cha et al. 2016 GCA_001007995.1
Bv
562 Streptococcus mutans UA159 GCA_000007465.2
418 Streptococcus ratti FA-1 = DSM 20564 GCA_000286075.1
402 Streptococcus gordonii str. Challis substr. CH1 GCA_000017005.1
Bw
696 Streptococcus mutans UA159 GCA_000007465.2
520 Streptococcus ratti FA-1 = DSM 20564 GCA_000286075.1
490 Streptococcus henryi DSM 19005 GCA_000376985.1
Bx
556 Clostridium beijerinckii GCA_000833105.2
531 Clostridium saccharoperbutylacetonicum N1-4_28HMT_29 GCA_000340885.1
517 Clostridium butyricum GCA_001456065.2
By
630 Enterococcus faecalis V583 GCA_000007785.1
583 Streptomyces cinnamoneus GCA_001885705.1
500 Enterococcus rivorum GCA_001742285.1
Bz
780 Enterococcus faecalis V583 GCA_000007785.1
739 Streptomyces cinnamoneus GCA_001885705.1
653 Enterococcus rivorum GCA_001742285.1
BA
459 Rhodobacter sphaeroides 2.4.1 GCA_000012905.2
416 Rhodobacter sphaeroides ATCC 17025 GCA_000016405.1
338 Defluviimonas alba GCA_001620265.1
BB
811 Lactobacillus gasseri ATCC 33323 = JCM 1131 GCA_000014425.1
756 Lactobacillus hominis DSM 23910 = CRBIP 24.179 GCA_000296835.1
509 Lactobacillus iners DSM 13335 GCA_000160875.1
BC
531 Clostridium beijerinckii GCA_000833105.2
473 Clostridium saccharoperbutylacetonicum N1-4_28HMT_29 GCA_000340885.1
448 Clostridium butyricum GCA_001456065.2
BD
331 Clostridium beijerinckii GCA_000833105.2
272 Clostridium saccharoperbutylacetonicum N1-4_28HMT_29 GCA_000340885.1
267 Clostridium sartagoforme AAU1 GCA_000401215.1
BE
712 Rhodobacter sphaeroides 2.4.1 GCA_000012905.2
653 Rhodobacter sphaeroides ATCC 17025 GCA_000016405.1

566 Gemmobacter nectariphilus DSM 15620 GCA_000429765.1
BF
846 Rhodobacter sphaeroides 2.4.1 GCA_000012905.2
757 Rhodobacter sphaeroides ATCC 17025 GCA_000016405.1
610 Gemmobacter megaterium GCA_900156815.1
BG
731 Deinococcus radiodurans R1 GCA_000008565.1
498 Deinococcus deserti VCD115 GCA_000020685.1
484 Deinococcus gobiensis I-0 GCA_000252445.1
BH
578 Streptococcus mutans UA159 GCA_000007465.2
398 Streptococcus ratti FA-1 = DSM 20564 GCA_000286075.1
380 Streptococcus sobrinus DSM 20742 = ATCC 33478 GCA_000686605.1
BI
911 Lactobacillus gasseri ATCC 33323 = JCM 1131 GCA_000014425.1
878 Lactobacillus hominis DSM 23910 = CRBIP 24.179 GCA_000296835.1
608 Lactobacillus hamsteri DSM 5661 = JCM 6256 GCA_000615445.1
BJ
508 Clostridium beijerinckii GCA_000833105.2
464 Clostridium saccharoperbutylacetonicum N1-4_28HMT_29 GCA_000340885.1
453 Clostridium puniceum GCA_002006345.1
BK
776 Lactobacillus gasseri ATCC 33323 = JCM 1131 GCA_000014425.1
640 Lactobacillus hominis DSM 23910 = CRBIP 24.179 GCA_000296835.1
460 Lactobacillus delbrueckii subsp. bulgaricus ATCC 11842 = JCM 1002 GCA_000056065.1
BL
543 Bifidobacterium adolescentis ATCC 15703 GCA_000010425.1
419 Bifidobacterium angulatum DSM 20098 = JCM 7096 GCA_001025155.1
415 Bifidobacterium dentium JCM 1195 = DSM 20436 GCA_001042595.1
BM
732 Lactobacillus gasseri ATCC 33323 = JCM 1131 GCA_000014425.1
699 Lactobacillus hominis DSM 23910 = CRBIP 24.179 GCA_000296835.1
458 Lactobacillus psittaci DSM 15354 GCA_000425905.1
BN
662 Staphylococcus epidermidis ATCC 12228 GCA_000007645.1
654 Staphylococcus capitis subsp. capitis GCA_001028645.1
634 Staphylococcus haemolyticus JCSC1435 GCA_000009865.1
BO
809 Rhodobacter sphaeroides 2.4.1 GCA_000012905.2
751 Rhodobacter sphaeroides ATCC 17025 GCA_000016405.1
604 Defluviimonas alba GCA_001620265.1
BP
639 Clostridium beijerinckii GCA_000833105.2
585 Clostridium saccharoperbutylacetonicum N1-4_28HMT_29 GCA_000340885.1
580 Clostridium puniceum GCA_002006345.1
BQ
828 Staphylococcus epidermidis ATCC 12228 GCA_000007645.1
759 Staphylococcus capitis subsp. capitis GCA_001028645.1
745 Staphylococcus warneri SG1 GCA_000332735.1
BR
862 Lactobacillus gasseri ATCC 33323 = JCM 1131 GCA_000014425.1
808 Lactobacillus hominis DSM 23910 = CRBIP 24.179 GCA_000296835.1
547 Lactobacillus amylovorus GCA_000191545.1
BS
542 Lactobacillus gasseri ATCC 33323 = JCM 1131 GCA_000014425.1
499 Lactobacillus hominis DSM 23910 = CRBIP 24.179 GCA_000296835.1
369 Lactobacillus iners DSM 13335 GCA_000160875.1
BT
582 Clostridium beijerinckii GCA_000833105.2
548 Clostridium saccharoperbutylacetonicum N1-4_28HMT_29 GCA_000340885.1
543 Clostridium saccharobutylicum DSM 13864 GCA_000473995.1
BU
778 Deinococcus radiodurans R1 GCA_000008565.1
467 Deinococcus deserti VCD115 GCA_000020685.1
463 Deinococcus soli Cha et al. 2016 GCA_001007995.1
BV
429 Streptococcus mutans UA159 GCA_000007465.2
337 Streptococcus ratti FA-1 = DSM 20564 GCA_000286075.1
303 Streptococcus ferus DSM 20646 GCA_000372425.1
BW
741 Bifidobacterium adolescentis ATCC 15703 GCA_000010425.1
635 Bifidobacterium dentium JCM 1195 = DSM 20436 GCA_001042595.1
625 Bifidobacterium angulatum DSM 20098 = JCM 7096 GCA_001025155.1
BX
814 Clostridium beijerinckii GCA_000833105.2
802 Clostridium saccharoperbutylacetonicum N1-4_28HMT_29 GCA_000340885.1

772 Clostridium butyricum GCA_001456065.2
BY
728 Deinococcus radiodurans R1 GCA_000008565.1
466 Deinococcus gobiensis I-0 GCA_000252445.1
443 Deinococcus deserti VCD115 GCA_000020685.1
BZ
483 Bifidobacterium adolescentis ATCC 15703 GCA_000010425.1
397 Bifidobacterium angulatum DSM 20098 = JCM 7096 GCA_001025155.1
392 Bifidobacterium gallicum DSM 20093 = LMG 11596 GCA_000741205.1
C0
756 Lactobacillus gasseri ATCC 33323 = JCM 1131 GCA_000014425.1
697 Lactobacillus hominis DSM 23910 = CRBIP 24.179 GCA_000296835.1
482 Lactobacillus iners DSM 13335 GCA_000160875.1
C1
629 Streptococcus mutans UA159 GCA_000007465.2
512 Streptococcus ratti FA-1 = DSM 20564 GCA_000286075.1
467 Streptococcus criceti HS-6 GCA_000187975.3
467 Streptococcus macacae NCTC 11558 GCA_000187995.3
C2
716 Bifidobacterium adolescentis ATCC 15703 GCA_000010425.1
543 Bifidobacterium angulatum DSM 20098 = JCM 7096 GCA_001025155.1
534 Bifidobacterium dentium JCM 1195 = DSM 20436 GCA_001042595.1
C3
769 Staphylococcus epidermidis ATCC 12228 GCA_000007645.1
735 Staphylococcus capitis subsp. capitis GCA_001028645.1
683 Staphylococcus lugdunensis HKU09-01 GCA_000025085.1
C4
652 Clostridium beijerinckii GCA_000833105.2
628 Clostridium saccharoperbutylacetonicum N1-4_28HMT_29 GCA_000340885.1
613 Clostridium puniceum GCA_002006345.1
C5
586 Rhodobacter sphaeroides 2.4.1 GCA_000012905.2
552 Rhodobacter sphaeroides ATCC 17025 GCA_000016405.1
455 Gemmobacter aquatilis GCA_900110025.1
C6
502 Rhodobacter sphaeroides 2.4.1 GCA_000012905.2
419 Rhodobacter sphaeroides ATCC 17025 GCA_000016405.1
356 Gemmobacter megaterium GCA_900156815.1
C7
766 Bifidobacterium adolescentis ATCC 15703 GCA_000010425.1
642 Bifidobacterium dentium JCM 1195 = DSM 20436 GCA_001042595.1
615 Bifidobacterium angulatum DSM 20098 = JCM 7096 GCA_001025155.1
C8
592 Streptococcus mutans UA159 GCA_000007465.2
467 Clostridium beijerinckii GCA_000833105.2
464 Streptococcus gordonii str. Challis substr. CH1 GCA_000017005.1
C9
432 Clostridium beijerinckii GCA_000833105.2
421 Clostridium saccharoperbutylacetonicum N1-4_28HMT_29 GCA_000340885.1
403 Clostridium puniceum GCA_002006345.1
Ca
698 Clostridium beijerinckii GCA_000833105.2
647 Clostridium saccharobutylicum DSM 13864 GCA_000473995.1
621 Clostridium puniceum GCA_002006345.1
621 Clostridium saccharoperbutylacetonicum N1-4_28HMT_29 GCA_000340885.1
Cb
810 Streptococcus mutans UA159 GCA_000007465.2
602 Streptococcus ratti FA-1 = DSM 20564 GCA_000286075.1
543 Streptococcus equinus GCA_000964315.1
Cc
631 Clostridium beijerinckii GCA_000833105.2
550 Clostridium butyricum GCA_001456065.2
550 Clostridium saccharoperbutylacetonicum N1-4_28HMT_29 GCA_000340885.1
542 Clostridium chromiireducens GCA_002029255.1
542 Clostridium saccharobutylicum DSM 13864 GCA_000473995.1
Cd
745 Streptococcus mutans UA159 GCA_000007465.2
529 Streptococcus macacae NCTC 11558 GCA_000187995.3
525 Streptococcus ratti FA-1 = DSM 20564 GCA_000286075.1
Ce
794 Bacillus anthracis str. Ames GCA_000007845.1
794 Bacillus anthracis str. Sterne GCA_000008165.1
794 _5BBacillus thuringiensis_5D serovar konkukian str. 97-27 GCA_000008505.1
779 Bacillus cereus ATCC 14579 GCA_000007825.1
767 Bacillus thuringiensis YBT-1518 GCA_000497525.2
Cf

723 Deinococcus radiodurans R1 GCA_000008565.1
413 Deinococcus deserti VCD115 GCA_000020685.1
390 Deinococcus gobiensis I-0 GCA_000252445.1
Cg
725 Clostridium beijerinckii GCA_000833105.2
699 Clostridium saccharoperbutylacetonicum N1-4_28HMT_29 GCA_000340885.1
679 Clostridium saccharobutylicum DSM 13864 GCA_000473995.1
Ch
808 Streptococcus mutans UA159 GCA_000007465.2
574 Streptococcus gordonii str. Challis substr. CH1 GCA_000017005.1
546 Streptococcus ratti FA-1 = DSM 20564 GCA_000286075.1
Ci
767 Enterococcus faecalis V583 GCA_000007785.1
726 Streptomyces cinnamoneus GCA_001885705.1
654 Enterococcus asini ATCC 700915 GCA_000407365.1
Cj
677 Deinococcus radiodurans R1 GCA_000008565.1
439 Deinococcus gobiensis I-0 GCA_000252445.1
430 Deinococcus murrayi DSM 11303 GCA_000482805.1
Ck
820 Clostridium beijerinckii GCA_000833105.2
795 Clostridium saccharoperbutylacetonicum N1-4_28HMT_29 GCA_000340885.1
761 Clostridium saccharobutylicum DSM 13864 GCA_000473995.1
Cl
485 Rhodobacter sphaeroides 2.4.1 GCA_000012905.2
428 Rhodobacter sphaeroides ATCC 17025 GCA_000016405.1
406 Defluviimonas alba GCA_001620265.1
Cm
613 Staphylococcus epidermidis ATCC 12228 GCA_000007645.1
598 Staphylococcus capitis subsp. capitis GCA_001028645.1
563 Megasphaera cerevisiae DSM 20462 GCA_001045675.1
563 Staphylococcus warneri SG1 GCA_000332735.1
Cn
681 Rhodobacter sphaeroides 2.4.1 GCA_000012905.2
561 Rhodobacter sphaeroides ATCC 17025 GCA_000016405.1
522 Gemmobacter megaterium GCA_900156815.1
Co
742 Escherichia coli str. K-12 substr. MG1655 GCA_000005845.2
722 Escherichia coli O157_3AH7 str. Sakai GCA_000008865.1
722 Shigella flexneri 2a str. 301 GCA_000006925.2
716 Escherichia coli O104_3AH4 str. 2011C-3493 GCA_000299455.1
Cp
588 Rhodobacter sphaeroides 2.4.1 GCA_000012905.2
480 Rhodobacter sphaeroides ATCC 17025 GCA_000016405.1
467 Gemmobacter aquatilis GCA_900110025.1
Cq
345 Lactobacillus gasseri ATCC 33323 = JCM 1131 GCA_000014425.1
331 Lactobacillus hominis DSM 23910 = CRBIP 24.179 GCA_000296835.1
240 Lactobacillus iners DSM 13335 GCA_000160875.1
Cr
814 Streptococcus mutans UA159 GCA_000007465.2
646 Streptococcus ratti FA-1 = DSM 20564 GCA_000286075.1
612 Streptococcus ferus DSM 20646 GCA_000372425.1
Cs
574 Bifidobacterium adolescentis ATCC 15703 GCA_000010425.1
471 Bifidobacterium dentium JCM 1195 = DSM 20436 GCA_001042595.1
437 Bifidobacterium angulatum DSM 20098 = JCM 7096 GCA_001025155.1
Ct
620 Streptococcus mutans UA159 GCA_000007465.2
465 Streptococcus ratti FA-1 = DSM 20564 GCA_000286075.1
407 Streptococcus sanguinis SK36 GCA_000014205.1
Cu
712 _5BBacillus thuringiensis_5D serovar konkukian str. 97-27 GCA_000008505.1
711 Bacillus anthracis str. Ames GCA_000007845.1
711 Bacillus anthracis str. Sterne GCA_000008165.1
703 Bacillus cereus ATCC 14579 GCA_000007825.1
Cv
548 Clostridium beijerinckii GCA_000833105.2
515 Clostridium saccharoperbutylacetonicum N1-4_28HMT_29 GCA_000340885.1
492 Clostridium saccharobutylicum DSM 13864 GCA_000473995.1
Cw
941 Clostridium beijerinckii GCA_000833105.2
898 Clostridium saccharoperbutylacetonicum N1-4_28HMT_29 GCA_000340885.1
878 Clostridium puniceum GCA_002006345.1
Cx
665 Clostridium beijerinckii GCA_000833105.2

637 Clostridium saccharoperbutylacetonicum N1-4_28HMT_29 GCA_000340885.1
587 Clostridium puniceum GCA_002006345.1
Cy
800 Deinococcus radiodurans R1 GCA_000008565.1
508 Deinococcus deserti VCD115 GCA_000020685.1
495 Deinococcus soli Cha et al. 2016 GCA_001007995.1
Cz
474 Clostridium beijerinckii GCA_000833105.2
473 Clostridium saccharoperbutylacetonicum N1-4_28HMT_29 GCA_000340885.1
459 Clostridium saccharobutylicum DSM 13864 GCA_000473995.1
CA
698 Streptococcus mutans UA159 GCA_000007465.2
505 Streptococcus ratti FA-1 = DSM 20564 GCA_000286075.1
472 Streptococcus gordonii str. Challis substr. CH1 GCA_000017005.1
CB
518 Bifidobacterium adolescentis ATCC 15703 GCA_000010425.1
472 Bifidobacterium dentium JCM 1195 = DSM 20436 GCA_001042595.1
457 Bifidobacterium coryneforme GCA_000737865.1
CC
674 Bifidobacterium adolescentis ATCC 15703 GCA_000010425.1
543 Bifidobacterium dentium JCM 1195 = DSM 20436 GCA_001042595.1
530 Bifidobacterium angulatum DSM 20098 = JCM 7096 GCA_001025155.1
CD
594 Bifidobacterium adolescentis ATCC 15703 GCA_000010425.1
563 Bifidobacterium dentium JCM 1195 = DSM 20436 GCA_001042595.1
510 Bifidobacterium stellenboschense GCA_000741785.1
CE
884 Streptococcus mutans UA159 GCA_000007465.2
710 Streptococcus ratti FA-1 = DSM 20564 GCA_000286075.1
658 Streptococcus gordonii str. Challis substr. CH1 GCA_000017005.1
658 Streptococcus henryi DSM 19005 GCA_000376985.1
CF
640 Bacillus cereus ATCC 14579 GCA_000007825.1
630 Bacillus thuringiensis YBT-1518 GCA_000497525.2
628 Bacillus anthracis str. Ames GCA_000007845.1
628 Bacillus anthracis str. Sterne GCA_000008165.1
628 _5BBacillus thuringiensis_5D serovar konkukian str. 97-27 GCA_000008505.1
CG
641 Clostridium beijerinckii GCA_000833105.2
604 Clostridium saccharoperbutylacetonicum N1-4_28HMT_29 GCA_000340885.1
592 Clostridium saccharobutylicum DSM 13864 GCA_000473995.1
CH
685 Staphylococcus epidermidis ATCC 12228 GCA_000007645.1
642 Staphylococcus capitis subsp. capitis GCA_001028645.1
594 Staphylococcus haemolyticus JCSC1435 GCA_000009865.1
CI
657 Lactobacillus gasseri ATCC 33323 = JCM 1131 GCA_000014425.1
641 Lactobacillus hominis DSM 23910 = CRBIP 24.179 GCA_000296835.1
461 Lactobacillus iners DSM 13335 GCA_000160875.1
CJ
784 Clostridium beijerinckii GCA_000833105.2
737 Clostridium saccharoperbutylacetonicum N1-4_28HMT_29 GCA_000340885.1
705 Clostridium saccharobutylicum DSM 13864 GCA_000473995.1
CK
639 Clostridium beijerinckii GCA_000833105.2
605 Clostridium saccharoperbutylacetonicum N1-4_28HMT_29 GCA_000340885.1
598 Clostridium saccharobutylicum DSM 13864 GCA_000473995.1
CL
421 Bacillus anthracis str. Ames GCA_000007845.1
421 Bacillus anthracis str. Sterne GCA_000008165.1
421 _5BBacillus thuringiensis_5D serovar konkukian str. 97-27 GCA_000008505.1
413 Bacillus pseudomycoides DSM 12442 GCA_000161455.1
406 Bacillus cereus ATCC 14579 GCA_000007825.1
406 Bacillus thuringiensis YBT-1518 GCA_000497525.2
CM
798 Staphylococcus epidermidis ATCC 12228 GCA_000007645.1
749 Staphylococcus capitis subsp. capitis GCA_001028645.1
726 Staphylococcus hominis subsp. hominis C80 GCA_000183685.1
CN
741 Staphylococcus epidermidis ATCC 12228 GCA_000007645.1
729 Staphylococcus capitis subsp. capitis GCA_001028645.1
675 Staphylococcus aureus subsp. aureus NCTC 8325 GCA_000013425.1
CO
405 Clostridium beijerinckii GCA_000833105.2
404 Clostridium saccharoperbutylacetonicum N1-4_28HMT_29 GCA_000340885.1
360 Clostridium puniceum GCA_002006345.1

CP
611 Clostridium puniceum GCA_002006345.1
605 Clostridium beijerinckii GCA_000833105.2
603 Clostridium saccharoperbutylacetonicum N1-4_28HMT_29 GCA_000340885.1
CQ
604 Deinococcus radiodurans R1 GCA_000008565.1
351 Deinococcus deserti VCD115 GCA_000020685.1
307 Deinococcus marmoris DSM 12784 GCA_000701405.1
307 Deinococcus soli Cha et al. 2016 GCA_001007995.1
CR
684 Deinococcus radiodurans R1 GCA_000008565.1
373 Deinococcus deserti VCD115 GCA_000020685.1
370 Deinococcus gobiensis I-0 GCA_000252445.1
CS
529 Staphylococcus epidermidis ATCC 12228 GCA_000007645.1
521 Staphylococcus capitis subsp. capitis GCA_001028645.1
485 Staphylococcus aureus subsp. aureus NCTC 8325 GCA_000013425.1
CT
502 Staphylococcus epidermidis ATCC 12228 GCA_000007645.1
459 Staphylococcus capitis subsp. capitis GCA_001028645.1
457 Staphylococcus aureus subsp. aureus NCTC 8325 GCA_000013425.1
CU
669 Deinococcus radiodurans R1 GCA_000008565.1
388 Deinococcus soli Cha et al. 2016 GCA_001007995.1
380 Deinococcus gobiensis I-0 GCA_000252445.1
CV
733 Clostridium beijerinckii GCA_000833105.2
689 Clostridium saccharoperbutylacetonicum N1-4_28HMT_29 GCA_000340885.1
664 Clostridium puniceum GCA_002006345.1
CW
627 Rhodobacter sphaeroides 2.4.1 GCA_000012905.2
530 Rhodobacter sphaeroides ATCC 17025 GCA_000016405.1
460 Gemmobacter aquatilis GCA_900110025.1
CX
436 Lactobacillus gasseri ATCC 33323 = JCM 1131 GCA_000014425.1
422 Lactobacillus hominis DSM 23910 = CRBIP 24.179 GCA_000296835.1
323 Lactobacillus jensenii GCA_001936235.1
323 Lactobacillus psittaci DSM 15354 GCA_000425905.1
CY
739 Clostridium beijerinckii GCA_000833105.2
709 Clostridium saccharoperbutylacetonicum N1-4_28HMT_29 GCA_000340885.1
670 Clostridium saccharobutylicum DSM 13864 GCA_000473995.1
CZ
770 Deinococcus radiodurans R1 GCA_000008565.1
473 Deinococcus gobiensis I-0 GCA_000252445.1
450 Deinococcus deserti VCD115 GCA_000020685.1
D0
709 Shigella flexneri 2a str. 301 GCA_000006925.2
698 Escherichia coli UMN026 GCA_000026325.2
698 Escherichia coli str. K-12 substr. MG1655 GCA_000005845.2
693 Escherichia coli O157_3AH7 str. Sakai GCA_000008865.1
D1
802 Deinococcus radiodurans R1 GCA_000008565.1
591 Deinococcus deserti VCD115 GCA_000020685.1
556 Deinococcus puniceus GCA_001644565.1
D2
698 Enterococcus faecalis V583 GCA_000007785.1
651 Streptomyces cinnamoneus GCA_001885705.1
603 Enterococcus faecium DO GCA_000174395.2
D3
542 Lactobacillus gasseri ATCC 33323 = JCM 1131 GCA_000014425.1
485 Lactobacillus hominis DSM 23910 = CRBIP 24.179 GCA_000296835.1
328 Lactobacillus iners DSM 13335 GCA_000160875.1
D4
585 Rhodobacter sphaeroides 2.4.1 GCA_000012905.2
514 Rhodobacter sphaeroides ATCC 17025 GCA_000016405.1
469 Gemmobacter aquatilis GCA_900110025.1
D5
620 Clostridium beijerinckii GCA_000833105.2
597 Clostridium saccharobutylicum DSM 13864 GCA_000473995.1
586 Clostridium saccharoperbutylacetonicum N1-4_28HMT_29 GCA_000340885.1
D6
471 Rhodobacter sphaeroides 2.4.1 GCA_000012905.2
441 Rhodobacter sphaeroides ATCC 17025 GCA_000016405.1
363 Pseudorhodobacter psychrotolerans GCA_001294535.1
D7

635 Clostridium beijerinckii GCA_000833105.2
600 Clostridium saccharoperbutylacetonicum N1-4_28HMT_29 GCA_000340885.1
580 Clostridium saccharobutylicum DSM 13864 GCA_000473995.1
D8
681 Deinococcus radiodurans R1 GCA_000008565.1
441 Deinococcus deserti VCD115 GCA_000020685.1
389 Deinococcus gobiensis I-0 GCA_000252445.1
D9
627 Staphylococcus epidermidis ATCC 12228 GCA_000007645.1
578 Staphylococcus capitis subsp. capitis GCA_001028645.1
546 Staphylococcus lugdunensis HKU09-01 GCA_000025085.1
Da
825 Staphylococcus epidermidis ATCC 12228 GCA_000007645.1
754 Staphylococcus capitis subsp. capitis GCA_001028645.1
679 Staphylococcus lugdunensis HKU09-01 GCA_000025085.1
Db
661 Staphylococcus epidermidis ATCC 12228 GCA_000007645.1
623 Staphylococcus capitis subsp. capitis GCA_001028645.1
565 Staphylococcus warneri SG1 GCA_000332735.1
Dc
782 Deinococcus radiodurans R1 GCA_000008565.1
463 Deinococcus deserti VCD115 GCA_000020685.1
447 Deinococcus gobiensis I-0 GCA_000252445.1
Dd
697 Clostridium beijerinckii GCA_000833105.2
657 Clostridium saccharoperbutylacetonicum N1-4_28HMT_29 GCA_000340885.1
622 Clostridium saccharobutylicum DSM 13864 GCA_000473995.1
De
591 Deinococcus radiodurans R1 GCA_000008565.1
375 Deinococcus puniceus GCA_001644565.1
362 Deinococcus deserti VCD115 GCA_000020685.1
Df
705 Bacillus anthracis str. Ames GCA_000007845.1
705 Bacillus anthracis str. Sterne GCA_000008165.1
705 _5BBacillus thuringiensis_5D serovar konkukian str. 97-27 GCA_000008505.1
690 Bacillus cereus ATCC 14579 GCA_000007825.1
672 Bacillus thuringiensis YBT-1518 GCA_000497525.2
Dg
787 Staphylococcus epidermidis ATCC 12228 GCA_000007645.1
775 Staphylococcus capitis subsp. capitis GCA_001028645.1
706 Staphylococcus warneri SG1 GCA_000332735.1
Dh
412 Rhodobacter sphaeroides 2.4.1 GCA_000012905.2
397 Rhodobacter sphaeroides ATCC 17025 GCA_000016405.1
328 Gemmobacter megaterium GCA_900156815.1
Di
823 Bifidobacterium adolescentis ATCC 15703 GCA_000010425.1
728 Bifidobacterium dentium JCM 1195 = DSM 20436 GCA_001042595.1
665 Bifidobacterium angulatum DSM 20098 = JCM 7096 GCA_001025155.1
Dj
744 Lactobacillus gasseri ATCC 33323 = JCM 1131 GCA_000014425.1
662 Lactobacillus hominis DSM 23910 = CRBIP 24.179 GCA_000296835.1
498 Lactobacillus iners DSM 13335 GCA_000160875.1
Dk
715 Rhodobacter sphaeroides 2.4.1 GCA_000012905.2
645 Rhodobacter sphaeroides ATCC 17025 GCA_000016405.1
546 Thioclava indica GCA_000714545.1
Dl
848 Clostridium saccharoperbutylacetonicum N1-4_28HMT_29 GCA_000340885.1
814 Clostridium beijerinckii GCA_000833105.2
779 Clostridium puniceum GCA_002006345.1
Dm
626 Clostridium beijerinckii GCA_000833105.2
571 Clostridium saccharoperbutylacetonicum N1-4_28HMT_29 GCA_000340885.1
566 Clostridium saccharobutylicum DSM 13864 GCA_000473995.1
Dn
662 Rhodobacter sphaeroides 2.4.1 GCA_000012905.2
609 Rhodobacter sphaeroides ATCC 17025 GCA_000016405.1
506 Pseudorhodobacter ferrugineus DSM 5888 GCA_000420745.1
506 Pseudorhodobacter wandonensis GCA_001202035.1
Do
764 Escherichia coli O157_3AH7 str. Sakai GCA_000008865.1
764 Escherichia coli str. K-12 substr. MG1655 GCA_000005845.2
764 Shigella flexneri 2a str. 301 GCA_000006925.2
761 Escherichia coli O83_3AH1 str. NRG 857C GCA_000183345.1
759 Escherichia coli UMN026 GCA_000026325.2

Dp
754 Lactobacillus gasseri ATCC 33323 = JCM 1131 GCA_000014425.1
706 Lactobacillus hominis DSM 23910 = CRBIP 24.179 GCA_000296835.1
499 Lactobacillus hamsteri DSM 5661 = JCM 6256 GCA_000615445.1
Dq
687 Lactobacillus gasseri ATCC 33323 = JCM 1131 GCA_000014425.1
639 Lactobacillus hominis DSM 23910 = CRBIP 24.179 GCA_000296835.1
489 Lactobacillus iners DSM 13335 GCA_000160875.1
Dr
642 Rhodobacter sphaeroides 2.4.1 GCA_000012905.2
612 Rhodobacter sphaeroides ATCC 17025 GCA_000016405.1
458 Pseudorhodobacter psychrotolerans GCA_001294535.1
Ds
772 Rhodobacter sphaeroides 2.4.1 GCA_000012905.2
735 Rhodobacter sphaeroides ATCC 17025 GCA_000016405.1
609 Gemmobacter aquatilis GCA_900110025.1
Dt
632 Enterococcus faecalis V583 GCA_000007785.1
585 Streptomyces cinnamoneus GCA_001885705.1
559 Enterococcus thailandicus GCA_001652875.1
Du
621 Escherichia coli O104_3AH4 str. 2011C-3493 GCA_000299455.1
621 Escherichia coli str. K-12 substr. MG1655 GCA_000005845.2
611 Shigella flexneri 2a str. 301 GCA_000006925.2
600 Escherichia coli IAI39 GCA_000026345.1
600 Escherichia coli O157_3AH7 str. Sakai GCA_000008865.1
Dv
812 Rhodobacter sphaeroides 2.4.1 GCA_000012905.2
734 Rhodobacter sphaeroides ATCC 17025 GCA_000016405.1
613 Gemmobacter megaterium GCA_900156815.1
Dw
605 Bifidobacterium adolescentis ATCC 15703 GCA_000010425.1
479 Bifidobacterium angulatum DSM 20098 = JCM 7096 GCA_001025155.1
475 Bifidobacterium callitrichos DSM 23973 GCA_000741175.1
Dx
730 Lactobacillus gasseri ATCC 33323 = JCM 1131 GCA_000014425.1
689 Lactobacillus hominis DSM 23910 = CRBIP 24.179 GCA_000296835.1
568 Lactobacillus iners DSM 13335 GCA_000160875.1
Dy
736 Bifidobacterium adolescentis ATCC 15703 GCA_000010425.1
643 Bifidobacterium angulatum DSM 20098 = JCM 7096 GCA_001025155.1
603 Bifidobacterium dentium JCM 1195 = DSM 20436 GCA_001042595.1
Dz
649 Staphylococcus epidermidis ATCC 12228 GCA_000007645.1
597 Staphylococcus capitis subsp. capitis GCA_001028645.1
595 Staphylococcus warneri SG1 GCA_000332735.1
DA
758 Deinococcus radiodurans R1 GCA_000008565.1
463 Deinococcus deserti VCD115 GCA_000020685.1
430 Deinococcus gobiensis I-0 GCA_000252445.1
DB
694 Staphylococcus capitis subsp. capitis GCA_001028645.1
694 Staphylococcus epidermidis ATCC 12228 GCA_000007645.1
637 Staphylococcus haemolyticus JCSC1435 GCA_000009865.1
626 Staphylococcus aureus subsp. aureus NCTC 8325 GCA_000013425.1
DC
975 Lactobacillus gasseri ATCC 33323 = JCM 1131 GCA_000014425.1
895 Lactobacillus hominis DSM 23910 = CRBIP 24.179 GCA_000296835.1
737 Lactobacillus hamsteri DSM 5661 = JCM 6256 GCA_000615445.1
DD
744 Bifidobacterium adolescentis ATCC 15703 GCA_000010425.1
603 Bifidobacterium angulatum DSM 20098 = JCM 7096 GCA_001025155.1
562 Bifidobacterium callitrichos DSM 23973 GCA_000741175.1
DE
644 Bifidobacterium adolescentis ATCC 15703 GCA_000010425.1
507 Bifidobacterium dentium JCM 1195 = DSM 20436 GCA_001042595.1
492 Bifidobacterium angulatum DSM 20098 = JCM 7096 GCA_001025155.1
DF
594 Bifidobacterium adolescentis ATCC 15703 GCA_000010425.1
494 Bifidobacterium angulatum DSM 20098 = JCM 7096 GCA_001025155.1
469 Bifidobacterium dentium JCM 1195 = DSM 20436 GCA_001042595.1
DG
496 Staphylococcus epidermidis ATCC 12228 GCA_000007645.1
455 Staphylococcus hominis subsp. hominis C80 GCA_000183685.1
441 Staphylococcus lugdunensis HKU09-01 GCA_000025085.1
DH

608 Rhodobacter sphaeroides 2.4.1 GCA_000012905.2
574 Rhodobacter sphaeroides ATCC 17025 GCA_000016405.1
501 Gemmobacter aquatilis GCA_900110025.1
DI
674 Rhodobacter sphaeroides 2.4.1 GCA_000012905.2
593 Rhodobacter sphaeroides ATCC 17025 GCA_000016405.1
452 Pseudorhodobacter psychrotolerans GCA_001294535.1
DJ
687 Lactobacillus gasseri ATCC 33323 = JCM 1131 GCA_000014425.1
670 Lactobacillus hominis DSM 23910 = CRBIP 24.179 GCA_000296835.1
470 Lactobacillus iners DSM 13335 GCA_000160875.1
DK
745 Enterococcus faecalis V583 GCA_000007785.1
698 Streptomyces cinnamoneus GCA_001885705.1
637 Enterococcus rivorum GCA_001742285.1
DL
526 Staphylococcus epidermidis ATCC 12228 GCA_000007645.1
510 Staphylococcus capitis subsp. capitis GCA_001028645.1
494 Staphylococcus haemolyticus JCSC1435 GCA_000009865.1
DM
590 Bifidobacterium adolescentis ATCC 15703 GCA_000010425.1
475 Bifidobacterium callitrichos DSM 23973 GCA_000741175.1
470 Bifidobacterium dentium JCM 1195 = DSM 20436 GCA_001042595.1
DN
795 Escherichia coli O157_3AH7 str. Sakai GCA_000008865.1
795 Escherichia coli str. K-12 substr. MG1655 GCA_000005845.2
787 Escherichia coli O83_3AH1 str. NRG 857C GCA_000183345.1
787 Escherichia coli UMN026 GCA_000026325.2
786 Escherichia coli IAI39 GCA_000026345.1
DO
614 Escherichia coli str. K-12 substr. MG1655 GCA_000005845.2
608 Escherichia coli O157_3AH7 str. Sakai GCA_000008865.1
596 Escherichia coli UMN026 GCA_000026325.2
DP
652 Clostridium beijerinckii GCA_000833105.2
652 Clostridium saccharoperbutylacetonicum N1-4_28HMT_29 GCA_000340885.1
631 Clostridium puniceum GCA_002006345.1
628 Clostridium saccharobutylicum DSM 13864 GCA_000473995.1
DQ
639 Enterococcus faecalis V583 GCA_000007785.1
593 Streptomyces cinnamoneus GCA_001885705.1
553 Enterococcus faecium DO GCA_000174395.2
DR
654 Rhodobacter sphaeroides 2.4.1 GCA_000012905.2
592 Rhodobacter sphaeroides ATCC 17025 GCA_000016405.1
491 Defluviimonas alba GCA_001620265.1
DS
809 Escherichia coli O104_3AH4 str. 2011C-3493 GCA_000299455.1
809 Escherichia coli str. K-12 substr. MG1655 GCA_000005845.2
809 Shigella flexneri 2a str. 301 GCA_000006925.2
782 Escherichia coli O157_3AH7 str. Sakai GCA_000008865.1
770 Escherichia coli O83_3AH1 str. NRG 857C GCA_000183345.1
DT
769 Deinococcus radiodurans R1 GCA_000008565.1
489 Deinococcus puniceus GCA_001644565.1
485 Deinococcus hopiensis KR-140 GCA_900176165.1
DU
534 Bacillus anthracis str. Ames GCA_000007845.1
534 Bacillus anthracis str. Sterne GCA_000008165.1
534 _5BBacillus thuringiensis_5D serovar konkukian str. 97-27 GCA_000008505.1
521 Bacillus pseudomycoides DSM 12442 GCA_000161455.1
518 Bacillus cereus ATCC 14579 GCA_000007825.1
518 Bacillus thuringiensis YBT-1518 GCA_000497525.2
DV
554 Clostridium beijerinckii GCA_000833105.2
506 Clostridium saccharobutylicum DSM 13864 GCA_000473995.1
503 Clostridium butyricum GCA_001456065.2
503 Clostridium saccharoperbutylacetonicum N1-4_28HMT_29 GCA_000340885.1
DW
680 Staphylococcus epidermidis ATCC 12228 GCA_000007645.1
650 Staphylococcus capitis subsp. capitis GCA_001028645.1
584 Staphylococcus haemolyticus JCSC1435 GCA_000009865.1
DX
744 Streptococcus mutans UA159 GCA_000007465.2
564 Streptococcus ratti FA-1 = DSM 20564 GCA_000286075.1
519 Streptococcus macacae NCTC 11558 GCA_000187995.3

DY
390 Clostridium beijerinckii GCA_000833105.2
351 Clostridium saccharoperbutylacetonicum N1-4_28HMT_29 GCA_000340885.1
350 Clostridium butyricum GCA_001456065.2
DZ
790 Bacillus anthracis str. Ames GCA_000007845.1
790 Bacillus anthracis str. Sterne GCA_000008165.1
790 _5BBacillus thuringiensis_5D serovar konkukian str. 97-27 GCA_000008505.1
788 Bacillus pseudomycoides DSM 12442 GCA_000161455.1
775 Bacillus cereus ATCC 14579 GCA_000007825.1
E0
371 Bifidobacterium adolescentis ATCC 15703 GCA_000010425.1
313 Bifidobacterium angulatum DSM 20098 = JCM 7096 GCA_001025155.1
307 Bifidobacterium dentium JCM 1195 = DSM 20436 GCA_001042595.1
E1
790 Bifidobacterium adolescentis ATCC 15703 GCA_000010425.1
656 Bifidobacterium dentium JCM 1195 = DSM 20436 GCA_001042595.1
630 Bifidobacterium angulatum DSM 20098 = JCM 7096 GCA_001025155.1
E2
836 Enterococcus faecalis V583 GCA_000007785.1
804 Streptomyces cinnamoneus GCA_001885705.1
719 Enterococcus rivorum GCA_001742285.1
E3
702 Staphylococcus epidermidis ATCC 12228 GCA_000007645.1
673 Staphylococcus capitis subsp. capitis GCA_001028645.1
633 Staphylococcus haemolyticus JCSC1435 GCA_000009865.1
E4
628 Deinococcus radiodurans R1 GCA_000008565.1
405 Deinococcus deserti VCD115 GCA_000020685.1
383 Deinococcus gobiensis I-0 GCA_000252445.1
E5
855 Streptococcus mutans UA159 GCA_000007465.2
633 Streptococcus ratti FA-1 = DSM 20564 GCA_000286075.1
603 Streptococcus gordonii str. Challis substr. CH1 GCA_000017005.1
E6
726 Deinococcus radiodurans R1 GCA_000008565.1
400 Deinococcus hopiensis KR-140 GCA_900176165.1
392 Deinococcus deserti VCD115 GCA_000020685.1
E7
907 Clostridium beijerinckii GCA_000833105.2
883 Clostridium saccharoperbutylacetonicum N1-4_28HMT_29 GCA_000340885.1
869 Clostridium saccharobutylicum DSM 13864 GCA_000473995.1
E8
311 Clostridium beijerinckii GCA_000833105.2
296 Clostridium saccharobutylicum DSM 13864 GCA_000473995.1
287 Clostridium butyricum GCA_001456065.2
E9
684 Streptococcus mutans UA159 GCA_000007465.2
482 Streptococcus ratti FA-1 = DSM 20564 GCA_000286075.1
445 Streptococcus macacae NCTC 11558 GCA_000187995.3
Ea
532 Bifidobacterium adolescentis ATCC 15703 GCA_000010425.1
528 Bifidobacterium dentium JCM 1195 = DSM 20436 GCA_001042595.1
473 Bifidobacterium angulatum DSM 20098 = JCM 7096 GCA_001025155.1
Eb
725 Clostridium beijerinckii GCA_000833105.2
689 Clostridium saccharoperbutylacetonicum N1-4_28HMT_29 GCA_000340885.1
670 Clostridium puniceum GCA_002006345.1
Ec
505 Escherichia coli O157_3AH7 str. Sakai GCA_000008865.1
505 Escherichia coli UMN026 GCA_000026325.2
505 Escherichia coli str. K-12 substr. MG1655 GCA_000005845.2
486 Escherichia coli IAI39 GCA_000026345.1
486 Escherichia coli O83_3AH1 str. NRG 857C GCA_000183345.1
486 Shigella dysenteriae Sd197 GCA_000012005.1
486 Shigella flexneri 2a str. 301 GCA_000006925.2
465 Escherichia coli O104_3AH4 str. 2011C-3493 GCA_000299455.1
Ed
581 Clostridium beijerinckii GCA_000833105.2
556 Clostridium puniceum GCA_002006345.1
550 Clostridium saccharoperbutylacetonicum N1-4_28HMT_29 GCA_000340885.1
Ee
757 Deinococcus radiodurans R1 GCA_000008565.1
531 Deinococcus gobiensis I-0 GCA_000252445.1
527 Deinococcus hopiensis KR-140 GCA_900176165.1
Ef

828 Lactobacillus gasseri ATCC 33323 = JCM 1131 GCA_000014425.1
757 Lactobacillus hominis DSM 23910 = CRBIP 24.179 GCA_000296835.1
514 Lactobacillus psittaci DSM 15354 GCA_000425905.1
Eg
671 Staphylococcus epidermidis ATCC 12228 GCA_000007645.1
639 Staphylococcus capitis subsp. capitis GCA_001028645.1
582 Megasphaera cerevisiae DSM 20462 GCA_001045675.1
582 Staphylococcus warneri SG1 GCA_000332735.1
Eh
858 Staphylococcus epidermidis ATCC 12228 GCA_000007645.1
801 Staphylococcus capitis subsp. capitis GCA_001028645.1
756 Staphylococcus lugdunensis HKU09-01 GCA_000025085.1
Ei
465 Bifidobacterium adolescentis ATCC 15703 GCA_000010425.1
334 Bifidobacterium dentium JCM 1195 = DSM 20436 GCA_001042595.1
316 Bifidobacterium angulatum DSM 20098 = JCM 7096 GCA_001025155.1
Ej
653 Deinococcus radiodurans R1 GCA_000008565.1
427 Deinococcus deserti VCD115 GCA_000020685.1
404 Deinococcus frigens DSM 12807 GCA_000701425.1
404 Deinococcus soli Cha et al. 2016 GCA_001007995.1
Ek
660 Bacillus anthracis str. Ames GCA_000007845.1
660 Bacillus anthracis str. Sterne GCA_000008165.1
660 _5BBacillus thuringiensis_5D serovar konkukian str. 97-27 GCA_000008505.1
651 Bacillus cereus ATCC 14579 GCA_000007825.1
639 Bacillus pseudomycoides DSM 12442 GCA_000161455.1
El
478 Clostridium beijerinckii GCA_000833105.2
475 Clostridium saccharobutylicum DSM 13864 GCA_000473995.1
471 Clostridium saccharoperbutylacetonicum N1-4_28HMT_29 GCA_000340885.1
Em
518 Lactobacillus gasseri ATCC 33323 = JCM 1131 GCA_000014425.1
467 Lactobacillus hominis DSM 23910 = CRBIP 24.179 GCA_000296835.1
317 Lactobacillus iners DSM 13335 GCA_000160875.1
En
805 Streptococcus mutans UA159 GCA_000007465.2
586 Streptococcus gordonii str. Challis substr. CH1 GCA_000017005.1
576 Streptococcus ratti FA-1 = DSM 20564 GCA_000286075.1
Eo
367 Deinococcus radiodurans R1 GCA_000008565.1
259 Deinococcus gobiensis I-0 GCA_000252445.1
211 Deinococcus deserti VCD115 GCA_000020685.1
Ep
896 Bacillus anthracis str. Ames GCA_000007845.1
896 Bacillus anthracis str. Sterne GCA_000008165.1
890 Bacillus cereus ATCC 14579 GCA_000007825.1
890 _5BBacillus thuringiensis_5D serovar konkukian str. 97-27 GCA_000008505.1
881 Bacillus pseudomycoides DSM 12442 GCA_000161455.1
Eq
661 Rhodobacter sphaeroides 2.4.1 GCA_000012905.2
585 Rhodobacter sphaeroides ATCC 17025 GCA_000016405.1
503 Gemmobacter aquatilis GCA_900110025.1
Er
919 Rhodobacter sphaeroides 2.4.1 GCA_000012905.2
855 Rhodobacter sphaeroides ATCC 17025 GCA_000016405.1
711 Gemmobacter aquatilis GCA_900110025.1
Es
683 Clostridium beijerinckii GCA_000833105.2
673 Clostridium saccharoperbutylacetonicum N1-4_28HMT_29 GCA_000340885.1
631 Clostridium saccharobutylicum DSM 13864 GCA_000473995.1
Et
874 Streptococcus mutans UA159 GCA_000007465.2
655 Streptococcus ratti FA-1 = DSM 20564 GCA_000286075.1
611 Streptococcus gordonii str. Challis substr. CH1 GCA_000017005.1
Eu
804 Clostridium beijerinckii GCA_000833105.2
768 Clostridium saccharoperbutylacetonicum N1-4_28HMT_29 GCA_000340885.1
742 Clostridium puniceum GCA_002006345.1
Ev
679 Lactobacillus gasseri ATCC 33323 = JCM 1131 GCA_000014425.1
615 Lactobacillus hominis DSM 23910 = CRBIP 24.179 GCA_000296835.1
451 Lactobacillus psittaci DSM 15354 GCA_000425905.1
Ew
727 Streptococcus mutans UA159 GCA_000007465.2
569 Streptococcus gordonii str. Challis substr. CH1 GCA_000017005.1

560 Streptococcus ratti FA-1 = DSM 20564 GCA_000286075.1
Ex
537 Rhodobacter sphaeroides 2.4.1 GCA_000012905.2
503 Rhodobacter sphaeroides ATCC 17025 GCA_000016405.1
391 Pseudorhodobacter ferrugineus DSM 5888 GCA_000420745.1
Ey
605 Rhodobacter sphaeroides 2.4.1 GCA_000012905.2
540 Rhodobacter sphaeroides ATCC 17025 GCA_000016405.1
437 Thioclava nitratireducens GCA_001940525.2
Ez
514 Streptococcus mutans UA159 GCA_000007465.2
362 Streptococcus gordonii str. Challis substr. CH1 GCA_000017005.1
338 Streptococcus iniae GCA_000831485.1
EA
901 Streptococcus mutans UA159 GCA_000007465.2
654 Streptococcus ratti FA-1 = DSM 20564 GCA_000286075.1
600 Streptococcus gordonii str. Challis substr. CH1 GCA_000017005.1
EB
719 Rhodobacter sphaeroides 2.4.1 GCA_000012905.2
615 Rhodobacter sphaeroides ATCC 17025 GCA_000016405.1
521 Pseudorhodobacter ferrugineus DSM 5888 GCA_000420745.1
EC
680 Staphylococcus epidermidis ATCC 12228 GCA_000007645.1
613 Staphylococcus capitis subsp. capitis GCA_001028645.1
595 Staphylococcus warneri SG1 GCA_000332735.1
ED
700 Streptococcus mutans UA159 GCA_000007465.2
468 Streptococcus ratti FA-1 = DSM 20564 GCA_000286075.1
446 Streptococcus gordonii str. Challis substr. CH1 GCA_000017005.1
EE
946 Staphylococcus epidermidis ATCC 12228 GCA_000007645.1
922 Staphylococcus capitis subsp. capitis GCA_001028645.1
857 Staphylococcus haemolyticus JCSC1435 GCA_000009865.1
EF
700 Lactobacillus gasseri ATCC 33323 = JCM 1131 GCA_000014425.1
668 Lactobacillus hominis DSM 23910 = CRBIP 24.179 GCA_000296835.1
525 Lactobacillus psittaci DSM 15354 GCA_000425905.1
EG
812 Rhodobacter sphaeroides 2.4.1 GCA_000012905.2
723 Rhodobacter sphaeroides ATCC 17025 GCA_000016405.1
603 Gemmobacter megaterium GCA_900156815.1
EH
655 Streptococcus mutans UA159 GCA_000007465.2
448 Streptococcus ratti FA-1 = DSM 20564 GCA_000286075.1
426 Streptococcus gordonii str. Challis substr. CH1 GCA_000017005.1
EI
568 Staphylococcus epidermidis ATCC 12228 GCA_000007645.1
542 Staphylococcus capitis subsp. capitis GCA_001028645.1
518 Staphylococcus simulans GCA_001559115.1
EJ
715 Clostridium beijerinckii GCA_000833105.2
707 Clostridium saccharoperbutylacetonicum N1-4_28HMT_29 GCA_000340885.1
696 Clostridium butyricum GCA_001456065.2
EK
719 Bifidobacterium adolescentis ATCC 15703 GCA_000010425.1
544 Bifidobacterium angulatum DSM 20098 = JCM 7096 GCA_001025155.1
537 Bifidobacterium dentium JCM 1195 = DSM 20436 GCA_001042595.1
EL
610 Lactobacillus gasseri ATCC 33323 = JCM 1131 GCA_000014425.1
540 Lactobacillus hominis DSM 23910 = CRBIP 24.179 GCA_000296835.1
376 Lactobacillus iners DSM 13335 GCA_000160875.1
EM
473 Enterococcus faecalis V583 GCA_000007785.1
438 Enterococcus haemoperoxidus ATCC BAA-382 GCA_000407165.1
435 Enterococcus rivorum GCA_001742285.1
EN
724 Staphylococcus epidermidis ATCC 12228 GCA_000007645.1
688 Staphylococcus lugdunensis HKU09-01 GCA_000025085.1
677 Staphylococcus capitis subsp. capitis GCA_001028645.1
EO
821 Bacillus pseudomycoides DSM 12442 GCA_000161455.1
819 Bacillus anthracis str. Ames GCA_000007845.1
819 Bacillus anthracis str. Sterne GCA_000008165.1
819 Bacillus cereus ATCC 14579 GCA_000007825.1
819 _5BBacillus thuringiensis_5D serovar konkukian str. 97-27 GCA_000008505.1
797 Bacillus mycoides GCA_000832605.1

EP
879 Streptococcus mutans UA159 GCA_000007465.2
659 Streptococcus ratti FA-1 = DSM 20564 GCA_000286075.1
609 Streptococcus gordonii str. Challis substr. CH1 GCA_000017005.1
EQ
662 Lactobacillus gasseri ATCC 33323 = JCM 1131 GCA_000014425.1
634 Lactobacillus hominis DSM 23910 = CRBIP 24.179 GCA_000296835.1
503 Lactobacillus psittaci DSM 15354 GCA_000425905.1
ER
564 Staphylococcus epidermidis ATCC 12228 GCA_000007645.1
509 Staphylococcus capitis subsp. capitis GCA_001028645.1
459 Staphylococcus simulans GCA_001559115.1
ES
608 Clostridium beijerinckii GCA_000833105.2
584 Clostridium saccharoperbutylacetonicum N1-4_28HMT_29 GCA_000340885.1
583 Clostridium saccharobutylicum DSM 13864 GCA_000473995.1
ET
504 Staphylococcus epidermidis ATCC 12228 GCA_000007645.1
498 Staphylococcus capitis subsp. capitis GCA_001028645.1
467 Staphylococcus aureus subsp. aureus NCTC 8325 GCA_000013425.1
467 Staphylococcus haemolyticus JCSC1435 GCA_000009865.1
467 Staphylococcus hominis subsp. hominis C80 GCA_000183685.1
EU
634 Bifidobacterium adolescentis ATCC 15703 GCA_000010425.1
503 Bifidobacterium angulatum DSM 20098 = JCM 7096 GCA_001025155.1
496 Bifidobacterium dentium JCM 1195 = DSM 20436 GCA_001042595.1
EV
561 Staphylococcus epidermidis ATCC 12228 GCA_000007645.1
507 Staphylococcus aureus subsp. aureus NCTC 8325 GCA_000013425.1
506 Staphylococcus capitis subsp. capitis GCA_001028645.1
EW
734 Rhodobacter sphaeroides 2.4.1 GCA_000012905.2
651 Rhodobacter sphaeroides ATCC 17025 GCA_000016405.1
547 Gemmobacter aquatilis GCA_900110025.1
EX
519 Streptococcus mutans UA159 GCA_000007465.2
412 Streptococcus ratti FA-1 = DSM 20564 GCA_000286075.1
367 Streptococcus criceti HS-6 GCA_000187975.3
EY
729 Bifidobacterium adolescentis ATCC 15703 GCA_000010425.1
631 Bifidobacterium dentium JCM 1195 = DSM 20436 GCA_001042595.1
563 Bifidobacterium angulatum DSM 20098 = JCM 7096 GCA_001025155.1
EZ
609 Bifidobacterium adolescentis ATCC 15703 GCA_000010425.1
531 Bifidobacterium dentium JCM 1195 = DSM 20436 GCA_001042595.1
529 Bifidobacterium angulatum DSM 20098 = JCM 7096 GCA_001025155.1
F0
834 Lactobacillus gasseri ATCC 33323 = JCM 1131 GCA_000014425.1
726 Lactobacillus hominis DSM 23910 = CRBIP 24.179 GCA_000296835.1
540 Lactobacillus iners DSM 13335 GCA_000160875.1
F1
216 Bifidobacterium adolescentis ATCC 15703 GCA_000010425.1
215 Bifidobacterium dentium JCM 1195 = DSM 20436 GCA_001042595.1
194 Bifidobacterium breve DSM 20213 = JCM 1192 GCA_001025175.1
194 Bifidobacterium lemurum GCA_001895165.1
F2
495 Clostridium beijerinckii GCA_000833105.2
455 Clostridium saccharoperbutylacetonicum N1-4_28HMT_29 GCA_000340885.1
417 Clostridium butyricum GCA_001456065.2
F3
506 Clostridium beijerinckii GCA_000833105.2
462 Clostridium saccharoperbutylacetonicum N1-4_28HMT_29 GCA_000340885.1
460 Clostridium puniceum GCA_002006345.1
F4
602 Clostridium beijerinckii GCA_000833105.2
593 Clostridium saccharobutylicum DSM 13864 GCA_000473995.1
591 Clostridium saccharoperbutylacetonicum N1-4_28HMT_29 GCA_000340885.1
F5
278 Escherichia coli IAI39 GCA_000026345.1
278 Escherichia coli O104_3AH4 str. 2011C-3493 GCA_000299455.1
278 Escherichia coli O157_3AH7 str. Sakai GCA_000008865.1
278 Escherichia coli str. K-12 substr. MG1655 GCA_000005845.2
278 Shigella dysenteriae Sd197 GCA_000012005.1
278 Shigella flexneri 2a str. 301 GCA_000006925.2
270 Escherichia coli O83_3AH1 str. NRG 857C GCA_000183345.1
270 Escherichia coli UMN026 GCA_000026325.2

246 Tumebacillus flagellatus GCA_000714935.1
F6
721 Staphylococcus epidermidis ATCC 12228 GCA_000007645.1
679 Staphylococcus capitis subsp. capitis GCA_001028645.1
626 Staphylococcus aureus subsp. aureus NCTC 8325 GCA_000013425.1
F7
807 Streptococcus mutans UA159 GCA_000007465.2
577 Streptococcus gordonii str. Challis substr. CH1 GCA_000017005.1
560 Streptococcus equinus GCA_000964315.1
F8
691 Rhodobacter sphaeroides 2.4.1 GCA_000012905.2
620 Rhodobacter sphaeroides ATCC 17025 GCA_000016405.1
515 Pseudorhodobacter ferrugineus DSM 5888 GCA_000420745.1
F9
365 Bifidobacterium adolescentis ATCC 15703 GCA_000010425.1
286 Bifidobacterium angulatum DSM 20098 = JCM 7096 GCA_001025155.1
269 Bifidobacterium dentium JCM 1195 = DSM 20436 GCA_001042595.1
Fa
308 Rhodobacter sphaeroides 2.4.1 GCA_000012905.2
294 Rhodobacter sphaeroides ATCC 17025 GCA_000016405.1
241 Gemmobacter aquatilis GCA_900110025.1
Fb
722 Staphylococcus epidermidis ATCC 12228 GCA_000007645.1
664 Staphylococcus capitis subsp. capitis GCA_001028645.1
601 Megasphaera cerevisiae DSM 20462 GCA_001045675.1
601 Staphylococcus warneri SG1 GCA_000332735.1
Fc
868 Streptococcus mutans UA159 GCA_000007465.2
640 Streptococcus ratti FA-1 = DSM 20564 GCA_000286075.1
625 Streptococcus gordonii str. Challis substr. CH1 GCA_000017005.1
Fd
512 Rhodobacter sphaeroides 2.4.1 GCA_000012905.2
451 Rhodobacter sphaeroides ATCC 17025 GCA_000016405.1
386 Pseudorhodobacter ferrugineus DSM 5888 GCA_000420745.1
Fe
716 Rhodobacter sphaeroides 2.4.1 GCA_000012905.2
594 Rhodobacter sphaeroides ATCC 17025 GCA_000016405.1
520 Gemmobacter aquatilis GCA_900110025.1
Ff
423 Deinococcus radiodurans R1 GCA_000008565.1
262 Deinococcus proteolyticus MRP GCA_000190555.1
245 Deinococcus frigens DSM 12807 GCA_000701425.1
Fg
597 Rhodobacter sphaeroides 2.4.1 GCA_000012905.2
505 Rhodobacter sphaeroides ATCC 17025 GCA_000016405.1
406 Gemmobacter aquatilis GCA_900110025.1
Fh
713 Staphylococcus epidermidis ATCC 12228 GCA_000007645.1
684 Staphylococcus capitis subsp. capitis GCA_001028645.1
623 Staphylococcus simulans GCA_001559115.1
Fi
752 Streptococcus mutans UA159 GCA_000007465.2
536 Streptococcus ratti FA-1 = DSM 20564 GCA_000286075.1
529 Streptococcus equinus GCA_000964315.1
529 Streptococcus henryi DSM 19005 GCA_000376985.1
Fj
438 Clostridium saccharoperbutylacetonicum N1-4_28HMT_29 GCA_000340885.1
425 Clostridium beijerinckii GCA_000833105.2
404 Clostridium saccharobutylicum DSM 13864 GCA_000473995.1
Fk
433 Deinococcus radiodurans R1 GCA_000008565.1
298 Deinococcus marmoris DSM 12784 GCA_000701405.1
295 Deinococcus soli Cha et al. 2016 GCA_001007995.1
Fl
980 Bacillus anthracis str. Ames GCA_000007845.1
980 Bacillus anthracis str. Sterne GCA_000008165.1
980 _5BBacillus thuringiensis_5D serovar konkukian str. 97-27 GCA_000008505.1
971 Bacillus cereus ATCC 14579 GCA_000007825.1
956 Bacillus thuringiensis YBT-1518 GCA_000497525.2
Fm
886 Bifidobacterium adolescentis ATCC 15703 GCA_000010425.1
757 Bifidobacterium dentium JCM 1195 = DSM 20436 GCA_001042595.1
730 Bifidobacterium angulatum DSM 20098 = JCM 7096 GCA_001025155.1
Fn
840 Lactobacillus gasseri ATCC 33323 = JCM 1131 GCA_000014425.1
740 Lactobacillus hominis DSM 23910 = CRBIP 24.179 GCA_000296835.1

553 Lactobacillus gallinarum GCA_001314245.2
Fo
397 Deinococcus radiodurans R1 GCA_000008565.1
273 Deinococcus gobiensis I-0 GCA_000252445.1
265 Deinococcus soli Cha et al. 2016 GCA_001007995.1
Fp
620 Clostridium beijerinckii GCA_000833105.2
607 Clostridium saccharobutylicum DSM 13864 GCA_000473995.1
602 Clostridium saccharoperbutylacetonicum N1-4_28HMT_29 GCA_000340885.1
Fq
645 Clostridium beijerinckii GCA_000833105.2
633 Clostridium saccharoperbutylacetonicum N1-4_28HMT_29 GCA_000340885.1
604 Clostridium puniceum GCA_002006345.1
Fr
794 Lactobacillus gasseri ATCC 33323 = JCM 1131 GCA_000014425.1
720 Lactobacillus hominis DSM 23910 = CRBIP 24.179 GCA_000296835.1
515 Lactobacillus iners DSM 13335 GCA_000160875.1
Fs
374 Streptococcus mutans UA159 GCA_000007465.2
267 Streptococcus iniae GCA_000831485.1
267 Streptococcus uberis 0140J GCA_000009545.1
266 Streptococcus dysgalactiae subsp. equisimilis AC-2713 GCA_000317855.1
Ft
663 Clostridium beijerinckii GCA_000833105.2
654 Clostridium saccharoperbutylacetonicum N1-4_28HMT_29 GCA_000340885.1
645 Clostridium saccharobutylicum DSM 13864 GCA_000473995.1
Fu
695 Staphylococcus epidermidis ATCC 12228 GCA_000007645.1
654 Staphylococcus capitis subsp. capitis GCA_001028645.1
613 Staphylococcus lugdunensis HKU09-01 GCA_000025085.1
Fv
835 Lactobacillus gasseri ATCC 33323 = JCM 1131 GCA_000014425.1
807 Lactobacillus hominis DSM 23910 = CRBIP 24.179 GCA_000296835.1
632 Lactobacillus jensenii GCA_001936235.1
Fw
588 Bacillus anthracis str. Ames GCA_000007845.1
588 Bacillus anthracis str. Sterne GCA_000008165.1
588 _5BBacillus thuringiensis_5D serovar konkukian str. 97-27 GCA_000008505.1
578 Bacillus cereus ATCC 14579 GCA_000007825.1
578 Bacillus thuringiensis YBT-1518 GCA_000497525.2
577 Bacillus pseudomycoides DSM 12442 GCA_000161455.1
Fx
736 Staphylococcus epidermidis ATCC 12228 GCA_000007645.1
682 Staphylococcus capitis subsp. capitis GCA_001028645.1
665 Staphylococcus haemolyticus JCSC1435 GCA_000009865.1
Fy
746 Bifidobacterium adolescentis ATCC 15703 GCA_000010425.1
608 Bifidobacterium angulatum DSM 20098 = JCM 7096 GCA_001025155.1
589 Bifidobacterium dentium JCM 1195 = DSM 20436 GCA_001042595.1
Fz
475 Clostridium beijerinckii GCA_000833105.2
445 Clostridium butyricum GCA_001456065.2
441 Clostridium neonatale GCA_001458595.1
FA
709 Clostridium beijerinckii GCA_000833105.2
667 Clostridium puniceum GCA_002006345.1
667 Clostridium saccharoperbutylacetonicum N1-4_28HMT_29 GCA_000340885.1
634 Clostridium saccharobutylicum DSM 13864 GCA_000473995.1
FB
531 Escherichia coli str. K-12 substr. MG1655 GCA_000005845.2
530 Escherichia coli IAI39 GCA_000026345.1
530 Escherichia coli O157_3AH7 str. Sakai GCA_000008865.1
522 Kosakonia cowanii GCA_001975225.1
FC
638 Rhodobacter sphaeroides 2.4.1 GCA_000012905.2
586 Rhodobacter sphaeroides ATCC 17025 GCA_000016405.1
489 Pseudorhodobacter psychrotolerans GCA_001294535.1
FD
685 Staphylococcus epidermidis ATCC 12228 GCA_000007645.1
656 Staphylococcus capitis subsp. capitis GCA_001028645.1
639 Staphylococcus haemolyticus JCSC1435 GCA_000009865.1
FE
838 Rhodobacter sphaeroides 2.4.1 GCA_000012905.2
727 Rhodobacter sphaeroides ATCC 17025 GCA_000016405.1
606 Gemmobacter megaterium GCA_900156815.1
FF

938 Bacillus anthracis str. Ames GCA_000007845.1
938 Bacillus anthracis str. Sterne GCA_000008165.1
938 _5BBacillus thuringiensis_5D serovar konkukian str. 97-27 GCA_000008505.1
930 Bacillus cereus ATCC 14579 GCA_000007825.1
925 Bacillus pseudomycoides DSM 12442 GCA_000161455.1
FG
542 Bacillus anthracis str. Ames GCA_000007845.1
542 Bacillus anthracis str. Sterne GCA_000008165.1
542 Bacillus cereus ATCC 14579 GCA_000007825.1
542 Bacillus thuringiensis YBT-1518 GCA_000497525.2
542 _5BBacillus thuringiensis_5D serovar konkukian str. 97-27 GCA_000008505.1
527 Bacillus pseudomycoides DSM 12442 GCA_000161455.1
523 Bacillus mycoides GCA_000832605.1
FH
687 Deinococcus radiodurans R1 GCA_000008565.1
397 Deinococcus puniceus GCA_001644565.1
395 Deinococcus deserti VCD115 GCA_000020685.1
FI
493 Rhodobacter sphaeroides 2.4.1 GCA_000012905.2
420 Rhodobacter sphaeroides ATCC 17025 GCA_000016405.1
408 Defluviimonas alba GCA_001620265.1
FJ
630 Lactobacillus gasseri ATCC 33323 = JCM 1131 GCA_000014425.1
556 Lactobacillus hominis DSM 23910 = CRBIP 24.179 GCA_000296835.1
440 Lactobacillus iners DSM 13335 GCA_000160875.1
FK
962 Streptococcus mutans UA159 GCA_000007465.2
720 Streptococcus ratti FA-1 = DSM 20564 GCA_000286075.1
689 Streptococcus gallolyticus subsp. gallolyticus DSM 16831 GCA_002000985.1
FL
742 Lactobacillus gasseri ATCC 33323 = JCM 1131 GCA_000014425.1
655 Lactobacillus hominis DSM 23910 = CRBIP 24.179 GCA_000296835.1
512 Lactobacillus jensenii GCA_001936235.1
FM
819 Staphylococcus epidermidis ATCC 12228 GCA_000007645.1
786 Staphylococcus capitis subsp. capitis GCA_001028645.1
718 Staphylococcus warneri SG1 GCA_000332735.1
FN
661 Deinococcus radiodurans R1 GCA_000008565.1
352 Deinococcus gobiensis I-0 GCA_000252445.1
344 Deinococcus deserti VCD115 GCA_000020685.1
FO
485 Rhodobacter sphaeroides 2.4.1 GCA_000012905.2
468 Rhodobacter sphaeroides ATCC 17025 GCA_000016405.1
406 Defluviimonas alba GCA_001620265.1
FP
736 Staphylococcus epidermidis ATCC 12228 GCA_000007645.1
667 Staphylococcus capitis subsp. capitis GCA_001028645.1
626 Staphylococcus simulans GCA_001559115.1
FQ
661 Rhodobacter sphaeroides 2.4.1 GCA_000012905.2
607 Rhodobacter sphaeroides ATCC 17025 GCA_000016405.1
524 Gemmobacter aquatilis GCA_900110025.1
FR
678 Deinococcus radiodurans R1 GCA_000008565.1
421 Deinococcus gobiensis I-0 GCA_000252445.1
408 Deinococcus deserti VCD115 GCA_000020685.1
FS
894 Lactobacillus gasseri ATCC 33323 = JCM 1131 GCA_000014425.1
829 Lactobacillus hominis DSM 23910 = CRBIP 24.179 GCA_000296835.1
656 Lactobacillus jensenii GCA_001936235.1
FT
323 Rhodobacter sphaeroides 2.4.1 GCA_000012905.2
299 Rhodobacter sphaeroides ATCC 17025 GCA_000016405.1
234 Rhodobacter capsulatus SB 1003 GCA_000021865.1
FU
460 Clostridium beijerinckii GCA_000833105.2
437 Clostridium saccharoperbutylacetonicum N1-4_28HMT_29 GCA_000340885.1
412 Clostridium butyricum GCA_001456065.2
FV
629 Escherichia coli O157_3AH7 str. Sakai GCA_000008865.1
629 Escherichia coli UMN026 GCA_000026325.2
629 Escherichia coli str. K-12 substr. MG1655 GCA_000005845.2
628 Escherichia coli O83_3AH1 str. NRG 857C GCA_000183345.1
624 Escherichia coli IAI39 GCA_000026345.1
FW

426 Bifidobacterium adolescentis ATCC 15703 GCA_000010425.1
295 Bifidobacterium callitrichos DSM 23973 GCA_000741175.1
293 Bifidobacterium angulatum DSM 20098 = JCM 7096 GCA_001025155.1
FX
674 Streptococcus mutans UA159 GCA_000007465.2
478 Streptococcus ratti FA-1 = DSM 20564 GCA_000286075.1
397 Streptococcus gordonii str. Challis substr. CH1 GCA_000017005.1
FY
765 Clostridium beijerinckii GCA_000833105.2
737 Clostridium saccharoperbutylacetonicum N1-4_28HMT_29 GCA_000340885.1
708 Clostridium saccharobutylicum DSM 13864 GCA_000473995.1
FZ
822 Streptococcus mutans UA159 GCA_000007465.2
640 Streptococcus equinus GCA_000964315.1
637 Streptococcus gallolyticus subsp. gallolyticus DSM 16831 GCA_002000985.1
G0
800 Enterococcus faecalis V583 GCA_000007785.1
753 Streptomyces cinnamoneus GCA_001885705.1
712 Enterococcus rivorum GCA_001742285.1
G1
715 Staphylococcus epidermidis ATCC 12228 GCA_000007645.1
704 Staphylococcus capitis subsp. capitis GCA_001028645.1
672 Staphylococcus warneri SG1 GCA_000332735.1
G2
661 Lactobacillus gasseri ATCC 33323 = JCM 1131 GCA_000014425.1
656 Lactobacillus hominis DSM 23910 = CRBIP 24.179 GCA_000296835.1
463 Lactobacillus iners DSM 13335 GCA_000160875.1
G3
611 Clostridium beijerinckii GCA_000833105.2
598 Clostridium saccharoperbutylacetonicum N1-4_28HMT_29 GCA_000340885.1
585 Clostridium puniceum GCA_002006345.1
G4
724 Clostridium beijerinckii GCA_000833105.2
669 Clostridium saccharoperbutylacetonicum N1-4_28HMT_29 GCA_000340885.1
657 Clostridium puniceum GCA_002006345.1
G5
443 Rhodobacter sphaeroides 2.4.1 GCA_000012905.2
360 Rhodobacter sphaeroides ATCC 17025 GCA_000016405.1
349 Gemmobacter aquatilis GCA_900110025.1
G6
678 Clostridium beijerinckii GCA_000833105.2
619 Clostridium puniceum GCA_002006345.1
615 Clostridium saccharoperbutylacetonicum N1-4_28HMT_29 GCA_000340885.1
G7
780 Staphylococcus epidermidis ATCC 12228 GCA_000007645.1
765 Staphylococcus capitis subsp. capitis GCA_001028645.1
706 Staphylococcus hominis subsp. hominis C80 GCA_000183685.1
G8
727 Staphylococcus epidermidis ATCC 12228 GCA_000007645.1
712 Staphylococcus capitis subsp. capitis GCA_001028645.1
641 Staphylococcus warneri SG1 GCA_000332735.1
G9
762 Lactobacillus gasseri ATCC 33323 = JCM 1131 GCA_000014425.1
676 Lactobacillus hominis DSM 23910 = CRBIP 24.179 GCA_000296835.1
487 Lactobacillus helveticus GCA_001308285.1
Ga
674 Bifidobacterium adolescentis ATCC 15703 GCA_000010425.1
520 Bifidobacterium dentium JCM 1195 = DSM 20436 GCA_001042595.1
484 Bifidobacterium longum NCC2705 GCA_000007525.1
Gb
683 Lactobacillus gasseri ATCC 33323 = JCM 1131 GCA_000014425.1
606 Lactobacillus hominis DSM 23910 = CRBIP 24.179 GCA_000296835.1
462 Lactobacillus psittaci DSM 15354 GCA_000425905.1
Gc
719 Rhodobacter sphaeroides 2.4.1 GCA_000012905.2
633 Rhodobacter sphaeroides ATCC 17025 GCA_000016405.1
567 Gemmobacter aquatilis GCA_900110025.1
Gd
853 Clostridium beijerinckii GCA_000833105.2
843 Clostridium saccharoperbutylacetonicum N1-4_28HMT_29 GCA_000340885.1
781 Clostridium puniceum GCA_002006345.1
Ge
689 Lactobacillus gasseri ATCC 33323 = JCM 1131 GCA_000014425.1
646 Lactobacillus hominis DSM 23910 = CRBIP 24.179 GCA_000296835.1
508 Lactobacillus iners DSM 13335 GCA_000160875.1
Gf

714 Rhodobacter sphaeroides 2.4.1 GCA_000012905.2
655 Rhodobacter sphaeroides ATCC 17025 GCA_000016405.1
560 Gemmobacter aquatilis GCA_900110025.1
Gg
819 Lactobacillus gasseri ATCC 33323 = JCM 1131 GCA_000014425.1
743 Lactobacillus hominis DSM 23910 = CRBIP 24.179 GCA_000296835.1
566 Lactobacillus psittaci DSM 15354 GCA_000425905.1
Gh
589 Lactobacillus gasseri ATCC 33323 = JCM 1131 GCA_000014425.1
540 Lactobacillus hominis DSM 23910 = CRBIP 24.179 GCA_000296835.1
431 Lactobacillus iners DSM 13335 GCA_000160875.1
Gi
677 Lactobacillus gasseri ATCC 33323 = JCM 1131 GCA_000014425.1
586 Lactobacillus hominis DSM 23910 = CRBIP 24.179 GCA_000296835.1
453 Lactobacillus hamsteri DSM 5661 = JCM 6256 GCA_000615445.1
Gj
652 Bacillus thuringiensis YBT-1518 GCA_000497525.2
638 Bacillus anthracis str. Ames GCA_000007845.1
638 Bacillus anthracis str. Sterne GCA_000008165.1
638 Bacillus cereus ATCC 14579 GCA_000007825.1
638 _5BBacillus thuringiensis_5D serovar konkukian str. 97-27 GCA_000008505.1
611 Bacillus pseudomycoides DSM 12442 GCA_000161455.1
Gk
630 Streptococcus mutans UA159 GCA_000007465.2
476 Streptococcus ratti FA-1 = DSM 20564 GCA_000286075.1
451 Streptococcus gordonii str. Challis substr. CH1 GCA_000017005.1
451 Streptococcus macacae NCTC 11558 GCA_000187995.3
Gl
422 Lactobacillus gasseri ATCC 33323 = JCM 1131 GCA_000014425.1
407 Lactobacillus hominis DSM 23910 = CRBIP 24.179 GCA_000296835.1
317 Lactobacillus iners DSM 13335 GCA_000160875.1
Gm
325 Staphylococcus epidermidis ATCC 12228 GCA_000007645.1
277 Staphylococcus capitis subsp. capitis GCA_001028645.1
269 Staphylococcus hominis subsp. hominis C80 GCA_000183685.1
269 Staphylococcus lugdunensis HKU09-01 GCA_000025085.1
Gn
343 Deinococcus radiodurans R1 GCA_000008565.1
189 Deinococcus gobiensis I-0 GCA_000252445.1
187 Deinococcus deserti VCD115 GCA_000020685.1
Go
596 Staphylococcus epidermidis ATCC 12228 GCA_000007645.1
552 Staphylococcus capitis subsp. capitis GCA_001028645.1
509 Staphylococcus pettenkoferi GCA_002208805.1
Gp
709 Clostridium beijerinckii GCA_000833105.2
667 Clostridium saccharoperbutylacetonicum N1-4_28HMT_29 GCA_000340885.1
652 Clostridium puniceum GCA_002006345.1
Gq
672 Streptococcus mutans UA159 GCA_000007465.2
427 Streptococcus ratti FA-1 = DSM 20564 GCA_000286075.1
406 Streptococcus gordonii str. Challis substr. CH1 GCA_000017005.1
Gr
491 Deinococcus radiodurans R1 GCA_000008565.1
306 Deinococcus deserti VCD115 GCA_000020685.1
296 Deinococcus puniceus GCA_001644565.1
Gs
755 Lactobacillus gasseri ATCC 33323 = JCM 1131 GCA_000014425.1
687 Lactobacillus hominis DSM 23910 = CRBIP 24.179 GCA_000296835.1
557 Lactobacillus iners DSM 13335 GCA_000160875.1
Gt
875 Enterococcus faecalis V583 GCA_000007785.1
828 Streptomyces cinnamoneus GCA_001885705.1
737 Enterococcus rivorum GCA_001742285.1
Gu
723 Deinococcus radiodurans R1 GCA_000008565.1
454 Deinococcus gobiensis I-0 GCA_000252445.1
429 Deinococcus deserti VCD115 GCA_000020685.1
Gv
804 Escherichia coli O157_3AH7 str. Sakai GCA_000008865.1
804 Escherichia coli str. K-12 substr. MG1655 GCA_000005845.2
804 Shigella flexneri 2a str. 301 GCA_000006925.2
797 Escherichia coli O83_3AH1 str. NRG 857C GCA_000183345.1
794 Escherichia coli UMN026 GCA_000026325.2
Gw
628 Rhodobacter sphaeroides 2.4.1 GCA_000012905.2

552 Rhodobacter sphaeroides ATCC 17025 GCA_000016405.1
532 Gemmobacter aquatilis GCA_900110025.1
Gx
766 Rhodobacter sphaeroides 2.4.1 GCA_000012905.2
677 Rhodobacter sphaeroides ATCC 17025 GCA_000016405.1
659 Gemmobacter megaterium GCA_900156815.1
Gy
552 Clostridium beijerinckii GCA_000833105.2
528 Clostridium saccharoperbutylacetonicum N1-4_28HMT_29 GCA_000340885.1
501 Clostridium puniceum GCA_002006345.1
Gz
926 Streptococcus mutans UA159 GCA_000007465.2
674 Streptococcus gordonii str. Challis substr. CH1 GCA_000017005.1
666 Streptococcus ratti FA-1 = DSM 20564 GCA_000286075.1
GA
751 Rhodobacter sphaeroides 2.4.1 GCA_000012905.2
695 Rhodobacter sphaeroides ATCC 17025 GCA_000016405.1
581 Gemmobacter aquatilis GCA_900110025.1
GB
914 Enterococcus faecalis V583 GCA_000007785.1
867 Streptomyces cinnamoneus GCA_001885705.1
770 Enterococcus rivorum GCA_001742285.1
GC
923 Staphylococcus epidermidis ATCC 12228 GCA_000007645.1
875 Staphylococcus capitis subsp. capitis GCA_001028645.1
809 Staphylococcus hominis subsp. hominis C80 GCA_000183685.1
GD
734 Staphylococcus epidermidis ATCC 12228 GCA_000007645.1
668 Staphylococcus capitis subsp. capitis GCA_001028645.1
645 Staphylococcus aureus subsp. aureus NCTC 8325 GCA_000013425.1
GE
856 Deinococcus radiodurans R1 GCA_000008565.1
566 Deinococcus deserti VCD115 GCA_000020685.1
553 Deinococcus soli Cha et al. 2016 GCA_001007995.1
GF
504 Lactobacillus gasseri ATCC 33323 = JCM 1131 GCA_000014425.1
417 Lactobacillus hominis DSM 23910 = CRBIP 24.179 GCA_000296835.1
318 Lactobacillus kalixensis DSM 16043 GCA_001434335.1
GG
633 Streptococcus mutans UA159 GCA_000007465.2
440 Streptococcus ratti FA-1 = DSM 20564 GCA_000286075.1
378 Streptococcus gordonii str. Challis substr. CH1 GCA_000017005.1
GH
786 Rhodobacter sphaeroides 2.4.1 GCA_000012905.2
737 Rhodobacter sphaeroides ATCC 17025 GCA_000016405.1
561 Gemmobacter aquatilis GCA_900110025.1
561 Pseudorhodobacter ferrugineus DSM 5888 GCA_000420745.1
GI
738 Lactobacillus gasseri ATCC 33323 = JCM 1131 GCA_000014425.1
672 Lactobacillus hominis DSM 23910 = CRBIP 24.179 GCA_000296835.1
528 Lactobacillus iners DSM 13335 GCA_000160875.1
GJ
834 Staphylococcus epidermidis ATCC 12228 GCA_000007645.1
807 Staphylococcus capitis subsp. capitis GCA_001028645.1
763 Staphylococcus hominis subsp. hominis C80 GCA_000183685.1
GK
658 Rhodobacter sphaeroides 2.4.1 GCA_000012905.2
582 Rhodobacter sphaeroides ATCC 17025 GCA_000016405.1
534 Gemmobacter megaterium GCA_900156815.1
GL
459 Rhodobacter sphaeroides 2.4.1 GCA_000012905.2
401 Rhodobacter sphaeroides ATCC 17025 GCA_000016405.1
332 Gemmobacter aquatilis GCA_900110025.1
GM
915 Escherichia coli str. K-12 substr. MG1655 GCA_000005845.2
911 Escherichia coli IAI39 GCA_000026345.1
911 Escherichia coli O157_3AH7 str. Sakai GCA_000008865.1
908 Escherichia coli UMN026 GCA_000026325.2
GN
713 Deinococcus radiodurans R1 GCA_000008565.1
500 Deinococcus hopiensis KR-140 GCA_900176165.1
474 Deinococcus deserti VCD115 GCA_000020685.1
GO
522 Enterococcus faecalis V583 GCA_000007785.1
475 Streptomyces cinnamoneus GCA_001885705.1
446 Enterococcus faecium DO GCA_000174395.2

GP
732 Deinococcus radiodurans R1 GCA_000008565.1
451 Deinococcus soli Cha et al. 2016 GCA_001007995.1
444 Deinococcus deserti VCD115 GCA_000020685.1
GQ
526 Streptococcus mutans UA159 GCA_000007465.2
406 Streptococcus sobrinus DSM 20742 = ATCC 33478 GCA_000686605.1
397 Streptococcus ratti FA-1 = DSM 20564 GCA_000286075.1
GR
354 Bifidobacterium adolescentis ATCC 15703 GCA_000010425.1
290 Bifidobacterium dentium JCM 1195 = DSM 20436 GCA_001042595.1
241 Bifidobacterium thermophilum GCA_000741495.1
GS
504 Clostridium beijerinckii GCA_000833105.2
471 Clostridium saccharoperbutylacetonicum N1-4_28HMT_29 GCA_000340885.1
439 Clostridium butyricum GCA_001456065.2
GT
773 Rhodobacter sphaeroides 2.4.1 GCA_000012905.2
733 Rhodobacter sphaeroides ATCC 17025 GCA_000016405.1
635 Gemmobacter aquatilis GCA_900110025.1
GU
844 Streptococcus mutans UA159 GCA_000007465.2
643 Streptococcus gordonii str. Challis substr. CH1 GCA_000017005.1
623 Streptococcus ratti FA-1 = DSM 20564 GCA_000286075.1
GV
372 Streptococcus mutans UA159 GCA_000007465.2
304 Streptococcus ratti FA-1 = DSM 20564 GCA_000286075.1
251 Streptococcus macacae NCTC 11558 GCA_000187995.3
GW
739 Staphylococcus epidermidis ATCC 12228 GCA_000007645.1
682 Staphylococcus capitis subsp. capitis GCA_001028645.1
641 Staphylococcus haemolyticus JCSC1435 GCA_000009865.1
641 Staphylococcus warneri SG1 GCA_000332735.1
GX
676 Enterococcus faecalis V583 GCA_000007785.1
629 Streptomyces cinnamoneus GCA_001885705.1
620 Enterococcus asini ATCC 700915 GCA_000407365.1
GY
677 Bifidobacterium adolescentis ATCC 15703 GCA_000010425.1
549 Bifidobacterium dentium JCM 1195 = DSM 20436 GCA_001042595.1
543 Bifidobacterium breve DSM 20213 = JCM 1192 GCA_001025175.1
GZ
644 Staphylococcus epidermidis ATCC 12228 GCA_000007645.1
635 Staphylococcus capitis subsp. capitis GCA_001028645.1
589 Staphylococcus aureus subsp. aureus NCTC 8325 GCA_000013425.1
H0
633 Lactobacillus gasseri ATCC 33323 = JCM 1131 GCA_000014425.1
575 Lactobacillus hominis DSM 23910 = CRBIP 24.179 GCA_000296835.1
428 Lactobacillus iners DSM 13335 GCA_000160875.1
H1
852 Rhodobacter sphaeroides 2.4.1 GCA_000012905.2
775 Rhodobacter sphaeroides ATCC 17025 GCA_000016405.1
616 Pseudorhodobacter ferrugineus DSM 5888 GCA_000420745.1
H2
733 Clostridium beijerinckii GCA_000833105.2
733 Clostridium saccharoperbutylacetonicum N1-4_28HMT_29 GCA_000340885.1
702 Clostridium saccharobutylicum DSM 13864 GCA_000473995.1
674 Clostridium puniceum GCA_002006345.1
H3
588 Deinococcus radiodurans R1 GCA_000008565.1
391 Deinococcus deserti VCD115 GCA_000020685.1
385 Deinococcus hopiensis KR-140 GCA_900176165.1
H4
790 Bacillus thuringiensis YBT-1518 GCA_000497525.2
777 Bacillus anthracis str. Ames GCA_000007845.1
777 Bacillus anthracis str. Sterne GCA_000008165.1
777 _5BBacillus thuringiensis_5D serovar konkukian str. 97-27 GCA_000008505.1
776 Bacillus cereus ATCC 14579 GCA_000007825.1
H5
717 Streptococcus mutans UA159 GCA_000007465.2
428 Streptococcus ratti FA-1 = DSM 20564 GCA_000286075.1
415 Streptococcus equinus GCA_000964315.1
H6
545 Clostridium saccharoperbutylacetonicum N1-4_28HMT_29 GCA_000340885.1
538 Clostridium beijerinckii GCA_000833105.2
516 Clostridium saccharobutylicum DSM 13864 GCA_000473995.1

H7
665 Lactobacillus gasseri ATCC 33323 = JCM 1131 GCA_000014425.1
636 Lactobacillus hominis DSM 23910 = CRBIP 24.179 GCA_000296835.1
485 Lactobacillus iners DSM 13335 GCA_000160875.1
H8
408 Clostridium beijerinckii GCA_000833105.2
404 Clostridium saccharobutylicum DSM 13864 GCA_000473995.1
393 Clostridium saccharoperbutylacetonicum N1-4_28HMT_29 GCA_000340885.1
H9
798 Staphylococcus epidermidis ATCC 12228 GCA_000007645.1
749 Staphylococcus capitis subsp. capitis GCA_001028645.1
717 Staphylococcus haemolyticus JCSC1435 GCA_000009865.1
Ha
781 Streptococcus mutans UA159 GCA_000007465.2
579 Streptococcus ratti FA-1 = DSM 20564 GCA_000286075.1
563 Streptococcus gordonii str. Challis substr. CH1 GCA_000017005.1
Hb
774 Lactobacillus gasseri ATCC 33323 = JCM 1131 GCA_000014425.1
717 Lactobacillus hominis DSM 23910 = CRBIP 24.179 GCA_000296835.1
555 Lactobacillus iners DSM 13335 GCA_000160875.1
Hc
561 Streptococcus mutans UA159 GCA_000007465.2
495 Streptococcus gordonii str. Challis substr. CH1 GCA_000017005.1
461 Streptococcus salivarius GCA_000785515.1
461 Streptococcus thermophilus JIM 8232 GCA_000253395.1
Hd
688 Rhodobacter sphaeroides 2.4.1 GCA_000012905.2
595 Rhodobacter sphaeroides ATCC 17025 GCA_000016405.1
510 Defluviimonas alba GCA_001620265.1
He
747 Bacillus anthracis str. Ames GCA_000007845.1
747 Bacillus anthracis str. Sterne GCA_000008165.1
747 _5BBacillus thuringiensis_5D serovar konkukian str. 97-27 GCA_000008505.1
732 Bacillus pseudomycoides DSM 12442 GCA_000161455.1
722 Bacillus cereus ATCC 14579 GCA_000007825.1
722 Bacillus thuringiensis YBT-1518 GCA_000497525.2
Hf
749 Streptococcus mutans UA159 GCA_000007465.2
583 Streptococcus ratti FA-1 = DSM 20564 GCA_000286075.1
571 Streptococcus gordonii str. Challis substr. CH1 GCA_000017005.1
Hg
619 Streptococcus mutans UA159 GCA_000007465.2
398 Streptococcus halotolerans GCA_001598035.1
392 Streptococcus sobrinus DSM 20742 = ATCC 33478 GCA_000686605.1
Hh
716 Bifidobacterium adolescentis ATCC 15703 GCA_000010425.1
614 Bifidobacterium angulatum DSM 20098 = JCM 7096 GCA_001025155.1
589 Bifidobacterium stellenboschense GCA_000741785.1
Hi
821 Enterococcus faecalis V583 GCA_000007785.1
774 Streptomyces cinnamoneus GCA_001885705.1
733 Enterococcus phoeniculicola ATCC BAA-412 GCA_000407505.1
Hj
466 Enterococcus faecalis V583 GCA_000007785.1
434 Streptomyces cinnamoneus GCA_001885705.1
386 Enterococcus faecium DO GCA_000174395.2
Hk
502 Rhodobacter sphaeroides 2.4.1 GCA_000012905.2
476 Rhodobacter sphaeroides ATCC 17025 GCA_000016405.1
437 Thioclava dalianensis GCA_000715505.1
437 Thioclava indica GCA_000714545.1
Hl
786 _5BBacillus thuringiensis_5D serovar konkukian str. 97-27 GCA_000008505.1
785 Bacillus anthracis str. Ames GCA_000007845.1
785 Bacillus anthracis str. Sterne GCA_000008165.1
769 Bacillus pseudomycoides DSM 12442 GCA_000161455.1
Hm
698 Lactobacillus gasseri ATCC 33323 = JCM 1131 GCA_000014425.1
633 Lactobacillus hominis DSM 23910 = CRBIP 24.179 GCA_000296835.1
524 Lactobacillus iners DSM 13335 GCA_000160875.1
Hn
673 Clostridium beijerinckii GCA_000833105.2
635 Clostridium saccharoperbutylacetonicum N1-4_28HMT_29 GCA_000340885.1
609 Clostridium puniceum GCA_002006345.1
Ho
882 Deinococcus radiodurans R1 GCA_000008565.1

609 Deinococcus deserti VCD115 GCA_000020685.1
591 Deinococcus gobiensis I-0 GCA_000252445.1
Hp
697 Clostridium beijerinckii GCA_000833105.2
676 Clostridium saccharoperbutylacetonicum N1-4_28HMT_29 GCA_000340885.1
661 Clostridium saccharobutylicum DSM 13864 GCA_000473995.1
Hq
621 Staphylococcus epidermidis ATCC 12228 GCA_000007645.1
568 Staphylococcus hominis subsp. hominis C80 GCA_000183685.1
567 Staphylococcus capitis subsp. capitis GCA_001028645.1
Hr
731 Escherichia coli IAI39 GCA_000026345.1
724 Escherichia coli O157_3AH7 str. Sakai GCA_000008865.1
724 Escherichia coli str. K-12 substr. MG1655 GCA_000005845.2
722 Shigella dysenteriae Sd197 GCA_000012005.1
Hs
806 Rhodobacter sphaeroides 2.4.1 GCA_000012905.2
711 Rhodobacter sphaeroides ATCC 17025 GCA_000016405.1
574 Pseudorhodobacter wandonensis GCA_001202035.1
Ht
601 Streptococcus mutans UA159 GCA_000007465.2
459 Streptococcus ratti FA-1 = DSM 20564 GCA_000286075.1
412 Streptococcus equinus GCA_000964315.1
Hu
777 Lactobacillus gasseri ATCC 33323 = JCM 1131 GCA_000014425.1
706 Lactobacillus hominis DSM 23910 = CRBIP 24.179 GCA_000296835.1
557 Lactobacillus iners DSM 13335 GCA_000160875.1
Hv
620 Lactobacillus gasseri ATCC 33323 = JCM 1131 GCA_000014425.1
592 Lactobacillus hominis DSM 23910 = CRBIP 24.179 GCA_000296835.1
437 Lactobacillus iners DSM 13335 GCA_000160875.1
Hw
716 Escherichia coli str. K-12 substr. MG1655 GCA_000005845.2
704 Shigella flexneri 2a str. 301 GCA_000006925.2
698 Escherichia coli O104_3AH4 str. 2011C-3493 GCA_000299455.1
Hx
825 Escherichia coli O104_3AH4 str. 2011C-3493 GCA_000299455.1
825 Escherichia coli str. K-12 substr. MG1655 GCA_000005845.2
791 Shigella flexneri 2a str. 301 GCA_000006925.2
784 Escherichia coli O157_3AH7 str. Sakai GCA_000008865.1
Hy
571 Staphylococcus epidermidis ATCC 12228 GCA_000007645.1
526 Staphylococcus capitis subsp. capitis GCA_001028645.1
506 Staphylococcus lugdunensis HKU09-01 GCA_000025085.1
Hz
468 Enterococcus faecalis V583 GCA_000007785.1
422 Streptomyces cinnamoneus GCA_001885705.1
374 Enterococcus rivorum GCA_001742285.1
HA
674 Deinococcus radiodurans R1 GCA_000008565.1
461 Deinococcus gobiensis I-0 GCA_000252445.1
455 Deinococcus deserti VCD115 GCA_000020685.1
HB
897 Clostridium beijerinckii GCA_000833105.2
841 Clostridium saccharoperbutylacetonicum N1-4_28HMT_29 GCA_000340885.1
804 Clostridium saccharobutylicum DSM 13864 GCA_000473995.1
HC
431 Enterococcus faecalis V583 GCA_000007785.1
428 Enterococcus rivorum GCA_001742285.1
414 Enterococcus canis NBRC 100695 GCA_001544375.1
414 Enterococcus dispar ATCC 51266 GCA_000406945.1
HD
592 Deinococcus radiodurans R1 GCA_000008565.1
359 Deinococcus deserti VCD115 GCA_000020685.1
345 Deinococcus gobiensis I-0 GCA_000252445.1
HE
897 Bifidobacterium adolescentis ATCC 15703 GCA_000010425.1
782 Bifidobacterium dentium JCM 1195 = DSM 20436 GCA_001042595.1
737 Bifidobacterium stellenboschense GCA_000741785.1
HF
720 Bacillus anthracis str. Ames GCA_000007845.1
720 Bacillus anthracis str. Sterne GCA_000008165.1
720 Bacillus cereus ATCC 14579 GCA_000007825.1
720 _5BBacillus thuringiensis_5D serovar konkukian str. 97-27 GCA_000008505.1
706 Bacillus pseudomycoides DSM 12442 GCA_000161455.1
701 Bacillus thuringiensis YBT-1518 GCA_000497525.2

HG
555 Deinococcus radiodurans R1 GCA_000008565.1
309 Deinococcus deserti VCD115 GCA_000020685.1
305 Deinococcus gobiensis I-0 GCA_000252445.1
HH
611 Deinococcus radiodurans R1 GCA_000008565.1
366 Deinococcus deserti VCD115 GCA_000020685.1
329 Deinococcus frigens DSM 12807 GCA_000701425.1
HI
636 Lactobacillus gasseri ATCC 33323 = JCM 1131 GCA_000014425.1
573 Lactobacillus hominis DSM 23910 = CRBIP 24.179 GCA_000296835.1
352 Lactobacillus iners DSM 13335 GCA_000160875.1
HJ
675 Rhodobacter sphaeroides 2.4.1 GCA_000012905.2
634 Rhodobacter sphaeroides ATCC 17025 GCA_000016405.1
478 Gemmobacter aquatilis GCA_900110025.1
HK
858 Clostridium beijerinckii GCA_000833105.2
846 Clostridium saccharoperbutylacetonicum N1-4_28HMT_29 GCA_000340885.1
835 Clostridium saccharobutylicum DSM 13864 GCA_000473995.1
HL
834 Lactobacillus gasseri ATCC 33323 = JCM 1131 GCA_000014425.1
781 Lactobacillus hominis DSM 23910 = CRBIP 24.179 GCA_000296835.1
573 Lactobacillus psittaci DSM 15354 GCA_000425905.1
HM
484 Lactobacillus gasseri ATCC 33323 = JCM 1131 GCA_000014425.1
424 Lactobacillus hominis DSM 23910 = CRBIP 24.179 GCA_000296835.1
313 Lactobacillus iners DSM 13335 GCA_000160875.1
HN
586 Streptococcus mutans UA159 GCA_000007465.2
460 Streptococcus ratti FA-1 = DSM 20564 GCA_000286075.1
417 Streptococcus gordonii str. Challis substr. CH1 GCA_000017005.1
HO
465 Clostridium beijerinckii GCA_000833105.2
450 Clostridium saccharoperbutylacetonicum N1-4_28HMT_29 GCA_000340885.1
447 Clostridium saccharobutylicum DSM 13864 GCA_000473995.1
HP
864 Lactobacillus gasseri ATCC 33323 = JCM 1131 GCA_000014425.1
784 Lactobacillus hominis DSM 23910 = CRBIP 24.179 GCA_000296835.1
607 Lactobacillus iners DSM 13335 GCA_000160875.1
HQ
588 Clostridium beijerinckii GCA_000833105.2
559 Clostridium saccharoperbutylacetonicum N1-4_28HMT_29 GCA_000340885.1
554 Clostridium saccharobutylicum DSM 13864 GCA_000473995.1
HR
875 Streptococcus mutans UA159 GCA_000007465.2
689 Streptococcus ratti FA-1 = DSM 20564 GCA_000286075.1
640 Streptococcus equi subsp. zooepidemicus GCA_000026605.1
640 Streptococcus sobrinus DSM 20742 = ATCC 33478 GCA_000686605.1
HS
624 Deinococcus radiodurans R1 GCA_000008565.1
376 Deinococcus deserti VCD115 GCA_000020685.1
335 Deinococcus hopiensis KR-140 GCA_900176165.1
HT
756 Staphylococcus epidermidis ATCC 12228 GCA_000007645.1
718 Staphylococcus capitis subsp. capitis GCA_001028645.1
645 Staphylococcus haemolyticus JCSC1435 GCA_000009865.1
HU
545 Clostridium beijerinckii GCA_000833105.2
540 Clostridium saccharoperbutylacetonicum N1-4_28HMT_29 GCA_000340885.1
523 Clostridium puniceum GCA_002006345.1
HV
693 Clostridium beijerinckii GCA_000833105.2
671 Clostridium saccharoperbutylacetonicum N1-4_28HMT_29 GCA_000340885.1
655 Clostridium puniceum GCA_002006345.1
HW
794 Rhodobacter sphaeroides 2.4.1 GCA_000012905.2
734 Rhodobacter sphaeroides ATCC 17025 GCA_000016405.1
646 Gemmobacter aquatilis GCA_900110025.1
HX
582 Clostridium beijerinckii GCA_000833105.2
553 Clostridium saccharoperbutylacetonicum N1-4_28HMT_29 GCA_000340885.1
534 Clostridium puniceum GCA_002006345.1
HY
841 Bacillus thuringiensis YBT-1518 GCA_000497525.2
824 Bacillus cereus ATCC 14579 GCA_000007825.1

822 Bacillus anthracis str. Ames GCA_000007845.1
822 Bacillus anthracis str. Sterne GCA_000008165.1
822 _5BBacillus thuringiensis_5D serovar konkukian str. 97-27 GCA_000008505.1
HZ
671 Staphylococcus lugdunensis HKU09-01 GCA_000025085.1
665 Staphylococcus epidermidis ATCC 12228 GCA_000007645.1
631 Staphylococcus haemolyticus JCSC1435 GCA_000009865.1
631 Staphylococcus hominis subsp. hominis C80 GCA_000183685.1
I0
807 Streptococcus mutans UA159 GCA_000007465.2
569 Streptococcus ratti FA-1 = DSM 20564 GCA_000286075.1
520 Streptococcus equinus GCA_000964315.1
520 Streptococcus gordonii str. Challis substr. CH1 GCA_000017005.1
I1
766 Bifidobacterium adolescentis ATCC 15703 GCA_000010425.1
610 Bifidobacterium dentium JCM 1195 = DSM 20436 GCA_001042595.1
575 Bifidobacterium callitrichos DSM 23973 GCA_000741175.1
I2
990 Staphylococcus epidermidis ATCC 12228 GCA_000007645.1
955 Staphylococcus capitis subsp. capitis GCA_001028645.1
864 Staphylococcus warneri SG1 GCA_000332735.1
I3
732 Deinococcus radiodurans R1 GCA_000008565.1
438 Deinococcus gobiensis I-0 GCA_000252445.1
425 Deinococcus deserti VCD115 GCA_000020685.1
I4
890 Clostridium beijerinckii GCA_000833105.2
867 Clostridium saccharoperbutylacetonicum N1-4_28HMT_29 GCA_000340885.1
846 Clostridium saccharobutylicum DSM 13864 GCA_000473995.1
I5
729 Bacillus anthracis str. Ames GCA_000007845.1
729 Bacillus anthracis str. Sterne GCA_000008165.1
729 Bacillus cereus ATCC 14579 GCA_000007825.1
729 _5BBacillus thuringiensis_5D serovar konkukian str. 97-27 GCA_000008505.1
718 Bacillus pseudomycoides DSM 12442 GCA_000161455.1
716 Bacillus thuringiensis YBT-1518 GCA_000497525.2
I6
758 Clostridium beijerinckii GCA_000833105.2
742 Clostridium saccharoperbutylacetonicum N1-4_28HMT_29 GCA_000340885.1
735 Clostridium puniceum GCA_002006345.1
I7
892 Lactobacillus gasseri ATCC 33323 = JCM 1131 GCA_000014425.1
829 Lactobacillus hominis DSM 23910 = CRBIP 24.179 GCA_000296835.1
616 Lactobacillus iners DSM 13335 GCA_000160875.1
I8
666 Rhodobacter sphaeroides 2.4.1 GCA_000012905.2
597 Rhodobacter sphaeroides ATCC 17025 GCA_000016405.1
464 Pseudorhodobacter psychrotolerans GCA_001294535.1
I9
539 Lactobacillus gasseri ATCC 33323 = JCM 1131 GCA_000014425.1
494 Lactobacillus hominis DSM 23910 = CRBIP 24.179 GCA_000296835.1
404 Lactobacillus iners DSM 13335 GCA_000160875.1
Ia
786 Staphylococcus epidermidis ATCC 12228 GCA_000007645.1
745 Staphylococcus capitis subsp. capitis GCA_001028645.1
699 Staphylococcus warneri SG1 GCA_000332735.1
Ib
443 Bifidobacterium adolescentis ATCC 15703 GCA_000010425.1
297 Bifidobacterium dentium JCM 1195 = DSM 20436 GCA_001042595.1
296 Bifidobacterium pseudolongum PV8-2 GCA_000800475.2
Ic
668 Clostridium beijerinckii GCA_000833105.2
618 Clostridium saccharoperbutylacetonicum N1-4_28HMT_29 GCA_000340885.1
603 Clostridium puniceum GCA_002006345.1
Id
651 Escherichia coli O157_3AH7 str. Sakai GCA_000008865.1
651 Escherichia coli str. K-12 substr. MG1655 GCA_000005845.2
648 Escherichia coli UMN026 GCA_000026325.2
620 Escherichia coli O83_3AH1 str. NRG 857C GCA_000183345.1
Ie
592 Streptococcus mutans UA159 GCA_000007465.2
455 Streptococcus ratti FA-1 = DSM 20564 GCA_000286075.1
428 Streptococcus equinus GCA_000964315.1
If
630 Deinococcus radiodurans R1 GCA_000008565.1
415 Deinococcus deserti VCD115 GCA_000020685.1

392 Deinococcus proteolyticus MRP GCA_000190555.1
Ig
744 Streptococcus mutans UA159 GCA_000007465.2
528 Streptococcus gordonii str. Challis substr. CH1 GCA_000017005.1
527 Streptococcus ratti FA-1 = DSM 20564 GCA_000286075.1
Ih
725 Escherichia coli O104_3AH4 str. 2011C-3493 GCA_000299455.1
725 Escherichia coli str. K-12 substr. MG1655 GCA_000005845.2
714 Escherichia coli O157_3AH7 str. Sakai GCA_000008865.1
711 Escherichia coli O83_3AH1 str. NRG 857C GCA_000183345.1
Ii
749 Rhodobacter sphaeroides 2.4.1 GCA_000012905.2
673 Rhodobacter sphaeroides ATCC 17025 GCA_000016405.1
548 Gemmobacter aquatilis GCA_900110025.1
Ij
602 Escherichia coli str. K-12 substr. MG1655 GCA_000005845.2
595 Escherichia coli O104_3AH4 str. 2011C-3493 GCA_000299455.1
587 Shigella flexneri 2a str. 301 GCA_000006925.2
Ik
534 Rhodobacter sphaeroides 2.4.1 GCA_000012905.2
462 Rhodobacter sphaeroides ATCC 17025 GCA_000016405.1
395 Pseudorhodobacter ferrugineus DSM 5888 GCA_000420745.1
Il
399 Rhodobacter sphaeroides 2.4.1 GCA_000012905.2
361 Rhodobacter sphaeroides ATCC 17025 GCA_000016405.1
320 Gemmobacter aquatilis GCA_900110025.1
Im
588 Lactobacillus gasseri ATCC 33323 = JCM 1131 GCA_000014425.1
535 Lactobacillus hominis DSM 23910 = CRBIP 24.179 GCA_000296835.1
425 Lactobacillus iners DSM 13335 GCA_000160875.1
In
873 Escherichia coli str. K-12 substr. MG1655 GCA_000005845.2
872 Escherichia coli O157_3AH7 str. Sakai GCA_000008865.1
862 Escherichia coli O83_3AH1 str. NRG 857C GCA_000183345.1
Io
753 Lactobacillus gasseri ATCC 33323 = JCM 1131 GCA_000014425.1
753 Lactobacillus hominis DSM 23910 = CRBIP 24.179 GCA_000296835.1
577 Lactobacillus iners DSM 13335 GCA_000160875.1
573 Lactobacillus hamsteri DSM 5661 = JCM 6256 GCA_000615445.1
Ip
593 Rhodobacter sphaeroides 2.4.1 GCA_000012905.2
534 Rhodobacter sphaeroides ATCC 17025 GCA_000016405.1
434 Pseudorhodobacter ferrugineus DSM 5888 GCA_000420745.1
Iq
436 Enterococcus faecalis V583 GCA_000007785.1
389 Streptomyces cinnamoneus GCA_001885705.1
347 Enterococcus faecium DO GCA_000174395.2
Ir
599 Rhodobacter sphaeroides 2.4.1 GCA_000012905.2
528 Rhodobacter sphaeroides ATCC 17025 GCA_000016405.1
460 Pseudorhodobacter ferrugineus DSM 5888 GCA_000420745.1
Is
511 Escherichia coli O104_3AH4 str. 2011C-3493 GCA_000299455.1
511 Escherichia coli str. K-12 substr. MG1655 GCA_000005845.2
505 Escherichia coli IAI39 GCA_000026345.1
505 Escherichia coli UMN026 GCA_000026325.2
504 Shigella flexneri 2a str. 301 GCA_000006925.2
It
403 Clostridium beijerinckii GCA_000833105.2
378 Clostridium saccharobutylicum DSM 13864 GCA_000473995.1
378 Clostridium saccharoperbutylacetonicum N1-4_28HMT_29 GCA_000340885.1
374 Clostridium puniceum GCA_002006345.1
Iu
861 Lactobacillus gasseri ATCC 33323 = JCM 1131 GCA_000014425.1
792 Lactobacillus hominis DSM 23910 = CRBIP 24.179 GCA_000296835.1
577 Lactobacillus iners DSM 13335 GCA_000160875.1
Iv
781 Streptococcus mutans UA159 GCA_000007465.2
576 Streptococcus ratti FA-1 = DSM 20564 GCA_000286075.1
533 Streptococcus gordonii str. Challis substr. CH1 GCA_000017005.1
Iw
729 Bifidobacterium adolescentis ATCC 15703 GCA_000010425.1
647 Bifidobacterium dentium JCM 1195 = DSM 20436 GCA_001042595.1
617 Bifidobacterium angulatum DSM 20098 = JCM 7096 GCA_001025155.1
Ix
651 Staphylococcus epidermidis ATCC 12228 GCA_000007645.1

593 Staphylococcus capitis subsp. capitis GCA_001028645.1
564 Staphylococcus warneri SG1 GCA_000332735.1
Iy
801 Clostridium saccharoperbutylacetonicum N1-4_28HMT_29 GCA_000340885.1
795 Clostridium beijerinckii GCA_000833105.2
780 Clostridium saccharobutylicum DSM 13864 GCA_000473995.1
Iz
753 Clostridium beijerinckii GCA_000833105.2
734 Clostridium saccharoperbutylacetonicum N1-4_28HMT_29 GCA_000340885.1
715 Clostridium puniceum GCA_002006345.1
IA
673 Staphylococcus epidermidis ATCC 12228 GCA_000007645.1
640 Staphylococcus capitis subsp. capitis GCA_001028645.1
630 Staphylococcus lugdunensis HKU09-01 GCA_000025085.1
IB
382 Enterococcus faecalis V583 GCA_000007785.1
382 Streptomyces cinnamoneus GCA_001885705.1
367 Enterococcus canis NBRC 100695 GCA_001544375.1
367 Enterococcus hirae ATCC 9790 GCA_000271405.2
367 Enterococcus rivorum GCA_001742285.1
367 Enterococcus saccharolyticus subsp. saccharolyticus ATCC 43076 GCA_000407285.1
366 Enterococcus casseliflavus EC20 GCA_000157355.2
366 Enterococcus dispar ATCC 51266 GCA_000406945.1
366 Enterococcus gilvus ATCC BAA-350 GCA_000407545.1
366 Enterococcus malodoratus ATCC 43197 GCA_000407185.1
366 Enterococcus pallens ATCC BAA-351 GCA_000407485.1
IC
770 Rhodobacter sphaeroides 2.4.1 GCA_000012905.2
700 Rhodobacter sphaeroides ATCC 17025 GCA_000016405.1
577 Pseudorhodobacter ferrugineus DSM 5888 GCA_000420745.1
ID
529 Clostridium beijerinckii GCA_000833105.2
525 Clostridium saccharoperbutylacetonicum N1-4_28HMT_29 GCA_000340885.1
489 Clostridium puniceum GCA_002006345.1
IE
533 Clostridium beijerinckii GCA_000833105.2
502 Clostridium saccharobutylicum DSM 13864 GCA_000473995.1
500 Clostridium saccharoperbutylacetonicum N1-4_28HMT_29 GCA_000340885.1
IF
466 Lactobacillus gasseri ATCC 33323 = JCM 1131 GCA_000014425.1
442 Lactobacillus hominis DSM 23910 = CRBIP 24.179 GCA_000296835.1
340 Lactobacillus iners DSM 13335 GCA_000160875.1
IG
657 Enterococcus faecalis V583 GCA_000007785.1
611 Streptomyces cinnamoneus GCA_001885705.1
543 Enterococcus thailandicus GCA_001652875.1
IH
699 Escherichia coli O157_3AH7 str. Sakai GCA_000008865.1
699 Escherichia coli str. K-12 substr. MG1655 GCA_000005845.2
689 Escherichia coli O83_3AH1 str. NRG 857C GCA_000183345.1
688 Escherichia coli UMN026 GCA_000026325.2
II
434 Streptococcus mutans UA159 GCA_000007465.2
273 Streptococcus macacae NCTC 11558 GCA_000187995.3
272 Streptococcus ratti FA-1 = DSM 20564 GCA_000286075.1
IJ
869 Streptococcus mutans UA159 GCA_000007465.2
602 Streptococcus gordonii str. Challis substr. CH1 GCA_000017005.1
596 Streptococcus ratti FA-1 = DSM 20564 GCA_000286075.1
IK
560 Staphylococcus epidermidis ATCC 12228 GCA_000007645.1
551 Staphylococcus capitis subsp. capitis GCA_001028645.1
520 Staphylococcus simulans GCA_001559115.1
IL
830 Deinococcus radiodurans R1 GCA_000008565.1
469 Deinococcus gobiensis I-0 GCA_000252445.1
439 Deinococcus deserti VCD115 GCA_000020685.1
IM
839 Bacillus cereus ATCC 14579 GCA_000007825.1
834 Bacillus thuringiensis YBT-1518 GCA_000497525.2
833 Bacillus anthracis str. Ames GCA_000007845.1
833 Bacillus anthracis str. Sterne GCA_000008165.1
833 _5BBacillus thuringiensis_5D serovar konkukian str. 97-27 GCA_000008505.1
IN
728 Clostridium beijerinckii GCA_000833105.2
716 Clostridium saccharoperbutylacetonicum N1-4_28HMT_29 GCA_000340885.1

682 Clostridium butyricum GCA_001456065.2
IO
672 Rhodobacter sphaeroides 2.4.1 GCA_000012905.2
628 Rhodobacter sphaeroides ATCC 17025 GCA_000016405.1
528 Pseudorhodobacter ferrugineus DSM 5888 GCA_000420745.1
528 Pseudorhodobacter wandonensis GCA_001202035.1
IP
949 Staphylococcus epidermidis ATCC 12228 GCA_000007645.1
903 Staphylococcus capitis subsp. capitis GCA_001028645.1
846 Staphylococcus haemolyticus JCSC1435 GCA_000009865.1
IQ
830 Streptococcus mutans UA159 GCA_000007465.2
628 Streptococcus ratti FA-1 = DSM 20564 GCA_000286075.1
613 Streptococcus ferus DSM 20646 GCA_000372425.1
IR
821 Clostridium beijerinckii GCA_000833105.2
800 Clostridium saccharobutylicum DSM 13864 GCA_000473995.1
796 Clostridium saccharoperbutylacetonicum N1-4_28HMT_29 GCA_000340885.1
IS
672 Clostridium beijerinckii GCA_000833105.2
642 Clostridium saccharobutylicum DSM 13864 GCA_000473995.1
633 Clostridium saccharoperbutylacetonicum N1-4_28HMT_29 GCA_000340885.1
IT
410 Rhodobacter sphaeroides 2.4.1 GCA_000012905.2
374 Rhodobacter sphaeroides ATCC 17025 GCA_000016405.1
293 Gemmobacter aquatilis GCA_900110025.1
293 Pseudorhodobacter psychrotolerans GCA_001294535.1
IU
607 Lactobacillus gasseri ATCC 33323 = JCM 1131 GCA_000014425.1
541 Lactobacillus hominis DSM 23910 = CRBIP 24.179 GCA_000296835.1
396 Lactobacillus iners DSM 13335 GCA_000160875.1
IV
765 Enterococcus faecalis V583 GCA_000007785.1
724 Streptomyces cinnamoneus GCA_001885705.1
704 Enterococcus rivorum GCA_001742285.1
IW
724 Bacillus thuringiensis YBT-1518 GCA_000497525.2
713 Bacillus anthracis str. Ames GCA_000007845.1
713 Bacillus anthracis str. Sterne GCA_000008165.1
713 _5BBacillus thuringiensis_5D serovar konkukian str. 97-27 GCA_000008505.1
707 Bacillus cereus ATCC 14579 GCA_000007825.1
IX
701 Deinococcus radiodurans R1 GCA_000008565.1
397 Deinococcus deserti VCD115 GCA_000020685.1
372 Deinococcus hopiensis KR-140 GCA_900176165.1
IY
548 Lactobacillus gasseri ATCC 33323 = JCM 1131 GCA_000014425.1
525 Lactobacillus hominis DSM 23910 = CRBIP 24.179 GCA_000296835.1
451 Lactobacillus iners DSM 13335 GCA_000160875.1
IZ
135 Rhodobacter sphaeroides 2.4.1 GCA_000012905.2
119 Rhodobacter sphaeroides ATCC 17025 GCA_000016405.1
85 Pseudorhodobacter psychrotolerans GCA_001294535.1
85 Pseudorhodobacter wandonensis GCA_001202035.1
J0
830 _5BBacillus thuringiensis_5D serovar konkukian str. 97-27 GCA_000008505.1
829 Bacillus anthracis str. Ames GCA_000007845.1
829 Bacillus anthracis str. Sterne GCA_000008165.1
821 Bacillus cereus ATCC 14579 GCA_000007825.1
J1
802 Streptococcus mutans UA159 GCA_000007465.2
573 Streptococcus ratti FA-1 = DSM 20564 GCA_000286075.1
561 Streptococcus gordonii str. Challis substr. CH1 GCA_000017005.1
J2
779 Deinococcus radiodurans R1 GCA_000008565.1
527 Deinococcus deserti VCD115 GCA_000020685.1
479 Deinococcus gobiensis I-0 GCA_000252445.1
J3
772 Clostridium beijerinckii GCA_000833105.2
735 Clostridium saccharoperbutylacetonicum N1-4_28HMT_29 GCA_000340885.1
726 Clostridium saccharobutylicum DSM 13864 GCA_000473995.1
J4
818 Lactobacillus gasseri ATCC 33323 = JCM 1131 GCA_000014425.1
737 Lactobacillus hominis DSM 23910 = CRBIP 24.179 GCA_000296835.1
544 Lactobacillus psittaci DSM 15354 GCA_000425905.1
J5

614 Staphylococcus epidermidis ATCC 12228 GCA_000007645.1
564 Staphylococcus capitis subsp. capitis GCA_001028645.1
534 Staphylococcus lugdunensis HKU09-01 GCA_000025085.1
J6
699 Clostridium beijerinckii GCA_000833105.2
655 Clostridium saccharoperbutylacetonicum N1-4_28HMT_29 GCA_000340885.1
640 Clostridium puniceum GCA_002006345.1
J7
525 Deinococcus radiodurans R1 GCA_000008565.1
281 Deinococcus hopiensis KR-140 GCA_900176165.1
277 Deinococcus gobiensis I-0 GCA_000252445.1
J8
683 Lactobacillus gasseri ATCC 33323 = JCM 1131 GCA_000014425.1
635 Lactobacillus hominis DSM 23910 = CRBIP 24.179 GCA_000296835.1
487 Lactobacillus iners DSM 13335 GCA_000160875.1
J9
519 Streptococcus mutans UA159 GCA_000007465.2
355 Streptococcus gordonii str. Challis substr. CH1 GCA_000017005.1
353 Streptococcus iniae GCA_000831485.1
Ja
596 Clostridium beijerinckii GCA_000833105.2
562 Clostridium saccharoperbutylacetonicum N1-4_28HMT_29 GCA_000340885.1
530 Clostridium puniceum GCA_002006345.1
Jb
871 Streptococcus mutans UA159 GCA_000007465.2
654 Streptococcus ratti FA-1 = DSM 20564 GCA_000286075.1
653 Streptococcus gordonii str. Challis substr. CH1 GCA_000017005.1
Jc
776 Deinococcus radiodurans R1 GCA_000008565.1
478 Deinococcus deserti VCD115 GCA_000020685.1
416 Deinococcus soli Cha et al. 2016 GCA_001007995.1
Jd
330 Rhodobacter sphaeroides 2.4.1 GCA_000012905.2
309 Rhodobacter sphaeroides ATCC 17025 GCA_000016405.1
227 Gemmobacter aquatilis GCA_900110025.1
Je
582 Streptococcus mutans UA159 GCA_000007465.2
431 Streptococcus ratti FA-1 = DSM 20564 GCA_000286075.1
372 Streptococcus sobrinus DSM 20742 = ATCC 33478 GCA_000686605.1
Jf
673 Deinococcus radiodurans R1 GCA_000008565.1
415 Deinococcus deserti VCD115 GCA_000020685.1
398 Deinococcus gobiensis I-0 GCA_000252445.1
Jg
698 Clostridium beijerinckii GCA_000833105.2
698 Clostridium saccharoperbutylacetonicum N1-4_28HMT_29 GCA_000340885.1
678 Clostridium puniceum GCA_002006345.1
675 Clostridium saccharobutylicum DSM 13864 GCA_000473995.1
Jh
639 Staphylococcus epidermidis ATCC 12228 GCA_000007645.1
612 Staphylococcus capitis subsp. capitis GCA_001028645.1
587 Staphylococcus aureus subsp. aureus NCTC 8325 GCA_000013425.1
Ji
542 Lactobacillus gasseri ATCC 33323 = JCM 1131 GCA_000014425.1
471 Lactobacillus hominis DSM 23910 = CRBIP 24.179 GCA_000296835.1
312 Lactobacillus iners DSM 13335 GCA_000160875.1
Jj
428 Bacillus thuringiensis YBT-1518 GCA_000497525.2
412 Bacillus anthracis str. Ames GCA_000007845.1
412 Bacillus anthracis str. Sterne GCA_000008165.1
412 Bacillus cereus ATCC 14579 GCA_000007825.1
412 _5BBacillus thuringiensis_5D serovar konkukian str. 97-27 GCA_000008505.1
403 Bacillus pseudomycoides DSM 12442 GCA_000161455.1
Jk
800 Lactobacillus gasseri ATCC 33323 = JCM 1131 GCA_000014425.1
772 Lactobacillus hominis DSM 23910 = CRBIP 24.179 GCA_000296835.1
647 Lactobacillus iners DSM 13335 GCA_000160875.1
Jl
631 Bacillus anthracis str. Ames GCA_000007845.1
631 Bacillus anthracis str. Sterne GCA_000008165.1
631 _5BBacillus thuringiensis_5D serovar konkukian str. 97-27 GCA_000008505.1
611 Bacillus cereus ATCC 14579 GCA_000007825.1
611 Bacillus thuringiensis YBT-1518 GCA_000497525.2
590 Bacillus pseudomycoides DSM 12442 GCA_000161455.1
Jm
653 Bifidobacterium adolescentis ATCC 15703 GCA_000010425.1

516 Bifidobacterium dentium JCM 1195 = DSM 20436 GCA_001042595.1
504 Bifidobacterium angulatum DSM 20098 = JCM 7096 GCA_001025155.1
Jn
720 Clostridium beijerinckii GCA_000833105.2
702 Clostridium saccharoperbutylacetonicum N1-4_28HMT_29 GCA_000340885.1
691 Clostridium saccharobutylicum DSM 13864 GCA_000473995.1
Jo
578 Rhodobacter sphaeroides 2.4.1 GCA_000012905.2
490 Rhodobacter sphaeroides ATCC 17025 GCA_000016405.1
445 Defluviimonas alba GCA_001620265.1
Jp
694 Enterococcus faecalis V583 GCA_000007785.1
648 Streptomyces cinnamoneus GCA_001885705.1
643 Enterococcus faecium DO GCA_000174395.2
Jq
534 Clostridium beijerinckii GCA_000833105.2
483 Clostridium saccharoperbutylacetonicum N1-4_28HMT_29 GCA_000340885.1
461 Clostridium puniceum GCA_002006345.1
Jr
809 Bacillus anthracis str. Ames GCA_000007845.1
809 Bacillus anthracis str. Sterne GCA_000008165.1
809 Bacillus cereus ATCC 14579 GCA_000007825.1
809 _5BBacillus thuringiensis_5D serovar konkukian str. 97-27 GCA_000008505.1
794 Bacillus pseudomycoides DSM 12442 GCA_000161455.1
794 Bacillus thuringiensis YBT-1518 GCA_000497525.2
756 Bacillus mycoides GCA_000832605.1
Js
590 Lactobacillus gasseri ATCC 33323 = JCM 1131 GCA_000014425.1
573 Lactobacillus hominis DSM 23910 = CRBIP 24.179 GCA_000296835.1
448 Lactobacillus psittaci DSM 15354 GCA_000425905.1
Jt
626 Bifidobacterium adolescentis ATCC 15703 GCA_000010425.1
494 Bifidobacterium dentium JCM 1195 = DSM 20436 GCA_001042595.1
473 Bifidobacterium callitrichos DSM 23973 GCA_000741175.1
Ju
556 Rhodobacter sphaeroides 2.4.1 GCA_000012905.2
513 Rhodobacter sphaeroides ATCC 17025 GCA_000016405.1
401 Pseudorhodobacter ferrugineus DSM 5888 GCA_000420745.1
Jv
476 Bacillus thuringiensis YBT-1518 GCA_000497525.2
461 Bacillus anthracis str. Ames GCA_000007845.1
461 Bacillus anthracis str. Sterne GCA_000008165.1
461 Bacillus cereus ATCC 14579 GCA_000007825.1
461 _5BBacillus thuringiensis_5D serovar konkukian str. 97-27 GCA_000008505.1
431 Bacillus pseudomycoides DSM 12442 GCA_000161455.1
Jw
788 Streptococcus mutans UA159 GCA_000007465.2
570 Streptococcus ratti FA-1 = DSM 20564 GCA_000286075.1
555 Streptococcus equinus GCA_000964315.1
Jx
583 Bacillus thuringiensis YBT-1518 GCA_000497525.2
570 Bacillus anthracis str. Ames GCA_000007845.1
570 Bacillus anthracis str. Sterne GCA_000008165.1
570 Bacillus cereus ATCC 14579 GCA_000007825.1
570 Bacillus pseudomycoides DSM 12442 GCA_000161455.1
570 _5BBacillus thuringiensis_5D serovar konkukian str. 97-27 GCA_000008505.1
523 Bacillus cytotoxicus NVH 391-98 GCA_000017425.1
Jy
827 Enterococcus faecalis V583 GCA_000007785.1
784 Streptomyces cinnamoneus GCA_001885705.1
744 Enterococcus rivorum GCA_001742285.1
Jz
690 Bifidobacterium adolescentis ATCC 15703 GCA_000010425.1
572 Bifidobacterium dentium JCM 1195 = DSM 20436 GCA_001042595.1
535 Bifidobacterium angulatum DSM 20098 = JCM 7096 GCA_001025155.1
JA
699 Clostridium beijerinckii GCA_000833105.2
669 Clostridium saccharoperbutylacetonicum N1-4_28HMT_29 GCA_000340885.1
644 Clostridium puniceum GCA_002006345.1
JB
588 Clostridium beijerinckii GCA_000833105.2
569 Clostridium saccharobutylicum DSM 13864 GCA_000473995.1
569 Clostridium saccharoperbutylacetonicum N1-4_28HMT_29 GCA_000340885.1
543 Clostridium puniceum GCA_002006345.1
JC
372 Clostridium beijerinckii GCA_000833105.2

338 Clostridium saccharoperbutylacetonicum N1-4_28HMT_29 GCA_000340885.1
336 Clostridium saccharobutylicum DSM 13864 GCA_000473995.1
JD
647 Streptococcus mutans UA159 GCA_000007465.2
450 Streptococcus ratti FA-1 = DSM 20564 GCA_000286075.1
440 Streptococcus gordonii str. Challis substr. CH1 GCA_000017005.1
JE
672 Deinococcus radiodurans R1 GCA_000008565.1
425 Deinococcus deserti VCD115 GCA_000020685.1
379 Deinococcus hopiensis KR-140 GCA_900176165.1
JF
701 Bacillus anthracis str. Ames GCA_000007845.1
701 Bacillus anthracis str. Sterne GCA_000008165.1
701 _5BBacillus thuringiensis_5D serovar konkukian str. 97-27 GCA_000008505.1
691 Bacillus cereus ATCC 14579 GCA_000007825.1
691 Bacillus thuringiensis YBT-1518 GCA_000497525.2
643 Bacillus pseudomycoides DSM 12442 GCA_000161455.1
JG
806 Deinococcus radiodurans R1 GCA_000008565.1
520 Deinococcus deserti VCD115 GCA_000020685.1
501 Deinococcus hopiensis KR-140 GCA_900176165.1
JH
541 Lactobacillus gasseri ATCC 33323 = JCM 1131 GCA_000014425.1
541 Lactobacillus hominis DSM 23910 = CRBIP 24.179 GCA_000296835.1
433 Lactobacillus iners DSM 13335 GCA_000160875.1
354 Lactobacillus farraginis DSM 18382 = JCM 14108 GCA_000583655.1
JI
502 Staphylococcus epidermidis ATCC 12228 GCA_000007645.1
493 Staphylococcus capitis subsp. capitis GCA_001028645.1
457 Staphylococcus aureus subsp. aureus NCTC 8325 GCA_000013425.1
JJ
672 Clostridium saccharoperbutylacetonicum N1-4_28HMT_29 GCA_000340885.1
671 Clostridium beijerinckii GCA_000833105.2
665 Clostridium saccharobutylicum DSM 13864 GCA_000473995.1
JK
798 Lactobacillus gasseri ATCC 33323 = JCM 1131 GCA_000014425.1
766 Lactobacillus hominis DSM 23910 = CRBIP 24.179 GCA_000296835.1
595 Lactobacillus iners DSM 13335 GCA_000160875.1
JL
524 Rhodobacter sphaeroides 2.4.1 GCA_000012905.2
466 Rhodobacter sphaeroides ATCC 17025 GCA_000016405.1
381 Defluviimonas alba GCA_001620265.1
JM
355 Rhodobacter sphaeroides 2.4.1 GCA_000012905.2
295 Rhodobacter sphaeroides ATCC 17025 GCA_000016405.1
274 Gemmobacter megaterium GCA_900156815.1
JN
663 Clostridium beijerinckii GCA_000833105.2
638 Clostridium saccharoperbutylacetonicum N1-4_28HMT_29 GCA_000340885.1
630 Clostridium puniceum GCA_002006345.1
JO
611 Enterococcus faecalis V583 GCA_000007785.1
564 Streptomyces cinnamoneus GCA_001885705.1
537 Enterococcus rivorum GCA_001742285.1
JP
805 Rhodobacter sphaeroides 2.4.1 GCA_000012905.2
714 Rhodobacter sphaeroides ATCC 17025 GCA_000016405.1
623 Pseudorhodobacter psychrotolerans GCA_001294535.1
JQ
530 Clostridium beijerinckii GCA_000833105.2
485 Clostridium saccharoperbutylacetonicum N1-4_28HMT_29 GCA_000340885.1
478 Clostridium puniceum GCA_002006345.1
JR
872 Lactobacillus gasseri ATCC 33323 = JCM 1131 GCA_000014425.1
796 Lactobacillus hominis DSM 23910 = CRBIP 24.179 GCA_000296835.1
598 Lactobacillus iners DSM 13335 GCA_000160875.1
JS
549 Clostridium beijerinckii GCA_000833105.2
519 Clostridium butyricum GCA_001456065.2
519 Clostridium puniceum GCA_002006345.1
519 Clostridium saccharoperbutylacetonicum N1-4_28HMT_29 GCA_000340885.1
492 Clostridium saccharobutylicum DSM 13864 GCA_000473995.1
JT
535 Enterococcus faecalis V583 GCA_000007785.1
488 Streptomyces cinnamoneus GCA_001885705.1
472 Enterococcus asini ATCC 700915 GCA_000407365.1

JU
530 Bacillus anthracis str. Ames GCA_000007845.1
530 Bacillus anthracis str. Sterne GCA_000008165.1
530 _5BBacillus thuringiensis_5D serovar konkukian str. 97-27 GCA_000008505.1
509 Bacillus cereus ATCC 14579 GCA_000007825.1
504 Bacillus pseudomycoides DSM 12442 GCA_000161455.1
JV
771 Clostridium beijerinckii GCA_000833105.2
707 Clostridium saccharoperbutylacetonicum N1-4_28HMT_29 GCA_000340885.1
679 Clostridium saccharobutylicum DSM 13864 GCA_000473995.1
JW
640 Clostridium beijerinckii GCA_000833105.2
626 Clostridium saccharoperbutylacetonicum N1-4_28HMT_29 GCA_000340885.1
601 Clostridium puniceum GCA_002006345.1
JX
805 Lactobacillus gasseri ATCC 33323 = JCM 1131 GCA_000014425.1
777 Lactobacillus hominis DSM 23910 = CRBIP 24.179 GCA_000296835.1
600 Lactobacillus iners DSM 13335 GCA_000160875.1
JY
539 Deinococcus radiodurans R1 GCA_000008565.1
308 Deinococcus deserti VCD115 GCA_000020685.1
280 Deinococcus soli Cha et al. 2016 GCA_001007995.1
JZ
589 Rhodobacter sphaeroides 2.4.1 GCA_000012905.2
497 Rhodobacter sphaeroides ATCC 17025 GCA_000016405.1
435 Gemmobacter aquatilis GCA_900110025.1
K0
888 Bacillus anthracis str. Ames GCA_000007845.1
888 Bacillus anthracis str. Sterne GCA_000008165.1
888 _5BBacillus thuringiensis_5D serovar konkukian str. 97-27 GCA_000008505.1
877 Bacillus cereus ATCC 14579 GCA_000007825.1
863 Bacillus thuringiensis YBT-1518 GCA_000497525.2
K1
359 Clostridium beijerinckii GCA_000833105.2
359 Clostridium saccharoperbutylacetonicum N1-4_28HMT_29 GCA_000340885.1
352 Clostridium saccharobutylicum DSM 13864 GCA_000473995.1
302 Clostridium butyricum GCA_001456065.2
K2
675 Bifidobacterium adolescentis ATCC 15703 GCA_000010425.1
527 Bifidobacterium dentium JCM 1195 = DSM 20436 GCA_001042595.1
507 Bifidobacterium breve DSM 20213 = JCM 1192 GCA_001025175.1
507 Bifidobacterium reuteri DSM 23975 GCA_000741695.1
K3
794 Lactobacillus gasseri ATCC 33323 = JCM 1131 GCA_000014425.1
736 Lactobacillus hominis DSM 23910 = CRBIP 24.179 GCA_000296835.1
499 Lactobacillus iners DSM 13335 GCA_000160875.1
K4
717 Clostridium beijerinckii GCA_000833105.2
696 Clostridium saccharoperbutylacetonicum N1-4_28HMT_29 GCA_000340885.1
646 Clostridium saccharobutylicum DSM 13864 GCA_000473995.1
K5
419 Rhodobacter sphaeroides 2.4.1 GCA_000012905.2
376 Rhodobacter sphaeroides ATCC 17025 GCA_000016405.1
364 Gemmobacter megaterium GCA_900156815.1
K6
472 Bacillus anthracis str. Ames GCA_000007845.1
472 Bacillus anthracis str. Sterne GCA_000008165.1
472 Bacillus pseudomycoides DSM 12442 GCA_000161455.1
472 _5BBacillus thuringiensis_5D serovar konkukian str. 97-27 GCA_000008505.1
456 Bacillus cereus ATCC 14579 GCA_000007825.1
456 Bacillus mycoides GCA_000832605.1
442 Bacillus cytotoxicus NVH 391-98 GCA_000017425.1
K7
922 Rhodobacter sphaeroides 2.4.1 GCA_000012905.2
872 Rhodobacter sphaeroides ATCC 17025 GCA_000016405.1
713 Pseudorhodobacter psychrotolerans GCA_001294535.1
K8
460 Staphylococcus epidermidis ATCC 12228 GCA_000007645.1
457 Staphylococcus capitis subsp. capitis GCA_001028645.1
418 Staphylococcus warneri SG1 GCA_000332735.1
K9
830 Clostridium beijerinckii GCA_000833105.2
796 Clostridium saccharoperbutylacetonicum N1-4_28HMT_29 GCA_000340885.1
758 Clostridium puniceum GCA_002006345.1
Ka
793 Deinococcus radiodurans R1 GCA_000008565.1

517 Deinococcus deserti VCD115 GCA_000020685.1
487 Deinococcus murrayi DSM 11303 GCA_000482805.1
Kb
760 Rhodobacter sphaeroides 2.4.1 GCA_000012905.2
713 Rhodobacter sphaeroides ATCC 17025 GCA_000016405.1
616 Gemmobacter aquatilis GCA_900110025.1
Kc
741 Deinococcus radiodurans R1 GCA_000008565.1
523 Deinococcus soli Cha et al. 2016 GCA_001007995.1
505 Deinococcus deserti VCD115 GCA_000020685.1
Kd
648 Streptococcus mutans UA159 GCA_000007465.2
448 Streptococcus ratti FA-1 = DSM 20564 GCA_000286075.1
446 Streptococcus equinus GCA_000964315.1
Ke
722 Streptococcus mutans UA159 GCA_000007465.2
497 Streptococcus equinus GCA_000964315.1
497 Streptococcus ratti FA-1 = DSM 20564 GCA_000286075.1
496 Streptococcus gallolyticus subsp. gallolyticus DSM 16831 GCA_002000985.1
496 Streptococcus gordonii str. Challis substr. CH1 GCA_000017005.1
Kf
537 Staphylococcus epidermidis ATCC 12228 GCA_000007645.1
490 Staphylococcus capitis subsp. capitis GCA_001028645.1
469 Staphylococcus haemolyticus JCSC1435 GCA_000009865.1
Kg
809 Streptococcus mutans UA159 GCA_000007465.2
612 Streptococcus ratti FA-1 = DSM 20564 GCA_000286075.1
569 Streptococcus gordonii str. Challis substr. CH1 GCA_000017005.1
Kh
715 Deinococcus radiodurans R1 GCA_000008565.1
492 Deinococcus soli Cha et al. 2016 GCA_001007995.1
483 Deinococcus deserti VCD115 GCA_000020685.1
Ki
809 Enterococcus faecalis V583 GCA_000007785.1
768 Streptomyces cinnamoneus GCA_001885705.1
708 Enterococcus saccharolyticus subsp. saccharolyticus ATCC 43076 GCA_000407285.1
Kj
577 Deinococcus radiodurans R1 GCA_000008565.1
377 Deinococcus deserti VCD115 GCA_000020685.1
368 Deinococcus proteolyticus MRP GCA_000190555.1
Kk
467 Bifidobacterium adolescentis ATCC 15703 GCA_000010425.1
403 Bifidobacterium dentium JCM 1195 = DSM 20436 GCA_001042595.1
383 Bifidobacterium bifidum PRL2010 GCA_000165905.1
Kl
693 Bacillus thuringiensis YBT-1518 GCA_000497525.2
685 Bacillus anthracis str. Ames GCA_000007845.1
685 Bacillus anthracis str. Sterne GCA_000008165.1
685 _5BBacillus thuringiensis_5D serovar konkukian str. 97-27 GCA_000008505.1
679 Bacillus cereus ATCC 14579 GCA_000007825.1
Km
635 Lactobacillus gasseri ATCC 33323 = JCM 1131 GCA_000014425.1
605 Lactobacillus hominis DSM 23910 = CRBIP 24.179 GCA_000296835.1
450 Lactobacillus iners DSM 13335 GCA_000160875.1
Kn
592 Deinococcus radiodurans R1 GCA_000008565.1
347 Deinococcus deserti VCD115 GCA_000020685.1
321 Deinococcus gobiensis I-0 GCA_000252445.1
Ko
868 Rhodobacter sphaeroides 2.4.1 GCA_000012905.2
773 Rhodobacter sphaeroides ATCC 17025 GCA_000016405.1
633 Gemmobacter megaterium GCA_900156815.1
Kp
826 Clostridium beijerinckii GCA_000833105.2
796 Clostridium saccharoperbutylacetonicum N1-4_28HMT_29 GCA_000340885.1
758 Clostridium saccharobutylicum DSM 13864 GCA_000473995.1
Kq
642 Bifidobacterium adolescentis ATCC 15703 GCA_000010425.1
544 Bifidobacterium dentium JCM 1195 = DSM 20436 GCA_001042595.1
490 Bifidobacterium stellenboschense GCA_000741785.1
Kr
575 Lactobacillus gasseri ATCC 33323 = JCM 1131 GCA_000014425.1
476 Lactobacillus hominis DSM 23910 = CRBIP 24.179 GCA_000296835.1
322 Lactobacillus iners DSM 13335 GCA_000160875.1
Ks
742 Streptococcus mutans UA159 GCA_000007465.2

555 Streptococcus ratti FA-1 = DSM 20564 GCA_000286075.1
524 Streptococcus ferus DSM 20646 GCA_000372425.1
Kt
735 Lactobacillus gasseri ATCC 33323 = JCM 1131 GCA_000014425.1
703 Lactobacillus hominis DSM 23910 = CRBIP 24.179 GCA_000296835.1
577 Lactobacillus psittaci DSM 15354 GCA_000425905.1
Ku
702 Bacillus anthracis str. Ames GCA_000007845.1
702 Bacillus anthracis str. Sterne GCA_000008165.1
702 _5BBacillus thuringiensis_5D serovar konkukian str. 97-27 GCA_000008505.1
689 Bacillus cereus ATCC 14579 GCA_000007825.1
689 Bacillus thuringiensis YBT-1518 GCA_000497525.2
676 Bacillus pseudomycoides DSM 12442 GCA_000161455.1
Kv
760 Staphylococcus capitis subsp. capitis GCA_001028645.1
760 Staphylococcus epidermidis ATCC 12228 GCA_000007645.1
677 Staphylococcus aureus subsp. aureus NCTC 8325 GCA_000013425.1
667 Staphylococcus haemolyticus JCSC1435 GCA_000009865.1
Kw
509 Clostridium beijerinckii GCA_000833105.2
490 Clostridium butyricum GCA_001456065.2
481 Clostridium saccharobutylicum DSM 13864 GCA_000473995.1
Kx
645 Clostridium beijerinckii GCA_000833105.2
609 Clostridium saccharoperbutylacetonicum N1-4_28HMT_29 GCA_000340885.1
582 Clostridium puniceum GCA_002006345.1
Ky
805 Clostridium beijerinckii GCA_000833105.2
764 Clostridium saccharoperbutylacetonicum N1-4_28HMT_29 GCA_000340885.1
744 Clostridium puniceum GCA_002006345.1
Kz
638 Lactobacillus gasseri ATCC 33323 = JCM 1131 GCA_000014425.1
560 Lactobacillus hominis DSM 23910 = CRBIP 24.179 GCA_000296835.1
481 Lactobacillus iners DSM 13335 GCA_000160875.1
KA
753 Clostridium beijerinckii GCA_000833105.2
693 Clostridium saccharoperbutylacetonicum N1-4_28HMT_29 GCA_000340885.1
663 Clostridium saccharobutylicum DSM 13864 GCA_000473995.1
KB
361 Staphylococcus epidermidis ATCC 12228 GCA_000007645.1
356 Staphylococcus aureus subsp. aureus NCTC 8325 GCA_000013425.1
352 Staphylococcus capitis subsp. capitis GCA_001028645.1
KC
547 Clostridium beijerinckii GCA_000833105.2
532 Clostridium butyricum GCA_001456065.2
528 Clostridium puniceum GCA_002006345.1
528 Clostridium saccharoperbutylacetonicum N1-4_28HMT_29 GCA_000340885.1
KD
907 Enterococcus faecalis V583 GCA_000007785.1
861 Streptomyces cinnamoneus GCA_001885705.1
799 Enterococcus rivorum GCA_001742285.1
KE
768 Rhodobacter sphaeroides 2.4.1 GCA_000012905.2
675 Rhodobacter sphaeroides ATCC 17025 GCA_000016405.1
595 Defluviimonas alba GCA_001620265.1
KF
736 Staphylococcus epidermidis ATCC 12228 GCA_000007645.1
694 Staphylococcus capitis subsp. capitis GCA_001028645.1
641 Staphylococcus warneri SG1 GCA_000332735.1
KG
509 Enterococcus faecalis V583 GCA_000007785.1
490 Enterococcus saccharolyticus subsp. saccharolyticus ATCC 43076 GCA_000407285.1
477 Enterococcus sulfureus ATCC 49903 GCA_000407605.1
KH
843 Deinococcus radiodurans R1 GCA_000008565.1
507 Deinococcus gobiensis I-0 GCA_000252445.1
485 Deinococcus deserti VCD115 GCA_000020685.1
KI
692 Escherichia coli O157_3AH7 str. Sakai GCA_000008865.1
692 Escherichia coli str. K-12 substr. MG1655 GCA_000005845.2
691 Escherichia coli UMN026 GCA_000026325.2
677 Shigella flexneri 2a str. 301 GCA_000006925.2
KJ
638 Rhodobacter sphaeroides 2.4.1 GCA_000012905.2
580 Rhodobacter sphaeroides ATCC 17025 GCA_000016405.1
490 Defluviimonas alba GCA_001620265.1

KK
689 Clostridium beijerinckii GCA_000833105.2
621 Clostridium chromiireducens GCA_002029255.1
611 Clostridium saccharoperbutylacetonicum N1-4_28HMT_29 GCA_000340885.1
KL
269 Lactobacillus gasseri ATCC 33323 = JCM 1131 GCA_000014425.1
240 Lactobacillus hominis DSM 23910 = CRBIP 24.179 GCA_000296835.1
166 Lactobacillus acetotolerans GCA_001042405.1
KM
700 Deinococcus radiodurans R1 GCA_000008565.1
464 Deinococcus deserti VCD115 GCA_000020685.1
425 Deinococcus soli Cha et al. 2016 GCA_001007995.1
KN
801 Streptococcus mutans UA159 GCA_000007465.2
661 Streptococcus ratti FA-1 = DSM 20564 GCA_000286075.1
616 Streptococcus criceti HS-6 GCA_000187975.3
616 Streptococcus gordonii str. Challis substr. CH1 GCA_000017005.1
KO
584 Enterococcus faecalis V583 GCA_000007785.1
555 Streptomyces cinnamoneus GCA_001885705.1
523 Enterococcus rivorum GCA_001742285.1
KP
828 Enterococcus faecalis V583 GCA_000007785.1
781 Streptomyces cinnamoneus GCA_001885705.1
768 Enterococcus faecium DO GCA_000174395.2
KQ
409 Streptococcus mutans UA159 GCA_000007465.2
266 Streptococcus macacae NCTC 11558 GCA_000187995.3
255 Streptococcus ratti FA-1 = DSM 20564 GCA_000286075.1
KR
751 _5BBacillus thuringiensis_5D serovar konkukian str. 97-27 GCA_000008505.1
749 Bacillus anthracis str. Ames GCA_000007845.1
749 Bacillus anthracis str. Sterne GCA_000008165.1
737 Bacillus cereus ATCC 14579 GCA_000007825.1
KS
465 Lactobacillus gasseri ATCC 33323 = JCM 1131 GCA_000014425.1
407 Lactobacillus hominis DSM 23910 = CRBIP 24.179 GCA_000296835.1
350 Lactobacillus iners DSM 13335 GCA_000160875.1
KT
445 Rhodobacter sphaeroides 2.4.1 GCA_000012905.2
371 Rhodobacter sphaeroides ATCC 17025 GCA_000016405.1
317 Gemmobacter megaterium GCA_900156815.1
KU
427 Rhodobacter sphaeroides 2.4.1 GCA_000012905.2
408 Rhodobacter sphaeroides ATCC 17025 GCA_000016405.1
317 Gemmobacter nectariphilus DSM 15620 GCA_000429765.1
317 Pseudorhodobacter psychrotolerans GCA_001294535.1
KV
638 Lactobacillus gasseri ATCC 33323 = JCM 1131 GCA_000014425.1
587 Lactobacillus hominis DSM 23910 = CRBIP 24.179 GCA_000296835.1
433 Lactobacillus iners DSM 13335 GCA_000160875.1
KW
596 Streptococcus mutans UA159 GCA_000007465.2
447 Streptococcus ratti FA-1 = DSM 20564 GCA_000286075.1
402 Streptococcus ferus DSM 20646 GCA_000372425.1
KX
647 Clostridium beijerinckii GCA_000833105.2
632 Clostridium saccharoperbutylacetonicum N1-4_28HMT_29 GCA_000340885.1
619 Clostridium saccharobutylicum DSM 13864 GCA_000473995.1
KY
948 Streptococcus mutans UA159 GCA_000007465.2
620 Streptococcus gordonii str. Challis substr. CH1 GCA_000017005.1
614 Streptococcus ratti FA-1 = DSM 20564 GCA_000286075.1
KZ
813 Deinococcus radiodurans R1 GCA_000008565.1
472 Deinococcus deserti VCD115 GCA_000020685.1
456 Deinococcus gobiensis I-0 GCA_000252445.1
L0
981 Lactobacillus gasseri ATCC 33323 = JCM 1131 GCA_000014425.1
923 Lactobacillus hominis DSM 23910 = CRBIP 24.179 GCA_000296835.1
728 Lactobacillus iners DSM 13335 GCA_000160875.1
L1
512 Lactobacillus gasseri ATCC 33323 = JCM 1131 GCA_000014425.1
434 Lactobacillus hominis DSM 23910 = CRBIP 24.179 GCA_000296835.1
367 Lactobacillus iners DSM 13335 GCA_000160875.1
L2

660 Rhodobacter sphaeroides 2.4.1 GCA_000012905.2
571 Rhodobacter sphaeroides ATCC 17025 GCA_000016405.1
461 Gemmobacter aquatilis GCA_900110025.1
L3
479 Rhodobacter sphaeroides 2.4.1 GCA_000012905.2
468 Rhodobacter sphaeroides ATCC 17025 GCA_000016405.1
360 Pseudorhodobacter wandonensis GCA_001202035.1
L4
835 Staphylococcus epidermidis ATCC 12228 GCA_000007645.1
833 Staphylococcus capitis subsp. capitis GCA_001028645.1
775 Staphylococcus aureus subsp. aureus NCTC 8325 GCA_000013425.1
L5
614 Bacillus anthracis str. Ames GCA_000007845.1
614 Bacillus anthracis str. Sterne GCA_000008165.1
614 Bacillus cereus ATCC 14579 GCA_000007825.1
614 _5BBacillus thuringiensis_5D serovar konkukian str. 97-27 GCA_000008505.1
608 Bacillus thuringiensis YBT-1518 GCA_000497525.2
607 Bacillus mycoides GCA_000832605.1
L6
656 Deinococcus radiodurans R1 GCA_000008565.1
439 Deinococcus puniceus GCA_001644565.1
428 Deinococcus hopiensis KR-140 GCA_900176165.1
L7
804 Enterococcus faecalis V583 GCA_000007785.1
757 Streptomyces cinnamoneus GCA_001885705.1
714 Enterococcus asini ATCC 700915 GCA_000407365.1
714 Enterococcus malodoratus ATCC 43197 GCA_000407185.1
L8
513 Rhodobacter sphaeroides 2.4.1 GCA_000012905.2
495 Rhodobacter sphaeroides ATCC 17025 GCA_000016405.1
449 Gemmobacter aquatilis GCA_900110025.1
L9
365 Enterococcus faecalis V583 GCA_000007785.1
318 Streptomyces cinnamoneus GCA_001885705.1
305 Enterococcus dispar ATCC 51266 GCA_000406945.1
305 Enterococcus phoeniculicola ATCC BAA-412 GCA_000407505.1
La
558 Escherichia coli str. K-12 substr. MG1655 GCA_000005845.2
547 Escherichia coli IAI39 GCA_000026345.1
547 Escherichia coli O104_3AH4 str. 2011C-3493 GCA_000299455.1
547 Shigella flexneri 2a str. 301 GCA_000006925.2
546 Escherichia coli UMN026 GCA_000026325.2
Lb
576 Streptococcus mutans UA159 GCA_000007465.2
434 Streptococcus sobrinus DSM 20742 = ATCC 33478 GCA_000686605.1
433 Streptococcus ratti FA-1 = DSM 20564 GCA_000286075.1
Lc
835 Enterococcus faecalis V583 GCA_000007785.1
789 Streptomyces cinnamoneus GCA_001885705.1
700 Enterococcus rivorum GCA_001742285.1
Ld
664 Bifidobacterium adolescentis ATCC 15703 GCA_000010425.1
525 Bifidobacterium dentium JCM 1195 = DSM 20436 GCA_001042595.1
496 Bifidobacterium angulatum DSM 20098 = JCM 7096 GCA_001025155.1
Le
690 Enterococcus faecalis V583 GCA_000007785.1
643 Streptomyces cinnamoneus GCA_001885705.1
641 Enterococcus rivorum GCA_001742285.1
Lf
632 Lactobacillus gasseri ATCC 33323 = JCM 1131 GCA_000014425.1
605 Lactobacillus hominis DSM 23910 = CRBIP 24.179 GCA_000296835.1
451 Lactobacillus psittaci DSM 15354 GCA_000425905.1
Lg
658 Streptococcus mutans UA159 GCA_000007465.2
481 Streptococcus ratti FA-1 = DSM 20564 GCA_000286075.1
481 Streptococcus sobrinus DSM 20742 = ATCC 33478 GCA_000686605.1
467 Streptococcus gordonii str. Challis substr. CH1 GCA_000017005.1
Lh
765 Staphylococcus epidermidis ATCC 12228 GCA_000007645.1
713 Staphylococcus capitis subsp. capitis GCA_001028645.1
695 Staphylococcus hominis subsp. hominis C80 GCA_000183685.1
Li
758 Staphylococcus epidermidis ATCC 12228 GCA_000007645.1
727 Staphylococcus capitis subsp. capitis GCA_001028645.1
642 Staphylococcus lugdunensis HKU09-01 GCA_000025085.1
Lj

548 Rhodobacter sphaeroides 2.4.1 GCA_000012905.2
483 Rhodobacter sphaeroides ATCC 17025 GCA_000016405.1
374 Pseudorhodobacter ferrugineus DSM 5888 GCA_000420745.1
374 Pseudorhodobacter psychrotolerans GCA_001294535.1
Lk
863 Rhodobacter sphaeroides 2.4.1 GCA_000012905.2
774 Rhodobacter sphaeroides ATCC 17025 GCA_000016405.1
669 Gemmobacter megaterium GCA_900156815.1
Ll
737 Clostridium beijerinckii GCA_000833105.2
720 Clostridium saccharoperbutylacetonicum N1-4_28HMT_29 GCA_000340885.1
675 Clostridium saccharobutylicum DSM 13864 GCA_000473995.1
Lm
506 Bacillus cereus ATCC 14579 GCA_000007825.1
506 Bacillus mycoides GCA_000832605.1
506 Bacillus thuringiensis YBT-1518 GCA_000497525.2
504 Bacillus anthracis str. Ames GCA_000007845.1
504 Bacillus anthracis str. Sterne GCA_000008165.1
504 _5BBacillus thuringiensis_5D serovar konkukian str. 97-27 GCA_000008505.1
486 Bacillus pseudomycoides DSM 12442 GCA_000161455.1
Ln
462 Bifidobacterium adolescentis ATCC 15703 GCA_000010425.1
385 Bifidobacterium angulatum DSM 20098 = JCM 7096 GCA_001025155.1
324 Bifidobacterium callitrichos DSM 23973 GCA_000741175.1
Lo
681 Rhodobacter sphaeroides 2.4.1 GCA_000012905.2
606 Rhodobacter sphaeroides ATCC 17025 GCA_000016405.1
547 Defluviimonas alba GCA_001620265.1
Lp
779 Streptococcus mutans UA159 GCA_000007465.2
587 Streptococcus ratti FA-1 = DSM 20564 GCA_000286075.1
547 Streptococcus gordonii str. Challis substr. CH1 GCA_000017005.1
Lq
491 Lactobacillus gasseri ATCC 33323 = JCM 1131 GCA_000014425.1
431 Lactobacillus hominis DSM 23910 = CRBIP 24.179 GCA_000296835.1
330 Lactobacillus iners DSM 13335 GCA_000160875.1
Lr
796 Enterococcus faecalis V583 GCA_000007785.1
749 Streptomyces cinnamoneus GCA_001885705.1
667 Enterococcus mundtii QU 25 GCA_000504125.1
Ls
506 Staphylococcus epidermidis ATCC 12228 GCA_000007645.1
482 Staphylococcus capitis subsp. capitis GCA_001028645.1
445 Staphylococcus haemolyticus JCSC1435 GCA_000009865.1
445 Staphylococcus hominis subsp. hominis C80 GCA_000183685.1
Lt
753 Rhodobacter sphaeroides 2.4.1 GCA_000012905.2
679 Rhodobacter sphaeroides ATCC 17025 GCA_000016405.1
597 Defluviimonas alba GCA_001620265.1
Lu
836 Streptococcus mutans UA159 GCA_000007465.2
575 Streptococcus ratti FA-1 = DSM 20564 GCA_000286075.1
562 Streptococcus sobrinus DSM 20742 = ATCC 33478 GCA_000686605.1
Lv
709 Streptococcus mutans UA159 GCA_000007465.2
549 Streptococcus ratti FA-1 = DSM 20564 GCA_000286075.1
473 Streptococcus sobrinus DSM 20742 = ATCC 33478 GCA_000686605.1
Lw
844 Bacillus anthracis str. Ames GCA_000007845.1
844 Bacillus anthracis str. Sterne GCA_000008165.1
844 Bacillus cereus ATCC 14579 GCA_000007825.1
844 Bacillus pseudomycoides DSM 12442 GCA_000161455.1
844 _5BBacillus thuringiensis_5D serovar konkukian str. 97-27 GCA_000008505.1
833 Bacillus mycoides GCA_000832605.1
821 Bacillus thuringiensis YBT-1518 GCA_000497525.2
Lx
861 Staphylococcus epidermidis ATCC 12228 GCA_000007645.1
801 Staphylococcus capitis subsp. capitis GCA_001028645.1
751 Staphylococcus haemolyticus JCSC1435 GCA_000009865.1
Ly
401 Clostridium beijerinckii GCA_000833105.2
365 Clostridium puniceum GCA_002006345.1
365 Clostridium saccharoperbutylacetonicum N1-4_28HMT_29 GCA_000340885.1
358 Clostridium butyricum GCA_001456065.2
Lz
855 Streptococcus mutans UA159 GCA_000007465.2

630 Streptococcus ratti FA-1 = DSM 20564 GCA_000286075.1
575 Streptococcus merionis DSM 19192 GCA_000380085.1
LA
593 Rhodobacter sphaeroides 2.4.1 GCA_000012905.2
572 Rhodobacter sphaeroides ATCC 17025 GCA_000016405.1
470 Gemmobacter aquatilis GCA_900110025.1
LB
500 Bifidobacterium adolescentis ATCC 15703 GCA_000010425.1
460 Bifidobacterium dentium JCM 1195 = DSM 20436 GCA_001042595.1
423 Bifidobacterium callitrichos DSM 23973 GCA_000741175.1
LC
676 Clostridium beijerinckii GCA_000833105.2
635 Clostridium saccharoperbutylacetonicum N1-4_28HMT_29 GCA_000340885.1
610 Clostridium butyricum GCA_001456065.2
LD
661 Bacillus anthracis str. Ames GCA_000007845.1
661 Bacillus anthracis str. Sterne GCA_000008165.1
661 Bacillus cereus ATCC 14579 GCA_000007825.1
661 Bacillus pseudomycoides DSM 12442 GCA_000161455.1
661 Bacillus thuringiensis YBT-1518 GCA_000497525.2
661 _5BBacillus thuringiensis_5D serovar konkukian str. 97-27 GCA_000008505.1
645 Bacillus mycoides GCA_000832605.1
624 Bacillus cytotoxicus NVH 391-98 GCA_000017425.1
LE
768 Rhodobacter sphaeroides 2.4.1 GCA_000012905.2
654 Rhodobacter sphaeroides ATCC 17025 GCA_000016405.1
593 Defluviimonas alba GCA_001620265.1
LF
540 Lactobacillus gasseri ATCC 33323 = JCM 1131 GCA_000014425.1
498 Lactobacillus hominis DSM 23910 = CRBIP 24.179 GCA_000296835.1
397 Lactobacillus jensenii GCA_001936235.1
LG
764 Streptococcus mutans UA159 GCA_000007465.2
552 Streptococcus ratti FA-1 = DSM 20564 GCA_000286075.1
525 Streptococcus gordonii str. Challis substr. CH1 GCA_000017005.1
LH
618 Clostridium beijerinckii GCA_000833105.2
610 Clostridium saccharoperbutylacetonicum N1-4_28HMT_29 GCA_000340885.1
591 Clostridium saccharobutylicum DSM 13864 GCA_000473995.1
LI
733 Clostridium beijerinckii GCA_000833105.2
730 Clostridium saccharoperbutylacetonicum N1-4_28HMT_29 GCA_000340885.1
702 Clostridium saccharobutylicum DSM 13864 GCA_000473995.1
LJ
540 Escherichia coli O104_3AH4 str. 2011C-3493 GCA_000299455.1
525 Escherichia coli str. K-12 substr. MG1655 GCA_000005845.2
525 Shigella flexneri 2a str. 301 GCA_000006925.2
510 Escherichia coli IAI39 GCA_000026345.1
LK
620 Streptococcus mutans UA159 GCA_000007465.2
369 Streptococcus ratti FA-1 = DSM 20564 GCA_000286075.1
355 Streptococcus equinus GCA_000964315.1
355 Streptococcus gallolyticus subsp. gallolyticus DSM 16831 GCA_002000985.1
LL
707 Escherichia coli str. K-12 substr. MG1655 GCA_000005845.2
691 Escherichia coli O157_3AH7 str. Sakai GCA_000008865.1
691 Escherichia coli UMN026 GCA_000026325.2
691 Shigella flexneri 2a str. 301 GCA_000006925.2
690 Escherichia coli O104_3AH4 str. 2011C-3493 GCA_000299455.1
LM
692 Rhodobacter sphaeroides 2.4.1 GCA_000012905.2
647 Rhodobacter sphaeroides ATCC 17025 GCA_000016405.1
512 Gemmobacter megaterium GCA_900156815.1
LN
884 Enterococcus faecalis V583 GCA_000007785.1
838 Streptomyces cinnamoneus GCA_001885705.1
759 Enterococcus asini ATCC 700915 GCA_000407365.1
LO
283 Staphylococcus capitis subsp. capitis GCA_001028645.1
283 Staphylococcus epidermidis ATCC 12228 GCA_000007645.1
241 Staphylococcus warneri SG1 GCA_000332735.1
240 Staphylococcus aureus subsp. aureus NCTC 8325 GCA_000013425.1
LP
439 Clostridium puniceum GCA_002006345.1
439 Clostridium saccharoperbutylacetonicum N1-4_28HMT_29 GCA_000340885.1
434 Clostridium beijerinckii GCA_000833105.2

430 Clostridium saccharobutylicum DSM 13864 GCA_000473995.1
LQ
538 Clostridium saccharoperbutylacetonicum N1-4_28HMT_29 GCA_000340885.1
533 Clostridium beijerinckii GCA_000833105.2
495 Clostridium saccharobutylicum DSM 13864 GCA_000473995.1
LR
776 Deinococcus radiodurans R1 GCA_000008565.1
491 Deinococcus deserti VCD115 GCA_000020685.1
464 Deinococcus gobiensis I-0 GCA_000252445.1
LS
479 Clostridium beijerinckii GCA_000833105.2
470 Clostridium saccharoperbutylacetonicum N1-4_28HMT_29 GCA_000340885.1
463 Clostridium saccharobutylicum DSM 13864 GCA_000473995.1
LT
488 Deinococcus radiodurans R1 GCA_000008565.1
272 Deinococcus deserti VCD115 GCA_000020685.1
266 Deinococcus marmoris DSM 12784 GCA_000701405.1
266 Deinococcus swuensis GCA_000800395.1
LU
454 Lactobacillus gasseri ATCC 33323 = JCM 1131 GCA_000014425.1
439 Lactobacillus hominis DSM 23910 = CRBIP 24.179 GCA_000296835.1
319 Lactobacillus iners DSM 13335 GCA_000160875.1
LV
609 Rhodobacter sphaeroides 2.4.1 GCA_000012905.2
547 Rhodobacter sphaeroides ATCC 17025 GCA_000016405.1
458 Gemmobacter aquatilis GCA_900110025.1
LW
799 Enterococcus faecalis V583 GCA_000007785.1
752 Streptomyces cinnamoneus GCA_001885705.1
701 Enterococcus rivorum GCA_001742285.1
LX
765 Enterococcus faecalis V583 GCA_000007785.1
718 Streptomyces cinnamoneus GCA_001885705.1
698 Enterococcus rivorum GCA_001742285.1
LY
521 Lactobacillus gasseri ATCC 33323 = JCM 1131 GCA_000014425.1
488 Lactobacillus hominis DSM 23910 = CRBIP 24.179 GCA_000296835.1
388 Lactobacillus iners DSM 13335 GCA_000160875.1
LZ
786 Escherichia coli str. K-12 substr. MG1655 GCA_000005845.2
784 Escherichia coli O157_3AH7 str. Sakai GCA_000008865.1
779 Escherichia coli UMN026 GCA_000026325.2
M0
946 Bifidobacterium adolescentis ATCC 15703 GCA_000010425.1
761 Bifidobacterium angulatum DSM 20098 = JCM 7096 GCA_001025155.1
738 Bifidobacterium dentium JCM 1195 = DSM 20436 GCA_001042595.1
M1
620 Bifidobacterium adolescentis ATCC 15703 GCA_000010425.1
543 Bifidobacterium breve DSM 20213 = JCM 1192 GCA_001025175.1
535 Bifidobacterium dentium JCM 1195 = DSM 20436 GCA_001042595.1
M2
800 Staphylococcus epidermidis ATCC 12228 GCA_000007645.1
759 Staphylococcus capitis subsp. capitis GCA_001028645.1
684 Staphylococcus hominis subsp. hominis C80 GCA_000183685.1
M3
754 Streptococcus mutans UA159 GCA_000007465.2
646 Streptococcus ratti FA-1 = DSM 20564 GCA_000286075.1
630 Streptococcus gordonii str. Challis substr. CH1 GCA_000017005.1
M4
761 Bifidobacterium adolescentis ATCC 15703 GCA_000010425.1
651 Bifidobacterium dentium JCM 1195 = DSM 20436 GCA_001042595.1
613 Bifidobacterium angulatum DSM 20098 = JCM 7096 GCA_001025155.1
M5
686 Staphylococcus epidermidis ATCC 12228 GCA_000007645.1
657 Staphylococcus capitis subsp. capitis GCA_001028645.1
593 Staphylococcus aureus subsp. aureus NCTC 8325 GCA_000013425.1
M6
811 Streptococcus mutans UA159 GCA_000007465.2
610 Streptococcus ratti FA-1 = DSM 20564 GCA_000286075.1
546 Streptococcus macacae NCTC 11558 GCA_000187995.3
M7
638 Enterococcus faecalis V583 GCA_000007785.1
591 Streptomyces cinnamoneus GCA_001885705.1
538 Enterococcus rivorum GCA_001742285.1
M8
271 Rhodobacter sphaeroides 2.4.1 GCA_000012905.2

260 Rhodobacter sphaeroides ATCC 17025 GCA_000016405.1
209 Gemmobacter aquatilis GCA_900110025.1
M9
652 Enterococcus faecalis V583 GCA_000007785.1
605 Streptomyces cinnamoneus GCA_001885705.1
564 Enterococcus faecium DO GCA_000174395.2
Ma
717 Clostridium beijerinckii GCA_000833105.2
688 Clostridium saccharoperbutylacetonicum N1-4_28HMT_29 GCA_000340885.1
673 Clostridium saccharobutylicum DSM 13864 GCA_000473995.1
Mb
546 Rhodobacter sphaeroides 2.4.1 GCA_000012905.2
502 Rhodobacter sphaeroides ATCC 17025 GCA_000016405.1
429 Pseudorhodobacter ferrugineus DSM 5888 GCA_000420745.1
429 Pseudorhodobacter psychrotolerans GCA_001294535.1
429 Pseudorhodobacter wandonensis GCA_001202035.1
Mc
619 Rhodobacter sphaeroides 2.4.1 GCA_000012905.2
550 Rhodobacter sphaeroides ATCC 17025 GCA_000016405.1
454 Gemmobacter aquatilis GCA_900110025.1
Md
443 Streptococcus mutans UA159 GCA_000007465.2
367 Streptococcus anginosus C238 GCA_000463505.1
361 Streptococcus merionis DSM 19192 GCA_000380085.1
Me
543 Bifidobacterium adolescentis ATCC 15703 GCA_000010425.1
420 Bifidobacterium dentium JCM 1195 = DSM 20436 GCA_001042595.1
419 Bifidobacterium angulatum DSM 20098 = JCM 7096 GCA_001025155.1
Mf
507 Staphylococcus epidermidis ATCC 12228 GCA_000007645.1
486 Staphylococcus haemolyticus JCSC1435 GCA_000009865.1
481 Staphylococcus capitis subsp. capitis GCA_001028645.1
Mg
517 Bifidobacterium adolescentis ATCC 15703 GCA_000010425.1
364 Bifidobacterium dentium JCM 1195 = DSM 20436 GCA_001042595.1
353 Bifidobacterium angulatum DSM 20098 = JCM 7096 GCA_001025155.1
Mh
783 Enterococcus faecalis V583 GCA_000007785.1
783 Streptomyces cinnamoneus GCA_001885705.1
736 Enterococcus asini ATCC 700915 GCA_000407365.1
723 Enterococcus italicus DSM 15952 GCA_000185365.1
Mi
757 Escherichia coli O157_3AH7 str. Sakai GCA_000008865.1
757 Escherichia coli str. K-12 substr. MG1655 GCA_000005845.2
748 Escherichia coli IAI39 GCA_000026345.1
737 Escherichia coli UMN026 GCA_000026325.2
Mj
732 Clostridium beijerinckii GCA_000833105.2
698 Clostridium saccharoperbutylacetonicum N1-4_28HMT_29 GCA_000340885.1
664 Clostridium puniceum GCA_002006345.1
Mk
668 Enterococcus faecalis V583 GCA_000007785.1
622 Streptomyces cinnamoneus GCA_001885705.1
565 Enterococcus faecium DO GCA_000174395.2
Ml
645 Bacillus anthracis str. Ames GCA_000007845.1
645 Bacillus anthracis str. Sterne GCA_000008165.1
645 Bacillus cereus ATCC 14579 GCA_000007825.1
645 Bacillus mycoides GCA_000832605.1
645 Bacillus pseudomycoides DSM 12442 GCA_000161455.1
645 _5BBacillus thuringiensis_5D serovar konkukian str. 97-27 GCA_000008505.1
632 Bacillus thuringiensis YBT-1518 GCA_000497525.2
558 Bacillus cytotoxicus NVH 391-98 GCA_000017425.1
Mm
735 Streptococcus mutans UA159 GCA_000007465.2
498 Streptococcus ratti FA-1 = DSM 20564 GCA_000286075.1
460 Streptococcus ferus DSM 20646 GCA_000372425.1
Mn
845 Enterococcus faecalis V583 GCA_000007785.1
804 Streptomyces cinnamoneus GCA_001885705.1
749 Enterococcus rivorum GCA_001742285.1
Mo
819 Deinococcus radiodurans R1 GCA_000008565.1
513 Deinococcus marmoris DSM 12784 GCA_000701405.1
502 Deinococcus deserti VCD115 GCA_000020685.1
